# Supplementary material for: The ancestral flower of angiosperms and its early diversification
Source: Nat Commun. 2017 Aug 1;8:16047. doi: 10.1038/ncomms16047 (PMC5543309; doi:10.1038/ncomms16047)
Supplement: Supplementary Information [file ncomms16047-s1.pdf]

Title of file for HTML: Supplementary Information
Description: Supplementary Figures, Supplementary Tables, Supplementary Discussion, Supplementary Methods and Supplementary References

Title of file for HTML: Supplementary Data 1
Description: This file contains a summary of reconstructed ancestral states and associated probabilities for 15 key nodes of angiosperms, according to each analysis.

Title of file for HTML: Supplementary Data 2
Description: This file contains a summary of reconstructed ancestral combined states and associated probabilities for 15 key nodes of angiosperms, according to each pairwise correlation analysis.

Title of file for HTML: Supplementary Data 3
Description: Maximum clade credibility tree from the BEAST analysis of the A series.

Title of file for HTML: Supplementary Data 4
Description: Maximum clade credibility tree from the BEAST analysis of the B series.

Title of file for HTML: Supplementary Data 5
Description: Maximum clade credibility tree from the BEAST analysis of the C series.

Title of file for HTML: Supplementary Data 6
Description: Maximum clade credibility tree from the BEAST analysis of the D series.

Title of file for HTML: Supplementary Data 7
Description: Maximum clade credibility tree from the BEAST analysis of the E series.

Title of file for HTML: Supplementary Data 8
Description: Maximum clade credibility tree from the BEAST analysis of the A200 series.

Title of file for HTML: Supplementary Data 9
Description: Maximum clade credibility tree from the BEAST analysis of the B200 series.

Title of file for HTML: Supplementary Data 10
Description: Maximum clade credibility tree from the BEAST analysis of the C200 series.

Title of file for HTML: Supplementary Data 11
Description: Maximum clade credibility tree from the BEAST analysis of the D200 series.

Title of file for HTML: Supplementary Data 12
Description: Maximum clade credibility tree from the BEAST analysis of the E200 series.

Title of file for HTML: Supplementary Data 13
Description: This file contains: 1) the complete list of morphological data records and associated references extracted from the PROTEUS database; 2) a list of explicit character transformations; 3) a summary of the original data (as recorded) presented as a matrix; 4) a summary of the transformed data (as analyzed) presented as a matrix.

Title of file for HTML: Supplementary Data 14

Description: Explicit MP and ML ancestral state reconstructions for all floral traits for the A tree series. Note that these results may differ from those of rjMCMC (Bayesian). For a complete summary of results for key nodes across all analyses, see Supplementary Data 1.

Title of file for HTML: Supplementary Data 15
Description: Explicit MP and ML ancestral state reconstructions for all floral traits for the B tree series. Note that these results may differ from those of rjMCMC (Bayesian). For a complete summary of results for key nodes across all analyses, see Supplementary Data 1.

Title of file for HTML: Supplementary Data 16
Description: Explicit MP and ML ancestral state reconstructions for all floral traits for the C tree series. Note that these results may differ from those of rjMCMC (Bayesian). For a complete summary of results for key nodes across all analyses, see Supplementary Data 1.

Title of file for HTML: Supplementary Data 17
Description: Explicit MP and ML ancestral state reconstructions for all floral traits for the D tree series. Note that these results may differ from those of rjMCMC (Bayesian). For a complete summary of results for key nodes across all analyses, see Supplementary Data 1.

Title of file for HTML: Supplementary Data 18
Description: Explicit MP and ML ancestral state reconstructions for all floral traits for the E tree series. Note that these results may differ from those of rjMCMC (Bayesian). For a complete summary of results for key nodes across all analyses, see Supplementary Data 1.

Title of file for HTML: Supplementary Data 19
Description: Explicit MP and ML ancestral state reconstructions for all floral traits for the A200 tree series. Note that these results may differ from those of rjMCMC (Bayesian). For a complete summary of results for key nodes across all analyses, see Supplementary Data 1.

Title of file for HTML: Supplementary Data 20
Description: Explicit MP and ML ancestral state reconstructions for all floral traits for the B200 tree series. Note that these results may differ from those of rjMCMC (Bayesian). For a complete summary of results for key nodes across all analyses, see Supplementary Data 1.

Title of file for HTML: Supplementary Data 21
Description: Explicit MP and ML ancestral state reconstructions for all floral traits for the C200 tree series. Note that these results may differ from those of rjMCMC (Bayesian). For a complete summary of results for key nodes across all analyses, see Supplementary Data 1.

Title of file for HTML: Supplementary Data 22
Description: Explicit MP and ML ancestral state reconstructions for all floral traits for the D200 tree series. Note that these results may differ from those of rjMCMC (Bayesian). For a complete summary of results for key nodes across all analyses, see Supplementary Data 1.

Title of file for HTML: Supplementary Data 23
Description: Explicit MP and ML ancestral state reconstructions for all floral traits for the E200 tree series. Note that these results may differ from those of rjMCMC (Bayesian). For a complete summary of results for key nodes across all analyses, see Supplementary Data 1.

Title of file for HTML: Supplementary Movie 1

Description: Short video showing the 3D model of the reconstructed ancestral flower (Fig. 1) rotating along various angles.

Title of file for HTML: Peer Review File
Description:

Supplementary Methods

Definition and explanation of characters

This section provides the criteria for how we defined and treated each floral character analyzed in this study. All floral characters were recorded in the PROTEUS database[1]. The complete list of records, each of them linked to an explicit reference, and the final matrix used in all analyses are provided in Supplementary Data 13. In total, our analyses are based on 27 floral characters, which are derived from 21 distinct primary characters (11 discrete and 10 continuous).

Primary characters, either qualitative/discrete (D1) or quantitative/ordinal/continuous (C1), represent characters as recorded from primary literature sources. *Secondary characters* used in the analyses are all discrete (D2) and were derived by reduction of states from discrete primary characters (D2d), or from modification of continuous primary characters into discrete classes of variation (D2c). We acknowledge that some quantitative characters, such as number of organs, may be discrete in their variation rather than continuous, but refer to them as continuous for the sake of convenience. Each character was assigned a number within a range that refers to organ type: 100-199 for general floral characters, 200-299 for perianth characters, 300-399 for androecial characters, and 400-499 for gynoecial characters. Primary characters have a number only (e.g., 100), while secondary characters share the number of the primary character from which they are derived and, in addition, are provided with a letter suffix (e.g., 100_A). In several cases, we have used various alternative secondary characters that are based on the same primary character (e.g., 100_A, 100_B). This allowed us to address different questions as well as to test the effect of many vs. two or three character states on the results from model selection analyses, parameter estimations, and ancestral state reconstructions.

Scoring philosophy

There is no general consensus on the optimal way to score phenotypic data, especially with respect to discrete characters. In particular, the appropriate number of character states may depend on the type of analysis and/or the type of questions being asked. Thus, we have developed the two-step approach to character scoring outlined above, whereby we separate primary characters for scoring the observed data without too many assumptions, from secondary characters used in a given analysis. This approach also provides the option for future users to query the primary scorings and derive scoring schemes that fit their own needs.

Rationale for reducing the number of character states. In general, we have aimed to reduce the number of character states of discrete secondary characters (D2) as much as possible. While a large number of character states is not necessarily a problem with parsimony optimization (at least as long as the sample size is large), this becomes a serious problem with maximum likelihood or Bayesian analyses based on probabilistic models. The latter types of analyses require much greater computational power as the number of transition rate parameters for the general Markov model with all rates different increases very fast. The number of such parameters for a k-state character is equal to k (k – 1) meaning that a 3-state character has six transition rates, whereas a 6-state character has already 30. Even

with a large data set such as ours, we may not have enough data to accurately estimate these transition rates, particularly for rare character states. Model selection can partly correct for this problem by constraining two or more parameters to be equal or zero, and by the use of metrics such as the Akaike Information Criterion (AIC) to choose the best-fit model for a given analysis[2]. In spite of these aids, the size of model space increases even more drastically with additional character states. For instance, a 3-state character has 876 distinct Markov models, while a 4-state character has 27,644,436 distinct models. Even Bayesian strategies developed to explore model space, such as reversible-jump Markov Chain Monte Carlo[3], may not have enough power to explore the entire model space within a reasonable computational time frame. For these reasons, we have strived to keep the number of character states low in discrete secondary characters (used in analyses) and have occasionally excluded (i.e., treated as missing data) rare or exceptional character states (e.g., hexamery). However, when doing so, we also maintained the full version of the same character with all original character states included (e.g., 232_A and 232_B).

Inapplicable and missing data. None of the currently available methods for character analysis and ancestral state reconstruction can distinguish inapplicable data from missing data. Scoring inapplicable data as a separate character state has several disadvantages: (1) it adds an extra state to the model (see above); (2) it adds redundancy to the data set (e.g., adding a “perianth absent” state each to the characters perianth phyllotaxis, merism, and differentiation); and (3) it transforms the question asked by the analysis. For instance, it is not the same to ask how spiral and whorled flowers evolved from each other in a 2-state character analysis as to ask how flowers with no perianth, a spiral perianth, or a whorled perianth evolved from each other in a 3-state character analysis. For these reasons, all inapplicable data were treated as missing data for this project.

Polymorphic data. Whenever two or more states co-exist in any given species (either due to intraspecific variation, actual co-existence in the same individual, or intermediate states), we scored them as polymorphic data. Not all methods for ancestral state reconstruction can take polymorphic data into account. However, all methods we used in this paper allowed polymorphic data (incl. maximum likelihood as implemented in the R package corHMM[4] and Bayesian analysis as implemented in BayesTraits[5]).

Sexual dimorphism. It is not uncommon that unisexual flowers differ in specific aspects of their morphology. For instance, in the genus *Buxus*, male flowers have a whorled perianth whereas female flowers have a spiral perianth[6]. Accounting properly for such differences would be interesting in lower-level studies focusing on a particular unisexual clade, for instance by using separate characters for male and female flowers or by adding special characters to record sexual dimorphism. However, for this large-scale project, such an approach is neither suitable nor relevant. Therefore, we scored all cases of sexual dimorphism of nonsexual characters as polymorphic data. For instance, in *Buxus*, we scored perianth phyllotaxis as both whorled and spiral. For sexual characters (androecium and gynoecium), sexual dimorphism is usually directly linked to unisexuality. Thus, we scored androecial characters (e.g., number of stamens) of unisexual species based on their male flowers, and gynoecial characters (e.g., number of carpels) of unisexual species based on their female flowers.

Continuous characters. For continuous (quantitative) characters (C1), we scored either a value or a range of values (as minimum and maximum), depending on intraspecific variation and accuracy of measurement. For instance, a flower with perianth parts fused more or less along 50% of their length may be scored with a value range from 0.4 to 0.6. When compiling discrete secondary characters from such a continuous character (D2c), the entire range of values scored in each species is taken into account.

Congenital vs. postgenital fusion. In theory, it would be interesting and important to distinguish between congenital and postgenital fusion[6]. In practice, this is mostly impossible unless adequate developmental data are available, which was the case only for a fraction of the species in our sample. Therefore, all of the fusion characters in this paper include both congenital and postgenital fusion.

Developmental evidence. Detailed observations of floral organs throughout floral development may inform us on their homology and the processes that lead to their final shape and architecture at anthesis. However, such data are available only in a small number of angiosperm species. In addition, some characters may change during ontogeny. For instance, perianth parts may be initiated spirally but be arranged in whorls at anthesis[7,8]. For this project, we were careful to only record characters at anthesis so that all of our characters could be comparable and scorable across our entire sample. This does not mean that we ignored solid, morphological studies including developmental observations, but we made sure to score only data as observed in the later (near-anthetic) stages of the floral developmental sequence.

General floral characters

100. Sex (D1). Flowers can be either bisexual (hermaphrodite) or unisexual. Here, we distinguish among the many possible ways to be unisexual, depending on whether sterile organs of the opposite sex (staminodes or carpellodes) are found in flowers of a given sex, whether male and female flowers are found on the same plant (monoecy) or separate plants (dioecy), as well as the various intermediate combinations that exist (e.g., androdioecy, gynomonoecy). There are many ways in which this character could be analyzed, depending on the questions asked. Here, we are interested in reconstructing the sex of ancestral flowers rather than the sex of individual plants (i.e., sexual systems). Therefore, we do not distinguish among different sexual systems such as monoecy and dioecy in the two following secondary characters.

100_A. Functional sex of flowers (D2d). Here we treat all functionally unisexual flowers as unisexual. These may include structurally bisexual flowers (e.g., male flowers with carpellodes and female flowers with staminodes) that are functionally unisexual. Co-occurrence of functionally unisexual and bisexual flowers (e.g., androdioecy, gynomonoecy) is polymorphic and treated as missing data here. *Amborella trichopoda*, with structurally male and bisexual flowers[9] but functionally unisexual dioecious plants (i.e., the bisexual flowers are functionally female flowers), is treated here as unisexual.

100_B. Structural sex of flowers (D2d). Here we treat functionally unisexual flowers as either bisexual, when organs of the opposite sex are observed, or as unisexual, when no such remnants are found. Co-occurrence of structurally unisexual and bisexual flowers is polymorphic and treated as missing data here. For instance, *Amborella trichopoda*, with structurally male and bisexual flowers but acting

functionally unisexual dioecious (i.e., the bisexual flowers are functionally female flowers), is polymorphic for this character.

102. Ovary position (D1). The ovary is the part of the gynoecium where the ovules are produced. The ovary may be located on the receptacle and thus be positioned above the insertion level of the remaining floral organs (i.e., the ovary is superior and the flower is hypogynous). Alternatively, the ovary may be embedded in the receptacle and therefore be located below the insertion level of the remaining floral organs (i.e., the ovary is inferior and the flower is epigynous). Flowers with a hypanthium may either have a superior ovary (perigyny; e.g., many Rosaceae) or an inferior ovary (epiperigyny)[10]. It is also possible that the ovary is inferior to a certain degree only, such as half-inferior, if the receptacle is surrounding the ovary to its mid-level. Here we recorded the ovary position either as superior, inferior, or one of the following intermediate states: ¼ inferior or less, half-inferior, ¾ inferior or more.

102_B. Ovary position (binary) (D2d). Here we treat ovary position as a binary character, distinguishing only superior and inferior ovaries, and conservatively including all intermediate inferior states (incl. half-inferior) in the character state inferior.

Perianth

There is considerable variation in the number and morphology of sterile organs surrounding the fertile organs across angiosperms. Some species do not have a perianth at all (e.g., *Chloranthus*), while others have a simple perianth of morphologically similar organs arranged either in a single whorl (e.g., *Myristica*), in two whorls (e.g., *Lilium*), or in a continuous spiral (e.g., *Amborella*), and the majority of species have a differentiated perianth commonly comprising an outer whorl of sepals and an inner whorl of petals (e.g., *Geranium*). In addition to this structural variation, it is generally acknowledged that perianth organs have different evolutionary origins, depending on the lineage considered[6]. Thus, petals are probably not homologous across angiosperms as a whole, which is why we decided not to use distinct characters for sepals and petals for this angiosperm-wide study. Instead, we only use broadly defined perianth characters to compare and reconstruct the evolutionary history of the perianth at the functional level. We acknowledge that at the developmental or genetic level the perianth as such may not have a single evolutionary origin in the angiosperms, but current knowledge on perianth organ homology is too fragmentary to use it as a basis for defining perianth characters across all angiosperms.

Perianth vs. staminodes. Here, we define the perianth at the functional level as the collection of all sterile organs surrounding the reproductive floral organs. In some species, sterile organs resembling fertile stamens are present in addition to the fertile stamens. These organs are generally referred to as staminodes and may be outer staminodes, that is, inserted outside the fertile stamens (e.g., *Galbulimima*), inner staminodes, inserted between the fertile stamens and the gynoecium (e.g., *Degeneria*), or staminodes intermixed with fertile stamens of the same whorl or series (e.g., *Penstemon*)[11–14]. In many cases, such organs are morphologically more similar to fertile stamens than to typical perianth organs; hence, there is a general consensus that they should be considered as part of the androecium. However, some taxa challenge the traditional boundary between the perianth and the androecium[15]. For instance, staminodes may be petaloid and take over the attractive function of the perianth (e.g., *Canna*). Or there may exist unique floral organs

between the perianth and the androecium that resemble neither typical perianth organs nor fertile stamens (e.g., funnel-shaped nectar leaves of *Helleborus*). Or in exceptional cases, fertile stamens and peripheral sterile organs may both be petaloid and be morphologically nearly identical except for the presence of the pollen sacs (e.g., *Galbulimima*). In such cases, we decided to treat as members of the perianth only sterile organs that are morphologically distinct from the fertile stamens. Thus, we treat the "petals" of *Nuphar* as part of the perianth, the staminodes of *Canna* as part of the androecium, the nectar leaves of *Helleborus* (not sampled in this data set) as part of the perianth (together with the sepaloid tepals), and the peripheral organs of *Galbulimima* as part of the androecium[14].

Perianth vs. bracts. In addition, the boundary between outer perianth organs and extrafloral organs such as bracts and prophylls is problematic in some taxa and there is no unique criterion that allows delimiting the flower from preceding organs. Here we follow published hypotheses on a case-by-case basis. For example, we treat the epicalyx of Malvaceae and the calyptra of *Eupomatia* as bracts[16,17], but the pair of deciduous organs enclosing the bud in *Papaver* as outer perianth organs[18,19].

Interpretation of hypanthia and inferior ovaries. Floral cups, or hypanthia, are common in angiosperms. In most cases, these correspond to concave structures of undifferentiated tissue, on the margins of which both the perianth and the androecium are inserted, and at the base of which the gynoecium is inserted. Usually, hypanthia are interpreted to be expanded receptacles[20]. Here we follow this interpretation and consider perianth parts and stamens to begin on the rim of the hypanthium, not at its base, and therefore not necessarily all fused at the base. Similarly, when ovaries are inferior, we consider both the perianth and the androecium to start above the ovary, not at its base, and therefore not necessarily all fused along the length of the ovary.

Double positions. Organ doubling often happens when there is a change from broader to narrower organs, or when the meristem is elongated[12,21]. This process appears to be restricted to whorled organs and will cause a change in merism: typically, the number of parts in a given whorl is doubled. This is the common interpretation, for instance, to explain the octamerous inner perianth whorls of *Nymphaea* or the hexamerous androecium of *Alisma*, *Aristolochia*, and *Cabomba*. It has also been proposed as an explanation for the peculiar androecium of Fumarioideae[22] and the inner (tetramerous) stamen whorl of Brassicaceae[23]. However, no consensus exists for these interpretations. Therefore, we opted for an agnostic approach when scoring perianth or androecium merism in such taxa and simply counted the number of organs in each distinct whorl.

Special cases. We treat the pappus of Asteraceae as a highly specialized calyx[24] and thus scored two perianth whorls and marked perianth differentiation in all species of Asteraceae. However, because it is not possible to count the number of original sepals in a pappus, we scored the number of perianth parts as five (based on the corolla only) in members of this family. We treat the lodicules of Poaceae as perianth parts, but only when they are clearly visible at anthesis[25,26]. The calyx of Apiaceae is, in many cases, small and only present as a toothed or non-toothed, truncate rim on top of the ovary; in a few taxa the calyx has been lost entirely. We treat the perianth in Apiaceae as two-whorled if a calyx rim of any form is present. In a non-toothed, truncate calyx rim we interpret the sepals as entirely united and at the same time it is not possible to determine the number of sepals and as a consequence the character 201 'Number of perianth parts' is inapplicable. The cyathium of Euphorbiaceae is

interpreted as an inflorescence of perianthless unisexual flowers rather than a flower[27]. Pseudanthia, in general, are treated as inflorescences, not flowers. Reproductive structures of Hydatellaceae represent a special challenge to interpretation and have been termed by some authors as 'nonflowers'[28]. Here we do not opt for either their interpretation as inflorescences of unisexual flowers or very peculiar flowers with an inversion of the androecium and the gynoecium. Instead, we chose to not score (i.e., leave as missing data) characters that depend on the interpretation of the reproductive structures of Hydatellaceae. Flowers of Proteaceae have been interpreted as either tetramerous or dimerous. The latter is supported in part by the fact that the four stamens are always opposite the four tepals. However, this situation could equally be interpreted as loss of a perianth or androecial whorl. Although there is some indication that the four tepals develop as two decussate pairs in some species[29], this does not appear to be the case of stamens, and the perianth of all flowers of Proteaceae clearly appear as a single whorl of four tepals at anthesis[30]. Thus, we treat the perianth and androecium of Proteaceae as tetramerous.

201. Number of perianth parts (C1). In this character, we scored the total number of perianth parts, including sepals, petals, or any form of tepal. A value of zero was scored when the perianth is absent. In flowers with perianth whorls fused along their complete length (e.g., *Convolvulus*), counting the number of perianth organs may be difficult or challenging. This is traditionally done based on merism (e.g., if a calyx has 5 distinct sepals and the corolla is entirely fused, then the corolla is often interpreted to consist of five fused petals), anatomy (e.g., number of vascular traces), development (e.g., number of primordia), or comparison with closely related taxa. This character is linked with several other aspects of perianth structure, in particular phyllotaxis, merism, and number of whorls, each of which was also recorded as a separate character and analyzed on its own (see below). Our main goal with this character was to reconstruct the number of perianth parts in key nodes of the angiosperm tree taking into account these combined characters and, in whorled clades, contrast it to inferences based on merism and number of whorls. The total number of perianth parts also has the advantage of being applicable to all flowering plants, including those with spiral phyllotaxis.

201_A. Perianth presence (D2c). Simplest discretization of the number of perianth parts to reconstruct the evolution of perianth absence (0 parts) vs. presence (1 or more parts).

201_B. Number of perianth parts (3-state) (D2c). Here we discretized the number of perianth parts into three character states. Flowers with a perianth consisting of a single or two parts are extremely rare in the angiosperms and were treated here as the same state as perianths consisting of three, four, or five parts, which includes all single whorls of merism up to five. Perianths of six to ten parts were pooled together in a state that includes all double whorls of merism between three and five. Last, all numbers above ten were treated together (usually corresponding to spiral perianths or rare perianths of four or more whorls).

201_C. Number of perianth parts (binary) (D2c). Here we experimented with a different discrete version of this character, on the one hand, pooling together all perianths with six or fewer parts (incl. some rare spirals with few tepals, single whorls of merism up to six, double dimerous or trimerous whorls, and triple dimerous whorls) and, on the other hand, pooling all perianths with more than seven parts (incl. most

spirals, double tetramerous or pentamerous whorls, and most perianths with more than two whorls).

230. Perianth phyllotaxis (D1). Perianth parts may be organized in one or more whorls or along a continuous spiral, usually with wide divergence angles more or less equal to 137.5°[31–34]. Less frequently, perianth phyllotaxis may be irregular[35]. Perianth phyllotaxis at anthesis may differ from phyllotaxis of perianth part primordia at their inception and it is not uncommon that spirally initiated perianths become whorled later through development[7,8,34,36–38]. Here we score perianth phyllotaxis only at anthesis because developmental data are lacking for most species in our data set.

Perianth phyllotaxis at anthesis is not always easy to recognize. The first criterion, when perianth parts are in sufficient number, is the pattern of parastichies and presence or absence of orthostichies[32,36]. However, this criterion cannot be used when perianth parts are few (e.g., five) and the insertion of parts along a circle at anthesis does not inform us on whether phyllotaxis is whorled or spiral or irregular. A more universal criterion lies in the divergence angles between successively initiated parts, which usually approximates 137.5° (golden angle) in Fibonacci spiral flowers[39]. However, it may be difficult or impossible to recognize successively initiated perianth parts in an anthetic flower. If we define a series as a set of more or less similar parts occupying the space of and arranged more or less in a circle, we may then use the number of distinct distances between two adjacent parts in a series as a criterion to identify phyllotaxis. Whorled series only have one identical distance between parts, whereas spiral series are typically expected to show two distinct distances: a short distance and a longer distance[40]. In addition, most spiral series are characterized by a Fibonacci number of parts (e.g., 3, 5, 8) although individual variations are common so that this number is rarely completely fixed in spiral flowers. Thus, series with a consistent number of parts of 4 or 6 are less likely to be spiral. Furthermore, developmental data, when available, may still inform us on phyllotaxis at anthesis because spiral phyllotaxis never appears to result from simultaneous, whorled initiation (but note that the contrary is not true). Last, a potentially very confusing situation may arise when a perianth has two or more distinct, well differentiated series of identical number of parts. In such cases, whorled perianths are generally expected to show perfect alternation of successive whorls in addition to equal angles and distances within each whorl, whereas spiral perianths are expected to display less exact and less regular alternation of successive series and are expected to show the above pattern of two distances within each series caused by the golden angle. Such is the case of *Ranunculus acris* (Ranunculaceae), a species commonly assumed to have a whorled perianth, but which in fact has a spiral perianth [41]. Although these rules can help us clarify difficult situations, one should note that they are not universal: zygomorphy and double positions may superpose on all types of phyllotaxis and create more complex patterns. In particular, unequal distances and divergence angles are expected at anthesis in whorled flowers that are also zygomorphic or characterized by double positions.

Scoring perianth phyllotaxis is further complicated by the fact that many eudicots appear to have a more or less spiral calyx and a whorled corolla[20]. However, this has not been thoroughly documented yet across the clade and most descriptions and illustrations of these taxa do not allow us to distinguish easily between a spiral and a whorled calyx, even with the rules outlined above. Therefore, in this study, we have scored perianth phyllotaxis of most eudicots based on phyllotaxis of the inner perianth series (i.e., the corolla).

230_A. Perianth phyllotaxis (binary) (D2d). Perianth phyllotaxis treated as a binary character: spiral vs. whorled (rare cases of irregular phyllotaxis treated as missing data).

231. Number of perianth whorls (C1). The number of perianth whorls was recorded as a continuous character (with integer values of 1 and above). Not applicable when perianth phyllotaxis is spiral or irregular or when the perianth is absent.

231_A. Number of perianth whorls (D2c). Simple discretization of the number of perianth whorls as a three-state character: one, two, or more than two whorls.

232. Perianth merism (C1). Here we define perianth merism as the number of perianth parts in each whorl, recorded as a continuous character (with integer values of 1 and above). Not applicable when perianth phyllotaxis is spiral or irregular or when the perianth is absent.

232_A. Perianth merism (4-state) (D2c). Discretization of perianth merism as a four-state character, distinguishing dimerous, trimerous, tetramerous, and pentamerous perianths. We ignore (treat as missing data) other merisms (e.g., hexamery, octomery) because these states are comparatively very rare and caused reconstruction artefacts (due to low statistical power) in early analyses of this data set.

232_B. Perianth merism (3-state) (D2c). Discretization of perianth merism as a three-state character: trimerous, tetramerous, or pentamerous. Here we treat dimery as missing data.

234. Perianth differentiation (D1). There are many ways in which perianth organs may look different from each other in a given flower. Typically, outer perianth parts are sepaloid and protect the other floral organs during floral development, while inner organs are often petaloid and play a role in pollinator attraction[12]. However, it is also possible that all parts are either sepaloid or petaloid but remain differentiated in shape, size, and/or texture. In case of spiral perianths, differentiation may be continuous (i.e., gradual), whereby two successively initiated organs are very similar or only slightly different, while the outermost and innermost organs at both ends of the spiral are very different from each other (e.g., *Chimonanthus*). Here, we broadly define differentiation as any form of such differences, but record these various situations as separate character states. In contrast, we score all undifferentiated perianths (i.e., with all organs alike) in the same character state, regardless of phyllotaxis and number of whorls. In the special case of perianths consisting of a single whorl, we have decided to score them as undifferentiated rather than treat them as inapplicable because we aimed at a broadly comparable character. Thus, our perianth differentiation character may be seen as both functional (the parting vs. sharing of functions among perianth parts) and developmental (the expression of a genetic program for different forms of perianth parts vs. a single program for a single type of perianth part morphology). Within-whorl differentiation, whereby organs of the same whorl take different forms, is common in zygomorphic flowers (e.g., Balsaminaceae, Fabaceae, Orchidaceae) but is not taken into account here. This character is not applicable when the perianth is absent.

234_A. Perianth differentiation (binary) (D2d). Simplification of the character above as a binary character, distinguishing undifferentiated (or nearly so) from markedly differentiated perianths. Here we treated all forms of continuous differentiation as undifferentiated.

204. Fusion of perianth (C1). Fusion of perianth organs (congenital or postgenital) at anthesis, recorded on a continuous scale, from 0 (free parts) to 1 (parts fused along their entire length). Partial fusion was recorded using an approximate number between these two extremes (e.g., 0.1 corresponds to basal fusion, 0.5 to fusion along half of the length of perianth parts). In case of multiple whorls, we recorded within-whorl fusion in this character. For example, if organs within each whorl are fused along their entire length, we have recorded a value of 1 here. In cases where organs of two whorls are united into a common tubular structure, such as frequently observed in monocots (e.g., *Polygonatum*), we have also recorded this as fusion in this character. If the two (or more) whorls differed in their extent of fusion, we have recorded this character as a range of values. For example, if the calyx is only basally fused, up to 10% of its length, but the corolla is entirely fused, we have recorded a range of 0.1 to 1 here. Our rationale for this was to provide a general character that allows comparison of fusion among all flowering plants, regardless of perianth architecture. Finally, we acknowledge that perianth parts may be fused very early in their development but then appear to be free at anthesis (i.e., early sympetaly as in Apiaceae for instance[42]). Given that developmental studies are lacking for most species in our data set, we treat such flowers as having free perianth parts in order to treat all species in a comparable way (i.e., at anthesis).

204_A. Fusion of perianth (D2c). Here we discretized fusion of the perianth as a binary character (free vs. fused) with a threshold at 5%. For example, a perianth with parts fused up to 4% of their length will be treated as free, whereas a perianth with parts fused along 10% of their length will be treated as fused (both cases would traditionally be referred to as basal fusion). The rationale for the 5% threshold is that organs of the same whorl or even of two successive whorls often appear to have a short common base (which may be interpreted as part of the receptacle) and accordingly are often described as “basally connate” or “basally adhering” without being clearly fused. In addition, it appears reasonable to treat perianths fused for only less than 5% of their length in the same way as secondarily free perianths (early sympetaly) mentioned above.

207. Symmetry of perianth (D1). There are many ways in which flowers can be zygomorphic (monosymmetric, with a single plane of bilateral symmetry). Here we record perianth symmetry, regardless of androecium or gynoecium symmetry; thus the character is not applicable when the perianth is absent. We distinguish strict actinomorphy (i.e., polysymmetry, with three or more planes of bilateral symmetry) from spiral actinomorphy. In addition, disymmetry (two orthogonal planes of bilateral symmetry; e.g., Papaveraceae) and asymmetry are treated here as separate character states. As for the fusion of the perianth, this character is applied to the perianth as a whole. In case of flowers with two or more perianth whorls, species were scored as actinomorphic if all whorls are actinomorphic and as zygomorphic if one or more whorls are zygomorphic.

207_A. Symmetry of perianth (binary) (D2d). Here we treat perianth symmetry as a binary character, merging strict and spiral actinomorphy, and ignoring disymmetry (which may be considered intermediate between actinomorphy and zygomorphy[43]) and asymmetry. Accordingly, the two latter states are treated as missing data.

Androecium

The androecium encompasses the male reproductive organs of the flower and is sometimes composed of both fertile and sterile stamens (i.e., staminodes).

Staminodes are widely distributed taxonomically and occur in at least one species in 32.5% of the angiosperm families[13]. Although we are not analyzing the morphological diversity of staminodes, the presence of staminodes is taken into account for characters that are related to the overall androecial organization. Thus, they are included when recording the number of androecium whorls, androecium phyllotaxis as well as androecial merism, but not when recording number of fertile stamens, filament shape, and the various anther characters. The presence of outer petaloid staminodes is also discussed in the section on the definition of the perianth (see above).

Stamen shape is highly variable in angiosperms, including the shape of both the anther and the filament. In order to compare these variable structures across angiosperms, we work with the following simple definition of stamen morphology: the part of a stamen where pollen sacs are positioned is referred to as the anther (see also[44]). The part proximal to the anther corresponds to the filament and the area distal to the anther is defined as the connective extension.

301. Number of fertile stamens (C1). Number of fertile (functional) stamens in bisexual or male flowers. Staminodes (co-occurring with fertile stamens) are not counted and female flowers are ignored for this character. Stamen number is highly variable within angiosperms and ranges from one (e.g., Chloranthaceae[45]) to several thousands (e.g., Cactaceae[46]). We record the number of fertile stamens in whorled or spiral flowers as a continuous character (with integer values of 1 and above). In cases of fusion among stamen whorls, the number of stamens may be difficult to determine. In such cases, additional information based on merism, anatomy, development, or comparison with closely related taxa may be taken into account. In synandria of Myristicaceae, for example, the number of fertile stamens can be deduced from the number of thecae present[47]. In cases where stamen or anther morphology is not fully understood, we recorded the number of fertile stamens only when unequivocally clarified in the literature (e.g., Malvaceae[48]). Equivocal cases were left as missing data.

301_B. Number of fertile stamens (3-state) (D2c). Here we distinguish among androecia with 1-5 stamens, 6-10 stamens, and more than 10 stamens. These character states allow us to analyse stamen number in relation to various perianth characters irrespective of the number of stamens in one whorl. Thus, in clades with a whorled perianth and androecium, they inform us about transitions between haplostemonous and diplostemonous flowers regardless of whether the transition is from a trimerous or pentamerous stamen whorl to two such whorls or vice versa. Polystemony is the presence of an increased number of stamens, which can be achieved by the insertion of stamens pairs (e.g., Fouquieriaceae[8]), the formation of stamen fascicles (e.g., Clusiaceae[49]), the development of multiple stamens on a ring-primordium (e.g., Lecythidaceae[50]; Actinidiaceae[38]) or the multiplication of stamens whorls (e.g., Ranunculaceae[51,52]).

301_C. Number of fertile stamens (binary) (D2c). Here we distinguish between flowers with a lower (1-6) and higher (7 or more) number of stamens with the aim of highlighting the occurrence of flowers with more than one androecial whorl or polystemonous flowers (particularly in eudicots).

330. Androecium structural phyllotaxis (D1). Androecium phyllotaxis may be spiral, whorled or irregular[31]. We call this character structural because both fertile stamens and staminodes were considered here. Furthermore, in cases of stamen fascicles, it

is the phyllotaxis of fascicles, not individual stamens that we record here. Stamen arrangement is most often either spiral or whorled and in the latter case stamens may be arranged in one or more whorls. Irregular stamen arrangements occur less commonly and are often associated with polystemony (e.g., *Medusagyne*[53]). Androecium phyllotaxis is not always easy to determine, especially when there are many stamens (for guidelines, see notes under character 230. Perianth phyllotaxis). Particular caution should be exerted when scoring this character based on taxonomic literature, where irregular phyllotaxis is commonly mistaken for spiral phyllotaxis (e.g., Annonaceae[54,55]). This character is not applicable when there is a single structural stamen (i.e., one stamen, no staminodes; e.g., *Chloranthus,* Chloranthaceae).

330_A. Androecium structural phyllotaxis (binary) (D2d). Androecium phyllotaxis treated as a binary character (spiral vs. whorled; irregular phyllotaxis treated as missing data).

331. Number of androecium structural whorls (C1). The number of whorls was recorded as a continuous character (with integer values of 1 and above). We call this character structural because both fertile stamens and staminodes were considered. Furthermore, in cases of stamen fascicles, it is the whorls of fascicles that we record here. This character is not applicable for spiral or irregular stamen arrangements, or when there is a single structural stamen (i.e., one stamen, no staminodes; e.g., *Chloranthus,* Chloranthaceae). In the case of stamen fusion, the organization of the androecium may be difficult to evaluate and in such cases additional information from developmental or anatomical studies was considered if available. Equivocal cases were left as missing data.

331_A. Number of androecium structural whorls (3-state) (D2c). Here we discretized the number of androecium whorls into three character states: androecia consisting of a single whorl, androecia with two whorls, or androecia with more than two whorls (spiral or irregular androecia not scored).

332. Androecium structural merism (C1). Androecium merism is defined as the number of stamens or stamen bundles (fascicles) in one whorl and was recorded as a continuous character (with integer values of 1 and above). We call this character structural because both fertile stamens and staminodes were considered. This character is not applicable for spiral or irregular stamen arrangements, nor when there is a single structural stamen (i.e., one stamen, no staminodes; e.g., *Chloranthus*).

332_A. Androecium structural merism (4-state) (D2c). Discretization of androecium merism as a four-state character, distinguishing dimerous, trimerous, tetramerous, and pentamerous androecia. We ignore (treat as missing data) other merisms (e.g., hexamery, octomery) because these states are comparatively rare and caused reconstruction artefacts due to low statistical power in early analyses of this data set.

332_B. Androecium structural merism (3-state) (D2c). Discretization of androecium merism as a three-state character: trimerous, tetramerous, or pentamerous. Here we treat dimery as missing data.

305. Filament (D1). Here we here record absence or presence of the filament, and in the latter case, the shape of the filament. Shape is considered in terms of length and width and is defined in relation to anther length/width. The width of the filament may thus either be broad as in laminar (e.g., Eupomatiaceae) or bulky stamens (e.g.,

Chloranthaceae), or narrow (filamentous) as found in many core eudicots groups (e.g., Rosaceae). This character was considered inapplicable when filaments were entirely fused with each other or to the perianth.

305_A. Filament (binary) (D2d). The length of the filament is not taken into account here and two forms of filament differentiation are compared: laminar (wide filament) vs. typical (narrow filament).

311. Anther orientation (D1). Anthers of angiosperms are rather uniform in their basic structure. They normally have four microsporangia (pollen sacs) that are arranged pair-wise in two thecae. The two microsporangia of a theca usually release their pollen grains through a common opening (stomium). At anthesis, the stomium of these thecae may face the floral centre (i.e., anther orientation is introrse) or the floral periphery (i.e., anther orientation is extrorse). A third possibility is latrorse anther orientation, where pollen is released toward the side (i.e., toward neighbouring anthers). Lastly, thecae may be positioned in a transverse position at the tip of the connective and thus dehisce upward in the flower (e.g., *Sinofranchetia*[56]).

312. Anther attachment (D1). Anther attachment refers to the insertion of the filament on the anther connective (i.e. the tissue connecting the two thecae of an anther). Anthers may be basifixed, with the filament attached to the base of the connective; dorsifixed, with the filament attached to the dorsal side of the anther, or ventrifixed, with the filament attached to the ventral side of the anther. With our definition of the filament as encompassing the region below the pollen sacs (see Character 305), we score laminar stamens as basifixed. Anther versatility is not scored here as it is not directly linked to any of the three types of anther attachment, for example the basifixed stamens of *Tulipa* or the dorsifixed stamens of *Lilium* may be versatile.

312_A. Anther attachment (binary) (D2d). Here we treat anther attachment as a binary character, treating the rare ventrifixed state as missing data.

313. Anther dehiscence (D1). Anther dehiscence refers to the type of opening of the anther when releasing its pollen through the stomia. The most common mode of dehiscence is by longitudinal slits that extend along the entire length of each theca. The stomium may bifurcate at its distal and/or proximal end and thus a valve is formed (e.g., *Eupomatia*[12,56]). Such dehiscence is commonly referred to as H-valvate. Specialized valves in the form of flaps for each pollen sac occur in some Laurales and basal eudicots[56]. We refer to them here as flap-valvate. Dehiscence of longitudinal slits may be incomplete and only occur over a short extent in the distal, proximal or central part of these slits and may thus be pore-like (e.g., *Bixa*[57]). In several taxa, however, specialized pores are observed (e.g., *Erica*[58]). In addition, there are several rarer modes of dehiscence such as circular dehiscence of one anther (e.g., *Hennecartia*) or several anthers (*Stephania*[59]). Longitudinal slits may also be confluent distally (e.g., *Cocculus*[56]; Triuridaceae[60]).

313_A. Anther dehiscence (3-state) (D2d). Here we categorize anther dehiscence into three states, focussing on variation in angiosperms outside monocots and core eudicots: longitudinal, H-valvate, and flap-valvate. Pores and short apical slits are morphologically derived from longitudinal slits and are treated here as such.

Gynoecium

401. Number of structural carpels (C1). Number of fertile or sterile carpels in bisexual or female flowers, recorded as a continuous character (with integer values of 1 and

above). Contrary to the number of stamens (character 301), the number of co-occurring carpellodes (sterile carpels) is counted here because this number is often more easily obtained from the literature than the actual number of fertile carpels. However, consistently with our treatment of sexual dimorphism, the number of carpellodes in male flowers is ignored for this character. In multicarpellate, unilocular gynoecia with complete carpel fusion up to the stigma (e.g., *Primula*), it may be difficult to assess the number of carpels unequivocally. In such cases, we have scored the number of carpels only if it is well established based on anatomical or developmental investigations. Similarly, in gynoecia where one or more carpels are reduced (e. g., in the pseudomonomerous gynoecia of some Arecaceae[61]), the total number of structural carpels was only scored when unequivocally determined in the literature. Contrary to the perianth and the androecium, we do not have a separate character for gynoecium merism here. This is because gynoecia with two or more whorls are rare in angiosperms (see above) and gynoecium merism therefore usually equals the number of carpels per flower.

401_B. Number of structural carpels (5-state) (D2c). About 10% of angiosperms are unicarpellate, but in the majority of angiosperms carpel number is either three (most monocots) or between two and five (most core eudicots). Compared to two and five, presence of four carpels is relatively rare and here we treat it in the same state as five carpels. In addition, we lump all multicarpellate gynoecia (i.e., with more than five carpels[62]) in a single category.

400. Gynoecium phyllotaxis (D1). Gynoecium phyllotaxis may be spiral or whorled[62]. In rare cases, carpels may also be arranged in an irregular fashion, but such cases are not represented in our data set. Gynoecia with two or more whorls of carpels are rare in angiosperms[39]. Therefore, contrary to what was done in scoring the perianth and the androecium, we do not have a separate character for number of gynoecium whorls. Instead, we distinguish between single whorls and multiple whorls in this character. This character is not applicable to unicarpellate flowers.

400_A. Gynoecium phyllotaxis (D2d). Here we distinguish only between spiral and whorled carpel arrangement, irrespective of the number of whorls.

403. Fusion of ovaries (C1). Degree of ovary fusion expressed as a fraction of the total vertical (longitudinal) length of the ovary. Fusion of styles and stigmas is not taken into account here. Not applicable when there is a single carpel.

403_A. Fusion of ovaries (binary) (D2c). Here we basically distinguish between apocarpous (free) and syncarpous (fused) gynoecia. Ovaries with less than 5% of their total length fused are treated here as apocarpous. The reason for this is that floral organs of the same whorl or even of two successive whorls often appear to have a short common base and accordingly are often described as “basally connate” or “basally adhering” without being clearly fused.

411. Number of ovules per functional carpel (C1). Number of ovules per carpel recorded as a continuous character (with integer values of 1 and above). Reduced (sterile) carpels are not taken into account here.

411_A. Number of ovules per functional carpel (3-state) (D2c). Here we discretize the number of ovules per carpel as a three-state character, distinguishing among one, two, and three or more ovules per carpel.

Reconstruction of the 3D model

To obtain the 3D model of the reconstructed ancestral flower shown in Fig. 1 and Supplementary Movie 1, we first summarized our ancestral state reconstruction results in the form of a floral diagram and a floral formula (see section Reconstructing the ancestral flower and Supplementary Fig. 2 below). A.H. then proceeded to make an actual sculpture of the hypothesized ancestral flower using professional oil clay (Plastiline®, hardness 60, J. Herbin manufacturer) modelled on a skeleton of metallic wires. Each element was first modelled independently as a wire structure and then attached to the main axis and covered with plastiline. The sculpture weighing ca. 2 kg is about 30 cm in diameter and 20 cm in height. To ensure consistency with the results obtained in this paper in terms of organ phyllotaxis, the final sculpture was adjusted by the lead author (H.S.). R.C. then reconstructed a 3D model of the sculpture using photogrammetry. An initial set of ca. 450 colour photographs of the object were taken from many different angles using a Pentax K10D camera (10.2 megapixels, focal length: 18–55 mm). To increase resolution of details in the centre of the flower, a special focus set of ca. 200 photographs of the androecium and gynoecium completed the original whole-sculpture set. The 3D reconstruction was performed using Agisoft PhotoScan (© 2014 Agisoft LLC, 27 Gzhatskaya St., St. Petersburg, Russia). The 3D mesh was then imported in Geomagic Studio 2012 (Geomagic Worldwide Headquarters 430 Davisdrive, Marisville, NC) to correct artefacts. S.P. further edited the 3D object in Amira 5.4.1 (FEITM) to refine the base of the gynoecium and androecium, and in Autodesk®Meshmixer 11.0.544 (Autodesk Ink.) to smooth the surfaces. Last, the final model was segmented and coloured in Amira, then photographed and recorded in movement to produce the image and film presented here. Although this method allowed us to obtain a satisfying rendering of the reconstructed ancestral flower, we note that arbitrary choices had to be made for both artistic and practical reasons. In particular, the shape and colour of organs were not reconstructed by our analyses and thus many other alternative representations of the same set of results may exist.

Supplementary Discussion

Phylogenetic and molecular dating results

For the ancestral state reconstruction analyses presented in this paper, 10 distinct sets of phylogenetic trees were used, which we refer to as series. The A series corresponds to the original trees produced by Magallón et al.[63], while the B, C, D, and E series correspond to re-analyses of the same dataset in BEAST without fixing the tree topology (Supplementary Fig. 1; see Methods). The A200, B200, C200, D200, and E200 series correspond to the same analyses without a maximum age constraint on the crown node of angiosperms. All maximum clade credibility (MCC) trees are provided as Supplementary Data 3-12.

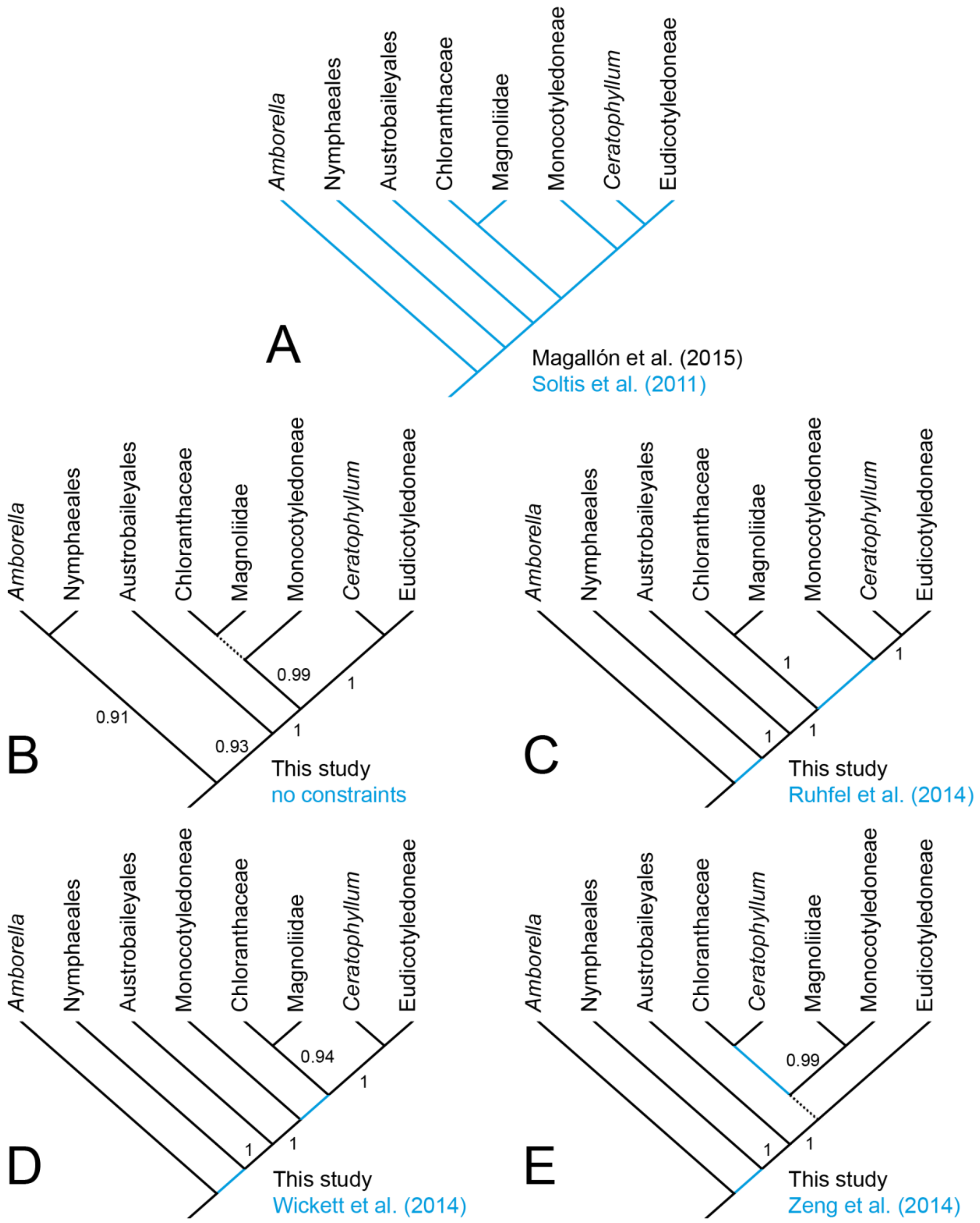

Supplementary Figure 1. Summary of topology, support, and constraints across phylogenetic (BEAST) analyses. A, Original analysis of Magallón et al.[63]: tree fixed, estimated from a RAxML analysis with relationships constrained following Soltis et al.[64]. B, Unconstrained analysis. C, Two topological constraints according to complete plastid genome analyses[65–68]. D, Two topological constraints according to the phylotranscriptomic analyses of Wickett et al.[69]. E, Two topological constraints according to the phylotranscriptomic analyses of Zeng et al.[70]. The letters correspond to the same codes used throughout this study. Blue branches denote topological constraints enforced in the analyses. Posterior probability branch support is given for unconstrained (estimated) relationships. Dotted branches received support less than 0.90. For full details (complete MCC trees), see Supplementary Data 3-12.

While the general structure of the B, C, D, and E trees was very similar to that of the A tree (except where specific constraints were enforced), we found a number of small differences, typically affecting relationships that have historically been difficult to resolve. These include basal relationships in Ranunculales, the relative positions of Buxales and Trochodendrales, the positions of Dasypogonaceae and Dilleniaceae, relationships within Santalales, Cornales, Ericales, Lamiales, and Malpighiales, and higher-level relationships within Lamiidae, Campanulidae, and Fabidae. The most striking difference concerns the relationships among orders of Rosidae. Whereas the A series shows a large clade that includes Geraniales, Myrtales, Crossosomatales, Picramniaceae, and Malvidae, in the B series [Picramniaceae + Malvidae] are instead sister to [Crossosomatales + Fabideae], and Geraniales and Myrtales successively diverge at the base of Rosidae. The C, D, and E series display different alternative sets of relationships in Rosidae. However, none of these conflicts were statistically supported. The posterior samples of trees from the B, C, D, and E series are therefore expected to effectively capture this uncertainty by sampling many plausible alternatives, unlike the A series in which topology was entirely fixed in BEAST.

The PhyloCode definitions[64,71] used to locate key nodes in our trees can accommodate phylogenetic uncertainty. However, for this study, 'floating nodes' were not desired for the key clades under focus. Importantly, all fifteen key clades considered in this study were very well supported across all analyses (Supplementary Table 1). This implies that in nearly all of the trees sampled here, key nodes (defined with the getMRCA and AddMRCA from the ape package of R and from BayesTraits, respectively) designated the exact same clades, with exactly the same number of tips (with one exception: see footnote on Superasteridae below).

Supplementary Table 1. Summary of branch support (BEAST posterior probabilities) for the fifteen key clades across the five main series.

Clade	Nr of tips	A	B	C	D	E
Angiospermae	792	const.*	1	1	1	1
Mesangiospermae	781	const.*	1	1	1	1
Magnoliidae	42	const.*	1	1	1	1
Monocotyledoneae	96	const.*	1	1	1	1
Eudicotyledoneae	638	const.*	1	1	1	1
Commelinidae	40	const.*	1	1	1	1
Pentapetalae	602	const.*	1	1	1	1
Superasteridae	289	const.*	0.84	0.82	0.89	?[†] / 0.93[§]
Asteridae	230	const.*	1	1	1	1
Lamiidae	59	const.*	1	1	1	1
Campanulidae	118	const.*	1	0.98	0.99	0.86
Superrosidae	313	const.*	1	1	1	1[†] / 0.04[§]
Rosidae	285	const.*	1	1	1	1
Malvidae	38	const.*	1	1	1	1
Fabidae	216	const.*	1	1	1	1

*Node support not evaluated (node constrained).

†Exactly same group as in the remaining analyses.

§Modified clade content, following a strict application of the PhyloCode definition: in the E series, Dilleniales were more closely related to Rosidae than Asteridae, and should therefore be included in Superrosidae rather than Superasteridae in these trees[64]. However, for practical reasons, we had to define the Superasteridae node as the most recent common ancestor of Asteridae, Caryophyllales, Berberidopsidales, Santalales, and Dilleniales in all of our analyses. Therefore, in the ancestral states reported for the E series in this paper, Superasteridae are the same clade as Pentapetalae.

The A200, B200, C200, D200, and E200 series produced very similar tree topologies as their respective constrained series, although in some instances removing the strong constraint on the age of the angiosperms did affect weakly supported relationships higher up in the tree. For instance, while the MCC tree from the B series showed Chloranthaceae sister to Magnoliidae (with posterior probability, hereafter p.p. = 0.21), the MCC tree from the B200 series instead showed Monocotyledoneae sister to Magnoliidae (p.p. = 0.80).

The A, B, C, D, and E analyses produced very similar age estimates for the 15 key nodes considered in this study (Supplementary Table 2). This was expected because all analyses were conducted using the same molecular dataset and comprehensive set of fossil age constraints as in the reference analysis[63]. The A200, B200, C200, D200, and E200 analyses also produced very similar age estimates, all of which were significantly older than the ages obtained in the A-E analyses, due to the removal of the maximum age constraint of 139.5 Ma on the crown-group age of angiosperms. For instance, the crown-group age of angiosperms in the A200 and B200 analyses were 160.0-255.8 Ma and 205.4-262.5, respectively, consistent with recent molecular dating analyses of angiosperms that do not assume a maximum age constraint on the crown-group[63,72–75]. Although the true crown-group age of angiosperms is a difficult and controversial question that may never be possible to resolve using current data, the A-E and A200-E200 sets of trees used in this study provided two ends of the spectrum of possible time scales, allowing us to test effectively the sensitivity of our results to this question.

Supplementary Table 2. Summary of divergence time estimates (median ages and 95% credibility intervals, in million years) for 15 key nodes across analyses.

Clade	A	B	C	D	E
Angiospermae	139.4 (139.0-139.5)	139.4 (138.3-158.1)	139.4 (139.0-139.5)	139.4 (138.8-139.5)	139.4 (139.0-139.5
Mesangiospermae	135.9 (135.0-136.9)	136.6 (137.6-138.9)	136.0 (135.0-136.7)	136.1 (135.2-137.0)	135.9 (134.9-136.7)
Magnoliidae	132.4 (130.2-134.1)	133.5 (131.3-135.3)	132.5 (130.7-134.4)	132.7 (130.7-134.1)	133.1 (131.3-134.7)
Monocotyledoneae	133.2 (131.6-134.7)	132.9 (131.0-134.4)	132.9 (131.6-134.3)	133.4 (132.0-134.5)	132.1 (130.8-133.6)
Eudicotyledoneae	131.7 (129.7-133.4)	132.5 (130.5-134.2)	131.5 (130.1-133.3)	131.9 (130.4-133.6)	132.5 (131.1-133.6)
Commelinidae	108.2 (101.0-117.3)	114.5 (110.0-119.0)	110.3 (104.9-116.9)	114.6 (109.7-120.7)	112.2 (108.3-115.7)
Pentapetalae	123.7 (120.9-126.5)	124.8 (122.2-127.3)	124.3 (122.2-126.7)	123.9 (120.5-126.8)	125.3 (123.6-126.9)
Superasteridae	122.6 (119.6-125.6)	123.5 (120.7-126.3)	123.2 (120.8-126.0)	122.7 (119.0-126.3)	123.3 (121.2-125.1)
Asteridae	114.6 (110.4-	114.1 (110.1-	114.1 (109.9-	114.9 (110.0-	115.8 (112.6-

Clade	A	B	C	D	E
	118.9)	118.0)	118.1)	122.2)	119.2)
Lamiidae	92.7 (81.5-105.4)	92.1 (83.1-99.7)	93.4 (83.9-106.3)	90.7 (80.4-101.3)	94.3 (83.3-102.5)
Campanulidae	102.7 (93.9-112.6)	103.5 (95.1-110.3)	102.9 (93.9-110.9)	103.8 (96.2-115.7)	105.4 (98.5-110.7)
Superrosidae	122.4 (119.5-125.4)	123.2 (120.8-125.8)	122.8 (120.4-125.4)	122.5 (119.3-125.6)	124.8 (123.7-126.2)
Rosidae	121.3 (118.4-124.6)	122.1 (119.7-124.8)	121.7 (119.3-124.6)	121.5 (118.5-125.0)	122.4 (120.3-124.3)
Malvidae	104.3 (98.3-112.2)	113.2 (106.4-118.0)	107.2 (99.7-114.8)	106.8 (100.2-113.5)	107.1 (99.9-112.5)
Fabidae	116.8 (113.3-120.9)	116.2 (113.0-119.6)	115.9 (113.1-118.7)	115.4 (112.8-118.6)	116.2 (113.0-118.9)

Dealing with uncertainty in ancestral state reconstructions

In this study, we explored ancestral state reconstruction using three distinct approaches: maximum parsimony (MP), maximum likelihood (ML), and reversible-jump Markov Chain Monte Carlo (rjMCMC). Our rationale was that if the three approaches consistently reconstructed the same ancestral state for a given node, one may have greater confidence in this reconstruction. Although the novelty of this paper was to use model-based methods for floral evolution through deep time, we thought it was also important to use MP for two reasons. First, MP is not conditional on a stochastic model of evolution. Although MP also comes with its own assumptions and typically suits characters for which change is rare (i.e., occurs at slow rates), this approach has the attractive property that no assumption is made on the relationship between change and branch length; in other words, it is similar to a model where rates are allowed to vary widely and be different for each branch of the tree[76,77]. Conversely, ML and rjMCMC results are conditional on the assumption that rates of morphological change are constant through time and across lineages. Thus MP and ML / rjMCMC results presented here may be seen as two ends of the spectrum of rate variation. Obtaining identical results with all three approaches might thus indicate that reconstructions are robust to different assumptions on rate variation. Second, we also chose to report on MP results to allow comparison with previous similar work using MP focused on early-diverging angiosperms[78–83].

The main result that emerged from these comparisons (Supplementary Table 2) is that rjMCMC analyses were best at capturing uncertainty in ancestral state reconstructions, whereas ML analyses often presented a result with misleadingly high confidence. This is because the rjMCMC approach was specifically designed to integrate over uncertainty in parameter (transition rate) uncertainty, phylogenetic (topological) uncertainty, molecular dating (branch length) uncertainty, and model uncertainty[3,84]. Conversely, ML analyses typically reflect a result that corresponds to a point estimate of transition rates (the single set of values that optimize the likelihood), given a fixed tree with fixed branch lengths, and given a fixed model. While our exploration of multiple models in ML analyses allowed us to select the best-fit model (see Methods), we observed that for some characters different models could produce drastically different ML ancestral states, often with high confidence, while there was no significant support for one model over another. Therefore, we call for great caution with our ML results and especially the 'probabilities' (proportional

likelihoods) reported with each ancestral state. We also call for caution with our MP results. While the ML and rjMCMC results of this study allowed us to resolve some long-standing ambiguities on ancestral states left equivocal by MP in this and previous studies (e.g., sex of the ancestral flower), they also highlighted that some unequivocal MP results are in fact uncertain (e.g., laminar vs. filamentous stamens). For these reasons, we use the rjMCMC analyses as our main reference for the results of this study and their associated confidence.

To help visualize result consistency across the three methods, we computed a confidence score for each trait-node combination as follows:

*** reconstructed state identical across the three methods *and* lower bound of the 95% HPD† of the rjMCMC analysis ≥ 0.95

** reconstructed state identical across the three methods *and* lower bound of the 95% HPD of the rjMCMC analysis > 0.5

or reconstructed state identical across ML and rjMCMC *and* lower bound of the 95% HPD of the rjMCMC analysis ≥ 0.95

* otherwise

†Highest Posterior Density interval. In this paper, we refer to this interval as the Credibility Interval (CI).

We found that most trait-node combinations were reconstructed with the highest confidence (Fig. 3; Supplementary Data 1). For instance, in the A series, 215 out of 405 nodes received a score of *** (75 were scored ** and 115 were scored *). These numbers were remarkably conserved across the 10 series of trees (e.g., the number of nodes scoring *** varied from 185 to 216).

In general, relaxing the maximum constraint on the crown-group age of angiosperms had very little impact on the reconstructed ancestral states (Supplementary Data 1), but it did slightly lower overall confidence (A200-E200: 185-204 nodes scoring ***, vs. A-E: 199-216 nodes scoring ***). Decreasing confidence with increasing branch times is an expected property of the Markov models used in this study.

Furthermore, the general pattern of confidence distribution across nodes and traits was also remarkably conserved across the 10 tree series (Fig. 3). We found that general floral traits (sex, ovary position) and perianth traits could be reconstructed with higher confidence than androecium and gynoecium traits. The traits reconstructed with highest confidence were structural sex (100_B) and perianth presence (201_A), both with a mean score of 3 stars across focal nodes in the A series. The traits reconstructed with lowest confidence were androecium structural merism (332_A, 332_B) and number of stamens (301_B), with mean scores across focal nodes of 1.2, 1.3, and 1.5, respectively, in the A series.

We also found that focal nodes nested in monocots and eudicots were typically reconstructed with higher confidence (e.g., mean score across traits in A series: 2.3-2.5 stars) than deeper, more basal nodes. Of the latter, Eudicotyledoneae received the lowest confidence (A series: 1.7) and Monocotyledoneae the highest confidence (A series: 2.2). Angiospermae were intermediate on this scale, with a mean score of 2.1 in the A series (1.6-2.1 across series).

Because the A and C series reflect to the most widely accepted view of higher-level relationships among angiosperms (Supplementary Fig. 1) and the rjMCMC results of the C series take into account phylogenetic uncertainty (unlike the A series), we

decided to use the results from the C series as our main reference for the rest of this study. However, the 10 series of analyses (A-E, A200-E200) produced remarkably consistent results, with identical reconstructed ancestral states for most trait-node-method combinations. For instance, the A and C series differed by only 17 trait-node combinations out of 405 using MP, 17 using ML, and 16 using rjMCMC. Thus, nearly all of the results discussed below also hold true for the remaining series (for full details, see Supplementary Data 1, 14-23).

Reconstructing the ancestral flower

Estimating features of the flower of the most recent common ancestor of all living angiosperms (hereafter referred to as the *ancestral flower*) is a difficult problem, because there are neither suitable outgroups for comparison, nor fossil flowers known from the time period when this ancestor existed (see Discussion). In this study, we make these inferences using the distribution of traits in extant angiosperms and their phylogenetic tree. We take this inference further than any previous study by using the largest dataset ever assembled for this purpose and, for the first time, methods based on explicit models of stochastic evolution for morphological characters. While these analyses help us resolve long-standing ambiguities and re-evaluate early floral diversification with a new hypothesis, such reconstructions necessarily come with limitations and some uncertainty. Ignoring uncertainty would entail the risk of presenting our reconstruction as arbitrarily definitive, while this may never be possible. Therefore, in this section we emphasize both strong, robust results and ambiguities that remain due to high uncertainty as measured by the rjMCMC analyses. Our main purpose here is to explain how we selected the single set of character states used to depict the reconstructed ancestral flower (Fig. 1) while acknowledging uncertainty where it remains.

Sex and perianth presence

The ancestral flower was most likely bisexual according to our results. This long-standing question had remained unresolved in MP analyses of smaller datasets focusing on early-diverging angiosperms[78,79,81,85]. Our own MP analyses of functional sex (char. 100_A) likewise produced an equivocal result. This is because *Amborella*, the putative sister group to all remaining angiosperms, has functionally unisexual flowers[9,86] and most Nymphaeales have bisexual flowers. Contrary to MP optimization, ML and rjMCMC analyses offer the advantage of taking into account the very long time (at least 139 million years) for which the *Amborella* lineage has evolved on its own. These analyses strongly support an ancestral functionally bisexual flower in all tree series (Supplementary Data 1). For instance, in the C series, the associated credibility interval for the ‘probability’ (proportional likelihood) of ancestral bisexuality is 0.99-1.00 (indicating that in 95% of the posterior sample, this probability was 0.99 or higher). In the rest of this paper, we refer to this interval as rjC-CI. In addition, female flowers of *Amborella* are in fact structurally bisexual, with one or two staminodes (sterile stamens[9,86]), making it more plausible that such flowers evolved from a bisexual ancestor[21], an hypothesis further supported by the recent report of partially bisexual male plants of *Amborella*[85]. All of our analyses (incl. MP) of structural sex (character 100_B) indeed strongly supported an ancestral structurally bisexual flower (rjC-CI = 1.00-1.00).

While we acknowledge that all our results will require further testing with more densely sampled floral trait datasets and matching dated phylogenies, this result in particular is unlikely to be challenged by increased sampling. Given the position of *Trithuria* (Hydatellaceae) as sister group of all remaining Nymphaeales[87] and the diversity of sexual systems among the 12 species of the genus[88], it would be tempting to think that denser sampling of *Trithuria* in particular might have an influence on the results obtained here. Because of the controversial interpretation of cosexual plants with bisexual reproductive units formed of stamens at the centre surrounded by carpels[28], we chose to not score (i.e., leave as missing data) any character that depended on interpretation of these units as flowers or inflorescences (see above under Special cases). Iles et al.[89] and Anger et al.[85] recently investigated the ancestral sexual system of *Trithuria* and angiosperms, respectively, using small datasets and phylogenies that included all species of the genus. While the two studies differed in character scoring (two binary vs. one multistate character), both found the ancestral state of *Trithuria* to be ambiguous because of the phylogenetic distribution of the variation in the genus. Therefore, we do not expect that our results would change had we sampled all species of *Trithuria* and scored them according to either interpretation of the bisexual reproductive units observed in some of the species.

The ancestral flower also most certainly possessed a perianth (char. 201_A; rjC-CI = 1.00-1.00), a strong result supported by all methods across all tree series.

Perianth phyllotaxis

Our analyses also suggest that perianth phyllotaxis (char. 230_A) of the ancestral flower was whorled, although we acknowledge that support for this result remained low. Whether perianth phyllotaxis was ancestrally whorled or spiral is a fundamental question in floral evolution that had remained vexingly unanswered in the last 15 years, following the identification of the earliest-diverging lineages of angiosperms (ANA grade). This is because of high variation in this trait among early-diverging angiosperms. *Amborella* and Austrobaileyales have entirely spiral flowers (perianth, androecium, and gynoecium), whereas Nymphaeales have whorled flowers[9,86,90]. All MP analyses of perianth phyllotaxis had left this character equivocal for the ancestral flower[21,32,78–81], a result echoed by all of our own MP analyses (Supplementary Data 1).

All of our ML analyses supported ancestral spiral phyllotaxis for the perianth. However, we found this result to be extremely sensitive to model choice and the prior on root state frequencies. Ancestral spiral phyllotaxis was typically supported by the all rates different (ARD) model when root state frequencies were set to a flat prior, which was consistently selected as the best-fit model in most analyses of this character. However, implementations of the ARD model with root state frequencies set to equilibrium (ARDeq) and the equal rates model (ER) instead supported ancestral whorled phyllotaxis. These differences may be interpreted with an examination of estimated transition rates. For the ARD model, the spiral-to-whorled transition rate (q_{10} = 0.0110) was estimated to be 55 times higher than the whorled-to-spiral transition rate (q_{01} = 0.0002). Without a strong prior on the root, such high transition rate asymmetry invariably implied that it was much more likely to have spiral phyllotaxis as the ancestral state, since transitions out of this state occurred at a high rate. Conversely, with a prior derived from equilibrium frequencies[91], the two rates were estimated to be very similar (q_{01} = 0.0004, q_{10} = 0.0005) and whorled

phyllotaxis was strongly supported as ancestral. Importantly, the equilibrium prior in the latter inference was only slightly biased towards whorled phyllotaxis (equilibrium frequency of whorled phyllotaxis implied by the estimated transition rates: $\pi_0 = 0.56$). Of course, there is no objective reason to assume a certain prior on root state frequencies or transition rates. However, there are several clear cases of transitions from whorled to spiral phyllotaxis (e.g., *Berberidopsis*, *Camellia*, *Paeonia*), which are supported by all of our analyses regardless of the reconstruction method or the inferred ancestral state in angiosperms, whereas the opposite is not true (spiral to whorled phyllotaxis was only inferred when angiosperms are ancestrally spiral).

In contrast, all of our rjMCMC analyses suggested whorled phyllotaxis as ancestral for the perianth (Supplementary Data 1). Support for this result remained low in all tree series, indicating high uncertainty (e.g., rjC-CI = 0.00-1.00). The analyses typically oscillated between inferences with whorled and spiral phyllotaxis reconstructed as ancestral (each with high 'probability'). However, the ER model was invariably visited more often through the rjMCMC chains (with posterior probability varying from 0.57 to 0.76 across analyses). Since both these models imply whorled phyllotaxis as ancestral in ML inferences, it is not surprising that whorled phyllotaxis was reconstructed as the most probable state (with mean probability varying from 0.71 to 0.81) in all rjMCMC analyses.

To further explore the sensitivity of this result, we conducted analyses in which core monocots (Petrosaviidae sensu Cantino et al.[71]) and Pentapetalae were pruned out of the tree. The idea of these analyses was to allow a distinct rate and mode of evolution for perianth phyllotaxis in early-diverging angiosperms (ANA grade, Magnoliidae, early-diverging monocots, and early-diverging eudicots) following long-standing observations that perianth phyllotaxis in these lineages is particularly labile and not fixed[32,92]. By pruning out the remaining angiosperms, we mimic similar procedures as described by O'Meara et al.[93] and Zanne et al.[94] for allowing lineage-specific rates of morphological evolution in ancestral state reconstruction with pre-defined rate shift locations. Our ML analyses using this procedure strongly supported whorled phyllotaxis as ancestral for the perianth of angiosperms. Not only was the UNI01 model (irreversible evolution from whorled to spiral) selected as best-fit in these analyses, but the ARD, ARDeq, and ER models also strongly supported this result. The ER transition rate ($q = 0.0021$) was estimated to be 5 times higher than the ER transition rate ($q = 0.0004$) of the analysis with all angiosperms, consistent with the prediction from comparative morphological studies that this trait should be particularly labile among early-diverging angiosperms.

Finally, whether the ancestral flower had a whorled or spiral perianth, subsequent deep nodes that gave rise to the vast majority of angiosperm diversity were more likely whorled. Indeed, all of our MP and rjMCMC analyses supported ancestral whorled phyllotaxis for the perianth of Mesangiospermae, Magnoliidae, Monocotyledoneae, and Eudicotyledoneae, although best-fit ML models still supported spiral phyllotaxis in most analyses of these nodes (Supplementary Data 1).

For all these reasons, we have favoured a whorled perianth for the reconstructed ancestral flower pictured in this paper (Fig. 1, Supplementary Fig. 2) and we believe our results further challenge the traditional view that early angiosperm flowers were mostly spiral. However, we fully acknowledge that further work will be needed to confirm this result and that comparative analyses alone might not allow us to provide

a definitive answer. Fossil discoveries and evo-devo studies will be equally important to shed new light on this important question.

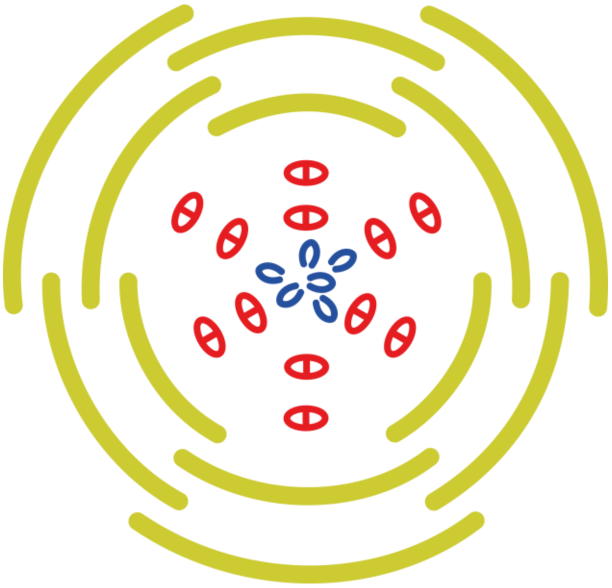

* P3+3+3+3 A3+3+3+3 G>5↺

Supplementary Figure 2. Floral diagram and formula of the reconstructed ancestral flower of Angiospermae, Mesangiospermae, and Magnoliidae, illustrating the most probable of several alternatives implied by our analyses. Note that the numbers of organs could not be inferred precisely (see below). Floral diagram colour code: light green = undifferentiated tepals; red = stamens; blue = carpels.

Number of perianth parts, whorls, and merism

We used two different discrete versions of the same character (201) to reconstruct the ancestral number of perianth parts (i.e., tepals or sepals and petals). When treated as a binary character with a threshold of six (char. 201_C), the ancestral number of perianth parts was invariably reconstructed as more than six with high confidence across all methods and tree series (e.g., rjC-CI = 0.93-1.00). When treated as a three-state character with thresholds of six and ten (char. 201_B), the ancestral number of parts remained equivocal (six to ten / more than ten) in MP analyses, while ML and rjMCMC analyses consistently reconstructed more than ten parts in all tree series, with high ML and rjMCMC support (rjC-CI = 0.75-1.00).

If the ancestral flower had a whorled perianth, then it most likely had more than two whorls (char. 231_A). MP analyses reconstructed either more than two whorls as ancestral (B, D, E, B200-E200) or were equivocal (two whorls or more than two whorls; A, C, A200), while ML and rjMCMC analyses always reconstructed more than two whorls with high support (e.g., rjC-CI = 1.00-1.00). This character was only scored in species with a whorled perianth and treated as missing data where inapplicable (spiral perianth). It is therefore particularly influenced by the sampling of taxa near the base of the angiosperms, and this influence is expected to be more pronounced in MP analyses than model-based methods. Furthermore, previous authors who expanded this character to be applicable to flowers with a spiral perianth (i.e., number of whorls or series) have unequivocally reconstructed this number as more than two in the ancestral flower using MP[78,79]. For these reasons, we consider it

more likely that the ancestral flower had a perianth formed of more than two whorls. Because comparatively few living angiosperms fall into this category (most of them belong to Nymphaeaceae, Magnoliidae, and Ranunculales) it was impractical to break it into more character states and thus we cannot reconstruct the ancestral number of perianth whorls more precisely.

Ancestral perianth merism (if whorled) was reconstructed as trimerous with high confidence across all methods and tree series, whether treated as a 4-state character (232_A; rjC-CI = 0.97-0.99) or as a 3-state character (232_B; rjC-CI = 0.99-1.00), consistent with previous work[78].

Given these results, we have decided to tentatively portray the reconstructed ancestral perianth with four whorls of three parts each (12 in total; Fig. 1, Supplementary Fig. 2), but we acknowledge that other alternatives with fewer or more parts cannot be ruled out based on our analyses.

The other three perianth traits considered in this study were all reconstructed with high confidence across all methods and tree series. Perianth differentiation (e.g., into sepals and petals; char. 234_A) was absent in the ancestral flower (rjC-CI = 1.00-1.00). Perianth parts were free (char. 204_A; rjC-CI = 1.00-1.00). Lastly, the perianth was actinomorphic (char. 207_A; rjC-CI = 0.99-1.00), consistent with a recent comprehensive study of this trait across angiosperms[95].

Androecium

Reconstructing ancestral states of the androecium posed the greatest challenges in this study, particularly so in the deepest nodes of the angiosperm tree.

We found ancestral phyllotaxis for the androecium (char. 330_A) to be equivocal with MP, as in previous work, and equally sensitive to model choice and assumptions as perianth phyllotaxis with ML and rjMCMC approaches. Some ML analyses supported whorled phyllotaxis as ancestral (A, C, and D series), as did all rjMCMC analyses, with low support (rjC-CI = 0.02-1.00). Given these results and for the same reasons outlined above for perianth phyllotaxis, we opted to portray the ancestral flower with whorled androecium phyllotaxis (Fig. 1, Supplementary Fig. 2), but we acknowledge that ancestral spiral phyllotaxis cannot be entirely ruled out at this stage.

All analyses reconstructed a high number of stamens: probably more than ten (char. 301_B; all analyses consistent, but confidence is low, e.g., rjC-CI = 0.11-1.00), definitely more than six (char. 301_C; rjC-CI = 1.00-1.00). If whorled, then the androecium had probably more than two whorls (char. 331_A; results highly uncertain across all analyses) and was probably trimerous (chars. 332_A and 332_B). Given these results, we tentatively chose to depict the ancestral androecium as formed of four whorls of three parts each (as the perianth), but we acknowledge that other alternatives are possible.

The ancestral stamens may have been laminar (char. 305_A). Laminar stamens were supported by MP and ML analyses of all tree series. rjMCMC analyses instead suggested more typical stamens with a narrow filament (state with the highest mean probability), but this remained highly uncertain in all analyses (e.g., rjC-CI = 0.01-0.98). Anther orientation was most likely introrse (char. 311_A; rjC-CI = 0.90-1.00) and anthers were basifixed (char. 312_A; rjC-CI = 0.99-1.00). Both these characters were reconstructed consistently across all methods and tree series. Last, anther

dehiscence (char. 313_A) may have been longitudinal, as supported by both MP and ML analyses but with low rjMCMC confidence (rjC-CI = 0.00-1.00).

Gynoecium

The ancestral flower most likely had a superior ovary (i.e., flower hypogynous; char. 102_B; result consistent and strongly supported across all analyses, e.g., rjC-CI = 1.00-1.00) with more than five carpels (char. 401_B; rjC-CI = 0.99-1.00). Despite considerable variation across the angiosperms and the use of five distinct states, it is remarkable that the reconstructed ancestral number of carpels was so consistent and well supported across all methods and tree series, suggesting strong signal in the data. Endress and Doyle[78] considered a binary character and reconstructed the ancestral number as more than one carpel, while Doyle and Endress[79] distinguished three states and reconstructed the ancestral number as two to five carpels. The ancestral carpels were probably free from one another (char. 403_A), but we note that this result remains uncertain (rjC-CI = 0.09-1.00). While MP analyses suggested that each carpel had a single ovule (char. 411_A), ML and rjMCMC consistently reconstructed an ancestral number of more than two ovules per carpel, with high confidence (rjC-CI = 0.99-1.00).

Last, ancestral gynoecium phyllotaxis (char. 400_A) was as difficult to reconstruct as ancestral perianth and androecium phyllotaxis, and remained equivocal in all MP analyses. However, here our ML and rjMCMC analyses consistently favoured spiral phyllotaxis, with high support in the A-E series (rjC-CI = 1.00-1.00). Although we cannot entirely rule out an ancestral whorled gynoecium, these results explain why we chose to depict the reconstructed ancestral flower with six spirally arranged carpels (Fig. 1, Supplementary Fig. 2).

Other features of the reconstructed ancestral angiosperm

Although our study focused on selected floral traits, it is possible to complement the portrait of the most recent common ancestor of all living angiosperms based on other recent studies using parsimony. First, the ancestral angiosperm was most likely an evergreen, woody plant with alternate, simple leaves with low-density pinnate-reticulate venation and chloranthoid leaf teeth[81,96–98]. Ecophysiological studies of both early-diverging angiosperms and the fossil record indicate that this ancestor may have lived in dark and unstable, wet (but not aquatic) habitats such as disturbed forest understory or shady streamside environments[99,100]. The ancestral flower probably had a pedicel and was borne in an inflorescence[78]. The stamens likely produced globose pollen grains with a single elongated aperture (i.e., monosulcate pollen) and columellar exine structure[81,101,102]. The carpels were ascidiate (i.e., flask-shaped with a distal opening that was closed by secretion) and the ovules most likely had two integuments[78]. Embryological studies suggest that the ancestral female gametophyte of angiosperms was four-celled and four-nucleate[103]. Recent work also led to a refined understanding of ancestral floral biology. The ancestral flower was most likely small and protogynous (i.e., with a female phase before the male phase), and probably pollinated by small insects such as flies (or perhaps thrips or moths) attracted to the flower by olfactory and visual cues[104,105]. Reconstructions of the fruit as fleshy and indehiscent[81] also suggest that seeds of the ancestral angiosperm were dispersed by animals, probably vertebrates[106]. It would be interesting in the future to further test these hypotheses by using a similar approach as we have applied here for key floral traits.

Reconstructing the ancestral flower of other key clades

Here we justify briefly how we reconstructed ancestral floral diagrams of other key angiosperm clades. As for the ancestral flower of all angiosperms, these reconstructions are neither fixed nor definitive and naturally entail some form of uncertainty. Traits reconstructed with high confidence such as sex, ovary position, and presence and symmetry of perianth are not discussed below. For all details, see Supplementary Data 1, 14-23.

Mesangiospermae

Our analyses did not support any key difference between the reconstructed ancestral flowers of angiosperms as a whole and Mesangiospermae (all differences were restricted to inferences with a low confidence score). MP results varied the most: some characters that remained equivocal for the ancestral flower of angiosperms became unequivocal in that of Mesangiospermae (e.g., bisexual flowers with a whorled perianth), or vice versa. ML results were identical and rjMCMC results very similar, with greater confidence for some key traits that remained uncertain in angiosperms (Mesangiospermae whorled phyllotaxis of androecium: rjC-CI = 0.92-1.00).

Magnoliidae

The ancestral flower of Magnoliidae reconstructed in this study is very similar to that of Angiospermae and Mesangiospermae (Supplementary Fig. 2), with similar levels of uncertainty for some traits, including perianth phyllotaxis, androecium phyllotaxis, and number of organs. The latter is not surprising, given the considerable variation of these traits among living members of the clade.

There were most likely more than two whorls (rjC-CI = 0.99-1.00) and these were trimerous (rjC-CI = 0.91-0.99). The total number of parts was certainly more than six (char. 201_C; rjC-CI = 0.96-1.00), and likely no more than ten (char. 201_B; rjC-CI = 0.77-1.00).

One interesting difference, not illustrated in our simplified floral diagrams, is anther orientation (char. 311_A). Whereas all other key nodes considered here, including angiosperms, were inferred to have ancestral introrse stamens (i.e., shedding pollen inwards), Magnoliidae were consistently inferred to have started with extrorse stamens by all three methods across all tree series (rjC-CI = 0.95-1.00), as inferred with MP in a previous study[78].

Monocotyledoneae (incl. Commelinidae)

In comparison with the other deepest nodes of angiosperm phylogeny considered here, reconstructing the ancestral flower of Monocotyledoneae (Supplementary Fig. 3) was relatively straightforward and most traits could be inferred with high confidence. This was expected, given the relative conservation of general floral structure across the entire clade. Our results are generally consistent with previous inferences based on MP analyses of smaller datasets[78] and reviews of the ancestral monocot flower[107–109].

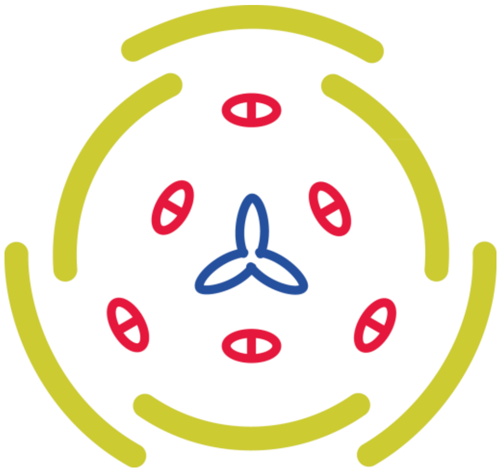

* P3+3 A3+3 G(3)

Supplementary Figure 3. Floral diagram and formula of the reconstructed ancestral flower of Monocotyledoneae and Commelinidae, illustrating the most probable alternative implied by our analyses. Floral diagram colour code: light green = undifferentiated tepals; red = stamens; blue = carpels.

Interestingly, ML analyses once again suggested potentially misleading ancestral states contradicted by both MP and rjMCMC analyses. For instance, ancestral perianth phyllotaxis was inferred to be spiral with low probability with ML (0.51 in C series) and whorled with high confidence with rjMCMC (rjC-CI = 0.95-1.00). A similar observation was made for the number of stamens (char. 301_C): more than six with ML (0.57) and one to six with rjMCMC (rjC-CI = 0.94-0.99). The ancestral number of three carpels was inferred with lower confidence (rjC-CI = 0.46-1.00) and so was ancestral syncarpy in monocots (rjC-CI = 0.53-1.00). For both these traits, ML analyses suggested again contradictory results. We interpret this sensitivity as a result of higher variation among early-diverging lineages of monocots and the very short branches marking the splitting of Mesangiospermae into Magnoliidae, Monocotyledoneae, and Eudicotyledoneae in all of our trees.

The ancestral flower of Commelinidae was reconstructed to be identical to that of Monocotyledoneae (Supplementary Fig. 3) for all traits considered in this study, albeit with greater confidence.

Eudicotyledoneae

Reconstructing the ancestral flower of Eudicotyledoneae was more difficult than for any other key clade considered in this study. This is due to a particularly high level of variation in flower structure among early-diverging lineages of eudicots (Ranunculales, Proteales, Trochodendrales, and Buxales).

Ancestral perianth phyllotaxis in eudicots was most likely whorled (Supplementary Fig. 4). Although best-fit model ML analyses of this trait continued to support ancestral spiral phyllotaxis for this node (as for angiosperms), both MP and rjMCMC analyses consistently indicated whorled phyllotaxis across all tree series, with low confidence (rjC-CI = 0.00-1.00). The ancestral number of perianth parts is uncertain: perhaps more than ten (char. 201_B; rjC-CI = 0.07-0.95), but most likely more than six (char. 201_C; rjC-CI = 0.71-1.00). These were probably arranged in more than

two whorls (rjC-CI = 0.75-0.99). Perianth merism was either dimerous or trimerous, as suggested by MP analyses (ancestral state equivocal). Dimery emerged as the most probable state in rjMCMC analyses but with low confidence (char. 232_A; rjC-CI = 0.19-0.99), while trimery was supported by all ML analyses. When dimery was treated as missing data (char. 232_B), rjMCMC analyses suggested pentamery as ancestral but with low confidence (rjC-CI = 0.44-0.72). The ancestral perianth of eudicots was probably undifferentiated, as consistently supported across nearly all MP and ML analyses and suggested by rjMCMC analyses with low confidence (rjC-CI = 0.08-1.00). Given these results, we tentatively depict the ancestral perianth of eudicots as formed of four whorls of two parts each (8 in total; Supplementary Fig. 4), but we note that other alternatives exist, in particular a trimerous version similar to the reconstructed perianth of angiosperms and Mesangiospermae (Supplementary Fig. 2). Similar uncertainty in reconstructing ancestral merism in eudicots had been found in previous MP studies[78,79].

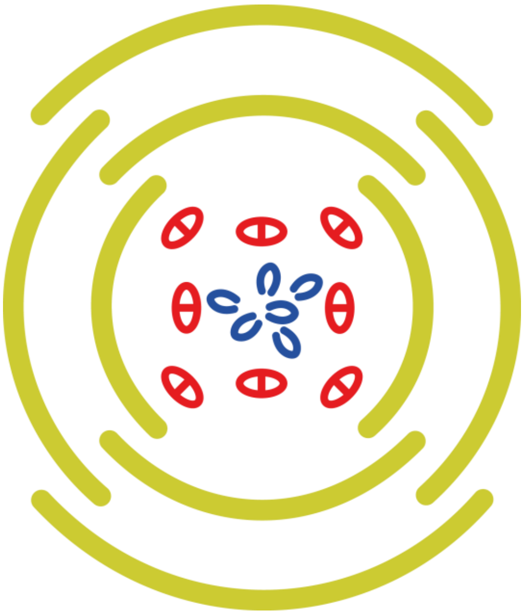

* P2+2+2+2 A4+4 G>5↺

Supplementary Figure 4. Floral diagram and formula of the reconstructed ancestral flower of Eudicotyledoneae, illustrating the most probable of several alternatives implied by our analyses. Note that the numbers of organs could not be inferred precisely. Floral diagram colour code: light green = undifferentiated tepals; red = stamens; blue = carpels.

The ancestral number of stamens in eudicots remains highly uncertain. It was probably more than six (char. 301_C; rjC-CI = 0.96-1.00) although MP analyses reconstructed one to six stamens. The 3-state version of this character favoured more than ten stamens but always with very poor confidence (rjC-CI = 0.01-1.00). Androecium phyllotaxis was most likely whorled (rjC-CI = 0.98-1.00), with perhaps two whorls (rjC-CI = 0.65-1.00). The ancestral merism of the eudicot androecium is uncertain. All rjMCMC analyses of the 4-state version of this character (332_A) suggested tetramery as ancestral (rjC-CI = 0.00-0.99), while the 3-state version (char. 332-B) favoured pentamery (rjC-CI = 0.00-1.00) and all MP analyses left this state equivocal (trimerous / tetramerous / pentamerous) for both versions of the

character. For these reasons, we tentatively reconstruct the ancestral androecium of eudicots as formed of two whorls of four stamens each (Supplementary Fig. 4), but we note that this result is uncertain.

The ancestral number of carpels in eudicots also remains uncertain. MP and ML analyses consistently supported more than five carpels, but rjMCMC analyses instead suggested four or five carpels, with low confidence (rjC-CI = 0.22-0.97). We chose to depict the ancestral gynoecium of eudicots with six carpels, as for angiosperms, Mesangiospermae, and Magnoliidae. As for angiosperms as a whole, ancestral gynoecium phyllotaxis of eudicots also remains uncertain. For this node, all MP analyses suggested a whorled rather than equivocal ancestral state, whereas ML and rjMCMC analyses suggested a spiral arrangement of carpels with low confidence (rjC-CI = 0.52-1.00). Here we present a floral diagram with a spiral gynoecium, but we acknowledge that a whorled gynoecium is also possible. Last, our analyses favoured free carpels (rjC-CI = 0.04-1.00), but ancestral syncarpy in eudicots cannot be ruled out (for a discussion of this character, see Evolution of syncarpy in angiosperms).

Pentapetalae

The ancestral flower of Pentapetalae (Supplementary Fig. 5) was reconstructed with high confidence and very little conflict for most traits across all of our analyses (Supplementary Data 1). A key feature of this ancestral flower is pentamery of the perianth (chars. 232_A, 232_B), inferred with the highest confidence across all analyses (e.g., char. 232_A, rjC-CI = 1.00-1.00). To our knowledge, this is the first confirmation of this feature implied by the name originally given to this clade by Cantino et al.[71]. Other key features of the ancestral perianth of Pentapetalae (number of parts, two whorls, differentiation into sepals and petals, lack of fusion) were all reconstructed with high confidence across all analyses.

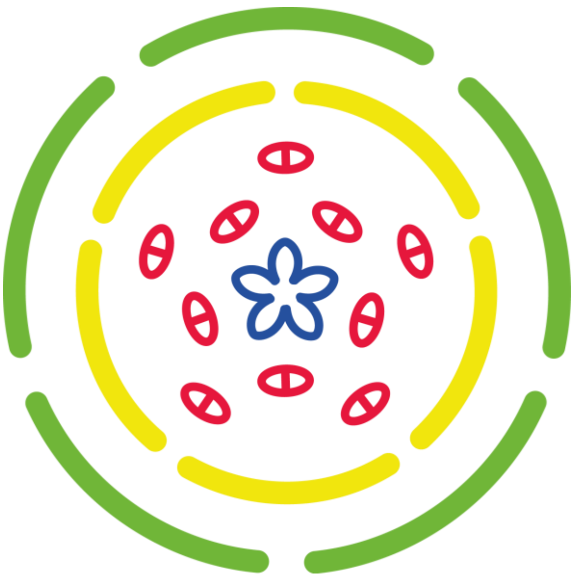

* K5 C5 A5+5 G(4-5)

Supplementary Figure 5. Floral diagram and formula of the reconstructed ancestral flower of Pentapetalae, Superasteridae, Asteridae, Superrosidae, Rosidae, Malvidae, and Fabidae, illustrating the most probable alternative implied by our analyses. Note that the numbers of stamens and carpels

could not be inferred precisely. Floral diagram colour code: green = sepals; yellow = petals; red = stamens; blue = carpels.

Conversely, reconstruction of the ancestral androecium of Pentapetalae was more problematic. The total number of stamens was definitely more than six (char. 301_B; rjC-CI = 1.00-1.00) and either six to ten (MP) or more than ten (char. 301_C; rjC-CI = 0.00-1.00). The androecium was whorled (rjC-CI = 1.00-1.00) and stamens were most likely arranged in two whorls (rjC-CI = 1.00-1.00) although MP analyses instead suggested a single whorl. The androecium was either tetramerous (as tentatively inferred for Eudicotyledoneae) or pentamerous, depending on the character considered, with high uncertainty either way (e.g., char. 332_B: rjC-CI = 0.00-1.00). We chose to depict the ancestral androecium of Pentapetalae as pentamerous because this is more likely given the ancestral pentamery of the perianth.

The ancestral gynoecium of Pentapetalae was whorled and composed of four or five fused carpels (traits reconstructed with high confidence across most analyses). We chose to depict it with five carpels for consistency with the perianth and androecium.

Superasteridae (incl. Asteridae, Lamiidae, Campanulidae)

The reconstructed ancestral flowers of Superasteridae and Asteridae are identical to that of Pentapetalae (Supplementary Fig. 5), with similar levels of confidence or uncertainty for each trait inferred. A possible difference could be fusion of perianth parts, suggested to be ancestral for these two clades in rjMCMC analyses, but with low confidence (rjC-CI Superasteridae = 0.07-0.86; Asteridae = 0.85-0.96) and contradicted by MP and ML analyses (perianth free).

The ancestral flowers of Lamiidae (Supplementary Fig. 6) and Campanulidae (Supplementary Fig. 7) are also similar to Pentapetalae, but differ significantly in having fewer stamens and fused perianth parts. Although the number of stamens cannot be inferred precisely from our analyses, several lines of evidence suggest the loss of a stamen whorl in these two clades. First, the number of stamen whorls is inferred to be one in both clades with high confidence across all analyses (rjC-CI Lamiidae = 0.98-1.00; Campanulidae = 0.98-1.00). Second, the total number of stamens is inferred to be one to five (char. 301_B) or one to six (char. 301_C) with high confidence for both clades. Perianth fusion was inferred to be ancestral with high confidence for both Lamiidae (rjC-CI = 0.91-0.99) and Campanulidae (rjC-CI = 0.95-1.00). Thus it is likely that both perianth fusion and the loss of a stamen whorl occurred along the lineage leading to Gentianidae, the larger clade of Asteridae that includes Lamiidae and Campanulidae (and Garryales) but not Ericales or Cornales[71]. It is also likely that these two transitions are functionally correlated, because floral specialization provided by floral tubes (resulting from perianth fusion[110]) may increase pollination efficiency, leading to a reduction in stamen number.

Lamiidae are also marked with a probable reduction to two carpels (rjC-CI = 0.97-1.00). It is possible that the ancestral flower of Campanulidae had an inferior ovary (rjC-CI = 0.80-0.94) but this is contradicted by both MP and ML analyses (ovary superior).

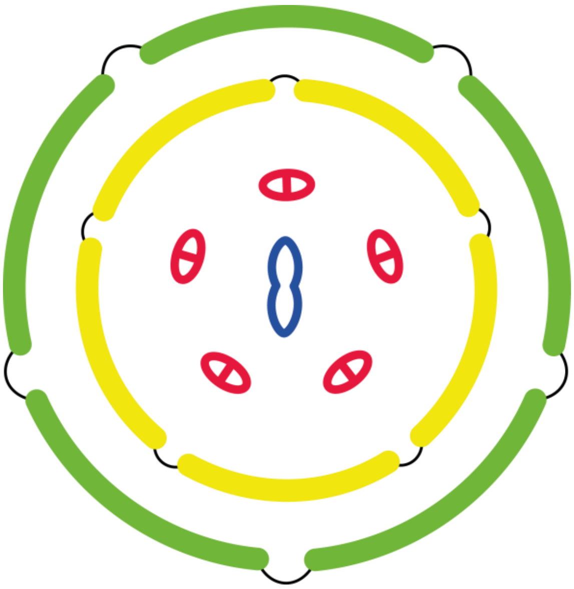

* K(5) C(5) A5 <u>G</u>(2)

Supplementary Figure 6. Floral diagram and formula of the reconstructed ancestral flower of Lamiidae, illustrating the most probable of several alternatives implied by our analyses. Floral diagram colour code: green = sepals; yellow = petals; red = stamens; blue = carpels.

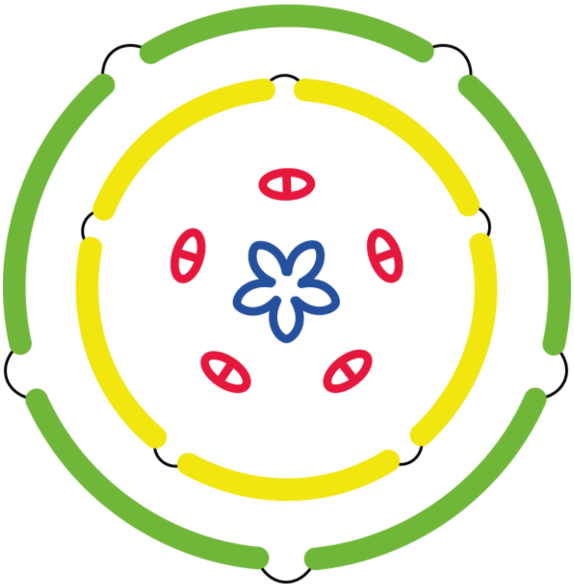

* K(5) C(5) A5 <u>G</u>(4-5)

Supplementary Figure 7. Floral diagram and formula of the reconstructed ancestral flower of Campanulidae, illustrating the most probable of several alternatives implied by our analyses. Floral diagram colour code: light green = green = sepals; yellow = petals; red = stamens; blue = carpels.

Superrosidae (incl. Rosidae, Malvidae, Fabidae)

The reconstructed ancestral flowers of Superrosidae, Rosidae, Malvidae, and Fabidae are identical to that of Pentapetalae (Supplementary Fig. 5), with similar levels of confidence or uncertainty for each trait inferred.

Results from the correlation analyses

We conducted pairwise correlation analyses with the main objective of evaluating the impact of potential correlations on reconstructed ancestral states obtained in our single-trait analyses (see Methods). These analyses also provided an unprecedented opportunity to quantify the level of correlation among floral traits at the scale of all angiosperms. As we report below, we found extensive evidence for correlated evolution among most floral traits. However, close examination of the reconstructed ancestral state combinations revealed little impact on the conclusions presented from the single-trait analyses.

Character pairs with absent states

Some of the character pairs we tested are intricately linked by applicability rules, typically causing two of the four state combinations to be biologically impossible (though not necessarily from a developmental or genetic point of view) and thus absent from our dataset. For instance, when analyzing the correlation between perianth presence (char. 201_A) and perianth symmetry (char. 207_A), the states "absent / actinomorphic" and "absent / zygomorphic" cannot be observed, because by definition our perianth symmetry character was only applicable to species with a perianth. Maintaining absent states in Markov models is problematic because transition rates in and out of such states cannot be estimated from the data. These rates are typically overestimated, often causing the absent state(s) to be inferred as ancestral for the root, especially with ML. In addition, characters linked by applicability rules are, by definition, correlated. In a few other cases, only a single combined state was absent, usually for similar reasons (applicability rules): for instance, ovaries are never observed to be free (char. 403_A) when they are inferior (char. 102_B). These are nontrivial issues of comparative methods that could be solved on a case-by-case basis (e.g., exclusion of pairs with two absent states, modification of the model for pairs with one absent state); however, they were not addressed here due to the large number of pairs tested. In total, out of 231 pairs tested, 10 have two absent states and 17 have one absent state. While the results for these pairs are included in our detailed summary (Supplementary Data 2), we have labelled them as problematic (with the number of absent states) and call for caution with interpretation of their results.

Support for floral integration

Both ML and rjMCMC analyses supported correlation between many of the character pairs tested, albeit in different proportions (Table 1). The ML analyses supported correlated evolution for 110 (48%) of the 231 pairs tested (i.e., with cumulative Akaike weight of correlated models, hereafter $w_D \geq 0.95$), while the rjMCMC analyses did so for 92 (40%) of the pairs tested (i.e., with Bayes Factor in support of correlated models, hereafter $BF_{DI} \geq 3$). While most of the pairs analyzed produced the same correlation result with the two methods (no support: 89 pairs; significant support: 60 pairs), 82 (35%) pairs did not (support with ML but not rjMCMC: 50 pairs; support

with rjMCMC but not ML: 32 pairs). These differences may be explained by three factors. First, the two approaches are grounded on different statistical frameworks and model selection philosophy[2]. Second, the ML approach only allowed us to explore a limited set of correlated and uncorrelated models, whereas the rjMCMC approach was specifically developed to perform a much more thorough exploration of model space[3]. Indeed, rjMCMC analyses always selected models with very few free transition rate parameters (typically in the range of 1 to 3), whereas our ML analyses explored more parameter-rich models (typically with 4 or 8 parameters, except for the ERnodual model) (Supplementary Data 2). This suggests that typical ML models for combined characters are overparameterized and that the models best fitting our data are much simpler but also asymmetric (i.e., not the ER model), a general result also echoed by our single-trait analyses (Supplementary Data 1). Last, rjMCMC analyses also integrate over phylogenetic uncertainty (topology and branch lengths) whereas ML analyses were performed on a single tree, which may have affected the outcome of correlation tests.

These results provide overwhelming evidence for integration of floral trait evolution. Although these findings are consistent with previous ideas and recent studies[111–116], this is to our knowledge the first large-scale test of floral integration in angiosperms taking into account the phylogeny and many floral traits. These findings are also compatible with the prevalence and functional importance of synorganization in many angiosperm flowers[34,117,118]. However, our results will remain to be tested using a framework for joint multivariate analysis of discrete traits when available, as recently developed for multivariate analysis of continuous traits[119–121].

Some of the correlations we found were expected from a functional point of view. For instance, perianth phyllotaxis (char. 230_A) is expected to correlate with fusion of perianth (char. 204_A) because whorled phyllotaxis has been argued as a developmental prerequisite for the evolution of perianth fusion[31], and indeed we find strong support for this correlation with both methods ($w_D = 1$; $BF_{DI} = \infty$). In other cases, correlations proposed by previous studies are not supported here. For instance, a correlation between zygomorphic flowers and a low number of stamens has been shown in Asteridae[122] and angiosperms[112], but our analyses do not support the correlation of perianth symmetry (char. 207_A) and number of stamens (char. 301_C; $w_D = 0.28$; $BF_{DI} = 0.01$). Differences between this and previous studies might be due to subtle variations in character definitions, taxon sampling, and methods. Last, we find signal for some surprising correlations, for instance between perianth differentiation (char. 234_A) and anther dehiscence (char. 313_B; $w_D = 0.76$; $BF_{DI} = \infty$). Although not expected, these are not absurd and may provide avenues for future research. Such correlations also certainly do not imply causation or a direct structural or functional link between the two traits; instead, these patterns might be caused by more direct correlations with other underlying traits[123] or pleiotropy of the genetic control of floral traits[124].

Impact on reconstructed ancestral states

Taking into account pairwise correlations in ancestral state reconstruction had little impact on the main conclusions presented in this study, especially when considering the uncertainty associated with reconstructed ancestral state combinations in the rjMCMC analyses (Supplementary Data 2). However, we did find some differences, especially within a given method (ML or rjMCMC), as well as contradictions between different pairs involving the same character. These issues have so far been very little

explored in the comparative literature, which tends to focus more on identification of correlations and selection of the model than on the implications for ancestral state reconstruction. While it is important to take these results into account, it is also essential to keep in mind that pairwise correlated models remain suboptimal approximations of multivariate integrated structures such as flowers. Here, we report primarily on reconstructed ancestral states for angiosperms as a whole.

In general, we found that the strong results that emerged from our single-trait analyses also received substantial support in the correlated analyses. For instance, our inference of an ancestrally bisexual flower is one of our most confident results (char. 100_A; rjC-CI = 0.99-1.00; see Supplementary Data 1 and the section above on Sex and perianth presence). In the correlation analyses, we tested the correlated evolution of this trait with 21 other floral traits. We found signal for correlation with 7 and 7 of these traits, using ML and rjMCMC, respectively. All ML analyses supported the bisexual state (in combination with the state of the other character) as ancestral for the angiosperms, and all rjMCMC analyses reconstructed the same ancestral state (in most cases with high confidence) except two. Likewise, all ML analyses but one and all rjMCMC analyses but two reconstructed perianth presence as ancestral, another result that received very strong support in our single-trait analyses (char. 201_A; rjC-CI = 1.00-1.00). A similar pattern again was found for the number of whorls (char. 231_B; more than two whorls reconstructed as ancestral in 17 and 20 pairs respectively).

Results that were more uncertain in our single-trait analyses remained so in our correlated analyses and were typically characterized by contradictions among different character pairs, between methods, and between single-trait and correlation analyses. Thus, ancestral perianth phyllotaxis (char. 230_A) was reconstructed as whorled in 10 and 9 out of 21 pairs with ML and rjMCMC, respectively, but most credibility intervals of the latter indicate the same level of uncertainty as our single-trait analyses. Furthermore, perianth, androecium, and gynoecium phyllotaxis are often of the same type in extant species, although exceptions exist (e.g., most flowers of *Magnolia* have a whorled perianth but a spiral androecium and gynoecium). Thus, we would expect to find these characters to be correlated, which is confirmed by all our correlation analyses of the three pairs (Table 1). Although our ML results from these three pairs are rather consistent with the main conclusions from this study (perianth whorled, androecium whorled or spiral, gynoecium spiral), the rjMCMC analyses suggest again that these results remain uncertain. A similar pattern was observed for the number of androecium whorls (char. 331_B; more than two whorls reconstructed as ancestral in 13 and 10 pairs with ML and rjMCMC respectively).

Eudicotyledoneae to Pentapetalae transition

As pointed out above, the reconstruction of the ancestral eudicot flower is uncertain for several characters. As a starting point for this discussion, we tentatively assume the ancestor of all Eudicotyledoneae to have had a perianth composed of four dimerous undifferentiated whorls and thus a total of eight identical perianth organs (see Supplementary Fig. 4). The ancestor of all Pentapetalae, on the other hand, was reconstructed with high confidence with a total of ten perianth organs arranged into two pentamerous whorls, differentiated into an outer whorl of five sepals and an inner whorl of five petals (see Supplementary Fig. 5). Thus, along the evolutionary path

from the eudicot ancestor to the Pentapetalae ancestor, there are primarily the following aspects that need to be considered: 1) the differentiation of the perianth into sepals and petals and 2) a change in merism of the whorls from two to five and, in close connection with this, a reduction in the number of whorls from four to two as well as an increase in the total number of perianth organs.

Perianth differentiation

In Pentapetalae, the differentiated perianth is most often characterized by the following series of features[83]: sepals and petals are each arranged in one whorl with the sepal whorl outside the petal whorl; sepals tend to be robust, green, broadly attached to the floral base, acute at their apex and are served by three vascular strands; petals tend to be delicate, coloured, narrowly attached, broad distally and served by a single vascular strand. Concomitant with their differing structural properties, sepals and petals also differ in their functions. While the main function of the sepals is to protect the inner organs during floral development, the main function of the petals it to attract and guide pollinators during anthesis. Among early-diverging eudicots, only few groups, especially in the Ranunculales (some Berberidaceae and Ranunculaceae) have a similar perianth with a distinct differentiation into two types of organs. In other lineages, the perianth is highly diverse and may be one-whorled and petaloid as in Proteaceae, weakly differentiated as in some Buxaceae, strongly reduced as in Platanaceae, or even lacking as in Eupteleaceae (see[83] and references therein).

The genetic basis of flower organ differentiation has received much attention since the first studies on the genetic control of flower development in *Arabidopsis* and *Antirrhinum*[125–129], and the early ABC model of three gene classes with its overlapping domains across the flower meristem has gained in complexity to accommodate new findings[130–132]. Our knowledge on floral identity genes has also expanded beyond the model plants belonging to the Pentapetalae, to encompass information from other angiosperm groups such as basal angiosperms, monocots, and early-diverging eudicots[17,132–139]. It has become clear that the petal identity program regulated by B-class MADS-box gene activity is highly variable outside Pentapetalae, both spatially and temporally[19,135,140–143]. This considerable variability in perianth identity programs across angiosperms may go hand in hand with the multiple transitions between an undifferentiated and a differentiated perianth[82,83,118,144,145]. However, the complexity of perianth differentiation is further enhanced by the fact that phyllotaxis, merism, and early primordium development are controlled independently of the floral organ identity program and therefore can evolve separately from each other[143,146]. Thus, there is still a long way to go before we fully understand the evolutionary transition from the undifferentiated perianth of the last common ancestor of all Eudicotyledoneae to the differentiated perianth of the Pentapetalae.

Merism

Dimery, inferred as ancestral for the Eudicotyledoneae in most of our analyses and in some earlier studies[144], is widespread throughout extant early-diverging eudicots and appears in many lineages (Ranunculales, Proteales, Trochodendrales, Buxales, Gunnerales)[36,147–155]. The number of whorls among these extant representatives, however, exceeds two only in Ranunculales, where for example Papaveraceae have

three dimerous perianth whorls[43], and possibly in some Buxaceae (e.g., *Pachysandra*[150]).

Trimery, supported as ancestral for the Eudicotyledoneae by our ML analyses and in an earlier study[82], is common among some families of Ranunculales (e.g., Lardizabalaceae, Menispermaceae) and also occurs in other early-diverging eudicots (e.g., Sabiaceae p.p.[156]; Platanaceae p.p.[157]).

Pentamery characterizes most Pentapetalae[36]. Outside Pentapetalae, pentamery is generally rare but is frequent in some lineages of early-diverging eudicots such as Sabiaceae[158,159] and Ranunculaceae[18,36,41]. In other early-diverging eudicots, the pentamerous condition is more unstable (e.g., Berberidaceae[31,160]; Papaveraceae[161]; Proteaceae[29]; Buxaceae[150]; and Platanaceae[157]).

Structurally, pentamery has been hypothesized to have evolved in different ways: from a dimerous flower by the addition of an organ in one whorl and the merging of two adjacent whorls; from a trimerous flower by the loss of an organ in one whorl or the fusion of two organs in one whorl and the merging of two adjacent whorls; or in a spiral flower by the regulation of spirally initiated organs into categories[80,162,163]. It appears that these pathways have been variously explored among early-diverging eudicots. However, the genetic mechanisms controlling floral merism are still little understood[163].

Closely related to meristic patterns, is the question about the number of perianth whorls. A hypothesis proposed in earlier studies[79] and that cannot be ruled out by our own analyses, suggests that there might have been a reduction to only two dimerous perianth whorls along the backbone leading to Pentapetalae. If we assume the formation of one pentamerous whorl, for example the calyx, from these two dimerous whorls as suggested above (addition of a third organ in one whorl and a concomitant merging of the whorls), the question about the origin of the second perianth whorl of the ancestral Pentapetalae flower arises. One option would be that each pentamerous whorl arose simply through an increase from two to five organs in each originally dimerous whorl and differentiation of the whorls into an outer calyx and an inner corolla. Another option would be that the corolla of Pentapetalae stems from the androecium as has been suggested for instance for some Ranunculales and Caryophyllales[143]. A third possibility is that it is the corolla of Pentapetalae that arose from the two original dimerous perianth whorls and that the calyx evolved via the incorporation of bracts into the flower. The evolutionary history that led to the differentiated, pentamerous, two-whorled perianth of Pentapetalae is still not clear and whether this happened through the incorporation of bracts, the sterilization of stamens or solely from the original, undifferentiated perianth of early diverging eudicots remains to be tested[79].

A closer look at different early-diverging eudicot lineages reveals that merism is highly variable in most groups. In Ranunculales, dimerous and trimerous flowers prevail among extant representatives but pentamerous flowers occur occasionally in a few families (Berberidaceae[31]; Papaveraceae[161]; and regularly in Ranunculaceae[18,36,41,164]). Based on our analyses, both dimery and trimery are possible as ancestral for the order (Supplementary Data 1). It has been suggested that the pentamerous flowers in Ranunculaceae were derived from trimerous flowers as many other representatives of the order are trimerous[162], however, earlier reconstructions left the merism of the ancestral Ranunculales flower equivocal between dimerous and trimerous[78,151,165]. Ranunculaceae not only have variable

merism numbers but also a highly dynamic phyllotaxis[31]. In pentamerous flowers, floral development commonly starts with two lateral prophylls and continues with the five sepals, all in a spiral sequence[36]. In these spiral flowers almost always five sepals and a higher Fibonacci number of petals are present (five spirally arranged petals in a few representatives; e.g., *Ranunculus acris*[41]), whereas whorled flowers either are dimerous or trimerous (e.g, *Eranthis*[166]). Pentamerous-whorled flowers, which are common in Pentapetalae, on the other hand, are rare in Ranunculaceae (e.g., *Aquilegia*[31]; *Leptopyrum*[41]).

In Proteales, some Sabiaceae (*Meliosma*) have been described as pentamerous and based on developmental data, pentamery is thought to have evolved from a trimerous ancestor[156,159]. However, an alternative hypothesis interprets these flowers as basically trimerous, with the loss of one organ in each whorl due to the establishment of floral monosymmetry[36]. Among extant Platanaceae, pentamery is unstable[157]. This is in contrast to fossil flowers of the family that either show stable pentamerism[167,168] or tetramerism[169]. Interestingly, the perianth of these fossil representatives is well-differentiated and thus indicates that the unstable merism and the reduced perianth of extant species most likely are linked with each other, and also that they represent a derived condition that evolved due to a switch from animal to wind pollination[170]. The structural evidence among extant representatives of Platanaceae indicates that pentamerism is either achieved by a reduction in one of the two trimerous whorls or by the addition of one organ in a tetramerous flower[157]. Among Proteaceae, pentamery is rare and only reported for some flowers of *Persoonia falcata*[152]; it has been explained as developing due to the increase in the floral apex and the concurrent switch from a dimerous whorled to a spiral phyllotaxis[171].

Finally, in Buxaceae (Buxales), structural evidence indicates that pentamery in male flowers of the fossil *Spanomera* evolved from a dimerous ancestor by duplication of one organ and the subsequent merging of two perianth whorls[172]. Derivation from a dimerous organization would also explain the superposed tepals and stamens. Female flowers of extant representatives, however, indicate the differentiation of a pentamerous perianth from spirally arranged phyllomes[157].

These highly variable meristic patterns and frequent changes in merism - often within species or even within individuals - indicate a lack of genetic canalization of floral organ number among early-diverging eudicots. A tighter genetic control and a canalization toward a stable - sometimes across entire families or orders - and predominantly pentamerous floral organization was only realized in the Pentapetalae[36] and even more so in the Asteridae, where it allowed for increased synorganization of floral organs[83,173].

Evolution of syncarpy in angiosperms

Syncarpy characterizes most major angiosperms lineages and is considered a key innovation of angiosperm evolution[34,117]. Possible advantages of syncarpy over apocarpy are manifold and include higher efficiency in pollination mechanisms, enhanced defence against seed predators, and increased diversity in seed/fruit dispersal strategies[117,174,175]. However, the arguably most significant advantage of syncarpy may be the presence of an intragynoecial pollen-tube transmitting tract (a compitum) shared by all carpels allowing for pollen tubes to cross between carpels and thus for centralized pollen tube selection[71,92,175,176]. Interestingly, simulation

studies support the hypothesis that syncarpy is generally advantageous over apocarpy in terms of both offspring number and quality[177]. Hence, syncarpous gynoecia are likely of to have high adaptive value, which is also reflected by the fact that reversals to apocarpy are relatively rare (Supplementary Data 14-23[34,117]).

Our analyses indicate that syncarpy has multiple origins. Among early-diverging angiosperms, it likely evolved independently in Nymphaeales and in several magnoliid lineages (e.g., Canellales, Piperales). Further, it is likely that the most recent common ancestors of Mesangiospermae and Eudicotyledoneae, respectively, were both still apocarpous. Within Eudicotyledoneae, syncarpy may have evolved at least three times independently, once in Papaveraceae, once in Sabiaceae, and a third time along the branch leading to Pentapetalae. Within Pentapetalae, syncarpy is ubiquitous and reversals to apocarpy are particularly rare and characterized be the retention of a compitum in the postgenitally united carpel tips (e.g., in some Apocynaceae, some Malvaceae[117,175]). For the most recent common ancestor of Monocotyledoneae, our results favour syncarpy. However, an apocarpous most recent monocot ancestor and multiple, independent origins of syncarpy within this clade cannot be excluded based on our reconstructions. It seems particularly interesting that the two major angiosperm clades, which together make up for almost 90% of all angiosperm species, i.e., Monocotyledoneae and Pentapetalae, are almost universally syncarpous, indicating that the evolution of stable syncarpy may indeed be a key innovation that has triggered the explosive radiations of these lineages.

The evolutionary scenario of multiple, independent origins of syncarpy outlined here is supported by the notion that syncarpy may occur by different developmental/morphological processes and patterns[78]: eusyncarpy, which is characterized by carpels that are fused at the centre of the gynoecium and usually forming a plurilocular ovary with axile placentas, occurs for instance in Aristolochiaceae, monocots and most eudicots. Paracarpy, with carpels fused into a unilocular ovary and parietal placentation is for instance present in Piperaceae, Saururaceae, Canellaceae, Winteraceae (*Takhtajania*), and Papaveraceae. In addition, comparative morphological studies found significant differences in modes of carpel fusion within monocots, suggesting multiple origins of syncarpy in this clade[108] (but see[79,178]). Thus, it is very likely that not all occurrences of syncarpy are homologous. In the present study, we have not distinguished between these and other potential types of syncarpy mainly because the structural circumscriptions and the phylogenetic distributions of these types are currently not well understood.

Further evolution of the gynoecium in the Mesangiospermae was marked by the reduction in the number of carpels (especially in the Superasteridae) and by the recurrent embedding of the ovary into the floral base (inferior ovary). The fossil record confirms that both syncarpy and inferior ovaries were already present relatively early in angiosperm evolution[179].

Furthermore, syncarpy seems tightly correlated with the evolution of whorled gynoecium phyllotaxis. This is not surprising based on architectural grounds that likely impede the fusion and synorganization of several carpels should they be located at differing distances from the floral centre as it is the case in a spiral gynoecium. Thus, whorled phyllotaxis is generally considered an important precondition for the synorganization of floral organs and by far the most common pattern of carpel arrangement in a syncarpous gynoecium[62]. Isolated cases of syncarpy combined with spiral carpel arrangement are present in otherwise

apocarpous lineages of the Magnoliidae, i.e., in *Eupomatia* (Eupomatiaceae) and *Gomortega* (Gomortegaceae). These cases differ significantly in their mode of carpel fusion from other syncarpous angiosperms and are presumably autapomorphic[81].

Finally, we note that according to our analysis the fusion and synorganization of perianth organs (syntepaly, synsepaly, sympetaly) occurred later in angiosperm evolution than syncarpy and that the fusion of stamens (synandry) is generally rare. The relative early evolution and the possible multiple origins of syncarpy may have been favoured by the central position of the carpels in the flower, which likely facilitated fusion and synorganization of carpels early in the evolution of flowers. This seems especially plausible in the light of the advantages of syncarpy over apocarpy pointed out above.

Supplementary References

1. Sauquet, H. PROTEUS: A database for recording morphological data and creating NEXUS matrices. Version 1.26. http://eflower.myspecies.info/proteus. (2016).
2. Posada, D. & Buckley, T. R. Model selection and model averaging in phylogenetics: advantages of Akaike Information Criterion and Bayesian approaches over likelihood ratio tests. *Syst. Biol.* **53,** 793–808 (2004).
3. Pagel, M. & Meade, A. Bayesian analysis of correlated evolution of discrete characters by reversible-jump Markov chain Monte Carlo. *Am. Nat.* **167,** 808–825 (2006).
4. Beaulieu, J. M., O'Meara, B. C. & Donoghue, M. J. Identifying hidden rate changes in the evolution of a binary morphological character: the evolution of plant habit in campanulid angiosperms. *Syst. Biol.* **62,** 725–737 (2013).
5. Pagel, M. & Meade, A. BayesTraits V2. (2013).
6. von Balthazar, M. & Endress, P. K. Reproductive structures and systematics of Buxaceae. *Bot. J. Linn. Soc.* **140,** 193–228 (2002).
7. Erbar, C. & Leins, P. Flowers in Magnoliidae and the origin of flowers in other subclasses of the angiosperms. I. The relationships between flowers of Magnoliidae and Alismatidae. *Plant Syst. Evol.* **Suppl. 8,** 193–208 (1994).
8. Schönenberger, J. & Grenhagen, A. Early floral development and androecium organization in Fouquieriaceae (Ericales). *Plant Syst. Evol.* **254,** 233–249 (2005).
9. Endress, P. K. & Igersheim, A. The reproductive structures of the basal angiosperm *Amborella trichopoda* (Amborellaceae). *Int. J. Plant Sci.* **161,** S237–S248 (2000).
10. Simpson, M. G. *Plant Systematics, 2nd edition*. (Academic Press, 2010).
11. Endress, P. K. Patterns of floral construction in ontogeny and phylogeny. *Biol. J. Linn. Soc.* **39,** 153–175 (1990).
12. Endress, P. K. *Diversity and evolutionary biology of tropical flowers*. (Cambridge University Press, 1994).
13. Walker-Larsen, J. & Harder, L. D. The evolution of staminodes in angiosperms: patterns of stamen reduction, loss, and functional re-invention. *Am. J. Bot.* **87,**

1367–1384 (2000).
14. Endress, P. K. The role of inner staminodes in the floral display of some relic Magnoliales. *Plant Syst. Evol.* **146,** 269–282 (1984).
15. Ronse De Craene, L. P. & Smets, E. F. Staminodes: Their morphological and evolutionary significance. *Bot. Rev.* **67,** 351–402 (2001).
16. Endress, P. K. Early floral development and nature of the calyptra in Eupomatiaceae (Magnoliales). *Int. J. Plant Sci.* **164,** 489–503 (2003).
17. Kim, S. *et al.* Sequence and expression studies of A-, B-, and E-class MADS-box homologues in *Eupomatia* (Eupomatiaceae): Support for the bracteate origin of the calyptra. *Int. J. Plant Sci.* **166,** 185–198 (2005).
18. Endress, P. K. Floral structure and evolution in Ranunculanae. *Plant Syst. Evol.* **Suppl.,** 47–61 (1995).
19. Rasmussen, D. A., Kramer, E. M. & Zimmer, E. A. One size fits all? Molecular evidence for a commonly inherited petal identity program in Ranunculales. *Am. J. Bot.* **96,** 96–109 (2009).
20. Ronse De Craene, L. P. *Floral Diagrams: An Aid to Understanding Flower Morphology and Evolution*. (Cambridge University Press, 2010).
21. Endress, P. K. & Doyle, J. A. Ancestral traits and specializations in the flowers of the basal grade of living angiosperms. *Taxon* **64,** 1093–1116 (2015).
22. Murbeck, S. Untersuchungen über den Blütenbau der Papaveraceen. *K. Sven. Vetenskapakademiens Handl.* **50,** 1–168 (1912).
23. Endress, P. K. Evolution and floral diversity: the phylogenetic surroundings of Arabidopsis and Antirrhinum. *Int. J. Plant Sci.* **153,** S106–S122 (1992).
24. Harris, E. Inflorescence and floral ontogeny in asteraceae: A synthesis of historical and current concepts. *Bot. Rev.* **61,** 93–278 (1995).
25. Whipple, C. J., Zanis, M. J., Kellogg, E. A. & Schmidt, R. J. Conservation of B class gene expression in the second whorl of a basal grass and outgroups links the origin of lodicules and petals. *Proc. Natl. Acad. Sci.* **104,** 1081–1086 (2007).
26. Yoshida, H. Is the lodicule a petal: molecular evidence? *Plant Sci.* **184,** 121–128 (2012).
27. Prenner, G. & Rudall, P. J. Comparative ontogeny of the cyathium in Euphorbia (Euphorbiaceae) and its allies: exploring the organ flower inflorescence boundary. *Am. J. Bot.* **94,** 1612–1629 (2007).
28. Rudall, P. J. *et al.* Morphology of Hydatellaceae, an anomalous aquatic family recently recognized as an early-divergent angiosperm lineage. *Am. J. Bot.* **94,** 1073–1092 (2007).
29. Douglas, A. W. & Tucker, S. C. Inflorescence ontogeny and floral organogenesis in Grevilleoideae (Proteaceae), with emphasis on the nature of the flower pairs. *Int. J. Plant Sci.* **157,** 341–372 (1996).
30. Weston, P. H. in *The Families and Genera of Vascular Plants. Volume IX* (ed. Kubitzki, K.) **9,** 364–404 (Springer-Verlag, 2006).
31. Endress, P. K. Floral phyllotaxis and floral evolution. *Bot. Jahrbücher für Syst.* **108,** 417–438 (1987).

32. Endress, P. K. & Doyle, J. A. Floral phyllotaxis in basal angiosperms: development and evolution. *Curr. Opin. Plant Biol.* **10,** 52–57 (2007).

33. Kuhlemeier, C. Phyllotaxis. *Trends Plant Sci.* **12,** 143–150 (2007).

34. Endress, P. K. Evolutionary diversification of the flowers in angiosperms. *Am. J. Bot.* **98,** 370–396 (2011).

35. Zhao, L., Bachelier, J., Chang, H., Tian, X. & Ren, Y. Inflorescence and floral development in Ranunculus and three allied genera in Ranunculeae (Ranunculoideae, Ranunculaceae). *Plant Syst. Evol.* **298,** 1057–1071 (2012).

36. Endress, P. K. Flower structure and trends of evolution in eudicots and their major subclades. *Ann. Missouri Bot. Gard.* **97,** 541–583 (2010).

37. Zhang, R.-J. & Schönenberger, J. Early floral development of Pentaphylacaceae (Ericales) and its systematic implications. *Plant Syst. Evol.* **300,** 1547–1560 (2014).

38. Löfstrand, S. D., von Balthazar, M. & Schönenberger, J. Early floral development and androecium organization in the sarracenioid clade (Actinidiaceae, Roridulaceae and Sarraceniaceae) of Ericales. *Bot. J. Linn. Soc.* **180,** 295–318 (2016).

39. Staedler, Y. M. & Endress, P. K. Diversity and lability of floral phyllotaxis in the pluricarpellate families of core Laurales (Gomortegaceae, Atherospermataceae, Siparunaceae, Monimiaceae). *Int. J. Plant Sci.* **170,** 522–550 (2009).

40. Hirmer, M. Zur Kenntnis der Schraubenstellungen im Pflanzenreich [Title translation: On spiral phyllotaxis in the plant kingdom]. *Planta* **14,** 132–206 (1931).

41. Schöffel, K. Untersuchungen über den Blütenbau der Ranunculaceen. *Planta* **17,** 315–371 (1932).

42. Erbar, C. & Leins, P. Distribution of the character states ‘early sympetaly’ and ‘late sympetaly’ within the ‘Sympetalae Tetracyclicae’ and presumably allied groups. *Bot. Acta* **109,** 427–440 (1996).

43. Sauquet, H. *et al.* Zygomorphy evolved from disymmetry in Fumarioideae (Papaveraceae, Ranunculales): new evidence from an expanded molecular phylogenetic framework. *Ann. Bot.* **115,** 895–914 (2015).

44. Hufford, L. in *The Anther: Form, Function, and Phylogeny* (eds. D’Arcy, W. G. & Keating, R. C.) 58–91 (Cambridge University Press, 1996).

45. Endress, P. K. The Chloranthaceae: reproductive structures and phylogenetic position. *Bot. Jahrbücher für Syst.* **109,** 153–226 (1987).

46. Barthlott, W. & Hunt, D. R. in *The Families and Genera of Vascular Plants. Vol. II. Flowering Plants: Dicotyledons* (eds. Kubitzki, K., Rohwer, J. & Bittrich, V.) **2,** 161–197 (Springer Berlin Heidelberg, 1993).

47. Sauquet, H. Androecium diversity and evolution in Myristicaceae (Magnoliales), with a description of a new Malagasy genus, *Doyleanthus* gen. nov. *Am. J. Bot.* **90,** 1293–1305 (2003).

48. von Balthazar, M., Alverson, W. S., Schönenberger, J. & Baum, D. A. Comparative floral development and androecium structure in Malvoideae

(Malvaceae s.l.). *Int. J. Plant Sci.* **165,** 445–473 (2004).

49. Perrier de la Bâthie, H. Guttifères. *Flore Madagascar des Comores* **136,** 1–96 (1951).

50. Tsou, C.-H. & Mori, S. A. Floral organogenesis and floral evolution of the Lecythidoideae (Lecythidaceae). *Am. J. Bot.* **94,** 716–736 (2007).

51. Ren, Y., Chang, H.-L., Tian, X.-H., Song, P. & Endress, P. Floral development in Adonideae (Ranunculaceae). *Flora* **204,** 506–517 (2009).

52. Ren, Y., Gu, T. & Chang, H. Floral development of Dichocarpum, Thalictrum, and *Aquilegia* (Thalictroideae, Ranunculaceae). *Plant Syst. Evol.* **292,** 203–213 (2011).

53. Matthews, M. L., Amaral, M. D. C. E. & Endress, P. K. Comparative floral structure and systematics in Ochnaceae s.l. (Ochnaceae, Quiinaceae and Medusagynaceae; Malpighiales). *Bot. J. Linn. Soc.* **170,** 299–392 (2012).

54. Xu, F. & Ronse De Craene, L. Floral ontogeny of Annonaceae: evidence for high variability in floral form. *Ann. Bot.* **106,** 591–605 (2010).

55. Endress, P. K. & Armstrong, J. E. Floral development and floral phyllotaxis in Anaxagorea (Annonaceae). *Ann. Bot.* **108,** 835–845 (2011).

56. Endress, P. K. & Hufford, L. D. The diversity of stamen structures and dehiscence patterns among Magnoliidae. *Bot. J. Linn. Soc.* **100,** 45–85 (1989).

57. Venkatesh, C. S. The curious anther of Bixa--its structure and dehiscence. *Am. Midl. Nat.* **55,** 473–476 (1956).

58. Hermann, P. M. & Palser, B. F. Stamen development in the Ericaceae. I. Anther wall, microsporogenesis, inversion, and appendages. *Am. J. Bot.* **87,** 934–957 (2000).

59. Meng, A., Zhang, Z., Li, J., Craene Louis Ronse De & Wang, H. Floral development of *Stephania* (Menispermaceae): Impact of organ reduction on symmetry. *Int. J. Plant Sci.* **173,** 861–874 (2012).

60. Rudall, P. J. Monocot pseudanthia revisited: Floral structure of the mycoheterotrophic family Triuridaceae. *Int. J. Plant Sci.* **164,** S307–S320 (2003).

61. Stauffer, F. W., Rutishauser, R. & Endress, P. K. Morphology and development of the female flowers in *Geonoma interrupta* (Arecaceae). *Am. J. Bot.* **89,** 220–229 (2002).

62. Endress, P. K. Multicarpellate gynoecia in angiosperms: occurrence, development, organization and architectural constraints. *Bot. J. Linn. Soc.* **174,** 1–43 (2014).

63. Magallón, S., Gómez-Acevedo, S., Sánchez-Reyes, L. L. & Hernández-Hernández, T. A metacalibrated time-tree documents the early rise of flowering plant phylogenetic diversity. *New Phytol.* **207,** 437–453 (2015).

64. Soltis, D. E. *et al.* Angiosperm phylogeny: 17 genes, 640 taxa. *Am. J. Bot.* **98,** 704–730 (2011).

65. Jansen, R. K. *et al.* Analysis of 81 genes from 64 plastid genomes resolves relationships in angiosperms and identifies genome-scale evolutionary patterns. *Proc. Natl. Acad. Sci. USA* **104,** 19369–19374 (2007).

66. Moore, M. J., Bell, C. D., Soltis, P. S. & Soltis, D. E. Using plastid genome-scale data to resolve enigmatic relationships among basal angiosperms. *Proc. Natl. Acad. Sci. USA* **104,** 19363–19368 (2007).

67. Moore, M. J., Soltis, P. S., Bell, C. D., Burleigh, J. G. & Soltis, D. E. Phylogenetic analysis of 83 plastid genes further resolves the early diversification of eudicots. *Proc. Natl. Acad. Sci. USA* **107,** 4623–4628 (2010).

68. Ruhfel, B. R., Gitzendanner, M. A., Soltis, P. S., Soltis, D. E. & Burleigh, J. G. From algae to angiosperms-inferring the phylogeny of green plants (Viridiplantae) from 360 plastid genomes. *BMC Evol. Biol.* **14,** 23 (2014).

69. Wickett, N. J. *et al.* Phylotranscriptomic analysis of the origin and early diversification of land plants. *Proc. Natl. Acad. Sci. USA* **111,** E4859–E4868 (2014).

70. Zeng, L. *et al.* Resolution of deep angiosperm phylogeny using conserved nuclear genes and estimates of early divergence times. *Nat. Commun.* **5,** 4956 (2014).

71. Cantino, P. D. *et al.* Towards a phylogenetic nomenclature of Tracheophyta. *Taxon* **56,** E1–E44 (2007).

72. Beaulieu, J. M., O'Meara, B. C., Crane, P. & Donoghue, M. J. Heterogeneous rates of molecular evolution and diversification could explain the Triassic age estimate for angiosperms. *Syst. Biol.* **64,** 869–878 (2015).

73. Foster, C. S. P. *et al.* Evaluating the impact of genomic data and priors on Bayesian estimates of the angiosperm evolutionary timescale. *Syst. Biol.* **66,** 338–351 (2017).

74. Smith, S. A., Beaulieu, J. M. & Donoghue, M. J. An uncorrelated relaxed-clock analysis suggests an earlier origin for flowering plants. *Proc. Natl. Acad. Sci. USA* **107,** 5897–5902 (2010).

75. Magallón, S. Using fossils to break long branches in molecular dating: a comparison of relaxed clocks applied to the origin of angiosperms. *Syst. Biol.* **59,** 384–399 (2010).

76. Schluter, D., Price, T., Mooers, A. O. & Ludwig, D. Likelihood of ancestor states in adaptive radiation. *Evolution* **51,** 1699–1711 (1997).

77. Lewis, P. O. A likelihood approach to estimating phylogeny from discrete morphological character data. *Syst. Biol.* **50,** 913–925 (2001).

78. Endress, P. K. & Doyle, J. A. Reconstructing the ancestral angiosperm flower and its initial specializations. *Am. J. Bot.* **96,** 22–66 (2009).

79. Doyle, J. A. & Endress, P. K. in *Flowers on the Tree of Life* (eds. Wanntorp, L. & Ronse De Craene, L. P.) 88–119 (Cambridge University Press, 2011).

80. Ronse De Craene, L. P., Soltis, P. S. & Soltis, D. E. Evolution of floral structures in basal angiosperms. *Int. J. Plant Sci.* **164,** S329–S363 (2003).

81. Doyle, J. A. & Endress, P. K. Morphological phylogenetic analysis of basal angiosperms: comparison and combination with molecular data. *Int. J. Plant Sci.* **161,** S121–S153 (2000).

82. Zanis, M. J., Soltis, P. S., Qiu, Y. L., Zimmer, E. & Soltis, D. E. Phylogenetic analyses and perianth evolution in basal angiosperms. *Ann. Missouri Bot.*

Gard. **90,** 129–150 (2003).

83. Soltis, D. E., Soltis, P. S., Endress, P. K. & Chase, M. W. *Phylogeny and evolution of angiosperms*. (Sinauer Associates, 2005).
84. Pagel, M., Meade, A. & Barker, D. Bayesian estimation of ancestral character states on phylogenies. *Syst. Biol.* **53,** 673–684 (2004).
85. Anger, N., Fogliani, B., Scutt, C. P. & Gâteblé, G. Dioecy in *Amborella trichopoda*: evidence for genetically based sex determination and its consequences for inferences of the breeding system in early angiosperms. *Ann. Bot.* **119,** 591–597 (2017).
86. Endress, P. K. The flowers in extant basal angiosperms and inferences on ancestral flowers. *Int. J. Plant Sci.* **162,** 1111–1140 (2001).
87. Saarela, J. M. *et al.* Hydatellaceae identified as a new branch near the base of the angiosperm phylogenetic tree. *Nature* **446,** 312–315 (2007).
88. Sokoloff, D. D., Remizowa, M. V, Macfarlane, T. D. & Rudall, P. J. Classification of the early-divergent angiosperm family Hydatellaceae: one genus instead of two, four new species and sexual dimorphism in dioecious taxa. *Taxon* **57,** 179–200 (2008).
89. Iles, W. J. D. *et al.* Molecular phylogenetics of Hydatellaceae (Nymphaeales): Sexual-system homoplasy and a new sectional classification. *Am. J. Bot.* **99,** 663–676 (2012).
90. Schneider, E. L., Tucker, S. C. & Williamson, P. S. Floral development in the Nymphaeales. *Int. J. Plant Sci.* **164,** S279–S292 (2003).
91. Maddison, W. P., Midford, P. E. & Otto, S. P. Estimating a binary character's effect on speciation and extinction. *Syst. Biol.* **56,** 701–710 (2007).
92. Endress, P. K. The early evolution of the angiosperm flower. *Trends Ecol. Evol.* **2,** 300–304 (1987).
93. O'Meara, B. C., Ané, C., Sanderson, M. J. & Wainwright, P. C. Testing for different rates of continuous trait evolution using likelihood. *Evolution* **60,** 922–33 (2006).
94. Zanne, A. E. *et al.* Three keys to the radiation of angiosperms into freezing environments. *Nature* **506,** 89–92 (2014).
95. Reyes, E., Sauquet, H. & Nadot, S. Perianth symmetry changed at least 199 times in angiosperm evolution. *Taxon* **65,** 945–964 (2016).
96. Feild, T. S., Arens, N. C. & Dawson, T. E. The ancestral ecology of angiosperms: emerging perspectives from extant basal lineages. *Int. J. Plant Sci.* **164,** S129–S142 (2003).
97. Feild, T. S. *et al.* Fossil evidence for Cretaceous escalation in angiosperm leaf vein evolution. *Proc. Natl. Acad. Sci. USA* **108,** 8363–8366 (2011).
98. de Boer, H. J., Eppinga, M. B., Wassen, M. J. & Dekker, S. C. A critical transition in leaf evolution facilitated the Cretaceous angiosperm revolution. *Nat. Commun.* **3,** 1221 (2012).
99. Feild, T. S., Arens, N. C., Doyle, J. A., Dawson, T. E. & Donoghue, M. J. Dark and disturbed: a new image of early angiosperm ecology. *Paleobiology* **30,** 82–107 (2004).

100. Feild, T. S. & Arens, N. C. Form, function and environments of the early angiosperms: merging extant phylogeny and ecophysiology with fossils. *New Phytol.* **166,** 383–408 (2005).

101. Doyle, J. A. Early evolution of angiosperm pollen as inferred from molecular and morphological phylogenetic analyses. *Grana* **44,** 227–251 (2005).

102. Lu, L., Wortley, A. H., Li, D., Wang, H. & Blackmore, S. Evolution of angiosperm pollen. 2. The basal angiosperms. *Ann. Missouri Bot. Gard.* **100,** 227–269 (2015).

103. Friedman, W. E. & Ryerson, K. C. Reconstructing the ancestral female gametophyte of angiosperms: Insights from Amborella and other ancient lineages of flowering plants. *Am. J. Bot.* **96,** 129–143 (2009).

104. Thien, L. B. *et al.* Pollination biology of basal angiosperms (ANITA grade). *Am. J. Bot.* **96,** 166–182 (2009).

105. Endress, P. K. The evolution of floral biology in basal angiosperms. *Philos. Trans. R. Soc. B Biol. Sci.* **365,** 411–421 (2010).

106. Eriksson, O. Evolution of seed size and biotic seed dispersal in angiosperms: paleoecological and neoecological evidence. *Int. J. Plant Sci.* **169,** 863–870 (2008).

107. Rudall, P. J. Identifying key features in the origin and early diversification of angiosperms. *Annu. Plant Rev.* **45,** 163–188 (2013).

108. Remizowa, M., Sokoloff, D. & Rudall, P. J. Evolution of the monocot gynoecium: Evidence from comparative morphology and development in *Tofieldia*, *Japonolirion*, *Petrosavia* and *Narthecium*. *Plant Syst. Evol.* **258,** 183–209 (2006).

109. Remizowa, M. V., Sokoloff, D. D. & Rudall, P. J. Evolutionary history of the monocot flower. *Ann. Missouri Bot. Gard.* **97,** 617–645 (2010).

110. Armbruster, W. S. in *Plant-pollinator interactions: from specialization to generalization* (eds. Waser, M. & Ollerton, J.) 260–282 (The University of Chicago Press, 2006).

111. Chartier, M. *et al.* The floral morphospace – a modern comparative approach to study angiosperm evolution. *New Phytol.* **204,** 841–853 (2014).

112. O'Meara, B. C. *et al.* Non-equilibrium dynamics and floral trait interactions shape extant angiosperm diversity. *Proc. R. Soc. London B Biol. Sci.* **283,** (2016).

113. Chartier, M. *et al.* How (much) do flowers vary? Unbalanced disparity among flower functional modules and a mosaic pattern of morphospace occupation in the order Ericales. *Proc. R. Soc. B Biol. Sci.* **284,** 20170066 (2017).

114. Stebbins, G. Natural selection and the differentiation of angiosperm families. *Evolution* **5,** 299–324 (1951).

115. Diggle, P. K. Modularity and intra-floral integration in metameric organisms: plants are more than the sum of their parts. *Philos. Trans. R. Soc. B Biol. Sci.* **369,** (2014).

116. Armbruster, W. S., Pélabon, C., Bolstad, G. H. & Hansen, T. F. Integrated phenotypes: understanding trait covariation in plants and animals. *Philos.*

Trans. R. Soc. B Biol. Sci. **369,** (2014).

117. Endress, P. K. Origins of flower morphology. *J. Exp. Zool.* **291,** 105–115 (2001).
118. Endress, P. K. Angiosperm floral evolution: morphological developmental framework. *Adv. Bot. Res.* **44,** 1–61 (2006).
119. Clavel, J., Escarguel, G. & Merceron, G. mvmorph: an r package for fitting multivariate evolutionary models to morphometric data. *Methods Ecol. Evol.* **6,** 1311–1319 (2015).
120. Adams, D. C. & Felice, R. N. Assessing trait covariation and morphological integration on phylogenies using evolutionary covariance matrices. *PLoS One* **9,** 1–8 (2014).
121. Bartoszek, K., Pienaar, J., Mostad, P., Andersson, S. & Hansen, T. F. A phylogenetic comparative method for studying multivariate adaptation. *J. Theor. Biol.* **314,** 204–215 (2012).
122. Jabbour, F., Damerval, C. & Nadot, S. Evolutionary trends in the flowers of Asteridae: Is polyandry an alternative to zygomorphy? *Ann. Bot.* **102,** 153–165 (2008).
123. Maddison, W. P. & FitzJohn, R. G. The unsolved challenge to phylogenetic correlation tests for categorical characters. *Syst. Biol.* **64,** 127–136 (2015).
124. Smith, S. D. Pleiotropy and the evolution of floral integration. *New Phytol.* **209,** 80–85 (2016).
125. Pruitt, R., Chang, C., Pang, P. & Meyerowitz, E. in *Genetic Regulation of Development: 45th Symposium of the Society for Developmental Biology* (ed. Loomis, W. R.) 327–338 (Alan R. Liss, 1987).
126. Komaki, M. K., Okada, K., Nshino, E. & Shimura, Y. Isolation and characterization of novel mutants of *Arabidopsis thaliana* defective in flower development. *De* **104,** 195–203 (1988).
127. Bowman, J. L., Smyth, D. R. & Meyerowitz, E. M. Genes directing flower development in *Arabidopsis*. *Plant Cell* **1,** 37–52 (1989).
128. Coen, E. S. & Meyerowitz, E. M. The war of the whorls: genetic interactions controlling flower development. *Nature* **353,** 31–37 (1991).
129. Weigel, D. & Meyerowitz, E. M. The ABCs of floral homeotic genes. *Cell* **78,** 203–209 (1994).
130. Pelaz, S., Ditta, G. S., Baumann, E., Wisman, E. & Yanofsky, M. F. B and C floral organ identity functions require SEPALLATA MADS-box genes. *Nature* **405,** 200–203 (2000).
131. Colombo, L. *et al.* The petunia MADS box gene FBP11 determines ovule identity. *Plant Cell* **7,** 1859–1868 (1995).
132. Soltis, D. E., Chanderbali, A. S., Kim, S., Buzgo, M. & Soltis, P. S. The ABC model and its applicability to basal angiosperms. *Ann. Bot.* **100,** 155–163 (2007).
133. Litt, A. & Irish, V. F. Duplication and diversification in the APETALA1/FRUITFULL floral homeotic gene lineage: Implications for the evolution of floral development. *Genetics* **165,** 821–833 (2003).

134. Kim, S. *et al.* Phylogeny and diversification of B-function MADS-box genes in angiosperms: evolutionary and functional implications of a 260-million-year-old duplication. *Am. J. Bot.* **91,** 2102–2118 (2004).

135. Kim, S. *et al.* Expression of floral MADS-box genes in basal angiosperms: implications for the evolution of floral regulators. *Plant J.* **43,** 724–744 (2005).

136. Kim, S., Soltis, P. S., Wall, K. & Soltis, D. E. Phylogeny and Domain Evolution in the APETALA2-like Gene Family. *Mol. Biol. Evol.* **23,** 107–120 (2006).

137. Stellari, G. M., Jaramillo, M. A. & Kramer, E. M. Evolution of the *APETALA3* and *PISTILLATA* lineages of MADS-box-containing genes in the basal angiosperms. *Mol. Biol. Evol.* **21,** 506–519 (2004).

138. Zahn, L. M. *et al.* The Evolution of the SEPALLATA Subfamily of MADS-Box Genes: A Preangiosperm Origin With Multiple Duplications Throughout Angiosperm History. *Genetics* **169,** 2209–2223 (2005).

139. Soltis, P. S. *et al.* in *Advances in Botanical Research* **Volume 44,** 483–506 (Academic Press, 2006).

140. Kramer, E. M. & Irish, V. F. Evolution of the petal and stamen developmental programs: evidence from comparative studies of the lower eudicots and basal angiosperms. *Int. J. Plant Sci.* **161,** S29–S40 (2000).

141. Kramer, E. M. & Jaramillo, M. A. Genetic basis for innovations in floral organ identity. *J. Exp. Zool. Part B Mol. Dev. Evol.* **304B,** 526–535 (2005).

142. Jaramillo, M. A. & Kramer, E. M. The role of developmental genetics in understanding homology and morphological evolution in plants. *Int. J. Plant Sci.* **168,** 61–72 (2007).

143. Ronse De Craene, L. P. & Brockington, S. F. Origin and evolution of petals in angiosperms. *Plant Ecol. Evol.* **146,** 5–25 (2013).

144. Ronse De Craene, L. P. Homology and evolution of petals in the core eudicots. *Syst. Bot.* **33,** 301–325 (2008).

145. Ronse De Craene, L. P. Are petals sterile stamens or bracts? The origin and evolution of petals in the core eudicots. *Ann. Bot.* **100,** 621–630 (2007).

146. Rasmussen, C. & Cameron, S. A. Global stingless bee phylogeny supports ancient divergence, vicariance, and long distance dispersal. *Biol. J. Linn. Soc.* **99,** 206–232 (2010).

147. Karrer, A. Blütenentwicklung und systematische Stellung der Papaveraceae und Capparaceae. Doctoral dissertation, University of Zurich, Zurich, Switzerland. (1991).

148. Drinnan, A. N., Crane, P. R. & Hoot, S. B. Patterns of floral evolution in the early diversification of non-magnoliid dicotyledons (eudicots). *Plant Syst. Evol.* **8,** 93–122 (1994).

149. Douglas, A. W. The Developmental Basis of Morphological Diversification and Synorganization in Flowers of Conospermeae (Stirlingia and Conosperminae: Proteaceae). *Int. J. Plant Sci.* **158,** S13–S48 (1997).

150. von Balthazar, M. & Endress, P. K. Development of inflorescences and flowers in Buxaceae and the problem of perianth interpretation. *Int. J. Plant Sci.* **163,** 847–876 (2002).

151. Soltis, D. E. *et al.* Gunnerales are sister to other core eudicots: implications for the evolution of pentamery. *Am. J. Bot.* **90,** 461–470 (2003).

152. Douglas, A. W. & Tucker, S. C. Comparative floral ontogenies among Persoonioideae including Bellendena (Proteaceae). *Am. J. Bot.* **83,** 1528–1555 (1996).

153. Ronse De Craene, L. P. & Wanntorp, L. Evolution of floral characters in *Gunnera* (Gunneraceae). *Syst. Bot.* **31,** 671–688 (2006).

154. Chen, L., Ren, Y., Endress, P. K., Tian, X. H. & Zhang, X. H. Floral organogenesis in Tetracentron sinense (Trochodendraceae) and its systematic significance. *Plant Syst. Evol.* **264,** 183–193 (2007).

155. González, F. & Bello, M. A. Intra-individual variation of flowers in *Gunnera* subgenus *Panke* (Gunneraceae) and proposed apomorphies for Gunnerales. *Bot. J. Linn. Soc.* **160,** 262–283 (2009).

156. Ronse De Craene, L. & Wanntorp, L. Morphology and anatomy of the flower of *Meliosma* (Sabiaceae): implications for pollination biology. *Plant Syst. Evol.* **271,** 79–91 (2008).

157. von Balthazar, M. & Schönenberger, J. Floral structure and organization in Platanaceae. *Int. J. Plant Sci.* **170,** 210–225 (2009).

158. Wanntorp, L. & Ronse De Craene, L. P. Flower development of *Meliosma* (Sabiaceae): evidence for multiple origins of pentamery in the eudicots. *Am. J. Bot.* **94,** 1828–1836 (2007).

159. Ronse De Craene, L. P., Quandt, D. & Wanntorp, L. Floral development of *Sabia* (Sabiaceae): Evidence for the derivation of pentamery from a trimerous ancestry. *Am. J. Bot.* **102,** 336–349 (2015).

160. Eichler, A. W. *Blüthendiagramme II*. (Engelmann, 1878).

161. Ronse Decraene, L. P. & Smets, E. The systematic relationship between Begoniaceae and Papaveraceae: A comparative study of their floral development. *Bull. du Jard. Bot. Natl. Belgique / Bull. van Natl. Plantentuin van België* **60,** 229–273 (1990).

162. Ronse De Craene, L. P. & Smets, E. F. Merosity in flowers: Definition, origin, and taxonomic significance. *Plant Syst. Evol.* **191,** 83–104 (1994).

163. Ronse De Craene, L. Meristic changes in flowering plants: How flowers play with numbers. *Flora* **221,** 22–37 (2016).

164. Jabbour, F., Ronse De Craene, L. P., Nadot, S. & Damerval, C. Establishment of zygomorphy on an ontogenic spiral and evolution of perianth in the tribe Delphinieae (Ranunculaceae). *Ann. Bot.* **104,** 809–822 (2009).

165. Damerval, C. & Nadot, S. Evolution of perianth and stamen characteristics with respect to floral symmetry in Ranunculales. *Ann. Bot.* **100,** 631–640 (2007).

166. Salisbury, E. J. Variation in *Eranthis hyemalis*, *Ficaria verna*, and other members of the Ranunculaceae, with special reference to trimery and the origin of the perianth. *Ann. Bot.* **os-33,** 47–79 (1919).

167. Manchester, S. R. Vegetative and reproductive morphology of an extinct plane tree (Platanaceae) from the Eocene of Western North America. *Bot. Gaz.* **147,** 200–226 (1986).

168. Friis, E. M., Crane, P. R. & Pedersen, K. R. Reproductive structures of Cretaceous Platanaceae. *Biol Medd K Dan Vidensk Selsk* **31,** 1–56 (1988).

169. Magallón-Puebla, S., Herendeen, P. S. & Crane, P. R. *Quadriplatanus georgianus* gen. et sp. nov.: staminate and pistillate platanaceous flowers from the Late Cretaceous (Coniacian-Santonian) of Georgia, U.S.A. *Int. J. Plant Sci.* **158,** 373–394 (1997).

170. Crane, P. R., Friis, E. M. & Pedersen, K. R. Lower Cretaceous angiosperm flowers: Fossil evidence on early radiation of dicotyledons. *Science* **232,** 852–854 (1986).

171. Cutter, E. G. Recent experimental studies of the shoot apex and shoot morphogenesis. *Bot. Rev.* **31,** 7–113 (1965).

172. Drinnan, A. N., Crane, P. R., Friis, E. M. & Pedersen, K. R. Angiosperm flowers and tricolpate pollen of buxaceous affinity from the Potomac Group (Mid-Cretaceous) of Eastern North America. *Am. J. Bot.* **78,** 153–176 (1991).

173. Endress, P. K. Development and evolution of extreme synorganization in angiosperm flowers and diversity: a comparison of Apocynaceae and Orchidaceae. *Ann. Bot.* **117,** 749–767 (2016).

174. Stebbins, G. *Flowering plants. Evolution above the species level*. (Harvard University Press, 1974).

175. Endress, P. K. Syncarpy and alternative modes of escaping disadvantages of apocarpy in primitive angiosperms. *Taxon* **31,** 48–52 (1982).

176. Endress, P. K. & Igersheim, A. Gynoecium structure and evolution in basal angiosperms. *Int. J. Plant Sci.* **161,** S211–S223 (2000).

177. Armbruster, W. S., Debevec, E. M. & Willson, M. F. Evolution of syncarpy in angiosperms: theoretical and phylogenetic analyses of the effects of carpel fusion on offspring quantity and quality. *J. Evol. Biol.* **15,** 657–672 (2002).

178. Chen, J. N., Chen, D., Gituru, W. R., Wang, Q. F. & Guo, Y. H. Evolution of apocarpy in Alismatidae using phylogenetic evidence from chloroplast rbcL gene sequence data. *Bot. Bull. Acad. Sin.* **45,** 33–40 (2004).

179. Friis, E. M., Crane, P. R. & Pedersen, K. R. *Early flowers and angiosperm evolution*. (Cambridge University Press, 2011).
