# Supplementary material for: The ancestral flower of angiosperms and its early diversification
Source: Nat Commun. 2017 Aug 1;8:16047. doi: 10.1038/ncomms16047 (PMC5543309; doi:10.1038/ncomms16047)

MP ancestral state reconstruction using ancestral.pars
(R:phangorn)
100_A. Functional sex of flowers (D2d), 98 steps

- bisexual
- unisexual

Node	MP state(s)
Angiospermae	bisexual / unisexual
Mesangiospermae	bisexual
Magnoliidae	bisexual
Monocotyledonae	bisexual
Eudicotyledonae	bisexual
Commelinidae	bisexual
Pentapetalae	bisexual
Superasteridae	bisexual
Asteridae	bisexual
Lamiidae	bisexual
Campanulidae	bisexual
Superrosidae	bisexual
Rosidae	bisexual
Malvidae	bisexual
Fabidae	bisexual

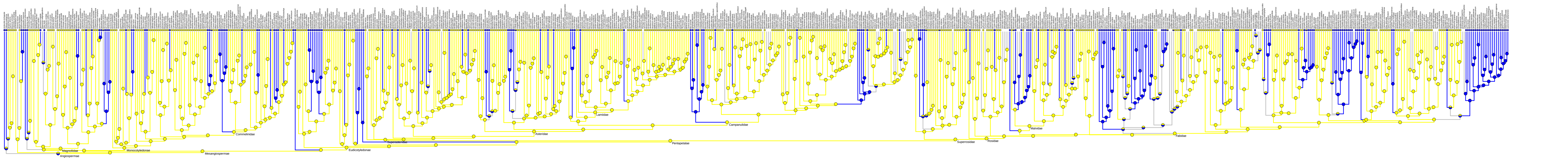

ML ancestral state reconstruction using rayDISC (R:corHMM)
100_A. Functional sex of flowers (D2d), ARDeq model

- bisexual
- unisexual

Node	ML state	Prob
Angiospermae	bisexual	1
Mesangiospermae	bisexual	1
Magnoliidae	bisexual	1
Monocotyledonae	bisexual	1
Eudicotyledonae	bisexual	1
Commelinidae	bisexual	0.9999
Pentapetalae	bisexual	1
Superasteridae	bisexual	1
Asteridae	bisexual	1
Lamiidae	bisexual	1
Campanulidae	bisexual	0.9997
Superrosidae	bisexual	1
Rosidae	bisexual	1
Malvidae	bisexual	0.9998
Fabidae	bisexual	1

Model	LogL	Npar	AIC	AICc	DeltaAICc	w	q01	q10
ARD	−312.19	2	628.38	628.39	1.39	0.22	0.003	6e−04
ARDeq*	−311.5	2	626.99	627.01	0	0.44	0.003	6e−04
ER	−315.39	1	632.78	632.79	5.78	0.02	0.0029	0.0029
UNI01	−312.84	1	627.68	627.69	0.68	0.31	0.003	
UNI10	−355.44	1	712.88	712.88	85.87	0		0.0117

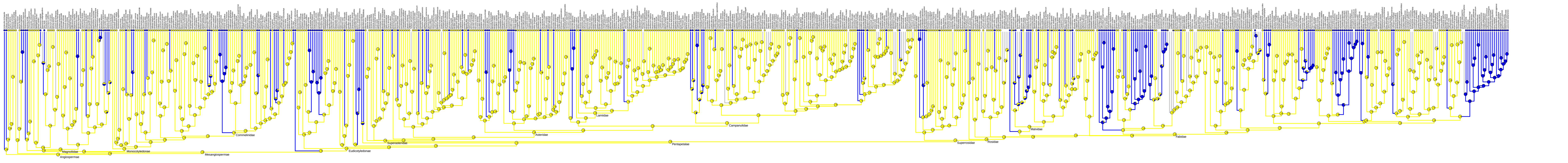

MP ancestral state reconstruction using ancestral.pars (R:phangorn)
100_B. Structural sex of flowers (D2d), 57 steps

- bisexual
- unisexual

Node	MP state(s)
Angiospermae	bisexual
Mesangiospermae	bisexual
Magnoliidae	bisexual
Monocotyledonae	bisexual
Eudicotyledonae	bisexual
Commelinidae	bisexual
Pentapetalae	bisexual
Superasteridae	bisexual
Asteridae	bisexual
Lamiidae	bisexual
Campanulidae	bisexual
Superrosidae	bisexual
Rosidae	bisexual
Malvidae	bisexual
Fabidae	bisexual

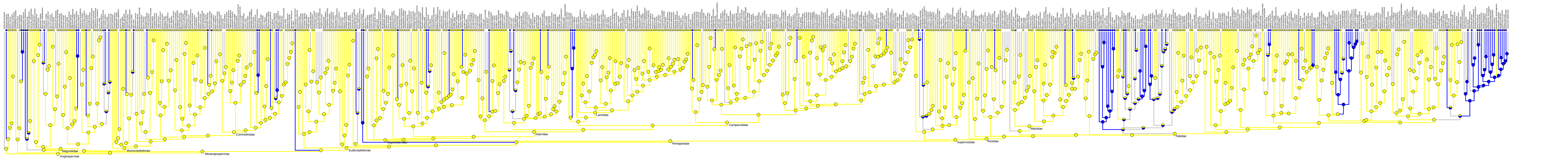

ML ancestral state reconstruction using rayDISC (R:corHMM)
100_B. Structural sex of flowers (D2d), ARDeq model

- bisexual
- unisexual

Node	ML state	Prob
Angiospermae	bisexual	1
Mesangiospermae	bisexual	1
Magnoliidae	bisexual	1
Monocotyledonae	bisexual	1
Eudicotyledonae	bisexual	1
Commelinidae	bisexual	1
Pentapetalae	bisexual	1
Superasteridae	bisexual	1
Asteridae	bisexual	1
Lamiidae	bisexual	0.9999
Campanulidae	bisexual	1
Superrosidae	bisexual	1
Rosidae	bisexual	1
Malvidae	bisexual	1
Fabidae	bisexual	0.9974

Model	LogL	Npar	AIC	AICc	DeltaAICc	w	q01	q10
ARD	−207.74	2	419.48	419.49	1.37	0.28	0.0015	0.0039
ARDeq**	−207.06	2	418.11	418.13	0	0.56	0.0015	0.0039
ER	−209.28	1	420.56	420.57	2.44	0.16	0.0016	0.0016
UNI01	−226.28	1	454.56	454.56	36.43	0	0.0017	
UNI10	−229.76	1	461.52	461.53	43.4	0		0.0164

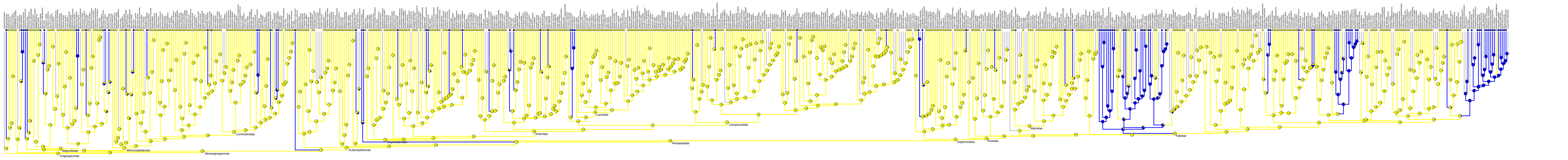

MP ancestral state reconstruction using ancestral.pars (R:phangorn)
102_B. Ovary position (binary) (D2d), 80 steps

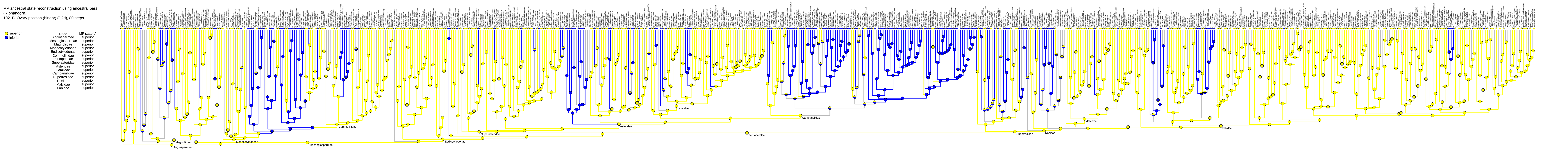

ML ancestral state reconstruction using rayDISC (R:corHMM)
102_B. Ovary position (binary) (D2d), ARDeq model

- superior
- inferior

Node	ML state	Prob
Angiospermae	superior	1
Mesangiospermae	superior	1
Magnoliidae	superior	0.9997
Monocotyledonae	superior	0.9987
Eudicotyledonae	superior	1
Commelinidae	superior	0.7957
Pentapetalae	superior	0.9998
Superasteridae	superior	1
Asteridae	superior	0.9773
Lamiidae	superior	0.9816
Campanulidae	superior	0.8158
Superrosidae	superior	0.9923
Rosidae	superior	0.9925
Malvidae	superior	1
Fabidae	superior	0.9999

Model	LogL	Npar	AIC	AICc	DeltaAICc	w	q01	q10
ARD	−273.09	2	550.19	550.2	1.38	0.23	0.0021	0.0034
ARDeq*	−272.4	2	548.8	548.82	0	0.45	0.0021	0.0034
ER	−273.76	1	549.53	549.53	0.71	0.32	0.0024	0.0024
UNI01	−281.53	1	565.06	565.06	16.24	0	0.0027	
UNI10	−296.7	1	595.39	595.4	46.58	0		0.0099

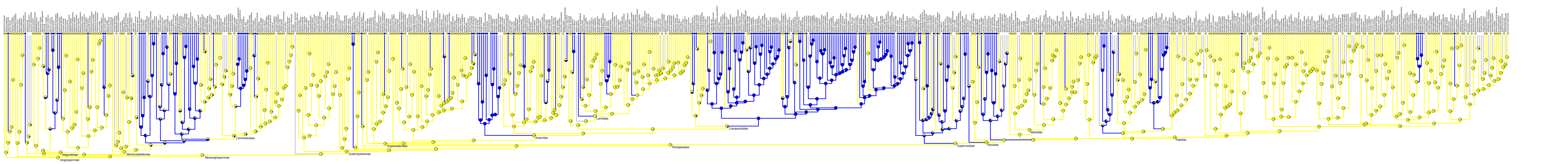

MP ancestral state reconstruction using ancestral.pars
(R:phangorn)
201_A. Perianth presence (D2c), 20 steps

- absent
- present

Node	MP state(s)
Angiospermae	present
Mesangiospermae	present
Magnoliidae	present
Monocotyledonae	present
Eudicotyledonae	present
Commelinidae	present
Pentapetalae	present
Superasteridae	present
Asteridae	present
Lamiidae	present
Campanulidae	present
Superrosidae	present
Rosidae	present
Malvidae	present
Fabidae	present

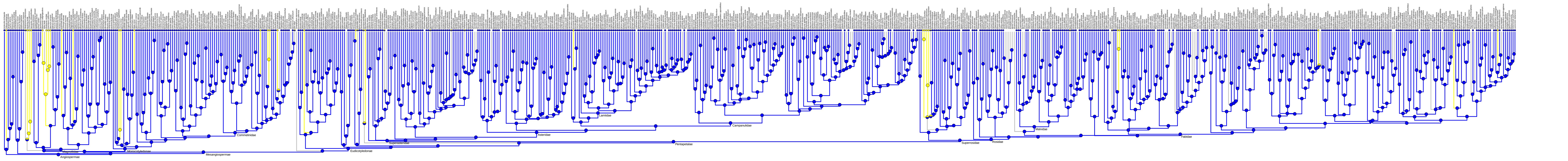

ML ancestral state reconstruction using rayDISC (R:corHMM)
201_A. Perianth presence (D2c), UNI10 model

- absent
- present

Node	ML state	Prob
Angiospermae	present	1
Mesangiospermae	present	1
Magnoliidae	present	1
Monocotyledonae	present	1
Eudicotyledonae	present	1
Commelinidae	present	1
Pentapetalae	present	1
Superasteridae	present	1
Asteridae	present	1
Lamiidae	present	1
Campanulidae	present	1
Superrosidae	present	1
Rosidae	present	1
Malvidae	present	1
Fabidae	present	1

Model	LogL	Npar	AIC	AICc	DeltaAICc	w	q01	q10
ARD	−95.71	2	195.41	195.43	2.01	0.13	0	5e−04
ARDeq	−95.01	2	194.03	194.04	0.62	0.26	0	5e−04
ER	−96.02	1	194.05	194.05	0.63	0.26	5e−04	5e−04
UNI01	−108.51	1	219.02	219.02	25.61	0	0.0222	
UNI10*	−95.71	1	193.41	193.42	0	0.35		5e−04

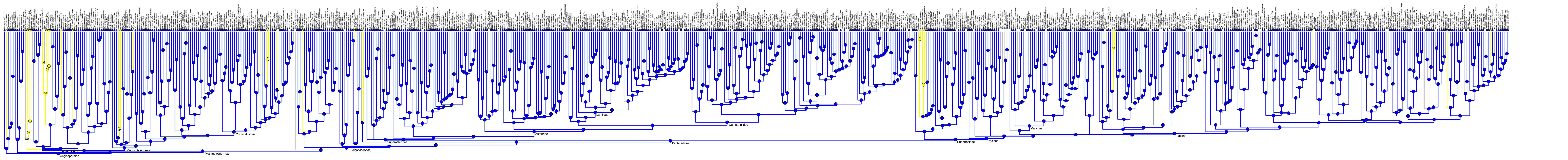

MP ancestral state reconstruction using ancestral.pars
(R:phangorn)
201_B. Number of perianth parts (3−state) (D2c), 85 steps

- one to five (1−5)
- six to ten (6−10)
- more than ten (>10)

Node	MP state(s)
Angiospermae	six to ten (6−10) / more than ten (>10)
Mesangiospermae	six to ten (6−10)
Magnoliidae	six to ten (6−10)
Monocotyledonae	six to ten (6−10)
Eudicotyledonae	six to ten (6−10)
Commelinidae	six to ten (6−10)
Pentapetalae	six to ten (6−10)
Superasteridae	six to ten (6−10)
Asteridae	six to ten (6−10)
Lamiidae	six to ten (6−10)
Campanulidae	six to ten (6−10)
Superrosidae	six to ten (6−10)
Rosidae	six to ten (6−10)
Malvidae	six to ten (6−10)
Fabidae	six to ten (6−10)

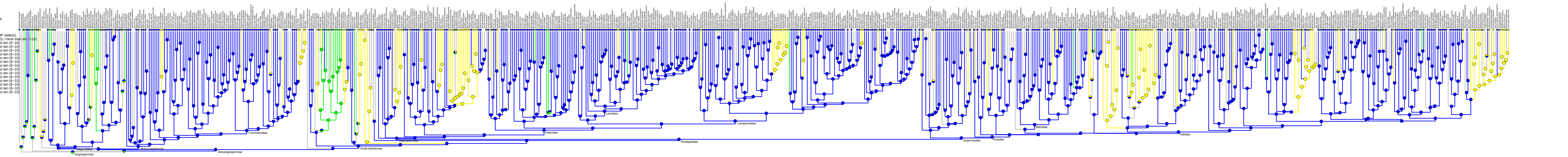

ML ancestral state reconstruction using rayDISC (R:corHMM)
201_B. Number of perianth parts (3−state) (D2c), ARDeq model

- one to five (1−5)
- six to ten (6−10)
- more than ten (>10)

Node	ML state	Prob
Angiospermae	more than ten (>10)	1
Mesangiospermae	more than ten (>10)	1
Magnoliidae	more than ten (>10)	0.9998
Monocotyledonae	six to ten (6−10)	0.6087
Eudicotyledonae	more than ten (>10)	0.9977
Commelinidae	six to ten (6−10)	0.9999
Pentapetalae	six to ten (6−10)	0.9958
Superasteridae	six to ten (6−10)	0.9984
Asteridae	six to ten (6−10)	1
Lamiidae	six to ten (6−10)	0.9999
Campanulidae	six to ten (6−10)	1
Superrosidae	six to ten (6−10)	0.9992
Rosidae	six to ten (6−10)	0.9994
Malvidae	six to ten (6−10)	0.9999
Fabidae	six to ten (6−10)	0.9992

Model	LogL	Npar	AIC	AICc	DeltaAICc	w	q01	···
ARD	−326.44	6	664.88	664.98	2.2	0.25	0.0038	···
ARDeq**	−325.34	6	662.68	662.79	0	0.74	0.0038	···
ER	−351.51	1	705.03	705.03	42.25	0	0.0013	···
SYM	−340.6	3	687.2	687.23	24.44	0	0.002	···
SYMeq	−339.52	3	685.05	685.08	22.29	0	0.002	···
ORD	−332.72	4	673.44	673.49	10.7	0	0.0039	···
ORDeq	−331.62	4	671.24	671.29	8.5	0.01	0.0039	···
ORDSYM	−342.95	2	689.9	689.92	27.13	0	0.002	···
ORDSYMeq	−341.89	2	687.78	687.79	25	0	0.002	···
ORDER	−352.31	1	706.62	706.62	43.83	0	0.0014	···

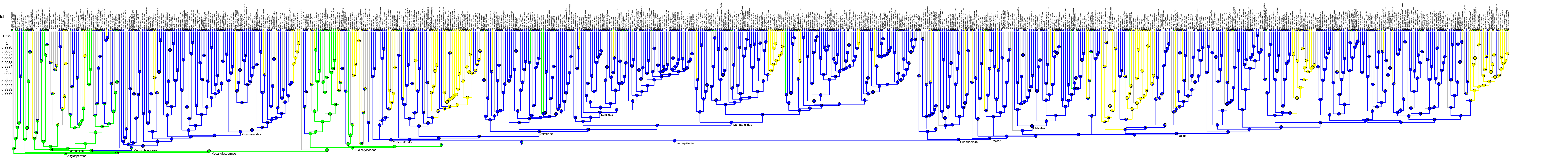

MP ancestral state reconstruction using ancestral.pars
(R:phangorn)
201_C. Number of perianth parts (binary) (D2c), 69 steps

- one to six (1−6)
- more than six (>6)

Node	MP state(s)
Angiospermae	more than six (>6)
Mesangiospermae	one to six (1−6)
Magnoliidae	one to six (1−6) / more than six (>6)
Monocotyledonae	one to six (1−6)
Eudicotyledonae	one to six (1−6)
Commelinidae	one to six (1−6)
Pentapetalae	more than six (>6)
Superasteridae	more than six (>6)
Asteridae	more than six (>6)
Lamiidae	more than six (>6)
Campanulidae	more than six (>6)
Superrosidae	more than six (>6)
Rosidae	more than six (>6)
Malvidae	more than six (>6)
Fabidae	more than six (>6)

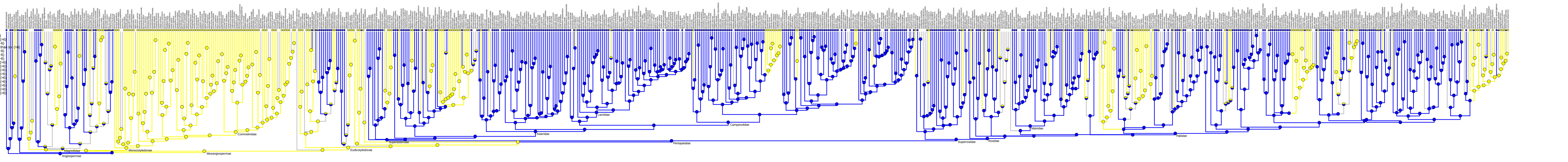

ML ancestral state reconstruction using rayDISC (R:corHMM)
201_C. Number of perianth parts (binary) (D2c), ARDeq model

- one to six (1−6)
- more than six (>6)

Node	ML state	Prob
Angiospermae	more than six (>6)	1
Mesangiospermae	more than six (>6)	1
Magnoliidae	more than six (>6)	0.9998
Monocotyledonae	one to six (1−6)	0.813
Eudicotyledonae	more than six (>6)	1
Commelinidae	one to six (1−6)	1
Pentapetalae	more than six (>6)	1
Superasteridae	more than six (>6)	1
Asteridae	more than six (>6)	1
Lamiidae	more than six (>6)	1
Campanulidae	more than six (>6)	1
Superrosidae	more than six (>6)	1
Rosidae	more than six (>6)	1
Malvidae	more than six (>6)	1
Fabidae	more than six (>6)	1

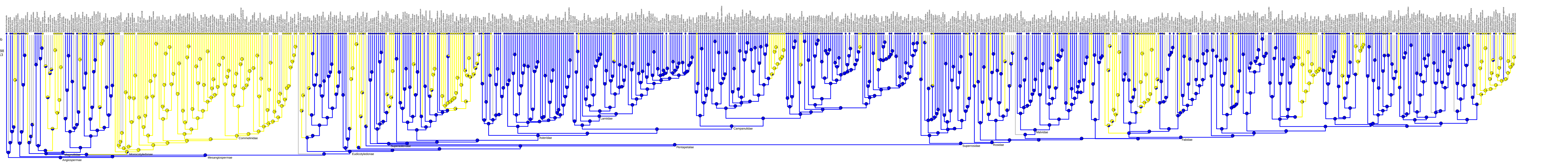

Model	LogL	Npar	AIC	AICc	DeltaAICc	w	q01	q10
ARD	−248.5	2	501	501.02	1.39	0.33	6e−04	0.0026
ARDeq**	−247.81	2	499.62	499.63	0	0.66	6e−04	0.0026
ER	−255.26	1	512.52	512.52	12.89	0	0.0021	0.0021
UNI01	−325.69	1	653.38	653.38	153.75	0	0.0082	
UNI10	−253.14	1	508.28	508.28	8.65	0.01		0.0029

MP ancestral state reconstruction using ancestral.pars
(R:phangorn)
230_A. Perianth phyllotaxy (binary) (D2d), 16 steps

- whorled (yellow)
- spiral (blue)

Node	MP state(s)
Angiospermae	whorled / spiral
Mesangiospermae	whorled
Magnoliidae	whorled
Monocotyledonae	whorled
Eudicotyledonae	whorled
Commelinidae	whorled
Pentapetalae	whorled
Superasteridae	whorled
Asteridae	whorled
Lamiidae	whorled
Campanulidae	whorled
Superrosidae	whorled
Rosidae	whorled
Malvidae	whorled
Fabidae	whorled

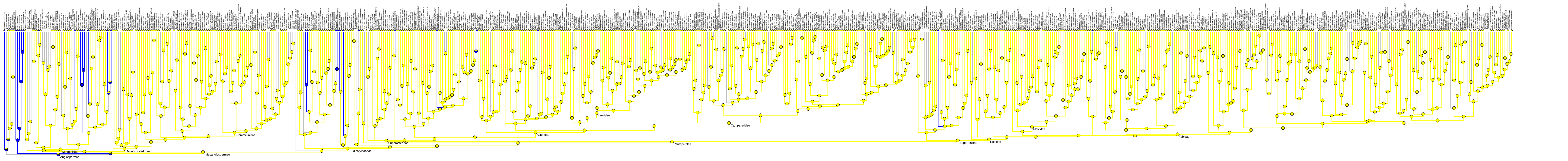

ML ancestral state reconstruction using rayDISC (R:corHMM)
230_A. Perianth phyllotaxy (binary) (D2d), UNI10 model

- whorled
- spiral

Node	ML state	Prob
Angiospermae	spiral	1
Mesangiospermae	spiral	1
Magnoliidae	spiral	1
Monocotyledonae	spiral	0.6562
Eudicotyledonae	spiral	1
Commelinidae	whorled	0.9822
Pentapetalae	spiral	1
Superasteridae	spiral	1
Asteridae	spiral	1
Lamiidae	whorled	0.9696
Campanulidae	whorled	0.8816
Superrosidae	spiral	1
Rosidae	spiral	0.7194
Malvidae	whorled	0.9942
Fabidae	whorled	0.9945

Model	LogL	Npar	AIC	AICc	DeltaAICc	w	q01	q10
ARD	−77.94	2	159.88	159.9	3.9	0.07	4e−04	5e−04
ARDeq	−77.27	2	158.54	158.55	2.56	0.13	4e−04	2e−04
ER	−77.94	1	157.88	157.89	1.89	0.18	4e−04	4e−04
UNI01	−77.98	1	157.96	157.96	1.97	0.17	4e−04	
UNI10*	−76.99	1	155.99	155.99	0	0.46		0.0181

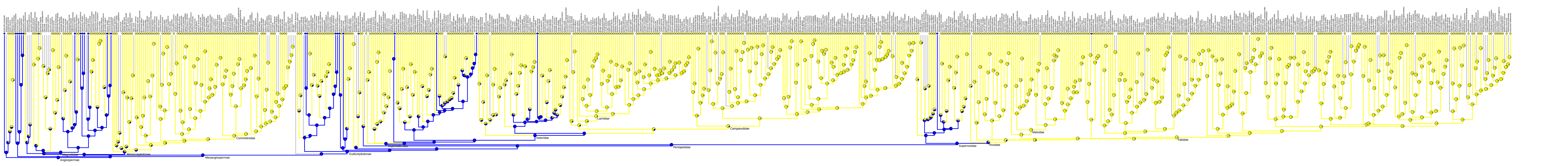

MP ancestral state reconstruction using ancestral.pars
(R:phangorn)
231_A. Number of perianth whorls (D2c), 73 steps

- one (1)
- two (2)
- more than two (>2)

Node	MP state(s)
Angiospermae	more than two (>2)
Mesangiospermae	more than two (>2)
Magnoliidae	more than two (>2)
Monocotyledonae	two (2)
Eudicotyledonae	more than two (>2)
Commelinidae	two (2)
Pentapetalae	two (2)
Superasteridae	two (2)
Asteridae	two (2)
Lamiidae	two (2)
Campanulidae	two (2)
Superrosidae	two (2)
Rosidae	two (2)
Malvidae	two (2)
Fabidae	two (2)

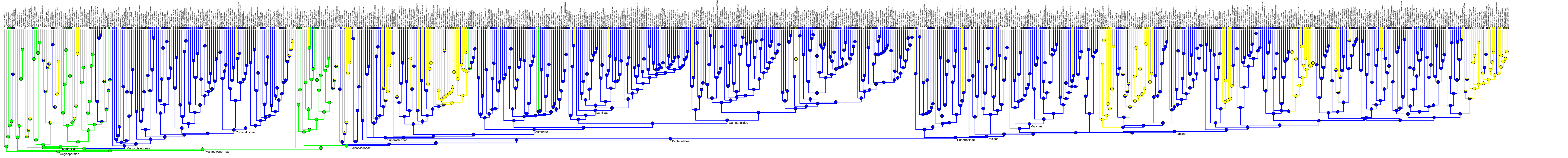

ML ancestral state reconstruction using rayDISC (R:corHMM)
231_A. Number of perianth whorls (D2c), ARDeq model

one (1)
two (2)
more than two (>2)

Node	ML state	Prob
Angiospermae	more than two (>2)	1
Mesangiospermae	more than two (>2)	1
Magnoliidae	more than two (>2)	1
Monocotyledonae	two (2)	0.739
Eudicotyledonae	more than two (>2)	0.9976
Commelinidae	two (2)	1
Pentapetalae	two (2)	0.9998
Superasteridae	two (2)	0.9998
Asteridae	two (2)	0.9999
Lamiidae	two (2)	0.9999
Campanulidae	two (2)	1
Superrosidae	two (2)	1
Rosidae	two (2)	1
Malvidae	two (2)	0.9999
Fabidae	two (2)	0.9978

Model	LogL	Npar	AIC	AICc	DeltaAICc	w	q01	···
ARD	−270.99	6	553.97	554.08	2.2	0.25	0.0046	···
ARDeq**	−269.89	6	551.77	551.88	0	0.75	0.0046	···
ER	−312.34	1	626.68	626.69	74.81	0	0.001	···
SYM	−294.44	3	594.89	594.92	43.03	0	0.0018	···
SYMeq	−293.48	3	592.95	592.98	41.1	0	0.0018	···
ORD	−278.36	4	564.71	564.77	12.88	0	0.0044	···
ORDeq	−277.26	4	562.52	562.57	10.69	0	0.0044	···
ORDSYM	−299.97	2	603.94	603.96	52.08	0	0.0019	···
ORDSYMeq	−298.9	2	601.81	601.82	49.94	0	0.0019	···
ORDER	−314.93	1	631.85	631.86	79.98	0	0.0012	···

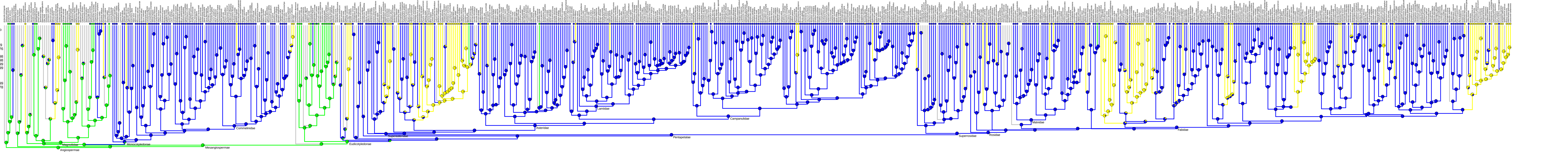

MP ancestral state reconstruction using ancestral.pars
(R:phangorn)
232_A. Perianth merism (4−state) (D2c), 84 steps

- dimerous
- trimerous
- tetramerous
- pentamerous

Node	MP state(s)
Angiospermae	trimerous
Mesangiospermae	trimerous
Magnoliidae	trimerous
Monocotyledonae	trimerous
Eudicotyledonae	dimerous / trimerous
Commelinidae	trimerous
Pentapetalae	pentamerous
Superasteridae	pentamerous
Asteridae	pentamerous
Lamiidae	pentamerous
Campanulidae	pentamerous
Superrosidae	pentamerous
Rosidae	pentamerous
Malvidae	pentamerous
Fabidae	pentamerous

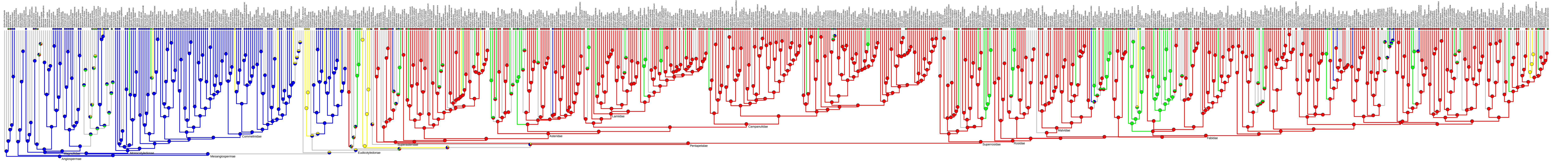

ML ancestral state reconstruction using rayDISC (R:corHMM)
232_A. Perianth merism (4−state) (D2c), SYMeq model

- dimerous (yellow)
- trimerous (blue)
- tetramerous (green)
- pentamerous (red)

Node	ML state	Prob
Angiospermae	trimerous	1
Mesangiospermae	trimerous	1
Magnoliidae	trimerous	1
Monocotyledonae	trimerous	1
Eudicotyledonae	trimerous	0.9685
Commelinidae	trimerous	1
Pentapetalae	pentamerous	0.9997
Superasteridae	pentamerous	1
Asteridae	pentamerous	1
Lamiidae	pentamerous	1
Campanulidae	pentamerous	1
Superrosidae	pentamerous	1
Rosidae	pentamerous	1
Malvidae	pentamerous	1
Fabidae	pentamerous	1

Model	LogL	Npar	AIC	AICc	DeltaAICc	w	q01	···
ARD	−323.89	12	671.77	672.17	11.22	0	0	···
ARDeq	−322.55	12	669.09	669.49	8.54	0.01	0	···
ER	−360.43	1	722.86	722.86	61.91	0	8e−04	···
SYM	−325.81	6	663.61	663.72	2.76	0.2	0.0013	···
SYMeq**	−324.42	6	660.85	660.96	0	0.79	0.0013	···
ORD	−336.79	6	685.59	685.7	24.74	0	0	···
ORDeq	−335.83	6	683.65	683.76	22.8	0	0	···
ORDSYM	−344.4	3	694.8	694.83	33.87	0	0.002	···
ORDSYMeq	−343.03	3	692.05	692.08	31.12	0	0.002	···
ORDER	−345.99	1	693.97	693.98	33.02	0	0.0025	···

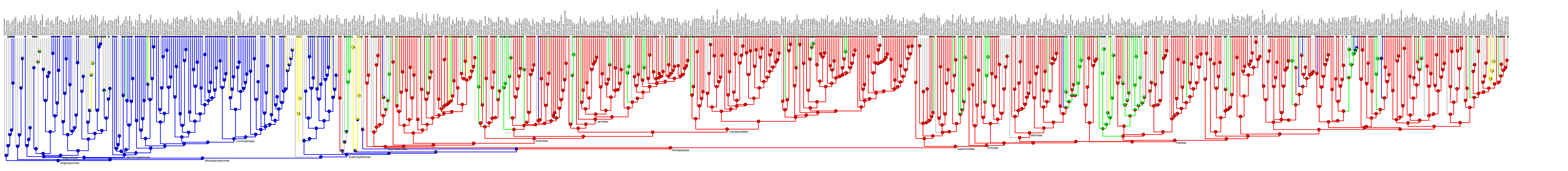

MP ancestral state reconstruction using ancestral.pars
(R:phangorn)
232_B. Perianth merism (3−state) (D2c), 70 steps

- trimerous
- tetramerous
- pentamerous

Node	MP state(s)
Angiospermae	trimerous
Mesangiospermae	trimerous
Magnoliidae	trimerous
Monocotyledonae	trimerous
Eudicotyledonae	trimerous
Commelinidae	trimerous
Pentapetalae	pentamerous
Superasteridae	pentamerous
Asteridae	pentamerous
Lamiidae	pentamerous
Campanulidae	pentamerous
Superrosidae	pentamerous
Rosidae	pentamerous
Malvidae	pentamerous
Fabidae	pentamerous

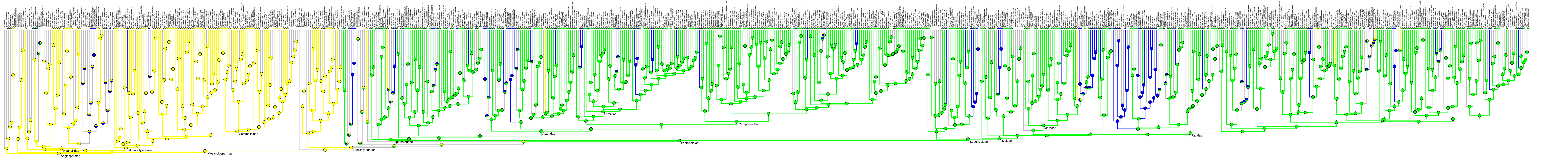

ML ancestral state reconstruction using rayDISC (R:corHMM)
232_B. Perianth merism (3−state) (D2c), SYMeq model

- trimerous
- tetramerous
- pentamerous

Node	ML state	Prob
Angiospermae	trimerous	1
Mesangiospermae	trimerous	1
Magnoliidae	trimerous	1
Monocotyledonae	trimerous	1
Eudicotyledonae	trimerous	0.9794
Commelinidae	trimerous	1
Pentapetalae	pentamerous	0.9998
Superasteridae	pentamerous	1
Asteridae	pentamerous	1
Lamiidae	pentamerous	1
Campanulidae	pentamerous	1
Superrosidae	pentamerous	1
Rosidae	pentamerous	1
Malvidae	pentamerous	1
Fabidae	pentamerous	1

Model	LogL	Npar	AIC	AICc	DeltaAICc	w	q01	···
ARD	−255.99	6	523.99	524.09	6.43	0.02	7e−04	···
ARDeq	−254.93	6	521.86	521.96	4.31	0.05	7e−04	···
ER	−281	1	564.01	564.01	46.35	0	0.0011	···
SYM	−256.91	3	519.82	519.85	2.19	0.15	9e−04	···
SYMeq*	−255.81	3	517.63	517.66	0	0.44	9e−04	···
ORD	−256.76	4	521.51	521.57	3.91	0.06	9e−04	···
ORDeq	−255.84	4	519.68	519.73	2.07	0.16	0.001	···
ORDSYM	−259.56	2	523.12	523.14	5.48	0.03	0.0015	···
ORDSYMeq	−258.49	2	520.98	520.99	3.34	0.08	0.0015	···
ORDER	−261.94	1	525.89	525.89	8.24	0.01	0.0023	···

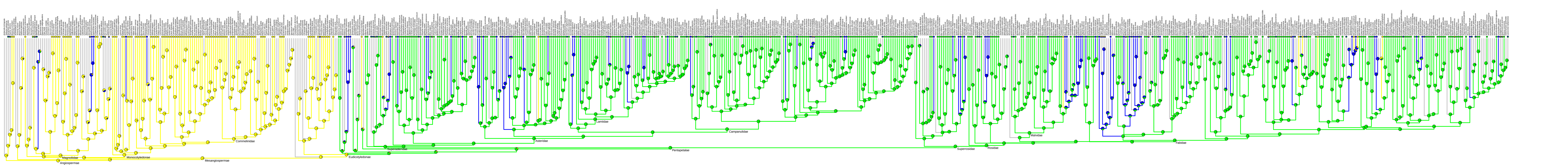

MP ancestral state reconstruction using ancestral.pars
(R:phangorn)
234_A. Perianth differentiation (binary) (D2d), 65 steps

- undifferentiated
- differentiated

Node	MP state(s)
Angiospermae	undifferentiated
Mesangiospermae	undifferentiated
Magnoliidae	undifferentiated / differentiated
Monocotyledonae	undifferentiated
Eudicotyledonae	undifferentiated
Commelinidae	undifferentiated
Pentapetalae	differentiated
Superasteridae	differentiated
Asteridae	differentiated
Lamiidae	differentiated
Campanulidae	differentiated
Superrosidae	differentiated
Rosidae	differentiated
Malvidae	differentiated
Fabidae	differentiated

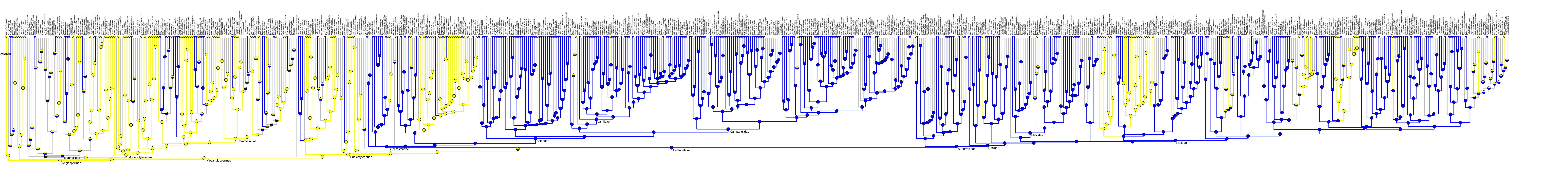

ML ancestral state reconstruction using rayDISC (R:corHMM)
234_A. Perianth differentiation (binary) (D2d), ARDeq model

- undifferentiated
- differentiated

Node	ML state	Prob
Angiospermae	undifferentiated	1
Mesangiospermae	undifferentiated	1
Magnoliidae	undifferentiated	0.9999
Monocotyledonae	undifferentiated	1
Eudicotyledonae	undifferentiated	0.9918
Commelinidae	undifferentiated	0.9999
Pentapetalae	differentiated	0.9471
Superasteridae	differentiated	0.9488
Asteridae	differentiated	0.9985
Lamiidae	differentiated	0.9999
Campanulidae	differentiated	1
Superrosidae	differentiated	0.9908
Rosidae	differentiated	0.9944
Malvidae	differentiated	0.9989
Fabidae	differentiated	0.9883

Model	LogL	Npar	AIC	AICc	DeltaAICc	w	q01	q10
ARD	−199.76	2	403.52	403.54	1.39	0.33	0.0045	0.0015
ARDeq**	−199.07	2	402.14	402.15	0	0.67	0.0045	0.0015
ER	−208.79	1	419.59	419.59	17.44	0	0.0025	0.0025
UNI01	−228.44	1	458.88	458.88	56.73	0	0.0088	
UNI10	−233.4	1	468.79	468.8	66.64	0		0.0034

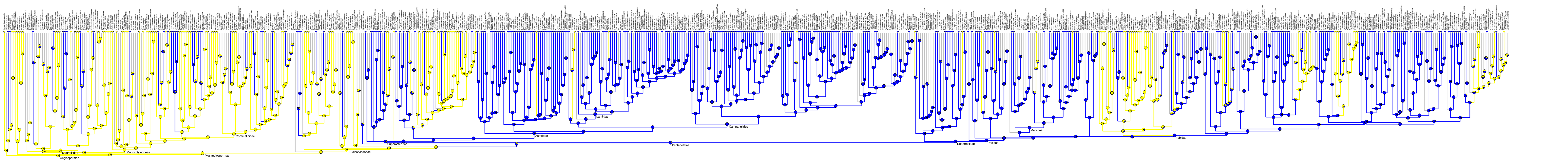

MP ancestral state reconstruction using ancestral.pars
(R:phangorn)
204_A. Fusion of perianth (D2c), 78 steps

free (<5%)
fused (>5%)

Node	MP state(s)
Angiospermae	free (<5%)
Mesangiospermae	free (<5%)
Magnoliidae	free (<5%)
Monocotyledonae	free (<5%)
Eudicotyledonae	free (<5%)
Commelinidae	free (<5%)
Pentapetalae	free (<5%)
Superasteridae	free (<5%)
Asteridae	free (<5%)
Lamiidae	free (<5%) / fused (>5%)
Campanulidae	fused (>5%)
Superrosidae	free (<5%)
Rosidae	free (<5%)
Malvidae	free (<5%)
Fabidae	free (<5%)

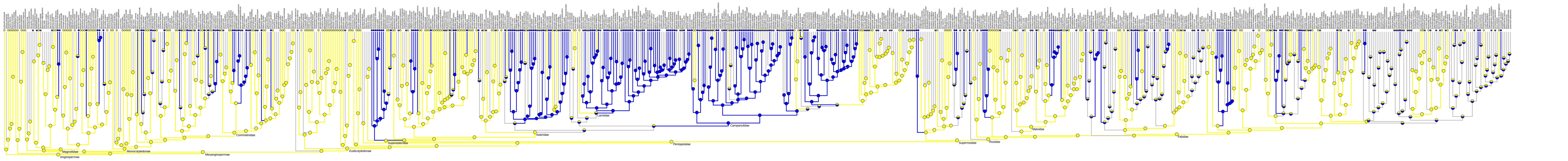

ML ancestral state reconstruction using rayDISC (R:corHMM)
204_A. Fusion of perianth (D2c), ARDeq model

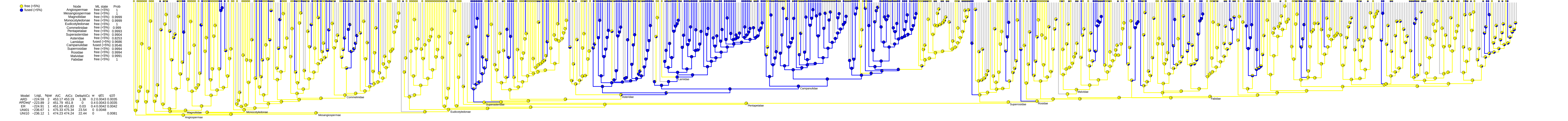

Node	ML state	Prob
Angiospermae	free (<5%)	1
Mesangiospermae	free (<5%)	1
Magnoliidae	free (<5%)	0.9999
Monocotyledonae	free (<5%)	0.9999
Eudicotyledonae	free (<5%)	1
Commelinidae	free (<5%)	0.999
Pentapetalae	free (<5%)	0.9993
Superasteridae	free (<5%)	0.9904
Asteridae	free (<5%)	0.8253
Lamiidae	fused (>5%)	0.9686
Campanulidae	fused (>5%)	0.9546
Superrosidae	free (<5%)	0.9994
Rosidae	free (<5%)	0.9994
Malvidae	free (<5%)	0.9991
Fabidae	free (<5%)	1

Model	LogL	Npar	AIC	AICc	DeltaAICc	w	q01	q10
ARD	−224.59	2	453.17	453.19	1.38	0.2	0.0043	0.0035
ARDeq*	−223.89	2	451.79	451.8	0	0.4	0.0043	0.0035
ER	−224.91	1	451.83	451.83	0.03	0.4	0.0042	0.0042
UNI01	−236.67	1	475.33	475.34	23.54	0	0.0048	
UNI10	−236.12	1	474.23	474.24	22.44	0		0.0081

MP ancestral state reconstruction using ancestral.pars
(R:phangorn)
207_A. Symmetry of perianth (binary) (D2d), 56 steps

- actinomorphic
- zygomorphic

Node	MP state(s)
Angiospermae	actinomorphic
Mesangiospermae	actinomorphic
Magnoliidae	actinomorphic
Monocotyledonae	actinomorphic
Eudicotyledonae	actinomorphic
Commelinidae	actinomorphic
Pentapetalae	actinomorphic
Superasteridae	actinomorphic
Asteridae	actinomorphic
Lamiidae	actinomorphic
Campanulidae	actinomorphic
Superrosidae	actinomorphic
Rosidae	actinomorphic
Malvidae	actinomorphic
Fabidae	actinomorphic

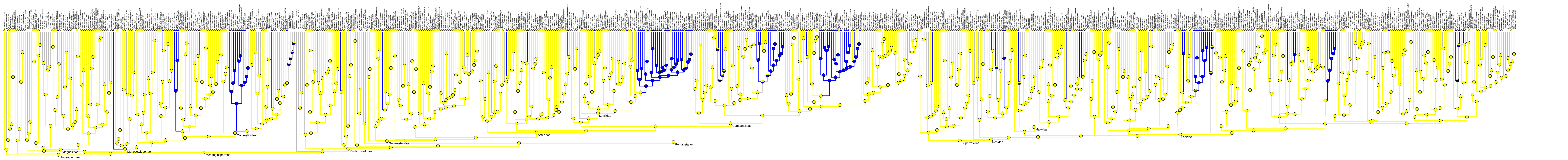

ML ancestral state reconstruction using rayDISC (R:corHMM)
207_A. Symmetry of perianth (binary) (D2d), ARDeq model

- actinomorphic (yellow)
- zygomorphic (blue)

Node	ML state	Prob
Angiospermae	actinomorphic	1
Mesangiospermae	actinomorphic	1
Magnoliidae	actinomorphic	1
Monocotyledonae	actinomorphic	0.9997
Eudicotyledonae	actinomorphic	1
Commelinidae	actinomorphic	0.9974
Pentapetalae	actinomorphic	1
Superasteridae	actinomorphic	1
Asteridae	actinomorphic	1
Lamiidae	actinomorphic	0.996
Campanulidae	actinomorphic	1
Superrosidae	actinomorphic	1
Rosidae	actinomorphic	1
Malvidae	actinomorphic	0.9997
Fabidae	actinomorphic	1

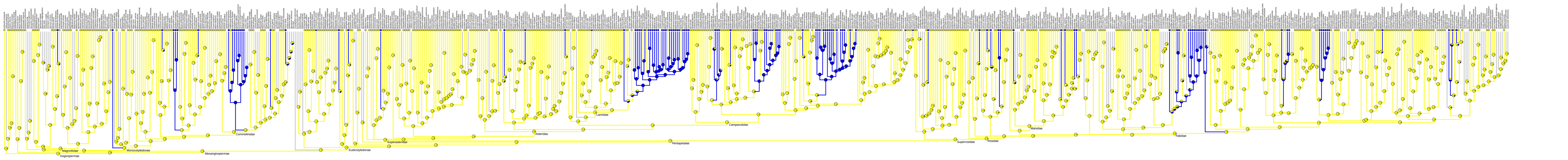

Model	LogL	Npar	AIC	AICc	DeltaAICc	w	q01	q10
ARD	−210.77	2	425.53	425.55	1.38	0.33	0.0014	0.0062
ARDeq**	−210.08	2	424.15	424.17	0	0.67	0.0014	0.0062
ER	−219.85	1	441.71	441.71	17.55	0	0.0015	0.0015
UNI01	−267.95	1	537.91	537.91	113.75	0	0.0019	
UNI10	−217.33	1	436.66	436.67	12.5	0		0.0167

MP ancestral state reconstruction using ancestral.pars
(R:phangorn)
301_B. Number of fertile stamens (3−state) (D2c), 156 steps

- one to five (1−5)
- six to ten (6−10)
- more than ten (>10)

Node	MP state(s)
Angiospermae	more than ten (>10)
Mesangiospermae	one to five (1−5) / more than ten (>10)
Magnoliidae	more than ten (>10)
Monocotyledonae	six to ten (6−10)
Eudicotyledonae	one to five (1−5)
Commelinidae	six to ten (6−10)
Pentapetalae	six to ten (6−10)
Superasteridae	six to ten (6−10)
Asteridae	one to five (1−5) / six to ten (6−10)
Lamiidae	one to five (1−5)
Campanulidae	one to five (1−5)
Superrosidae	six to ten (6−10)
Rosidae	six to ten (6−10)
Malvidae	six to ten (6−10)
Fabidae	six to ten (6−10)

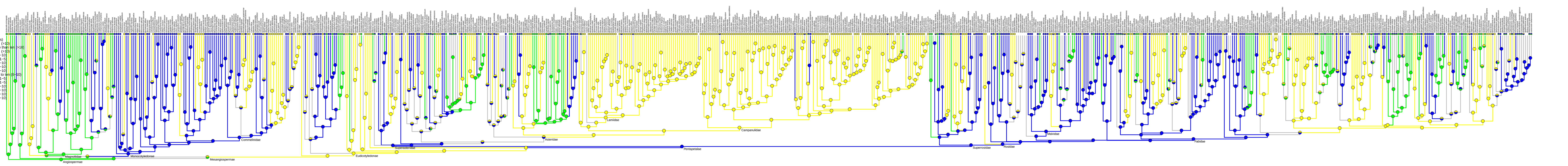

ML ancestral state reconstruction using rayDISC (R:corHMM)
301_B. Number of fertile stamens (3−state) (D2c), ARDeq model

- one to five (1−5)
- six to ten (6−10)
- more than ten (>10)

Node	ML state	Prob
Angiospermae	more than ten (>10)	1
Mesangiospermae	more than ten (>10)	1
Magnoliidae	more than ten (>10)	0.9999
Monocotyledonae	six to ten (6−10)	0.58
Eudicotyledonae	more than ten (>10)	1
Commelinidae	six to ten (6−10)	0.9997
Pentapetalae	more than ten (>10)	1
Superasteridae	more than ten (>10)	1
Asteridae	more than ten (>10)	0.9993
Lamiidae	one to five (1−5)	0.9995
Campanulidae	one to five (1−5)	0.9968
Superrosidae	more than ten (>10)	1
Rosidae	more than ten (>10)	0.9999
Malvidae	more than ten (>10)	0.9154
Fabidae	more than ten (>10)	1

Model	LogL	Npar	AIC	AICc	DeltaAICc	w	q01	···
ARD	−491.73	6	995.45	995.56	2.2	0.25	4e−04	···
ARDeq**	−490.63	6	993.25	993.36	0	0.75	4e−04	···
ER	−536.12	1	1074.24	1074.25	80.89	0	0.0027	···
SYM	−528.65	3	1063.3	1063.33	69.97	0	0.0038	···
SYMeq	−527.8	3	1061.59	1061.62	68.26	0	0.0038	···
ORD	−507.58	4	1023.16	1023.21	29.85	0	8e−04	···
ORDeq	−507	4	1022	1022.05	28.69	0	8e−04	···
ORDSYM	−531.31	2	1066.62	1066.64	73.27	0	0.0042	···
ORDSYMeq	−530.41	2	1064.81	1064.83	71.46	0	0.0042	···
ORDER	−531.35	1	1064.71	1064.71	71.35	0	0.0043	···

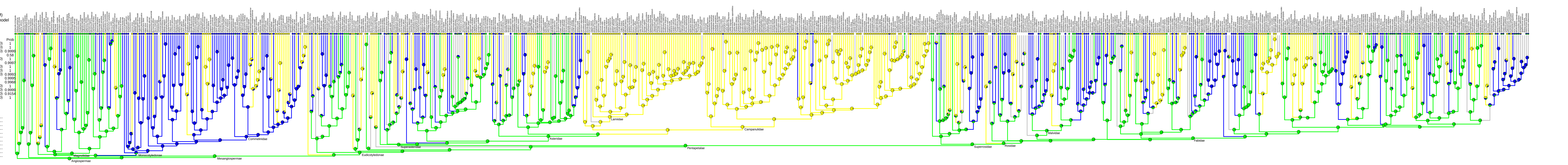

MP ancestral state reconstruction using ancestral.pars
(R:phangorn)
301_C. Number of fertile stamens (binary) (D2c), 91 steps

- one to six (1−6)
- more than six (>6)

Node	MP state(s)
Angiospermae	more than six (>6)
Mesangiospermae	one to six (1−6)
Magnoliidae	one to six (1−6) / more than six (>6)
Monocotyledonae	one to six (1−6)
Eudicotyledonae	one to six (1−6)
Commelinidae	one to six (1−6)
Pentapetalae	more than six (>6)
Superasteridae	more than six (>6)
Asteridae	more than six (>6)
Lamiidae	one to six (1−6)
Campanulidae	one to six (1−6)
Superrosidae	more than six (>6)
Rosidae	more than six (>6)
Malvidae	more than six (>6)
Fabidae	more than six (>6)

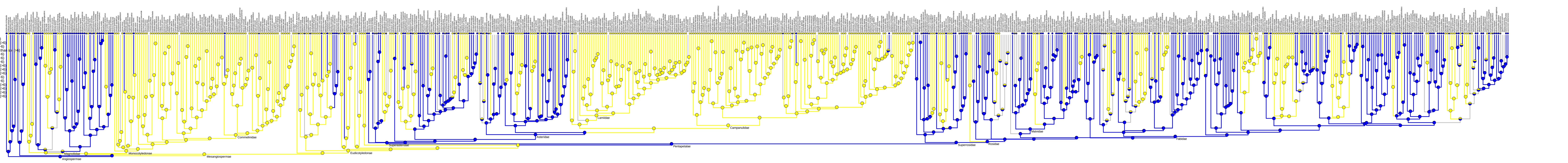

ML ancestral state reconstruction using rayDISC (R:corHMM)
301_C. Number of fertile stamens (binary) (D2c), ARDeq model

- one to six (1−6)
- more than six (>6)

Node	ML state	Prob
Angiospermae	more than six (>6)	1
Mesangiospermae	more than six (>6)	1
Magnoliidae	more than six (>6)	1
Monocotyledonae	one to six (1−6)	0.5065
Eudicotyledonae	more than six (>6)	0.9999
Commelinidae	one to six (1−6)	1
Pentapetalae	more than six (>6)	1
Superasteridae	more than six (>6)	1
Asteridae	more than six (>6)	0.9999
Lamiidae	one to six (1−6)	0.999
Campanulidae	one to six (1−6)	0.9944
Superrosidae	more than six (>6)	1
Rosidae	more than six (>6)	1
Malvidae	more than six (>6)	1
Fabidae	more than six (>6)	1

Model	LogL	Npar	AIC	AICc	DeltaAICc	w	q01	q10
ARD	−288.64	2	581.28	581.3	1.39	0.33	5e−04	0.0058
ARDeq**	−287.95	2	579.9	579.91	0	0.67	5e−04	0.0058
ER	−322.91	1	647.82	647.82	67.91	0	0.0031	0.0031
UNI01	−361.93	1	725.86	725.87	145.96	0	0.0046	
UNI10	−325.72	1	653.45	653.45	73.54	0		0.0073

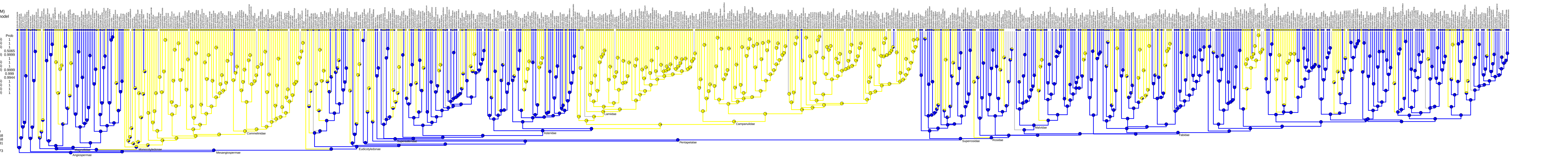

MP ancestral state reconstruction using ancestral.pars
(R:phangorn)
330_A. Androecium structural phyllotaxy (binary) (D2d), 12 steps

whorled
spiral

Node	MP state(s)
Angiospermae	whorled / spiral
Mesangiospermae	whorled
Magnoliidae	whorled
Monocotyledonae	whorled
Eudicotyledonae	whorled
Commelinidae	whorled
Pentapetalae	whorled
Superasteridae	whorled
Asteridae	whorled
Lamiidae	whorled
Campanulidae	whorled
Superrosidae	whorled
Rosidae	whorled
Malvidae	whorled
Fabidae	whorled

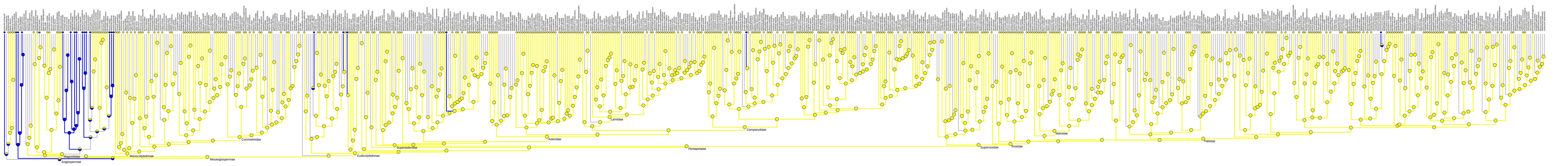

ML ancestral state reconstruction using rayDISC (R:corHMM)
330_A. Androecium structural phyllotaxy (binary) (D2d), ARDeq model

whorled
spiral

Node	ML state	Prob
Angiospermae	spiral	1
Mesangiospermae	spiral	0.999
Magnoliidae	spiral	0.9982
Monocotyledonae	whorled	0.5917
Eudicotyledonae	spiral	0.9786
Commelinidae	whorled	0.9997
Pentapetalae	whorled	0.9979
Superasteridae	whorled	0.9992
Asteridae	whorled	1
Lamiidae	whorled	1
Campanulidae	whorled	1
Superrosidae	whorled	0.9996
Rosidae	whorled	0.9998
Malvidae	whorled	1
Fabidae	whorled	1

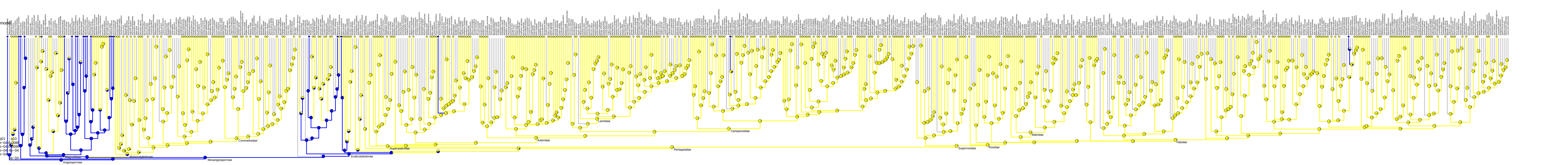

Model	LogL	Npar	AIC	AICc	DeltaAICc	w	q01	q10
ARD	−55.47	2	114.94	114.96	1.39	0.33	1e−04	0.0086
ARDeq**	−54.78	2	113.55	113.57	0	0.66	1e−04	0.0086
ER	−60.92	1	123.84	123.85	10.28	0	4e−04	4e−04
UNI01	−60.72	1	123.45	123.45	9.88	0	4e−04	
UNI10	−1e+06	1	2000002	2000002.01	1999888.43	0		4e−04

MP ancestral state reconstruction using ancestral.pars
(R:phangorn)
331_A. Number of androecium structural whorls (3−state) (D2c), 75 steps

- one (1)
- two (2)
- more than two (>2)

Node	MP state(s)
Angiospermae	more than two (>2)
Mesangiospermae	more than two (>2)
Magnoliidae	more than two (>2)
Monocotyledonae	two (2)
Eudicotyledonae	more than two (>2)
Commelinidae	two (2)
Pentapetalae	one (1)
Superasteridae	one (1)
Asteridae	one (1)
Lamiidae	one (1)
Campanulidae	one (1)
Superrosidae	one (1)
Rosidae	one (1)
Malvidae	one (1) / two (2)
Fabidae	one (1)

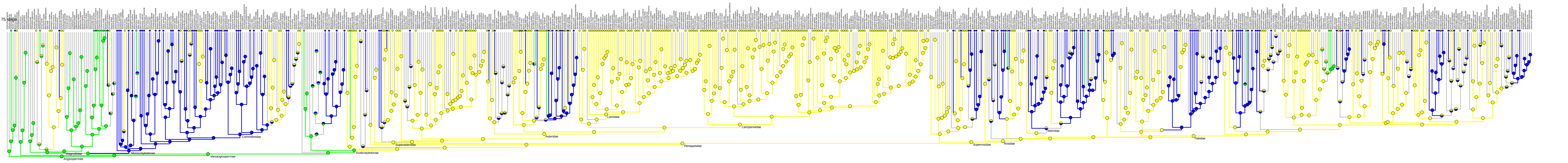

ML ancestral state reconstruction using rayDISC (R:corHMM)
331_A. Number of androecium structural whorls (3−state) (D2c), ARD model

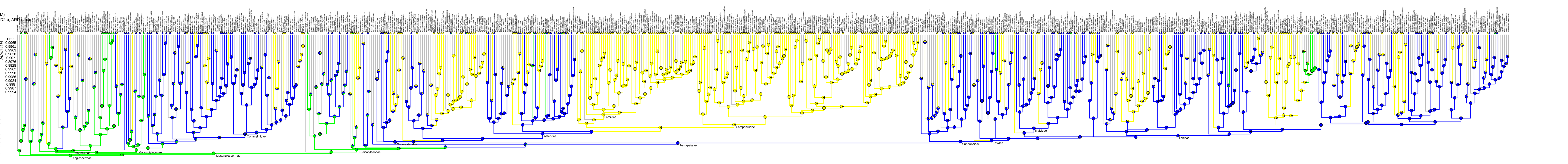

Node	ML state	Prob
Angiospermae	more than two (>2)	0.9965
Mesangiospermae	more than two (>2)	0.9961
Magnoliidae	more than two (>2)	0.9963
Monocotyledonae	more than two (>2)	0.9638
Eudicotyledonae	more than two (>2)	0.907
Commelinidae	two (2)	0.8976
Pentapetalae	two (2)	0.9928
Superasteridae	two (2)	0.9962
Asteridae	two (2)	0.9998
Lamiidae	one (1)	0.9986
Campanulidae	one (1)	0.9924
Superrosidae	two (2)	0.998
Rosidae	two (2)	0.9987
Malvidae	two (2)	0.9994
Fabidae	two (2)	1

Model	LogL	Npar	AIC	AICc	DeltaAICc	w	q01	···
ARD*	−231.02	6	474.03	474.14	0	0.39	0	···
ARDeq	−231.52	6	475.04	475.15	1.01	0.24	0	···
ER	−280.88	1	563.75	563.76	89.62	0	0.0017	···
SYM	−254.86	3	515.72	515.75	41.61	0	0.0035	···
SYMeq	−253.8	3	513.61	513.64	39.5	0	0.0035	···
ORD	−234.46	4	476.91	476.96	2.82	0.1	0	···
ORDeq	−233.41	4	474.81	474.86	0.72	0.27	0	···
ORDSYM	−255.25	2	514.49	514.51	40.37	0	0.0036	···
ORDSYMeq	−254.17	2	512.35	512.36	38.22	0	0.0036	···
ORDER	−260.53	1	523.05	523.06	48.92	0	0.0028	···

MP ancestral state reconstruction using ancestral.pars
(R:phangorn)
332_A. Androecium structural merism (4−state) (D2c), 56 steps

- dimerous
- trimerous
- tetramerous
- pentamerous

Node	MP state(s)
Angiospermae	trimerous / pentamerous
Mesangiospermae	trimerous / pentamerous
Magnoliidae	trimerous
Monocotyledonae	trimerous
Eudicotyledonae	trimerous / pentamerous
Commelinidae	trimerous
Pentapetalae	pentamerous
Superasteridae	pentamerous
Asteridae	pentamerous
Lamiidae	pentamerous
Campanulidae	pentamerous
Superrosidae	pentamerous
Rosidae	pentamerous
Malvidae	pentamerous
Fabidae	pentamerous

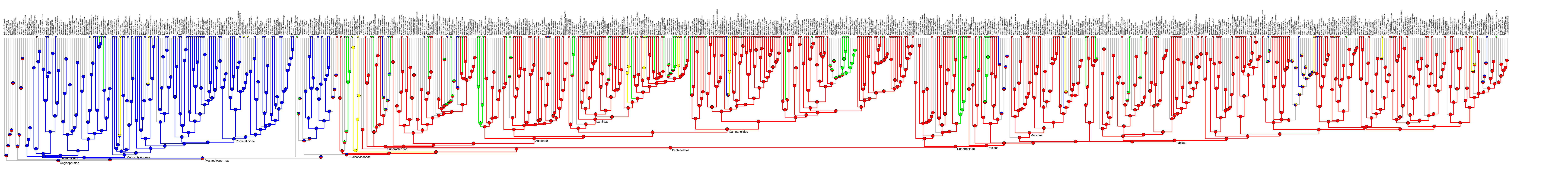

ML ancestral state reconstruction using rayDISC (R:corHMM)
332_A. Androecium structural merism (4−state) (D2c), SYMeq model

- dimerous (yellow)
- trimerous (blue)
- tetramerous (green)
- pentamerous (red)

Node	ML state	Prob
Angiospermae	trimerous	1
Mesangiospermae	trimerous	0.9993
Magnoliidae	trimerous	1
Monocotyledonae	trimerous	1
Eudicotyledonae	trimerous	0.8545
Commelinidae	trimerous	1
Pentapetalae	pentamerous	1
Superasteridae	pentamerous	1
Asteridae	pentamerous	0.9996
Lamiidae	pentamerous	1
Campanulidae	pentamerous	1
Superrosidae	pentamerous	1
Rosidae	pentamerous	1
Malvidae	pentamerous	1
Fabidae	pentamerous	1

Model	LogL	Npar	AIC	AICc	DeltaAICc	w	q01	···
ARD	−223.12	12	470.25	470.65	10.88	0	0.0072	···
ARDeq	−221.81	12	467.61	468.01	8.25	0.01	0.0071	···
ER	−237.53	1	477.06	477.06	17.3	0	9e−04	···
SYM	−225.16	6	462.31	462.42	2.65	0.21	6e−04	···
SYMeq**	−223.83	6	459.66	459.77	0	0.78	6e−04	···
ORD	−237.28	6	486.56	486.67	26.9	0	100	···
ORDeq	−235.92	6	483.84	483.94	24.18	0	100	···
ORDSYM	−259.11	3	524.22	524.25	64.49	0	0.0021	···
ORDSYMeq	−257.87	3	521.75	521.78	62.01	0	0.0021	···
ORDER	−259.98	1	521.96	521.96	62.2	0	0.0031	···

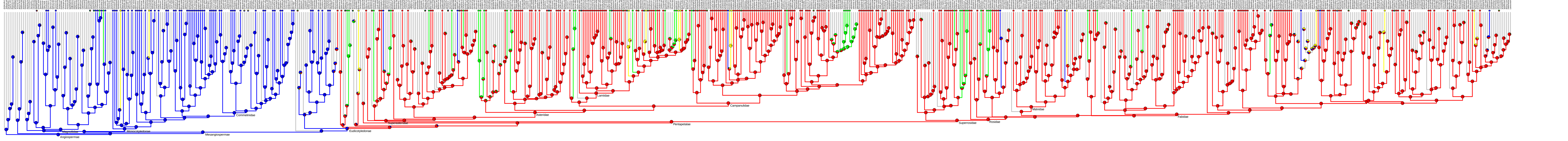

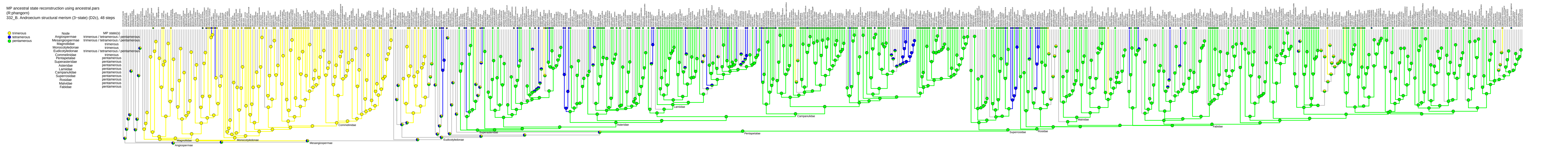
MP ancestral state reconstruction using ancestral.pars
(R:phangorn)
332_B. Androecium structural merism (3−state) (D2c), 48 steps
trimerous
tetramerous
pentamerous
Node
MP state(s)
Angiospermae trimerous / tetramerous / pentamerous
Mesangiospermae trimerous / tetramerous / pentamerous
Magnoliidae trimerous
Monocotyledonae trimerous
Eudicotyledonae trimerous / tetramerous / pentamerous
Commelinidae trimerous
Pentapetalae pentamerous
Superasteridae pentamerous
Asteridae pentamerous
Lamiidae pentamerous
Campanulidae pentamerous
Superrosidae pentamerous
Rosidae pentamerous
Malvidae pentamerous
Fabidae pentamerous
Angiospermae
Magnoliidae
Monocotyledonae
Commelinidae
Mesangiospermae
Eudicotyledonae
Superasteridae
Asteridae
Lamiidae
Campanulidae
Pentapetalae
Superrosidae
Rosidae
Malvidae
Fabidae

ML ancestral state reconstruction using rayDISC (R:corHMM)
332_B. Androecium structural merism (3−state) (D2c), SYMeq model

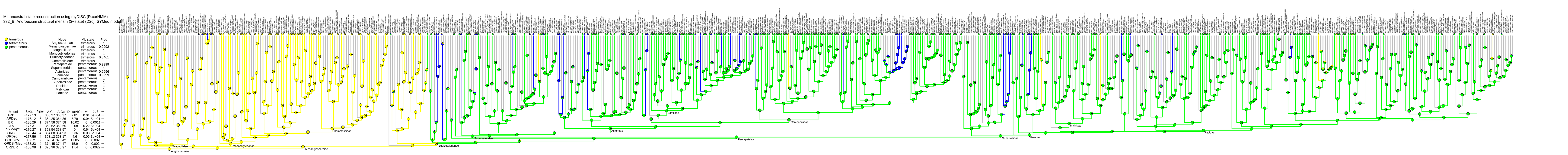

Node	ML state	Prob
Angiospermae	trimerous	1
Mesangiospermae	trimerous	0.9992
Magnoliidae	trimerous	1
Monocotyledonae	trimerous	1
Eudicotyledonae	trimerous	0.8461
Commelinidae	trimerous	1
Pentapetalae	pentamerous	0.9999
Superasteridae	pentamerous	1
Asteridae	pentamerous	0.9996
Lamiidae	pentamerous	0.9999
Campanulidae	pentamerous	1
Superrosidae	pentamerous	1
Rosidae	pentamerous	1
Malvidae	pentamerous	1
Fabidae	pentamerous	1

Model	LogL	Npar	AIC	AICc	DeltaAICc	w	q01	···
ARD	−177.13	6	366.27	366.37	7.81	0.01	5e−04	···
ARDeq	−176.12	6	364.25	364.36	5.79	0.04	5e−04	···
ER	−186.29	1	374.58	374.58	16.02	0	0.0011	···
SYM	−177.31	3	360.62	360.65	2.08	0.22	5e−04	···
SYMeq**	−176.27	3	358.54	358.57	0	0.64	5e−04	···
ORD	−178.44	4	364.88	364.93	6.36	0.03	5e−04	···
ORDeq	−177.56	4	363.12	363.17	4.6	0.06	3e−04	···
ORDSYM	−186.2	2	376.4	376.42	17.85	0	0.002	···
ORDSYMeq	−185.23	2	374.45	374.47	15.9	0	0.002	···
ORDER	−186.98	1	375.96	375.97	17.4	0	0.0027	···

MP ancestral state reconstruction using ancestral.pars
(R:phangorn)
305_A. Filament (binary) (D2d), 53 steps

- laminar (wide)
- typical (narrow)

Node	MP state(s)
Angiospermae	laminar (wide) / typical (narrow)
Mesangiospermae	laminar (wide) / typical (narrow)
Magnoliidae	laminar (wide)
Monocotyledonae	typical (narrow)
Eudicotyledonae	typical (narrow)
Commelinidae	laminar (wide) / typical (narrow)
Pentapetalae	typical (narrow)
Superasteridae	typical (narrow)
Asteridae	typical (narrow)
Lamiidae	typical (narrow)
Campanulidae	typical (narrow)
Superrosidae	typical (narrow)
Rosidae	typical (narrow)
Malvidae	typical (narrow)
Fabidae	typical (narrow)

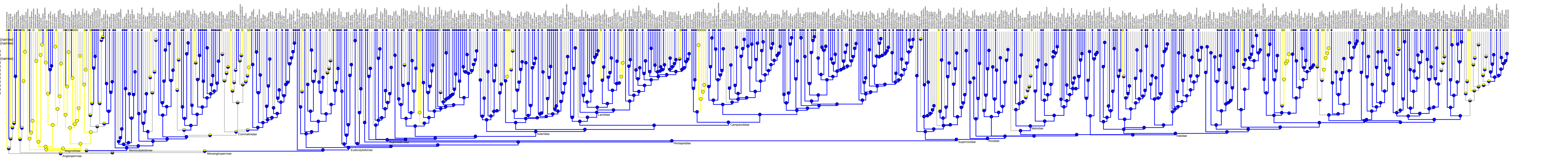

ML ancestral state reconstruction using rayDISC (R:corHMM)
305_A. Filament (binary) (D2d), ARDeq model

- laminar (wide)
- typical (narrow)

Node	ML state	Prob
Angiospermae	laminar (wide)	1
Mesangiospermae	laminar (wide)	0.9923
Magnoliidae	laminar (wide)	0.9985
Monocotyledonae	laminar (wide)	0.8479
Eudicotyledonae	typical (narrow)	0.6209
Commelinidae	laminar (wide)	0.6586
Pentapetalae	typical (narrow)	0.9989
Superasteridae	typical (narrow)	0.9994
Asteridae	typical (narrow)	0.9996
Lamiidae	typical (narrow)	0.9973
Campanulidae	typical (narrow)	0.9954
Superrosidae	typical (narrow)	0.9994
Rosidae	typical (narrow)	0.9996
Malvidae	typical (narrow)	0.9973
Fabidae	typical (narrow)	1

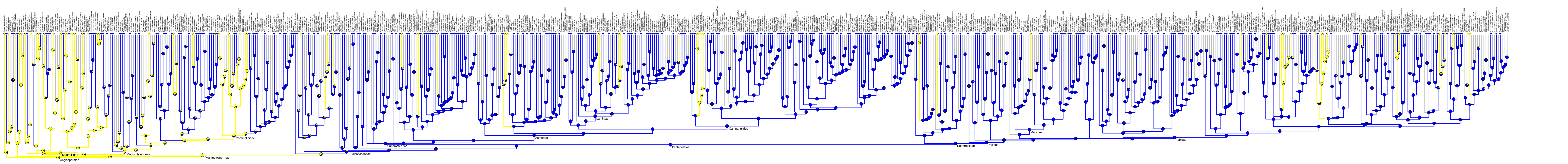

Model	LogL	Npar	AIC	AICc	DeltaAICc	w	q01	q10
ARD	−172.3	2	348.59	348.61	1.28	0.34	0.0088	0.0021
ARDeq**	−171.66	2	347.31	347.33	0	0.65	0.009	0.0021
ER	−177.98	1	357.95	357.96	10.63	0	0.0024	0.0024
UNI01	−183.97	1	369.94	369.95	22.62	0	0.0146	
UNI10	−187.51	1	377.02	377.03	29.7	0		0.0023

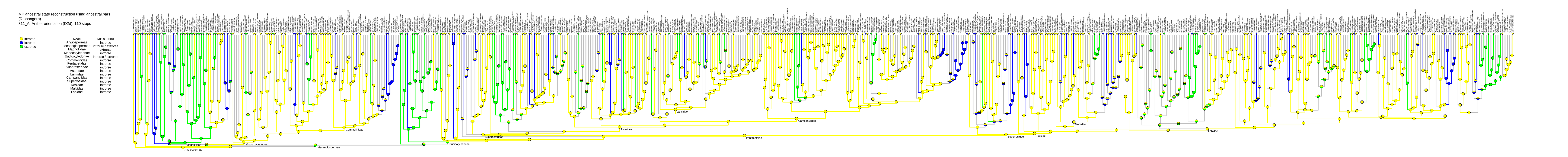
MP ancestral state reconstruction using ancestral.pars
(R:phangorn)
311_A. Anther orientation (D2d), 110 steps
introrse
latrorse
extrorse
Node
Angiospermae
Mesangiospermae
Magnoliidae
Monocotyledonae
Eudicotyledonae
Commelinidae
Pentapetalae
Superasteridae
Asteridae
Lamiidae
Campanulidae
Superrosidae
Rosidae
Malvidae
Fabidae
MP state(s)
introrse
introrse / extrorse
extrorse
introrse
introrse / extrorse
introrse
introrse
introrse
introrse
introrse
introrse
introrse
introrse
introrse
introrse

ML ancestral state reconstruction using rayDISC (R:corHMM)
311_A. Anther orientation (D2d), SYMeq model

- introrse
- latrorse
- extrorse

Node	ML state	Prob
Angiospermae	introrse	1
Mesangiospermae	introrse	0.9984
Magnoliidae	extrorse	0.6989
Monocotyledonae	introrse	0.9976
Eudicotyledonae	introrse	0.997
Commelinidae	introrse	0.9959
Pentapetalae	introrse	1
Superasteridae	introrse	1
Asteridae	introrse	0.9996
Lamiidae	introrse	0.9998
Campanulidae	introrse	1
Superrosidae	introrse	1
Rosidae	introrse	1
Malvidae	introrse	0.9998
Fabidae	introrse	0.9998

Model	LogL	Npar	AIC	AICc	DeltaAICc	w	q01	···
ARD	−345.14	6	702.29	702.4	3.74	0.06	0.0022	···
ARDeq	−344.04	6	700.08	700.19	1.54	0.18	0.0037	···
ER	−348.75	1	699.49	699.5	0.85	0.25	0.0027	···
SYM	−347.37	3	700.74	700.77	2.12	0.13	0.0031	···
SYMeq*	−346.31	3	698.62	698.65	0	0.38	0.0031	···

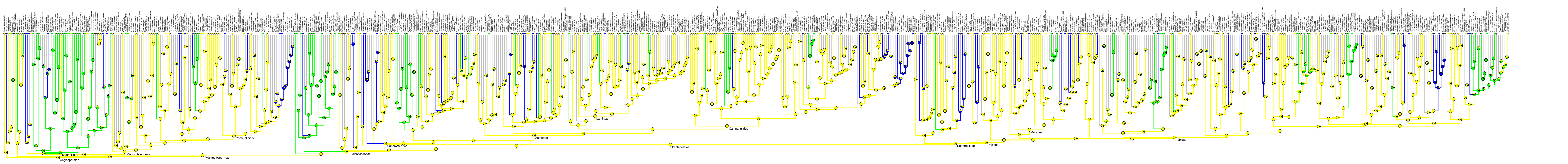

MP ancestral state reconstruction using ancestral.pars
(R:phangorn)
312_A. Anther attachment (binary) (D2d), 98 steps

- basifixed
- dorsifixed

Node	MP state(s)
Angiospermae	basifixed
Mesangiospermae	basifixed
Magnoliidae	basifixed
Monocotyledonae	basifixed
Eudicotyledonae	basifixed
Commelinidae	basifixed
Pentapetalae	basifixed
Superasteridae	basifixed
Asteridae	basifixed
Lamiidae	basifixed
Campanulidae	basifixed / dorsifixed
Superrosidae	basifixed
Rosidae	dorsifixed
Malvidae	dorsifixed
Fabidae	dorsifixed

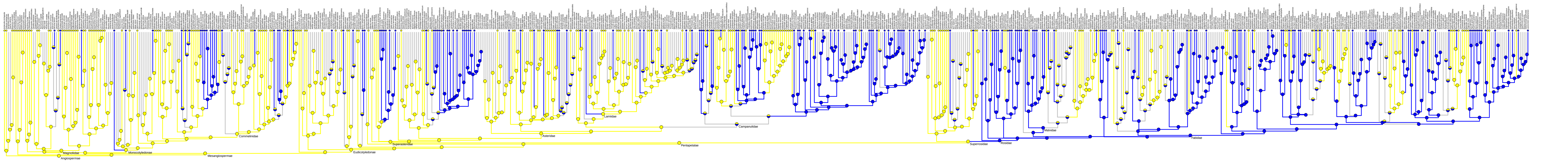

ML ancestral state reconstruction using rayDISC (R:corHMM)
312_A. Anther attachment (binary) (D2d), ARDeq model

- basifixed
- dorsifixed

Node	ML state	Prob
Angiospermae	basifixed	1
Mesangiospermae	basifixed	0.9986
Magnoliidae	basifixed	0.999
Monocotyledonae	basifixed	0.9939
Eudicotyledonae	basifixed	0.8331
Commelinidae	basifixed	0.9764
Pentapetalae	dorsifixed	0.8981
Superasteridae	dorsifixed	0.9059
Asteridae	dorsifixed	0.9031
Lamiidae	dorsifixed	0.7032
Campanulidae	dorsifixed	0.9752
Superrosidae	dorsifixed	0.907
Rosidae	dorsifixed	0.9456
Malvidae	dorsifixed	0.9776
Fabidae	dorsifixed	0.9997

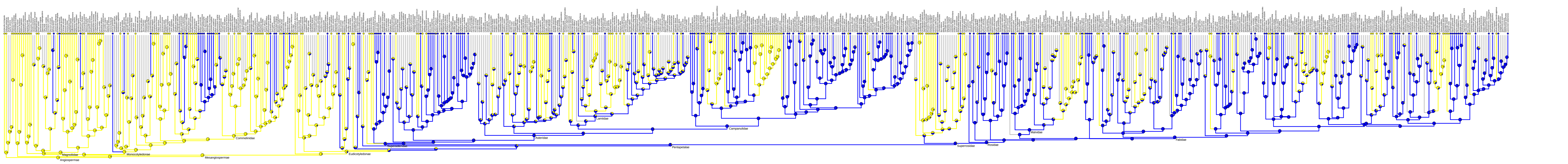

Model	LogL	Npar	AIC	AICc	DeltaAICc	w	q01	q10
ARD	−266.33	2	536.66	536.67	1.31	0.29	0.0043	0.0076
ARDeq**	−265.67	2	535.35	535.36	0	0.55	0.0043	0.0076
ER	−267.94	1	537.87	537.88	2.51	0.16	0.0056	0.0056
UNI01	−293.46	1	588.93	588.93	53.57	0	0.0061	
UNI10	−276.74	1	555.49	555.49	20.13	0		0.008

MP ancestral state reconstruction using ancestral.pars
(R:phangorn)
313_A. Anther dehiscence (3−state) (D2d), 10 steps

- longitudinal slit
- H−valvate
- flap−valvate

Node	MP state(s)
Angiospermae	longitudinal slit
Mesangiospermae	longitudinal slit
Magnoliidae	longitudinal slit
Monocotyledonae	longitudinal slit
Eudicotyledonae	longitudinal slit
Commelinidae	longitudinal slit
Pentapetalae	longitudinal slit
Superasteridae	longitudinal slit
Asteridae	longitudinal slit
Lamiidae	longitudinal slit
Campanulidae	longitudinal slit
Superrosidae	longitudinal slit
Rosidae	longitudinal slit
Malvidae	longitudinal slit
Fabidae	longitudinal slit

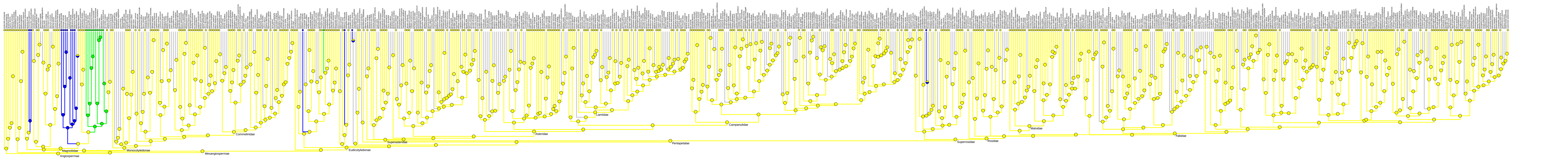

ML ancestral state reconstruction using rayDISC (R:corHMM)
313_A. Anther dehiscence (3−state) (D2d), ARDeq model

- longitudinal slit (yellow)
- H−valvate (blue)
- flap−valvate (green)

Node	ML state	Prob
Angiospermae	longitudinal slit	1
Mesangiospermae	longitudinal slit	1
Magnoliidae	longitudinal slit	0.998
Monocotyledonae	longitudinal slit	1
Eudicotyledonae	longitudinal slit	1
Commelinidae	longitudinal slit	1
Pentapetalae	longitudinal slit	1
Superasteridae	longitudinal slit	1
Asteridae	longitudinal slit	1
Lamiidae	longitudinal slit	1
Campanulidae	longitudinal slit	1
Superrosidae	longitudinal slit	1
Rosidae	longitudinal slit	1
Malvidae	longitudinal slit	1
Fabidae	longitudinal slit	1

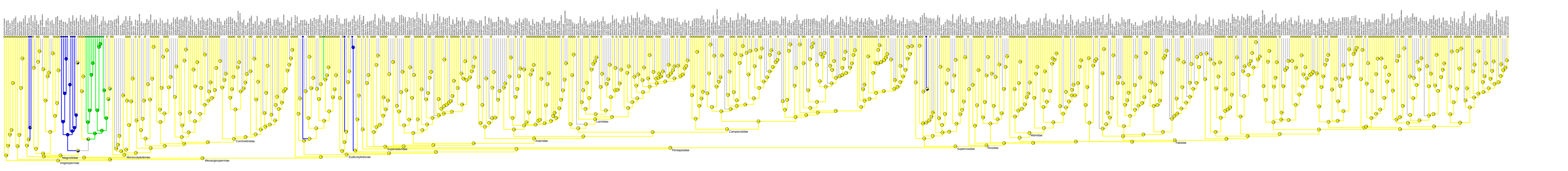

Model	LogL	Npar	AIC	AICc	DeltaAICc	w	q01	···
ARD	−56.75	6	125.49	125.6	2.2	0.12	2e−04	···
ARDeq*	−55.65	6	123.29	123.4	0	0.36	2e−04	···
ER	−61.26	1	124.52	124.52	1.12	0.21	2e−04	···
SYM	−60.23	3	126.46	126.49	3.09	0.08	2e−04	···
SYMeq	−59.13	3	124.26	124.29	0.89	0.23	2e−04	···

MP ancestral state reconstruction using ancestral.pars (R:phangorn)

401_B. Number of structural carpels (5−state) (D2c), 187 steps

- one (1)
- two (2)
- three (3)
- four or five (4−5)
- more than five (>5)

Node	MP state(s)
Angiospermae	more than five (>5)
Mesangiospermae	more than five (>5)
Magnoliidae	more than five (>5)
Monocotyledonae	three (3)
Eudicotyledonae	more than five (>5)
Commelinidae	three (3)
Pentapetalae	three (3) / four or five (4−5)
Superasteridae	three (3) / four or five (4−5)
Asteridae	two (2) / four or five (4−5)
Lamiidae	two (2)
Campanulidae	two (2)
Superrosidae	three (3) / four or five (4−5)
Rosidae	three (3) / four or five (4−5)
Malvidae	four or five (4−5)
Fabidae	four or five (4−5)

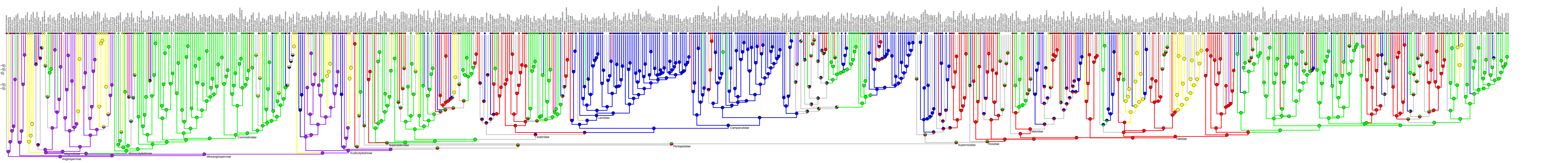

ML ancestral state reconstruction using rayDISC (R:corHMM)
401_B. Number of structural carpels (5−state) (D2c), ARDeq model

- one (1)
- two (2)
- three (3)
- four or five (4−5)
- more than five (>5)

Node	ML state	Prob
Angiospermae	more than five (>5)	1
Mesangiospermae	more than five (>5)	1
Magnoliidae	more than five (>5)	1
Monocotyledonae	more than five (>5)	0.8475
Eudicotyledonae	more than five (>5)	0.9962
Commelinidae	three (3)	0.9999
Pentapetalae	four or five (4−5)	0.9998
Superasteridae	four or five (4−5)	0.9999
Asteridae	four or five (4−5)	0.9957
Lamiidae	two (2)	0.9966
Campanulidae	two (2)	0.9374
Superrosidae	four or five (4−5)	0.9999
Rosidae	four or five (4−5)	0.9998
Malvidae	four or five (4−5)	0.9988
Fabidae	four or five (4−5)	1

Model	LogL	Npar	AIC	AICc	DeltaAICc	w	q01	···
ARD	−663.57	20	1367.15	1368.24	3.22	0.17	0.0017	···
ARDeq**	−661.96	20	1363.93	1365.02	0	0.83	0.0017	···
ER	−717.57	1	1437.13	1437.14	72.12	0	0.0016	···
SYM	−684.18	10	1388.36	1388.64	23.62	0	9e−04	···
SYMeq	−682.63	10	1385.27	1385.55	20.53	0	9e−04	···
ORD	−754.3	8	1524.61	1524.79	159.77	0	0	···
ORDeq	−752.77	8	1521.54	1521.73	156.71	0	0	···
ORDSYM	−760.49	4	1528.97	1529.03	164.01	0	0.0042	···
ORDSYMeq	−759.02	4	1526.04	1526.09	161.07	0	0.0042	···
ORDER	−765.92	1	1533.83	1533.84	168.82	0	0.0057	···

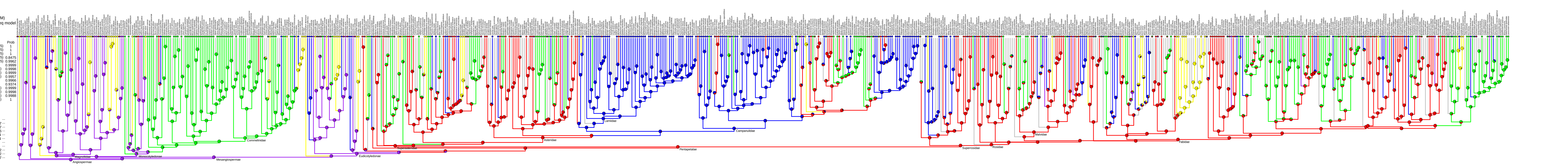

MP ancestral state reconstruction using ancestral.pars
(R:phangorn)
400_A. Gynoecium phyllotaxy (D2d), 10 steps

- whorled
- spiral

Node	MP state(s)
Angiospermae	whorled / spiral
Mesangiospermae	whorled
Magnoliidae	whorled
Monocotyledonae	whorled
Eudicotyledonae	whorled
Commelinidae	whorled
Pentapetalae	whorled
Superasteridae	whorled
Asteridae	whorled
Lamiidae	whorled
Campanulidae	whorled
Superrosidae	whorled
Rosidae	whorled
Malvidae	whorled
Fabidae	whorled

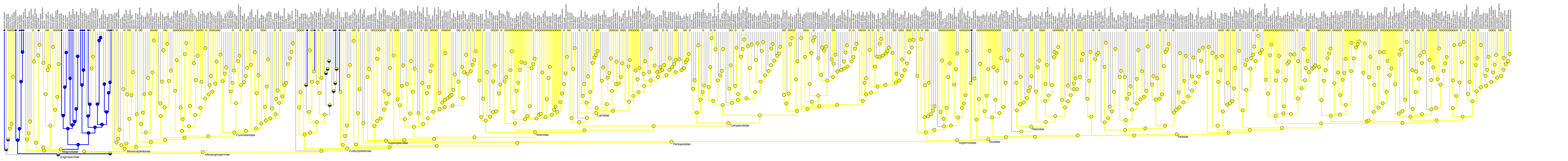

ML ancestral state reconstruction using rayDISC (R:corHMM)
400_A. Gynoecium phyllotaxy (D2d), ARDeq model

- whorled
- spiral

Node	ML state	Prob
Angiospermae	spiral	1
Mesangiospermae	spiral	1
Magnoliidae	spiral	0.9998
Monocotyledonae	whorled	0.5472
Eudicotyledonae	spiral	0.9997
Commelinidae	whorled	0.9997
Pentapetalae	whorled	0.9777
Superasteridae	whorled	0.9928
Asteridae	whorled	1
Lamiidae	whorled	1
Campanulidae	whorled	1
Superrosidae	whorled	0.9802
Rosidae	whorled	0.9804
Malvidae	whorled	1
Fabidae	whorled	1

Model	LogL	Npar	AIC	AICc	DeltaAICc	w	q01	q10
ARD	−39.69	2	83.37	83.39	1.39	0.31	1e−04	0.0082
ARDeq**	−38.99	2	81.98	82	0	0.63	1e−04	0.0082
ER	−51.09	1	104.18	104.19	22.19	0	4e−04	4e−04
UNI01	−52.93	1	107.85	107.86	25.86	0	4e−04	
UNI10	−42.4	1	86.8	86.81	4.81	0.06		0.0096

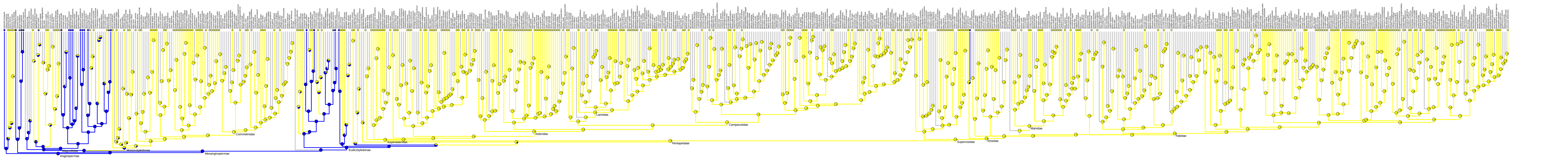

MP ancestral state reconstruction using ancestral.pars
(R:phangorn)
403_A. Fusion of ovaries (binary) (D2c), 24 steps

- free (<5%)
- fused (>5%)

Node	MP state(s)
Angiospermae	free (<5%) / fused (>5%)
Mesangiospermae	fused (>5%)
Magnoliidae	fused (>5%)
Monocotyledonae	fused (>5%)
Eudicotyledonae	fused (>5%)
Commelinidae	fused (>5%)
Pentapetalae	fused (>5%)
Superasteridae	fused (>5%)
Asteridae	fused (>5%)
Lamiidae	fused (>5%)
Campanulidae	fused (>5%)
Superrosidae	fused (>5%)
Rosidae	fused (>5%)
Malvidae	fused (>5%)
Fabidae	fused (>5%)

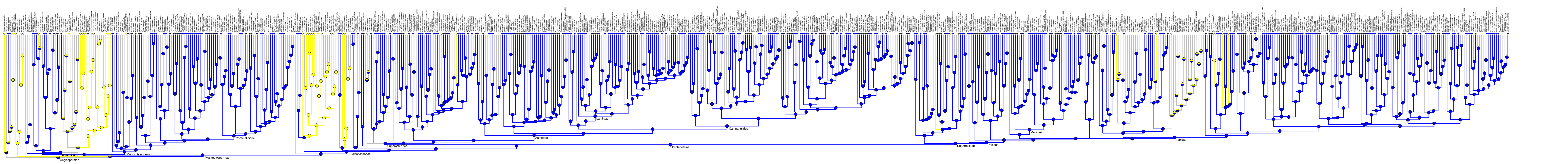

ML ancestral state reconstruction using rayDISC (R:corHMM)
403_A. Fusion of ovaries (binary) (D2c), ARD model

free (<5%)
fused (>5%)

Node	ML state	Prob
Angiospermae	free (<5%)	1
Mesangiospermae	free (<5%)	1
Magnoliidae	free (<5%)	0.9999
Monocotyledonae	free (<5%)	0.9297
Eudicotyledonae	free (<5%)	0.9991
Commelinidae	fused (>5%)	0.9999
Pentapetalae	fused (>5%)	0.9976
Superasteridae	fused (>5%)	0.9979
Asteridae	fused (>5%)	1
Lamiidae	fused (>5%)	1
Campanulidae	fused (>5%)	1
Superrosidae	fused (>5%)	0.9997
Rosidae	fused (>5%)	0.9999
Malvidae	fused (>5%)	1
Fabidae	fused (>5%)	0.9998

Model	LogL	Npar	AIC	AICc	DeltaAICc	w	q01	q10
ARD**	−95.2	2	194.39	194.41	0	0.91	0.0052	5e−04
ARDeq	−102.35	2	208.71	208.72	14.31	0	7e−04	8e−04
ER	−102.85	1	207.7	207.7	13.3	0	8e−04	8e−04
UNI01	−98.54	1	199.08	199.08	4.67	0.09	0.0111	
UNI10	−107.17	1	216.35	216.35	21.94	0		9e−04

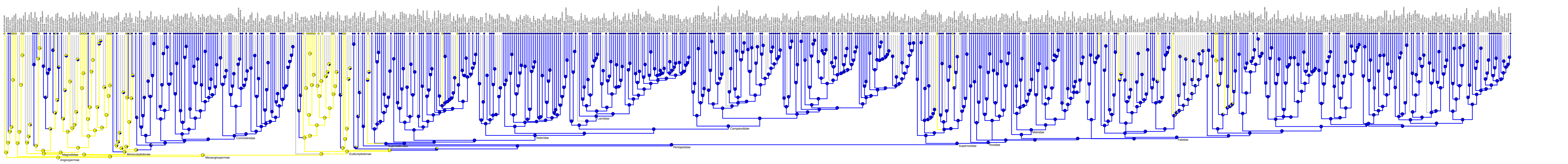

MP ancestral state reconstruction using ancestral.pars
(R:phangorn)
411_A. Number of ovules per functional carpel (3−state) (D2c), 120 steps

- one (1)
- two (2)
- three or more (>2)

Node	MP state(s)
Angiospermae	one (1)
Mesangiospermae	one (1)
Magnoliidae	one (1)
Monocotyledonae	one (1) / three or more (>2)
Eudicotyledonae	three or more (>2)
Commelinidae	one (1)
Pentapetalae	three or more (>2)
Superasteridae	three or more (>2)
Asteridae	one (1)
Lamiidae	one (1) / two (2) / three or more (>2)
Campanulidae	one (1)
Superrosidae	two (2) / three or more (>2)
Rosidae	two (2)
Malvidae	two (2)
Fabidae	two (2)

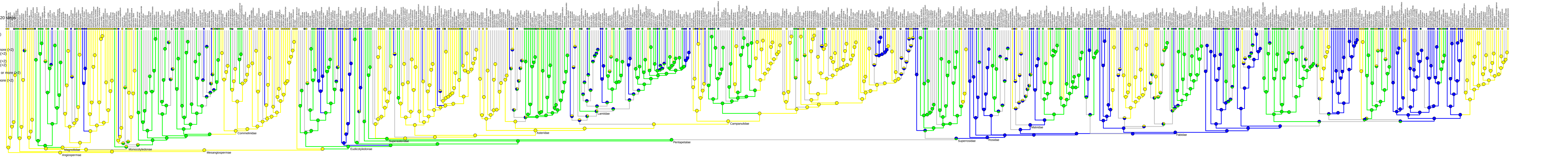

ML ancestral state reconstruction using rayDISC (R:corHMM)
411_A. Number of ovules per functional carpel (3−state) (D2c), ARDeq model

- one (1)
- two (2)
- three or more (>2)

Node	ML state	Prob
Angiospermae	three or more (>2)	1
Mesangiospermae	three or more (>2)	1
Magnoliidae	three or more (>2)	0.9998
Monocotyledonae	three or more (>2)	1
Eudicotyledonae	three or more (>2)	1
Commelinidae	three or more (>2)	0.9041
Pentapetalae	three or more (>2)	1
Superasteridae	three or more (>2)	0.9998
Asteridae	three or more (>2)	0.9972
Lamiidae	three or more (>2)	1
Campanulidae	three or more (>2)	0.9986
Superrosidae	three or more (>2)	1
Rosidae	three or more (>2)	1
Malvidae	three or more (>2)	0.9996
Fabidae	three or more (>2)	1

Model	LogL	Npar	AIC	AICc	DeltaAICc	w	q01	···
ARD	−386.85	6	785.7	785.8	2.19	0.25	0.0013	···
ARDeq**	−385.75	6	783.51	783.61	0	0.75	0.0013	···
ER	−402.49	1	806.98	806.99	23.37	0	0.0028	···
SYM	−401.96	3	809.92	809.95	26.34	0	0.0025	···
SYMeq	−400.99	3	807.98	808.02	24.4	0	0.0025	···
ORD	−398.93	4	805.86	805.91	22.3	0	5e−04	···
ORDeq	−397.84	4	803.67	803.72	20.11	0	5e−04	···
ORDSYM	−412.5	2	828.99	829.01	45.39	0	0.0054	···
ORDSYMeq	−411.57	2	827.14	827.15	43.54	0	0.0054	···
ORDER	−413.51	1	829.02	829.03	45.41	0	0.0062	···

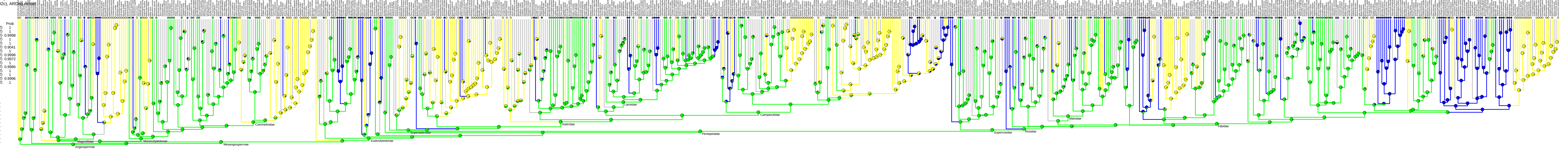

Supplement: Supplementary Data 15 [file ncomms16047-s16.pdf]
