# Supplementary material for: The ancestral flower of angiosperms and its early diversification
Source: Nat Commun. 2017 Aug 1;8:16047. doi: 10.1038/ncomms16047 (PMC5543309; doi:10.1038/ncomms16047)
Supplement: Supplementary Data 16 [file ncomms16047-s17.pdf]

MP ancestral state reconstruction using ancestral.pars
(R:phangorn)
100_A. Functional sex of flowers (D2d), 98 steps

- bisexual (yellow)
- unisexual (blue)

Node	MP state(s)
Angiospermae	bisexual / unisexual
Mesangiospermae	bisexual
Magnoliidae	bisexual
Monocotyledonae	bisexual
Eudicotyledonae	bisexual
Commelinidae	bisexual
Pentapetalae	bisexual
Superasteridae	bisexual
Asteridae	bisexual
Lamiidae	bisexual
Campanulidae	bisexual
Superrosidae	bisexual
Rosidae	bisexual
Malvidae	bisexual
Fabidae	bisexual

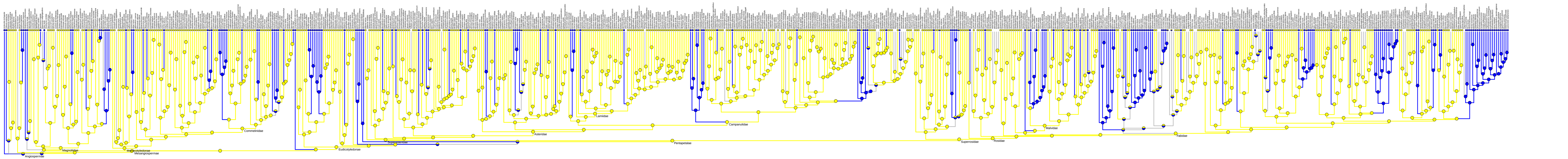

ML ancestral state reconstruction using rayDISC (R:corHMM)
100_A. Functional sex of flowers (D2d), ARDeq model

- bisexual
- unisexual

Node	ML state	Prob
Angiospermae	bisexual	1
Mesangiospermae	bisexual	1
Magnoliidae	bisexual	1
Monocotyledonae	bisexual	1
Eudicotyledonae	bisexual	1
Commelinidae	bisexual	1
Pentapetalae	bisexual	1
Superasteridae	bisexual	1
Asteridae	bisexual	1
Lamiidae	bisexual	1
Campanulidae	bisexual	0.9993
Superrosidae	bisexual	1
Rosidae	bisexual	1
Malvidae	bisexual	0.9997
Fabidae	bisexual	1

Model	LogL	Npar	AIC	AICc	DeltaAICc	w	q01	q10
ARD	−312.55	2	629.11	629.12	1.38	0.24	0.003	8e−04
ARDeq*	−311.86	2	627.73	627.74	0	0.47	0.003	8e−04
ER	−315.27	1	632.54	632.54	4.8	0.04	0.0029	0.0029
UNI01	−313.49	1	628.98	628.98	1.24	0.25	0.003	
UNI10	−354.49	1	710.98	710.99	83.24	0		0.0119

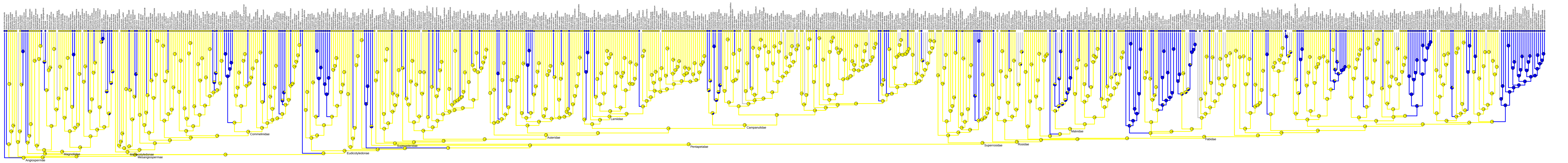

MP ancestral state reconstruction using ancestral.pars (R:phangorn)
100_B. Structural sex of flowers (D2d), 57 steps

- bisexual
- unisexual

Node	MP state(s)
Angiospermae	bisexual
Mesangiospermae	bisexual
Magnoliidae	bisexual
Monocotyledonae	bisexual
Eudicotyledonae	bisexual
Commelinidae	bisexual
Pentapetalae	bisexual
Superasteridae	bisexual
Asteridae	bisexual
Lamiidae	bisexual
Campanulidae	bisexual
Superrosidae	bisexual
Rosidae	bisexual
Malvidae	bisexual
Fabidae	bisexual

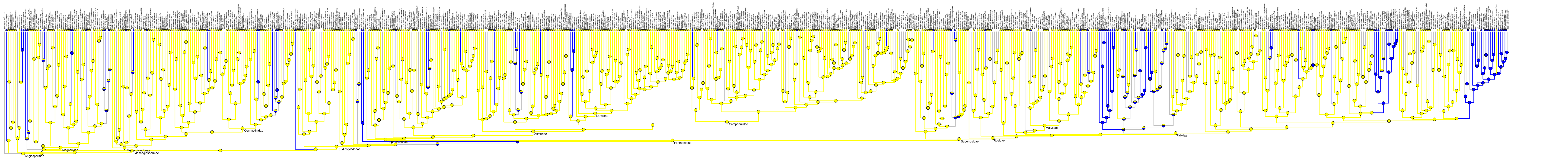

ML ancestral state reconstruction using rayDISC (R:corHMM)
100_B. Structural sex of flowers (D2d), ARDeq model

- bisexual (yellow)
- unisexual (blue)

Node	ML state	Prob
Angiospermae	bisexual	1
Mesangiospermae	bisexual	1
Magnoliidae	bisexual	1
Monocotyledonae	bisexual	1
Eudicotyledonae	bisexual	1
Commelinidae	bisexual	0.9999
Pentapetalae	bisexual	1
Superasteridae	bisexual	1
Asteridae	bisexual	1
Lamiidae	bisexual	0.9999
Campanulidae	bisexual	1
Superrosidae	bisexual	1
Rosidae	bisexual	1
Malvidae	bisexual	1
Fabidae	bisexual	0.9977

Model	LogL	Npar	AIC	AICc	DeltaAICc	w	q01	q10
ARD	−208.28	2	420.56	420.58	1.36	0.28	0.0015	0.0039
ARDeq**	−207.6	2	419.2	419.22	0	0.55	0.0015	0.0039
ER	−209.74	1	421.47	421.48	2.26	0.18	0.0016	0.0016
UNI01	−226.92	1	455.84	455.85	36.63	0	0.0017	
UNI10	−231.58	1	465.15	465.16	45.94	0		0.0167

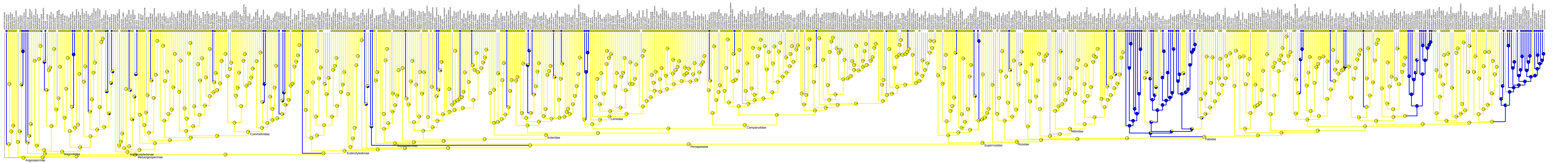

MP ancestral state reconstruction using ancestral.pars
(R:phangorn)
102_B. Ovary position (binary) (D2d), 79 steps

- superior
- inferior

Node	MP state(s)
Angiospermae	superior
Mesangiospermae	superior
Magnoliidae	superior
Monocotyledonae	superior
Eudicotyledonae	superior
Commelinidae	superior
Pentapetalae	superior
Superasteridae	superior
Asteridae	superior
Lamiidae	superior
Campanulidae	superior
Superrosidae	superior
Rosidae	superior / inferior
Malvidae	superior
Fabidae	superior

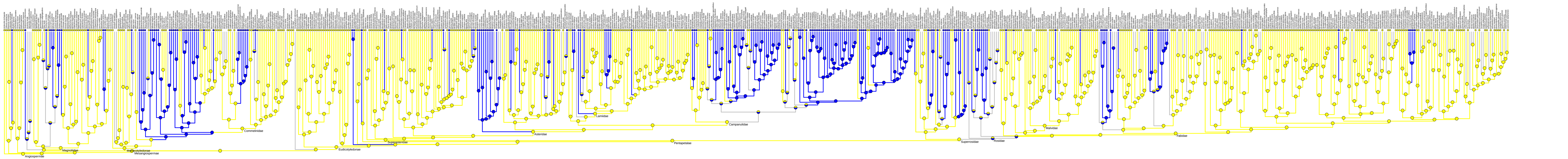

ML ancestral state reconstruction using rayDISC (R:corHMM)
102_B. Ovary position (binary) (D2d), ARDeq model

- superior
- inferior

Node	ML state	Prob
Angiospermae	superior	1
Mesangiospermae	superior	1
Magnoliidae	superior	0.9997
Monocotyledonae	superior	0.9983
Eudicotyledonae	superior	1
Commelinidae	superior	0.766
Pentapetalae	superior	0.9995
Superasteridae	superior	0.9998
Asteridae	superior	0.9785
Lamiidae	superior	0.9791
Campanulidae	superior	0.8204
Superrosidae	superior	0.9776
Rosidae	superior	0.9773
Malvidae	superior	1
Fabidae	superior	0.9997

Model	LogL	Npar	AIC	AICc	DeltaAICc	w	q01	q10
ARD	−272.01	2	548.03	548.04	1.38	0.25	0.0021	0.0036
ARDeq*	−271.32	2	546.65	546.66	0	0.49	0.0021	0.0036
ER	−272.95	1	547.89	547.9	1.24	0.26	0.0024	0.0024
UNI01	−281.44	1	564.88	564.89	18.23	0	0.0027	
UNI10	−294.63	1	591.27	591.27	44.61	0		0.0099

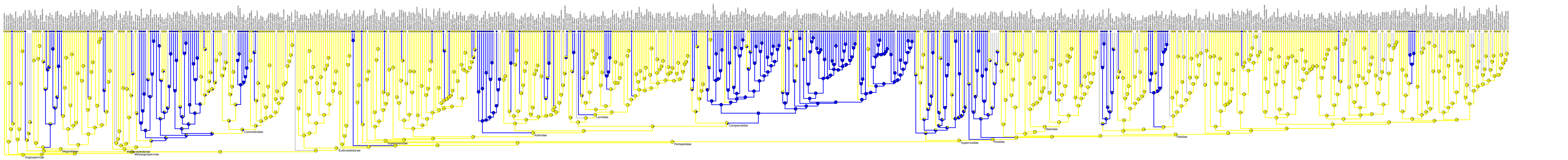

MP ancestral state reconstruction using ancestral.pars
(R:phangorn)
201_A. Perianth presence (D2c), 21 steps

absent
present

Node	MP state(s)
Angiospermae	present
Mesangiospermae	present
Magnoliidae	present
Monocotyledonae	present
Eudicotyledonae	present
Commelinidae	present
Pentapetalae	present
Superasteridae	present
Asteridae	present
Lamiidae	present
Campanulidae	present
Superrosidae	present
Rosidae	present
Malvidae	present
Fabidae	present

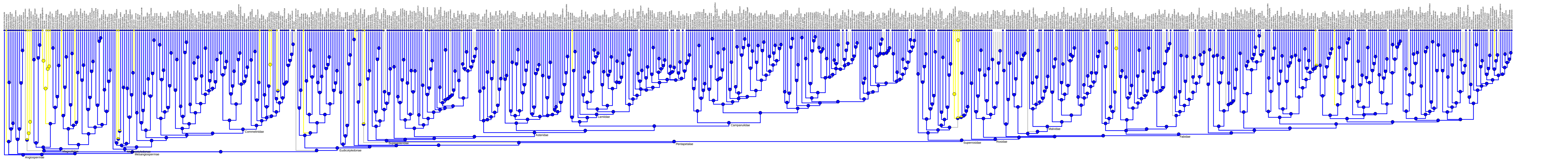

ML ancestral state reconstruction using rayDISC (R:corHMM)
201_A. Perianth presence (D2c), UNI10 model

absent
present

Node	ML state	Prob
Angiospermae	present	1
Mesangiospermae	present	1
Magnoliidae	present	1
Monocotyledonae	present	1
Eudicotyledonae	present	1
Commelinidae	present	1
Pentapetalae	present	1
Superasteridae	present	1
Asteridae	present	1
Lamiidae	present	1
Campanulidae	present	1
Superrosidae	present	1
Rosidae	present	1
Malvidae	present	1
Fabidae	present	1

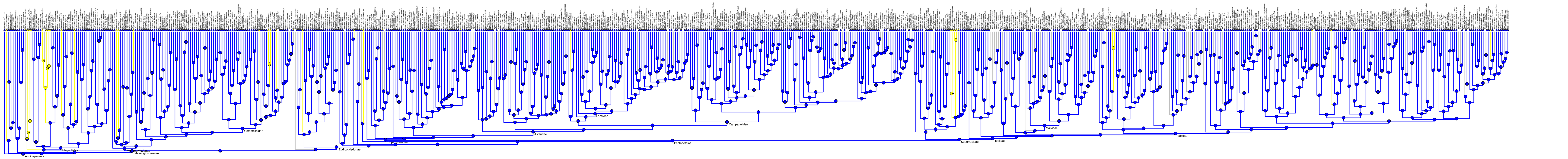

Model	LogL	Npar	AIC	AICc	DeltaAICc	w	q01	q10
ARD	−96.83	2	197.67	197.69	2.01	0.13	0	5e−04
ARDeq	−96.14	2	196.28	196.3	0.62	0.26	0	5e−04
ER	−97.11	1	196.21	196.22	0.54	0.27	5e−04	5e−04
UNI01	−109.88	1	221.76	221.76	26.09	0	0.0224	
UNI10*	−96.83	1	195.67	195.67	0	0.35		5e−04

MP ancestral state reconstruction using ancestral.pars
(R:phangorn)
201_B. Number of perianth parts (3−state) (D2c), 86 steps

- one to five (1−5)
- six to ten (6−10)
- more than ten (>10)

Node	MP state(s)
Angiospermae	six to ten (6−10) / more than ten (>10)
Mesangiospermae	six to ten (6−10)
Magnoliidae	six to ten (6−10)
Monocotyledonae	six to ten (6−10)
Eudicotyledonae	six to ten (6−10)
Commelinidae	six to ten (6−10)
Pentapetalae	six to ten (6−10)
Superasteridae	six to ten (6−10)
Asteridae	six to ten (6−10)
Lamiidae	six to ten (6−10)
Campanulidae	six to ten (6−10)
Superrosidae	six to ten (6−10)
Rosidae	six to ten (6−10)
Malvidae	six to ten (6−10)
Fabidae	six to ten (6−10)

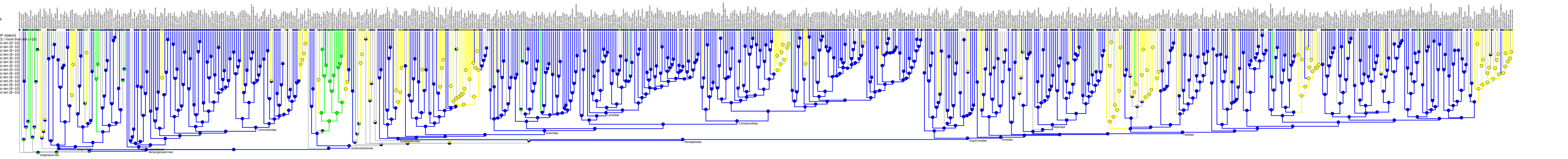

ML ancestral state reconstruction using rayDISC (R:corHMM)
201_B. Number of perianth parts (3−state) (D2c), ARDeq model

- one to five (1−5)
- six to ten (6−10)
- more than ten (>10)

Node	ML state	Prob
Angiospermae	more than ten (>10)	1
Mesangiospermae	more than ten (>10)	0.9999
Magnoliidae	more than ten (>10)	0.9999
Monocotyledonae	six to ten (6−10)	0.5684
Eudicotyledonae	more than ten (>10)	0.9952
Commelinidae	six to ten (6−10)	0.9999
Pentapetalae	six to ten (6−10)	0.9952
Superasteridae	six to ten (6−10)	0.9974
Asteridae	six to ten (6−10)	1
Lamiidae	six to ten (6−10)	0.9999
Campanulidae	six to ten (6−10)	0.9999
Superrosidae	six to ten (6−10)	0.9991
Rosidae	six to ten (6−10)	0.9994
Malvidae	six to ten (6−10)	0.9999
Fabidae	six to ten (6−10)	0.9993

Model	LogL	Npar	AIC	AICc	DeltaAICc	w	q01	···
ARD	−325.17	6	662.34	662.45	2.2	0.25	0.0038	···
ARDeq**	−324.07	6	660.15	660.25	0	0.74	0.0038	···
ER	−350.39	1	702.78	702.79	42.53	0	0.0013	···
SYM	−339.53	3	685.06	685.09	24.83	0	0.002	···
SYMeq	−338.46	3	682.91	682.94	22.69	0	0.002	···
ORD	−331.17	4	670.34	670.39	10.13	0	0.004	···
ORDeq	−330.07	4	668.14	668.19	7.94	0.01	0.004	···
ORDSYM	−341.97	2	687.94	687.95	27.7	0	0.0021	···
ORDSYMeq	−340.91	2	685.81	685.83	25.57	0	0.0021	···
ORDER	−351.35	1	704.71	704.71	44.46	0	0.0014	···

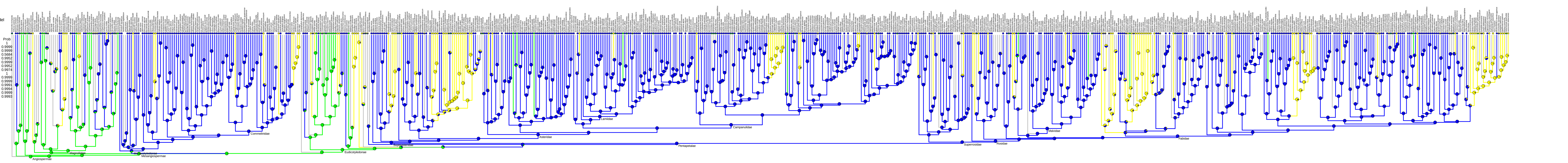

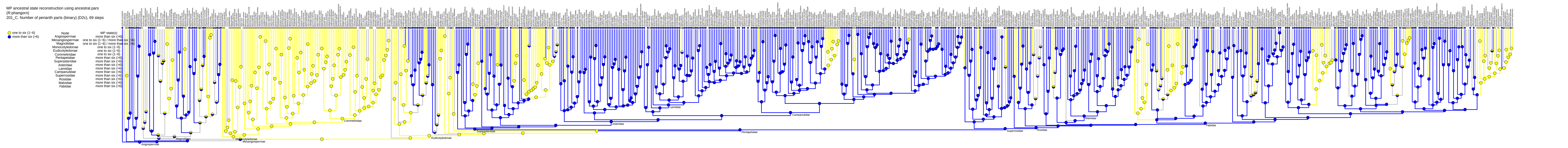
MP ancestral state reconstruction using ancestral.pars
(R:phangorn)
201_C. Number of perianth parts (binary) (D2c), 69 steps
one to six (1−6)
more than six (>6)
Node | MP state(s)
Angiospermae | more than six (>6)
Mesangiospermae | one to six (1−6) / more than six (>6)
Magnoliidae | one to six (1−6) / more than six (>6)
Monocotyledonae | one to six (1−6)
Eudicotyledonae | one to six (1−6)
Commelinidae | one to six (1−6)
Pentapetalae | more than six (>6)
Superasteridae | more than six (>6)
Asteridae | more than six (>6)
Lamiidae | more than six (>6)
Campanulidae | more than six (>6)
Superrosidae | more than six (>6)
Rosidae | more than six (>6)
Malvidae | more than six (>6)
Fabidae | more than six (>6)
Angiospermae
Magnoliidae
Monocotyledonae
Mesangiospermae
Commelinidae
Eudicotyledonae
Superasteridae
Asteridae
Lamiidae
Campanulidae
Pentapetalae
Superrosidae
Rosidae
Malvidae
Fabidae

ML ancestral state reconstruction using rayDISC (R:corHMM)
201_C. Number of perianth parts (binary) (D2c), ARDeq model

- one to six (1−6)
- more than six (>6)

Node	ML state	Prob
Angiospermae	more than six (>6)	1
Mesangiospermae	more than six (>6)	1
Magnoliidae	more than six (>6)	1
Monocotyledonae	one to six (1−6)	0.7998
Eudicotyledonae	more than six (>6)	1
Commelinidae	one to six (1−6)	1
Pentapetalae	more than six (>6)	1
Superasteridae	more than six (>6)	1
Asteridae	more than six (>6)	1
Lamiidae	more than six (>6)	1
Campanulidae	more than six (>6)	1
Superrosidae	more than six (>6)	1
Rosidae	more than six (>6)	1
Malvidae	more than six (>6)	1
Fabidae	more than six (>6)	1

Model	LogL	Npar	AIC	AICc	DeltaAICc	w	q01	q10
ARD	−247.75	2	499.51	499.52	1.39	0.33	6e−04	0.0026
ARDeq**	−247.06	2	498.12	498.14	0	0.66	6e−04	0.0026
ER	−254.33	1	510.67	510.67	12.54	0	0.0022	0.0022
UNI01	−323.73	1	649.45	649.46	151.32	0	0.0083	
UNI10	−252.79	1	507.58	507.59	9.45	0.01		0.0029

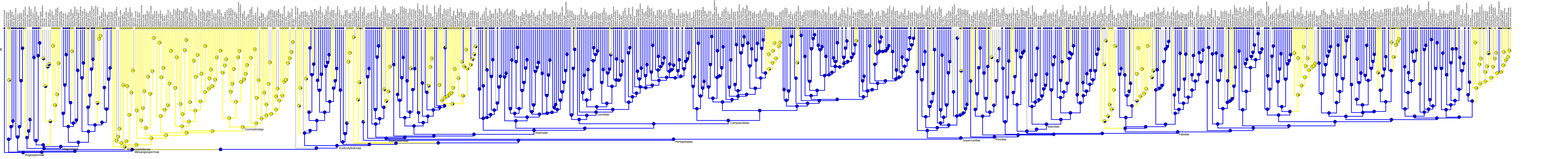

MP ancestral state reconstruction using ancestral.pars
(R:phangorn)
230_A. Perianth phyllotaxy (binary) (D2d), 16 steps

- whorled
- spiral

Node	MP state(s)
Angiospermae	whorled / spiral
Mesangiospermae	whorled
Magnoliidae	whorled
Monocotyledonae	whorled
Eudicotyledonae	whorled
Commelinidae	whorled
Pentapetalae	whorled
Superasteridae	whorled
Asteridae	whorled
Lamiidae	whorled
Campanulidae	whorled
Superrosidae	whorled
Rosidae	whorled
Malvidae	whorled
Fabidae	whorled

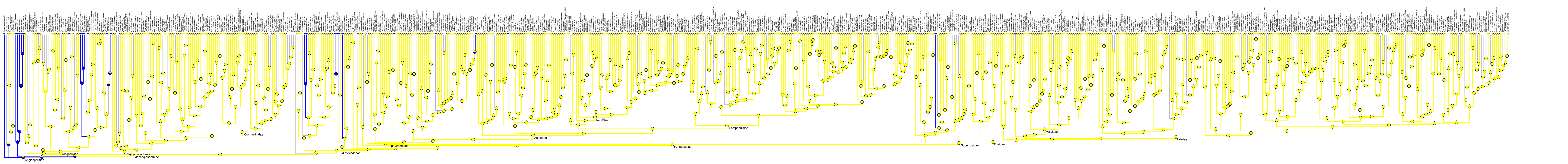

ML ancestral state reconstruction using rayDISC (R:corHMM)
230_A. Perianth phyllotaxy (binary) (D2d), ARD model

- whorled (yellow)
- spiral (blue)

Node	ML state	Prob
Angiospermae	spiral	1
Mesangiospermae	spiral	1
Magnoliidae	spiral	0.9997
Monocotyledonae	spiral	0.5143
Eudicotyledonae	spiral	0.9996
Commelinidae	whorled	0.9998
Pentapetalae	whorled	0.9804
Superasteridae	whorled	0.9835
Asteridae	whorled	0.9997
Lamiidae	whorled	1
Campanulidae	whorled	1
Superrosidae	whorled	0.9919
Rosidae	whorled	0.9951
Malvidae	whorled	1
Fabidae	whorled	1

Model	LogL	Npar	AIC	AICc	DeltaAICc	w	q01	q10
ARD***	−70.06	2	144.12	144.13	0	0.99	2e−04	0.011
ARDeq	−77.1	2	158.19	158.21	14.07	0	4e−04	5e−04
ER	−77.75	1	157.5	157.5	13.37	0	4e−04	4e−04
UNI01	−78	1	158	158.01	13.87	0	4e−04	
UNI10	−76.04	1	154.08	154.08	9.95	0.01		0.0178

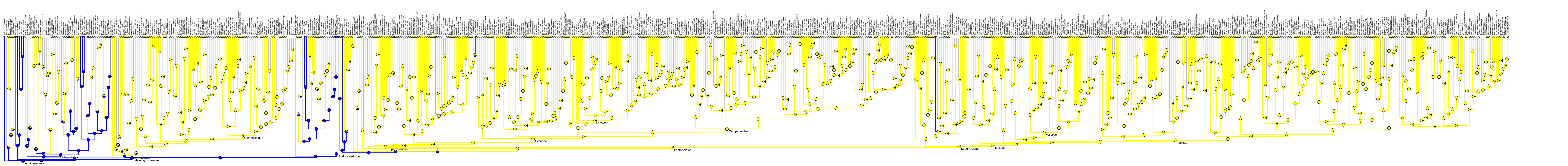

MP ancestral state reconstruction using ancestral.pars
(R:phangorn)
231_A. Number of perianth whorls (D2c), 73 steps

- one (1)
- two (2)
- more than two (>2)

Node	MP state(s)
Angiospermae	two (2) / more than two (>2)
Mesangiospermae	two (2) / more than two (>2)
Magnoliidae	one (1) / two (2) / more than two (>2)
Monocotyledonae	two (2)
Eudicotyledonae	two (2) / more than two (>2)
Commelinidae	two (2)
Pentapetalae	two (2)
Superasteridae	two (2)
Asteridae	two (2)
Lamiidae	two (2)
Campanulidae	two (2)
Superrosidae	two (2)
Rosidae	two (2)
Malvidae	two (2)
Fabidae	two (2)

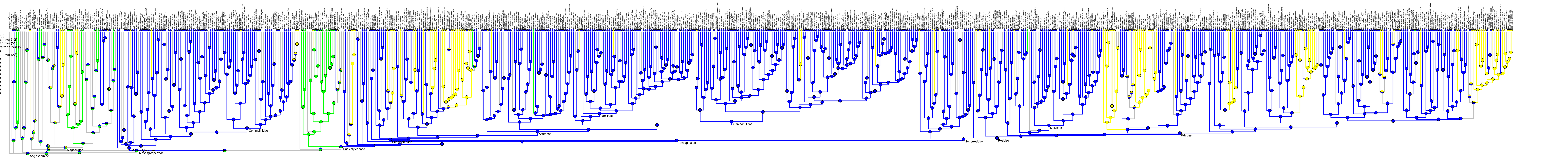

ML ancestral state reconstruction using rayDISC (R:corHMM)
231_A. Number of perianth whorls (D2c), ARDeq model

- one (1)
- two (2)
- more than two (>2)

Node	ML state	Prob
Angiospermae	more than two (>2)	1
Mesangiospermae	more than two (>2)	1
Magnoliidae	more than two (>2)	1
Monocotyledonae	two (2)	0.7194
Eudicotyledonae	more than two (>2)	0.992
Commelinidae	two (2)	1
Pentapetalae	two (2)	0.9998
Superasteridae	two (2)	0.9998
Asteridae	two (2)	0.9998
Lamiidae	two (2)	0.9999
Campanulidae	two (2)	1
Superrosidae	two (2)	1
Rosidae	two (2)	1
Malvidae	two (2)	0.9999
Fabidae	two (2)	0.9977

Model	LogL	Npar	AIC	AICc	DeltaAICc	w	q01	···
ARD	−269.95	6	551.91	552.02	2.2	0.25	0.0046	···
ARDeq**	−268.86	6	549.71	549.82	0	0.75	0.0046	···
ER	−311.23	1	624.46	624.46	74.64	0	0.001	···
SYM	−292.92	3	591.85	591.88	42.06	0	0.0019	···
SYMeq	−291.92	3	589.84	589.87	40.05	0	0.0019	···
ORD	−277.05	4	562.1	562.15	12.33	0	0.0045	···
ORDeq	−275.95	4	559.9	559.95	10.13	0	0.0045	···
ORDSYM	−298.13	2	600.27	600.28	50.46	0	0.0019	···
ORDSYMeq	−297.05	2	598.1	598.12	48.3	0	0.0019	···
ORDER	−313.56	1	629.13	629.13	79.32	0	0.0012	···

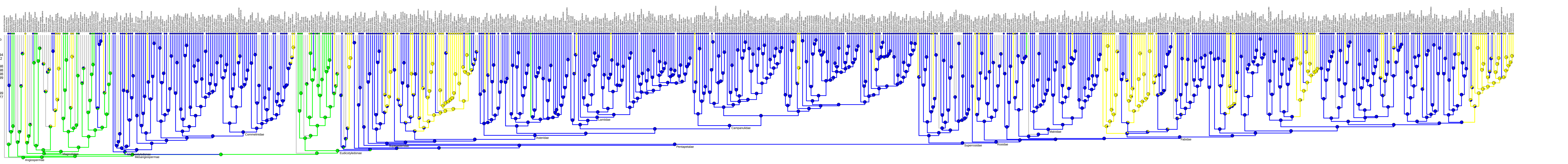

MP ancestral state reconstruction using ancestral.pars
(R:phangorn)
232_A. Perianth merism (4−state) (D2c), 84 steps

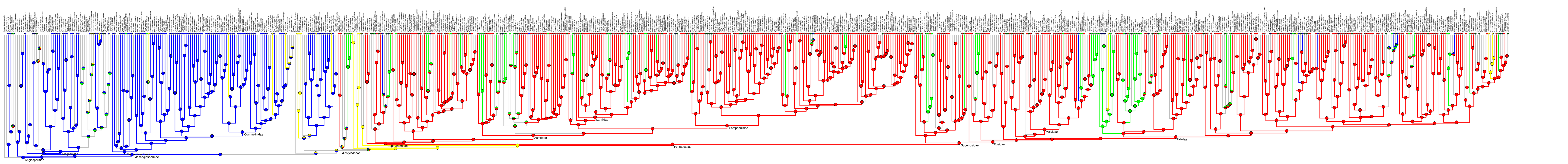

Node	MP state(s)
Angiospermae	trimerous
Mesangiospermae	trimerous
Magnoliidae	trimerous
Monocotyledonae	trimerous
Eudicotyledonae	dimerous / trimerous
Commelinidae	trimerous
Pentapetalae	pentamerous
Superasteridae	pentamerous
Asteridae	pentamerous
Lamiidae	pentamerous
Campanulidae	pentamerous
Superrosidae	pentamerous
Rosidae	pentamerous
Malvidae	pentamerous
Fabidae	pentamerous

ML ancestral state reconstruction using rayDISC (R:corHMM)
232_A. Perianth merism (4−state) (D2c), SYMeq model

- dimerous
- trimerous
- tetramerous
- pentamerous

Node	ML state	Prob
Angiospermae	trimerous	1
Mesangiospermae	trimerous	1
Magnoliidae	trimerous	1
Monocotyledonae	trimerous	1
Eudicotyledonae	trimerous	0.9676
Commelinidae	trimerous	1
Pentapetalae	pentamerous	0.9997
Superasteridae	pentamerous	1
Asteridae	pentamerous	1
Lamiidae	pentamerous	1
Campanulidae	pentamerous	1
Superrosidae	pentamerous	1
Rosidae	pentamerous	1
Malvidae	pentamerous	1
Fabidae	pentamerous	1

Model	LogL	Npar	AIC	AICc	DeltaAICc	w	q01	⋯
ARD	−323.65	12	671.31	671.71	10.48	0	0	⋯
ARDeq	−322.29	12	668.57	668.98	7.75	0.02	0	⋯
ER	−360.68	1	723.35	723.36	62.13	0	8e−04	⋯
SYM	−325.94	6	663.88	663.99	2.76	0.2	0.0013	⋯
SYMeq**	−324.56	6	661.12	661.22	0	0.78	0.0013	⋯
ORD	−336.72	6	685.43	685.54	24.32	0	0	⋯
ORDeq	−335.4	6	682.81	682.92	21.69	0	0	⋯
ORDSYM	−344.43	3	694.87	694.9	33.68	0	0.002	⋯
ORDSYMeq	−343.06	3	692.12	692.15	30.93	0	0.002	⋯
ORDER	−345.97	1	693.93	693.94	32.71	0	0.0025	⋯

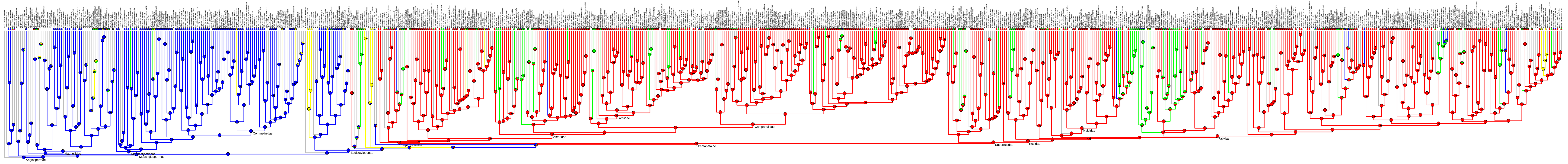

MP ancestral state reconstruction using ancestral.pars (R:phangorn)
232_B. Perianth merism (3−state) (D2c), 70 steps

- trimerous
- tetramerous
- pentamerous

Node	MP state(s)
Angiospermae	trimerous
Mesangiospermae	trimerous
Magnoliidae	trimerous
Monocotyledonae	trimerous
Eudicotyledonae	trimerous
Commelinidae	trimerous
Pentapetalae	pentamerous
Superasteridae	pentamerous
Asteridae	pentamerous
Lamiidae	pentamerous
Campanulidae	pentamerous
Superrosidae	pentamerous
Rosidae	pentamerous
Malvidae	pentamerous
Fabidae	pentamerous

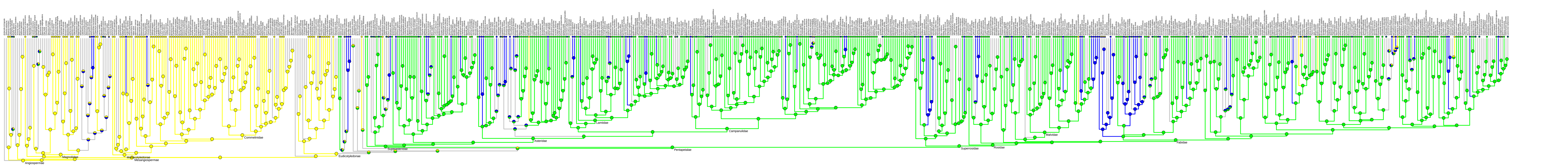

ML ancestral state reconstruction using rayDISC (R:corHMM)
232_B. Perianth merism (3−state) (D2c), SYMeq model

- trimerous
- tetramerous
- pentamerous

Node	ML state	Prob
Angiospermae	trimerous	1
Mesangiospermae	trimerous	1
Magnoliidae	trimerous	1
Monocotyledonae	trimerous	1
Eudicotyledonae	trimerous	0.9795
Commelinidae	trimerous	1
Pentapetalae	pentamerous	0.9998
Superasteridae	pentamerous	1
Asteridae	pentamerous	0.9999
Lamiidae	pentamerous	1
Campanulidae	pentamerous	1
Superrosidae	pentamerous	1
Rosidae	pentamerous	1
Malvidae	pentamerous	1
Fabidae	pentamerous	1

Model	LogL	Npar	AIC	AICc	DeltaAICc	w	q01	···
ARD	−256.15	6	524.3	524.41	6.05	0.02	8e−04	···
ARDeq	−255.07	6	522.14	522.25	3.89	0.06	8e−04	···
ER	−281.41	1	564.83	564.83	46.47	0	0.0011	···
SYM	−257.26	3	520.52	520.55	2.19	0.14	0.001	···
SYMeq*	−256.17	3	518.33	518.36	0	0.42	0.001	···
ORD	−256.99	4	521.98	522.03	3.67	0.07	0.001	···
ORDeq	−255.92	4	519.84	519.89	1.52	0.19	0.001	···
ORDSYM	−259.98	2	523.96	523.98	5.62	0.03	0.0015	···
ORDSYMeq	−258.89	2	521.78	521.79	3.43	0.07	0.0015	···
ORDER	−262.3	1	526.59	526.6	8.24	0.01	0.0024	···

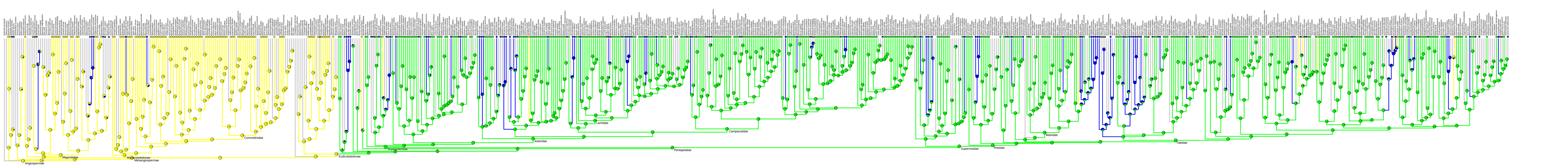

MP ancestral state reconstruction using ancestral.pars
(R:phangorn)
234_A. Perianth differentiation (binary) (D2d), 63 steps

- undifferentiated (yellow)
- differentiated (blue)

Node	MP state(s)
Angiospermae	undifferentiated
Mesangiospermae	undifferentiated
Magnoliidae	undifferentiated / differentiated
Monocotyledonae	undifferentiated
Eudicotyledonae	undifferentiated
Commelinidae	undifferentiated
Pentapetalae	differentiated
Superasteridae	differentiated
Asteridae	differentiated
Lamiidae	differentiated
Campanulidae	differentiated
Superrosidae	differentiated
Rosidae	differentiated
Malvidae	differentiated
Fabidae	differentiated

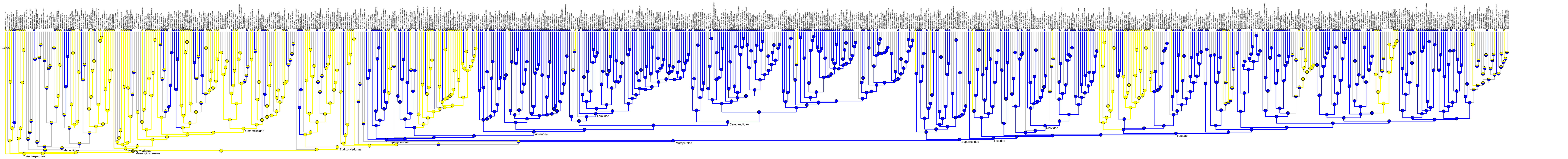

ML ancestral state reconstruction using rayDISC (R:corHMM)
234_A. Perianth differentiation (binary) (D2d), ARDeq model

- undifferentiated (yellow)
- differentiated (blue)

Node	ML state	Prob
Angiospermae	undifferentiated	1
Mesangiospermae	undifferentiated	1
Magnoliidae	undifferentiated	0.9999
Monocotyledonae	undifferentiated	1
Eudicotyledonae	undifferentiated	0.9899
Commelinidae	undifferentiated	1
Pentapetalae	differentiated	0.9479
Superasteridae	differentiated	0.9492
Asteridae	differentiated	0.9982
Lamiidae	differentiated	0.9999
Campanulidae	differentiated	1
Superrosidae	differentiated	0.9921
Rosidae	differentiated	0.9953
Malvidae	differentiated	0.9989
Fabidae	differentiated	0.9872

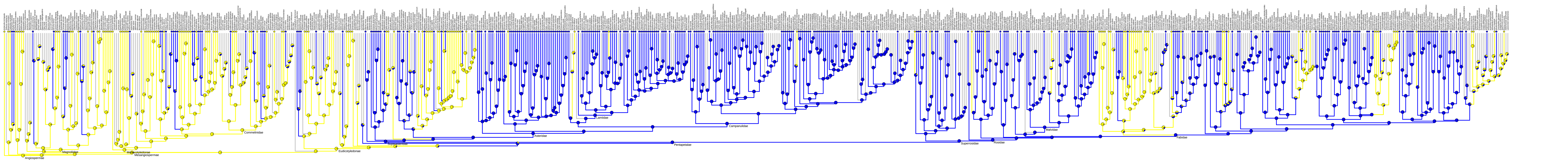

Model	LogL	Npar	AIC	AICc	DeltaAICc	w	q01	q10
ARD	−199.39	2	402.77	402.79	1.38	0.33	0.0045	0.0015
ARDeq**	−198.69	2	401.39	401.4	0	0.67	0.0045	0.0015
ER	−208.5	1	419	419	17.6	0	0.0025	0.0025
UNI01	−225.54	1	453.09	453.09	51.69	0	0.0086	
UNI10	−234.01	1	470.02	470.02	68.62	0		0.0034

MP ancestral state reconstruction using ancestral.pars
(R:phangorn)
204_A. Fusion of perianth (D2c), 77 steps

- free (<5%)
- fused (>5%)

Node	MP state(s)
Angiospermae	free (<5%)
Mesangiospermae	free (<5%)
Magnoliidae	free (<5%)
Monocotyledonae	free (<5%)
Eudicotyledonae	free (<5%)
Commelinidae	free (<5%)
Pentapetalae	free (<5%) / fused (>5%)
Superasteridae	free (<5%) / fused (>5%)
Asteridae	free (<5%)
Lamiidae	free (<5%) / fused (>5%)
Campanulidae	fused (>5%)
Superrosidae	free (<5%) / fused (>5%)
Rosidae	free (<5%) / fused (>5%)
Malvidae	free (<5%)
Fabidae	free (<5%)

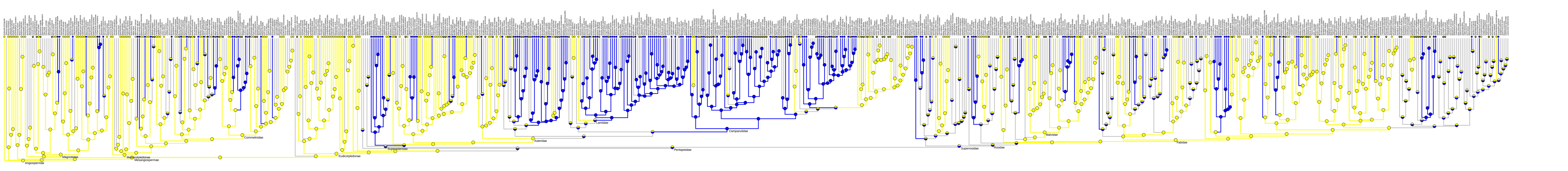

ML ancestral state reconstruction using rayDISC (R:corHMM)
204_A. Fusion of perianth (D2c), ARDeq model

- free (<5%)
- fused (>5%)

Node	ML state	Prob
Angiospermae	free (<5%)	1
Mesangiospermae	free (<5%)	1
Magnoliidae	free (<5%)	0.9999
Monocotyledonae	free (<5%)	0.9999
Eudicotyledonae	free (<5%)	1
Commelinidae	free (<5%)	0.9989
Pentapetalae	free (<5%)	0.9995
Superasteridae	free (<5%)	0.9935
Asteridae	free (<5%)	0.8548
Lamiidae	fused (>5%)	0.9552
Campanulidae	fused (>5%)	0.94
Superrosidae	free (<5%)	0.9995
Rosidae	free (<5%)	0.9995
Malvidae	free (<5%)	0.9986
Fabidae	free (<5%)	1

Model	LogL	Npar	AIC	AICc	DeltaAICc	w	q01	q10
ARD	−224.91	2	453.83	453.84	1.38	0.21	0.0044	0.0035
ARDeq*	−224.22	2	452.45	452.46	0	0.41	0.0044	0.0035
ER	−225.3	1	452.6	452.6	0.14	0.38	0.0042	0.0042
UNI01	−236	1	473.99	474	21.54	0	0.0049	
UNI10	−236.58	1	475.16	475.16	22.7	0		0.0081

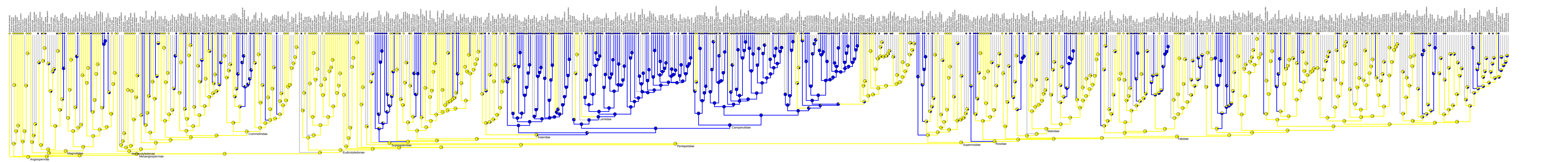

MP ancestral state reconstruction using ancestral.pars
(R:phangorn)
207_A. Symmetry of perianth (binary) (D2d), 55 steps

- actinomorphic (yellow)
- zygomorphic (blue)

Node	MP state(s)
Angiospermae	actinomorphic
Mesangiospermae	actinomorphic
Magnoliidae	actinomorphic
Monocotyledonae	actinomorphic
Eudicotyledonae	actinomorphic
Commelinidae	actinomorphic
Pentapetalae	actinomorphic
Superasteridae	actinomorphic
Asteridae	actinomorphic
Lamiidae	actinomorphic
Campanulidae	actinomorphic
Superrosidae	actinomorphic
Rosidae	actinomorphic
Malvidae	actinomorphic
Fabidae	actinomorphic

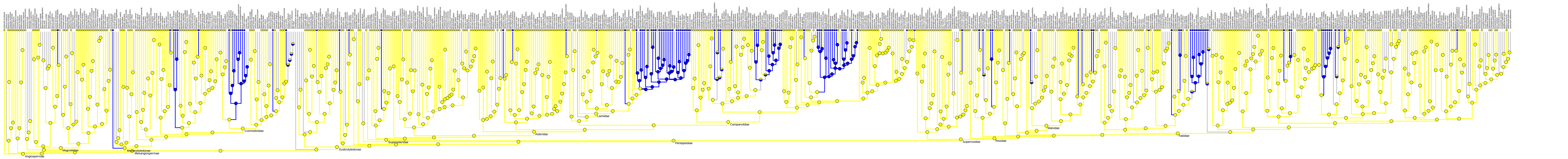

ML ancestral state reconstruction using rayDISC (R:corHMM)
207_A. Symmetry of perianth (binary) (D2d), ARDeq model

- actinomorphic (yellow)
- zygomorphic (blue)

Node	ML state	Prob
Angiospermae	actinomorphic	1
Mesangiospermae	actinomorphic	1
Magnoliidae	actinomorphic	1
Monocotyledonae	actinomorphic	0.9997
Eudicotyledonae	actinomorphic	1
Commelinidae	actinomorphic	0.9953
Pentapetalae	actinomorphic	1
Superasteridae	actinomorphic	1
Asteridae	actinomorphic	1
Lamiidae	actinomorphic	0.9959
Campanulidae	actinomorphic	0.9999
Superrosidae	actinomorphic	1
Rosidae	actinomorphic	1
Malvidae	actinomorphic	0.9997
Fabidae	actinomorphic	1

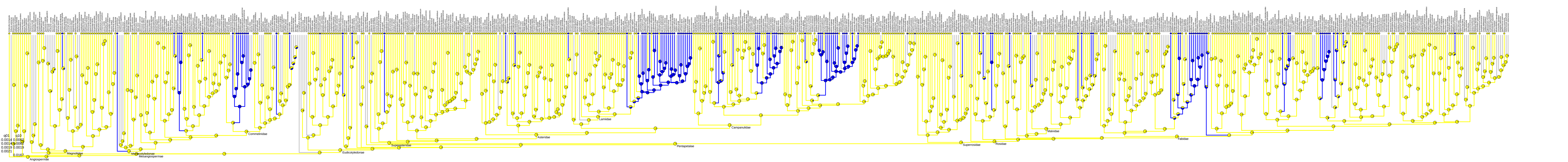

Model	LogL	Npar	AIC	AICc	DeltaAICc	w	q01	q10
ARD	−211.59	2	427.17	427.19	1.37	0.33	0.0014	0.0062
ARDeq**	−210.9	2	425.8	425.81	0	0.67	0.0014	0.0062
ER	−223.67	1	449.33	449.34	23.53	0	0.0019	0.0019
UNI01	−1e+06	1	2000002	2000002.01	1999576.19	0	0.0021	
UNI10	−219.37	1	440.74	440.75	14.93	0		0.0167

MP ancestral state reconstruction using ancestral.pars
(R:phangorn)
301_B. Number of fertile stamens (3−state) (D2c), 156 steps

- one to five (1−5)
- six to ten (6−10)
- more than ten (>10)

Node	MP state(s)
Angiospermae	more than ten (>10)
Mesangiospermae	one to five (1−5) / more than ten (>10)
Magnoliidae	more than ten (>10)
Monocotyledonae	six to ten (6−10)
Eudicotyledonae	one to five (1−5) / six to ten (6−10)
Commelinidae	six to ten (6−10)
Pentapetalae	six to ten (6−10)
Superasteridae	six to ten (6−10)
Asteridae	one to five (1−5) / six to ten (6−10)
Lamiidae	one to five (1−5)
Campanulidae	one to five (1−5)
Superrosidae	six to ten (6−10)
Rosidae	six to ten (6−10)
Malvidae	six to ten (6−10)
Fabidae	six to ten (6−10)

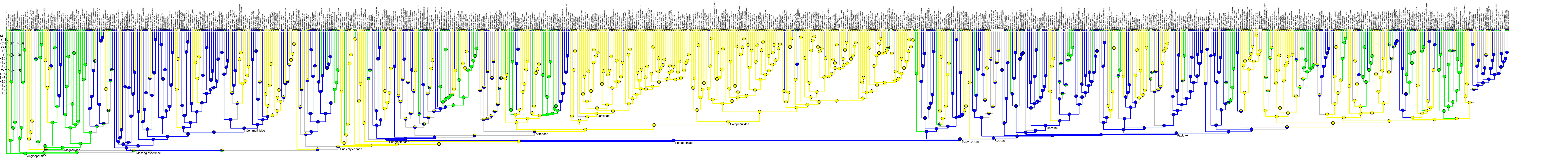

ML ancestral state reconstruction using rayDISC (R:corHMM)
301_B. Number of fertile stamens (3−state) (D2c), ARDeq model

- one to five (1−5)
- six to ten (6−10)
- more than ten (>10)

Node	ML state	Prob
Angiospermae	more than ten (>10)	1
Mesangiospermae	more than ten (>10)	1
Magnoliidae	more than ten (>10)	1
Monocotyledonae	six to ten (6−10)	0.5427
Eudicotyledonae	more than ten (>10)	1
Commelinidae	six to ten (6−10)	0.9998
Pentapetalae	more than ten (>10)	1
Superasteridae	more than ten (>10)	1
Asteridae	more than ten (>10)	0.9994
Lamiidae	one to five (1−5)	0.9994
Campanulidae	one to five (1−5)	0.9957
Superrosidae	more than ten (>10)	1
Rosidae	more than ten (>10)	0.9999
Malvidae	more than ten (>10)	0.9346
Fabidae	more than ten (>10)	1

Model	LogL	Npar	AIC	AICc	DeltaAICc	w	q01	···
ARD	−491.49	6	994.98	995.08	2.2	0.25	4e−04	···
ARDeq**	−490.39	6	992.78	992.89	0	0.75	4e−04	···
ER	−537.6	1	1077.2	1077.2	84.32	0	0.0027	···
SYM	−530.07	3	1066.15	1066.18	73.29	0	0.0039	···
SYMeq	−529.24	3	1064.49	1064.52	71.63	0	0.0039	···
ORD	−508.62	4	1025.23	1025.28	32.4	0	7e−04	···
ORDeq	−508.11	4	1024.23	1024.28	31.39	0	8e−04	···
ORDSYM	−533.66	2	1071.33	1071.34	78.46	0	0.0043	···
ORDSYMeq	−532.78	2	1069.56	1069.58	76.69	0	0.0043	···
ORDER	−533.69	1	1069.38	1069.39	76.5	0	0.0044	···

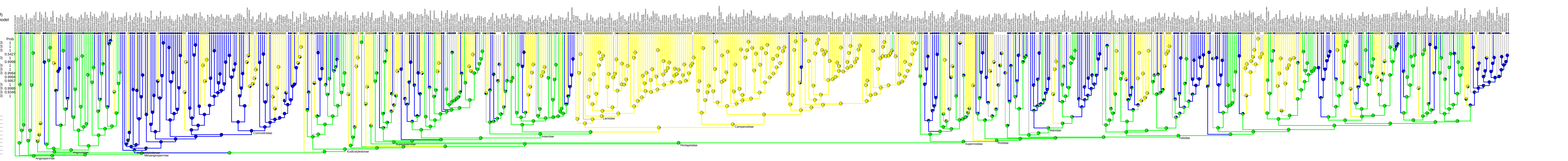

MP ancestral state reconstruction using ancestral.pars
(R:phangorn)
301_C. Number of fertile stamens (binary) (D2c), 94 steps

- one to six (1−6)
- more than six (>6)

Node	MP state(s)
Angiospermae	more than six (>6)
Mesangiospermae	one to six (1−6) / more than six (>6)
Magnoliidae	one to six (1−6) / more than six (>6)
Monocotyledonae	one to six (1−6)
Eudicotyledonae	one to six (1−6)
Commelinidae	one to six (1−6)
Pentapetalae	more than six (>6)
Superasteridae	more than six (>6)
Asteridae	one to six (1−6) / more than six (>6)
Lamiidae	one to six (1−6)
Campanulidae	one to six (1−6)
Superrosidae	more than six (>6)
Rosidae	more than six (>6)
Malvidae	more than six (>6)
Fabidae	more than six (>6)

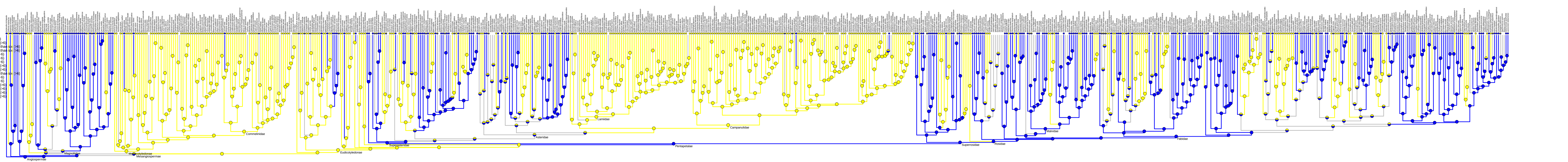

ML ancestral state reconstruction using rayDISC (R:corHMM)
301_C. Number of fertile stamens (binary) (D2c), ARDeq model

- one to six (1−6)
- more than six (>6)

Node	ML state	Prob
Angiospermae	more than six (>6)	1
Mesangiospermae	more than six (>6)	1
Magnoliidae	more than six (>6)	1
Monocotyledonae	more than six (>6)	0.5725
Eudicotyledonae	more than six (>6)	0.9998
Commelinidae	one to six (1−6)	1
Pentapetalae	more than six (>6)	1
Superasteridae	more than six (>6)	1
Asteridae	more than six (>6)	0.9999
Lamiidae	one to six (1−6)	0.9987
Campanulidae	one to six (1−6)	0.9926
Superrosidae	more than six (>6)	1
Rosidae	more than six (>6)	1
Malvidae	more than six (>6)	1
Fabidae	more than six (>6)	1

Model	LogL	Npar	AIC	AICc	DeltaAICc	w	q01	q10
ARD	−288.16	2	580.33	580.34	1.39	0.33	5e−04	0.0059
ARDeq**	−287.47	2	578.94	578.96	0	0.67	5e−04	0.0059
ER	−323.09	1	648.19	648.19	69.24	0	0.0031	0.0031
UNI01	−362.46	1	726.93	726.93	147.98	0	0.0046	
UNI10	−327.51	1	657.02	657.02	78.07	0		0.0074

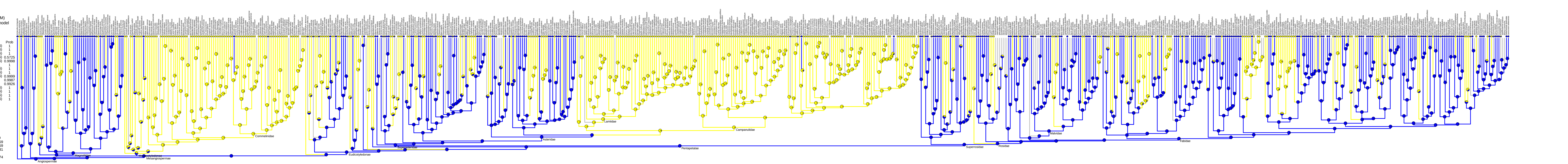

MP ancestral state reconstruction using ancestral.pars
(R:phangorn)
330_A. Androecium structural phyllotaxy (binary) (D2d), 12 steps

- whorled
- spiral

Node	MP state(s)
Angiospermae	whorled / spiral
Mesangiospermae	whorled / spiral
Magnoliidae	whorled / spiral
Monocotyledonae	whorled
Eudicotyledonae	whorled
Commelinidae	whorled
Pentapetalae	whorled
Superasteridae	whorled
Asteridae	whorled
Lamiidae	whorled
Campanulidae	whorled
Superrosidae	whorled
Rosidae	whorled
Malvidae	whorled
Fabidae	whorled

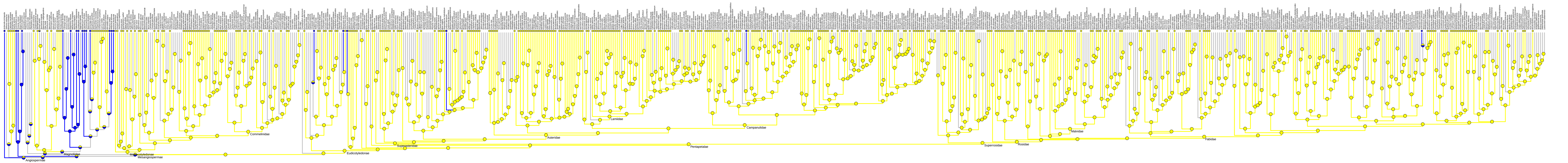

ML ancestral state reconstruction using rayDISC (R:corHMM)
330_A. Androecium structural phyllotaxy (binary) (D2d), UNI01 model

- whorled
- spiral

Node	ML state	Prob
Angiospermae	whorled	1
Mesangiospermae	whorled	1
Magnoliidae	whorled	1
Monocotyledonae	whorled	1
Eudicotyledonae	whorled	1
Commelinidae	whorled	1
Pentapetalae	whorled	1
Superasteridae	whorled	1
Asteridae	whorled	1
Lamiidae	whorled	1
Campanulidae	whorled	1
Superrosidae	whorled	1
Rosidae	whorled	1
Malvidae	whorled	1
Fabidae	whorled	1

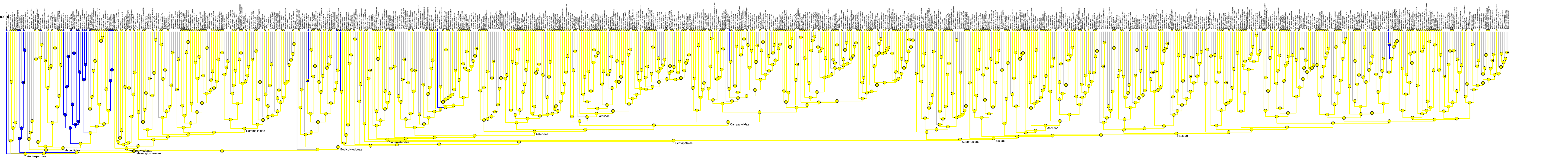

Model	LogL	Npar	AIC	AICc	DeltaAICc	w	q01	q10
ARD	−60.11	2	124.23	124.24	2.01	0.13	4e−04	0
ARDeq	−59.42	2	122.84	122.86	0.62	0.25	4e−04	0
ER	−60.36	1	122.73	122.73	0.5	0.27	4e−04	4e−04
UNI01*	−60.11	1	122.23	122.23	0	0.35	4e−04	
UNI10	−76.39	1	154.79	154.79	32.56	0		0.0209

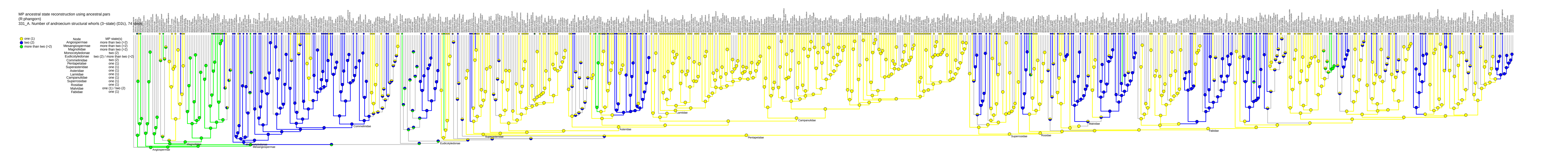
MP ancestral state reconstruction using ancestral.pars
(R:phangorn)
331_A. Number of androecium structural whorls (3−state) (D2c), 74 steps
one (1)
two (2)
more than two (>2)
Node | MP state(s)
Angiospermae | more than two (>2)
Mesangiospermae | more than two (>2)
Magnoliidae | more than two (>2)
Monocotyledonae | two (2)
Eudicotyledonae | two (2) / more than two (>2)
Commelinidae | two (2)
Pentapetalae | one (1)
Superasteridae | one (1)
Asteridae | one (1)
Lamiidae | one (1)
Campanulidae | one (1)
Superrosidae | one (1)
Rosidae | one (1)
Malvidae | one (1) / two (2)
Fabidae | one (1)
Angiospermae
Magnoliidae
Mesangiospermae
Commelinidae
Eudicotyledonae
Superasteridae
Asteridae
Lamiidae
Campanulidae
Pentapetalae
Superrosidae
Rosidae
Malvidae
Fabidae

ML ancestral state reconstruction using rayDISC (R:corHMM)
331_A. Number of androecium structural whorls (3−state) (D2c), ORDeq model

- one (1)
- two (2)
- more than two (>2)

Node	ML state	Prob
Angiospermae	two (2)	1
Mesangiospermae	two (2)	0.9999
Magnoliidae	two (2)	0.9917
Monocotyledonae	two (2)	1
Eudicotyledonae	two (2)	1
Commelinidae	two (2)	1
Pentapetalae	two (2)	1
Superasteridae	two (2)	1
Asteridae	two (2)	1
Lamiidae	one (1)	0.9986
Campanulidae	one (1)	0.9916
Superrosidae	two (2)	1
Rosidae	two (2)	1
Malvidae	two (2)	1
Fabidae	two (2)	1

Model	LogL	Npar	AIC	AICc	DeltaAICc	w	q01	⋯
ARD	−230.47	6	472.95	473.06	0.19	0.32	0	⋯
ARDeq	−230.91	6	473.82	473.92	1.06	0.21	0	⋯
ER	−280.24	1	562.49	562.49	89.63	0	0.0017	⋯
SYM	−254.21	3	514.43	514.46	41.59	0	0.0035	⋯
SYMeq	−253.16	3	512.33	512.36	39.49	0	0.0035	⋯
ORD	−233.46	4	474.93	474.98	2.11	0.12	0	⋯
ORDeq*	−232.41	4	472.82	472.87	0	0.35	0	⋯
ORDSYM	−255.12	2	514.24	514.26	41.39	0	0.0036	⋯
ORDSYMeq	−254.05	2	512.09	512.11	39.24	0	0.0036	⋯
ORDER	−260.45	1	522.9	522.9	50.04	0	0.0029	⋯

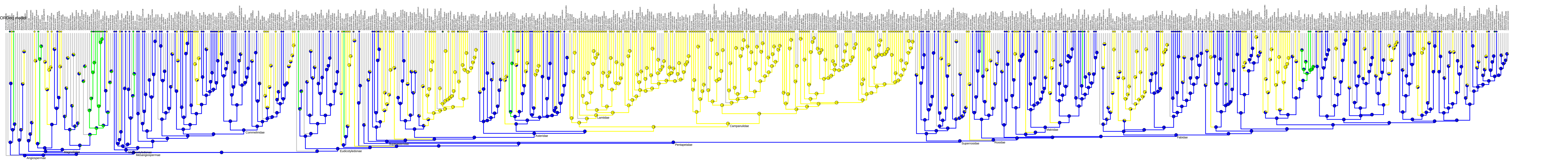

MP ancestral state reconstruction using ancestral.pars
(R:phangorn)
332_A. Androecium structural merism (4−state) (D2c), 58 steps

- dimerous
- trimerous
- tetramerous
- pentamerous

Node	MP state(s)
Angiospermae	trimerous
Mesangiospermae	trimerous
Magnoliidae	trimerous
Monocotyledonae	trimerous
Eudicotyledonae	trimerous / tetramerous / pentamerous
Commelinidae	trimerous
Pentapetalae	pentamerous
Superasteridae	pentamerous
Asteridae	pentamerous
Lamiidae	pentamerous
Campanulidae	pentamerous
Superrosidae	pentamerous
Rosidae	pentamerous
Malvidae	pentamerous
Fabidae	pentamerous

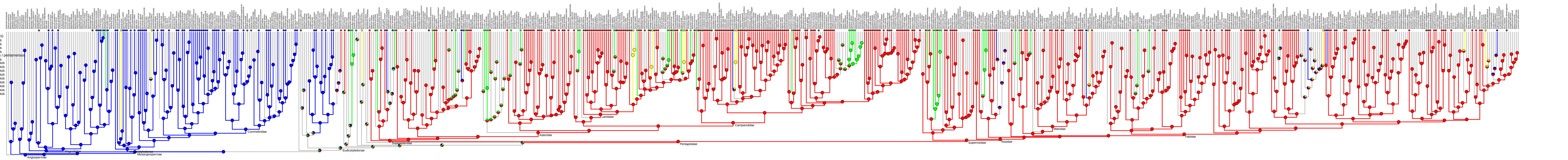

ML ancestral state reconstruction using rayDISC (R:corHMM)
332_A. Androecium structural merism (4−state) (D2c), ORDeq model

- dimerous
- trimerous
- tetramerous
- pentamerous

Node	ML state	Prob
Angiospermae	tetramerous	0.9846
Mesangiospermae	tetramerous	0.9215
Magnoliidae	tetramerous	0.8424
Monocotyledonae	trimerous	0.5155
Eudicotyledonae	tetramerous	0.9628
Commelinidae	trimerous	0.8489
Pentapetalae	pentamerous	0.9977
Superasteridae	pentamerous	0.9991
Asteridae	pentamerous	0.9998
Lamiidae	pentamerous	0.9999
Campanulidae	pentamerous	1
Superrosidae	pentamerous	0.9999
Rosidae	pentamerous	0.9999
Malvidae	pentamerous	0.9999
Fabidae	pentamerous	1

Model	LogL	Npar	AIC	AICc	DeltaAICc	w	q01	···
ARD	−222.8	12	469.59	469.99	348.76	0	0.0071	···
ARDeq	−221.46	12	466.92	467.32	346.09	0	0.0069	···
ER	−236.77	1	475.53	475.54	354.3	0	9e−04	···
SYM	−224.73	6	461.47	461.58	340.34	0	6e−04	···
SYMeq	−223.36	6	458.72	458.82	337.59	0	6e−04	···
ORD	−56.67	6	125.34	125.44	4.21	0.11	99.9998	···
ORDeq**	−54.56	6	121.13	121.24	0	0.89	100	···
ORDSYM	−256.73	3	519.46	519.49	398.26	0	0.0021	···
ORDSYMeq	−255.4	3	516.8	516.83	395.59	0	0.0021	···
ORDER	−257.49	1	516.97	516.98	395.74	0	0.0031	···

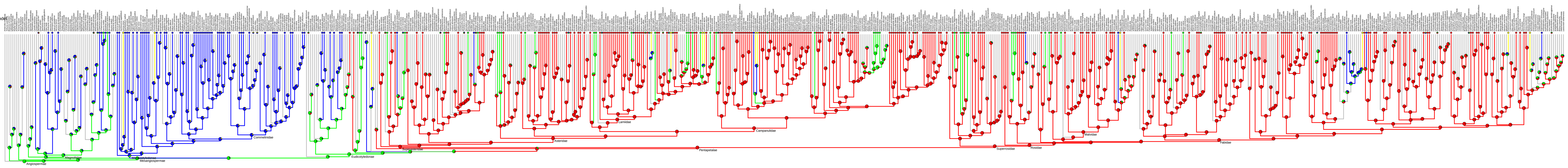

MP ancestral state reconstruction using ancestral.pars
(R:phangorn)
332_B. Androecium structural merism (3−state) (D2c), 49 steps

- trimerous
- tetramerous
- pentamerous

Node	MP state(s)
Angiospermae	trimerous
Mesangiospermae	trimerous
Magnoliidae	trimerous
Monocotyledonae	trimerous
Eudicotyledonae	trimerous / tetramerous / pentamerous
Commelinidae	trimerous
Pentapetalae	pentamerous
Superasteridae	pentamerous
Asteridae	pentamerous
Lamiidae	pentamerous
Campanulidae	pentamerous
Superrosidae	pentamerous
Rosidae	pentamerous
Malvidae	pentamerous
Fabidae	pentamerous

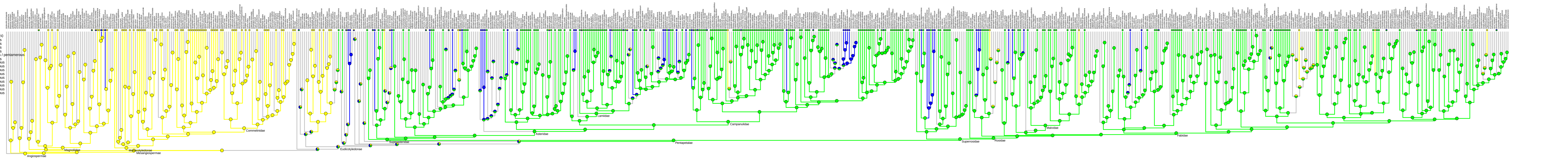

ML ancestral state reconstruction using rayDISC (R:corHMM)
332_B. Androecium structural merism (3−state) (D2c), SYMeq model

- trimerous
- tetramerous
- pentamerous

Node	ML state	Prob
Angiospermae	trimerous	1
Mesangiospermae	trimerous	1
Magnoliidae	trimerous	1
Monocotyledonae	trimerous	1
Eudicotyledonae	trimerous	0.8422
Commelinidae	trimerous	1
Pentapetalae	pentamerous	0.9999
Superasteridae	pentamerous	1
Asteridae	pentamerous	0.9996
Lamiidae	pentamerous	0.9999
Campanulidae	pentamerous	1
Superrosidae	pentamerous	1
Rosidae	pentamerous	1
Malvidae	pentamerous	1
Fabidae	pentamerous	1

Model	LogL	Npar	AIC	AICc	DeltaAICc	w	q01	···
ARD	−176.73	6	365.46	365.57	7.87	0.01	5e−04	···
ARDeq	−175.64	6	363.28	363.38	5.69	0.04	5e−04	···
ER	−185.95	1	373.9	373.91	16.21	0	0.0011	···
SYM	−176.92	3	359.85	359.88	2.18	0.22	5e−04	···
SYMeq**	−175.83	3	357.66	357.69	0	0.65	5e−04	···
ORD	−178.34	4	364.69	364.74	7.05	0.02	1e−04	···
ORDeq	−177.27	4	362.54	362.59	4.9	0.06	1e−04	···
ORDSYM	−185.89	2	375.78	375.79	18.1	0	0.002	···
ORDSYMeq	−184.81	2	373.62	373.64	15.94	0	0.002	···
ORDER	−186.63	1	375.25	375.26	17.56	0	0.0027	···

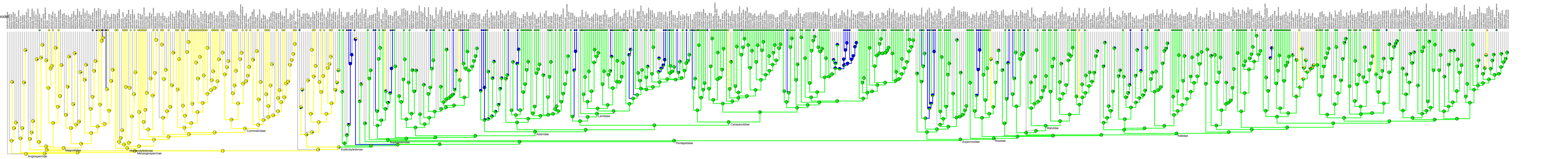

MP ancestral state reconstruction using ancestral.pars
(R:phangorn)
305_A. Filament (binary) (D2d), 52 steps

- laminar (wide)
- typical (narrow)

Node	MP state(s)
Angiospermae	laminar (wide)
Mesangiospermae	laminar (wide)
Magnoliidae	laminar (wide)
Monocotyledonae	typical (narrow)
Eudicotyledonae	typical (narrow)
Commelinidae	typical (narrow)
Pentapetalae	typical (narrow)
Superasteridae	typical (narrow)
Asteridae	typical (narrow)
Lamiidae	typical (narrow)
Campanulidae	typical (narrow)
Superrosidae	typical (narrow)
Rosidae	typical (narrow)
Malvidae	typical (narrow)
Fabidae	typical (narrow)

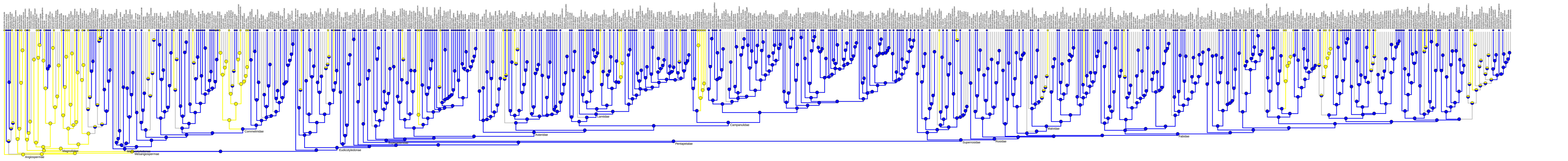

ML ancestral state reconstruction using rayDISC (R:corHMM)
305_A. Filament (binary) (D2d), ARDeq model

- laminar (wide)
- typical (narrow)

Node	ML state	Prob
Angiospermae	laminar (wide)	0.9999
Mesangiospermae	laminar (wide)	0.9854
Magnoliidae	laminar (wide)	0.9976
Monocotyledonae	laminar (wide)	0.564
Eudicotyledonae	typical (narrow)	0.7744
Commelinidae	typical (narrow)	0.6606
Pentapetalae	typical (narrow)	0.9996
Superasteridae	typical (narrow)	0.9997
Asteridae	typical (narrow)	0.9998
Lamiidae	typical (narrow)	0.9985
Campanulidae	typical (narrow)	0.9952
Superrosidae	typical (narrow)	0.9998
Rosidae	typical (narrow)	0.9998
Malvidae	typical (narrow)	0.9979
Fabidae	typical (narrow)	1

Model	LogL	Npar	AIC	AICc	DeltaAICc	w	q01	q10
ARD	−172.12	2	348.25	348.26	1.2	0.35	0.008	0.0021
ARDeq**	−171.52	2	347.04	347.06	0	0.64	0.0082	0.0021
ER	−178.09	1	358.18	358.19	11.13	0	0.0024	0.0024
UNI01	−183.16	1	368.33	368.33	21.27	0	0.0147	
UNI10	−188.67	1	379.34	379.35	32.29	0		0.0023

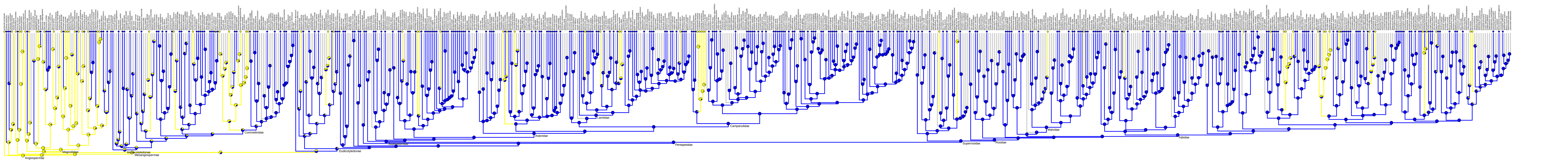

MP ancestral state reconstruction using ancestral.pars
(R:phangorn)
311_A. Anther orientation (D2d), 108 steps

- introrse
- latrorse
- extrorse

Node	MP state(s)
Angiospermae	introrse
Mesangiospermae	introrse / extrorse
Magnoliidae	extrorse
Monocotyledonae	introrse
Eudicotyledonae	introrse / extrorse
Commelinidae	introrse
Pentapetalae	introrse
Superasteridae	introrse
Asteridae	introrse
Lamiidae	introrse
Campanulidae	introrse
Superrosidae	introrse
Rosidae	introrse
Malvidae	introrse
Fabidae	introrse

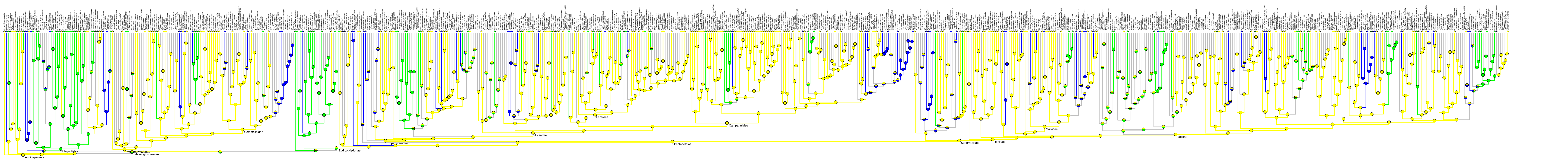

ML ancestral state reconstruction using rayDISC (R:corHMM)
311_A. Anther orientation (D2d), SYMeq model

- introrse
- latrorse
- extrorse

Node	ML state	Prob
Angiospermae	introrse	1
Mesangiospermae	introrse	0.998
Magnoliidae	extrorse	0.8122
Monocotyledonae	introrse	0.9985
Eudicotyledonae	introrse	0.9967
Commelinidae	introrse	0.9954
Pentapetalae	introrse	1
Superasteridae	introrse	0.9999
Asteridae	introrse	0.9997
Lamiidae	introrse	0.9998
Campanulidae	introrse	1
Superrosidae	introrse	1
Rosidae	introrse	1
Malvidae	introrse	0.9999
Fabidae	introrse	0.9998

Model	LogL	Npar	AIC	AICc	DeltaAICc	w	q01	···
ARD	−345.69	6	703.38	703.48	4.34	0.04	0.0036	···
ARDeq	−344.69	6	701.39	701.49	2.35	0.12	0.0036	···
ER	−348.69	1	699.39	699.39	0.25	0.33	0.0027	···
SYM	−347.61	3	701.22	701.25	2.1	0.13	0.003	···
SYMeq*	−346.56	3	699.11	699.14	0	0.38	0.003	···

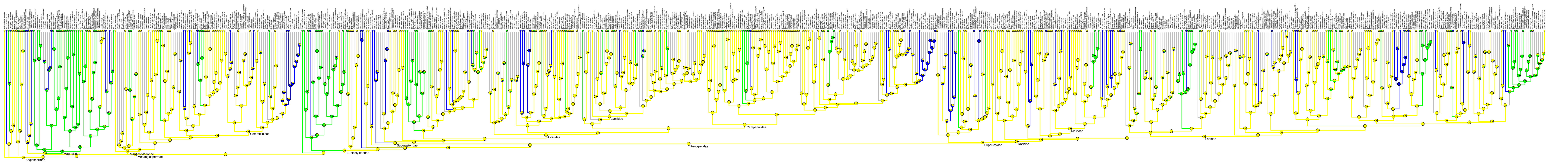

MP ancestral state reconstruction using ancestral.pars
(R:phangorn)
312_A. Anther attachment (binary) (D2d), 97 steps

- basifixed
- dorsifixed

Node	MP state(s)
Angiospermae	basifixed
Mesangiospermae	basifixed
Magnoliidae	basifixed
Monocotyledonae	basifixed
Eudicotyledonae	basifixed
Commelinidae	basifixed
Pentapetalae	basifixed
Superasteridae	basifixed
Asteridae	basifixed
Lamiidae	basifixed
Campanulidae	basifixed / dorsifixed
Superrosidae	basifixed
Rosidae	dorsifixed
Malvidae	dorsifixed
Fabidae	dorsifixed

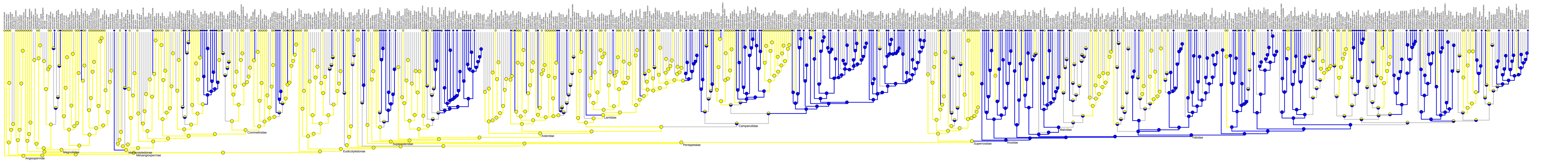

ML ancestral state reconstruction using rayDISC (R:corHMM)
312_A. Anther attachment (binary) (D2d), ARDeq model

- basifixed (yellow)
- dorsifixed (blue)

Node	ML state	Prob
Angiospermae	basifixed	1
Mesangiospermae	basifixed	0.9991
Magnoliidae	basifixed	0.9989
Monocotyledonae	basifixed	0.9932
Eudicotyledonae	basifixed	0.8303
Commelinidae	basifixed	0.9823
Pentapetalae	dorsifixed	0.892
Superasteridae	dorsifixed	0.8979
Asteridae	dorsifixed	0.8989
Lamiidae	dorsifixed	0.6781
Campanulidae	dorsifixed	0.9702
Superrosidae	dorsifixed	0.9017
Rosidae	dorsifixed	0.9483
Malvidae	dorsifixed	0.9799
Fabidae	dorsifixed	0.9998

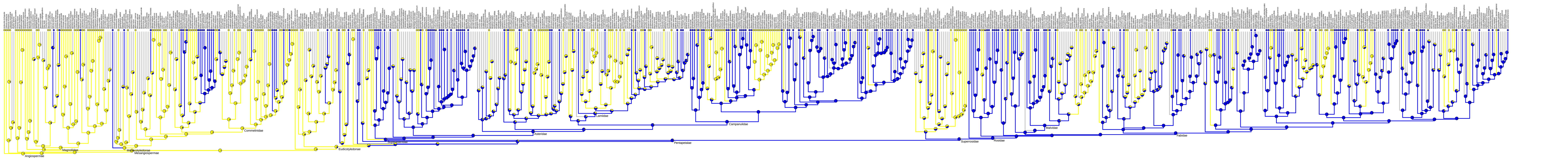

Model	LogL	Npar	AIC	AICc	DeltaAICc	w	q01	q10
ARD	−265.57	2	535.13	535.15	1.32	0.29	0.0043	0.0077
ARDeq**	−264.9	2	533.81	533.82	0	0.57	0.0043	0.0076
ER	−267.34	1	536.68	536.69	2.86	0.14	0.0056	0.0056
UNI01	−293.27	1	588.54	588.55	54.73	0	0.0061	
UNI10	−276.87	1	555.75	555.75	21.93	0		0.0081

MP ancestral state reconstruction using ancestral.pars
(R:phangorn)
313_A. Anther dehiscence (3−state) (D2d), 10 steps

- longitudinal slit
- H−valvate
- flap−valvate

Node	MP state(s)
Angiospermae	longitudinal slit
Mesangiospermae	longitudinal slit
Magnoliidae	longitudinal slit
Monocotyledonae	longitudinal slit
Eudicotyledonae	longitudinal slit
Commelinidae	longitudinal slit
Pentapetalae	longitudinal slit
Superasteridae	longitudinal slit
Asteridae	longitudinal slit
Lamiidae	longitudinal slit
Campanulidae	longitudinal slit
Superrosidae	longitudinal slit
Rosidae	longitudinal slit
Malvidae	longitudinal slit
Fabidae	longitudinal slit

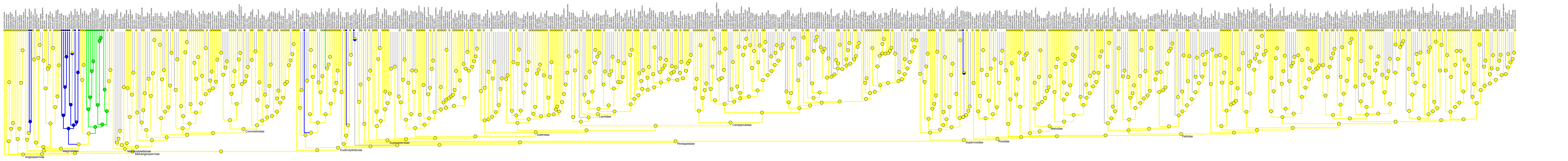

ML ancestral state reconstruction using rayDISC (R:corHMM)
313_A. Anther dehiscence (3−state) (D2d), ARDeq model

- longitudinal slit (yellow)
- H−valvate (blue)
- flap−valvate (green)

Node	ML state	Prob
Angiospermae	longitudinal slit	1
Mesangiospermae	longitudinal slit	1
Magnoliidae	longitudinal slit	1
Monocotyledonae	longitudinal slit	1
Eudicotyledonae	longitudinal slit	1
Commelinidae	longitudinal slit	1
Pentapetalae	longitudinal slit	1
Superasteridae	longitudinal slit	1
Asteridae	longitudinal slit	1
Lamiidae	longitudinal slit	1
Campanulidae	longitudinal slit	1
Superrosidae	longitudinal slit	1
Rosidae	longitudinal slit	1
Malvidae	longitudinal slit	1
Fabidae	longitudinal slit	1

Model	LogL	Npar	AIC	AICc	DeltaAICc	w	q01	···
ARD	−56.51	6	125.03	125.13	2.2	0.11	2e−04	···
ARDeq*	−55.41	6	122.83	122.94	0	0.32	2e−04	···
ER	−60.8	1	123.6	123.61	0.67	0.23	2e−04	···
SYM	−59.75	3	125.51	125.54	2.6	0.09	2e−04	···
SYMeq	−58.65	3	123.31	123.34	0.4	0.26	2e−04	···

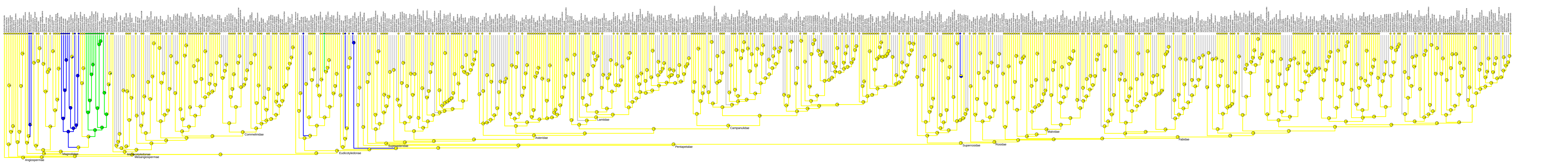

MP ancestral state reconstruction using ancestral.pars (R:phangorn)
401_B. Number of structural carpels (5−state) (D2c), 188 steps

- one (1)
- two (2)
- three (3)
- four or five (4−5)
- more than five (>5)

Node	MP state(s)
Angiospermae	more than five (>5)
Mesangiospermae	more than five (>5)
Magnoliidae	more than five (>5)
Monocotyledonae	three (3)
Eudicotyledonae	more than five (>5)
Commelinidae	three (3)
Pentapetalae	four or five (4−5)
Superasteridae	four or five (4−5)
Asteridae	two (2) / four or five (4−5)
Lamiidae	two (2)
Campanulidae	two (2)
Superrosidae	four or five (4−5)
Rosidae	four or five (4−5)
Malvidae	four or five (4−5)
Fabidae	four or five (4−5)

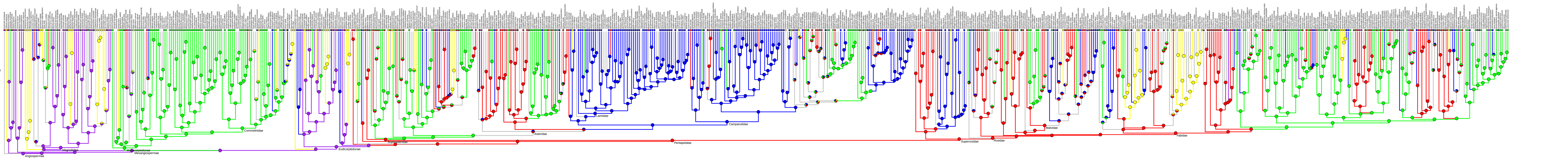

ML ancestral state reconstruction using rayDISC (R:corHMM)
401_B. Number of structural carpels (5−state) (D2c), ARDeq model

- one (1)
- two (2)
- three (3)
- four or five (4−5)
- more than five (>5)

Node	ML state	Prob
Angiospermae	more than five (>5)	1
Mesangiospermae	more than five (>5)	1
Magnoliidae	more than five (>5)	1
Monocotyledonae	more than five (>5)	0.9133
Eudicotyledonae	more than five (>5)	0.9984
Commelinidae	three (3)	1
Pentapetalae	three (3)	0.9997
Superasteridae	three (3)	0.9999
Asteridae	three (3)	0.9958
Lamiidae	two (2)	0.9986
Campanulidae	two (2)	0.9833
Superrosidae	three (3)	0.9999
Rosidae	three (3)	0.9999
Malvidae	three (3)	0.9989
Fabidae	three (3)	1

Model	LogL	Npar	AIC	AICc	DeltaAICc	w	q01	...
ARD	−672.96	20	1385.91	1387	3.21	0.13	0.0018	...
ARDeq**	−671.35	20	1382.7	1383.79	0	0.63	0.0018	...
ER	−717.6	1	1437.21	1437.21	53.43	0	0.0016	...
SYM	−684.46	10	1388.93	1389.21	5.42	0.04	9e−04	...
SYMeq	−682.93	10	1385.86	1386.14	2.35	0.2	9e−04	...
ORD	−754.76	8	1525.53	1525.71	141.93	0	9e−04	...
ORDeq	−753.24	8	1522.48	1522.66	138.88	0	9e−04	...
ORDSYM	−759.21	4	1526.42	1526.47	142.68	0	0.0042	...
ORDSYMeq	−757.77	4	1523.53	1523.58	139.8	0	0.0042	...
ORDER	−764.51	1	1531.03	1531.03	147.25	0	0.0058	...

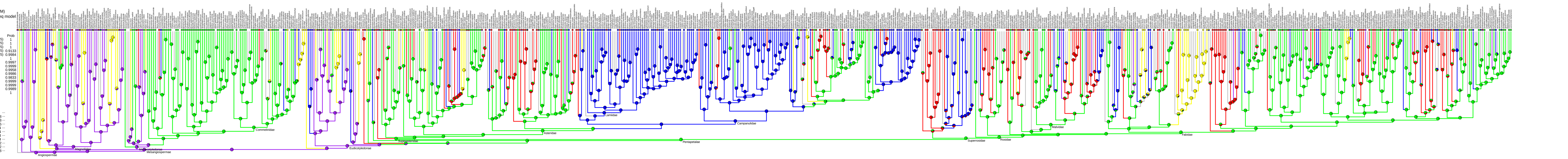

MP ancestral state reconstruction using ancestral.pars
(R:phangorn)
400_A. Gynoecium phyllotaxy (D2d), 10 steps

- whorled
- spiral

Node	MP state(s)
Angiospermae	whorled / spiral
Mesangiospermae	whorled / spiral
Magnoliidae	whorled / spiral
Monocotyledonae	whorled
Eudicotyledonae	whorled
Commelinidae	whorled
Pentapetalae	whorled
Superasteridae	whorled
Asteridae	whorled
Lamiidae	whorled
Campanulidae	whorled
Superrosidae	whorled
Rosidae	whorled
Malvidae	whorled
Fabidae	whorled

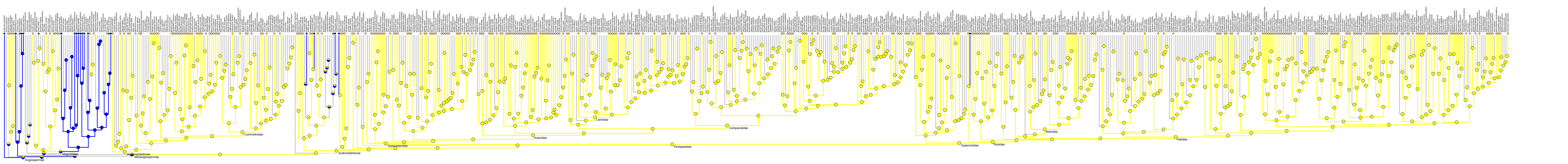

ML ancestral state reconstruction using rayDISC (R:corHMM)
400_A. Gynoecium phyllotaxy (D2d), ARDeq model

- whorled
- spiral

Node	ML state	Prob
Angiospermae	spiral	1
Mesangiospermae	spiral	1
Magnoliidae	spiral	1
Monocotyledonae	spiral	0.5045
Eudicotyledonae	spiral	0.9994
Commelinidae	whorled	0.9998
Pentapetalae	whorled	0.9763
Superasteridae	whorled	0.9876
Asteridae	whorled	1
Lamiidae	whorled	1
Campanulidae	whorled	1
Superrosidae	whorled	0.9795
Rosidae	whorled	0.9796
Malvidae	whorled	1
Fabidae	whorled	1

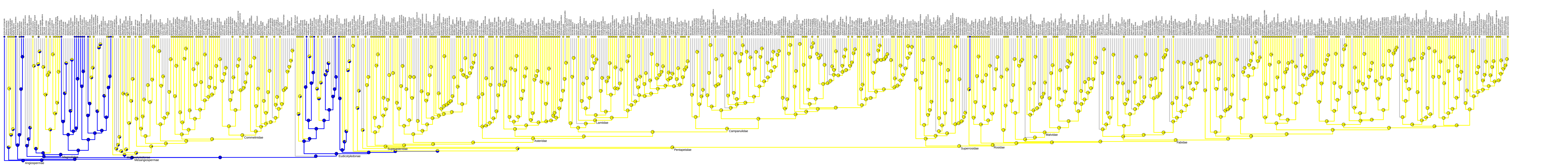

Model	LogL	Npar	AIC	AICc	DeltaAICc	w	q01	q10
ARD	−39.93	2	83.86	83.87	1.39	0.31	1e−04	0.0084
ARDeq**	−39.24	2	82.47	82.49	0	0.63	1e−04	0.0084
ER	−51.55	1	105.1	105.1	22.61	0	4e−04	4e−04
UNI01	−53.06	1	108.13	108.13	25.64	0	4e−04	
UNI10	−42.6	1	87.21	87.21	4.73	0.06		0.0098

MP ancestral state reconstruction using ancestral.pars
(R:phangorn)
403_A. Fusion of ovaries (binary) (D2c), 23 steps

- free (<5%)
- fused (>5%)

Node	MP state(s)
Angiospermae	free (<5%)
Mesangiospermae	free (<5%) / fused (>5%)
Magnoliidae	free (<5%) / fused (>5%)
Monocotyledonae	fused (>5%)
Eudicotyledonae	fused (>5%)
Commelinidae	fused (>5%)
Pentapetalae	fused (>5%)
Superasteridae	fused (>5%)
Asteridae	fused (>5%)
Lamiidae	fused (>5%)
Campanulidae	fused (>5%)
Superrosidae	fused (>5%)
Rosidae	fused (>5%)
Malvidae	fused (>5%)
Fabidae	fused (>5%)

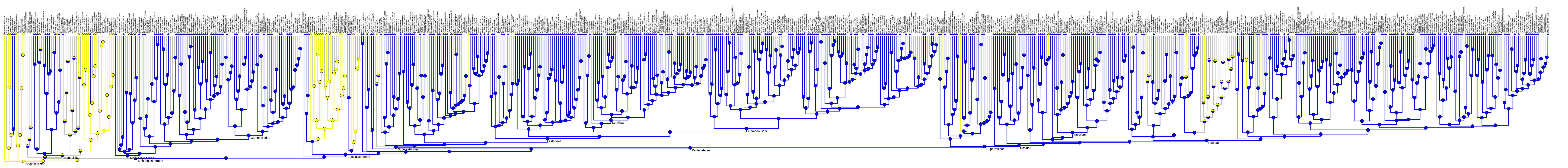

ML ancestral state reconstruction using rayDISC (R:corHMM)
403_A. Fusion of ovaries (binary) (D2c), ARD model

- free (<5%)
- fused (>5%)

Node	ML state	Prob
Angiospermae	free (<5%)	1
Mesangiospermae	free (<5%)	1
Magnoliidae	free (<5%)	0.9999
Monocotyledonae	free (<5%)	0.9461
Eudicotyledonae	free (<5%)	0.9989
Commelinidae	fused (>5%)	1
Pentapetalae	fused (>5%)	0.9951
Superasteridae	fused (>5%)	0.9953
Asteridae	fused (>5%)	1
Lamiidae	fused (>5%)	1
Campanulidae	fused (>5%)	1
Superrosidae	fused (>5%)	0.9995
Rosidae	fused (>5%)	0.9998
Malvidae	fused (>5%)	1
Fabidae	fused (>5%)	0.9998

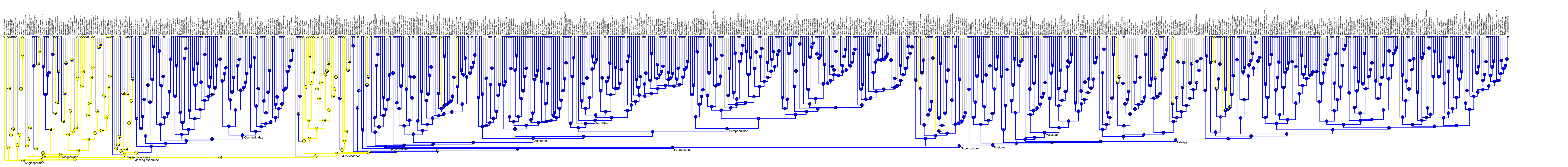

Model	LogL	Npar	AIC	AICc	DeltaAICc	w	q01	q10
ARD***	−94.69	2	193.37	193.39	0	0.95	0.0053	5e−04
ARDeq	−102.29	2	208.58	208.6	15.21	0	6e−04	8e−04
ER	−102.68	1	207.36	207.36	13.97	0	8e−04	8e−04
UNI01	−98.68	1	199.36	199.37	5.98	0.05	0.0113	
UNI10	−106.84	1	215.69	215.69	22.31	0		9e−04

MP ancestral state reconstruction using ancestral.pars
(R:phangorn)
411_A. Number of ovules per functional carpel (3−state) (D2c), 120 steps

- one (1)
- two (2)
- three or more (>2)

Node	MP state(s)
Angiospermae	one (1)
Mesangiospermae	one (1)
Magnoliidae	one (1)
Monocotyledonae	one (1) / three or more (>2)
Eudicotyledonae	three or more (>2)
Commelinidae	one (1)
Pentapetalae	three or more (>2)
Superasteridae	three or more (>2)
Asteridae	one (1)
Lamiidae	one (1) / two (2) / three or more (>2)
Campanulidae	one (1)
Superrosidae	two (2) / three or more (>2)
Rosidae	two (2)
Malvidae	two (2) / three or more (>2)
Fabidae	two (2)

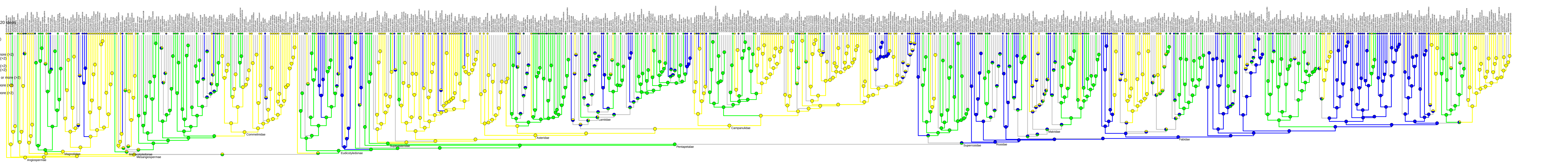

ML ancestral state reconstruction using rayDISC (R:corHMM)
411_A. Number of ovules per functional carpel (3−state) (D2c), ARDeq model

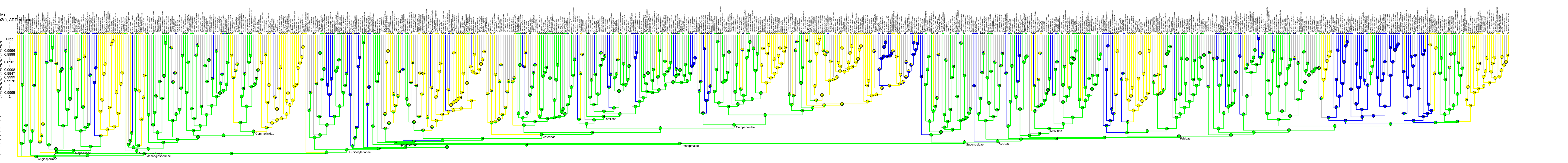

- one (1)
- two (2)
- three or more (>2)

Node	ML state	Prob
Angiospermae	three or more (>2)	1
Mesangiospermae	three or more (>2)	1
Magnoliidae	three or more (>2)	0.9996
Monocotyledonae	three or more (>2)	0.9999
Eudicotyledonae	three or more (>2)	1
Commelinidae	three or more (>2)	0.8901
Pentapetalae	three or more (>2)	1
Superasteridae	three or more (>2)	0.9998
Asteridae	three or more (>2)	0.9947
Lamiidae	three or more (>2)	0.9999
Campanulidae	three or more (>2)	0.9978
Superrosidae	three or more (>2)	1
Rosidae	three or more (>2)	1
Malvidae	three or more (>2)	0.9995
Fabidae	three or more (>2)	1

Model	LogL	Npar	AIC	AICc	DeltaAICc	w	q01	⋯
ARD	−388.52	6	789.04	789.15	2.18	0.25	0.0013	⋯
ARDeq**	−387.43	6	786.86	786.97	0	0.75	0.0013	⋯
ER	−402.57	1	807.14	807.15	20.18	0	0.0029	⋯
SYM	−402.09	3	810.19	810.22	23.25	0	0.0026	⋯
SYMeq	−401.15	3	808.3	808.33	21.36	0	0.0026	⋯
ORD	−399.49	4	806.99	807.04	20.07	0	0.0019	⋯
ORDeq	−398.41	4	804.82	804.87	17.9	0	0.0019	⋯
ORDSYM	−412.7	2	829.4	829.42	42.45	0	0.0055	⋯
ORDSYMeq	−411.82	2	827.63	827.65	40.68	0	0.0055	⋯
ORDER	−413.67	1	829.34	829.34	42.38	0	0.0063	⋯
