# Supplementary material for: The ancestral flower of angiosperms and its early diversification
Source: Nat Commun. 2017 Aug 1;8:16047. doi: 10.1038/ncomms16047 (PMC5543309; doi:10.1038/ncomms16047)

MP ancestral state reconstruction using ancestral.pars (R:phangorn)
100_A. Functional sex of flowers (D2d), 98 steps

- bisexual (yellow)
- unisexual (blue)

Node	MP state(s)
Angiospermae	bisexual / unisexual
Mesangiospermae	bisexual
Magnoliidae	bisexual
Monocotyledonae	bisexual
Eudicotyledonae	bisexual
Commelinidae	bisexual
Pentapetalae	bisexual
Superasteridae	bisexual
Asteridae	bisexual
Lamiidae	bisexual
Campanulidae	bisexual
Superrosidae	bisexual
Rosidae	bisexual
Malvidae	bisexual
Fabidae	bisexual

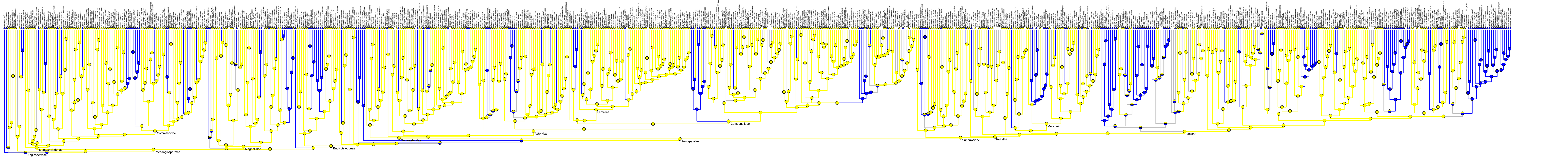

ML ancestral state reconstruction using rayDISC (R:corHMM)
100_A. Functional sex of flowers (D2d), ARDeq model

bisexual
unisexual

Node	ML state	Prob
Angiospermae	bisexual	1
Mesangiospermae	bisexual	1
Magnoliidae	bisexual	1
Monocotyledonae	bisexual	1
Eudicotyledonae	bisexual	1
Commelinidae	bisexual	1
Pentapetalae	bisexual	1
Superasteridae	bisexual	1
Asteridae	bisexual	1
Lamiidae	bisexual	1
Campanulidae	bisexual	0.9992
Superrosidae	bisexual	1
Rosidae	bisexual	1
Malvidae	bisexual	0.9997
Fabidae	bisexual	1

Model	LogL	Npar	AIC	AICc	DeltaAICc	w	q01	q10
ARD	−313.49	2	630.98	630.99	1.38	0.24	0.003	0.001
ARDeq*	−312.8	2	629.59	629.61	0	0.48	0.003	0.001
ER	−315.41	1	632.82	632.83	3.22	0.1	0.0029	0.0029
UNI01	−314.77	1	631.55	631.55	1.94	0.18	0.003	
UNI10	−352.85	1	707.7	707.71	78.1	0		0.012

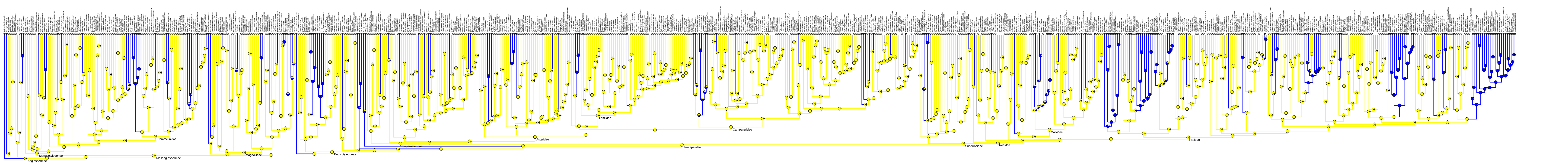

MP ancestral state reconstruction using ancestral.pars
(R:phangorn)
100_B. Structural sex of flowers (D2d), 57 steps

bisexual
unisexual

Node	MP state(s)
Angiospermae	bisexual
Mesangiospermae	bisexual
Magnoliidae	bisexual
Monocotyledonae	bisexual
Eudicotyledonae	bisexual
Commelinidae	bisexual
Pentapetalae	bisexual
Superasteridae	bisexual
Asteridae	bisexual
Lamiidae	bisexual
Campanulidae	bisexual
Superrosidae	bisexual
Rosidae	bisexual
Malvidae	bisexual
Fabidae	bisexual

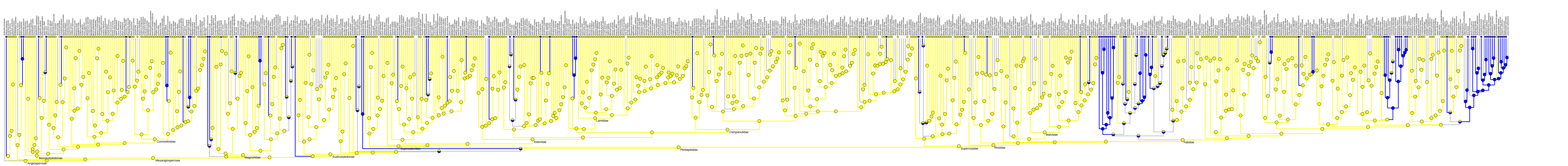

ML ancestral state reconstruction using rayDISC (R:corHMM)
100_B. Structural sex of flowers (D2d), ARDeq model

- bisexual (yellow)
- unisexual (blue)

Node	ML state	Prob
Angiospermae	bisexual	1
Mesangiospermae	bisexual	1
Magnoliidae	bisexual	1
Monocotyledonae	bisexual	1
Eudicotyledonae	bisexual	1
Commelinidae	bisexual	0.9999
Pentapetalae	bisexual	1
Superasteridae	bisexual	1
Asteridae	bisexual	1
Lamiidae	bisexual	0.9998
Campanulidae	bisexual	1
Superrosidae	bisexual	1
Rosidae	bisexual	1
Malvidae	bisexual	1
Fabidae	bisexual	0.9976

Model	LogL	Npar	AIC	AICc	DeltaAICc	w	q01	q10
ARD	−208.67	2	421.34	421.36	1.35	0.31	0.0015	0.0043
ARDeq**	−208	2	419.99	420.01	0	0.6	0.0015	0.0043
ER	−210.88	1	423.75	423.76	3.75	0.09	0.0016	0.0016
UNI01	−228.66	1	459.31	459.32	39.31	0	0.0017	
UNI10	−229.04	1	460.08	460.09	40.08	0		0.0167

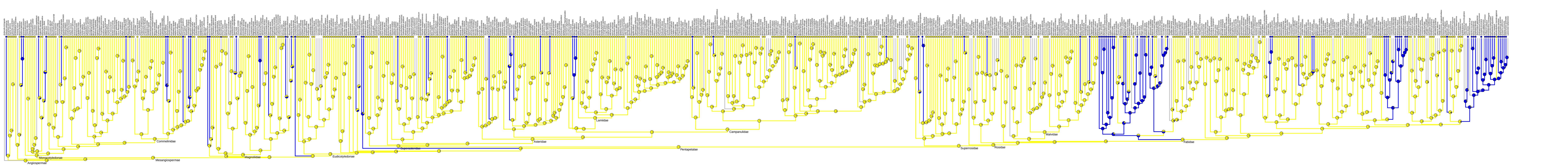

MP ancestral state reconstruction using ancestral.pars
(R:phangorn)
102_B. Ovary position (binary) (D2d), 79 steps

superior
inferior

Node	MP state(s)
Angiospermae	superior
Mesangiospermae	superior
Magnoliidae	superior
Monocotyledonae	superior
Eudicotyledonae	superior
Commelinidae	superior
Pentapetalae	superior
Superasteridae	superior
Asteridae	superior
Lamiidae	superior
Campanulidae	superior
Superrosidae	superior
Rosidae	superior / inferior
Malvidae	superior
Fabidae	superior

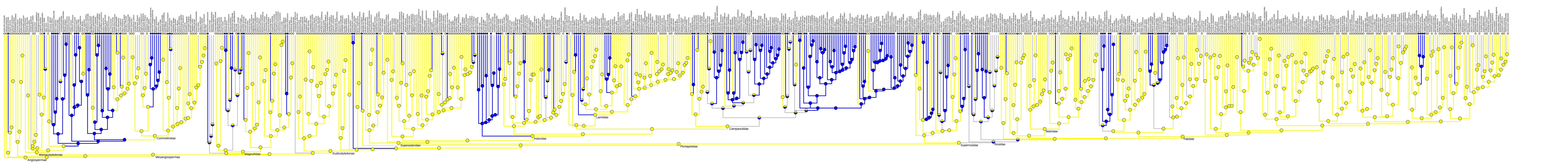

ML ancestral state reconstruction using rayDISC (R:corHMM)
102_B. Ovary position (binary) (D2d), ARDeq model

superior
inferior

Node	ML state	Prob
Angiospermae	superior	1
Mesangiospermae	superior	1
Magnoliidae	superior	0.9998
Monocotyledonae	superior	0.999
Eudicotyledonae	superior	1
Commelinidae	superior	0.8139
Pentapetalae	superior	0.9997
Superasteridae	superior	0.9999
Asteridae	superior	0.9789
Lamiidae	superior	0.9784
Campanulidae	superior	0.8397
Superrosidae	superior	0.9877
Rosidae	superior	0.9874
Malvidae	superior	1
Fabidae	superior	0.9999

Model	LogL	Npar	AIC	AICc	DeltaAICc	w	q01	q10
ARD	−270.92	2	545.84	545.85	1.38	0.22	0.0022	0.0034
ARDeq*	−270.23	2	544.45	544.47	0	0.45	0.0022	0.0034
ER	−271.53	1	545.06	545.07	0.6	0.33	0.0024	0.0024
UNI01	−280.08	1	562.17	562.17	17.7	0	0.0027	
UNI10	−294.52	1	591.04	591.05	46.58	0		0.0099

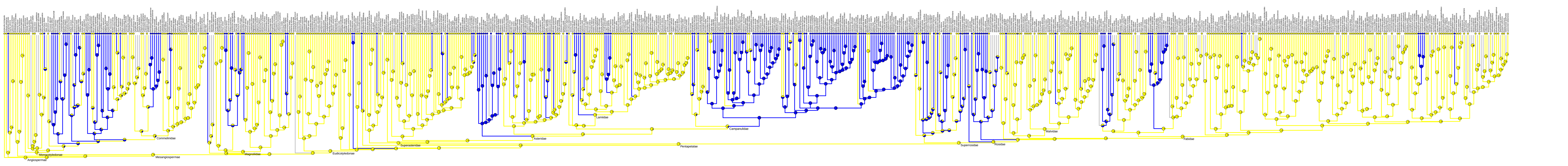

MP ancestral state reconstruction using ancestral.pars
(R:phangorn)
201_A. Perianth presence (D2c), 20 steps

Node	MP state(s)
Angiospermae	present
Mesangiospermae	present
Magnoliidae	present
Monocotyledonae	present
Eudicotyledonae	present
Commelinidae	present
Pentapetalae	present
Superasteridae	present
Asteridae	present
Lamiidae	present
Campanulidae	present
Superrosidae	present
Rosidae	present
Malvidae	present
Fabidae	present

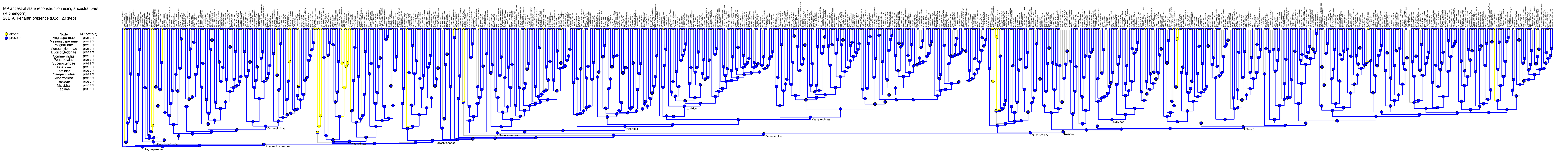

ML ancestral state reconstruction using rayDISC (R:corHMM)
201_A. Perianth presence (D2c), UNI10 model

- absent
- present

Node	ML state	Prob
Angiospermae	present	1
Mesangiospermae	present	1
Magnoliidae	present	1
Monocotyledonae	present	1
Eudicotyledonae	present	1
Commelinidae	present	1
Pentapetalae	present	1
Superasteridae	present	1
Asteridae	present	1
Lamiidae	present	1
Campanulidae	present	1
Superrosidae	present	1
Rosidae	present	1
Malvidae	present	1
Fabidae	present	1

Model	LogL	Npar	AIC	AICc	DeltaAICc	w	q01	q10
ARD	−96.06	2	196.11	196.13	2.01	0.13	0	5e−04
ARDeq	−95.36	2	194.72	194.74	0.62	0.26	0	5e−04
ER	−96.37	1	194.74	194.75	0.63	0.26	5e−04	5e−04
UNI01	−108.97	1	219.93	219.94	25.82	0	0.0222	
UNI10*	−96.06	1	194.11	194.12	0	0.35		5e−04

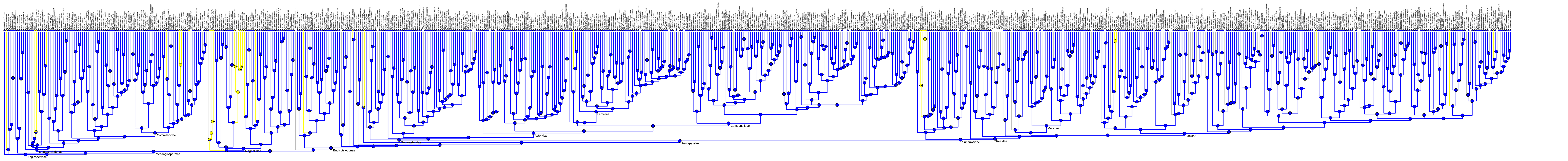

MP ancestral state reconstruction using ancestral.pars
(R:phangorn)
201_B. Number of perianth parts (3−state) (D2c), 86 steps

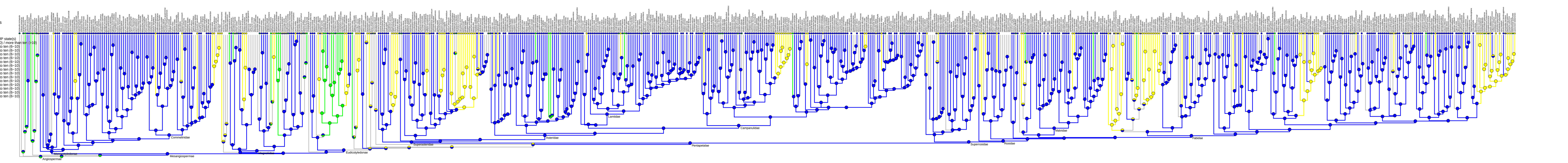

Node	MP state(s)
Angiospermae	six to ten (6−10) / more than ten (>10)
Mesangiospermae	six to ten (6−10)
Magnoliidae	six to ten (6−10)
Monocotyledonae	six to ten (6−10)
Eudicotyledonae	six to ten (6−10)
Commelinidae	six to ten (6−10)
Pentapetalae	six to ten (6−10)
Superasteridae	six to ten (6−10)
Asteridae	six to ten (6−10)
Lamiidae	six to ten (6−10)
Campanulidae	six to ten (6−10)
Superrosidae	six to ten (6−10)
Rosidae	six to ten (6−10)
Malvidae	six to ten (6−10)
Fabidae	six to ten (6−10)

ML ancestral state reconstruction using rayDISC (R:corHMM)
201_B. Number of perianth parts (3−state) (D2c), ARDeq model

- one to five (1−5)
- six to ten (6−10)
- more than ten (>10)

Node	ML state	Prob
Angiospermae	more than ten (>10)	1
Mesangiospermae	more than ten (>10)	0.9998
Magnoliidae	more than ten (>10)	0.9999
Monocotyledonae	six to ten (6−10)	0.5866
Eudicotyledonae	more than ten (>10)	0.998
Commelinidae	six to ten (6−10)	0.9999
Pentapetalae	six to ten (6−10)	0.9963
Superasteridae	six to ten (6−10)	0.9982
Asteridae	six to ten (6−10)	1
Lamiidae	six to ten (6−10)	0.9998
Campanulidae	six to ten (6−10)	1
Superrosidae	six to ten (6−10)	0.9992
Rosidae	six to ten (6−10)	0.9994
Malvidae	six to ten (6−10)	0.9998
Fabidae	six to ten (6−10)	0.9993

Model	LogL	Npar	AIC	AICc	DeltaAICc	w	q01	⋯
ARD	−324.73	6	661.47	661.57	2.2	0.25	0.004	⋯
ARDeq**	−323.64	6	659.27	659.38	0	0.74	0.004	⋯
ER	−349.9	1	701.8	701.81	42.43	0	0.0013	⋯
SYM	−339.09	3	684.18	684.21	24.83	0	0.002	⋯
SYMeq	−338.02	3	682.03	682.06	22.68	0	0.002	⋯
ORD	−331.17	4	670.33	670.39	11.01	0	0.0042	⋯
ORDeq	−330.07	4	668.14	668.19	8.81	0.01	0.0042	⋯
ORDSYM	−342.19	2	688.38	688.39	29.02	0	0.0021	⋯
ORDSYMeq	−341.13	2	686.25	686.27	26.89	0	0.0021	⋯
ORDER	−351.52	1	705.03	705.04	45.66	0	0.0014	⋯

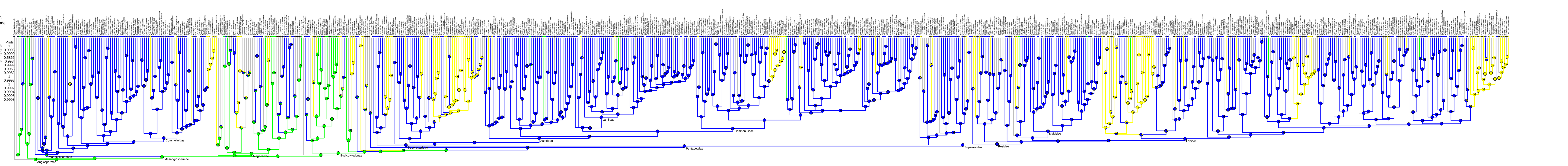

MP ancestral state reconstruction using ancestral.pars
(R:phangorn)
201_C. Number of perianth parts (binary) (D2c), 69 steps

- one to six (1−6)
- more than six (>6)

Node	MP state(s)
Angiospermae	more than six (>6)
Mesangiospermae	one to six (1−6)
Magnoliidae	one to six (1−6) / more than six (>6)
Monocotyledonae	one to six (1−6)
Eudicotyledonae	one to six (1−6)
Commelinidae	one to six (1−6)
Pentapetalae	more than six (>6)
Superasteridae	more than six (>6)
Asteridae	more than six (>6)
Lamiidae	more than six (>6)
Campanulidae	more than six (>6)
Superrosidae	more than six (>6)
Rosidae	more than six (>6)
Malvidae	more than six (>6)
Fabidae	more than six (>6)

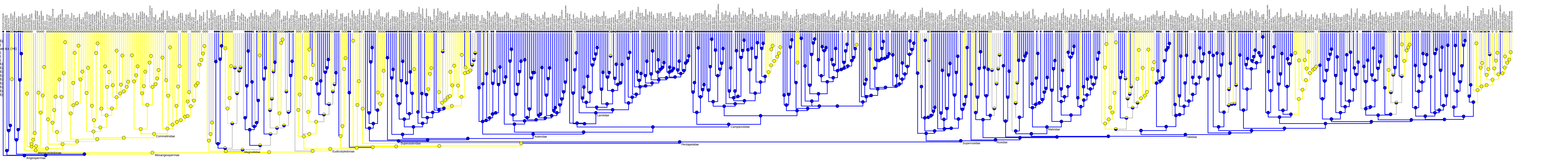

ML ancestral state reconstruction using rayDISC (R:corHMM)
201_C. Number of perianth parts (binary) (D2c), ARDeq model

one to six (1−6)
more than six (>6)

Node	ML state	Prob
Angiospermae	more than six (>6)	1
Mesangiospermae	more than six (>6)	0.9998
Magnoliidae	more than six (>6)	1
Monocotyledonae	one to six (1−6)	0.8013
Eudicotyledonae	more than six (>6)	1
Commelinidae	one to six (1−6)	1
Pentapetalae	more than six (>6)	1
Superasteridae	more than six (>6)	1
Asteridae	more than six (>6)	1
Lamiidae	more than six (>6)	1
Campanulidae	more than six (>6)	1
Superrosidae	more than six (>6)	1
Rosidae	more than six (>6)	1
Malvidae	more than six (>6)	1
Fabidae	more than six (>6)	1

Model	LogL	Npar	AIC	AICc	DeltaAICc	w	q01	q10
ARD	−247.89	2	499.77	499.79	1.39	0.32	6e−04	0.0026
ARDeq**	−247.19	2	498.39	498.4	0	0.65	6e−04	0.0026
ER	−254.48	1	510.95	510.96	12.56	0	0.0022	0.0022
UNI01	−323.21	1	648.42	648.43	150.03	0	0.0081	
UNI10	−251.44	1	504.87	504.88	6.48	0.03		0.0029

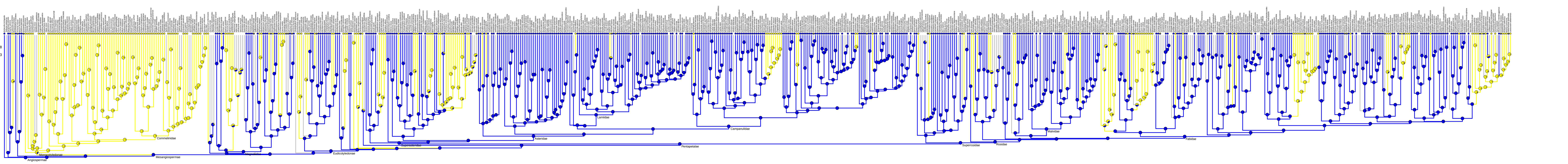

MP ancestral state reconstruction using ancestral.pars
(R:phangorn)
230_A. Perianth phyllotaxy (binary) (D2d), 16 steps

- whorled
- spiral

Node	MP state(s)
Angiospermae	whorled / spiral
Mesangiospermae	whorled
Magnoliidae	whorled
Monocotyledonae	whorled
Eudicotyledonae	whorled
Commelinidae	whorled
Pentapetalae	whorled
Superasteridae	whorled
Asteridae	whorled
Lamiidae	whorled
Campanulidae	whorled
Superrosidae	whorled
Rosidae	whorled
Malvidae	whorled
Fabidae	whorled

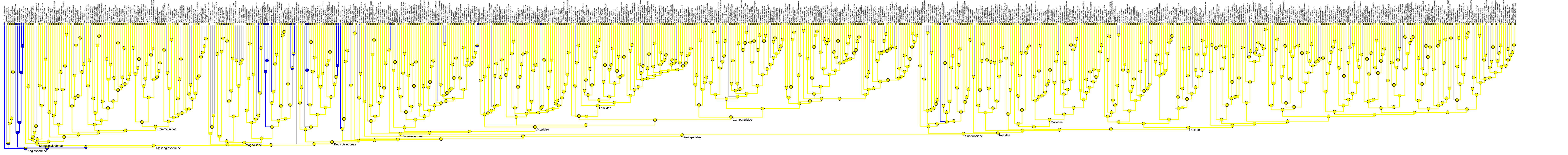

ML ancestral state reconstruction using rayDISC (R:corHMM)
230_A. Perianth phyllotaxy (binary) (D2d), ARD model

- whorled (yellow)
- spiral (blue)

Node	ML state	Prob
Angiospermae	spiral	1
Mesangiospermae	spiral	0.9999
Magnoliidae	spiral	0.9998
Monocotyledonae	spiral	0.5013
Eudicotyledonae	spiral	0.9997
Commelinidae	whorled	0.9996
Pentapetalae	whorled	0.9769
Superasteridae	whorled	0.9806
Asteridae	whorled	0.9995
Lamiidae	whorled	0.9999
Campanulidae	whorled	1
Superrosidae	whorled	0.9887
Rosidae	whorled	0.9926
Malvidae	whorled	1
Fabidae	whorled	1

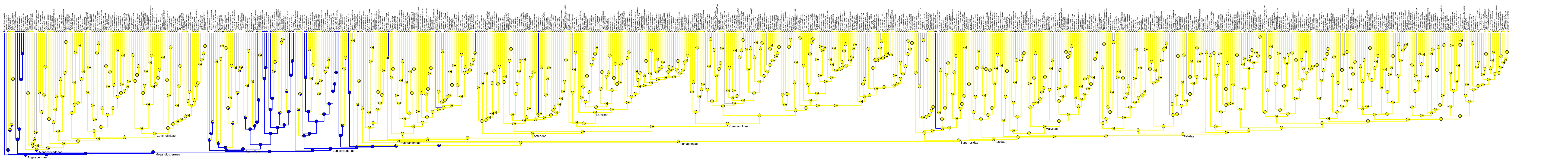

Model	LogL	Npar	AIC	AICc	DeltaAICc	w	q01	q10
ARD***	−70.65	2	145.29	145.31	0	0.99	2e−04	0.0113
ARDeq	−77.56	2	159.12	159.14	13.83	0	4e−04	8e−04
ER	−78.28	1	158.56	158.57	13.26	0	4e−04	4e−04
UNI01	−78.7	1	159.39	159.4	14.09	0	4e−04	
UNI10	−76.92	1	155.84	155.84	10.53	0.01		0.0185

MP ancestral state reconstruction using ancestral.pars
(R:phangorn)
231_A. Number of perianth whorls (D2c), 73 steps

- one (1)
- two (2)
- more than two (>2)

Node	MP state(s)
Angiospermae	more than two (>2)
Mesangiospermae	more than two (>2)
Magnoliidae	more than two (>2)
Monocotyledonae	two (2)
Eudicotyledonae	more than two (>2)
Commelinidae	two (2)
Pentapetalae	two (2)
Superasteridae	two (2)
Asteridae	two (2)
Lamiidae	two (2)
Campanulidae	two (2)
Superrosidae	two (2)
Rosidae	two (2)
Malvidae	two (2)
Fabidae	two (2)

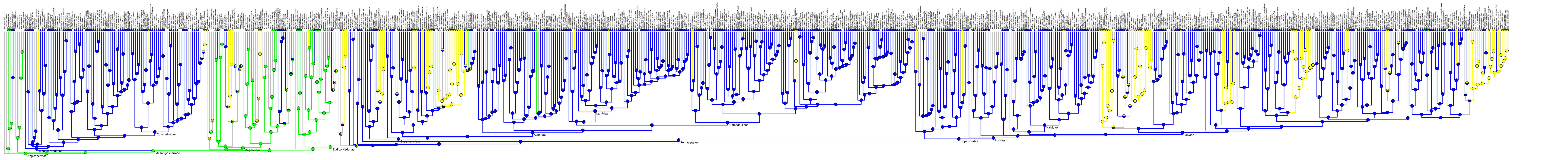

ML ancestral state reconstruction using rayDISC (R:corHMM)
231_A. Number of perianth whorls (D2c), ARDeq model

- one (1)
- two (2)
- more than two (>2)

Node	ML state	Prob
Angiospermae	more than two (>2)	1
Mesangiospermae	more than two (>2)	0.9999
Magnoliidae	more than two (>2)	1
Monocotyledonae	two (2)	0.724
Eudicotyledonae	more than two (>2)	0.9984
Commelinidae	two (2)	1
Pentapetalae	two (2)	0.9998
Superasteridae	two (2)	0.9998
Asteridae	two (2)	0.9999
Lamiidae	two (2)	0.9999
Campanulidae	two (2)	1
Superrosidae	two (2)	1
Rosidae	two (2)	1
Malvidae	two (2)	0.9999
Fabidae	two (2)	0.9976

Model	LogL	Npar	AIC	AICc	DeltaAICc	w	q01	...
ARD	−269.2	6	550.41	550.51	2.2	0.25	0.0048	...
ARDeq**	−268.1	6	548.21	548.32	0	0.75	0.0048	...
ER	−311.61	1	625.22	625.23	76.91	0	0.001	...
SYM	−293.84	3	593.67	593.7	45.39	0	0.0019	...
SYMeq	−292.85	3	591.69	591.72	43.4	0	0.0019	...
ORD	−277.03	4	562.06	562.11	13.79	0	0.0047	...
ORDeq	−275.93	4	559.86	559.91	11.59	0	0.0047	...
ORDSYM	−299.35	2	602.7	602.71	54.4	0	0.0019	...
ORDSYMeq	−298.28	2	600.56	600.58	52.26	0	0.0019	...
ORDER	−314.24	1	630.48	630.49	82.17	0	0.0012	...

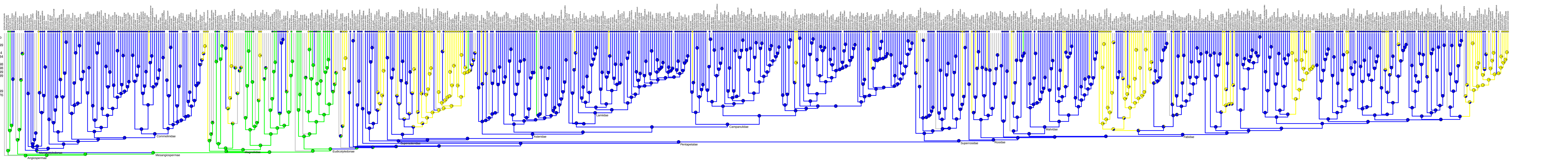

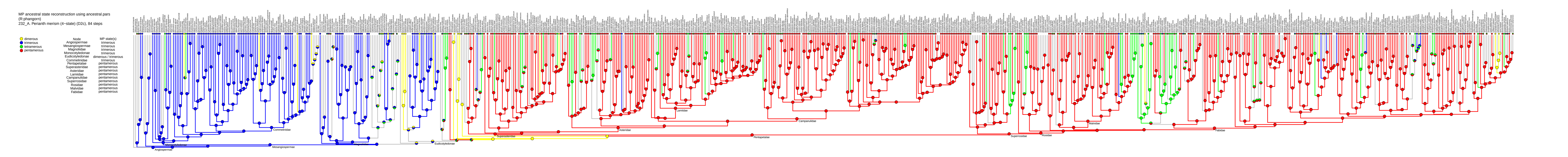
MP ancestral state reconstruction using ancestral.pars
(R:phangorn)
232_A. Perianth merism (4−state) (D2c), 84 steps
dimerous
trimerous
tetramerous
pentamerous
Node
MP state(s)
Angiospermae trimerous
Mesangiospermae trimerous
Magnoliidae trimerous
Monocotyledonae trimerous
Eudicotyledonae dimerous / trimerous
Commelinidae trimerous
Pentapetalae pentamerous
Superasteridae pentamerous
Asteridae pentamerous
Lamiidae pentamerous
Campanulidae pentamerous
Superrosidae pentamerous
Rosidae pentamerous
Malvidae pentamerous
Fabidae pentamerous

ML ancestral state reconstruction using rayDISC (R:corHMM)
232_A. Perianth merism (4−state) (D2c), SYMeq model

- dimerous
- trimerous
- tetramerous
- pentamerous

Node	ML state	Prob
Angiospermae	trimerous	1
Mesangiospermae	trimerous	1
Magnoliidae	trimerous	1
Monocotyledonae	trimerous	1
Eudicotyledonae	trimerous	0.9775
Commelinidae	trimerous	1
Pentapetalae	pentamerous	0.9997
Superasteridae	pentamerous	0.9999
Asteridae	pentamerous	1
Lamiidae	pentamerous	0.9999
Campanulidae	pentamerous	1
Superrosidae	pentamerous	1
Rosidae	pentamerous	1
Malvidae	pentamerous	1
Fabidae	pentamerous	1

Model	LogL	Npar	AIC	AICc	DeltaAICc	w	q01	···
ARD	−324.05	12	672.09	672.49	10.26	0	0	···
ARDeq	−322.68	12	669.36	669.76	7.53	0.02	0	···
ER	−360.93	1	723.86	723.87	61.64	0	8e−04	···
SYM	−326.44	6	664.89	664.99	2.76	0.2	0.0013	···
SYMeq**	−325.06	6	662.12	662.23	0	0.78	0.0013	···
ORD	−337.09	6	686.19	686.29	24.06	0	0	···
ORDeq	−335.78	6	683.56	683.66	21.43	0	0	···
ORDSYM	−345.25	3	696.5	696.53	34.3	0	0.002	···
ORDSYMeq	−343.88	3	693.75	693.78	31.55	0	0.002	···
ORDER	−346.93	1	695.87	695.87	33.64	0	0.0025	···

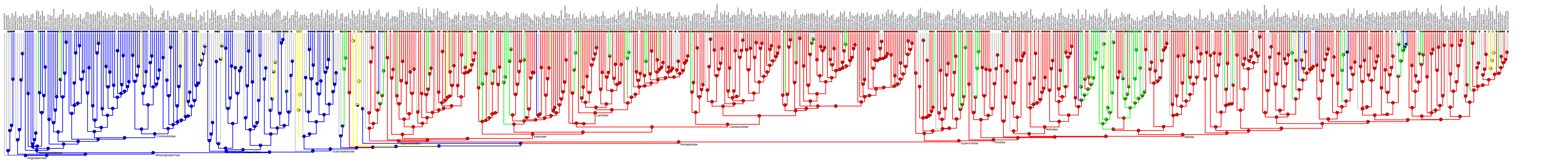

MP ancestral state reconstruction using ancestral.pars
(R:phangorn)
232_B. Perianth merism (3−state) (D2c), 70 steps

- trimerous
- tetramerous
- pentamerous

Node	MP state(s)
Angiospermae	trimerous
Mesangiospermae	trimerous
Magnoliidae	trimerous
Monocotyledonae	trimerous
Eudicotyledonae	trimerous
Commelinidae	trimerous
Pentapetalae	pentamerous
Superasteridae	pentamerous
Asteridae	pentamerous
Lamiidae	pentamerous
Campanulidae	pentamerous
Superrosidae	pentamerous
Rosidae	pentamerous
Malvidae	pentamerous
Fabidae	pentamerous

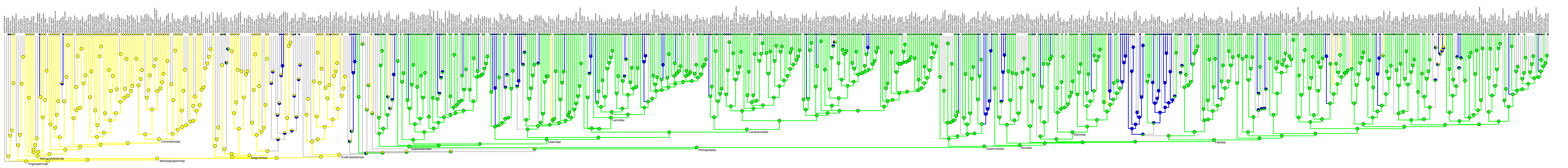

ML ancestral state reconstruction using rayDISC (R:corHMM)
232_B. Perianth merism (3−state) (D2c), SYMeq model

- trimerous (yellow)
- tetramerous (blue)
- pentamerous (green)

Node	ML state	Prob
Angiospermae	trimerous	1
Mesangiospermae	trimerous	1
Magnoliidae	trimerous	1
Monocotyledonae	trimerous	1
Eudicotyledonae	trimerous	0.9823
Commelinidae	trimerous	1
Pentapetalae	pentamerous	0.9998
Superasteridae	pentamerous	0.9999
Asteridae	pentamerous	1
Lamiidae	pentamerous	0.9999
Campanulidae	pentamerous	1
Superrosidae	pentamerous	1
Rosidae	pentamerous	1
Malvidae	pentamerous	1
Fabidae	pentamerous	1

Model	LogL	Npar	AIC	AICc	DeltaAICc	w	q01	···
ARD	−256.35	6	524.7	524.8	5.89	0.02	8e−04	···
ARDeq	−255.25	6	522.5	522.61	3.7	0.06	8e−04	···
ER	−281.81	1	565.62	565.63	46.72	0	0.0011	···
SYM	−257.53	3	521.07	521.1	2.19	0.12	0.001	···
SYMeq*	−256.44	3	518.88	518.91	0	0.37	0.001	···
ORD	−256.92	4	521.85	521.9	2.99	0.08	0.001	···
ORDeq	−255.85	4	519.69	519.75	0.84	0.24	0.001	···
ORDSYM	−260.14	2	524.28	524.29	5.38	0.03	0.0015	···
ORDSYMeq	−259.04	2	522.09	522.1	3.2	0.07	0.0015	···
ORDER	−262.6	1	527.21	527.21	8.3	0.01	0.0024	···

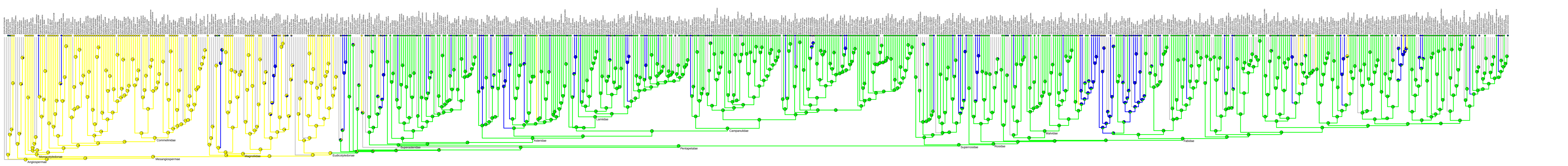

MP ancestral state reconstruction using ancestral.pars
(R:phangorn)
234_A. Perianth differentiation (binary) (D2d), 65 steps

- undifferentiated
- differentiated

Node	MP state(s)
Angiospermae	undifferentiated
Mesangiospermae	undifferentiated
Magnoliidae	undifferentiated / differentiated
Monocotyledonae	undifferentiated
Eudicotyledonae	undifferentiated / differentiated
Commelinidae	undifferentiated
Pentapetalae	differentiated
Superasteridae	differentiated
Asteridae	differentiated
Lamiidae	differentiated
Campanulidae	differentiated
Superrosidae	differentiated
Rosidae	differentiated
Malvidae	differentiated
Fabidae	differentiated

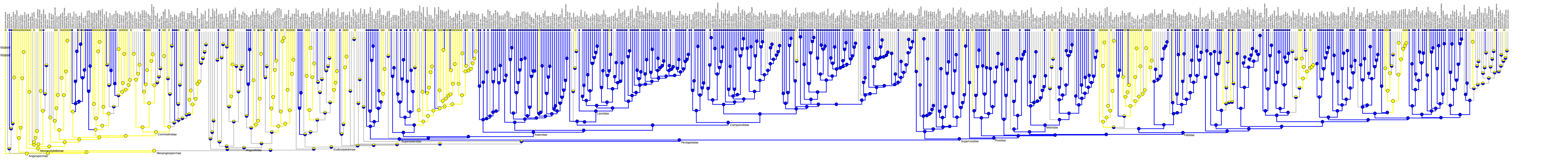

ML ancestral state reconstruction using rayDISC (R:corHMM)
234_A. Perianth differentiation (binary) (D2d), ARDeq model

- undifferentiated (yellow)
- differentiated (blue)

Node	ML state	Prob
Angiospermae	undifferentiated	1
Mesangiospermae	undifferentiated	1
Magnoliidae	undifferentiated	0.9999
Monocotyledonae	undifferentiated	1
Eudicotyledonae	undifferentiated	0.9914
Commelinidae	undifferentiated	1
Pentapetalae	differentiated	0.9564
Superasteridae	differentiated	0.9576
Asteridae	differentiated	0.9986
Lamiidae	differentiated	0.9998
Campanulidae	differentiated	1
Superrosidae	differentiated	0.9917
Rosidae	differentiated	0.9947
Malvidae	differentiated	0.9989
Fabidae	differentiated	0.987

Model	LogL	Npar	AIC	AICc	DeltaAICc	w	q01	q10
ARD	−199.62	2	403.23	403.25	1.38	0.33	0.0046	0.0015
ARDeq**	−198.92	2	401.85	401.86	0	0.67	0.0046	0.0015
ER	−209.01	1	420.03	420.03	18.17	0	0.0025	0.0025
UNI01	−228.55	1	459.09	459.1	57.23	0	0.0089	
UNI10	−237.12	1	476.25	476.25	74.39	0		0.0035

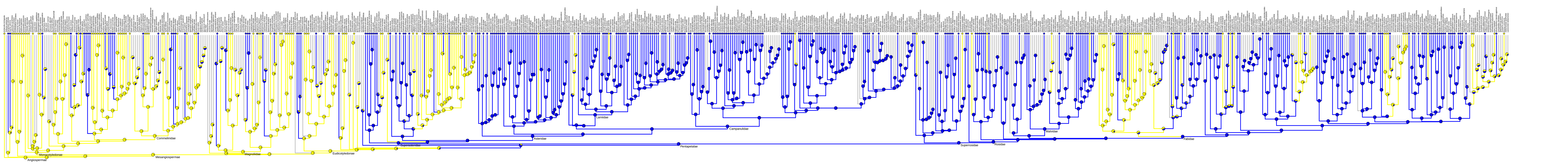

MP ancestral state reconstruction using ancestral.pars
(R:phangorn)
204_A. Fusion of perianth (D2c), 77 steps

free (<5%)
fused (>5%)

Node	MP state(s)
Angiospermae	free (<5%)
Mesangiospermae	free (<5%)
Magnoliidae	free (<5%)
Monocotyledonae	free (<5%)
Eudicotyledonae	free (<5%)
Commelinidae	free (<5%)
Pentapetalae	free (<5%)
Superasteridae	free (<5%)
Asteridae	free (<5%)
Lamiidae	free (<5%) / fused (>5%)
Campanulidae	fused (>5%)
Superrosidae	free (<5%)
Rosidae	free (<5%)
Malvidae	free (<5%)
Fabidae	free (<5%)

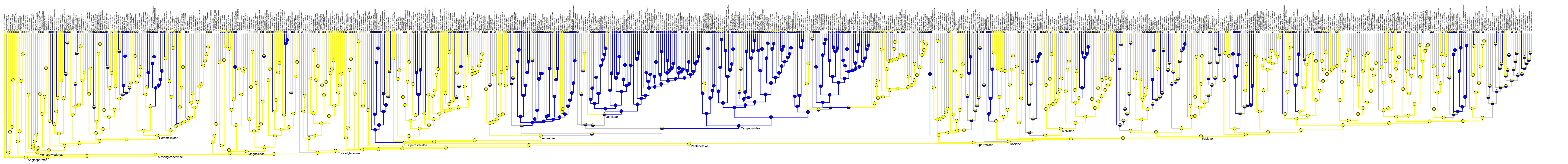

ML ancestral state reconstruction using rayDISC (R:corHMM)
204_A. Fusion of perianth (D2c), ARDeq model

- free (<5%)
- fused (>5%)

Node	ML state	Prob
Angiospermae	free (<5%)	1
Mesangiospermae	free (<5%)	1
Magnoliidae	free (<5%)	0.9999
Monocotyledonae	free (<5%)	1
Eudicotyledonae	free (<5%)	1
Commelinidae	free (<5%)	0.9989
Pentapetalae	free (<5%)	0.9993
Superasteridae	free (<5%)	0.9905
Asteridae	free (<5%)	0.8428
Lamiidae	fused (>5%)	0.9507
Campanulidae	fused (>5%)	0.923
Superrosidae	free (<5%)	0.9994
Rosidae	free (<5%)	0.9994
Malvidae	free (<5%)	0.9986
Fabidae	free (<5%)	1

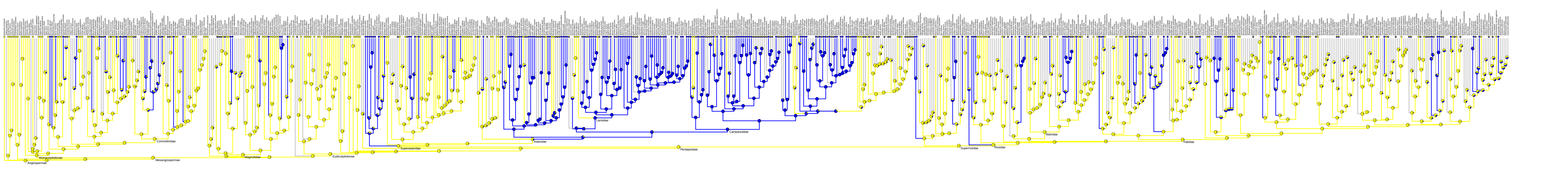

Model	LogL	Npar	AIC	AICc	DeltaAICc	w	q01	q10
ARD	−225.31	2	454.62	454.63	1.38	0.21	0.0044	0.0035
ARDeq*	−224.62	2	453.24	453.25	0	0.41	0.0044	0.0035
ER	−225.7	1	453.41	453.41	0.16	0.38	0.0042	0.0042
UNI01	−238.34	1	478.68	478.69	25.44	0	0.005	
UNI10	−236.27	1	474.53	474.54	21.29	0		0.0081

MP ancestral state reconstruction using ancestral.pars
(R:phangorn)
207_A. Symmetry of perianth (binary) (D2d), 55 steps

actinomorphic
zygomorphic

Node	MP state(s)
Angiospermae	actinomorphic
Mesangiospermae	actinomorphic
Magnoliidae	actinomorphic
Monocotyledonae	actinomorphic
Eudicotyledonae	actinomorphic
Commelinidae	actinomorphic
Pentapetalae	actinomorphic
Superasteridae	actinomorphic
Asteridae	actinomorphic
Lamiidae	actinomorphic
Campanulidae	actinomorphic
Superrosidae	actinomorphic
Rosidae	actinomorphic
Malvidae	actinomorphic
Fabidae	actinomorphic

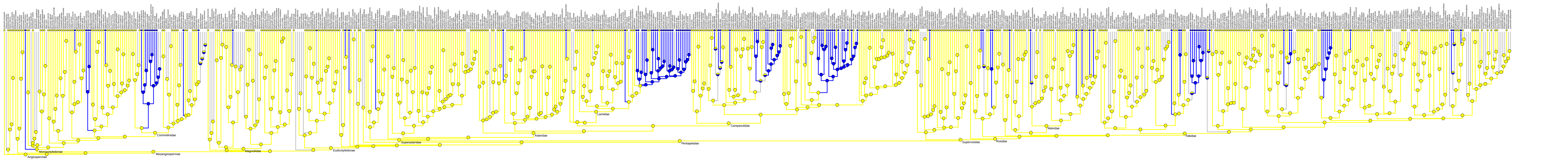

ML ancestral state reconstruction using rayDISC (R:corHMM)
207_A. Symmetry of perianth (binary) (D2d), ARDeq model

- actinomorphic
- zygomorphic

Node	ML state	Prob
Angiospermae	actinomorphic	1
Mesangiospermae	actinomorphic	1
Magnoliidae	actinomorphic	1
Monocotyledonae	actinomorphic	0.9998
Eudicotyledonae	actinomorphic	1
Commelinidae	actinomorphic	0.9967
Pentapetalae	actinomorphic	1
Superasteridae	actinomorphic	1
Asteridae	actinomorphic	1
Lamiidae	actinomorphic	0.9965
Campanulidae	actinomorphic	1
Superrosidae	actinomorphic	1
Rosidae	actinomorphic	1
Malvidae	actinomorphic	0.9997
Fabidae	actinomorphic	0.9999

Model	LogL	Npar	AIC	AICc	DeltaAICc	w	q01	q10
ARD	−210.43	2	424.86	424.88	1.38	0.33	0.0014	0.0059
ARDeq**	−209.74	2	423.48	423.5	0	0.66	0.0014	0.0059
ER	−218.72	1	439.44	439.45	15.95	0	0.0015	0.0015
UNI01	−261.41	1	524.82	524.82	101.33	0	0.0018	
UNI10	−217.44	1	436.87	436.88	13.38	0		0.0166

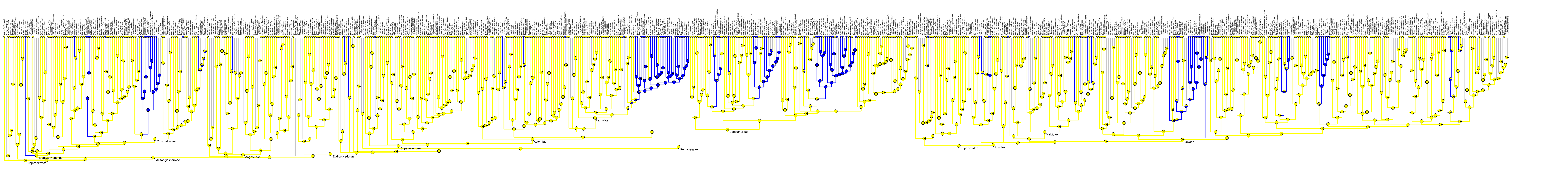

MP ancestral state reconstruction using ancestral.pars
(R:phangorn)
301_B. Number of fertile stamens (3−state) (D2c), 157 steps

- one to five (1−5)
- six to ten (6−10)
- more than ten (>10)

Node	MP state(s)
Angiospermae	more than ten (>10)
Mesangiospermae	one to five (1−5) / six to ten (6−10) / more than ten (>10)
Magnoliidae	more than ten (>10)
Monocotyledonae	six to ten (6−10)
Eudicotyledonae	one to five (1−5)
Commelinidae	six to ten (6−10)
Pentapetalae	six to ten (6−10)
Superasteridae	six to ten (6−10)
Asteridae	one to five (1−5) / six to ten (6−10)
Lamiidae	one to five (1−5)
Campanulidae	one to five (1−5)
Superrosidae	six to ten (6−10)
Rosidae	six to ten (6−10)
Malvidae	six to ten (6−10)
Fabidae	six to ten (6−10)

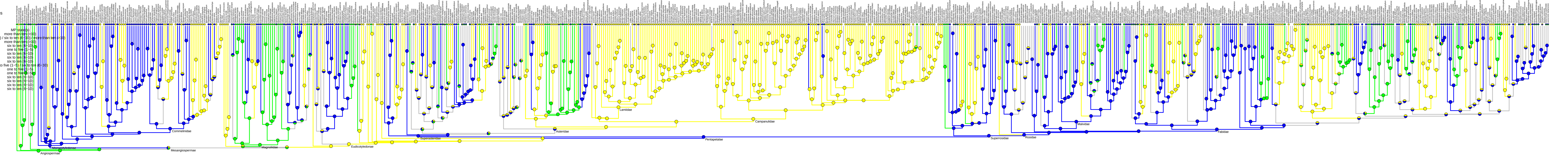

ML ancestral state reconstruction using rayDISC (R:corHMM)
301_B. Number of fertile stamens (3−state) (D2c), ARDeq model

- one to five (1−5)
- six to ten (6−10)
- more than ten (>10)

Node	ML state	Prob
Angiospermae	more than ten (>10)	1
Mesangiospermae	more than ten (>10)	0.9998
Magnoliidae	more than ten (>10)	1
Monocotyledonae	six to ten (6−10)	0.5554
Eudicotyledonae	more than ten (>10)	1
Commelinidae	six to ten (6−10)	0.9996
Pentapetalae	more than ten (>10)	1
Superasteridae	more than ten (>10)	1
Asteridae	more than ten (>10)	0.9994
Lamiidae	one to five (1−5)	0.9995
Campanulidae	one to five (1−5)	0.9966
Superrosidae	more than ten (>10)	1
Rosidae	more than ten (>10)	0.9999
Malvidae	more than ten (>10)	0.9418
Fabidae	more than ten (>10)	1

Model	LogL	Npar	AIC	AICc	DeltaAICc	w	q01	···
ARD	−493.28	6	998.56	998.66	2.2	0.25	4e−04	···
ARDeq**	−492.18	6	996.36	996.47	0	0.75	4e−04	···
ER	−538.35	1	1078.69	1078.7	82.23	0	0.0027	···
SYM	−530.98	3	1067.96	1067.99	71.52	0	0.0039	···
SYMeq	−530.13	3	1066.25	1066.28	69.82	0	0.0039	···
ORD	−509.98	4	1027.96	1028.01	31.54	0	8e−04	···
ORDeq	−509.41	4	1026.82	1026.87	30.4	0	8e−04	···
ORDSYM	−534.65	2	1073.29	1073.31	76.84	0	0.0044	···
ORDSYMeq	−533.73	2	1071.47	1071.49	75.02	0	0.0044	···
ORDER	−534.68	1	1071.36	1071.37	74.9	0	0.0044	···

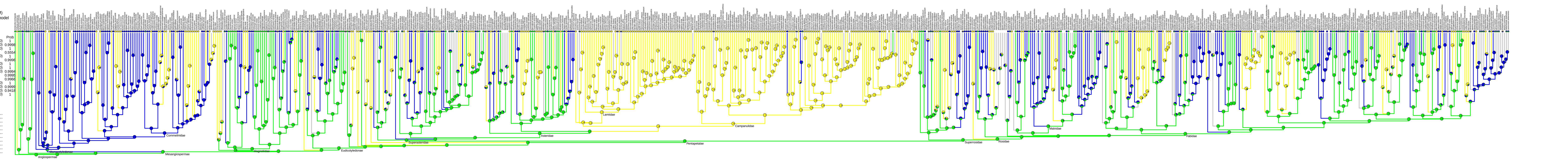

MP ancestral state reconstruction using ancestral.pars
(R:phangorn)
301_C. Number of fertile stamens (binary) (D2c), 95 steps

- one to six (1−6)
- more than six (>6)

Node	MP state(s)
Angiospermae	more than six (>6)
Mesangiospermae	one to six (1−6)
Magnoliidae	one to six (1−6) / more than six (>6)
Monocotyledonae	one to six (1−6)
Eudicotyledonae	one to six (1−6)
Commelinidae	one to six (1−6)
Pentapetalae	more than six (>6)
Superasteridae	more than six (>6)
Asteridae	one to six (1−6) / more than six (>6)
Lamiidae	one to six (1−6)
Campanulidae	one to six (1−6)
Superrosidae	more than six (>6)
Rosidae	more than six (>6)
Malvidae	more than six (>6)
Fabidae	more than six (>6)

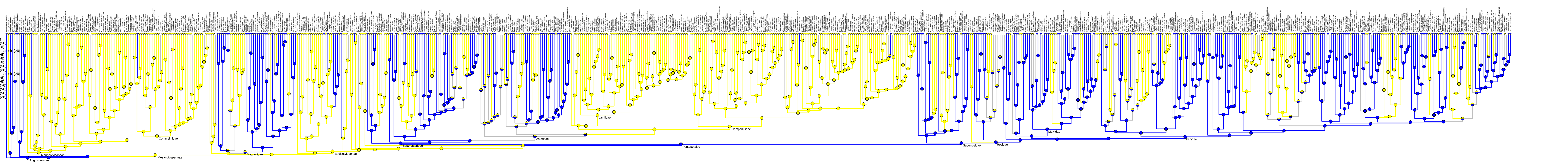

ML ancestral state reconstruction using rayDISC (R:corHMM)
301_C. Number of fertile stamens (binary) (D2c), ARDeq model

- one to six (1−6)
- more than six (>6)

Node	ML state	Prob
Angiospermae	more than six (>6)	1
Mesangiospermae	more than six (>6)	0.9999
Magnoliidae	more than six (>6)	1
Monocotyledonae	more than six (>6)	0.5202
Eudicotyledonae	more than six (>6)	0.9999
Commelinidae	one to six (1−6)	1
Pentapetalae	more than six (>6)	1
Superasteridae	more than six (>6)	1
Asteridae	more than six (>6)	0.9999
Lamiidae	one to six (1−6)	0.999
Campanulidae	one to six (1−6)	0.9937
Superrosidae	more than six (>6)	1
Rosidae	more than six (>6)	1
Malvidae	more than six (>6)	1
Fabidae	more than six (>6)	1

Model	LogL	Npar	AIC	AICc	DeltaAICc	w	q01	q10
ARD	−289.73	2	583.46	583.48	1.39	0.33	5e−04	0.006
ARDeq**	−289.04	2	582.08	582.09	0	0.67	5e−04	0.006
ER	−325.04	1	652.09	652.09	70	0	0.0032	0.0032
UNI01	−365.16	1	732.31	732.32	150.23	0	0.0046	
UNI10	−326.26	1	654.51	654.52	72.43	0		0.0075

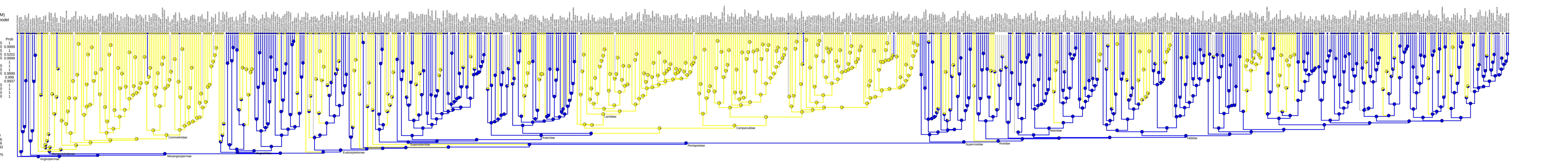

MP ancestral state reconstruction using ancestral.pars
(R:phangorn)
330_A. Androecium structural phyllotaxy (binary) (D2d), 12 steps

whorled
spiral

Node	MP state(s)
Angiospermae	whorled / spiral
Mesangiospermae	whorled
Magnoliidae	whorled
Monocotyledonae	whorled
Eudicotyledonae	whorled
Commelinidae	whorled
Pentapetalae	whorled
Superasteridae	whorled
Asteridae	whorled
Lamiidae	whorled
Campanulidae	whorled
Superrosidae	whorled
Rosidae	whorled
Malvidae	whorled
Fabidae	whorled

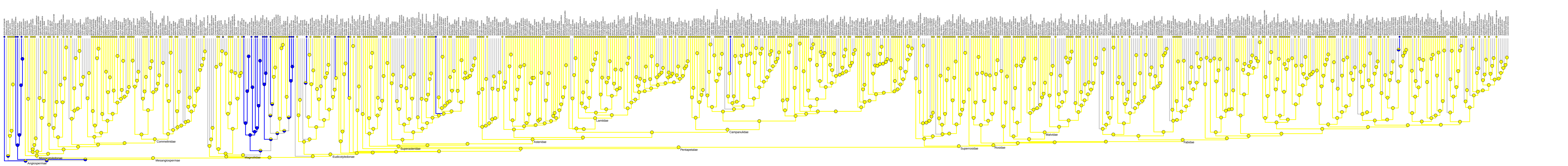

ML ancestral state reconstruction using rayDISC (R:corHMM)
330_A. Androecium structural phyllotaxy (binary) (D2d), UNI01 model

whorled
spiral

Node	ML state	Prob
Angiospermae	whorled	1
Mesangiospermae	whorled	1
Magnoliidae	whorled	1
Monocotyledonae	whorled	1
Eudicotyledonae	whorled	1
Commelinidae	whorled	1
Pentapetalae	whorled	1
Superasteridae	whorled	1
Asteridae	whorled	1
Lamiidae	whorled	1
Campanulidae	whorled	1
Superrosidae	whorled	1
Rosidae	whorled	1
Malvidae	whorled	1
Fabidae	whorled	1

Model	LogL	Npar	AIC	AICc	DeltaAICc	w	q01	q10
ARD	−59.74	2	123.48	123.49	2.01	0.13	4e−04	0
ARDeq	−59.05	2	122.09	122.11	0.62	0.26	4e−04	0
ER	−60	1	122	122.01	0.53	0.27	4e−04	4e−04
UNI01*	−59.74	1	121.48	121.48	0	0.35	4e−04	
UNI10	−76.07	1	154.14	154.14	32.66	0		0.0213

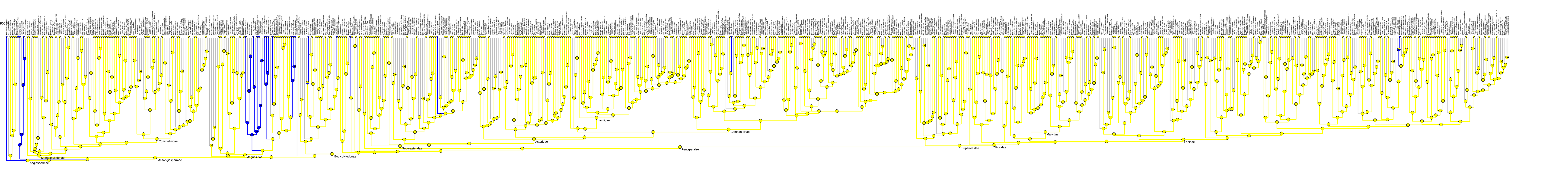

MP ancestral state reconstruction using ancestral.pars
(R:phangorn)
331_A. Number of androecium structural whorls (3−state) (D2c), 75 steps

- one (1)
- two (2)
- more than two (>2)

Node	MP state(s)
Angiospermae	more than two (>2)
Mesangiospermae	more than two (>2)
Magnoliidae	more than two (>2)
Monocotyledonae	two (2)
Eudicotyledonae	more than two (>2)
Commelinidae	two (2)
Pentapetalae	one (1)
Superasteridae	one (1)
Asteridae	one (1)
Lamiidae	one (1)
Campanulidae	one (1)
Superrosidae	one (1)
Rosidae	one (1)
Malvidae	one (1) / two (2)
Fabidae	one (1) / two (2)

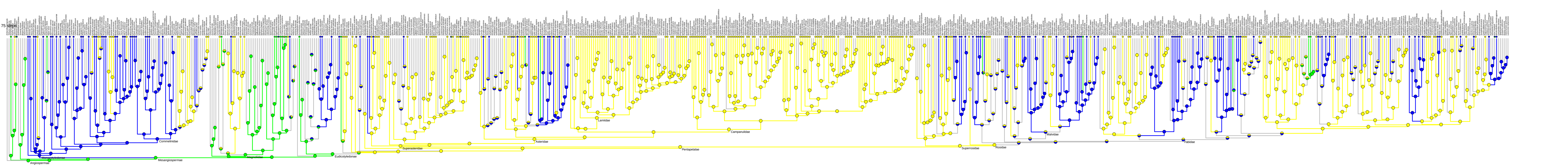

ML ancestral state reconstruction using rayDISC (R:corHMM)

331_A. Number of androecium structural whorls (3−state) (D2c), ARDeq model

- one (1)
- two (2)
- more than two (>2)

Node	ML state	Prob
Angiospermae	more than two (>2)	1
Mesangiospermae	more than two (>2)	0.9994
Magnoliidae	more than two (>2)	0.9991
Monocotyledonae	more than two (>2)	0.9699
Eudicotyledonae	more than two (>2)	0.9322
Commelinidae	two (2)	0.9087
Pentapetalae	two (2)	0.9958
Superasteridae	two (2)	0.9984
Asteridae	two (2)	0.9999
Lamiidae	one (1)	0.9987
Campanulidae	one (1)	0.9917
Superrosidae	two (2)	0.9988
Rosidae	two (2)	0.9992
Malvidae	two (2)	0.9994
Fabidae	two (2)	1

Model	LogL	Npar	AIC	AICc	DeltaAICc	w	q01	···
ARD	−232.27	6	476.53	476.64	2.18	0.2	0	···
ARDeq**	−231.17	6	474.35	474.46	0	0.6	0	···
ER	−281.75	1	565.5	565.51	91.05	0	0.0017	···
SYM	−256.04	3	518.08	518.11	43.66	0	0.0035	···
SYMeq	−254.98	3	515.96	515.99	41.53	0	0.0035	···
ORD	−235.64	4	479.27	479.32	4.87	0.05	0	···
ORDeq	−234.6	4	477.2	477.25	2.79	0.15	0	···
ORDSYM	−256.41	2	516.83	516.84	42.39	0	0.0036	···
ORDSYMeq	−255.34	2	514.68	514.69	40.24	0	0.0036	···
ORDER	−261.48	1	524.96	524.96	50.51	0	0.0029	···

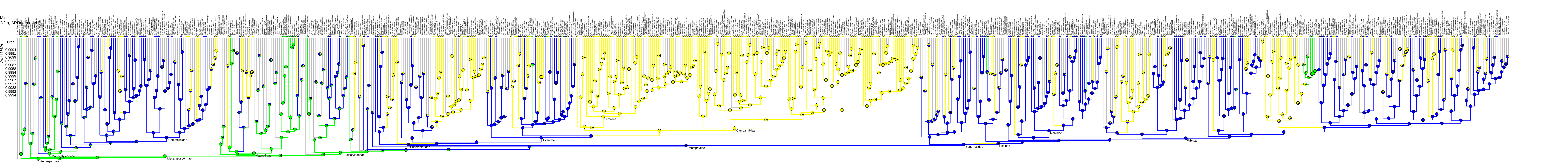

MP ancestral state reconstruction using ancestral.pars
(R:phangorn)
332_A. Androecium structural merism (4−state) (D2c), 58 steps

- dimerous
- trimerous
- tetramerous
- pentamerous

Node	MP state(s)
Angiospermae	trimerous
Mesangiospermae	trimerous
Magnoliidae	trimerous
Monocotyledonae	trimerous
Eudicotyledonae	trimerous / tetramerous / pentamerous
Commelinidae	trimerous
Pentapetalae	pentamerous
Superasteridae	pentamerous
Asteridae	pentamerous
Lamiidae	pentamerous
Campanulidae	pentamerous
Superrosidae	pentamerous
Rosidae	pentamerous
Malvidae	pentamerous
Fabidae	pentamerous

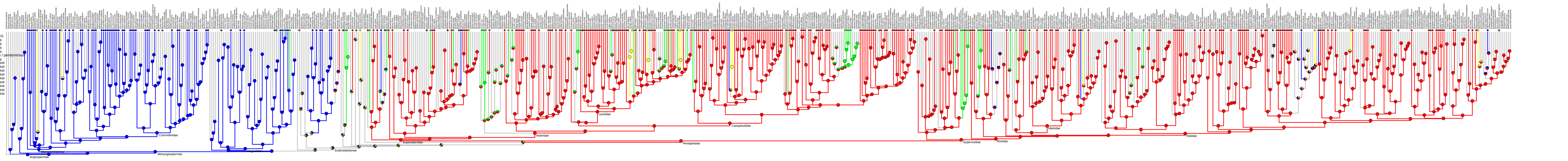

ML ancestral state reconstruction using rayDISC (R:corHMM)
332_A. Androecium structural merism (4−state) (D2c), ORDeq model

- dimerous
- trimerous
- tetramerous
- pentamerous

Node	ML state	Prob
Angiospermae	dimerous	0.4999
Mesangiospermae	trimerous	0.5059
Magnoliidae	trimerous	0.5057
Monocotyledonae	trimerous	0.5069
Eudicotyledonae	tetramerous	0.541
Commelinidae	trimerous	0.5071
Pentapetalae	tetramerous	0.8355
Superasteridae	tetramerous	0.8229
Asteridae	tetramerous	0.5847
Lamiidae	pentamerous	0.7485
Campanulidae	pentamerous	0.792
Superrosidae	tetramerous	0.7825
Rosidae	tetramerous	0.7451
Malvidae	pentamerous	0.542
Fabidae	pentamerous	0.5618

Model	LogL	Npar	AIC	AICc	DeltaAICc	w	q01	···
ARD	−223.41	12	470.82	471.22	231.43	0	0.007	···
ARDeq	−222.07	12	468.14	468.54	228.75	0	0.0068	···
ER	−237.58	1	477.17	477.17	237.38	0	9e−04	···
SYM	−225.35	6	462.71	462.81	223.02	0	5e−04	···
SYMeq	−223.98	6	459.95	460.06	220.27	0	5e−04	···
ORD	−160.64	6	333.28	333.38	93.59	0	100	···
ORDeq***	−113.84	6	239.68	239.79	0	1	99.997	···
ORDSYM	−259.14	3	524.29	524.32	284.53	0	0.0021	···
ORDSYMeq	−257.81	3	521.61	521.64	281.85	0	0.0021	···
ORDER	−260	1	521.99	522	282.21	0	0.0031	···

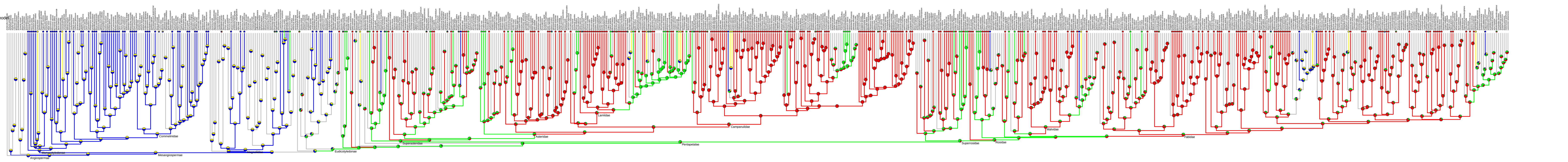

MP ancestral state reconstruction using ancestral.pars
(R:phangorn)
332_B. Androecium structural merism (3−state) (D2c), 49 steps

- trimerous
- tetramerous
- pentamerous

Node	MP state(s)
Angiospermae	trimerous
Mesangiospermae	trimerous
Magnoliidae	trimerous
Monocotyledonae	trimerous
Eudicotyledonae	trimerous / tetramerous / pentamerous
Commelinidae	trimerous
Pentapetalae	pentamerous
Superasteridae	pentamerous
Asteridae	pentamerous
Lamiidae	pentamerous
Campanulidae	pentamerous
Superrosidae	pentamerous
Rosidae	pentamerous
Malvidae	pentamerous
Fabidae	pentamerous

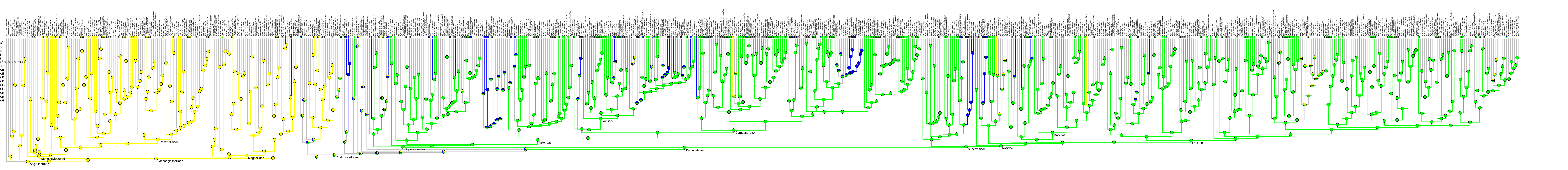

ML ancestral state reconstruction using rayDISC (R:corHMM)
332_B. Androecium structural merism (3−state) (D2c), ARDeq model

- trimerous
- tetramerous
- pentamerous

Node	ML state	Prob
Angiospermae	trimerous	1
Mesangiospermae	trimerous	1
Magnoliidae	trimerous	1
Monocotyledonae	trimerous	1
Eudicotyledonae	trimerous	0.9174
Commelinidae	trimerous	1
Pentapetalae	tetramerous	1
Superasteridae	tetramerous	1
Asteridae	tetramerous	1
Lamiidae	tetramerous	0.981
Campanulidae	tetramerous	0.9402
Superrosidae	tetramerous	1
Rosidae	tetramerous	0.999
Malvidae	pentamerous	0.5079
Fabidae	tetramerous	0.9427

Model	LogL	Npar	AIC	AICc	DeltaAICc	w	q01	···
ARD	−171.5	6	355	355.11	2.19	0.23	9e−04	···
ARDeq**	−170.41	6	352.81	352.92	0	0.7	9e−04	···
ER	−186.49	1	374.98	374.99	22.07	0	0.0011	···
SYM	−177.31	3	360.63	360.66	7.74	0.01	5e−04	···
SYMeq	−176.22	3	358.44	358.47	5.55	0.04	5e−04	···
ORD	−178.47	4	364.95	365	12.08	0	0	···
ORDeq	−177.4	4	362.79	362.84	9.92	0	0	···
ORDSYM	−186.31	2	376.62	376.64	23.72	0	0.002	···
ORDSYMeq	−185.23	2	374.46	374.47	21.55	0	0.002	···
ORDER	−187.15	1	376.29	376.3	23.38	0	0.0027	···

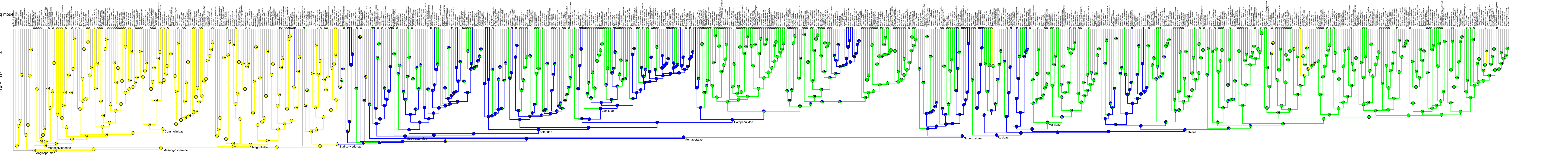

MP ancestral state reconstruction using ancestral.pars
(R:phangorn)
305_A. Filament (binary) (D2d), 53 steps

- laminar (wide)
- typical (narrow)

Node	MP state(s)
Angiospermae	laminar (wide) / typical (narrow)
Mesangiospermae	laminar (wide) / typical (narrow)
Magnoliidae	laminar (wide)
Monocotyledonae	typical (narrow)
Eudicotyledonae	typical (narrow)
Commelinidae	typical (narrow)
Pentapetalae	typical (narrow)
Superasteridae	typical (narrow)
Asteridae	typical (narrow)
Lamiidae	typical (narrow)
Campanulidae	typical (narrow)
Superrosidae	typical (narrow)
Rosidae	typical (narrow)
Malvidae	typical (narrow)
Fabidae	typical (narrow)

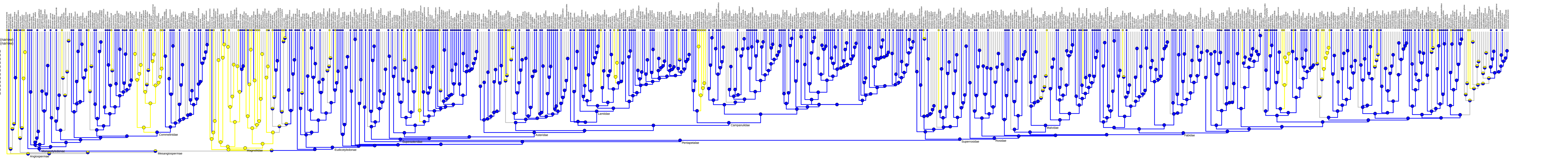

ML ancestral state reconstruction using rayDISC (R:corHMM)
305_A. Filament (binary) (D2d), ARDeq model

- laminar (wide)
- typical (narrow)

Node	ML state	Prob
Angiospermae	laminar (wide)	1
Mesangiospermae	laminar (wide)	0.9926
Magnoliidae	laminar (wide)	0.9985
Monocotyledonae	laminar (wide)	0.867
Eudicotyledonae	typical (narrow)	0.5676
Commelinidae	laminar (wide)	0.684
Pentapetalae	typical (narrow)	0.999
Superasteridae	typical (narrow)	0.9995
Asteridae	typical (narrow)	0.9997
Lamiidae	typical (narrow)	0.997
Campanulidae	typical (narrow)	0.9961
Superrosidae	typical (narrow)	0.9995
Rosidae	typical (narrow)	0.9996
Malvidae	typical (narrow)	0.9974
Fabidae	typical (narrow)	1

Model	LogL	Npar	AIC	AICc	DeltaAICc	w	q01	q10
ARD	−172.23	2	348.47	348.48	1.28	0.34	0.0089	0.0021
ARDeq**	−171.59	2	347.18	347.2	0	0.65	0.009	0.0021
ER	−177.9	1	357.8	357.81	10.61	0	0.0024	0.0024
UNI01	−183.93	1	369.86	369.87	22.67	0	0.0147	
UNI10	−187.81	1	377.61	377.62	30.42	0		0.0023

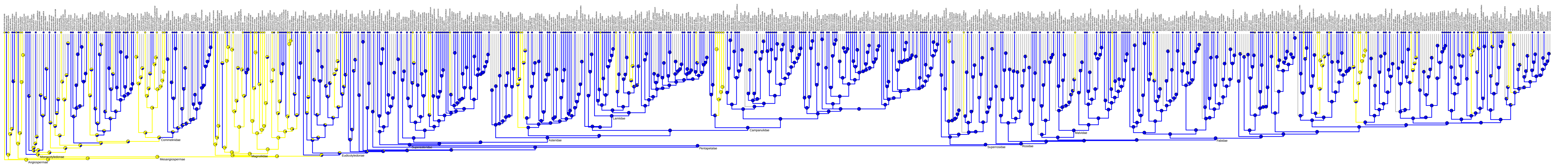

MP ancestral state reconstruction using ancestral.pars
(R:phangorn)
311_A. Anther orientation (D2d), 109 steps

- introrse
- latrorse
- extrorse

Node	MP state(s)
Angiospermae	introrse
Mesangiospermae	introrse
Magnoliidae	extrorse
Monocotyledonae	introrse
Eudicotyledonae	extrorse
Commelinidae	introrse
Pentapetalae	introrse
Superasteridae	introrse
Asteridae	introrse
Lamiidae	introrse
Campanulidae	introrse
Superrosidae	introrse
Rosidae	introrse
Malvidae	introrse
Fabidae	introrse

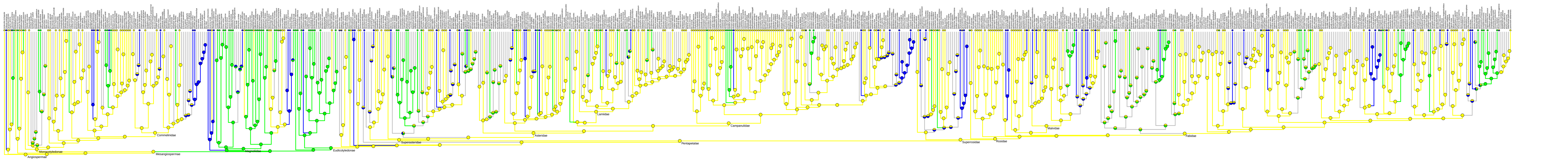

ML ancestral state reconstruction using rayDISC (R:corHMM)
311_A. Anther orientation (D2d), SYMeq model

- introrse
- latrorse
- extrorse

Node	ML state	Prob
Angiospermae	introrse	1
Mesangiospermae	introrse	0.9985
Magnoliidae	extrorse	0.7688
Monocotyledonae	introrse	0.9988
Eudicotyledonae	introrse	0.9822
Commelinidae	introrse	0.995
Pentapetalae	introrse	1
Superasteridae	introrse	1
Asteridae	introrse	0.9998
Lamiidae	introrse	0.9997
Campanulidae	introrse	1
Superrosidae	introrse	1
Rosidae	introrse	1
Malvidae	introrse	0.9998
Fabidae	introrse	0.9998

Model	LogL	Npar	AIC	AICc	DeltaAICc	w	q01	···
ARD	−345.14	6	702.28	702.39	2.85	0.08	0.0018	···
ARDeq	−344.48	6	700.96	701.06	1.53	0.16	0.0037	···
ER	−348.83	1	699.66	699.67	0.13	0.31	0.0028	···
SYM	−347.81	3	701.62	701.65	2.12	0.12	0.0031	···
SYMeq*	−346.75	3	699.51	699.54	0	0.33	0.0031	···

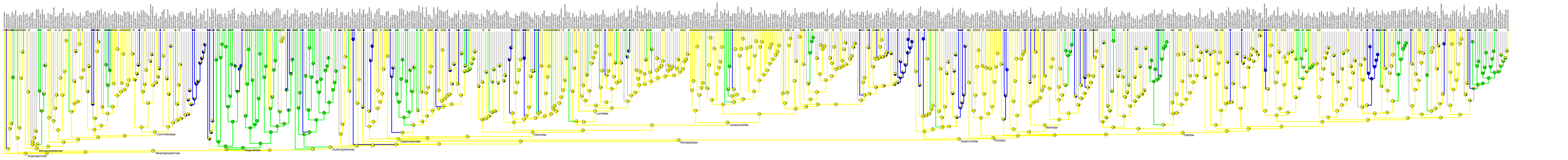

MP ancestral state reconstruction using ancestral.pars
(R:phangorn)
312_A. Anther attachment (binary) (D2d), 97 steps

- basifixed
- dorsifixed

Node	MP state(s)
Angiospermae	basifixed
Mesangiospermae	basifixed
Magnoliidae	basifixed
Monocotyledonae	basifixed
Eudicotyledonae	basifixed
Commelinidae	basifixed
Pentapetalae	basifixed
Superasteridae	basifixed
Asteridae	basifixed
Lamiidae	basifixed
Campanulidae	basifixed / dorsifixed
Superrosidae	basifixed
Rosidae	dorsifixed
Malvidae	dorsifixed
Fabidae	dorsifixed

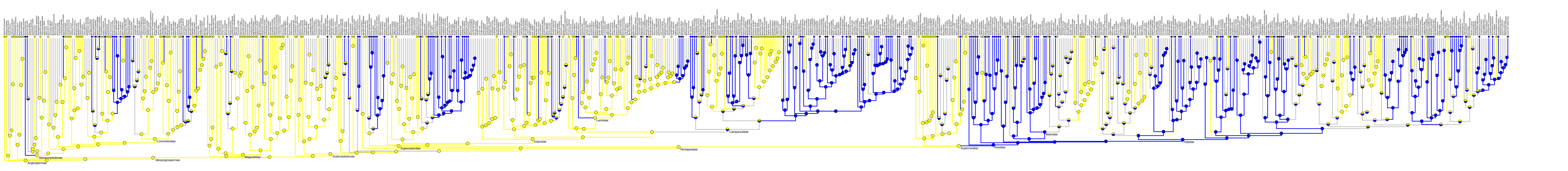

ML ancestral state reconstruction using rayDISC (R:corHMM)
312_A. Anther attachment (binary) (D2d), ARDeq model

- basifixed
- dorsifixed

Node	ML state	Prob
Angiospermae	basifixed	1
Mesangiospermae	basifixed	0.9995
Magnoliidae	basifixed	0.999
Monocotyledonae	basifixed	0.9958
Eudicotyledonae	basifixed	0.8848
Commelinidae	basifixed	0.9837
Pentapetalae	dorsifixed	0.8862
Superasteridae	dorsifixed	0.8927
Asteridae	dorsifixed	0.874
Lamiidae	dorsifixed	0.5707
Campanulidae	dorsifixed	0.9576
Superrosidae	dorsifixed	0.895
Rosidae	dorsifixed	0.9396
Malvidae	dorsifixed	0.9775
Fabidae	dorsifixed	0.9997

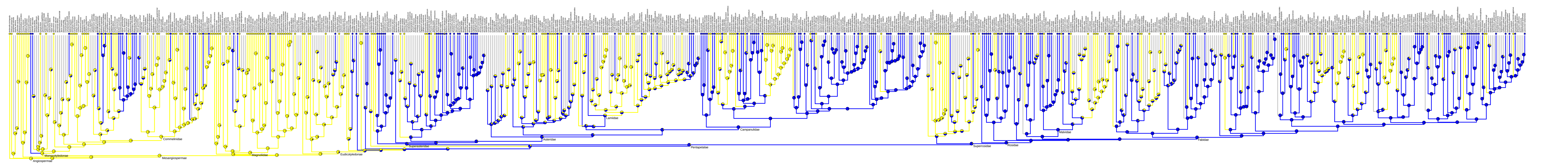

Model	LogL	Npar	AIC	AICc	DeltaAICc	w	q01	q10
ARD	−265.1	2	534.19	534.21	1.34	0.28	0.0043	0.0075
ARDeq**	−264.43	2	532.85	532.87	0	0.55	0.0043	0.0074
ER	−266.63	1	535.26	535.26	2.39	0.17	0.0055	0.0055
UNI01	−293.34	1	588.69	588.69	55.82	0	0.0062	
UNI10	−276.78	1	555.56	555.57	22.7	0		0.0081

MP ancestral state reconstruction using ancestral.pars
(R:phangorn)
313_A. Anther dehiscence (3−state) (D2d), 10 steps

- longitudinal slit
- H−valvate
- flap−valvate

Node	MP state(s)
Angiospermae	longitudinal slit
Mesangiospermae	longitudinal slit
Magnoliidae	longitudinal slit
Monocotyledonae	longitudinal slit
Eudicotyledonae	longitudinal slit
Commelinidae	longitudinal slit
Pentapetalae	longitudinal slit
Superasteridae	longitudinal slit
Asteridae	longitudinal slit
Lamiidae	longitudinal slit
Campanulidae	longitudinal slit
Superrosidae	longitudinal slit
Rosidae	longitudinal slit
Malvidae	longitudinal slit
Fabidae	longitudinal slit

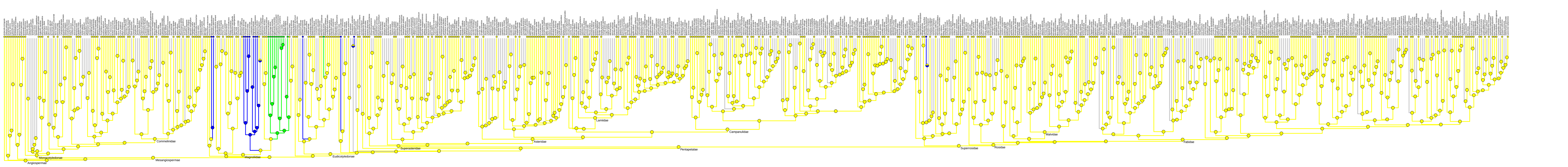

ML ancestral state reconstruction using rayDISC (R:corHMM)
313_A. Anther dehiscence (3−state) (D2d), ARDeq model

- longitudinal slit
- H−valvate
- flap−valvate

Node	ML state	Prob
Angiospermae	longitudinal slit	1
Mesangiospermae	longitudinal slit	1
Magnoliidae	longitudinal slit	0.9992
Monocotyledonae	longitudinal slit	1
Eudicotyledonae	longitudinal slit	1
Commelinidae	longitudinal slit	1
Pentapetalae	longitudinal slit	1
Superasteridae	longitudinal slit	1
Asteridae	longitudinal slit	1
Lamiidae	longitudinal slit	1
Campanulidae	longitudinal slit	1
Superrosidae	longitudinal slit	1
Rosidae	longitudinal slit	1
Malvidae	longitudinal slit	1
Fabidae	longitudinal slit	1

Model	LogL	Npar	AIC	AICc	DeltaAICc	w	q01	···
ARD	−56.42	6	124.84	124.95	2.2	0.1	2e−04	···
ARDeq*	−55.32	6	122.65	122.75	0	0.31	2e−04	···
ER	−60.68	1	123.36	123.36	0.61	0.23	2e−04	···
SYM	−59.63	3	125.25	125.28	2.53	0.09	2e−04	···
SYMeq	−58.53	3	123.06	123.09	0.33	0.26	2e−04	···

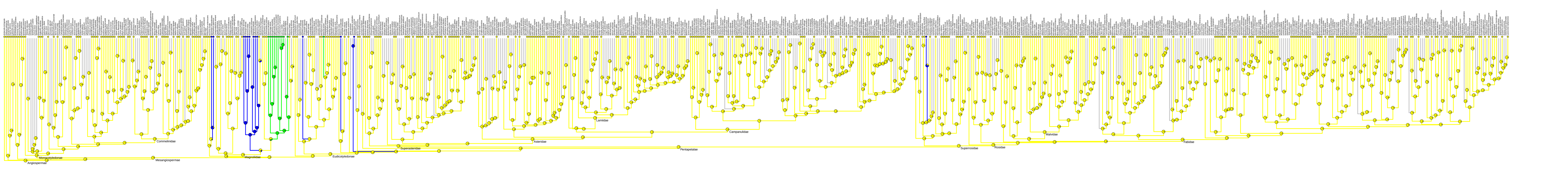

MP ancestral state reconstruction using ancestral.pars
(R:phangorn)
401_B. Number of structural carpels (5−state) (D2c), 190 steps

- one (1)
- two (2)
- three (3)
- four or five (4−5)
- more than five (>5)

Node	MP state(s)
Angiospermae	more than five (>5)
Mesangiospermae	more than five (>5)
Magnoliidae	more than five (>5)
Monocotyledonae	three (3)
Eudicotyledonae	more than five (>5)
Commelinidae	three (3)
Pentapetalae	three (3)
Superasteridae	three (3)
Asteridae	two (2) / four or five (4−5)
Lamiidae	two (2)
Campanulidae	two (2)
Superrosidae	three (3)
Rosidae	three (3)
Malvidae	four or five (4−5)
Fabidae	four or five (4−5)

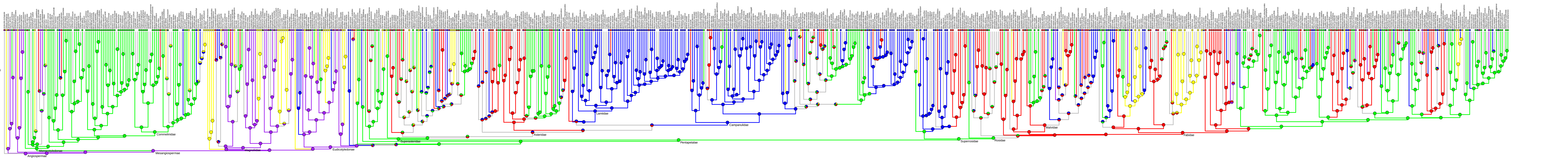

ML ancestral state reconstruction using rayDISC (R:corHMM)
401_B. Number of structural carpels (5−state) (D2c), ARDeq model

- one (1)
- two (2)
- three (3)
- four or five (4−5)
- more than five (>5)

Node	ML state	Prob
Angiospermae	more than five (>5)	1
Mesangiospermae	more than five (>5)	1
Magnoliidae	more than five (>5)	1
Monocotyledonae	more than five (>5)	0.8134
Eudicotyledonae	more than five (>5)	0.997
Commelinidae	three (3)	1
Pentapetalae	four or five (4−5)	0.9999
Superasteridae	four or five (4−5)	0.9998
Asteridae	four or five (4−5)	0.9949
Lamiidae	two (2)	0.9962
Campanulidae	two (2)	0.9351
Superrosidae	four or five (4−5)	0.9999
Rosidae	four or five (4−5)	0.9998
Malvidae	four or five (4−5)	0.999
Fabidae	four or five (4−5)	1

Model	LogL	Npar	AIC	AICc	DeltaAICc	w	q01	⋯
ARD	−665.76	20	1371.52	1372.61	3.22	0.17	0.0019	⋯
ARDeq**	−664.15	20	1368.31	1369.4	0	0.83	0.0019	⋯
ER	−721.35	1	1444.71	1444.71	75.32	0	0.0016	⋯
SYM	−686.03	10	1392.05	1392.33	22.94	0	9e−04	⋯
SYMeq	−684.52	10	1389.05	1389.33	19.93	0	9e−04	⋯
ORD	−758.83	8	1533.66	1533.84	164.45	0	9e−04	⋯
ORDeq	−757.3	8	1530.59	1530.78	161.38	0	9e−04	⋯
ORDSYM	−762.6	4	1533.21	1533.26	163.86	0	0.0043	⋯
ORDSYMeq	−761.14	4	1530.28	1530.33	160.93	0	0.0043	⋯
ORDER	−767.62	1	1537.23	1537.24	167.84	0	0.0059	⋯

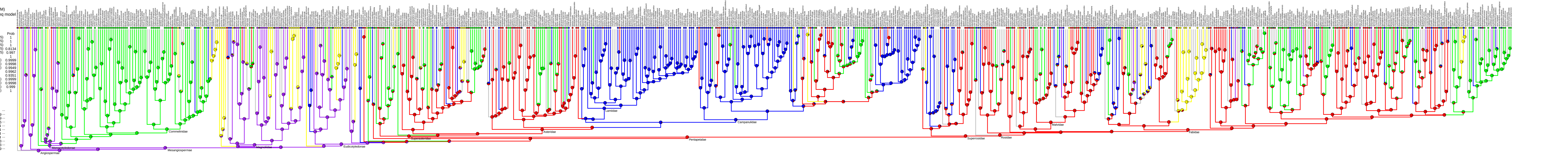

MP ancestral state reconstruction using ancestral.pars
(R:phangorn)
400_A. Gynoecium phyllotaxy (D2d), 10 steps

- whorled
- spiral

Node	MP state(s)
Angiospermae	whorled / spiral
Mesangiospermae	whorled
Magnoliidae	whorled
Monocotyledonae	whorled
Eudicotyledonae	whorled
Commelinidae	whorled
Pentapetalae	whorled
Superasteridae	whorled
Asteridae	whorled
Lamiidae	whorled
Campanulidae	whorled
Superrosidae	whorled
Rosidae	whorled
Malvidae	whorled
Fabidae	whorled

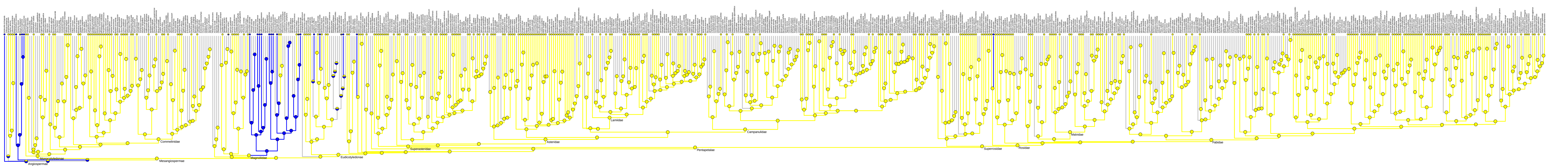

ML ancestral state reconstruction using rayDISC (R:corHMM)
400_A. Gynoecium phyllotaxy (D2d), ARDeq model

- whorled
- spiral

Node	ML state	Prob
Angiospermae	spiral	1
Mesangiospermae	spiral	1
Magnoliidae	spiral	1
Monocotyledonae	whorled	0.519
Eudicotyledonae	spiral	0.9997
Commelinidae	whorled	0.9996
Pentapetalae	whorled	0.9808
Superasteridae	whorled	0.995
Asteridae	whorled	1
Lamiidae	whorled	1
Campanulidae	whorled	1
Superrosidae	whorled	0.9829
Rosidae	whorled	0.983
Malvidae	whorled	1
Fabidae	whorled	1

Model	LogL	Npar	AIC	AICc	DeltaAICc	w	q01	q10
ARD	−40.28	2	84.57	84.58	1.39	0.32	1e−04	0.0084
ARDeq**	−39.59	2	83.18	83.2	0	0.63	1e−04	0.0084
ER	−51.76	1	105.52	105.52	22.32	0	4e−04	4e−04
UNI01	−53.31	1	108.62	108.62	25.42	0	4e−04	
UNI10	−43.15	1	88.31	88.31	5.11	0.05		0.0097

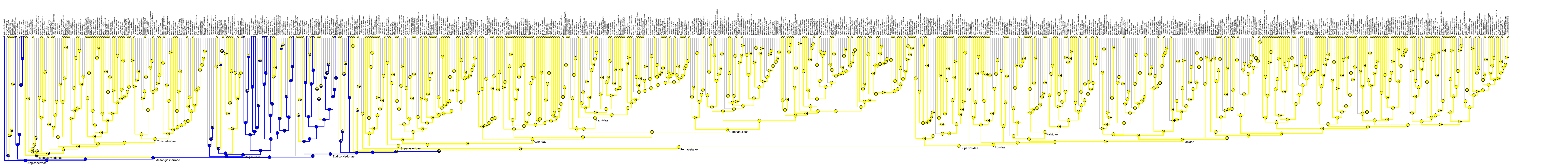

MP ancestral state reconstruction using ancestral.pars
(R:phangorn)
403_A. Fusion of ovaries (binary) (D2c), 24 steps

- free (<5%)
- fused (>5%)

Node	MP state(s)
Angiospermae	free (<5%) / fused (>5%)
Mesangiospermae	free (<5%) / fused (>5%)
Magnoliidae	free (<5%) / fused (>5%)
Monocotyledonae	fused (>5%)
Eudicotyledonae	free (<5%) / fused (>5%)
Commelinidae	fused (>5%)
Pentapetalae	fused (>5%)
Superasteridae	fused (>5%)
Asteridae	fused (>5%)
Lamiidae	fused (>5%)
Campanulidae	fused (>5%)
Superrosidae	fused (>5%)
Rosidae	fused (>5%)
Malvidae	fused (>5%)
Fabidae	fused (>5%)

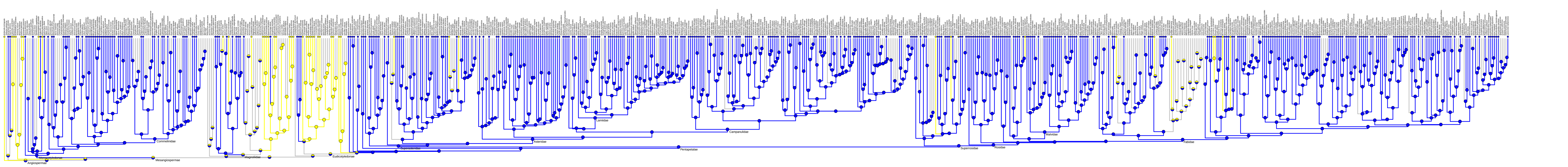

ML ancestral state reconstruction using rayDISC (R:corHMM)
403_A. Fusion of ovaries (binary) (D2c), ARDeq model

free (<5%)
fused (>5%)

Node	ML state	Prob
Angiospermae	free (<5%)	1
Mesangiospermae	free (<5%)	1
Magnoliidae	free (<5%)	0.9999
Monocotyledonae	free (<5%)	0.928
Eudicotyledonae	free (<5%)	0.9994
Commelinidae	fused (>5%)	0.9999
Pentapetalae	fused (>5%)	0.9998
Superasteridae	fused (>5%)	0.9999
Asteridae	fused (>5%)	1
Lamiidae	fused (>5%)	1
Campanulidae	fused (>5%)	1
Superrosidae	fused (>5%)	1
Rosidae	fused (>5%)	1
Malvidae	fused (>5%)	1
Fabidae	fused (>5%)	0.9998

Model	LogL	Npar	AIC	AICc	DeltaAICc	w	q01	q10
ARD	−94.56	2	193.13	193.14	1.39	0.33	0.0052	5e−04
ARDeq**	−93.87	2	191.74	191.76	0	0.66	0.0052	5e−04
ER	−102.62	1	207.24	207.25	15.49	0	8e−04	8e−04
UNI01	−98.62	1	199.23	199.24	7.48	0.02	0.0115	
UNI10	−106.61	1	215.22	215.22	23.47	0		9e−04

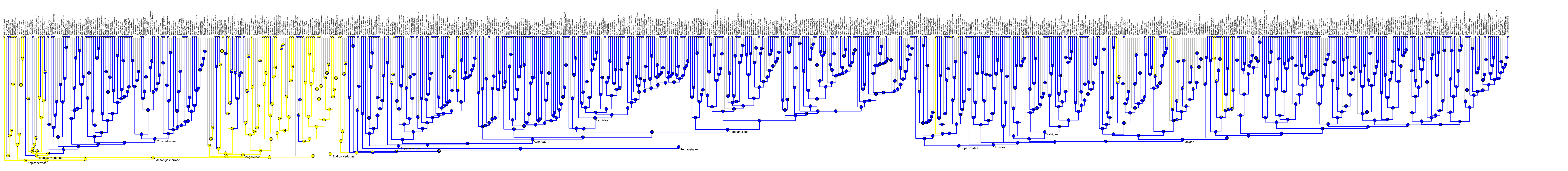

MP ancestral state reconstruction using ancestral.pars
(R:phangorn)
411_A. Number of ovules per functional carpel (3−state) (D2c), 119 steps

- one (1)
- two (2)
- three or more (>2)

Node	MP state(s)
Angiospermae	one (1)
Mesangiospermae	one (1)
Magnoliidae	one (1)
Monocotyledonae	three or more (>2)
Eudicotyledonae	one (1) / two (2) / three or more (>2)
Commelinidae	one (1)
Pentapetalae	one (1) / two (2)
Superasteridae	one (1) / two (2)
Asteridae	one (1)
Lamiidae	one (1) / two (2) / three or more (>2)
Campanulidae	one (1)
Superrosidae	two (2)
Rosidae	two (2)
Malvidae	two (2) / three or more (>2)
Fabidae	two (2)

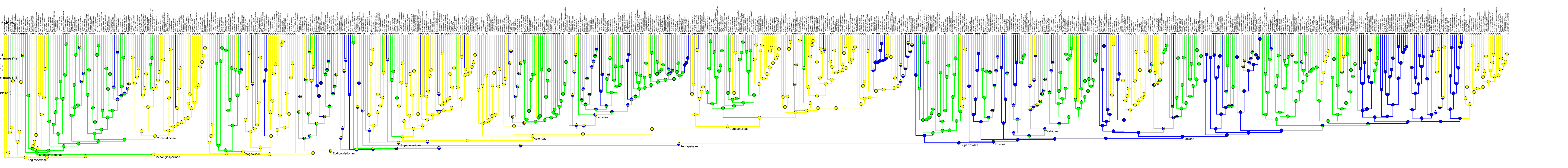

ML ancestral state reconstruction using rayDISC (R:corHMM)
411_A. Number of ovules per functional carpel (3−state) (D2c), ARDeq model

- one (1)
- two (2)
- three or more (>2)

Node	ML state	Prob
Angiospermae	three or more (>2)	1
Mesangiospermae	three or more (>2)	1
Magnoliidae	three or more (>2)	0.9998
Monocotyledonae	three or more (>2)	1
Eudicotyledonae	three or more (>2)	1
Commelinidae	three or more (>2)	0.9218
Pentapetalae	three or more (>2)	1
Superasteridae	three or more (>2)	0.9998
Asteridae	three or more (>2)	0.9994
Lamiidae	three or more (>2)	1
Campanulidae	three or more (>2)	0.9988
Superrosidae	three or more (>2)	1
Rosidae	three or more (>2)	1
Malvidae	three or more (>2)	0.9997
Fabidae	three or more (>2)	1

Model	LogL	Npar	AIC	AICc	DeltaAICc	w	q01	···
ARD	−382.96	6	777.92	778.02	2.19	0.25	0.0014	···
ARDeq**	−381.87	6	775.73	775.84	0	0.75	0.0014	···
ER	−399.09	1	800.18	800.19	24.35	0	0.0029	···
SYM	−398.69	3	803.38	803.41	27.57	0	0.0024	···
SYMeq	−397.78	3	801.56	801.59	25.75	0	0.0025	···
ORD	−398.46	4	804.92	804.97	29.14	0	8e−04	···
ORDeq	−397.37	4	802.74	802.79	26.95	0	8e−04	···
ORDSYM	−411.64	2	827.28	827.29	51.45	0	0.0055	···
ORDSYMeq	−410.71	2	825.43	825.44	49.6	0	0.0055	···
ORDER	−412.63	1	827.26	827.27	51.43	0	0.0064	···

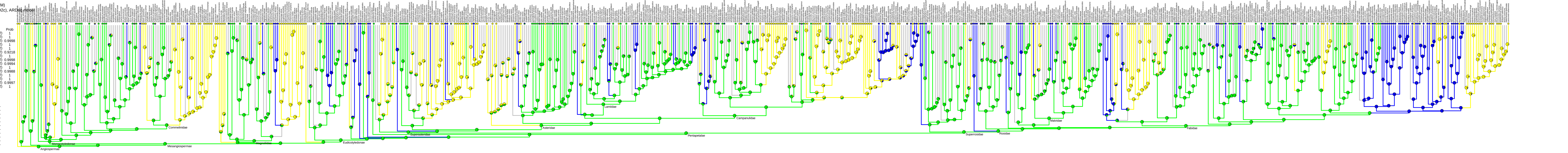

Supplement: Supplementary Data 17 [file ncomms16047-s18.pdf]
