# Supplementary material for: The ancestral flower of angiosperms and its early diversification
Source: Nat Commun. 2017 Aug 1;8:16047. doi: 10.1038/ncomms16047 (PMC5543309; doi:10.1038/ncomms16047)

MP ancestral state reconstruction using ancestral.pars
(R:phangorn)
100_A. Functional sex of flowers (D2d), 97 steps

- bisexual
- unisexual

Node	MP state(s)
Angiospermae	bisexual / unisexual
Mesangiospermae	bisexual
Magnoliidae	bisexual
Monocotyledonae	bisexual
Eudicotyledonae	bisexual
Commelinidae	bisexual
Pentapetalae	bisexual
Superasteridae	bisexual
Asteridae	bisexual
Lamiidae	bisexual
Campanulidae	bisexual
Superrosidae	bisexual
Rosidae	bisexual
Malvidae	bisexual
Fabidae	bisexual

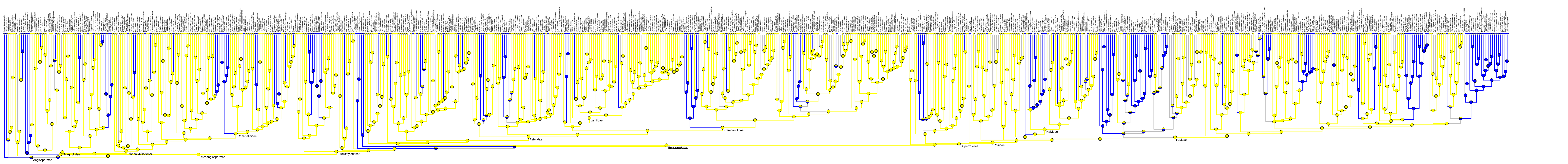

ML ancestral state reconstruction using rayDISC (R:corHMM)
100_A. Functional sex of flowers (D2d), ARDeq model

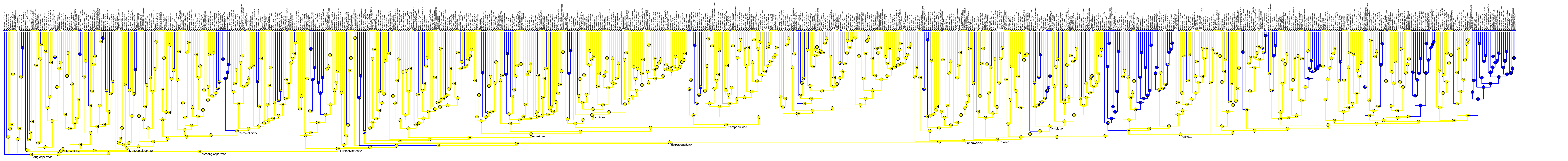

Node	ML state	Prob
Angiospermae	bisexual	1
Mesangiospermae	bisexual	1
Magnoliidae	bisexual	1
Monocotyledonae	bisexual	1
Eudicotyledonae	bisexual	1
Commelinidae	bisexual	0.9999
Pentapetalae	bisexual	1
Superasteridae	bisexual	1
Asteridae	bisexual	1
Lamiidae	bisexual	1
Campanulidae	bisexual	0.9996
Superrosidae	bisexual	1
Rosidae	bisexual	1
Malvidae	bisexual	0.9997
Fabidae	bisexual	1

Model	LogL	Npar	AIC	AICc	DeltaAICc	w	q01	q10
ARD	−313.25	2	630.5	630.52	1.38	0.23	0.003	7e−04
ARDeq*	−312.56	2	629.12	629.14	0	0.46	0.003	7e−04
ER	−316.07	1	634.13	634.14	5	0.04	0.0029	0.0029
UNI01	−314.06	1	630.12	630.12	0.99	0.28	0.003	
UNI10	−354.64	1	711.27	711.28	82.14	0		0.0118

MP ancestral state reconstruction using ancestral.pars
(R:phangorn)
100_B. Structural sex of flowers (D2d), 56 steps

- bisexual
- unisexual

Node	MP state(s)
Angiospermae	bisexual
Mesangiospermae	bisexual
Magnoliidae	bisexual
Monocotyledonae	bisexual
Eudicotyledonae	bisexual
Commelinidae	bisexual
Pentapetalae	bisexual
Superasteridae	bisexual
Asteridae	bisexual
Lamiidae	bisexual
Campanulidae	bisexual
Superrosidae	bisexual
Rosidae	bisexual
Malvidae	bisexual
Fabidae	bisexual

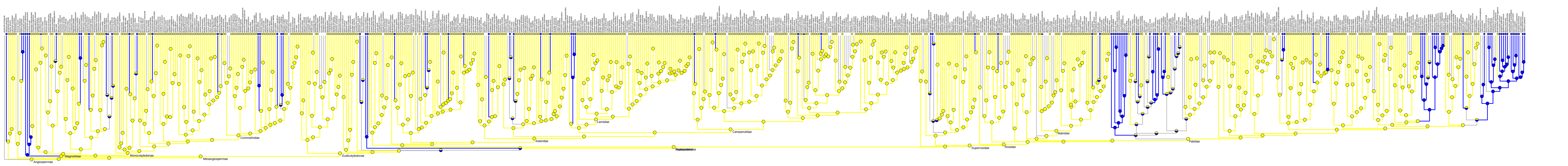

ML ancestral state reconstruction using rayDISC (R:corHMM)
100_B. Structural sex of flowers (D2d), ARDeq model

- bisexual
- unisexual

Node	ML state	Prob
Angiospermae	bisexual	1
Mesangiospermae	bisexual	1
Magnoliidae	bisexual	1
Monocotyledonae	bisexual	1
Eudicotyledonae	bisexual	1
Commelinidae	bisexual	1
Pentapetalae	bisexual	1
Superasteridae	bisexual	1
Asteridae	bisexual	1
Lamiidae	bisexual	0.9999
Campanulidae	bisexual	1
Superrosidae	bisexual	1
Rosidae	bisexual	1
Malvidae	bisexual	1
Fabidae	bisexual	0.9975

Model	LogL	Npar	AIC	AICc	DeltaAICc	w	q01	q10
ARD	−209.05	2	422.1	422.11	1.36	0.28	0.0015	0.004
ARDeq**	−208.37	2	420.74	420.75	0	0.56	0.0015	0.004
ER	−210.62	1	423.23	423.24	2.48	0.16	0.0016	0.0016
UNI01	−223.81	1	449.61	449.62	28.87	0	0.0017	
UNI10	−230.41	1	462.81	462.82	42.06	0		0.0165

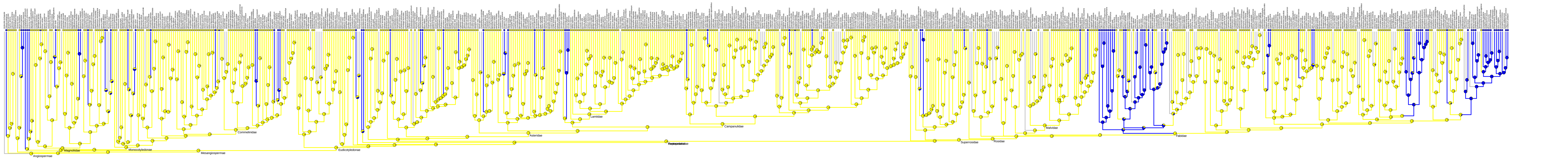

MP ancestral state reconstruction using ancestral.pars
(R:phangorn)
102_B. Ovary position (binary) (D2d), 78 steps

- superior (yellow)
- inferior (blue)

Node	MP state(s)
Angiospermae	superior
Mesangiospermae	superior
Magnoliidae	superior
Monocotyledonae	superior
Eudicotyledonae	superior
Commelinidae	superior
Pentapetalae	superior
Superasteridae	superior
Asteridae	superior
Lamiidae	superior
Campanulidae	superior
Superrosidae	superior / inferior
Rosidae	superior / inferior
Malvidae	superior
Fabidae	superior

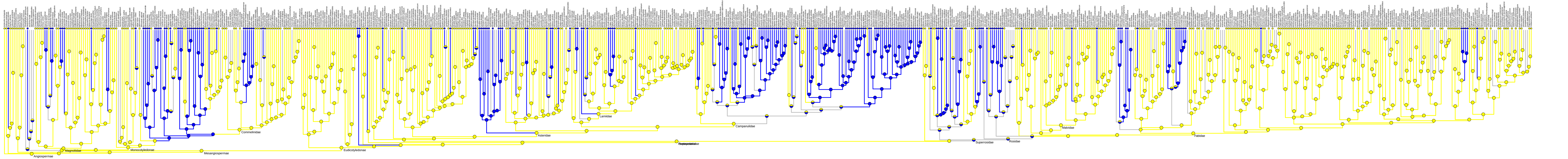

ML ancestral state reconstruction using rayDISC (R:corHMM)
102_B. Ovary position (binary) (D2d), ARDeq model

- superior (yellow)
- inferior (blue)

Node	ML state	Prob
Angiospermae	superior	1
Mesangiospermae	superior	1
Magnoliidae	superior	0.9999
Monocotyledonae	superior	0.9994
Eudicotyledonae	superior	1
Commelinidae	superior	0.8973
Pentapetalae	superior	1
Superasteridae	superior	1
Asteridae	superior	0.9873
Lamiidae	superior	0.9886
Campanulidae	superior	0.8484
Superrosidae	superior	0.9952
Rosidae	superior	0.9951
Malvidae	superior	1
Fabidae	superior	0.9999

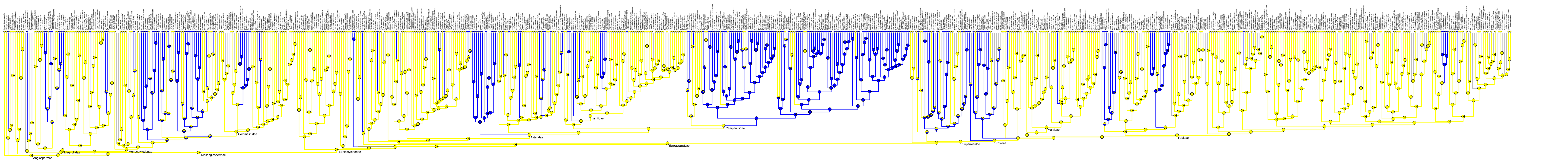

Model	LogL	Npar	AIC	AICc	DeltaAICc	w	q01	q10
ARD	−271.35	2	546.71	546.72	1.38	0.21	0.0022	0.0032
ARDeq*	−270.66	2	545.32	545.34	0	0.41	0.0022	0.0032
ER	−271.73	1	545.46	545.47	0.13	0.38	0.0023	0.0023
UNI01	−280.69	1	563.39	563.39	18.05	0	0.0027	
UNI10	−294.17	1	590.34	590.34	45	0		0.0098

MP ancestral state reconstruction using ancestral.pars
(R:phangorn)
201_A. Perianth presence (D2c), 21 steps

- absent
- present

Node	MP state(s)
Angiospermae	present
Mesangiospermae	present
Magnoliidae	present
Monocotyledonae	present
Eudicotyledonae	present
Commelinidae	present
Pentapetalae	present
Superasteridae	present
Asteridae	present
Lamiidae	present
Campanulidae	present
Superrosidae	present
Rosidae	present
Malvidae	present
Fabidae	present

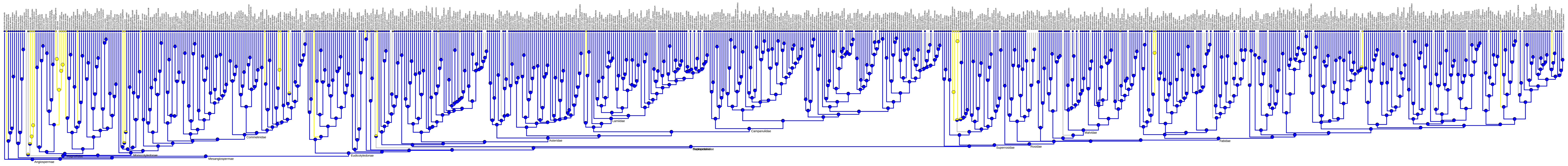

ML ancestral state reconstruction using rayDISC (R:corHMM)
201_A. Perianth presence (D2c), UNI10 model

- absent
- present

Node	ML state	Prob
Angiospermae	present	1
Mesangiospermae	present	1
Magnoliidae	present	1
Monocotyledonae	present	1
Eudicotyledonae	present	1
Commelinidae	present	1
Pentapetalae	present	1
Superasteridae	present	1
Asteridae	present	1
Lamiidae	present	1
Campanulidae	present	1
Superrosidae	present	1
Rosidae	present	1
Malvidae	present	1
Fabidae	present	1

Model	LogL	Npar	AIC	AICc	DeltaAICc	w	q01	q10
ARD	−97.4	2	198.81	198.82	2.01	0.13	0	5e−04
ARDeq	−96.71	2	197.42	197.44	0.62	0.25	0	5e−04
ER	−97.65	1	197.3	197.31	0.5	0.27	5e−04	5e−04
UNI01	−109.2	1	220.4	220.41	23.59	0	0.0221	
UNI10*	−97.4	1	196.81	196.81	0	0.35		5e−04

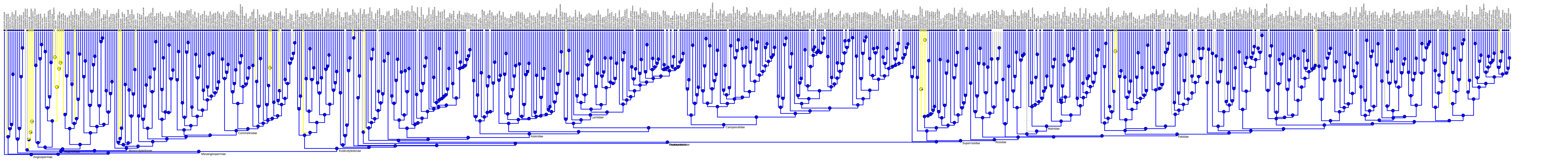

MP ancestral state reconstruction using ancestral.pars
(R:phangorn)
201_B. Number of perianth parts (3−state) (D2c), 86 steps

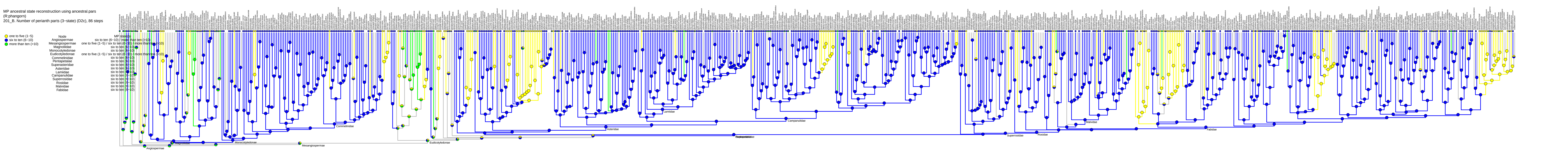

ML ancestral state reconstruction using rayDISC (R:corHMM)
201_B. Number of perianth parts (3−state) (D2c), ARDeq model

- one to five (1−5) (yellow)
- six to ten (6−10) (blue)
- more than ten (>10) (green)

Node	ML state	Prob
Angiospermae	more than ten (>10)	1
Mesangiospermae	more than ten (>10)	1
Magnoliidae	more than ten (>10)	0.9997
Monocotyledonae	six to ten (6−10)	0.5913
Eudicotyledonae	more than ten (>10)	0.9978
Commelinidae	six to ten (6−10)	0.9999
Pentapetalae	six to ten (6−10)	0.9946
Superasteridae	six to ten (6−10)	0.9946
Asteridae	six to ten (6−10)	0.9999
Lamiidae	six to ten (6−10)	0.9999
Campanulidae	six to ten (6−10)	1
Superrosidae	six to ten (6−10)	0.999
Rosidae	six to ten (6−10)	0.9993
Malvidae	six to ten (6−10)	0.9999
Fabidae	six to ten (6−10)	0.9992

Model	LogL	Npar	AIC	AICc	DeltaAICc	w	q01	···
ARD	−325.6	6	663.19	663.3	2.2	0.25	0.004	···
ARDeq**	−324.5	6	661	661.1	0	0.74	0.004	···
ER	−350.6	1	703.21	703.21	42.11	0	0.0013	···
SYM	−339.65	3	685.3	685.33	24.23	0	0.002	···
SYMeq	−338.58	3	683.16	683.19	22.08	0	0.002	···
ORD	−331.92	4	671.85	671.9	10.8	0	0.0041	···
ORDeq	−330.83	4	669.65	669.71	8.6	0.01	0.0041	···
ORDSYM	−342.6	2	689.2	689.22	28.11	0	0.0021	···
ORDSYMeq	−341.54	2	687.08	687.1	25.99	0	0.0021	···
ORDER	−351.99	1	705.98	705.98	44.88	0	0.0014	···

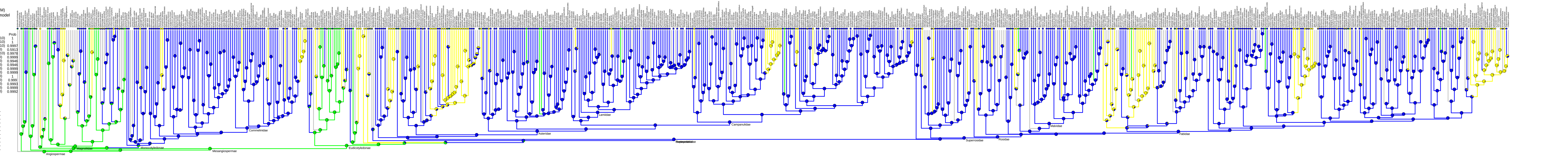

MP ancestral state reconstruction using ancestral.pars
(R:phangorn)
201_C. Number of perianth parts (binary) (D2c), 69 steps

- one to six (1−6)
- more than six (>6)

Node	MP state(s)
Angiospermae	more than six (>6)
Mesangiospermae	one to six (1−6)
Magnoliidae	one to six (1−6) / more than six (>6)
Monocotyledonae	one to six (1−6)
Eudicotyledonae	one to six (1−6)
Commelinidae	one to six (1−6)
Pentapetalae	more than six (>6)
Superasteridae	more than six (>6)
Asteridae	more than six (>6)
Lamiidae	more than six (>6)
Campanulidae	more than six (>6)
Superrosidae	more than six (>6)
Rosidae	more than six (>6)
Malvidae	more than six (>6)
Fabidae	more than six (>6)

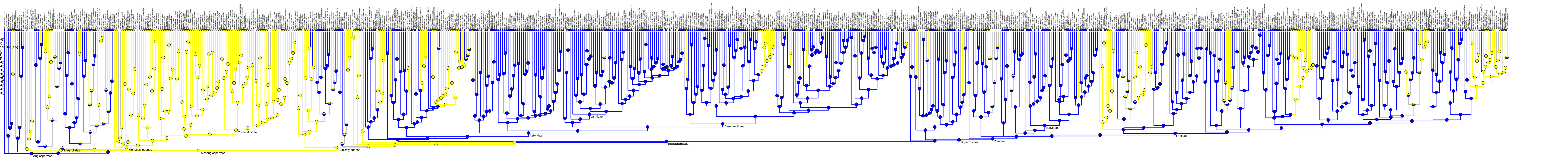

ML ancestral state reconstruction using rayDISC (R:corHMM)
201_C. Number of perianth parts (binary) (D2c), ARDeq model

- one to six (1−6)
- more than six (>6)

Node	ML state	Prob
Angiospermae	more than six (>6)	1
Mesangiospermae	more than six (>6)	1
Magnoliidae	more than six (>6)	0.9999
Monocotyledonae	one to six (1−6)	0.8097
Eudicotyledonae	more than six (>6)	1
Commelinidae	one to six (1−6)	1
Pentapetalae	more than six (>6)	1
Superasteridae	more than six (>6)	1
Asteridae	more than six (>6)	1
Lamiidae	more than six (>6)	1
Campanulidae	more than six (>6)	1
Superrosidae	more than six (>6)	1
Rosidae	more than six (>6)	1
Malvidae	more than six (>6)	1
Fabidae	more than six (>6)	1

Model	LogL	Npar	AIC	AICc	DeltaAICc	w	q01	q10
ARD	−248.39	2	500.79	500.8	1.39	0.33	6e−04	0.0026
ARDeq**	−247.7	2	499.4	499.42	0	0.65	6e−04	0.0026
ER	−254.88	1	511.75	511.76	12.34	0	0.0022	0.0022
UNI01	−322.49	1	646.98	646.98	147.57	0	0.0081	
UNI10	−252.26	1	506.52	506.52	7.1	0.02		0.0029

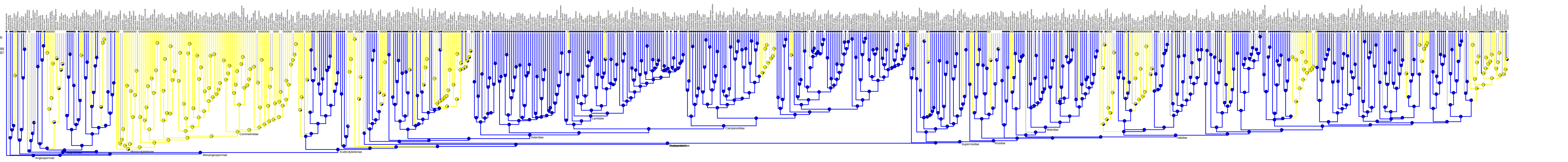

MP ancestral state reconstruction using ancestral.pars
(R:phangorn)
230_A. Perianth phyllotaxy (binary) (D2d), 16 steps

- whorled (yellow)
- spiral (blue)

Node	MP state(s)
Angiospermae	whorled / spiral
Mesangiospermae	whorled
Magnoliidae	whorled
Monocotyledonae	whorled
Eudicotyledonae	whorled
Commelinidae	whorled
Pentapetalae	whorled
Superasteridae	whorled
Asteridae	whorled
Lamiidae	whorled
Campanulidae	whorled
Superrosidae	whorled
Rosidae	whorled
Malvidae	whorled
Fabidae	whorled

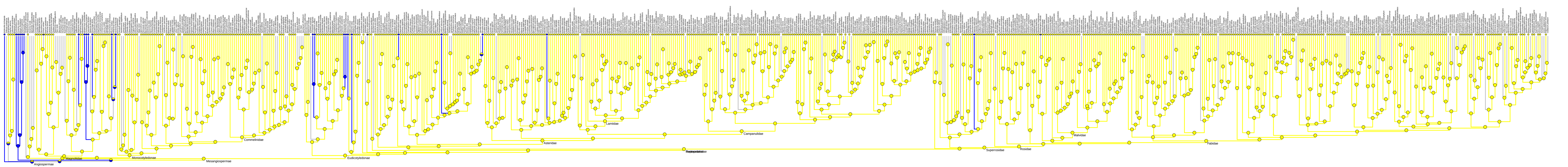

ML ancestral state reconstruction using rayDISC (R:corHMM)
230_A. Perianth phyllotaxy (binary) (D2d), ARD model

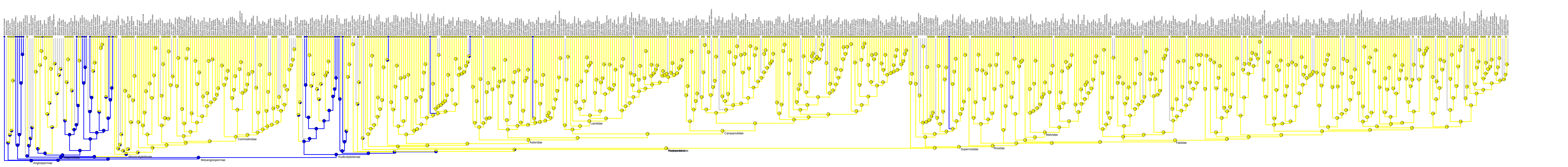

- whorled
- spiral

Node	ML state	Prob
Angiospermae	spiral	1
Mesangiospermae	spiral	1
Magnoliidae	spiral	0.9985
Monocotyledonae	whorled	0.5128
Eudicotyledonae	spiral	0.9998
Commelinidae	whorled	0.9997
Pentapetalae	whorled	0.9777
Superasteridae	whorled	0.9777
Asteridae	whorled	0.9995
Lamiidae	whorled	1
Campanulidae	whorled	1
Superrosidae	whorled	0.9916
Rosidae	whorled	0.9945
Malvidae	whorled	1
Fabidae	whorled	1

Model	LogL	Npar	AIC	AICc	DeltaAICc	w	q01	q10
ARD***	−70.5	2	145	145.01	0	0.99	2e−04	0.0111
ARDeq	−77.64	2	159.29	159.3	14.29	0	4e−04	2e−04
ER	−78.3	1	158.6	158.61	13.6	0	4e−04	4e−04
UNI01	−78.36	1	158.72	158.73	13.71	0	4e−04	
UNI10	−77.59	1	157.18	157.18	12.17	0		0.0185

MP ancestral state reconstruction using ancestral.pars
(R:phangorn)
231_A. Number of perianth whorls (D2c), 73 steps

- one (1)
- two (2)
- more than two (>2)

Node	MP state(s)
Angiospermae	more than two (>2)
Mesangiospermae	more than two (>2)
Magnoliidae	more than two (>2)
Monocotyledonae	two (2)
Eudicotyledonae	more than two (>2)
Commelinidae	two (2)
Pentapetalae	two (2)
Superasteridae	two (2)
Asteridae	two (2)
Lamiidae	two (2)
Campanulidae	two (2)
Superrosidae	two (2)
Rosidae	two (2)
Malvidae	two (2)
Fabidae	two (2)

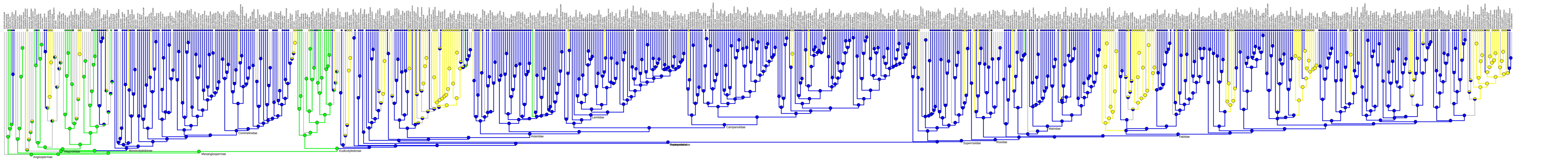

ML ancestral state reconstruction using rayDISC (R:corHMM)
231_A. Number of perianth whorls (D2c), ARDeq model

one (1)
two (2)
more than two (>2)

Node	ML state	Prob
Angiospermae	more than two (>2)	1
Mesangiospermae	more than two (>2)	1
Magnoliidae	more than two (>2)	1
Monocotyledonae	two (2)	0.7319
Eudicotyledonae	more than two (>2)	0.9982
Commelinidae	two (2)	1
Pentapetalae	two (2)	0.9998
Superasteridae	two (2)	0.9998
Asteridae	two (2)	0.9998
Lamiidae	two (2)	0.9999
Campanulidae	two (2)	1
Superrosidae	two (2)	1
Rosidae	two (2)	1
Malvidae	two (2)	0.9999
Fabidae	two (2)	0.9976

Model	LogL	Npar	AIC	AICc	DeltaAICc	w	q01	···
ARD	−269.29	6	550.58	550.69	2.2	0.25	0.0047	···
ARDeq**	−268.19	6	548.39	548.49	0	0.75	0.0047	···
ER	−310.86	1	623.72	623.73	75.23	0	0.001	···
SYM	−293.14	3	592.29	592.32	43.82	0	0.0018	···
SYMeq	−292.16	3	590.32	590.35	41.86	0	0.0018	···
ORD	−276.93	4	561.85	561.91	13.41	0	0.0045	···
ORDeq	−275.83	4	559.66	559.71	11.21	0	0.0045	···
ORDSYM	−298.57	2	601.14	601.16	52.67	0	0.0019	···
ORDSYMeq	−297.51	2	599.02	599.04	50.54	0	0.0019	···
ORDER	−313.4	1	628.81	628.81	80.32	0	0.0012	···

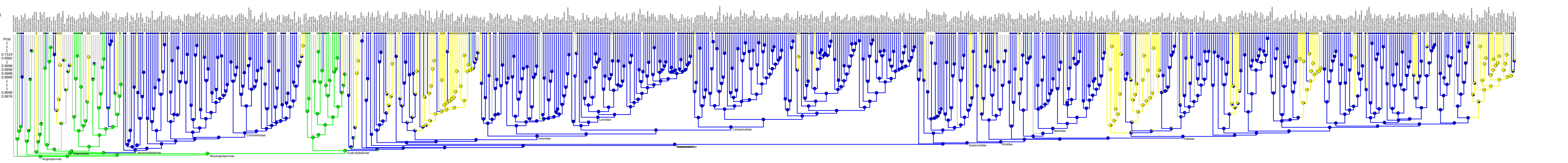

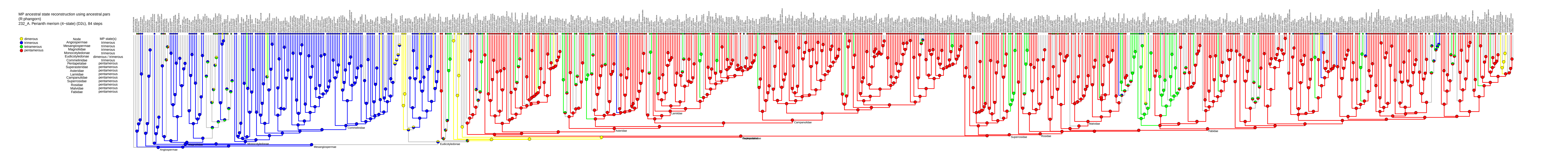
MP ancestral state reconstruction using ancestral.pars
(R:phangorn)
232_A. Perianth merism (4−state) (D2c), 84 steps
dimerous
trimerous
tetramerous
pentamerous
Node
Angiospermae
Mesangiospermae
Magnoliidae
Monocotyledonae
Eudicotyledonae
Commelinidae
Pentapetalae
Superasteridae
Asteridae
Lamiidae
Campanulidae
Superrosidae
Rosidae
Malvidae
Fabidae
MP state(s)
trimerous
trimerous
trimerous
trimerous
dimerous / trimerous
trimerous
pentamerous
pentamerous
pentamerous
pentamerous
pentamerous
pentamerous
pentamerous
pentamerous
pentamerous
Angiospermae
Mesangiospermae
Monocotyledonae
Commelinidae
Eudicotyledonae
Asteridae
Lamiidae
Campanulidae
Superrosidae
Rosidae
Malvidae
Fabidae

ML ancestral state reconstruction using rayDISC (R:corHMM)
232_A. Perianth merism (4−state) (D2c), SYMeq model

- dimerous
- trimerous
- tetramerous
- pentamerous

Node	ML state	Prob
Angiospermae	trimerous	1
Mesangiospermae	trimerous	1
Magnoliidae	trimerous	1
Monocotyledonae	trimerous	1
Eudicotyledonae	trimerous	0.9742
Commelinidae	trimerous	1
Pentapetalae	pentamerous	0.9998
Superasteridae	pentamerous	0.9998
Asteridae	pentamerous	1
Lamiidae	pentamerous	1
Campanulidae	pentamerous	1
Superrosidae	pentamerous	1
Rosidae	pentamerous	1
Malvidae	pentamerous	1
Fabidae	pentamerous	1

Model	LogL	Npar	AIC	AICc	DeltaAICc	w	q01	...
ARD	−321.85	12	667.7	668.1	7.62	0.02	6e−04	...
ARDeq	−320.5	12	665	665.4	4.92	0.06	5e−04	...
ER	−360.15	1	722.29	722.3	61.81	0	8e−04	...
SYM	−325.57	6	663.14	663.25	2.76	0.19	0.0013	...
SYMeq**	−324.19	6	660.38	660.49	0	0.74	0.0013	...
ORD	−336.7	6	685.4	685.51	25.02	0	0	...
ORDeq	−336.17	6	684.33	684.44	23.95	0	0	...
ORDSYM	−344.42	3	694.85	694.88	34.39	0	0.002	...
ORDSYMeq	−343.05	3	692.1	692.14	31.65	0	0.002	...
ORDER	−345.93	1	693.86	693.86	33.38	0	0.0025	...

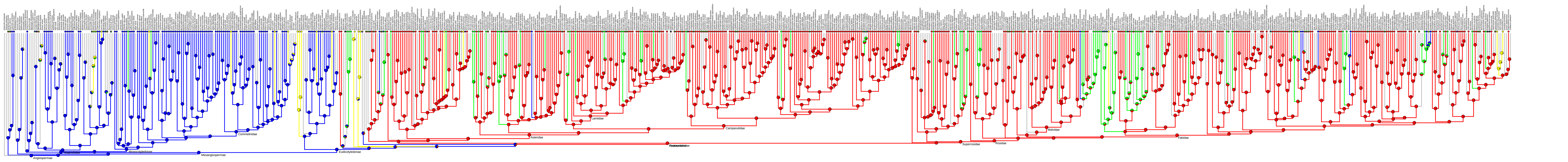

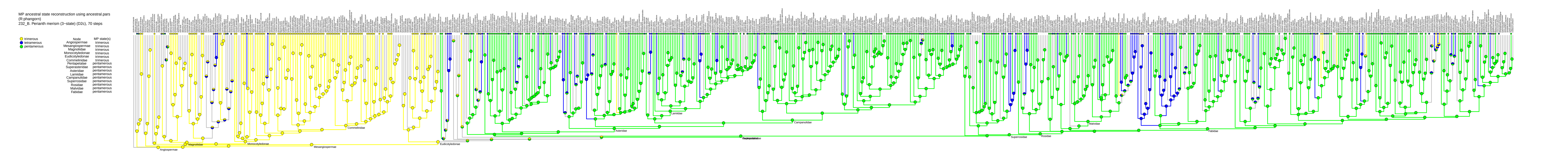

MP ancestral state reconstruction using ancestral.pars
(R:phangorn)
232_B. Perianth merism (3−state) (D2c), 70 steps
trimerous
tetramerous
pentamerous
Node
MP state(s)
Angiospermae trimerous
Mesangiospermae trimerous
Magnoliidae trimerous
Monocotyledonae trimerous
Eudicotyledonae trimerous
Commelinidae trimerous
Pentapetalae pentamerous
Superasteridae pentamerous
Asteridae pentamerous
Lamiidae pentamerous
Campanulidae pentamerous
Superrosidae pentamerous
Rosidae pentamerous
Malvidae pentamerous
Fabidae pentamerous
Angiospermae
Magnoliidae
Monocotyledonae
Mesangiospermae
Commelinidae
Eudicotyledonae
Asteridae
Lamiidae
Campanulidae
Superrosidae
Rosidae
Malvidae
Fabidae

ML ancestral state reconstruction using rayDISC (R:corHMM)
232_B. Perianth merism (3−state) (D2c), SYMeq model

- trimerous
- tetramerous
- pentamerous

Node	ML state	Prob
Angiospermae	trimerous	1
Mesangiospermae	trimerous	1
Magnoliidae	trimerous	1
Monocotyledonae	trimerous	1
Eudicotyledonae	trimerous	0.9831
Commelinidae	trimerous	1
Pentapetalae	pentamerous	0.9998
Superasteridae	pentamerous	0.9998
Asteridae	pentamerous	1
Lamiidae	pentamerous	1
Campanulidae	pentamerous	1
Superrosidae	pentamerous	1
Rosidae	pentamerous	1
Malvidae	pentamerous	1
Fabidae	pentamerous	1

Model	LogL	Npar	AIC	AICc	DeltaAICc	w	q01	···
ARD	−255.64	6	523.29	523.39	6.11	0.02	8e−04	···
ARDeq	−254.59	6	521.17	521.28	3.99	0.06	7e−04	···
ER	−280.83	1	563.65	563.66	46.37	0	0.0011	···
SYM	−256.72	3	519.45	519.48	2.19	0.14	0.001	···
SYMeq*	−255.63	3	517.26	517.29	0	0.43	0.001	···
ORD	−256.46	4	520.92	520.97	3.68	0.07	9e−04	···
ORDeq	−255.52	4	519.04	519.09	1.8	0.17	0.001	···
ORDSYM	−259.42	2	522.84	522.85	5.56	0.03	0.0015	···
ORDSYMeq	−258.34	2	520.69	520.7	3.41	0.08	0.0015	···
ORDER	−261.69	1	525.37	525.38	8.09	0.01	0.0023	···

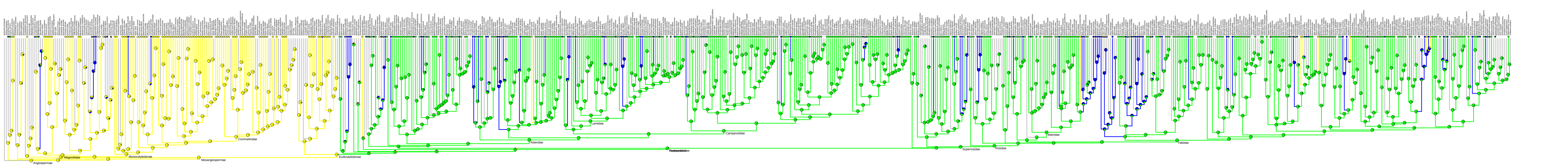

MP ancestral state reconstruction using ancestral.pars
(R:phangorn)
234_A. Perianth differentiation (binary) (D2d), 65 steps

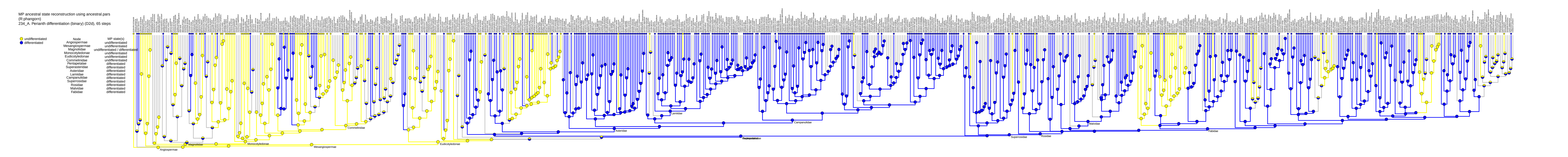

Node	MP state(s)
Angiospermae	undifferentiated
Mesangiospermae	undifferentiated
Magnoliidae	undifferentiated / differentiated
Monocotyledonae	undifferentiated
Eudicotyledonae	undifferentiated
Commelinidae	undifferentiated
Pentapetalae	differentiated
Superasteridae	differentiated
Asteridae	differentiated
Lamiidae	differentiated
Campanulidae	differentiated
Superrosidae	differentiated
Rosidae	differentiated
Malvidae	differentiated
Fabidae	differentiated

ML ancestral state reconstruction using rayDISC (R:corHMM)
234_A. Perianth differentiation (binary) (D2d), ARDeq model

- undifferentiated
- differentiated

Node	ML state	Prob
Angiospermae	undifferentiated	1
Mesangiospermae	undifferentiated	1
Magnoliidae	undifferentiated	1
Monocotyledonae	undifferentiated	1
Eudicotyledonae	undifferentiated	0.9915
Commelinidae	undifferentiated	0.9999
Pentapetalae	differentiated	0.9429
Superasteridae	differentiated	0.9429
Asteridae	differentiated	0.9977
Lamiidae	differentiated	0.9998
Campanulidae	differentiated	1
Superrosidae	differentiated	0.9913
Rosidae	differentiated	0.9945
Malvidae	differentiated	0.9988
Fabidae	differentiated	0.9865

Model	LogL	Npar	AIC	AICc	DeltaAICc	w	q01	q10
ARD	−199.82	2	403.64	403.66	1.38	0.33	0.0046	0.0015
ARDeq**	−199.13	2	402.26	402.27	0	0.67	0.0046	0.0015
ER	−209.24	1	420.48	420.48	18.21	0	0.0025	0.0025
UNI01	−228.29	1	458.58	458.59	56.32	0	0.0089	
UNI10	−233.78	1	469.55	469.56	67.28	0		0.0034

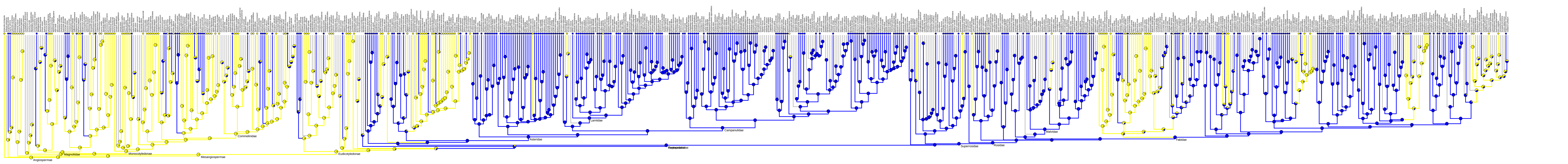

MP ancestral state reconstruction using ancestral.pars
(R:phangorn)
204_A. Fusion of perianth (D2c), 77 steps

- free (<5%)
- fused (>5%)

Node	MP state(s)
Angiospermae	free (<5%)
Mesangiospermae	free (<5%)
Magnoliidae	free (<5%)
Monocotyledonae	free (<5%)
Eudicotyledonae	free (<5%)
Commelinidae	free (<5%)
Pentapetalae	free (<5%)
Superasteridae	free (<5%)
Asteridae	free (<5%)
Lamiidae	free (<5%) / fused (>5%)
Campanulidae	fused (>5%)
Superrosidae	free (<5%)
Rosidae	free (<5%)
Malvidae	free (<5%)
Fabidae	free (<5%)

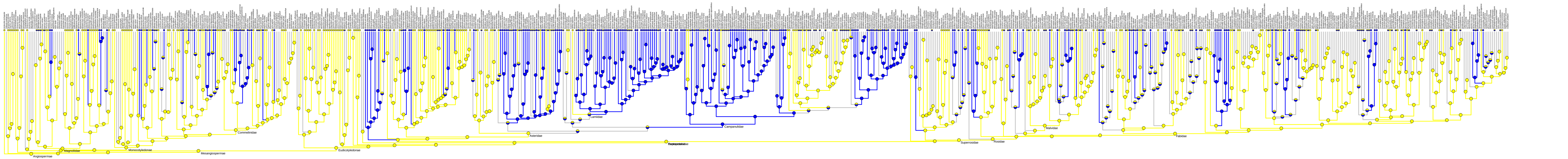

ML ancestral state reconstruction using rayDISC (R:corHMM)
204_A. Fusion of perianth (D2c), ARDeq model

- free (<5%)
- fused (>5%)

Node	ML state	Prob
Angiospermae	free (<5%)	1
Mesangiospermae	free (<5%)	1
Magnoliidae	free (<5%)	0.9999
Monocotyledonae	free (<5%)	1
Eudicotyledonae	free (<5%)	1
Commelinidae	free (<5%)	0.9991
Pentapetalae	free (<5%)	0.9994
Superasteridae	free (<5%)	0.9994
Asteridae	free (<5%)	0.8583
Lamiidae	fused (>5%)	0.9506
Campanulidae	fused (>5%)	0.9276
Superrosidae	free (<5%)	0.9994
Rosidae	free (<5%)	0.9994
Malvidae	free (<5%)	0.9983
Fabidae	free (<5%)	1

Model	LogL	Npar	AIC	AICc	DeltaAICc	w	q01	q10
ARD	−224.54	2	453.08	453.1	1.38	0.21	0.0044	0.0035
ARDeq*	−223.85	2	451.7	451.71	0	0.42	0.0044	0.0035
ER	−225.01	1	452.01	452.02	0.3	0.36	0.0042	0.0042
UNI01	−236.28	1	474.56	474.57	22.85	0	0.0049	
UNI10	−236.29	1	474.58	474.59	22.87	0		0.008

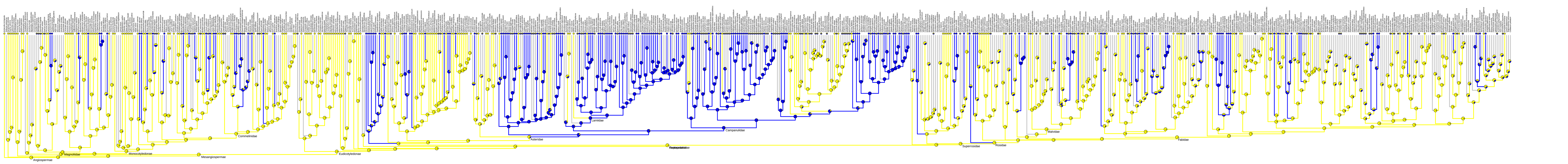

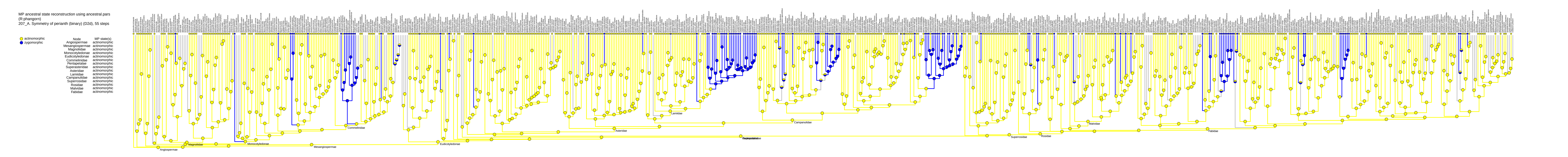
MP ancestral state reconstruction using ancestral.pars
(R:phangorn)
207_A. Symmetry of perianth (binary) (D2d), 55 steps
actinomorphic
zygomorphic
Node
MP state(s)
Angiospermae
actinomorphic
Mesangiospermae
actinomorphic
Magnoliidae
actinomorphic
Monocotyledonae
actinomorphic
Eudicotyledonae
actinomorphic
Commelinidae
actinomorphic
Pentapetalae
actinomorphic
Superasteridae
actinomorphic
Asteridae
actinomorphic
Lamiidae
actinomorphic
Campanulidae
actinomorphic
Superrosidae
actinomorphic
Rosidae
actinomorphic
Malvidae
actinomorphic
Fabidae
actinomorphic
Angiospermae
Magnoliidae
Monocotyledonae
Mesangiospermae
Commelinidae
Eudicotyledonae
Lamiidae
Asteridae
Campanulidae
Superrosidae
Rosidae
Malvidae
Fabidae

ML ancestral state reconstruction using rayDISC (R:corHMM)
207_A. Symmetry of perianth (binary) (D2d), ARDeq model

- actinomorphic
- zygomorphic

Node	ML state	Prob
Angiospermae	actinomorphic	1
Mesangiospermae	actinomorphic	1
Magnoliidae	actinomorphic	1
Monocotyledonae	actinomorphic	0.9997
Eudicotyledonae	actinomorphic	1
Commelinidae	actinomorphic	0.9976
Pentapetalae	actinomorphic	1
Superasteridae	actinomorphic	1
Asteridae	actinomorphic	1
Lamiidae	actinomorphic	0.998
Campanulidae	actinomorphic	1
Superrosidae	actinomorphic	1
Rosidae	actinomorphic	1
Malvidae	actinomorphic	0.9998
Fabidae	actinomorphic	1

Model	LogL	Npar	AIC	AICc	DeltaAICc	w	q01	q10
ARD	−209.77	2	423.55	423.56	1.37	0.33	0.0014	0.0059
ARDeq**	−209.09	2	422.17	422.19	0	0.66	0.0014	0.0059
ER	−217.62	1	437.24	437.24	15.06	0	0.0015	0.0015
UNI01	−258.94	1	519.88	519.88	97.7	0	0.0018	
UNI10	−216.82	1	435.64	435.64	13.45	0		0.0167

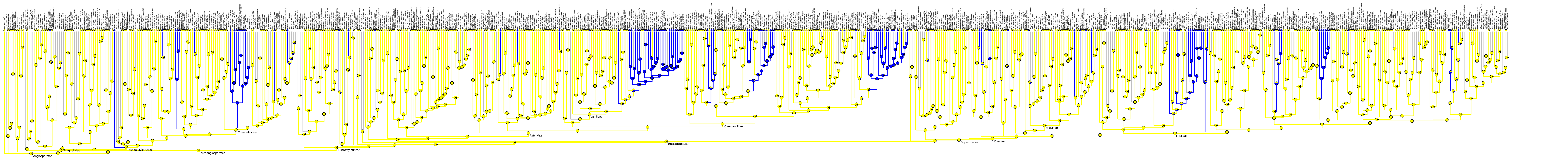

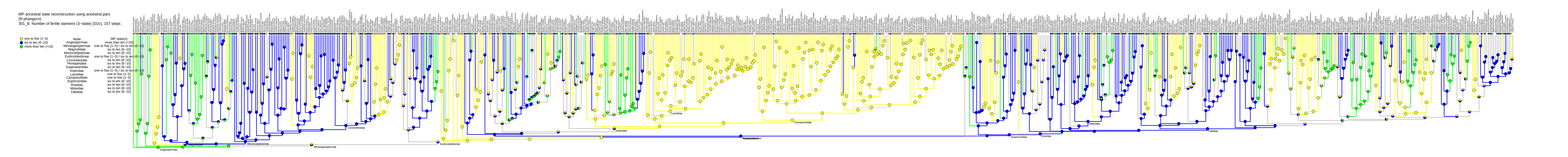
MP ancestral state reconstruction using ancestral.pars
(R:phangorn)
301_B. Number of fertile stamens (3−state) (D2c), 157 steps
one to five (1−5)
six to ten (6−10)
more than ten (>10)
Node
MP state(s)
Angiospermae more than ten (>10)
Mesangiospermae one to five (1−5) / six to ten (6−10)
Magnoliidae six to ten (6−10)
Monocotyledonae six to ten (6−10)
Eudicotyledonae one to five (1−5) / six to ten (6−10)
Commelinidae six to ten (6−10)
Pentapetalae six to ten (6−10)
Superasteridae six to ten (6−10)
Asteridae one to five (1−5) / six to ten (6−10)
Lamiidae one to five (1−5)
Campanulidae one to five (1−5)
Superrosidae six to ten (6−10)
Rosidae six to ten (6−10)
Malvidae six to ten (6−10)
Fabidae six to ten (6−10)
Angiospermae
Mesangiospermae
Monocotyledonae
Commelinidae
Eudicotyledonae
Asteridae
Lamiidae
Campanulidae
Superrosidae
Rosidae
Malvidae
Fabidae

ML ancestral state reconstruction using rayDISC (R:corHMM)
301_B. Number of fertile stamens (3−state) (D2c), ARDeq model

- one to five (1−5)
- six to ten (6−10)
- more than ten (>10)

Node	ML state	Prob
Angiospermae	more than ten (>10)	1
Mesangiospermae	more than ten (>10)	1
Magnoliidae	more than ten (>10)	0.9999
Monocotyledonae	six to ten (6−10)	0.5681
Eudicotyledonae	more than ten (>10)	1
Commelinidae	six to ten (6−10)	0.9998
Pentapetalae	more than ten (>10)	1
Superasteridae	more than ten (>10)	1
Asteridae	more than ten (>10)	0.9993
Lamiidae	one to five (1−5)	0.9994
Campanulidae	one to five (1−5)	0.9964
Superrosidae	more than ten (>10)	1
Rosidae	more than ten (>10)	0.9999
Malvidae	more than ten (>10)	0.928
Fabidae	more than ten (>10)	1

Model	LogL	Npar	AIC	AICc	DeltaAICc	w	q01	···
ARD	−495.22	6	1002.44	1002.54	2.2	0.25	4e−04	···
ARDeq**	−494.12	6	1000.24	1000.35	0	0.75	4e−04	···
ER	−538	1	1077.99	1078	77.65	0	0.0027	···
SYM	−531.78	3	1069.56	1069.59	69.24	0	0.0038	···
SYMeq	−530.98	3	1067.97	1068	67.65	0	0.0038	···
ORD	−511.87	4	1031.74	1031.79	31.45	0	8e−04	···
ORDeq	−511.35	4	1030.69	1030.74	30.4	0	8e−04	···
ORDSYM	−536.28	2	1076.56	1076.58	76.23	0	0.0043	···
ORDSYMeq	−535.41	2	1074.81	1074.83	74.48	0	0.0043	···
ORDER	−536.3	1	1074.61	1074.61	74.26	0	0.0044	···

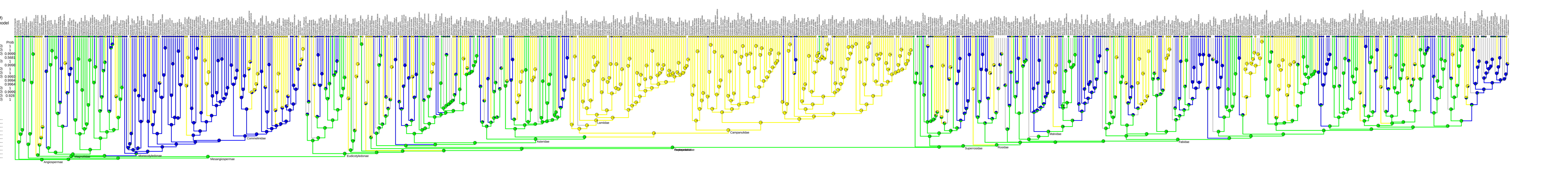

MP ancestral state reconstruction using ancestral.pars
(R:phangorn)
301_C. Number of fertile stamens (binary) (D2c), 93 steps

- one to six (1−6)
- more than six (>6)

Node	MP state(s)
Angiospermae	more than six (>6)
Mesangiospermae	one to six (1−6)
Magnoliidae	one to six (1−6) / more than six (>6)
Monocotyledonae	one to six (1−6)
Eudicotyledonae	one to six (1−6)
Commelinidae	one to six (1−6)
Pentapetalae	more than six (>6)
Superasteridae	more than six (>6)
Asteridae	more than six (>6)
Lamiidae	one to six (1−6)
Campanulidae	one to six (1−6)
Superrosidae	more than six (>6)
Rosidae	more than six (>6)
Malvidae	more than six (>6)
Fabidae	more than six (>6)

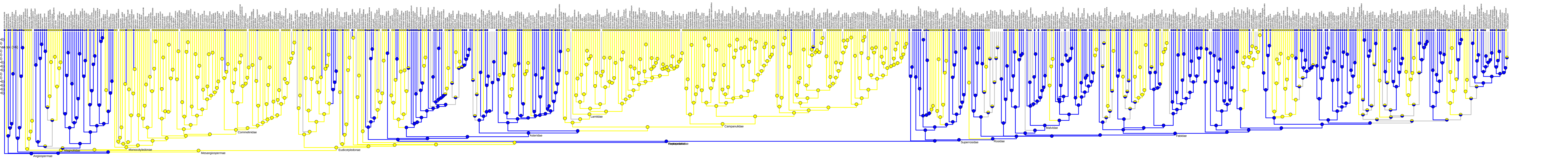

ML ancestral state reconstruction using rayDISC (R:corHMM)
301_C. Number of fertile stamens (binary) (D2c), ARDeq model

- one to six (1−6)
- more than six (>6)

Node	ML state	Prob
Angiospermae	more than six (>6)	1
Mesangiospermae	more than six (>6)	1
Magnoliidae	more than six (>6)	1
Monocotyledonae	more than six (>6)	0.5187
Eudicotyledonae	more than six (>6)	0.9999
Commelinidae	one to six (1−6)	1
Pentapetalae	more than six (>6)	1
Superasteridae	more than six (>6)	1
Asteridae	more than six (>6)	0.9999
Lamiidae	one to six (1−6)	0.9986
Campanulidae	one to six (1−6)	0.9937
Superrosidae	more than six (>6)	1
Rosidae	more than six (>6)	1
Malvidae	more than six (>6)	1
Fabidae	more than six (>6)	1

Model	LogL	Npar	AIC	AICc	DeltaAICc	w	q01	q10
ARD	−291.73	2	587.45	587.47	1.39	0.33	5e−04	0.0059
ARDeq**	−291.03	2	586.07	586.08	0	0.67	5e−04	0.0059
ER	−326.45	1	654.91	654.91	68.83	0	0.0032	0.0032
UNI01	−366.73	1	735.46	735.47	149.39	0	0.0046	
UNI10	−327.04	1	656.08	656.08	70	0		0.0073

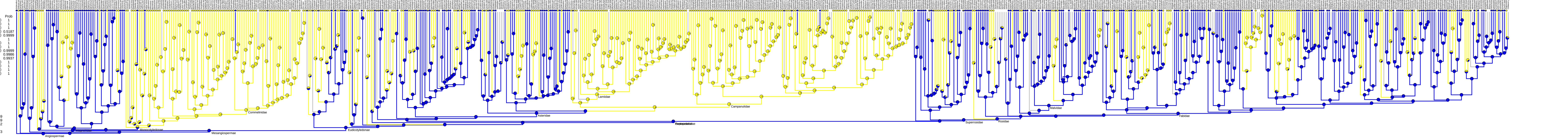

MP ancestral state reconstruction using ancestral.pars
(R:phangorn)
330_A. Androecium structural phyllotaxy (binary) (D2d), 12 steps

Node	MP state(s)
Angiospermae	whorled / spiral
Mesangiospermae	whorled
Magnoliidae	whorled
Monocotyledonae	whorled
Eudicotyledonae	whorled
Commelinidae	whorled
Pentapetalae	whorled
Superasteridae	whorled
Asteridae	whorled
Lamiidae	whorled
Campanulidae	whorled
Superrosidae	whorled
Rosidae	whorled
Malvidae	whorled
Fabidae	whorled

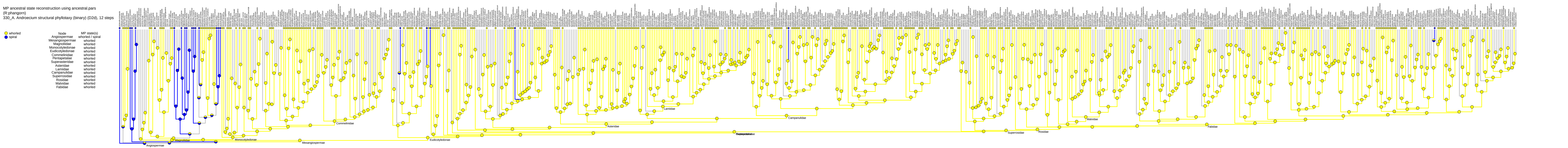

ML ancestral state reconstruction using rayDISC (R:corHMM)
330_A. Androecium structural phyllotaxy (binary) (D2d), ARD model

- whorled
- spiral

Node	ML state	Prob
Angiospermae	spiral	1
Mesangiospermae	spiral	0.9992
Magnoliidae	spiral	0.9982
Monocotyledonae	whorled	0.5792
Eudicotyledonae	spiral	0.9844
Commelinidae	whorled	0.9997
Pentapetalae	whorled	0.9975
Superasteridae	whorled	0.9975
Asteridae	whorled	1
Lamiidae	whorled	1
Campanulidae	whorled	1
Superrosidae	whorled	0.9996
Rosidae	whorled	0.9997
Malvidae	whorled	1
Fabidae	whorled	1

Model	LogL	Npar	AIC	AICc	DeltaAICc	w	q01	q10
ARD***	−55.27	2	114.54	114.55	0	0.97	1e−04	0.0087
ARDeq	−59.88	2	123.77	123.78	9.23	0.01	4e−04	0
ER	−60.81	1	123.62	123.62	9.07	0.01	4e−04	4e−04
UNI01	−60.58	1	123.16	123.16	8.61	0.01	4e−04	
UNI10	−76.52	1	155.04	155.04	40.49	0		0.0211

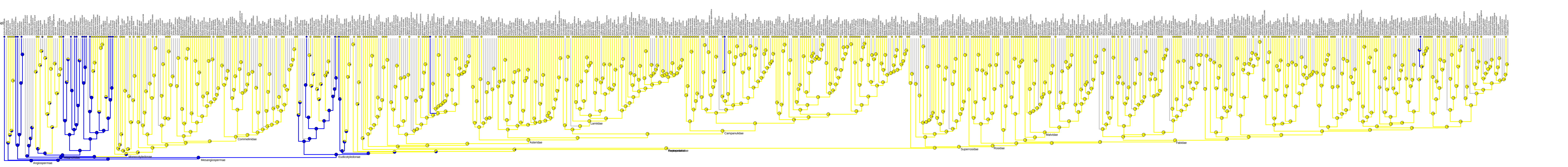

MP ancestral state reconstruction using ancestral.pars (R:phangorn)
331_A. Number of androecium structural whorls (3−state) (D2c), 76 steps

- one (1)
- two (2)
- more than two (>2)

Node	MP state(s)
Angiospermae	more than two (>2)
Mesangiospermae	more than two (>2)
Magnoliidae	more than two (>2)
Monocotyledonae	two (2)
Eudicotyledonae	more than two (>2)
Commelinidae	two (2)
Pentapetalae	one (1)
Superasteridae	one (1)
Asteridae	one (1)
Lamiidae	one (1)
Campanulidae	one (1)
Superrosidae	one (1)
Rosidae	one (1)
Malvidae	one (1) / two (2)
Fabidae	one (1)

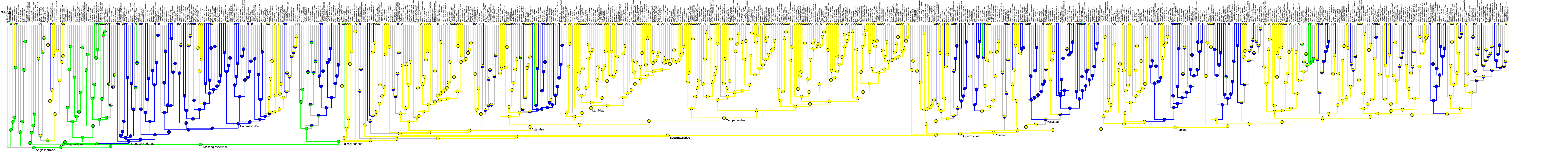

ML ancestral state reconstruction using rayDISC (R:corHMM)
331_A. Number of androecium structural whorls (3−state) (D2c), ORDeq model

- one (1)
- two (2)
- more than two (>2)

Node	ML state	Prob
Angiospermae	two (2)	1
Mesangiospermae	two (2)	0.9999
Magnoliidae	two (2)	0.9917
Monocotyledonae	two (2)	1
Eudicotyledonae	two (2)	1
Commelinidae	two (2)	1
Pentapetalae	two (2)	1
Superasteridae	two (2)	1
Asteridae	two (2)	1
Lamiidae	one (1)	0.9985
Campanulidae	one (1)	0.9927
Superrosidae	two (2)	1
Rosidae	two (2)	1
Malvidae	two (2)	1
Fabidae	two (2)	1

Model	LogL	Npar	AIC	AICc	DeltaAICc	w	q01	···
ARD	−234.55	6	481.1	481.21	2.55	0.12	0	···
ARDeq	−233.5	6	479	479.11	0.45	0.33	0	···
ER	−282.3	1	566.59	566.6	87.93	0	0.0017	···
SYM	−256.12	3	518.25	518.28	39.61	0	0.0035	···
SYMeq	−255.08	3	516.15	516.18	37.52	0	0.0035	···
ORD	−236.34	4	480.68	480.73	2.07	0.15	0	···
ORDeq*	−235.31	4	478.61	478.66	0	0.41	0	···
ORDSYM	−256.56	2	517.13	517.14	38.48	0	0.0036	···
ORDSYMeq	−255.5	2	515	515.01	36.35	0	0.0036	···
ORDER	−261.84	1	525.68	525.68	47.02	0	0.0029	···

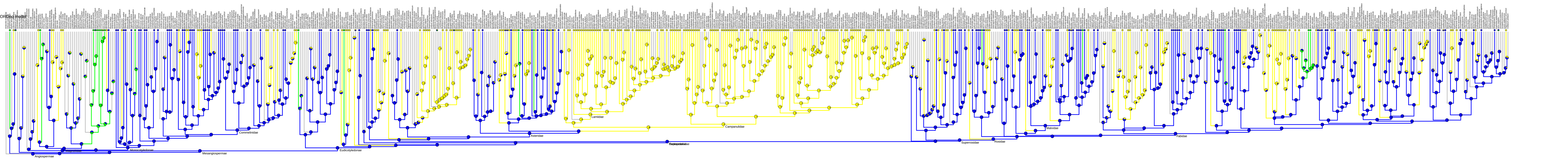

MP ancestral state reconstruction using ancestral.pars
(R:phangorn)
332_A. Androecium structural merism (4−state) (D2c), 57 steps

- dimerous
- trimerous
- tetramerous
- pentamerous

Node	MP state(s)
Angiospermae	trimerous / tetramerous / pentamerous
Mesangiospermae	trimerous / tetramerous / pentamerous
Magnoliidae	trimerous
Monocotyledonae	trimerous
Eudicotyledonae	trimerous / tetramerous / pentamerous
Commelinidae	trimerous
Pentapetalae	pentamerous
Superasteridae	pentamerous
Asteridae	pentamerous
Lamiidae	pentamerous
Campanulidae	pentamerous
Superrosidae	pentamerous
Rosidae	pentamerous
Malvidae	pentamerous
Fabidae	pentamerous

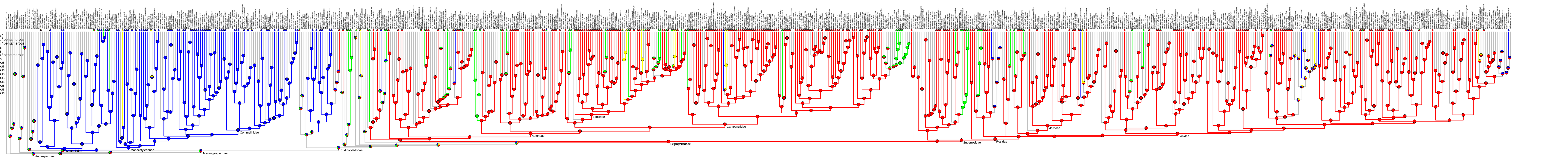

ML ancestral state reconstruction using rayDISC (R:corHMM)
332_A. Androecium structural merism (4−state) (D2c), ORD model

- dimerous (yellow)
- trimerous (blue)
- tetramerous (green)
- pentamerous (red)

Node	ML state	Prob
Angiospermae	tetramerous	0.6864
Mesangiospermae	tetramerous	0.6945
Magnoliidae	trimerous	0.678
Monocotyledonae	trimerous	0.7897
Eudicotyledonae	tetramerous	0.9312
Commelinidae	trimerous	0.8452
Pentapetalae	pentamerous	0.9708
Superasteridae	pentamerous	0.9708
Asteridae	pentamerous	0.9957
Lamiidae	pentamerous	0.9885
Campanulidae	pentamerous	0.9991
Superrosidae	pentamerous	0.9908
Rosidae	pentamerous	0.9938
Malvidae	pentamerous	0.994
Fabidae	pentamerous	0.9998

Model	LogL	Npar	AIC	AICc	DeltaAICc	w	q01	···
ARD	−223.55	12	471.09	471.49	22.1	0	0.0072	···
ARDeq	−222.25	12	468.5	468.9	19.51	0	0.0071	···
ER	−237.25	1	476.49	476.5	27.11	0	8e−04	···
SYM	−225.32	6	462.63	462.74	13.35	0	5e−04	···
SYMeq	−224.01	6	460.02	460.13	10.74	0	5e−04	···
ORD***	−218.64	6	449.28	449.39	0	0.99	15.4031	···
ORDeq	−236.42	6	484.84	484.95	35.56	0	11.9257	···
ORDSYM	−259.9	3	525.81	525.84	76.45	0	0.0021	···
ORDSYMeq	−258.72	3	523.45	523.48	74.09	0	0.0021	···
ORDER	−260.67	1	523.34	523.34	73.95	0	0.0031	···

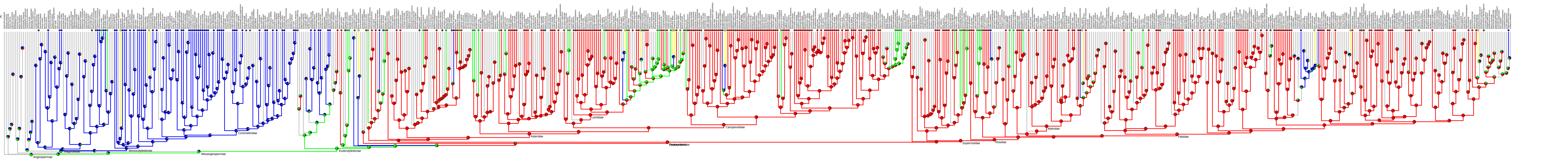

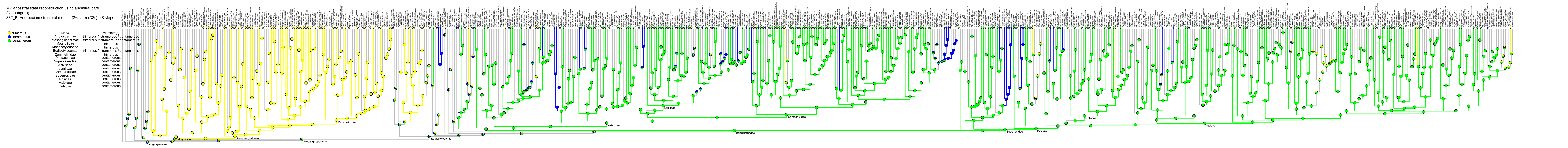

MP ancestral state reconstruction using ancestral.pars
(R:phangorn)
332_B. Androecium structural merism (3−state) (D2c), 48 steps
trimerous
tetramerous
pentamerous
Node
MP state(s)
Angiospermae trimerous / tetramerous / pentamerous
Mesangiospermae trimerous / tetramerous / pentamerous
Magnoliidae trimerous
Monocotyledonae trimerous
Eudicotyledonae trimerous / tetramerous / pentamerous
Commelinidae trimerous
Pentapetalae pentamerous
Superasteridae pentamerous
Asteridae pentamerous
Lamiidae pentamerous
Campanulidae pentamerous
Superrosidae pentamerous
Rosidae pentamerous
Malvidae pentamerous
Fabidae pentamerous
Angiospermae
Magnoliidae
Monocotyledonae
Mesangiospermae
Commelinidae
Eudicotyledonae
Asteridae
Lamiidae
Campanulidae
Superrosidae
Rosidae
Malvidae
Fabidae

ML ancestral state reconstruction using rayDISC (R:corHMM)
332_B. Androecium structural merism (3−state) (D2c), SYMeq model

- trimerous
- tetramerous
- pentamerous

Node	ML state	Prob
Angiospermae	trimerous	0.9999
Mesangiospermae	trimerous	0.9985
Magnoliidae	trimerous	1
Monocotyledonae	trimerous	1
Eudicotyledonae	trimerous	0.8633
Commelinidae	trimerous	1
Pentapetalae	pentamerous	0.9999
Superasteridae	pentamerous	0.9999
Asteridae	pentamerous	0.9997
Lamiidae	pentamerous	0.9999
Campanulidae	pentamerous	1
Superrosidae	pentamerous	1
Rosidae	pentamerous	1
Malvidae	pentamerous	1
Fabidae	pentamerous	1

Model	LogL	Npar	AIC	AICc	DeltaAICc	w	q01	···
ARD	−176.92	6	365.85	365.96	7.74	0.01	5e−04	···
ARDeq	−175.95	6	363.89	364	5.78	0.03	5e−04	···
ER	−185.99	1	373.98	373.98	15.77	0	0.0011	···
SYM	−177.11	3	360.22	360.25	2.04	0.22	5e−04	···
SYMeq**	−176.09	3	358.19	358.22	0	0.62	5e−04	···
ORD	−178.11	4	364.22	364.27	6.05	0.03	5e−04	···
ORDeq	−177.21	4	362.42	362.47	4.25	0.07	3e−04	···
ORDSYM	−186.25	2	376.49	376.51	18.29	0	0.002	···
ORDSYMeq	−185.32	2	374.64	374.66	16.44	0	0.002	···
ORDER	−186.96	1	375.91	375.92	17.7	0	0.0027	···

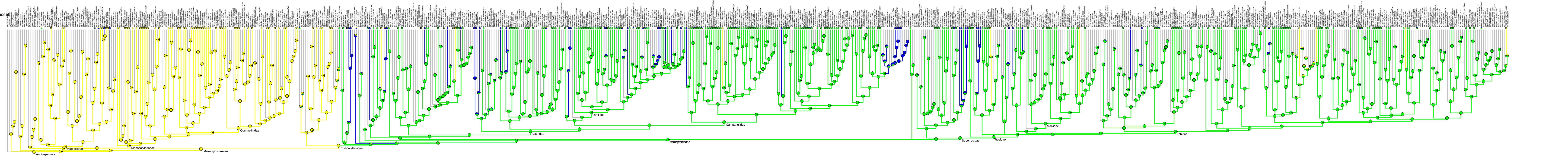

MP ancestral state reconstruction using ancestral.pars
(R:phangorn)
305_A. Filament (binary) (D2d), 55 steps

- laminar (wide)
- typical (narrow)

Node	MP state(s)
Angiospermae	laminar (wide) / typical (narrow)
Mesangiospermae	laminar (wide) / typical (narrow)
Magnoliidae	laminar (wide)
Monocotyledonae	typical (narrow)
Eudicotyledonae	typical (narrow)
Commelinidae	laminar (wide) / typical (narrow)
Pentapetalae	typical (narrow)
Superasteridae	typical (narrow)
Asteridae	typical (narrow)
Lamiidae	typical (narrow)
Campanulidae	typical (narrow)
Superrosidae	typical (narrow)
Rosidae	typical (narrow)
Malvidae	typical (narrow)
Fabidae	typical (narrow)

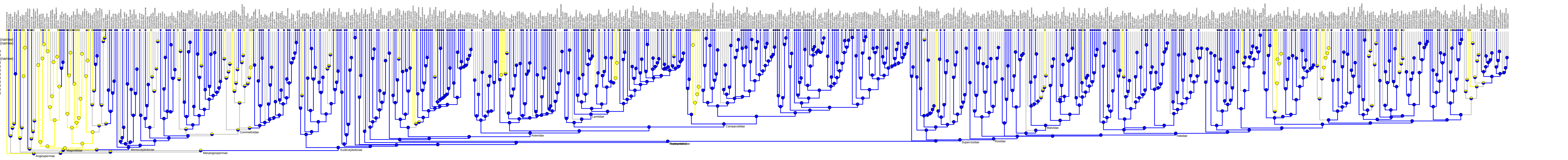

ML ancestral state reconstruction using rayDISC (R:corHMM)
305_A. Filament (binary) (D2d), ARDeq model

- laminar (wide)
- typical (narrow)

Node	ML state	Prob
Angiospermae	laminar (wide)	1
Mesangiospermae	laminar (wide)	0.9953
Magnoliidae	laminar (wide)	0.9978
Monocotyledonae	laminar (wide)	0.851
Eudicotyledonae	typical (narrow)	0.5337
Commelinidae	laminar (wide)	0.6355
Pentapetalae	typical (narrow)	0.9981
Superasteridae	typical (narrow)	0.9981
Asteridae	typical (narrow)	0.9995
Lamiidae	typical (narrow)	0.9971
Campanulidae	typical (narrow)	0.9945
Superrosidae	typical (narrow)	0.9991
Rosidae	typical (narrow)	0.9993
Malvidae	typical (narrow)	0.997
Fabidae	typical (narrow)	1

Model	LogL	Npar	AIC	AICc	DeltaAICc	w	q01	q10
ARD	−173.1	2	350.21	350.22	1.32	0.34	0.0092	0.0022
ARDeq**	−172.45	2	348.89	348.91	0	0.66	0.0093	0.0021
ER	−178.67	1	359.33	359.34	10.43	0	0.0024	0.0024
UNI01	−184.89	1	371.78	371.78	22.88	0	0.0147	
UNI10	−187.98	1	377.95	377.96	29.05	0		0.0023

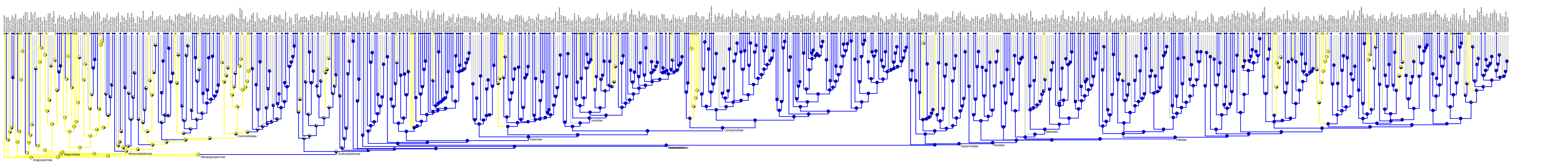

MP ancestral state reconstruction using ancestral.pars
(R:phangorn)
311_A. Anther orientation (D2d), 110 steps

- introrse
- latrorse
- extrorse

Node	MP state(s)
Angiospermae	introrse
Mesangiospermae	introrse / extrorse
Magnoliidae	extrorse
Monocotyledonae	introrse
Eudicotyledonae	introrse / extrorse
Commelinidae	introrse
Pentapetalae	introrse
Superasteridae	introrse
Asteridae	introrse
Lamiidae	introrse
Campanulidae	introrse
Superrosidae	introrse
Rosidae	introrse
Malvidae	introrse
Fabidae	introrse

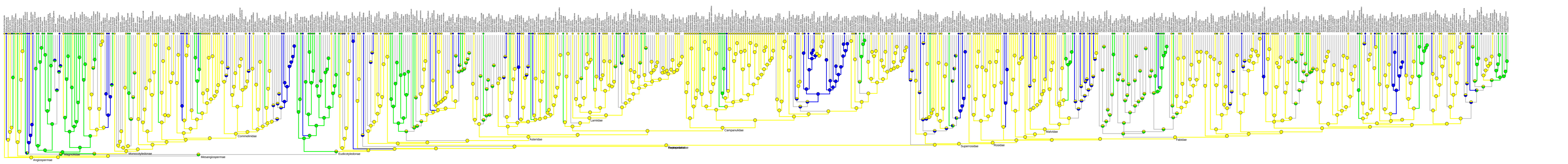

ML ancestral state reconstruction using rayDISC (R:corHMM)
311_A. Anther orientation (D2d), SYMeq model

- introrse
- latrorse
- extrorse

Node	ML state	Prob
Angiospermae	introrse	1
Mesangiospermae	introrse	0.9985
Magnoliidae	extrorse	0.7251
Monocotyledonae	introrse	0.9939
Eudicotyledonae	introrse	0.9978
Commelinidae	introrse	0.9946
Pentapetalae	introrse	1
Superasteridae	introrse	1
Asteridae	introrse	0.9997
Lamiidae	introrse	0.9997
Campanulidae	introrse	1
Superrosidae	introrse	1
Rosidae	introrse	1
Malvidae	introrse	0.9998
Fabidae	introrse	0.9998

Model	LogL	Npar	AIC	AICc	DeltaAICc	w	q01	···
ARD	−345.51	6	703.02	703.13	2.66	0.09	0.0021	···
ARDeq	−344.7	6	701.39	701.5	1.03	0.2	0.0038	···
ER	−349.52	1	701.05	701.05	0.59	0.25	0.0028	···
SYM	−348.28	3	702.56	702.59	2.12	0.12	0.0031	···
SYMeq*	−347.22	3	700.44	700.47	0	0.34	0.0031	···

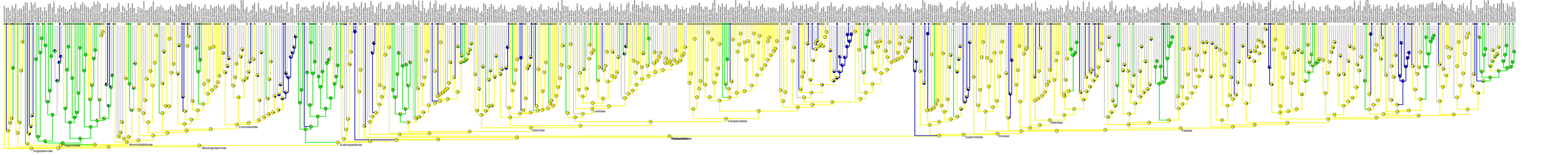

MP ancestral state reconstruction using ancestral.pars
(R:phangorn)
312_A. Anther attachment (binary) (D2d), 96 steps

- basifixed (yellow)
- dorsifixed (blue)

Node	MP state(s)
Angiospermae	basifixed
Mesangiospermae	basifixed
Magnoliidae	basifixed
Monocotyledonae	basifixed
Eudicotyledonae	basifixed
Commelinidae	basifixed
Pentapetalae	basifixed
Superasteridae	basifixed
Asteridae	basifixed
Lamiidae	basifixed
Campanulidae	basifixed / dorsifixed
Superrosidae	basifixed
Rosidae	dorsifixed
Malvidae	dorsifixed
Fabidae	dorsifixed

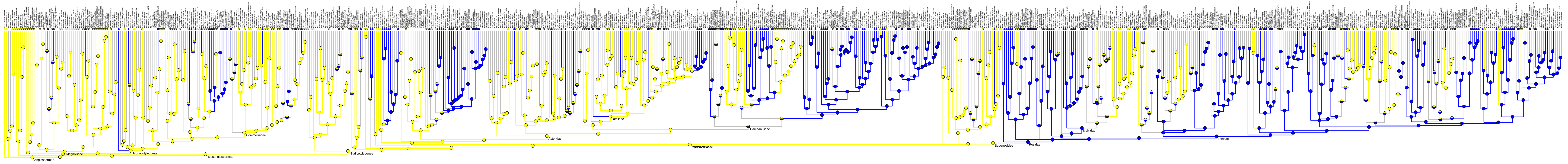

ML ancestral state reconstruction using rayDISC (R:corHMM)
312_A. Anther attachment (binary) (D2d), ARDeq model

- basifixed
- dorsifixed

Node	ML state	Prob
Angiospermae	basifixed	1
Mesangiospermae	basifixed	0.9994
Magnoliidae	basifixed	0.9993
Monocotyledonae	basifixed	0.9964
Eudicotyledonae	basifixed	0.8849
Commelinidae	basifixed	0.9857
Pentapetalae	dorsifixed	0.7638
Superasteridae	dorsifixed	0.7638
Asteridae	dorsifixed	0.7705
Lamiidae	basifixed	0.5045
Campanulidae	dorsifixed	0.9267
Superrosidae	dorsifixed	0.7834
Rosidae	dorsifixed	0.8723
Malvidae	dorsifixed	0.9743
Fabidae	dorsifixed	0.9994

Model	LogL	Npar	AIC	AICc	DeltaAICc	w	q01	q10
ARD	−265.7	2	535.4	535.41	1.34	0.27	0.0045	0.0073
ARDeq**	−265.03	2	534.06	534.08	0	0.53	0.0045	0.0073
ER	−266.99	1	535.98	535.99	1.91	0.2	0.0055	0.0055
UNI01	−292.69	1	587.37	587.38	53.3	0	0.0061	
UNI10	−277.87	1	557.73	557.74	23.66	0		0.008

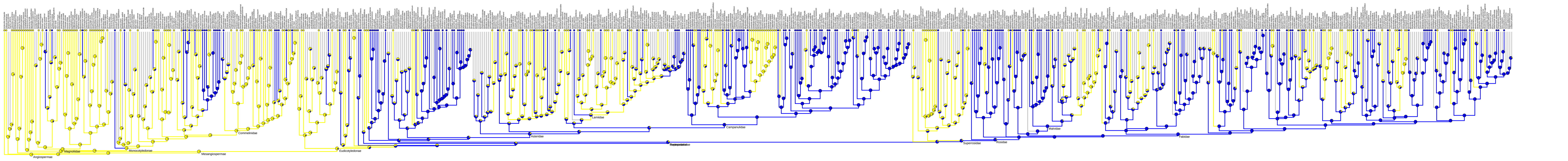

MP ancestral state reconstruction using ancestral.pars
(R:phangorn)
313_A. Anther dehiscence (3−state) (D2d), 10 steps

- longitudinal slit
- H−valvate
- flap−valvate

Node	MP state(s)
Angiospermae	longitudinal slit
Mesangiospermae	longitudinal slit
Magnoliidae	longitudinal slit
Monocotyledonae	longitudinal slit
Eudicotyledonae	longitudinal slit
Commelinidae	longitudinal slit
Pentapetalae	longitudinal slit
Superasteridae	longitudinal slit
Asteridae	longitudinal slit
Lamiidae	longitudinal slit
Campanulidae	longitudinal slit
Superrosidae	longitudinal slit
Rosidae	longitudinal slit
Malvidae	longitudinal slit
Fabidae	longitudinal slit

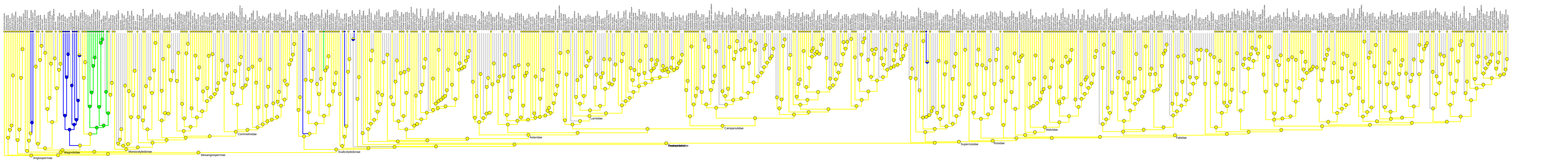

ML ancestral state reconstruction using rayDISC (R:corHMM)
313_A. Anther dehiscence (3−state) (D2d), ARDeq model

- longitudinal slit
- H−valvate
- flap−valvate

Node	ML state	Prob
Angiospermae	longitudinal slit	1
Mesangiospermae	longitudinal slit	1
Magnoliidae	longitudinal slit	0.9994
Monocotyledonae	longitudinal slit	1
Eudicotyledonae	longitudinal slit	1
Commelinidae	longitudinal slit	1
Pentapetalae	longitudinal slit	1
Superasteridae	longitudinal slit	1
Asteridae	longitudinal slit	1
Lamiidae	longitudinal slit	1
Campanulidae	longitudinal slit	1
Superrosidae	longitudinal slit	1
Rosidae	longitudinal slit	1
Malvidae	longitudinal slit	1
Fabidae	longitudinal slit	1

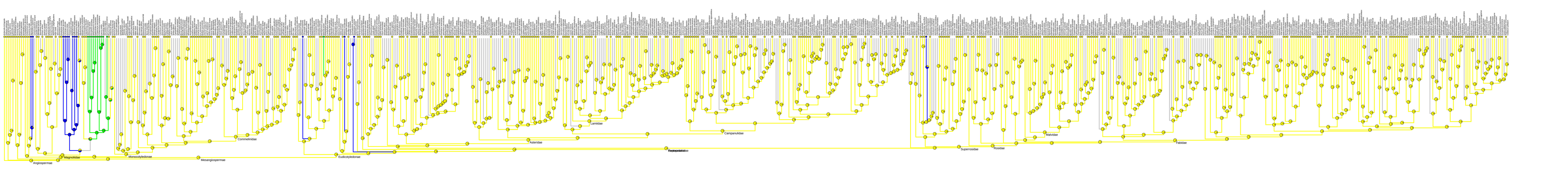

Model	LogL	Npar	AIC	AICc	DeltaAICc	w	q01	···
ARD	−56.78	6	125.55	125.66	2.19	0.13	2e−04	···
ARDeq*	−55.68	6	123.36	123.47	0	0.38	2e−04	···
ER	−61.41	1	124.81	124.82	1.35	0.2	2e−04	···
SYM	−60.38	3	126.77	126.8	3.33	0.07	2e−04	···
SYMeq	−59.29	3	124.57	124.6	1.14	0.22	2e−04	···

MP ancestral state reconstruction using ancestral.pars
(R:phangorn)
401_B. Number of structural carpels (5−state) (D2c), 187 steps

- one (1)
- two (2)
- three (3)
- four or five (4−5)
- more than five (>5)

Node	MP state(s)
Angiospermae	more than five (>5)
Mesangiospermae	more than five (>5)
Magnoliidae	more than five (>5)
Monocotyledonae	three (3)
Eudicotyledonae	more than five (>5)
Commelinidae	three (3)
Pentapetalae	three (3) / four or five (4−5)
Superasteridae	three (3) / four or five (4−5)
Asteridae	two (2) / four or five (4−5)
Lamiidae	two (2)
Campanulidae	two (2)
Superrosidae	three (3) / four or five (4−5)
Rosidae	three (3) / four or five (4−5)
Malvidae	four or five (4−5)
Fabidae	four or five (4−5)

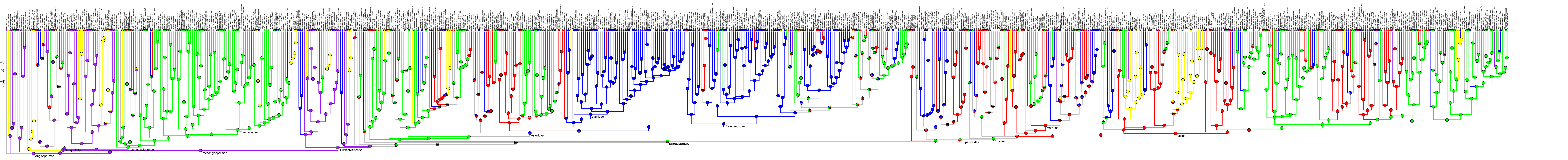

ML ancestral state reconstruction using rayDISC (R:corHMM)
401_B. Number of structural carpels (5−state) (D2c), ARDeq model

- one (1)
- two (2)
- three (3)
- four or five (4−5)
- more than five (>5)

Node	ML state	Prob
Angiospermae	more than five (>5)	1
Mesangiospermae	more than five (>5)	1
Magnoliidae	more than five (>5)	1
Monocotyledonae	more than five (>5)	0.8204
Eudicotyledonae	more than five (>5)	0.9956
Commelinidae	three (3)	0.9999
Pentapetalae	four or five (4−5)	0.9999
Superasteridae	four or five (4−5)	0.9999
Asteridae	four or five (4−5)	0.9953
Lamiidae	two (2)	0.9968
Campanulidae	two (2)	0.9551
Superrosidae	four or five (4−5)	0.9999
Rosidae	four or five (4−5)	0.9998
Malvidae	four or five (4−5)	0.9991
Fabidae	four or five (4−5)	1

Model	LogL	Npar	AIC	AICc	DeltaAICc	w	q01	···
ARD	−665.28	20	1370.55	1371.64	3.22	0.17	0.0017	···
ARDeq**	−663.67	20	1367.33	1368.42	0	0.83	0.0017	···
ER	−720.04	1	1442.09	1442.09	73.67	0	0.0016	···
SYM	−686.43	10	1392.85	1393.13	24.71	0	8e−04	···
SYMeq	−684.89	10	1389.77	1390.05	21.63	0	8e−04	···
ORD	−760.36	8	1536.72	1536.9	168.48	0	0.001	···
ORDeq	−758.84	8	1533.69	1533.87	165.45	0	0.001	···
ORDSYM	−764.3	4	1536.59	1536.64	168.22	0	0.0042	···
ORDSYMeq	−762.85	4	1533.7	1533.75	165.33	0	0.0042	···
ORDER	−769.83	1	1541.66	1541.67	173.25	0	0.0058	···

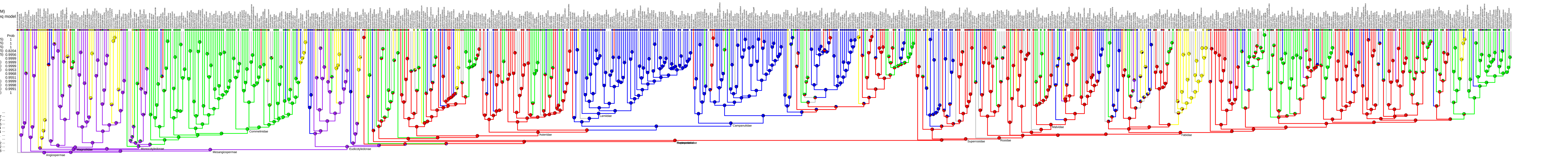

MP ancestral state reconstruction using ancestral.pars
(R:phangorn)
400_A. Gynoecium phyllotaxy (D2d), 10 steps

- whorled
- spiral

Node	MP state(s)
Angiospermae	whorled / spiral
Mesangiospermae	whorled
Magnoliidae	whorled
Monocotyledonae	whorled
Eudicotyledonae	whorled
Commelinidae	whorled
Pentapetalae	whorled
Superasteridae	whorled
Asteridae	whorled
Lamiidae	whorled
Campanulidae	whorled
Superrosidae	whorled
Rosidae	whorled
Malvidae	whorled
Fabidae	whorled

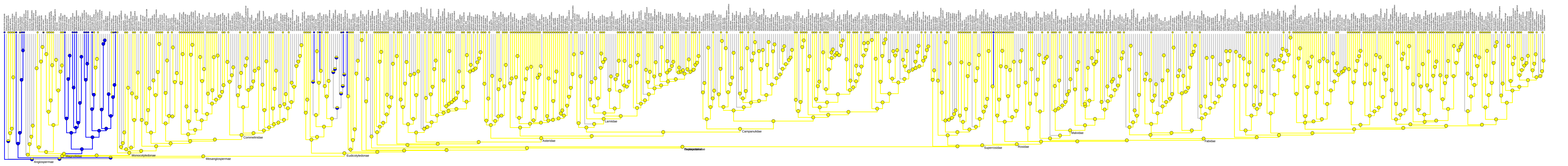

ML ancestral state reconstruction using rayDISC (R:corHMM)
400_A. Gynoecium phyllotaxy (D2d), ARDeq model

- whorled
- spiral

Node	ML state	Prob
Angiospermae	spiral	1
Mesangiospermae	spiral	1
Magnoliidae	spiral	0.9997
Monocotyledonae	whorled	0.5317
Eudicotyledonae	spiral	0.9997
Commelinidae	whorled	0.9997
Pentapetalae	whorled	0.9746
Superasteridae	whorled	0.9746
Asteridae	whorled	1
Lamiidae	whorled	1
Campanulidae	whorled	1
Superrosidae	whorled	0.9775
Rosidae	whorled	0.9777
Malvidae	whorled	1
Fabidae	whorled	1

Model	LogL	Npar	AIC	AICc	DeltaAICc	w	q01	q10
ARD	−39.73	2	83.46	83.48	1.39	0.31	1e−04	0.0083
ARDeq**	−39.04	2	82.07	82.09	0	0.62	1e−04	0.0083
ER	−51.33	1	104.66	104.66	22.57	0	4e−04	4e−04
UNI01	−52.95	1	107.89	107.9	25.81	0	4e−04	
UNI10	−42.27	1	86.55	86.55	4.46	0.07		0.0098

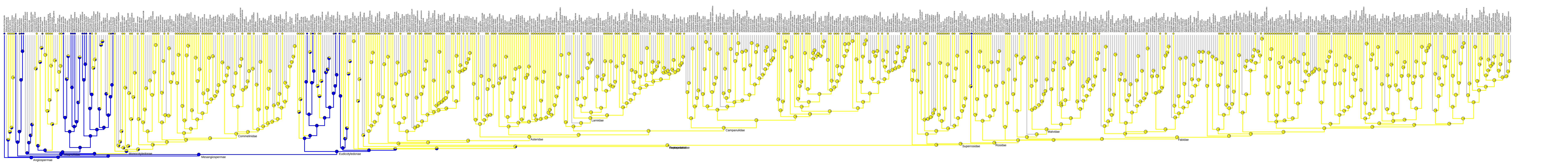

MP ancestral state reconstruction using ancestral.pars
(R:phangorn)
403_A. Fusion of ovaries (binary) (D2c), 24 steps

- free (<5%)
- fused (>5%)

Node	MP state(s)
Angiospermae	free (<5%) / fused (>5%)
Mesangiospermae	fused (>5%)
Magnoliidae	fused (>5%)
Monocotyledonae	fused (>5%)
Eudicotyledonae	fused (>5%)
Commelinidae	fused (>5%)
Pentapetalae	fused (>5%)
Superasteridae	fused (>5%)
Asteridae	fused (>5%)
Lamiidae	fused (>5%)
Campanulidae	fused (>5%)
Superrosidae	fused (>5%)
Rosidae	fused (>5%)
Malvidae	fused (>5%)
Fabidae	fused (>5%)

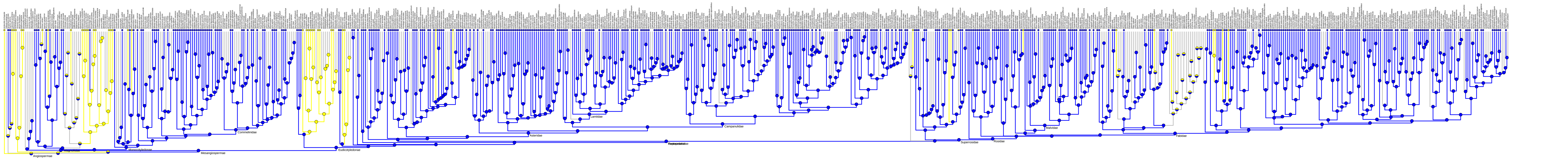

ML ancestral state reconstruction using rayDISC (R:corHMM)
403_A. Fusion of ovaries (binary) (D2c), ARD model

- free (<5%)
- fused (>5%)

Node	ML state	Prob
Angiospermae	free (<5%)	1
Mesangiospermae	free (<5%)	1
Magnoliidae	free (<5%)	0.9999
Monocotyledonae	free (<5%)	0.9344
Eudicotyledonae	free (<5%)	0.9992
Commelinidae	fused (>5%)	0.9999
Pentapetalae	fused (>5%)	0.9944
Superasteridae	fused (>5%)	0.9944
Asteridae	fused (>5%)	1
Lamiidae	fused (>5%)	1
Campanulidae	fused (>5%)	1
Superrosidae	fused (>5%)	0.9992
Rosidae	fused (>5%)	0.9996
Malvidae	fused (>5%)	1
Fabidae	fused (>5%)	0.9998

Model	LogL	Npar	AIC	AICc	DeltaAICc	w	q01	q10
ARD**	−95.19	2	194.39	194.4	0	0.93	0.0053	5e−04
ARDeq	−102.31	2	208.62	208.64	14.24	0	7e−04	8e−04
ER	−102.81	1	207.63	207.63	13.23	0	8e−04	8e−04
UNI01	−98.85	1	199.69	199.7	5.29	0.07	0.0113	
UNI10	−107.27	1	216.53	216.54	22.13	0		9e−04

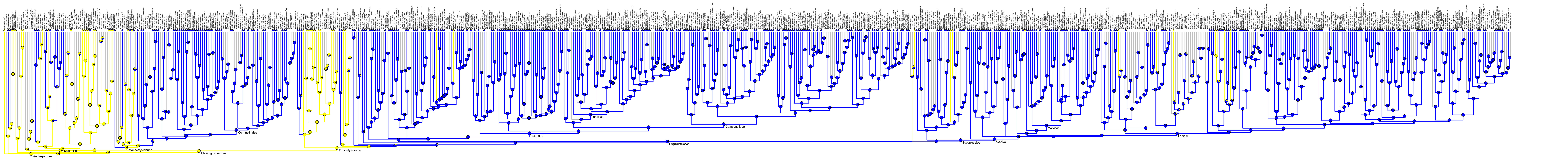

MP ancestral state reconstruction using ancestral.pars
(R:phangorn)
411_A. Number of ovules per functional carpel (3−state) (D2c), 119 steps

- one (1)
- two (2)
- three or more (>2)

Node	MP state(s)
Angiospermae	one (1)
Mesangiospermae	one (1) / three or more (>2)
Magnoliidae	one (1) / three or more (>2)
Monocotyledonae	one (1) / three or more (>2)
Eudicotyledonae	three or more (>2)
Commelinidae	one (1)
Pentapetalae	three or more (>2)
Superasteridae	three or more (>2)
Asteridae	one (1)
Lamiidae	one (1) / two (2) / three or more (>2)
Campanulidae	one (1)
Superrosidae	two (2) / three or more (>2)
Rosidae	two (2)
Malvidae	two (2) / three or more (>2)
Fabidae	two (2)

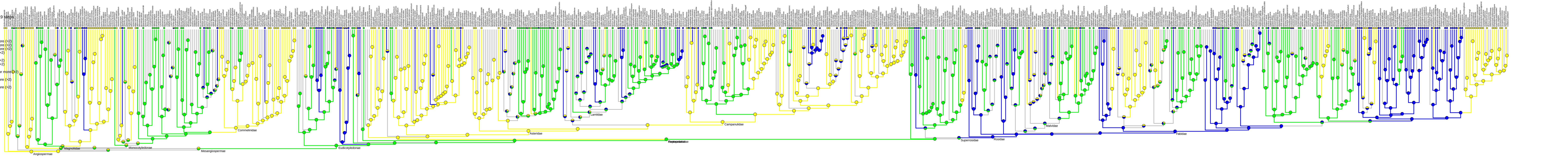

ML ancestral state reconstruction using rayDISC (R:corHMM)

411_A. Number of ovules per functional carpel (3−state) (D2c), ARDeq model

- one (1)
- two (2)
- three or more (>2)

Node	ML state	Prob
Angiospermae	three or more (>2)	1
Mesangiospermae	three or more (>2)	1
Magnoliidae	three or more (>2)	1
Monocotyledonae	three or more (>2)	1
Eudicotyledonae	three or more (>2)	1
Commelinidae	three or more (>2)	0.8553
Pentapetalae	three or more (>2)	1
Superasteridae	three or more (>2)	1
Asteridae	three or more (>2)	0.9948
Lamiidae	three or more (>2)	1
Campanulidae	three or more (>2)	0.9983
Superrosidae	three or more (>2)	1
Rosidae	three or more (>2)	1
Malvidae	three or more (>2)	0.9997
Fabidae	three or more (>2)	1

Model	LogL	Npar	AIC	AICc	DeltaAICc	w	q01	···
ARD	−383.3	6	778.6	778.7	2.18	0.25	0.0013	···
ARDeq**	−382.21	6	776.41	776.52	0	0.75	0.0013	···
ER	−398.81	1	799.62	799.63	23.11	0	0.0028	···
SYM	−398.31	3	802.61	802.65	26.13	0	0.0023	···
SYMeq	−397.45	3	800.89	800.92	24.4	0	0.0024	···
ORD	−399.24	4	806.47	806.53	30.01	0	0.0019	···
ORDeq	−398.16	4	804.31	804.36	27.84	0	0.0019	···
ORDSYM	−412.3	2	828.6	828.61	52.09	0	0.0054	···
ORDSYMeq	−411.4	2	826.79	826.81	50.29	0	0.0054	···
ORDER	−413.37	1	828.74	828.74	52.22	0	0.0063	···

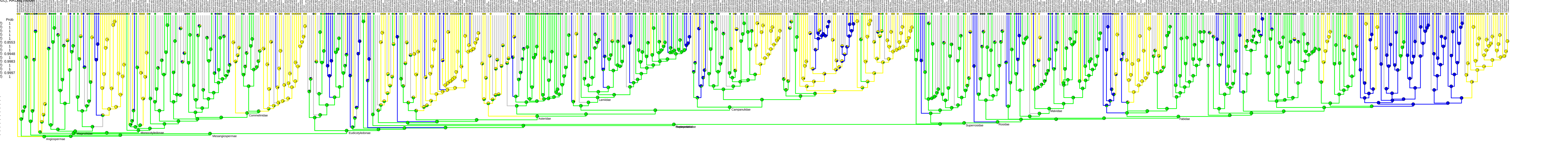

Supplement: Supplementary Data 18 [file ncomms16047-s19.pdf]
