# Supplementary material for: The ancestral flower of angiosperms and its early diversification
Source: Nat Commun. 2017 Aug 1;8:16047. doi: 10.1038/ncomms16047 (PMC5543309; doi:10.1038/ncomms16047)

MP ancestral state reconstruction using ancestral.pars
(R:phangorn)
100_A. Functional sex of flowers (D2d), 96 steps

- bisexual
- unisexual

Node	MP state(s)
Angiospermae	bisexual / unisexual
Mesangiospermae	bisexual
Magnoliidae	bisexual
Monocotyledonae	bisexual
Eudicotyledonae	bisexual
Commelinidae	bisexual
Pentapetalae	bisexual
Superasteridae	bisexual
Asteridae	bisexual
Lamiidae	bisexual
Campanulidae	bisexual
Superrosidae	bisexual
Rosidae	bisexual
Malvidae	bisexual
Fabidae	bisexual

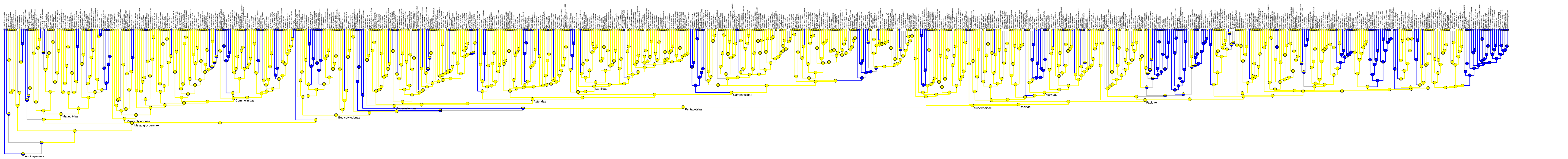

ML ancestral state reconstruction using rayDISC (R:corHMM)
100_A. Functional sex of flowers (D2d), ARDeq model

- bisexual
- unisexual

Node	ML state	Prob
Angiospermae	bisexual	1
Mesangiospermae	bisexual	1
Magnoliidae	bisexual	1
Monocotyledonae	bisexual	1
Eudicotyledonae	bisexual	1
Commelinidae	bisexual	1
Pentapetalae	bisexual	1
Superasteridae	bisexual	1
Asteridae	bisexual	1
Lamiidae	bisexual	1
Campanulidae	bisexual	0.9996
Superrosidae	bisexual	1
Rosidae	bisexual	1
Malvidae	bisexual	1
Fabidae	bisexual	1

Model	LogL	Npar	AIC	AICc	DeltaAICc	w	q01	q10
ARD	−312.88	2	629.76	629.78	1.27	0.22	0.0028	6e−04
ARDeq*	−312.25	2	628.49	628.51	0	0.42	0.0028	6e−04
ER	−315.45	1	632.9	632.91	4.4	0.05	0.0027	0.0027
UNI01	−313.53	1	629.06	629.07	0.56	0.32	0.0028	
UNI10	−360.51	1	723.01	723.02	94.51	0		0.0112

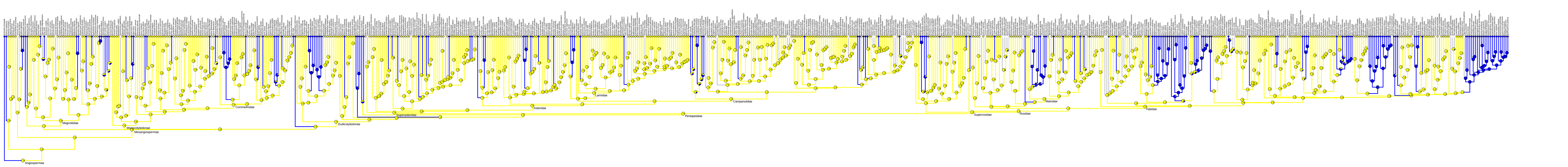

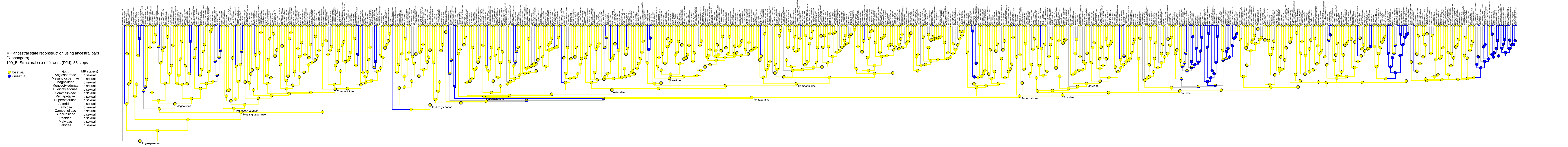
MP ancestral state reconstruction using ancestral.pars
(R:phangorn)
100_B. Structural sex of flowers (D2d), 55 steps
bisexual
unisexual
Node
MP state(s)
Angiospermae
bisexual
Mesangiospermae
bisexual
Magnoliidae
bisexual
Monocotyledonae
bisexual
Eudicotyledonae
bisexual
Commelinidae
bisexual
Pentapetalae
bisexual
Superasteridae
bisexual
Asteridae
bisexual
Lamiidae
bisexual
Campanulidae
bisexual
Superrosidae
bisexual
Rosidae
bisexual
Malvidae
bisexual
Fabidae
bisexual
Angiospermae
Magnoliidae
Monocotyledonae
Mesangiospermae
Commelinidae
Eudicotyledonae
Pentapetalae
Asteridae
Lamiidae
Campanulidae
Superrosidae
Rosidae
Malvidae
Fabidae

ML ancestral state reconstruction using rayDISC (R:corHMM)
100_B. Structural sex of flowers (D2d), ARDeq model

- bisexual
- unisexual

Node	ML state	Prob
Angiospermae	bisexual	0.9954
Mesangiospermae	bisexual	0.9997
Magnoliidae	bisexual	0.9998
Monocotyledonae	bisexual	0.9998
Eudicotyledonae	bisexual	0.9999
Commelinidae	bisexual	0.9999
Pentapetalae	bisexual	1
Superasteridae	bisexual	1
Asteridae	bisexual	1
Lamiidae	bisexual	0.9999
Campanulidae	bisexual	1
Superrosidae	bisexual	1
Rosidae	bisexual	1
Malvidae	bisexual	1
Fabidae	bisexual	0.9996

Model	LogL	Npar	AIC	AICc	DeltaAICc	w	q01	q10
ARD	−207.08	2	418.16	418.18	0.81	0.37	[illegible]	0.0039
ARDeq**	−206.68	2	417.35	417.37	0	0.55	0.0014	0.0037
ER	−209.56	1	421.13	421.13	3.76	0.08	0.0015	0.0015
UNI01	−224.83	1	451.65	451.66	34.29	0	0.0016	
UNI10	−235.26	1	472.51	472.52	55.15	0		0.0155

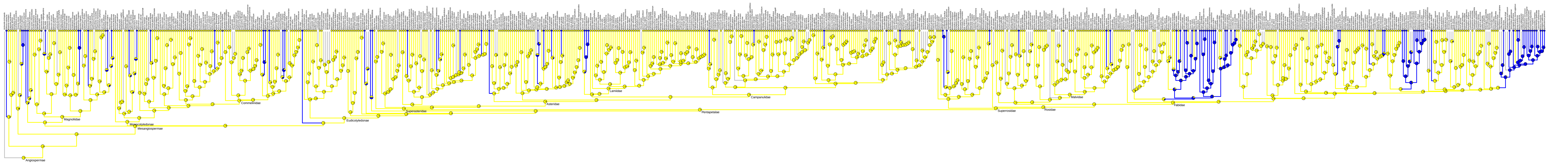

MP ancestral state reconstruction using ancestral.pars
(R:phangorn)
102_B. Ovary position (binary) (D2d), 81 steps

- superior
- inferior

Node	MP state(s)
Angiospermae	superior
Mesangiospermae	superior
Magnoliidae	superior
Monocotyledonae	superior
Eudicotyledonae	superior
Commelinidae	superior
Pentapetalae	superior
Superasteridae	superior
Asteridae	superior
Lamiidae	superior
Campanulidae	superior
Superrosidae	superior / inferior
Rosidae	superior / inferior
Malvidae	superior
Fabidae	superior

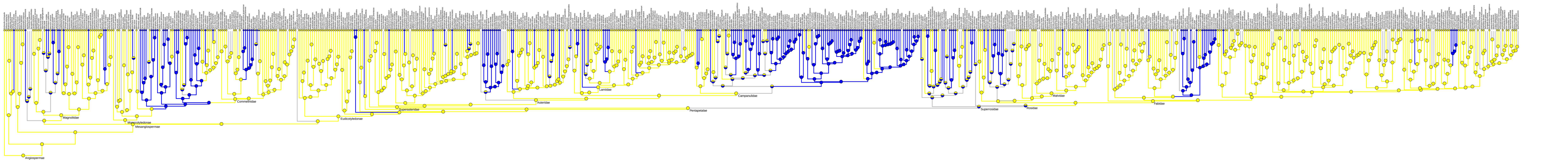

ML ancestral state reconstruction using rayDISC (R:corHMM)
102_B. Ovary position (binary) (D2d), ARDeq model

- superior
- inferior

Node	ML state	Prob
Angiospermae	superior	0.9998
Mesangiospermae	superior	0.9997
Magnoliidae	superior	0.9966
Monocotyledonae	superior	0.9937
Eudicotyledonae	superior	0.9999
Commelinidae	superior	0.7959
Pentapetalae	superior	0.9996
Superasteridae	superior	0.9998
Asteridae	superior	0.9521
Lamiidae	superior	0.9575
Campanulidae	superior	0.781
Superrosidae	superior	0.9917
Rosidae	superior	0.9922
Malvidae	superior	1
Fabidae	superior	0.9997

Model	LogL	Npar	AIC	AICc	DeltaAICc	w	q01	q10
ARD	−271.03	2	546.07	546.08	1.16	0.29	0.0019	0.0036
ARDeq**	−270.45	2	544.9	544.92	0	0.52	0.0019	0.0035
ER	−272.51	1	547.03	547.03	2.12	0.18	0.0022	0.0022
UNI01	−280.77	1	563.55	563.55	18.64	0	0.0025	
UNI10	−298.66	1	599.33	599.33	54.41	0		0.0095

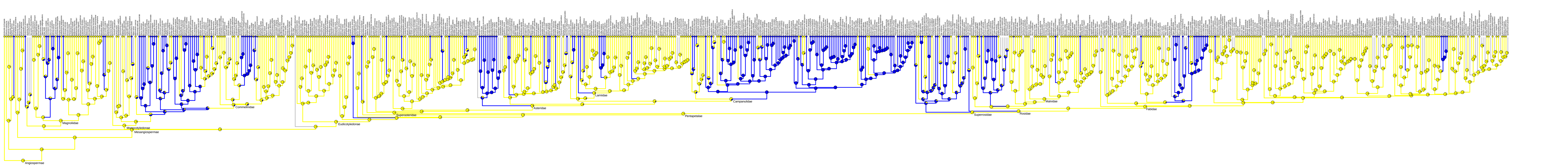

MP ancestral state reconstruction using ancestral.pars
(R:phangorn)
201_A. Perianth presence (D2c), 20 steps

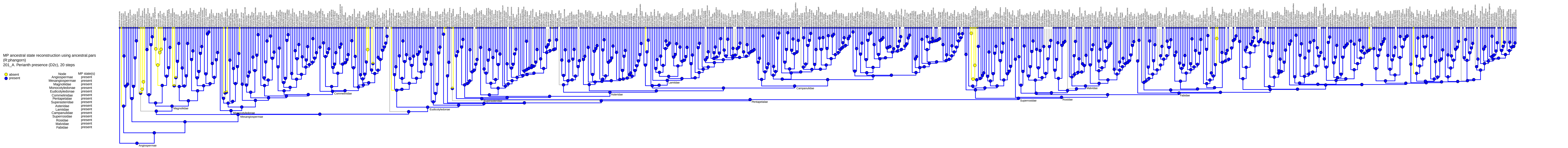

Node	MP state(s)
Angiospermae	present
Mesangiospermae	present
Magnoliidae	present
Monocotyledonae	present
Eudicotyledonae	present
Commelinidae	present
Pentapetalae	present
Superasteridae	present
Asteridae	present
Lamiidae	present
Campanulidae	present
Superrosidae	present
Rosidae	present
Malvidae	present
Fabidae	present

ML ancestral state reconstruction using rayDISC (R:corHMM)
201_A. Perianth presence (D2c), UNI10 model

absent
present

Node	ML state	Prob
Angiospermae	present	1
Mesangiospermae	present	1
Magnoliidae	present	1
Monocotyledonae	present	1
Eudicotyledonae	present	1
Commelinidae	present	1
Pentapetalae	present	1
Superasteridae	present	1
Asteridae	present	1
Lamiidae	present	1
Campanulidae	present	1
Superrosidae	present	1
Rosidae	present	1
Malvidae	present	1
Fabidae	present	1

Model	LogL	Npar	AIC	AICc	DeltaAICc	w	q01	q10
ARD	−95.9	2	195.8	195.82	2.01	0.13	0	5e−04
ARDeq	−95.21	2	194.41	194.43	0.62	0.25	0	5e−04
ER	−96.13	1	194.26	194.26	0.45	0.28	5e−04	5e−04
UNI01	−113.81	1	229.62	229.63	35.82	0	0.0201	
UNI10*	−95.9	1	193.8	193.81	0	0.35		5e−04

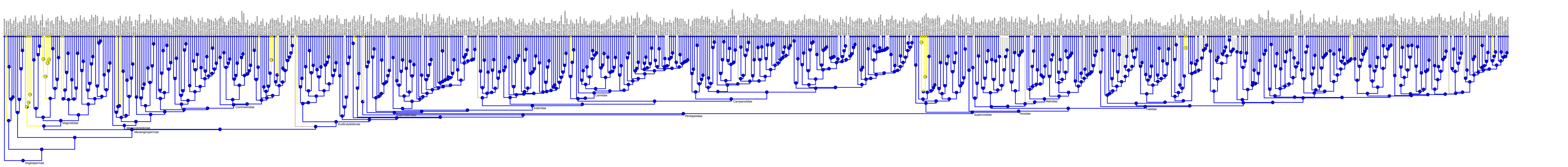

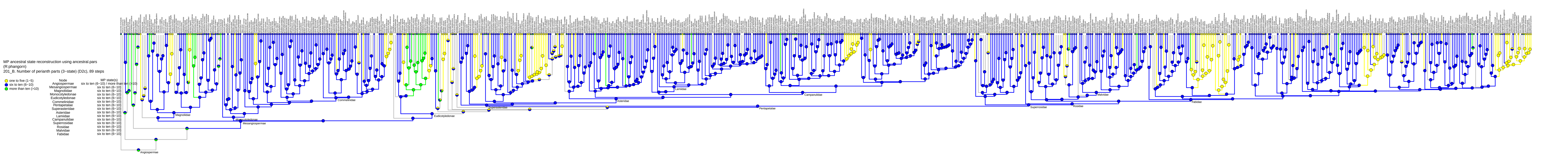
MP ancestral state reconstruction using ancestral.pars
(R:phangorn)
201_B. Number of perianth parts (3−state) (D2c), 89 steps
one to five (1−5)
six to ten (6−10)
more than ten (>10)
Node
MP state(s)
Angiospermae
six to ten (6−10) / more than ten (>10)
Mesangiospermae
six to ten (6−10)
Magnoliidae
six to ten (6−10)
Monocotyledonae
six to ten (6−10)
Eudicotyledonae
six to ten (6−10)
Commelinidae
six to ten (6−10)
Pentapetalae
six to ten (6−10)
Superasteridae
six to ten (6−10)
Asteridae
six to ten (6−10)
Lamiidae
six to ten (6−10)
Campanulidae
six to ten (6−10)
Superrosidae
six to ten (6−10)
Rosidae
six to ten (6−10)
Malvidae
six to ten (6−10)
Fabidae
six to ten (6−10)
Angiospermae
Magnoliidae
Monocotyledonae
Mesangiospermae
Commelinidae
Eudicotyledonae
Superasteridae
Asteridae
Lamiidae
Campanulidae
Pentapetalae
Superrosidae
Rosidae
Malvidae
Fabidae

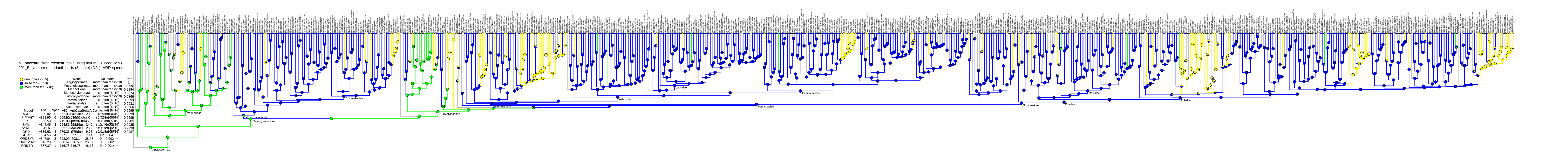
ML ancestral state reconstruction using rayDISC (R:corHMM)
201_B. Number of perianth parts (3−state) (D2c), ARDeq model
one to five (1−5)
six to ten (6−10)
more than ten (>10)
Node ML state Prob
Angiospermae more than ten (>10) 1
Mesangiospermae more than ten (>10) 0.999
Magnoliidae more than ten (>10) 0.9994
Monocotyledonae six to ten (6−10) 0.5374
Eudicotyledonae more than ten (>10) 0.9866
Commelinidae six to ten (6−10) 0.9999
Pentapetalae six to ten (6−10) 0.9931
Superasteridae six to ten (6−10) 0.9966
Asteridae six to ten (6−10) 0.9999
Lamiidae six to ten (6−10) 0.9998
Campanulidae six to ten (6−10) 0.9999
Superrosidae six to ten (6−10) 0.9981
Rosidae six to ten (6−10) 0.9985
Malvidae six to ten (6−10) 0.9998
Fabidae six to ten (6−10) 0.9987
Model LogL Npar AIC AICc DeltaAICc w q
ARD −330.02 6 672.03 672.14 2.12 0.25 0.0046 ···
ARDeq** −328.96 6 669.91 670.02 0 0.72 0.0046 ···
ER −356.53 1 715.06 715.07 45.05 0 0.0013 ···
SYM −344.45 3 694.89 694.92 24.9 0 0.002 ···
SYMeq −343.6 3 693.19 693.22 23.2 0 0.002 ···
ORD −335.62 4 679.25 679.3 9.28 0.01 0.0047 ···
ORDeq −334.55 4 677.11 677.16 7.14 0.02 0.0047 ···
ORDSYM −347.04 2 698.09 698.1 28.08 0 0.002 ···
ORDSYMeq −346.29 2 696.57 696.59 26.57 0 0.002 ···
ORDER −357.37 1 716.75 716.75 46.73 0 0.0014 ···
Angiospermae
Mesangiospermae
Magnoliidae
Monocotyledonae
Commelinidae
Eudicotyledonae
Pentapetalae
Asteridae
Lamiidae
Campanulidae
Superrosidae
Rosidae
Malvidae
Fabidae

MP ancestral state reconstruction using ancestral.pars
(R:phangorn)
201_C. Number of perianth parts (binary) (D2c), 71 steps

- one to six (1−6)
- more than six (>6)

Node	MP state(s)
Angiospermae	more than six (>6)
Mesangiospermae	one to six (1−6) / more than six (>6)
Magnoliidae	one to six (1−6) / more than six (>6)
Monocotyledonae	one to six (1−6)
Eudicotyledonae	one to six (1−6)
Commelinidae	one to six (1−6)
Pentapetalae	more than six (>6)
Superasteridae	more than six (>6)
Asteridae	more than six (>6)
Lamiidae	more than six (>6)
Campanulidae	more than six (>6)
Superrosidae	more than six (>6)
Rosidae	more than six (>6)
Malvidae	more than six (>6)
Fabidae	more than six (>6)

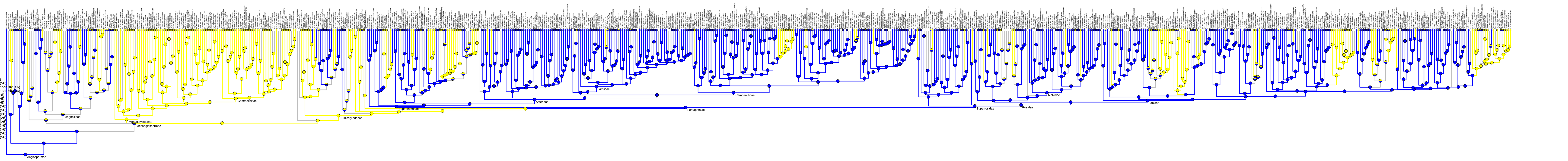

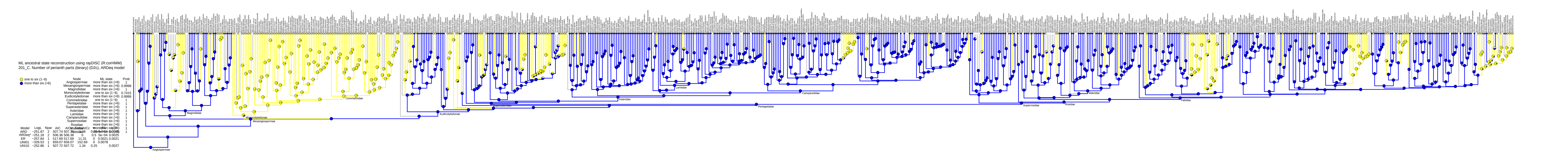
ML ancestral state reconstruction using rayDISC (R:corHMM)
201_C. Number of perianth parts (binary) (D2c), ARDeq model
one to six (1−6)
more than six (>6)
Node | ML state | Prob
Angiospermae | more than six (>6) | 1
Mesangiospermae | more than six (>6) | 0.9998
Magnoliidae | more than six (>6) | 1
Monocotyledonae | one to six (1−6) | 0.7415
Eudicotyledonae | more than six (>6) | 0.9999
Commelinidae | one to six (1−6) | 1
Pentapetalae | more than six (>6) | 1
Superasteridae | more than six (>6) | 1
Asteridae | more than six (>6) | 1
Lamiidae | more than six (>6) | 1
Campanulidae | more than six (>6) | 1
Superrosidae | more than six (>6) | 1
Rosidae | more than six (>6) | 1
Malvidae | more than six (>6) | 1
Fabidae | more than six (>6) | 1
Model | LogL | Npar | AIC | AICc | DeltaAICc | w | q01 | q10
ARD | −251.87 | 2 | 507.74 | 507.76 | 1.38 | 0.25 | [illegible] | [illegible]
ARDeq* | −251.18 | 2 | 506.36 | 506.38 | 0 | 0.5 | 5e−04 | 0.0025
ER | −257.84 | 1 | 517.69 | 517.69 | 11.31 | 0 | 0.0021 | 0.0021
UNI01 | −328.53 | 1 | 659.07 | 659.07 | 152.69 | 0 | 0.0078
UNI10 | −252.86 | 1 | 507.72 | 507.72 | 1.34 | 0.25 | | 0.0027
Angiospermae
Magnoliidae
Monocotyledonae
Mesangiospermae
Commelinidae
Eudicotyledonae
Pentapetalae
Lamiidae
Asteridae
Campanulidae
Superrosidae
Rosidae
Malvidae
Fabidae

MP ancestral state reconstruction using ancestral.pars
(R:phangorn)
230_A. Perianth phyllotaxy (binary) (D2d), 16 steps

- whorled
- spiral

Node	MP state(s)
Angiospermae	whorled / spiral
Mesangiospermae	whorled
Magnoliidae	whorled
Monocotyledonae	whorled
Eudicotyledonae	whorled
Commelinidae	whorled
Pentapetalae	whorled
Superasteridae	whorled
Asteridae	whorled
Lamiidae	whorled
Campanulidae	whorled
Superrosidae	whorled
Rosidae	whorled
Malvidae	whorled
Fabidae	whorled

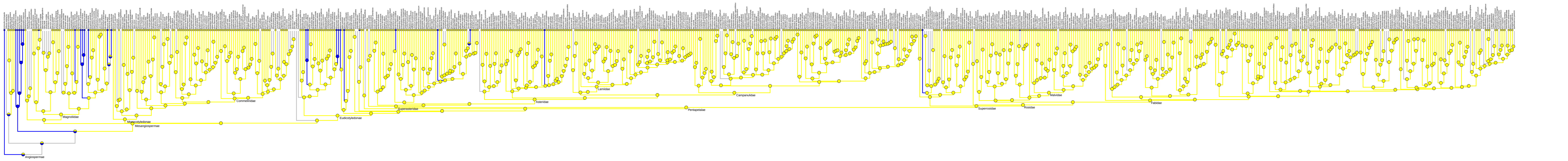

ML ancestral state reconstruction using rayDISC (R:corHMM)
230_A. Perianth phyllotaxy (binary) (D2d), ARDeq model

- whorled
- spiral

Node	ML state	Prob
Angiospermae	spiral	1
Mesangiospermae	spiral	0.9997
Magnoliidae	spiral	0.9975
Monocotyledonae	spiral	0.514
Eudicotyledonae	spiral	0.998
Commelinidae	whorled	0.9999
Pentapetalae	whorled	0.9876
Superasteridae	whorled	0.9919
Asteridae	whorled	0.9996
Lamiidae	whorled	1
Campanulidae	whorled	1
Superrosidae	whorled	0.9943
Rosidae	whorled	0.9963
Malvidae	whorled	1
Fabidae	whorled	1

Model	LogL	Npar	AIC	AICc	DeltaAICc	w	q01	q10
ARD	−70.62	2	145.24	145.26	1.38	0.33	2e−04	0.0094
ARDeq**	−69.93	2	143.86	143.88	0	0.66	2e−04	0.0094
ER	−76.48	1	154.96	154.97	11.09	0	4e−04	4e−04
UNI01	−76.87	1	155.74	155.75	11.87	0	4e−04	
UNI10	−79.49	1	160.97	160.98	17.1	0		0.0161

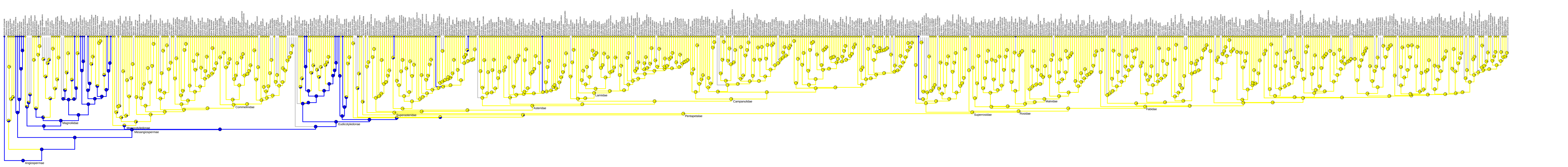

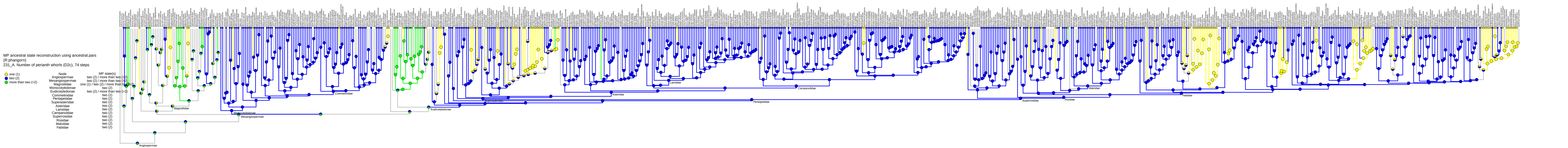
MP ancestral state reconstruction using ancestral.pars
(R:phangorn)
231_A. Number of perianth whorls (D2c), 74 steps
one (1)
two (2)
more than two (>2)
Node
MP state(s)
Angiospermae
two (2) / more than two (>2)
Mesangiospermae
two (2) / more than two (>2)
Magnoliidae
one (1) / two (2) / more than two (>2)
Monocotyledonae
two (2)
Eudicotyledonae
two (2) / more than two (>2)
Commelinidae
two (2)
Pentapetalae
two (2)
Superasteridae
two (2)
Asteridae
two (2)
Lamiidae
two (2)
Campanulidae
two (2)
Superrosidae
two (2)
Rosidae
two (2)
Malvidae
two (2)
Fabidae
two (2)
Angiospermae
Magnoliidae
Monocotyledonae
Mesangiospermae
Commelinidae
Eudicotyledonae
Asteridae
Lamiidae
Campanulidae
Pentapetalae
Superrosidae
Rosidae
Malvidae
Fabidae

ML ancestral state reconstruction using rayDISC (R:corHMM)
231_A. Number of perianth whorls (D2c), ARDeq model

- one (1)
- two (2)
- more than two (>2)

Node	ML state	Prob
Angiospermae	more than two (>2)	1
Mesangiospermae	more than two (>2)	0.9994
Magnoliidae	more than two (>2)	1
Monocotyledonae	two (2)	0.6671
Eudicotyledonae	more than two (>2)	0.984
Commelinidae	two (2)	1
Pentapetalae	two (2)	0.9996
Superasteridae	two (2)	0.9993
Asteridae	two (2)	0.9998
Lamiidae	two (2)	0.9999
Campanulidae	two (2)	1
Superrosidae	two (2)	0.9999
Rosidae	two (2)	0.9999
Malvidae	two (2)	0.9999
Fabidae	two (2)	0.9989

Model	LogL	Npar	AIC	AICc	DeltaAICc	wAICc	[illegible]	
ARD	−269.55	6	551.09	551.2	2.17	0.25	0.0047	···
ARDeq**	−268.46	6	548.92	549.03	0	0.75	0.0047	···
ER	−311.54	1	625.07	625.08	76.05	0	0.001	···
SYM	−292.58	3	591.16	591.19	42.17	0	0.0019	···
SYMeq	−292.09	3	590.18	590.21	41.18	0	0.0018	···
ORD	−277.79	4	563.58	563.63	14.6	0	0.0048	···
ORDeq	−276.7	4	561.39	561.44	12.42	0	0.0048	···
ORDSYM	−300.34	2	604.69	604.7	55.68	0	0.0018	···
ORDSYMeq	−299.54	2	603.07	603.09	54.06	0	0.0018	···
ORDER	−316.28	1	634.55	634.56	85.53	0	0.0011	···

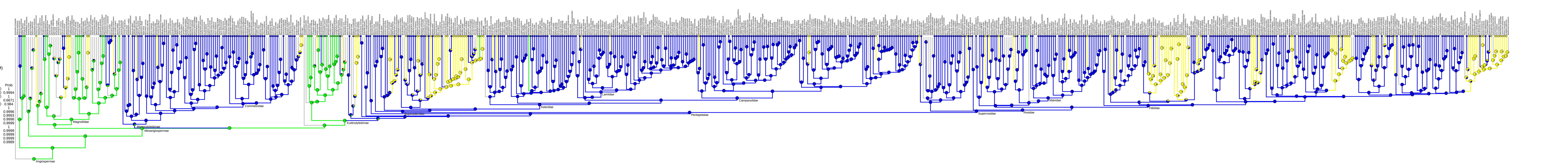

MP ancestral state reconstruction using ancestral.pars
(R:phangorn)
232_A. Perianth merism (4−state) (D2c), 84 steps

- dimerous
- trimerous
- tetramerous
- pentamerous

Node	MP state(s)
Angiospermae	trimerous
Mesangiospermae	trimerous
Magnoliidae	trimerous
Monocotyledonae	trimerous
Eudicotyledonae	dimerous / trimerous
Commelinidae	trimerous
Pentapetalae	pentamerous
Superasteridae	pentamerous
Asteridae	pentamerous
Lamiidae	pentamerous
Campanulidae	pentamerous
Superrosidae	pentamerous
Rosidae	pentamerous
Malvidae	pentamerous
Fabidae	pentamerous

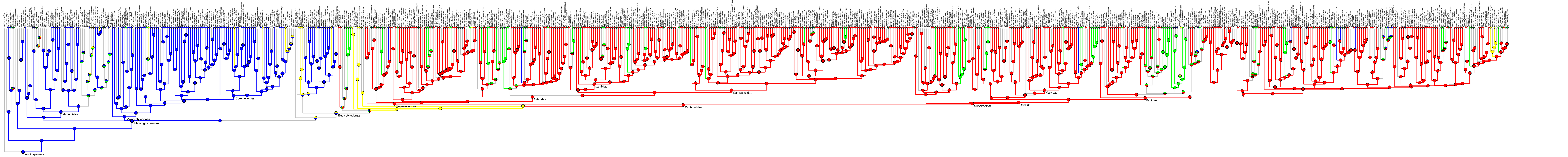

ML ancestral state reconstruction using rayDISC (R:corHMM)
232_A. Perianth merism (4−state) (D2c), SYMeq model

- dimerous (yellow)
- trimerous (blue)
- tetramerous (green)
- pentamerous (red)

Node	ML state	Prob
Angiospermae	trimerous	1
Mesangiospermae	trimerous	1
Magnoliidae	trimerous	1
Monocotyledonae	trimerous	1
Eudicotyledonae	trimerous	0.8914
Commelinidae	trimerous	1
Pentapetalae	pentamerous	0.9993
Superasteridae	pentamerous	0.9999
Asteridae	pentamerous	0.9999
Lamiidae	pentamerous	1
Campanulidae	pentamerous	1
Superrosidae	pentamerous	1
Rosidae	pentamerous	1
Malvidae	pentamerous	1
Fabidae	pentamerous	1

Model	LogL	Npar	AIC	AICc	DeltaAICc	w	q01	···
ARD	−322.95	12	669.89	670.29	5.92	0.03	8e−04	···
ARDeq	−321.84	12	667.68	668.08	3.71	0.11	6e−04	···
ER	−362.02	1	726.04	726.05	61.67	0	8e−04	···
SYM	−327.4	6	666.8	666.91	2.54	0.19	0.0012	···
SYMeq**	−326.13	6	664.26	664.37	0	0.67	0.0012	···
ORD	−337.6	6	687.2	687.31	22.94	0	0	···
ORDeq	−336.69	6	685.38	685.48	21.11	0	0	···
ORDSYM	−346.2	3	698.39	698.42	34.05	0	0.0019	···
ORDSYMeq	−345.06	3	696.11	696.14	31.77	0	0.0019	···
ORDER	−348.11	1	698.22	698.22	33.85	0	0.0024	···

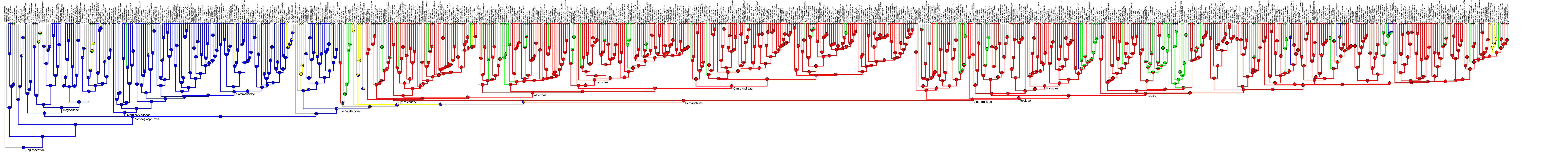

MP ancestral state reconstruction using ancestral.pars (R:phangorn)
232_B. Perianth merism (3−state) (D2c), 70 steps

- trimerous
- tetramerous
- pentamerous

Node	MP state(s)
Angiospermae	trimerous
Mesangiospermae	trimerous
Magnoliidae	trimerous
Monocotyledonae	trimerous
Eudicotyledonae	trimerous
Commelinidae	trimerous
Pentapetalae	pentamerous
Superasteridae	pentamerous
Asteridae	pentamerous
Lamiidae	pentamerous
Campanulidae	pentamerous
Superrosidae	pentamerous
Rosidae	pentamerous
Malvidae	pentamerous
Fabidae	pentamerous

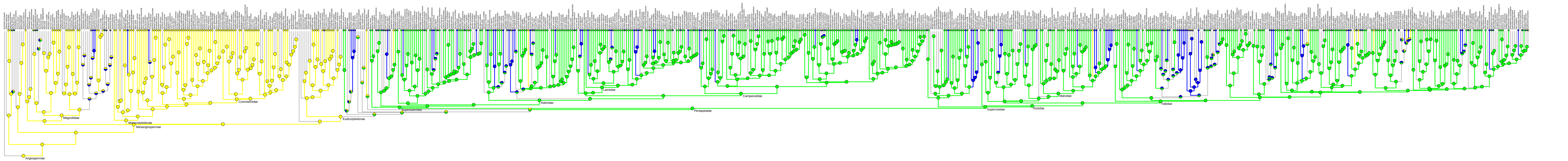

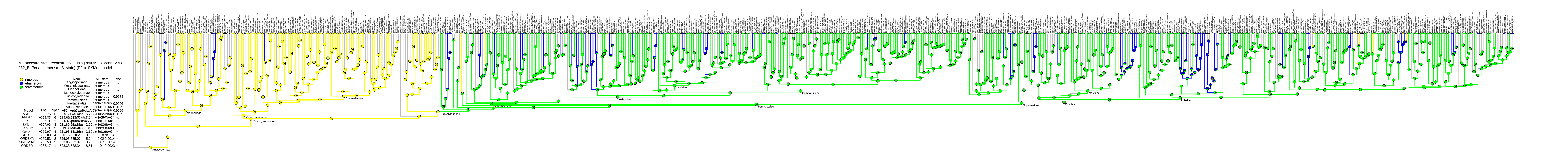
ML ancestral state reconstruction using rayDISC (R:corHMM)
232_B. Perianth merism (3−state) (D2c), SYMeq model
trimerous
tetramerous
pentamerous
Node ML state Prob
Angiospermae trimerous 1
Mesangiospermae trimerous 1
Magnoliidae trimerous 1
Monocotyledonae trimerous 1
Eudicotyledonae trimerous 0.9574
Commelinidae trimerous 1
Pentapetalae pentamerous 0.9996
Superasteridae pentamerous 0.9999
Model LogL Npar AIC
ARD −256.75 6 525.5
ARDeq −255.83 6
ER −282.3 1
SYM −257.93 3 521.85
SYMeq* −256.9 3 519.8
ORD −256.97 4 521.93
ORDeq −256.08 4 520.15 520.2 0.38 0.28 9e−04
ORDSYM −260.53 2 525.05 525.07 5.24 0.02 0.0014
ORDSYMeq −259.53 2 523.06 523.07 3.25 0.07 0.0014
ORDER −263.17 1 528.33 528.34 8.51 0 0.0023
Angiospermae
Magnoliidae
Monocotyledonae
Mesangiospermae
Commelinidae
Eudicotyledonae
Superasteridae
Pentapetalae
Asteridae
Lamiidae
Campanulidae
Superrosidae
Rosidae
Malvidae
Fabidae

MP ancestral state reconstruction using ancestral.pars
(R:phangorn)
234_A. Perianth differentiation (binary) (D2d), 64 steps

- undifferentiated
- differentiated

Node	MP state(s)
Angiospermae	undifferentiated
Mesangiospermae	undifferentiated
Magnoliidae	undifferentiated / differentiated
Monocotyledonae	undifferentiated
Eudicotyledonae	undifferentiated
Commelinidae	undifferentiated
Pentapetalae	differentiated
Superasteridae	differentiated
Asteridae	differentiated
Lamiidae	differentiated
Campanulidae	differentiated
Superrosidae	differentiated
Rosidae	differentiated
Malvidae	differentiated
Fabidae	differentiated

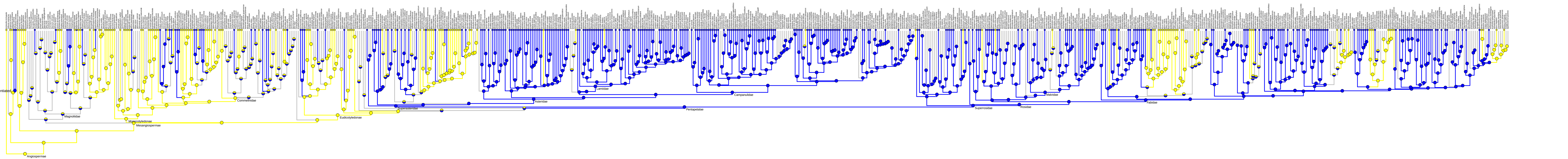

ML ancestral state reconstruction using rayDISC (R:corHMM)
234_A. Perianth differentiation (binary) (D2d), ARDeq model

- undifferentiated
- differentiated

Node	ML state	Prob
Angiospermae	undifferentiated	1
Mesangiospermae	undifferentiated	1
Magnoliidae	undifferentiated	0.9993
Monocotyledonae	undifferentiated	0.9999
Eudicotyledonae	undifferentiated	0.9759
Commelinidae	undifferentiated	0.9999
Pentapetalae	differentiated	0.9522
Superasteridae	differentiated	0.9545
Asteridae	differentiated	0.9984
Lamiidae	differentiated	0.9999
Campanulidae	differentiated	1
Superrosidae	differentiated	0.9908
Rosidae	differentiated	0.9943
Malvidae	differentiated	0.9996
Fabidae	differentiated	0.9957

Model	LogL	Npar	AIC	AICc	DeltaAICc	w	q01	q10
ARD	−198.71	2	401.43	401.44	1.3	0.34	0.0041	[illegible]
ARDeq**	−198.06	2	400.13	400.14	0	0.66	0.0041	0.0014
ER	−207.3	1	416.59	416.6	16.46	0	0.0024	0.0024
UNI01	−231.39	1	464.78	464.79	64.65	0	0.0083	
UNI10	−233.32	1	468.64	468.65	68.51	0		0.0032

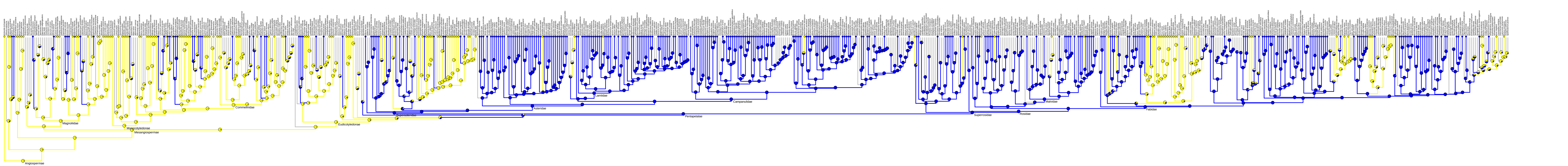

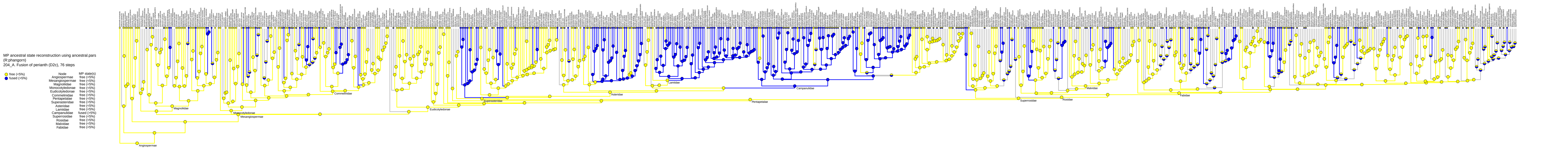
MP ancestral state reconstruction using ancestral.pars
(R:phangorn)
204_A. Fusion of perianth (D2c), 76 steps
free (<5%)
fused (>5%)
Node
MP state(s)
Angiospermae free (<5%)
Mesangiospermae free (<5%)
Magnoliidae free (<5%)
Monocotyledonae free (<5%)
Eudicotyledonae free (<5%)
Commelinidae free (<5%)
Pentapetalae free (<5%)
Superasteridae free (<5%)
Asteridae free (<5%)
Lamiidae free (<5%)
Campanulidae fused (>5%)
Superrosidae free (<5%)
Rosidae free (<5%)
Malvidae free (<5%)
Fabidae free (<5%)
Magnoliidae
Monocotyledonae
Mesangiospermae
Angiospermae
Commelinidae
Eudicotyledonae
Superasteridae
Asteridae
Lamiidae
Campanulidae
Pentapetalae
Superrosidae
Rosidae
Malvidae
Fabidae

ML ancestral state reconstruction using rayDISC (R:corHMM)
204_A. Fusion of perianth (D2c), ER model

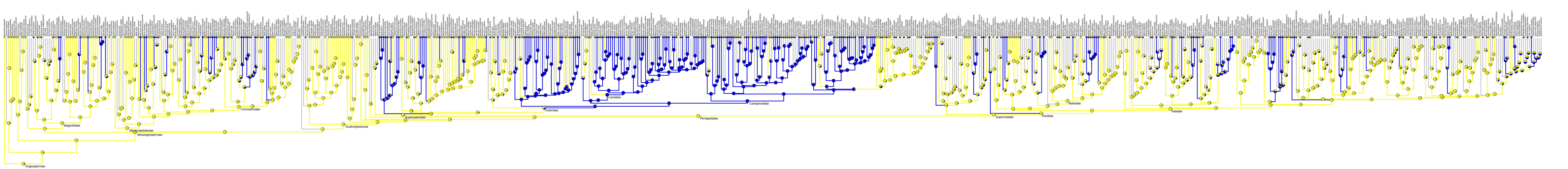

free (<5%)
fused (>5%)

Node	ML state	Prob
Angiospermae	free (<5%)	0.9237
Mesangiospermae	free (<5%)	1
Magnoliidae	free (<5%)	0.9988
Monocotyledonae	free (<5%)	0.9995
Eudicotyledonae	free (<5%)	0.9998
Commelinidae	free (<5%)	0.9968
Pentapetalae	free (<5%)	0.9943
Superasteridae	free (<5%)	0.9652
Asteridae	free (<5%)	0.697
Lamiidae	fused (>5%)	0.9779
Campanulidae	fused (>5%)	0.9765
Superrosidae	free (<5%)	0.9949
Rosidae	free (<5%)	0.9953
Malvidae	free (<5%)	0.9964
Fabidae	free (<5%)	0.9998

Model	LogL	Npar	AIC	AICc	DeltaAICc	w	q01	q10
ARD	−225.72	2	455.45	455.46	1.69	0.2	0.0041	0.0035
ARDeq	−225.16	2	454.31	454.33	0.55	0.35	0.0041	0.0035
ER*	−225.89	1	453.77	453.78	0	0.46	0.004	0.004
UNI01	−241	1	484	484	30.23	0	0.0046	
UNI10	−237.57	1	477.15	477.15	23.38	0		0.0078

MP ancestral state reconstruction using ancestral.pars (R:phangorn)
207_A. Symmetry of perianth (binary) (D2d), 57 steps

- actinomorphic
- zygomorphic

Node	MP state(s)
Angiospermae	actinomorphic
Mesangiospermae	actinomorphic
Magnoliidae	actinomorphic
Monocotyledonae	actinomorphic
Eudicotyledonae	actinomorphic
Commelinidae	actinomorphic
Pentapetalae	actinomorphic
Superasteridae	actinomorphic
Asteridae	actinomorphic
Lamiidae	actinomorphic
Campanulidae	actinomorphic
Superrosidae	actinomorphic
Rosidae	actinomorphic
Malvidae	actinomorphic
Fabidae	actinomorphic

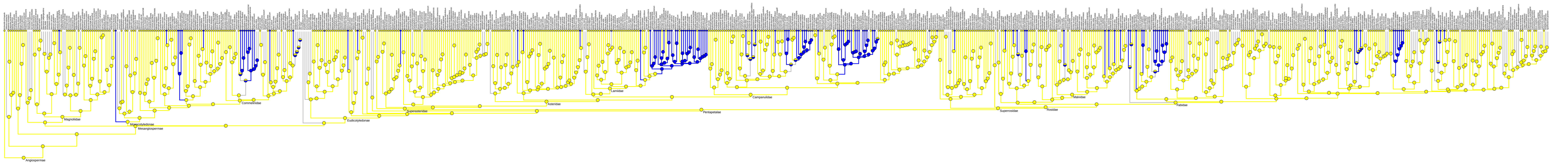

ML ancestral state reconstruction using rayDISC (R:corHMM)
207_A. Symmetry of perianth (binary) (D2d), ARDeq model

- actinomorphic
- zygomorphic

Node	ML state	Prob
Angiospermae	actinomorphic	0.9974
Mesangiospermae	actinomorphic	0.9999
Magnoliidae	actinomorphic	0.9997
Monocotyledonae	actinomorphic	0.9983
Eudicotyledonae	actinomorphic	0.9997
Commelinidae	actinomorphic	0.9961
Pentapetalae	actinomorphic	1
Superasteridae	actinomorphic	1
Asteridae	actinomorphic	1
Lamiidae	actinomorphic	0.9988
Campanulidae	actinomorphic	1
Superrosidae	actinomorphic	1
Rosidae	actinomorphic	0.9999
Malvidae	actinomorphic	0.9998
Fabidae	actinomorphic	0.9994

Model	LogL	Npar	AIC	AICc	DeltaAICc	w	q01	q10
ARD	−211.34	2	426.69	426.7	0.9	0.39	0.0013	0.0061
ARDeq**	−210.89	2	425.79	425.8	0	0.61	0.0013	0.006
ER	−221.54	1	445.08	445.09	19.28	0	0.0015	0.0015
UNI01	−270.67	1	543.34	543.34	117.54	0	0.0018	
UNI10	−221.29	1	444.57	444.58	18.77	0		0.0158

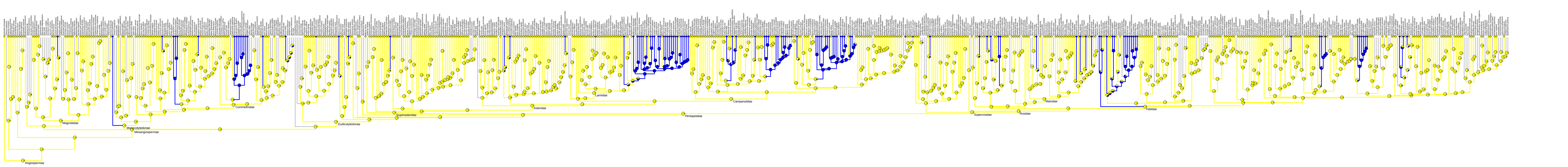

MP ancestral state reconstruction using ancestral.pars
(R:phangorn)
301_B. Number of fertile stamens (3−state) (D2c), 155 steps

- one to five (1−5)
- six to ten (6−10)
- more than ten (>10)

Node	MP state(s)
Angiospermae	more than ten (>10)
Mesangiospermae	one to five (1−5) / more than ten (>10)
Magnoliidae	more than ten (>10)
Monocotyledonae	six to ten (6−10)
Eudicotyledonae	one to five (1−5) / six to ten (6−10)
Commelinidae	six to ten (6−10)
Pentapetalae	six to ten (6−10)
Superasteridae	six to ten (6−10)
Asteridae	one to five (1−5) / six to ten (6−10)
Lamiidae	one to five (1−5)
Campanulidae	one to five (1−5)
Superrosidae	six to ten (6−10)
Rosidae	six to ten (6−10)
Malvidae	six to ten (6−10)
Fabidae	six to ten (6−10)

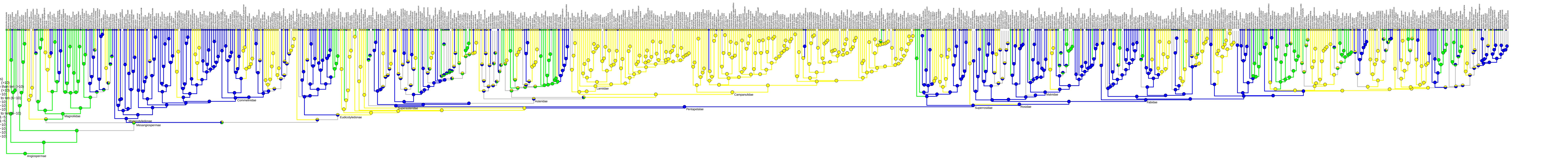

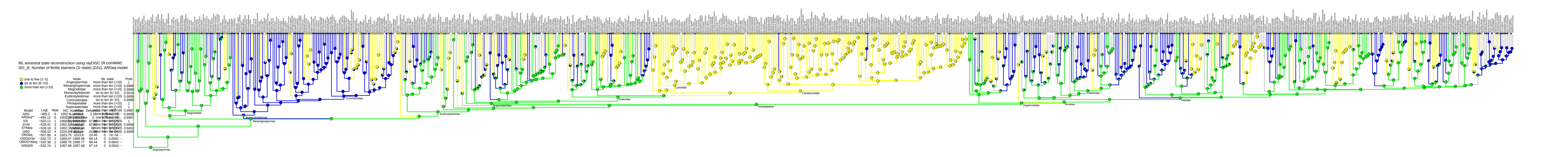
ML ancestral state reconstruction using rayDISC (R:corHMM)
301_B. Number of fertile stamens (3−state) (D2c), ARDeq model
one to five (1−5)
six to ten (6−10)
more than ten (>10)
Node
ML state
Prob
Angiospermae
more than ten (>10)
1
Mesangiospermae
more than ten (>10)
0.9998
Magnoliidae
more than ten (>10)
0.9998
Monocotyledonae
six to ten (6−10)
0.5016
Eudicotyledonae
more than ten (>10)
0.9999
Commelinidae
six to ten (6−10)
0.9998
Pentapetalae
more than ten (>10)
1
Superasteridae
more than ten (>10)
1
Model
LogL
Npar
AIC
ORDeq −507.88 4 1023.75 1023.8 23.46 0 7e−04 ···
ORDSYM −532.73 2 1069.47 1069.48 69.14 0 0.0041 ···
ORDSYMeq −532.38 2 1068.76 1068.77 68.44 0 0.0041 ···
ORDER −532.74 1 1067.48 1067.48 67.14 0 0.0042 ···
Angiospermae
Magnoliidae
Mesangiospermae
Commelinidae
Eudicotyledonae
Superasteridae
Asteridae
Lamiidae
Campanulidae
Pentapetalae
Superrosidae
Rosidae
Malvidae
Fabidae

MP ancestral state reconstruction using ancestral.pars
(R:phangorn)
301_C. Number of fertile stamens (binary) (D2c), 93 steps

- one to six (1−6)
- more than six (>6)

Node	MP state(s)
Angiospermae	more than six (>6)
Mesangiospermae	one to six (1−6) / more than six (>6)
Magnoliidae	one to six (1−6) / more than six (>6)
Monocotyledonae	one to six (1−6)
Eudicotyledonae	one to six (1−6)
Commelinidae	one to six (1−6)
Pentapetalae	more than six (>6)
Superasteridae	more than six (>6)
Asteridae	more than six (>6)
Lamiidae	one to six (1−6)
Campanulidae	one to six (1−6)
Superrosidae	more than six (>6)
Rosidae	more than six (>6)
Malvidae	more than six (>6)
Fabidae	more than six (>6)

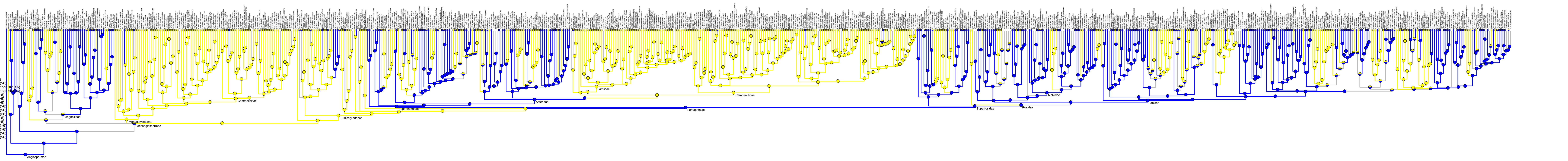

ML ancestral state reconstruction using rayDISC (R:corHMM)
301_C. Number of fertile stamens (binary) (D2c), ARDeq model

- one to six (1−6)
- more than six (>6)

Node	ML state	Prob
Angiospermae	more than six (>6)	1
Mesangiospermae	more than six (>6)	0.9995
Magnoliidae	more than six (>6)	1
Monocotyledonae	more than six (>6)	0.5454
Eudicotyledonae	more than six (>6)	0.9986
Commelinidae	one to six (1−6)	1
Pentapetalae	more than six (>6)	1
Superasteridae	more than six (>6)	1
Asteridae	more than six (>6)	0.9998
Lamiidae	one to six (1−6)	0.9988
Campanulidae	one to six (1−6)	0.9934
Superrosidae	more than six (>6)	1
Rosidae	more than six (>6)	0.9999
Malvidae	more than six (>6)	1
Fabidae	more than six (>6)	1

Model	LogL	Npar	AIC	AICc	DeltaAICc	w	q01	q10
ARD	−291	2	585.99	586.[illegible]	1.38	0.33	5e−04	0.0065
ARDeq**	−290.31	2	584.62	584.63	0	0.67	5e−04	0.0055
ER	−322.94	1	647.87	647.88	63.24	0	0.0029	0.0029
UNI01	−366.41	1	734.83	734.83	150.2	0	0.0044	
UNI10	−330.35	1	662.7	662.7	78.07	0		0.0069

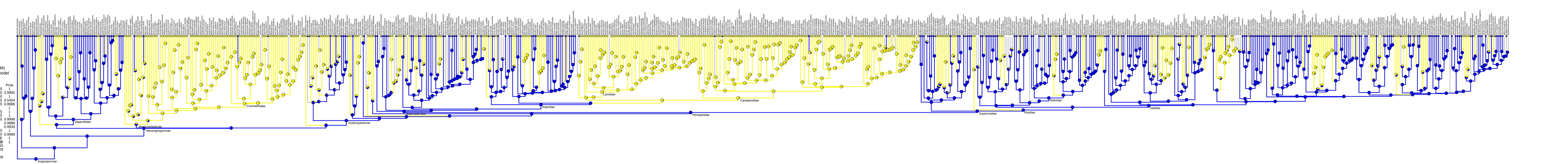

MP ancestral state reconstruction using ancestral.pars
(R:phangorn)
330_A. Androecium structural phyllotaxy (binary) (D2d), 12 steps

- whorled
- spiral

Node	MP state(s)
Angiospermae	whorled / spiral
Mesangiospermae	whorled / spiral
Magnoliidae	whorled / spiral
Monocotyledonae	whorled
Eudicotyledonae	whorled
Commelinidae	whorled
Pentapetalae	whorled
Superasteridae	whorled
Asteridae	whorled
Lamiidae	whorled
Campanulidae	whorled
Superrosidae	whorled
Rosidae	whorled
Malvidae	whorled
Fabidae	whorled

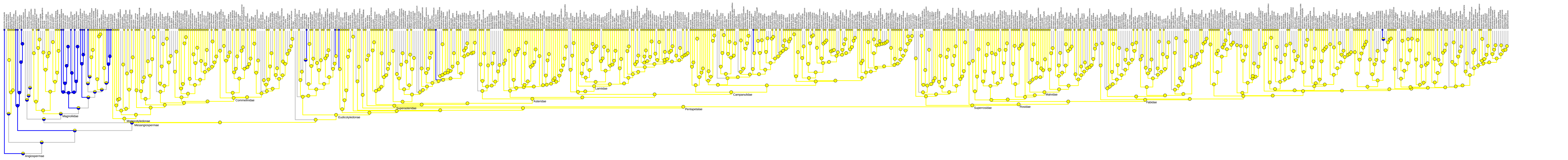

ML ancestral state reconstruction using rayDISC (R:corHMM)
330_A. Androecium structural phyllotaxy (binary) (D2d), ARD model

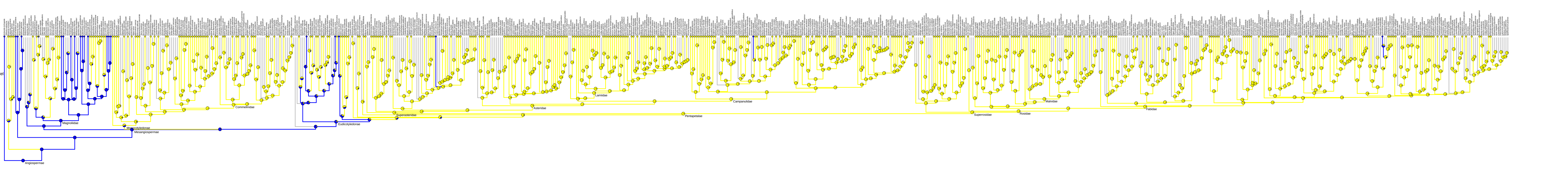

- whorled (yellow)
- spiral (blue)

Node	ML state	Prob
Angiospermae	spiral	0.9988
Mesangiospermae	spiral	0.9794
Magnoliidae	spiral	0.982
Monocotyledonae	whorled	0.5901
Eudicotyledonae	spiral	0.8243
Commelinidae	whorled	0.9999
Pentapetalae	whorled	0.9983
Superasteridae	whorled	0.9992
Asteridae	whorled	1
Lamiidae	whorled	1
Campanulidae	whorled	1
Superrosidae	whorled	0.9996
Rosidae	whorled	0.9998
Malvidae	whorled	1
Fabidae	whorled	1

Model	LogL	Npar	AIC	AICc	DeltaAICc	w	q01	q10
ARD**	−54.43	2	112.86	112.87	0	0.89	2e−04	0.0169
ARDeq	−57.81	2	119.62	119.64	6.76	0.03	4e−04	0
ER	−58.55	1	119.1	119.11	6.23	0.04	4e−04	4e−04
UNI01	−58.5	1	119.01	119.01	6.14	0.04	4e−04	
UNI10	−79.63	1	161.25	161.26	48.38	0		0.0181

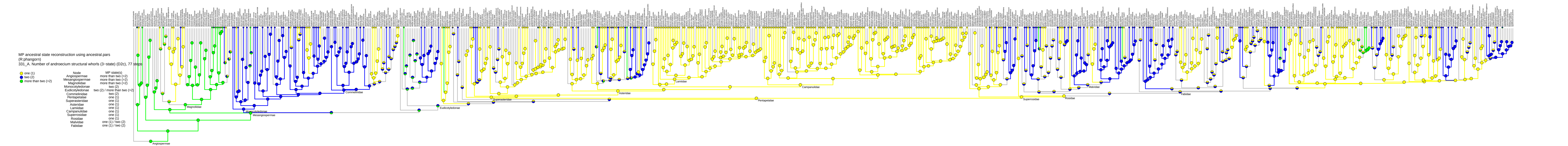
MP ancestral state reconstruction using ancestral.pars
(R:phangorn)
331_A. Number of androecium structural whorls (3−state) (D2c), 77 steps
one (1)
two (2)
more than two (>2)
Node
MP state(s)
Angiospermae
more than two (>2)
Mesangiospermae
more than two (>2)
Magnoliidae
more than two (>2)
Monocotyledonae
two (2)
Eudicotyledonae
two (2) / more than two (>2)
Commelinidae
two (2)
Pentapetalae
one (1)
Superasteridae
one (1)
Asteridae
one (1)
Lamiidae
one (1)
Campanulidae
one (1)
Superrosidae
one (1)
Rosidae
one (1)
Malvidae
one (1) / two (2)
Fabidae
one (1) / two (2)

ML ancestral state reconstruction using rayDISC (R:corHMM)
331_A. Number of androecium structural whorls (3−state) (D2c), ORDeq model

- one (1)
- two (2)
- more than two (>2)

Node	ML state	Prob
Angiospermae	more than two (>2)	1
Mesangiospermae	more than two (>2)	0.9882
Magnoliidae	more than two (>2)	0.99
Monocotyledonae	more than two (>2)	0.9386
Eudicotyledonae	more than two (>2)	0.8079
Commelinidae	two (2)	0.968
Pentapetalae	two (2)	0.9915
Superasteridae	two (2)	0.9948
Asteridae	two (2)	0.9999
Lamiidae	one (1)	0.9982
Campanulidae	one (1)	0.9904
Superrosidae	two (2)	0.9976
Rosidae	two (2)	0.9986
Malvidae	two (2)	0.9997
Fabidae	two (2)	0.9999

Model	LogL	Npar	AIC	AICc	DeltaAICc	wt	[illegible]	
ARD	−232.52	6	477.04	477.15	3.2	0.09	[illegible]	···
ARDeq	−231.47	6	474.94	475.05	1.1	0.27	[illegible]	···
ER	−279.55	1	561.1	561.11	87.16	0	0.0216	···
SYM	−254.85	3	515.7	515.73	41.78	0	0.0033	···
SYMeq	−254.19	3	514.37	514.4	40.45	0	0.0033	···
ORD	−233.99	4	475.98	476.04	2.09	0.17	[illegible]	···
ORDeq*	−232.95	4	473.9	473.95	0	0.47	0	···
ORDSYM	−255.62	2	515.24	515.26	41.31	0	0.0034	···
ORDSYMeq	−254.9	2	513.8	513.82	39.87	0	0.0034	···
ORDER	−261.04	1	524.09	524.09	50.15	0	0.0027	···

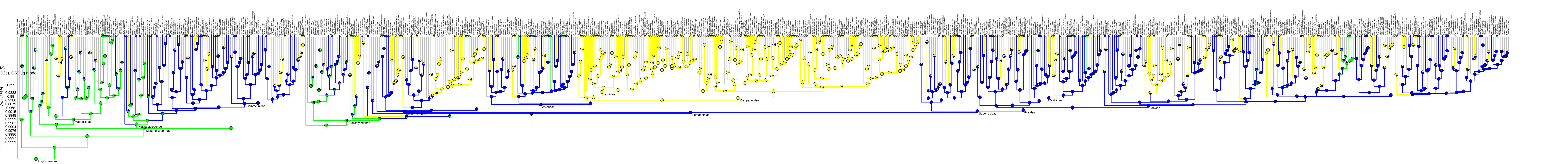

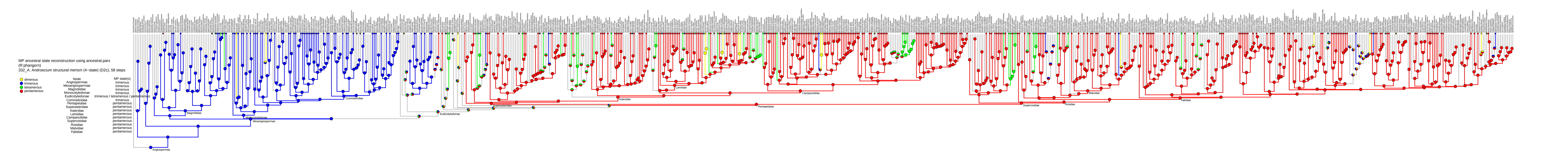
MP ancestral state reconstruction using ancestral.pars
(R:phangorn)
332_A. Androecium structural merism (4−state) (D2c), 58 steps
dimerous
trimerous
tetramerous
pentamerous
Node | MP state(s)
Angiospermae | trimerous
Mesangiospermae | trimerous
Magnoliidae | trimerous
Monocotyledonae | trimerous
Eudicotyledonae | trimerous / tetramerous / pentamerous
Commelinidae | trimerous
Pentapetalae | pentamerous
Superasteridae | pentamerous
Asteridae | pentamerous
Lamiidae | pentamerous
Campanulidae | pentamerous
Superrosidae | pentamerous
Rosidae | pentamerous
Malvidae | pentamerous
Fabidae | pentamerous
Magnoliidae
Monocotyledonae
Mesangiospermae
Commelinidae
Eudicotyledonae
Superasteridae
Asteridae
Lamiidae
Campanulidae
Pentapetalae
Superrosidae
Rosidae
Malvidae
Fabidae
Angiospermae

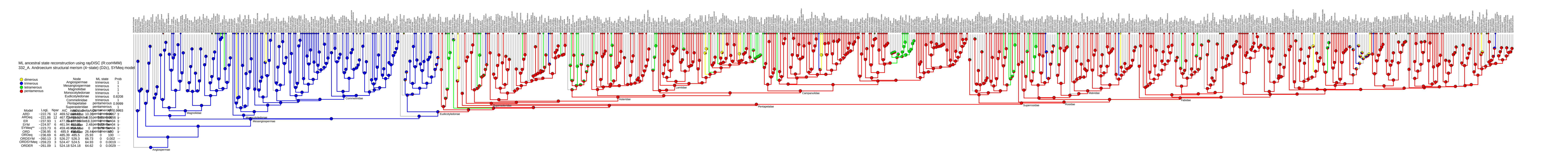
ML ancestral state reconstruction using rayDISC (R:corHMM)
332_A. Androecium structural merism (4−state) (D2c), SYMeq model
dimerous
trimerous
tetramerous
pentamerous
Node ML state Prob
Angiospermae trimerous 1
Mesangiospermae trimerous 1
Magnoliidae trimerous 1
Monocotyledonae trimerous 1
Eudicotyledonae trimerous 0.8208
Commelinidae trimerous 1
Pentapetalae pentamerous 0.9999
Superasteridae pentamerous 1
Asteridae pentamerous 0.9993
Lamiidae pentamerous 1
Campanulidae pentamerous 1
Superrosidae pentamerous 1
Rosidae pentamerous 1
Malvidae pentamerous 1
Fabidae pentamerous 1
Model LogL Npar AIC AICc DeltaAICc
ARD −222.76 12 469.52 10.36
ARDeq −221.86 12 8.55
ER −237.93 1 18.3
SYM −224.97 6 461.94 2.48
SYMeq** −223.73 6 459.46 0
ORD −236.95 6 485.9 26.44
ORDeq −236.69 6 485.39 485.5 25.93 0 100 ···
ORDSYM −260.13 3 526.27 526.3 66.73 0 0.002 ···
ORDSYMeq −259.23 3 524.47 524.5 64.93 0 0.0019 ···
ORDER −261.09 1 524.18 524.18 64.62 0 0.0029 ···
Angiospermae
Mesangiospermae
Magnoliidae
Monocotyledonae
Commelinidae
Eudicotyledonae
Pentapetalae
Superasteridae
Asteridae
Lamiidae
Campanulidae
Superrosidae
Rosidae
Malvidae
Fabidae

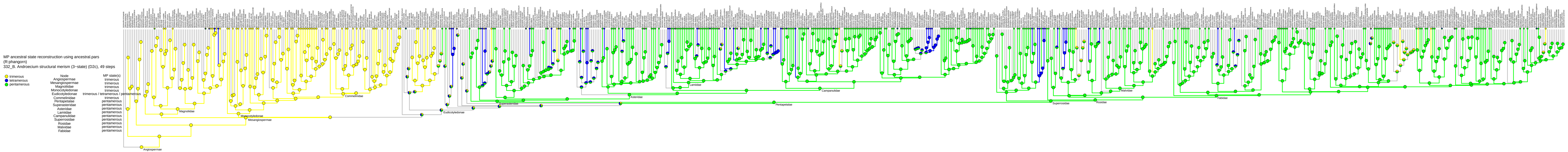
MP ancestral state reconstruction using ancestral.pars
(R:phangorn)
332_B. Androecium structural merism (3−state) (D2c), 49 steps
trimerous
tetramerous
pentamerous
Node | MP state(s)
Angiospermae | trimerous
Mesangiospermae | trimerous
Magnoliidae | trimerous
Monocotyledonae | trimerous
Eudicotyledonae | trimerous / tetramerous / pentamerous
Commelinidae | trimerous
Pentapetalae | pentamerous
Superasteridae | pentamerous
Asteridae | pentamerous
Lamiidae | pentamerous
Campanulidae | pentamerous
Superrosidae | pentamerous
Rosidae | pentamerous
Malvidae | pentamerous
Fabidae | pentamerous
Magnoliidae
Commelinidae
Monocotyledonae
Mesangiospermae
Angiospermae
Eudicotyledonae
Superasteridae
Asteridae
Lamiidae
Campanulidae
Pentapetalae
Superrosidae
Rosidae
Malvidae
Fabidae

ML ancestral state reconstruction using rayDISC (R:corHMM)
332_B. Androecium structural merism (3−state) (D2c), ARDeq model

- trimerous
- tetramerous
- pentamerous

Node	ML state	Prob
Angiospermae	trimerous	0.9999
Mesangiospermae	trimerous	1
Magnoliidae	trimerous	1
Monocotyledonae	trimerous	1
Eudicotyledonae	trimerous	0.8752
Commelinidae	trimerous	1
Pentapetalae	tetramerous	0.9999
Superasteridae	tetramerous	1
Asteridae	tetramerous	1
Lamiidae	tetramerous	0.9919
Campanulidae	tetramerous	0.9624
Superrosidae	tetramerous	1
Rosidae	tetramerous	0.9994
Malvidae	pentamerous	0.5774
Fabidae	tetramerous	0.9474

Model	LogL	Npar	AIC	AICc	DeltaAICc	w	q01	···
ARD	−170.44	6	352.88	352.99	1.97	0.26	8e−04	···
ARDeq**	−169.46	6	350.91	351.02	0	0.71	8e−04	···
ER	−186.52	1	375.04	375.05	24.03	0	0.0011	···
SYM	−177	3	360	360.03	9.01	0.01	4e−04	···
SYMeq	−176	3	358.01	358.04	7.02	0.02	4e−04	···
ORD	−178.86	4	365.72	365.77	14.75	0	7e−04	···
ORDeq	−178.69	4	365.37	365.43	14.41	0	1e−04	···
ORDSYM	−186.31	2	376.62	376.64	25.62	0	0.0019	···
ORDSYMeq	−185.41	2	374.83	374.84	23.82	0	0.0018	···
ORDER	−187.22	1	376.44	376.44	25.42	0	0.0026	···

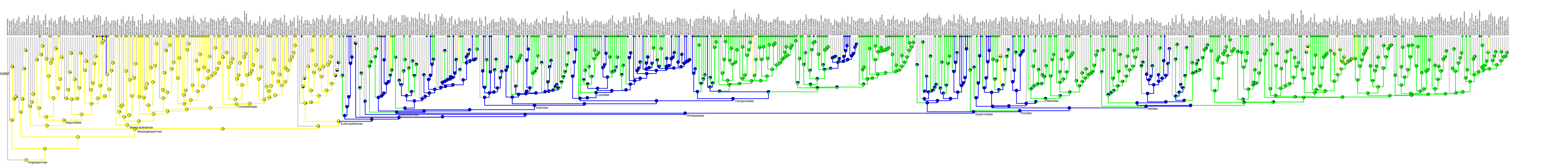

MP ancestral state reconstruction using ancestral.pars
(R:phangorn)
305_A. Filament (binary) (D2d), 52 steps

- laminar (wide)
- typical (narrow)

Node	MP state(s)
Angiospermae	laminar (wide)
Mesangiospermae	laminar (wide)
Magnoliidae	laminar (wide)
Monocotyledonae	typical (narrow)
Eudicotyledonae	typical (narrow)
Commelinidae	typical (narrow)
Pentapetalae	typical (narrow)
Superasteridae	typical (narrow)
Asteridae	typical (narrow)
Lamiidae	typical (narrow)
Campanulidae	typical (narrow)
Superrosidae	typical (narrow)
Rosidae	typical (narrow)
Malvidae	typical (narrow)
Fabidae	typical (narrow)

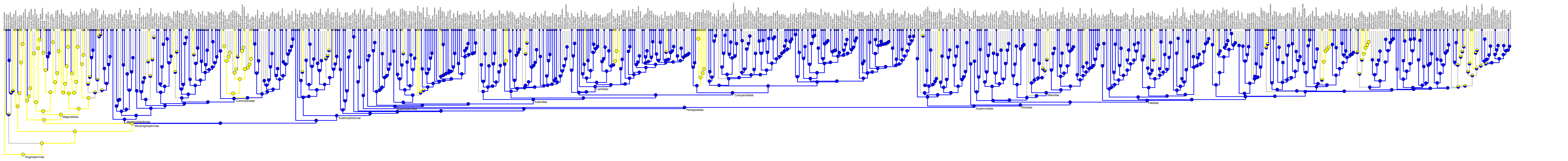

ML ancestral state reconstruction using rayDISC (R:corHMM)
305_A. Filament (binary) (D2d), ARDeq model

- laminar (wide)
- typical (narrow)

Node	ML state	Prob
Angiospermae	laminar (wide)	0.9845
Mesangiospermae	laminar (wide)	0.7691
Magnoliidae	laminar (wide)	0.9794
Monocotyledonae	typical (narrow)	0.6794
Eudicotyledonae	typical (narrow)	0.9083
Commelinidae	typical (narrow)	0.9014
Pentapetalae	typical (narrow)	0.9999
Superasteridae	typical (narrow)	0.9999
Asteridae	typical (narrow)	0.9998
Lamiidae	typical (narrow)	0.9992
Campanulidae	typical (narrow)	0.996
Superrosidae	typical (narrow)	0.9999
Rosidae	typical (narrow)	0.9999
Malvidae	typical (narrow)	0.9987
Fabidae	typical (narrow)	0.9999

Model	LogL	Npar	AIC	AICc	DeltaAICc	[illegible]	q01	q10
ARD	−171.41	2	346.82	346.84	0.58	0.43	0.0064	0.002
ARDeq**	−171.12	2	346.24	346.26	0	0.57	0.0066	0.002
ER	−176.86	1	355.71	355.72	9.46	0.01	0.0022	0.0022
UNI01	−188.13	1	378.27	378.27	32.02	0	0.0135	
UNI10	−188.56	1	379.11	379.12	32.86	0		0.0022

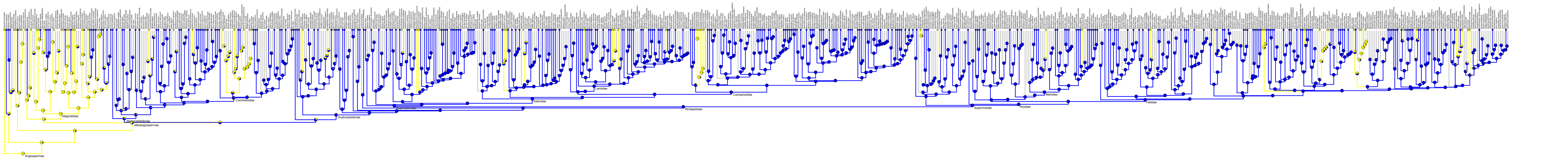

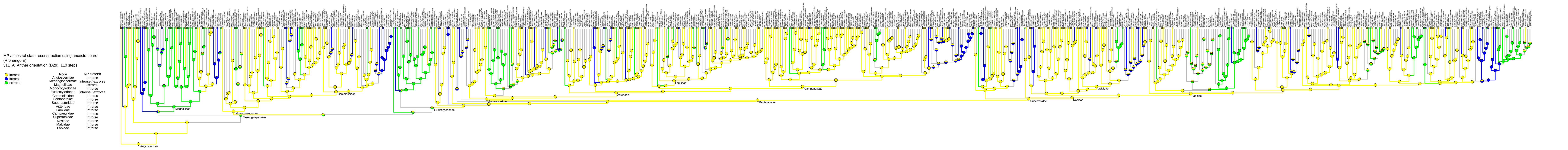
MP ancestral state reconstruction using ancestral.pars
(R:phangorn)
311_A. Anther orientation (D2d), 110 steps
introrse
latrorse
extrorse
Node
MP state(s)
Angiospermae introrse
Mesangiospermae introrse / extrorse
Magnoliidae extrorse
Monocotyledonae introrse
Eudicotyledonae introrse / extrorse
Commelinidae introrse
Pentapetalae introrse
Superasteridae introrse
Asteridae introrse
Lamiidae introrse
Campanulidae introrse
Superrosidae introrse
Rosidae introrse
Malvidae introrse
Fabidae introrse
Angiospermae
Mesangiospermae
Magnoliidae
Monocotyledonae
Commelinidae
Eudicotyledonae
Superasteridae
Pentapetalae
Asteridae
Lamiidae
Campanulidae
Superrosidae
Rosidae
Malvidae
Fabidae

ML ancestral state reconstruction using rayDISC (R:corHMM)
311_A. Anther orientation (D2d), ER model

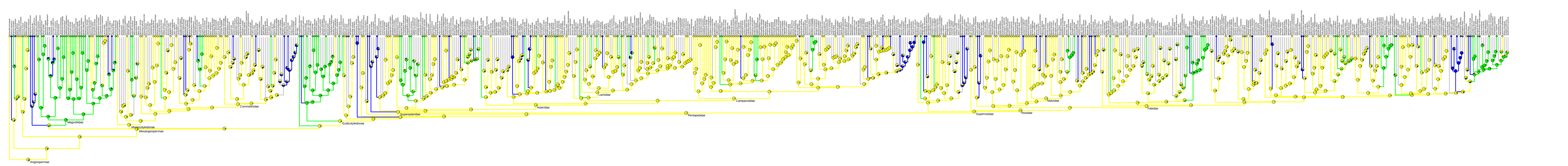

introrse
latrorse
extrorse

Node	ML state	Prob
Angiospermae	introrse	0.8346
Mesangiospermae	introrse	0.9161
Magnoliidae	extrorse	0.9217
Monocotyledonae	introrse	0.9488
Eudicotyledonae	introrse	0.9321
Commelinidae	introrse	0.9939
Pentapetalae	introrse	1
Superasteridae	introrse	0.9998
Asteridae	introrse	0.9997
Lamiidae	introrse	0.9998
Campanulidae	introrse	1
Superrosidae	introrse	1
Rosidae	introrse	1
Malvidae	introrse	0.9999
Fabidae	introrse	0.9997

Model	LogL	Npar	AIC	AICc	DeltaAICc	w	q01	
ARD	−344.54	6	701.08	701.19	2.19	0.11	0.0037	···
ARDeq	−344.25	6	700.5	700.6	1.61	0.14	0.0036	···
ER*	−348.5	1	698.99	699	0	0.32	0.0026	···
SYM	−347.33	3	700.65	700.68	1.68	0.14	0.003	···
SYMeq	−346.55	3	699.11	699.14	0.14	0.3	0.0029	···

MP ancestral state reconstruction using ancestral.pars
(R:phangorn)
312_A. Anther attachment (binary) (D2d), 97 steps

- basifixed
- dorsifixed

Node	MP state(s)
Angiospermae	basifixed
Mesangiospermae	basifixed
Magnoliidae	basifixed
Monocotyledonae	basifixed
Eudicotyledonae	basifixed
Commelinidae	basifixed
Pentapetalae	basifixed
Superasteridae	basifixed
Asteridae	basifixed
Lamiidae	basifixed
Campanulidae	dorsifixed
Superrosidae	basifixed / dorsifixed
Rosidae	dorsifixed
Malvidae	dorsifixed
Fabidae	dorsifixed

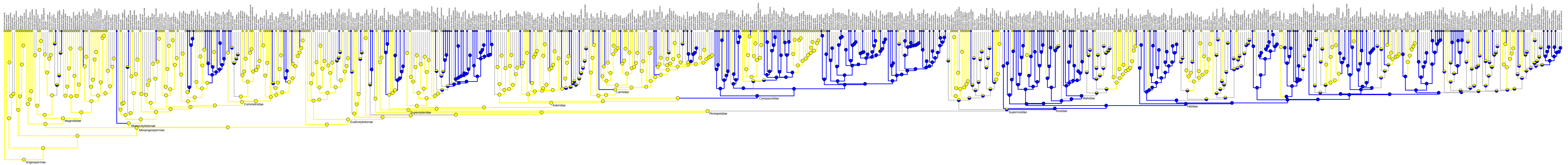

ML ancestral state reconstruction using rayDISC (R:corHMM)
312_A. Anther attachment (binary) (D2d), ARDeq model

- basifixed
- dorsifixed

Node	ML state	Prob
Angiospermae	basifixed	0.9776
Mesangiospermae	basifixed	0.9575
Magnoliidae	basifixed	0.9812
Monocotyledonae	basifixed	0.9448
Eudicotyledonae	basifixed	0.6833
Commelinidae	basifixed	0.9554
Pentapetalae	dorsifixed	0.9515
Superasteridae	dorsifixed	0.9568
Asteridae	dorsifixed	0.9534
Lamiidae	dorsifixed	0.7834
Campanulidae	dorsifixed	0.9902
Superrosidae	dorsifixed	0.9558
Rosidae	dorsifixed	0.9737
Malvidae	dorsifixed	[illegible]
Fabidae	dorsifixed	[illegible]

Model	LogL	Npar	AIC	AICc	DeltaAICc	w	q01	q10
ARD	−264.88	2	533.76	533.78	0.53	0.41	[illegible]	0.0074
ARDeq**	−264.62	2	533.23	533.25	0	0.53	0.0039	0.0073
ER	−267.81	1	537.61	537.62	4.37	0.06	0.0054	0.0054
UNI01	−293.06	1	588.12	588.13	54.88	0	0.0057	
UNI10	−276.55	1	555.11	555.11	21.86	0		0.0075

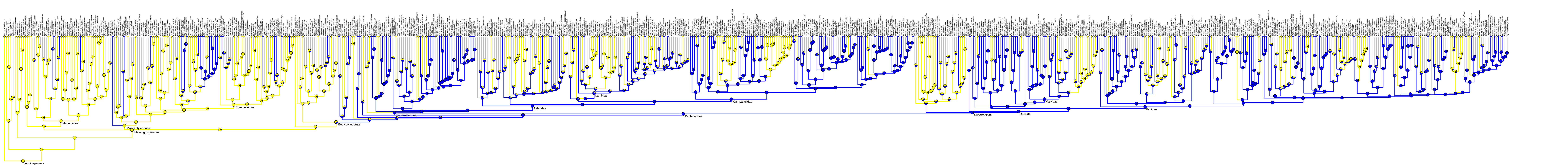

MP ancestral state reconstruction using ancestral.pars
(R:phangorn)
313_A. Anther dehiscence (3−state) (D2d), 10 steps

- longitudinal slit
- H−valvate
- flap−valvate

Node	MP state(s)
Angiospermae	longitudinal slit
Mesangiospermae	longitudinal slit
Magnoliidae	longitudinal slit
Monocotyledonae	longitudinal slit
Eudicotyledonae	longitudinal slit
Commelinidae	longitudinal slit
Pentapetalae	longitudinal slit
Superasteridae	longitudinal slit
Asteridae	longitudinal slit
Lamiidae	longitudinal slit
Campanulidae	longitudinal slit
Superrosidae	longitudinal slit
Rosidae	longitudinal slit
Malvidae	longitudinal slit
Fabidae	longitudinal slit

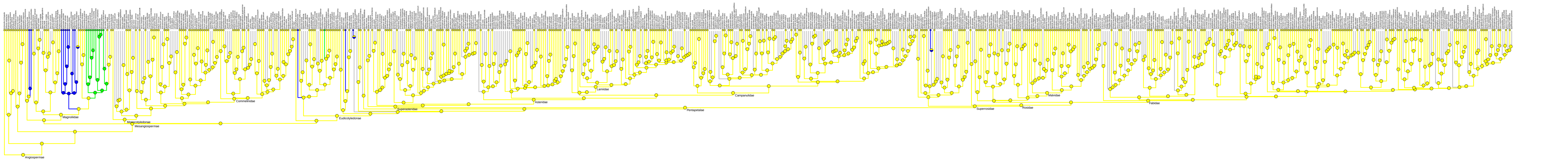

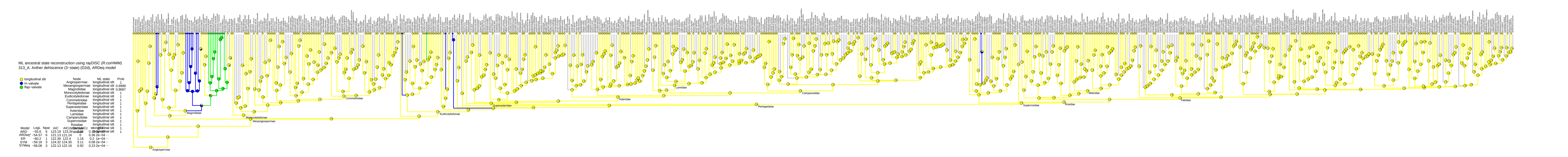
ML ancestral state reconstruction using rayDISC (R:corHMM)
313_A. Anther dehiscence (3−state) (D2d), ARDeq model
longitudinal slit
H−valvate
flap−valvate
Node | ML state | Prob
Angiospermae | longitudinal slit | 1
Mesangiospermae | longitudinal slit | 0.9999
Magnoliidae | longitudinal slit | 0.9687
Monocotyledonae | longitudinal slit | 1
Eudicotyledonae | longitudinal slit | 1
Commelinidae | longitudinal slit | 1
Pentapetalae | longitudinal slit | 1
Superasteridae | longitudinal slit | 1
Asteridae | longitudinal slit | 1
Lamiidae | longitudinal slit | 1
Campanulidae | longitudinal slit | 1
Superrosidae | longitudinal slit | 1
Rosidae | longitudinal slit | 1
Malvidae | longitudinal slit | 1
Fabidae | longitudinal slit | 1
Model | LogL | Npar | AIC | AICc | DeltaAICc | wAICc
ARD | −55.6 | 6 | 123.19 | 123.3 | 2.06 | 0.13 2e−04 ···
ARDeq* | −54.57 | 6 | 121.13 | 121.24 | 0 | 0.36 2e−04 ···
ER | −60.2 | 1 | 122.39 | 122.4 | 1.16 | 0.2 1e−04 ···
SYM | −59.16 | 3 | 124.32 | 124.35 | 3.11 | 0.08 2e−04 ···
SYMeq | −58.06 | 3 | 122.13 | 122.16 | 0.92 | 0.23 2e−04 ···
Magnoliidae
Monocotyledonae
Mesangiospermae
Commelinidae
Eudicotyledonae
Superasteridae
Asteridae
Lamiidae
Campanulidae
Pentapetalae
Superrosidae
Rosidae
Malvidae
Fabidae
Angiospermae

MP ancestral state reconstruction using ancestral.pars
(R:phangorn)
401_B. Number of structural carpels (5−state) (D2c), 186 steps

- one (1)
- two (2)
- three (3)
- four or five (4−5)
- more than five (>5)

Node	MP state(s)
Angiospermae	more than five (>5)
Mesangiospermae	more than five (>5)
Magnoliidae	more than five (>5)
Monocotyledonae	three (3)
Eudicotyledonae	more than five (>5)
Commelinidae	three (3)
Pentapetalae	three (3) / four or five (4−5)
Superasteridae	three (3) / four or five (4−5)
Asteridae	two (2)
Lamiidae	two (2)
Campanulidae	two (2)
Superrosidae	three (3) / four or five (4−5)
Rosidae	three (3) / four or five (4−5)
Malvidae	four or five (4−5)
Fabidae	four or five (4−5)

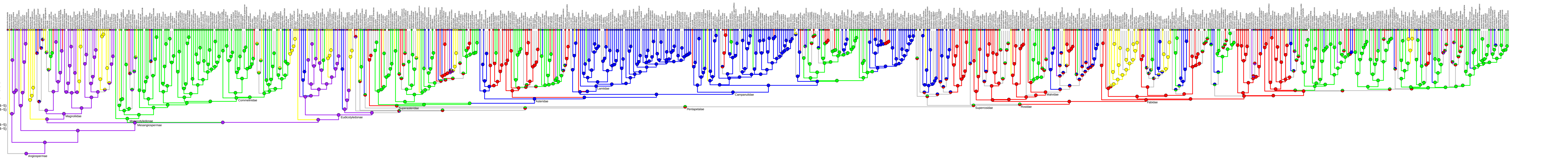

ML ancestral state reconstruction using rayDISC (R:corHMM)
401_B. Number of structural carpels (5−state) (D2c), ARDeq model

- one (1)
- two (2)
- three (3)
- four or five (4−5)
- more than five (>5)

Node	ML state	Prob
Angiospermae	more than five (>5)	1
Mesangiospermae	more than five (>5)	0.9999
Magnoliidae	more than five (>5)	0.9999
Monocotyledonae	more than five (>5)	0.7937
Eudicotyledonae	more than five (>5)	0.9913
Commelinidae	three (3)	0.9999
Pentapetalae	four or five (4−5)	0.9997
Superasteridae	four or five (4−5)	0.9999
Asteridae	four or five (4−5)	0.9831
Lamiidae	two (2)	0.9969
Campanulidae	two (2)	0.9614
Superrosidae	four or five (4−5)	0.9995
Rosidae	four or five (4−5)	0.9994
Malvidae	four or five (4−5)	0.9992
Fabidae	four or five (4−5)	0.9999

Model	LogL	Npar	AIC	AICc	DeltaAICc	[illegible]	[illegible]	⋯
ARD	−665.1	20	1370.2	1371.29	3.13	0.17	0.0011	⋯
ARDeq**	−663.53	20	1367.06	1368.15	0	0.83	0.0011	⋯
ER	−719.31	1	1440.61	1440.62	72.46	0	[illegible]	⋯
SYM	−684.82	10	1389.65	1389.93	21.77	0	[illegible]	⋯
SYMeq	−683.52	10	1387.05	1387.33	19.18	0	[illegible]	⋯
ORD	−757	8	1530.01	1530.19	162.04	0	[illegible]	⋯
ORDeq	−756.05	8	1528.11	1528.29	160.14	0	0	⋯
ORDSYM	−762.95	4	1533.89	1533.94	165.79	0	0.0038	⋯
ORDSYMeq	−762.22	4	1532.44	1532.5	164.34	0	0.0038	⋯
ORDER	−768.94	1	1539.87	1539.88	171.73	0	0.0055	⋯

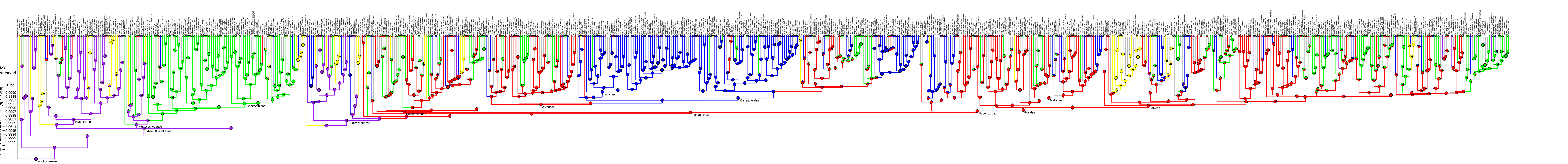

MP ancestral state reconstruction using ancestral.pars (R:phangorn)
400_A. Gynoecium phyllotaxy (D2d), 10 steps

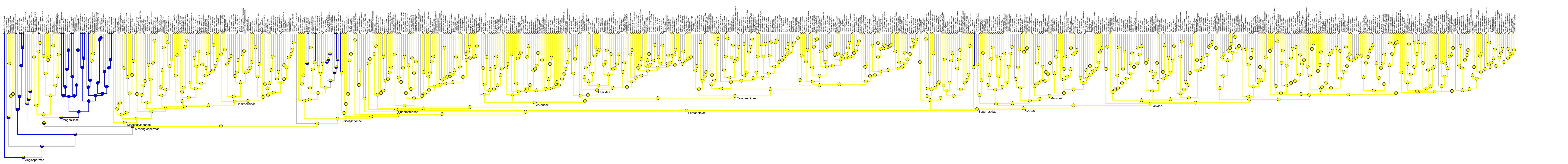

Node	MP state(s)
Angiospermae	whorled / spiral
Mesangiospermae	whorled / spiral
Magnoliidae	whorled / spiral
Monocotyledonae	whorled
Eudicotyledonae	whorled
Commelinidae	whorled
Pentapetalae	whorled
Superasteridae	whorled
Asteridae	whorled
Lamiidae	whorled
Campanulidae	whorled
Superrosidae	whorled
Rosidae	whorled
Malvidae	whorled
Fabidae	whorled

ML ancestral state reconstruction using rayDISC (R:corHMM)
400_A. Gynoecium phyllotaxy (D2d), ARDeq model

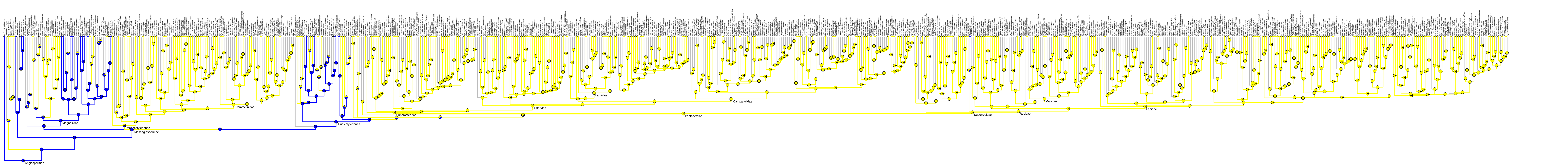

Node	ML state	Prob
Angiospermae	spiral	1
Mesangiospermae	spiral	0.9999
Magnoliidae	spiral	0.9997
Monocotyledonae	whorled	0.5215
Eudicotyledonae	spiral	0.9984
Commelinidae	whorled	1
Pentapetalae	whorled	0.9638
Superasteridae	whorled	0.984
Asteridae	whorled	1
Lamiidae	whorled	1
Campanulidae	whorled	1
Superrosidae	whorled	0.9668
Rosidae	whorled	0.967
Malvidae	whorled	1
Fabidae	whorled	[illegible]

Model	LogL	Npar	AIC	AICc	DeltaAICc	w	q01	q10
ARD	−39.15	2	82.31	82.32	1.39	0.3	0	[illegible]
ARDeq**	−38.46	2	80.92	80.93	0	0.61	0	0.0068
ER	−49.47	1	100.94	100.94	20.01	0	4e−04	4e−04
UNI01	−51.55	1	105.1	105.11	24.17	0	4e−04	
UNI10	−41.36	1	84.73	84.73	3.8	0.09		0.0079

MP ancestral state reconstruction using ancestral.pars
(R:phangorn)
403_A. Fusion of ovaries (binary) (D2c), 24 steps

- free (<5%)
- fused (>5%)

Node	MP state(s)
Angiospermae	free (<5%)
Mesangiospermae	free (<5%) / fused (>5%)
Magnoliidae	free (<5%) / fused (>5%)
Monocotyledonae	fused (>5%)
Eudicotyledonae	free (<5%) / fused (>5%)
Commelinidae	fused (>5%)
Pentapetalae	fused (>5%)
Superasteridae	fused (>5%)
Asteridae	fused (>5%)
Lamiidae	fused (>5%)
Campanulidae	fused (>5%)
Superrosidae	fused (>5%)
Rosidae	fused (>5%)
Malvidae	fused (>5%)
Fabidae	fused (>5%)

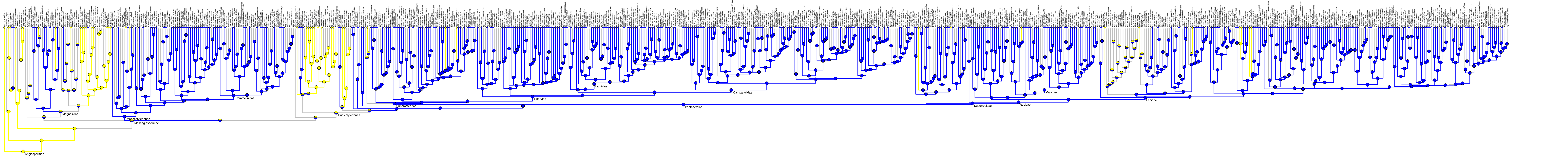

ML ancestral state reconstruction using rayDISC (R:corHMM)
403_A. Fusion of ovaries (binary) (D2c), ARDeq model

- free (<5%)
- fused (>5%)

Node	ML state	Prob
Angiospermae	free (<5%)	1
Mesangiospermae	free (<5%)	0.9998
Magnoliidae	free (<5%)	0.9993
Monocotyledonae	free (<5%)	0.9088
Eudicotyledonae	free (<5%)	0.9971
Commelinidae	fused (>5%)	1
Pentapetalae	fused (>5%)	0.9955
Superasteridae	fused (>5%)	0.9957
Asteridae	fused (>5%)	1
Lamiidae	fused (>5%)	1
Campanulidae	fused (>5%)	1
Superrosidae	fused (>5%)	0.9994
Rosidae	fused (>5%)	0.9997
Malvidae	fused (>5%)	1
Fabidae	fused (>5%)	0.9949

Model	LogL	Npar	AIC	AICc	DeltaAICc	w	q01	q10
ARD	−95.14	2	194.29	194.3	1.38	0.33	0.0045	5e−04
ARDeq**	−94.45	2	192.91	192.93	0	0.66	0.0045	5e−04
ER	−102	1	206	206.01	13.08	0	7e−04	7e−04
UNI01	−102.29	1	206.58	206.59	13.66	0	0.0102	
UNI10	−107.41	1	216.81	216.82	23.89	0		8e−04

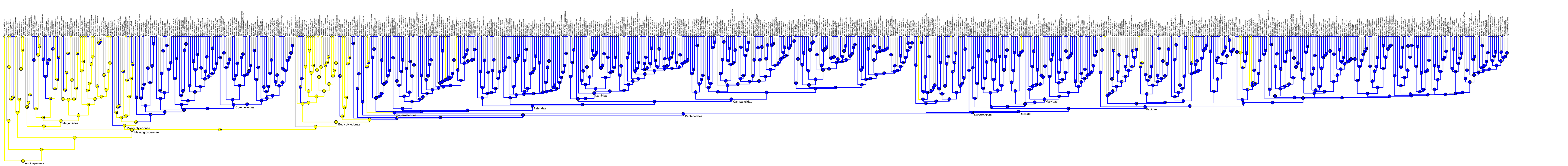

MP ancestral state reconstruction using ancestral.pars
(R:phangorn)
411_A. Number of ovules per functional carpel (3−state) (D2c), 121 steps

- one (1)
- two (2)
- three or more (>2)

Node	MP state(s)
Angiospermae	one (1)
Mesangiospermae	one (1)
Magnoliidae	one (1)
Monocotyledonae	one (1) / three or more (>2)
Eudicotyledonae	three or more (>2)
Commelinidae	one (1)
Pentapetalae	three or more (>2)
Superasteridae	three or more (>2)
Asteridae	one (1)
Lamiidae	two (2) / three or more (>2)
Campanulidae	one (1)
Superrosidae	two (2) / three or more (>2)
Rosidae	two (2)
Malvidae	two (2) / three or more (>2)
Fabidae	two (2)

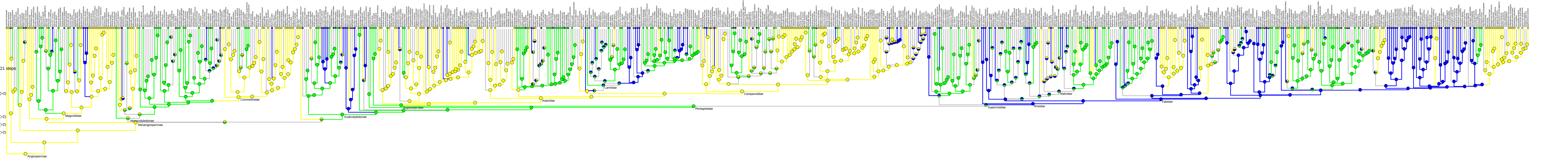

ML ancestral state reconstruction using rayDISC (R:corHMM)
411_A. Number of ovules per functional carpel (3−state) (D2c), ARDeq model

- one (1)
- two (2)
- three or more (>2)

Node	ML state	Prob
Angiospermae	three or more (>2)	0.9997
Mesangiospermae	three or more (>2)	1
Magnoliidae	three or more (>2)	0.9979
Monocotyledonae	three or more (>2)	0.9998
Eudicotyledonae	three or more (>2)	0.9999
Commelinidae	three or more (>2)	0.7501
Pentapetalae	three or more (>2)	1
Superasteridae	three or more (>2)	0.9997
Asteridae	three or more (>2)	0.9955
Lamiidae	three or more (>2)	0.9999
Campanulidae	three or more (>2)	0.9948
Superrosidae	three or more (>2)	1
Rosidae	three or more (>2)	0.9999
Malvidae	three or more (>2)	0.9996
Fabidae	three or more (>2)	0.9999

Model	LogL	Npar	AIC	AICc	DeltaAICc	w	[illegible]	
ARD	−387.49	6	786.99	[illegible]	1.85	0.28	0.0012	···
ARDeq**	−386.57	6	[illegible]	[illegible]	0	0.71	0.0012	···
ER	−399.78	1	801.56	801.56	16.32	0	0.0027	···
SYM	−399.11	3	804.21	804.24	19	0	0.0024	···
SYMeq	−398.53	3	803.05	803.08	17.84	0	0.0024	···
ORD	−394.33	4	796.66	796.71	11.47	0	0	···
ORDeq	−393.44	4	794.88	794.93	9.69	0.01	0	···
ORDSYM	−411.38	2	826.77	826.79	41.54	0	0.0052	···
ORDSYMeq	−411.17	2	826.33	826.35	41.1	0	0.0052	···
ORDER	−412.36	1	826.73	826.73	41.49	0	0.006	···

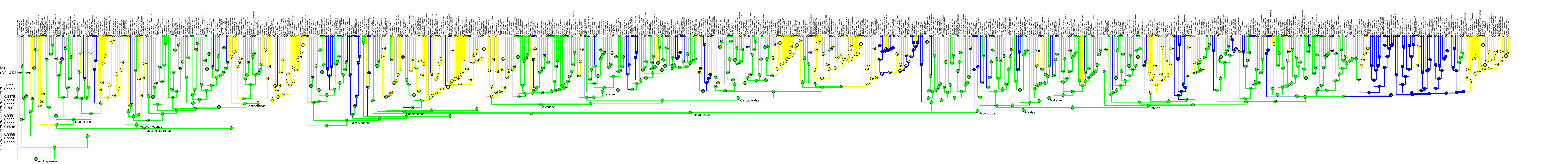

Supplement: Supplementary Data 19 [file ncomms16047-s20.pdf]
