# Supplementary material for: The ancestral flower of angiosperms and its early diversification
Source: Nat Commun. 2017 Aug 1;8:16047. doi: 10.1038/ncomms16047 (PMC5543309; doi:10.1038/ncomms16047)
Supplement: Supplementary Data 20 [file ncomms16047-s21.pdf]

MP ancestral state reconstruction using ancestral.pars
(R:phangorn)
100_A. Functional sex of flowers (D2d), 98 steps

- bisexual (yellow)
- unisexual (blue)

Node	MP state(s)
Angiospermae	bisexual / unisexual
Mesangiospermae	bisexual / unisexual
Magnoliidae	bisexual
Monocotyledonae	bisexual
Eudicotyledonae	bisexual
Commelinidae	bisexual
Pentapetalae	bisexual
Superasteridae	bisexual
Asteridae	bisexual
Lamiidae	bisexual
Campanulidae	bisexual
Superrosidae	bisexual
Rosidae	bisexual
Malvidae	bisexual
Fabidae	bisexual

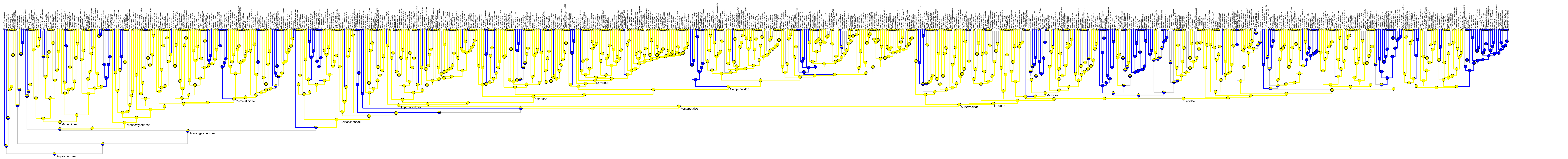

ML ancestral state reconstruction using rayDISC (R:corHMM)
100_A. Functional sex of flowers (D2d), ARDeq model

- bisexual
- unisexual

Node	ML state	Prob
Angiospermae	bisexual	1
Mesangiospermae	bisexual	1
Magnoliidae	bisexual	1
Monocotyledonae	bisexual	0.9999
Eudicotyledonae	bisexual	1
Commelinidae	bisexual	0.9996
Pentapetalae	bisexual	1
Superasteridae	bisexual	1
Asteridae	bisexual	1
Lamiidae	bisexual	1
Campanulidae	bisexual	0.9978
Superrosidae	bisexual	1
Rosidae	bisexual	1
Malvidae	bisexual	0.9997
Fabidae	bisexual	1

Model	LogL	Npar	AIC	AICc	DeltaAICc	w	q01	q10
ARD	−313.91	2	631.81	631.8[illegible]	1.33	0.24	0.0028	0.0011
ARDeq*	−313.24	2	630.48	630.5	0	0.46	0.0028	0.0011
ER	−315.34	1	632.69	632.69	2.2	0.15	0.0027	0.0027
UNI01	−315.41	1	632.82	632.82	2.33	0.14	0.0028	
UNI10	−364.24	1	730.49	730.49	100	0		0.0108

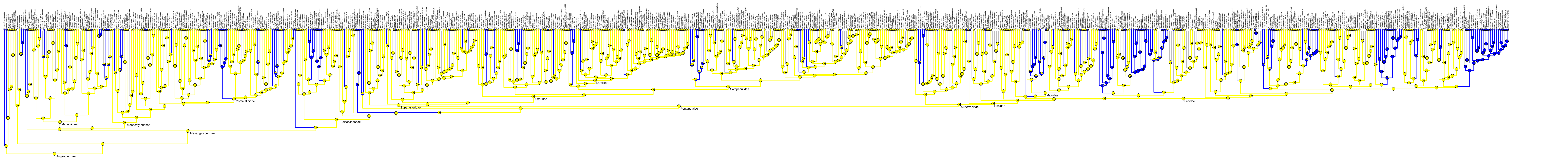

MP ancestral state reconstruction using ancestral.pars
(R:phangorn)
100_B. Structural sex of flowers (D2d), 57 steps

- bisexual
- unisexual

Node	MP state(s)
Angiospermae	bisexual
Mesangiospermae	bisexual
Magnoliidae	bisexual
Monocotyledonae	bisexual
Eudicotyledonae	bisexual
Commelinidae	bisexual
Pentapetalae	bisexual
Superasteridae	bisexual
Asteridae	bisexual
Lamiidae	bisexual
Campanulidae	bisexual
Superrosidae	bisexual
Rosidae	bisexual
Malvidae	bisexual
Fabidae	bisexual

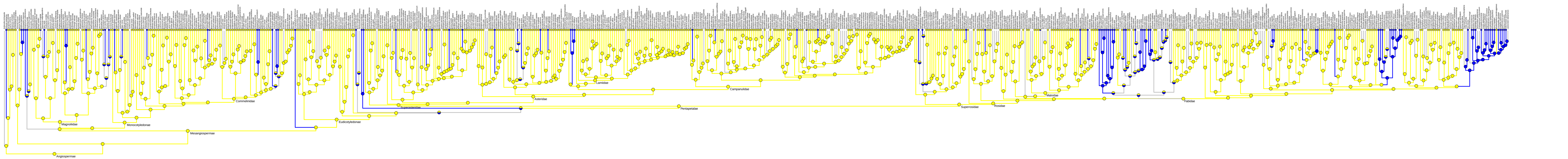

ML ancestral state reconstruction using rayDISC (R:corHMM)
100_B. Structural sex of flowers (D2d), ARDeq model

- bisexual
- unisexual

Node	ML state	Prob
Angiospermae	bisexual	0.9949
Mesangiospermae	bisexual	0.9974
Magnoliidae	bisexual	0.9995
Monocotyledonae	bisexual	0.9993
Eudicotyledonae	bisexual	0.999
Commelinidae	bisexual	0.9999
Pentapetalae	bisexual	0.9999
Superasteridae	bisexual	1
Asteridae	bisexual	1
Lamiidae	bisexual	0.9999
Campanulidae	bisexual	1
Superrosidae	bisexual	1
Rosidae	bisexual	1
Malvidae	bisexual	1
Fabidae	bisexual	1

Model	LogL	Npar	AIC	AICc	DeltaAICc	w	q01	q10
ARD	−207.3	2	418.6	418.61	0.79	0.38	0.0014	0.0042
ARDeq**	−206.91	2	417.81	417.83	0	0.56	0.0014	0.004
ER	−210.1	1	422.2	422.21	4.38	0.06	0.0015	0.0015
UNI01	−229.29	1	460.59	460.59	42.76	0	0.0016	
UNI10	−238.01	1	478.02	478.02	60.19	0		0.0149

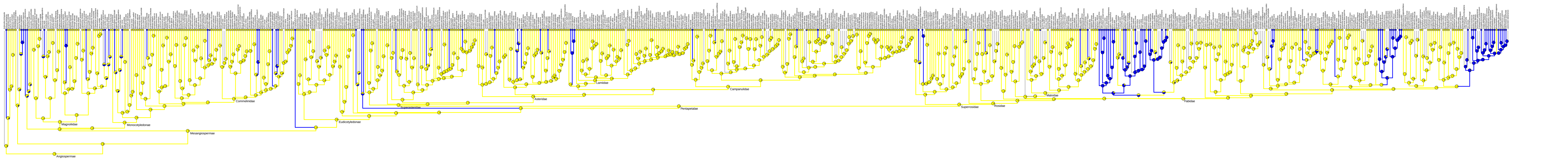

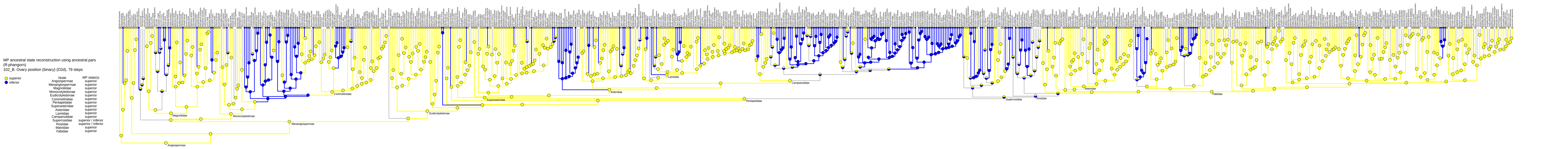
MP ancestral state reconstruction using ancestral.pars
(R:phangorn)
102_B. Ovary position (binary) (D2d), 79 steps
superior
inferior
Node
MP state(s)
Angiospermae superior
Mesangiospermae superior
Magnoliidae superior
Monocotyledonae superior
Eudicotyledonae superior
Commelinidae superior
Pentapetalae superior
Superasteridae superior
Asteridae superior
Lamiidae superior
Campanulidae superior
Superrosidae superior / inferior
Rosidae superior / inferior
Malvidae superior
Fabidae superior
Commelinidae
Magnoliidae
Monocotyledonae
Mesangiospermae
Angiospermae
Eudicotyledonae
Superasteridae
Asteridae
Lamiidae
Campanulidae
Pentapetalae
Superrosidae
Rosidae
Malvidae
Fabidae

ML ancestral state reconstruction using rayDISC (R:corHMM)
102_B. Ovary position (binary) (D2d), ARDeq model

- superior
- inferior

Node	ML state	Prob
Angiospermae	superior	1
Mesangiospermae	superior	0.9981
Magnoliidae	superior	0.9939
Monocotyledonae	superior	0.9811
Eudicotyledonae	superior	0.9995
Commelinidae	superior	0.6458
Pentapetalae	superior	0.9926
Superasteridae	superior	0.9968
Asteridae	superior	0.9166
Lamiidae	superior	0.9286
Campanulidae	superior	0.6699
Superrosidae	superior	0.9225
Rosidae	superior	0.9212
Malvidae	superior	1
Fabidae	superior	0.9996

Model	LogL	Npar	AIC	AICc	DeltaAICc	w	q01	q10
ARD	−270.17	2	544.35	544.36	1.25	0.32	0.0019	0.0038
ARDeq**	−269.55	2	543.09	543.11	0	0.6	0.0019	0.0037
ER	−272.53	1	547.07	547.07	3.96	0.08	0.0022	0.0022
UNI01	−287.14	1	576.28	576.28	33.17	0	0.0026	
UNI10	−296.3	1	594.61	594.62	51.51	0		0.0092

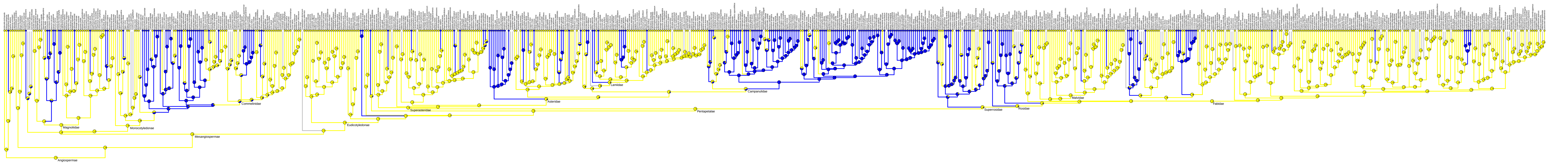

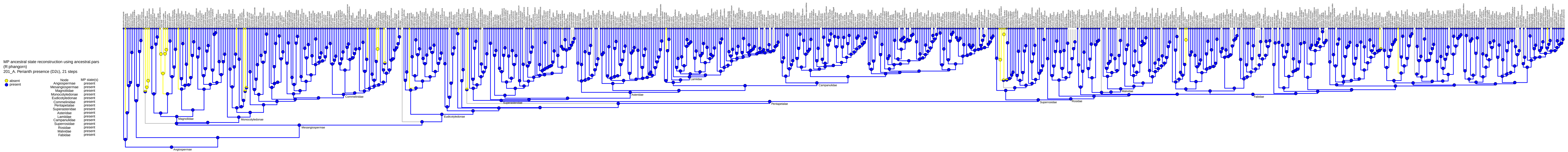
MP ancestral state reconstruction using ancestral.pars
(R:phangorn)
201_A. Perianth presence (D2c), 21 steps
absent
present
Node
MP state(s)
Angiospermae present
Mesangiospermae present
Magnoliidae present
Monocotyledonae present
Eudicotyledonae present
Commelinidae present
Pentapetalae present
Superasteridae present
Asteridae present
Lamiidae present
Campanulidae present
Superrosidae present
Rosidae present
Malvidae present
Fabidae present
Angiospermae
Mesangiospermae
Magnoliidae
Monocotyledonae
Commelinidae
Eudicotyledonae
Superasteridae
Pentapetalae
Asteridae
Lamiidae
Campanulidae
Superrosidae
Rosidae
Malvidae
Fabidae

ML ancestral state reconstruction using rayDISC (R:corHMM)
201_A. Perianth presence (D2c), UNI10 model

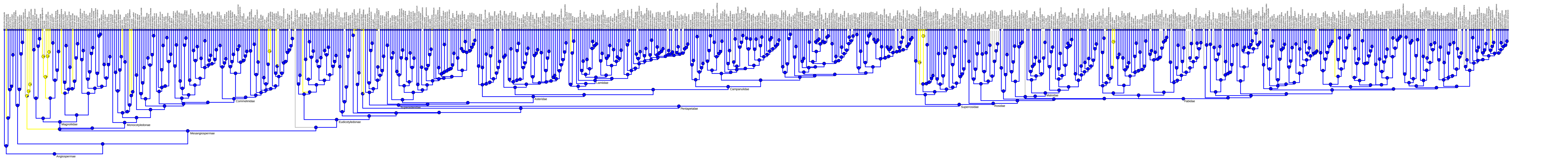

Node	ML state	Prob
Angiospermae	present	1
Mesangiospermae	present	1
Magnoliidae	present	1
Monocotyledonae	present	1
Eudicotyledonae	present	1
Commelinidae	present	1
Pentapetalae	present	1
Superasteridae	present	1
Asteridae	present	1
Lamiidae	present	1
Campanulidae	present	1
Superrosidae	present	1
Rosidae	present	1
Malvidae	present	1
Fabidae	present	1

Model	LogL	Npar	AIC	AICc	DeltaAICc	w	q01	q10
ARD	−95.7	2	195.39	195.41	2.01	0.12	0	5e−04
ARDeq	−95	2	194	194.02	0.62	0.25	0	5e−04
ER	−95.83	1	193.66	193.67	0.27	0.29	5e−04	5e−04
UNI01	−117.13	1	236.26	236.27	42.87	0	0.0186	
UNI10*	−95.7	1	193.39	193.4	0	0.34		5e−04

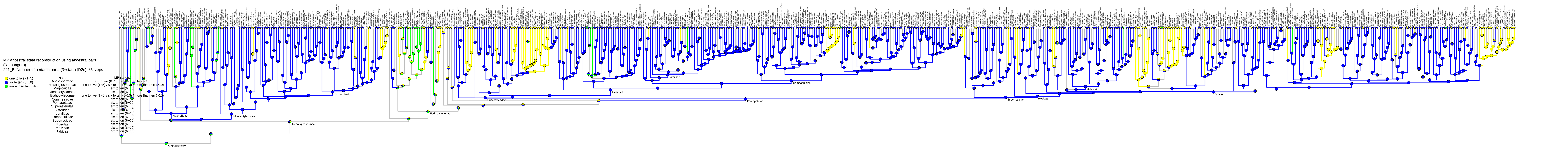
MP ancestral state reconstruction using ancestral.pars
(R:phangorn)
201_B. Number of perianth parts (3−state) (D2c), 86 steps
one to five (1−5)
six to ten (6−10)
more than ten (>10)
Node | MP state(s)
Angiospermae | six to ten (6−10) / more than ten (>10)
Mesangiospermae | one to five (1−5) / six to ten (6−10) / more than ten (>10)
Magnoliidae | six to ten (6−10)
Monocotyledonae | six to ten (6−10)
Eudicotyledonae | one to five (1−5) / six to ten (6−10) / more than ten (>10)
Commelinidae | six to ten (6−10)
Pentapetalae | six to ten (6−10)
Superasteridae | six to ten (6−10)
Asteridae | six to ten (6−10)
Lamiidae | six to ten (6−10)
Campanulidae | six to ten (6−10)
Superrosidae | six to ten (6−10)
Rosidae | six to ten (6−10)
Malvidae | six to ten (6−10)
Fabidae | six to ten (6−10)
Magnoliidae
Monocotyledonae
Commelinidae
Eudicotyledonae
Superasteridae
Asteridae
Lamiidae
Campanulidae
Pentapetalae
Superrosidae
Rosidae
Malvidae
Fabidae
Mesangiospermae
Angiospermae

ML ancestral state reconstruction using rayDISC (R:corHMM)
201_B. Number of perianth parts (3−state) (D2c), ARDeq model

- one to five (1−5)
- six to ten (6−10)
- more than ten (>10)

Node	ML state	Prob
Angiospermae	more than ten (>10)	1
Mesangiospermae	more than ten (>10)	0.9985
Magnoliidae	more than ten (>10)	0.9977
Monocotyledonae	six to ten (6−10)	0.5596
Eudicotyledonae	more than ten (>10)	0.9825
Commelinidae	six to ten (6−10)	0.9999
Pentapetalae	six to ten (6−10)	0.9949
Superasteridae	six to ten (6−10)	0.9976
Asteridae	six to ten (6−10)	0.9998
Lamiidae	six to ten (6−10)	0.9999
Campanulidae	six to ten (6−10)	0.9999
Superrosidae	six to ten (6−10)	0.9986
Rosidae	six to ten (6−10)	0.9988
Malvidae	six to ten (6−10)	0.9999
Fabidae	six to ten (6−10)	1

Model	LogL	Npar	AIC	AICc	DeltaAICc	w	q01	···
ARD	−326.29	6	664.57	664.68	2.18	0.24	0.004	···
ARDeq**	−325.2	6	662.4	662.5	0	0.72	0.004	···
ER	−351.28	1	704.56	704.56	42.06	0	0.0012	···
SYM	−340.38	3	686.75	686.78	24.28	0	0.0019	···
SYMeq	−339.55	3	685.09	685.12	22.62	0	0.0019	···
ORD	−331.52	4	671.05	671.1	8.6	0.01	0.0042	···
ORDeq	−330.43	4	668.87	668.92	6.42	0.03	0.0042	···
ORDSYM	−343.51	2	691.02	691.03	28.53	0	0.002	···
ORDSYMeq	−342.81	2	689.61	689.63	27.12	0	0.0019	···
ORDER	−352.85	1	707.7	707.71	45.2	0	0.0013	···

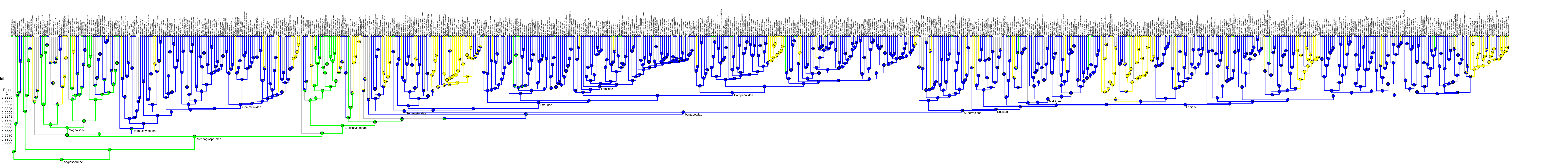

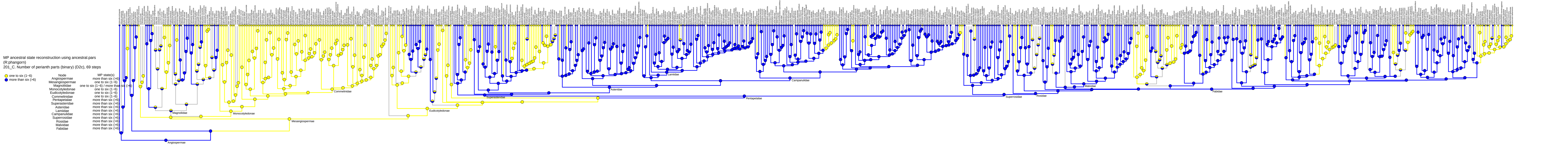

MP ancestral state reconstruction using ancestral.pars
(R:phangorn)
201_C. Number of perianth parts (binary) (D2c), 69 steps
one to six (1−6)
more than six (>6)
Node
MP state(s)
Angiospermae more than six (>6)
Mesangiospermae one to six (1−6)
Magnoliidae one to six (1−6) / more than six (>6)
Monocotyledonae one to six (1−6)
Eudicotyledonae one to six (1−6)
Commelinidae one to six (1−6)
Pentapetalae more than six (>6)
Superasteridae more than six (>6)
Asteridae more than six (>6)
Lamiidae more than six (>6)
Campanulidae more than six (>6)
Superrosidae more than six (>6)
Rosidae more than six (>6)
Malvidae more than six (>6)
Fabidae more than six (>6)
Angiospermae
Mesangiospermae
Magnoliidae
Monocotyledonae
Commelinidae
Eudicotyledonae
Superasteridae
Pentapetalae
Asteridae
Lamiidae
Campanulidae
Superrosidae
Rosidae
Malvidae
Fabidae

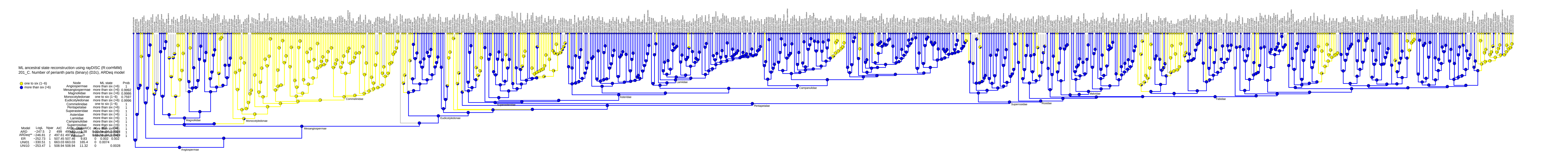
ML ancestral state reconstruction using rayDISC (R:corHMM)
201_C. Number of perianth parts (binary) (D2c), ARDeq model
one to six (1−6)
more than six (>6)
Node ML state Prob
Angiospermae more than six (>6) 1
Mesangiospermae more than six (>6) 0.9992
Magnoliidae more than six (>6) 0.9986
Monocotyledonae one to six (1−6) 0.7597
Eudicotyledonae more than six (>6) 0.9996
Commelinidae one to six (1−6) 1
Pentapetalae more than six (>6) 1
Superasteridae more than six (>6) 1
Asteridae more than six (>6) 1
Lamiidae more than six (>6) 1
Campanulidae more than six (>6) 1
Superrosidae more than six (>6) 1
Rosidae more than six (>6) 1
Malvidae more than six (>6) 1
Fabidae more than six (>6) 1
Model LogL Npar AIC AICc DeltaAICc w q01 q10
ARD −247.5 2 499 499.01 1.38
ARDeq** −246.81 2 497.61 497.63 0
ER −252.73 1 507.45 507.46 9.83 0 0.002 0.002
UNI01 −330.51 1 663.03 663.03 165.4 0 0.0074
UNI10 −253.47 1 508.94 508.94 11.32 0 0.0028
Angiospermae
Mesangiospermae
Magnoliidae
Monocotyledonae
Commelinidae
Eudicotyledonae
Pentapetalae
Asteridae
Lamiidae
Campanulidae
Superrosidae
Rosidae
Malvidae
Fabidae

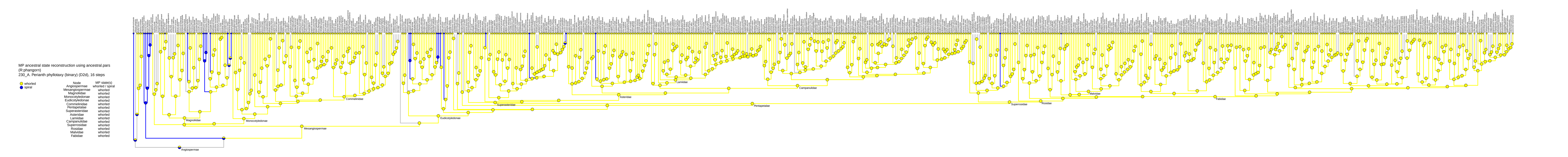
MP ancestral state reconstruction using ancestral.pars
(R:phangorn)
230_A. Perianth phyllotaxy (binary) (D2d), 16 steps
whorled
spiral
Node
MP state(s)
Angiospermae
whorled / spiral
Mesangiospermae
whorled
Magnoliidae
whorled
Monocotyledonae
whorled
Eudicotyledonae
whorled
Commelinidae
whorled
Pentapetalae
whorled
Superasteridae
whorled
Asteridae
whorled
Lamiidae
whorled
Campanulidae
whorled
Superrosidae
whorled
Rosidae
whorled
Malvidae
whorled
Fabidae
whorled

ML ancestral state reconstruction using rayDISC (R:corHMM)
230_A. Perianth phyllotaxy (binary) (D2d), ARD model

- whorled
- spiral

Node	ML state	Prob
Angiospermae	spiral	0.9994
Mesangiospermae	spiral	0.9994
Magnoliidae	spiral	0.9911
Monocotyledonae	whorled	0.5314
Eudicotyledonae	spiral	0.9977
Commelinidae	whorled	0.9999
Pentapetalae	whorled	0.9838
Superasteridae	whorled	0.9864
Asteridae	whorled	0.9997
Lamiidae	whorled	1
Campanulidae	whorled	1
Superrosidae	whorled	0.9947
Rosidae	whorled	0.9965
Malvidae	whorled	1
Fabidae	whorled	1

Model	LogL	Npar	AIC	AICc	DeltaAICc	w	q01	q10
ARD***	−70.11	2	144.22	144.23	0	0.99	2e−04	0.0087
ARDeq	−76.18	2	156.35	156.37	12.13	0	4e−04	1e−04
ER	−76.64	1	155.29	155.29	11.06	0	4e−04	4e−04
UNI01	−76.9	1	155.8	155.81	11.57	0	4e−04	
UNI10	−80.35	1	162.69	162.7	18.46	0		0.0148

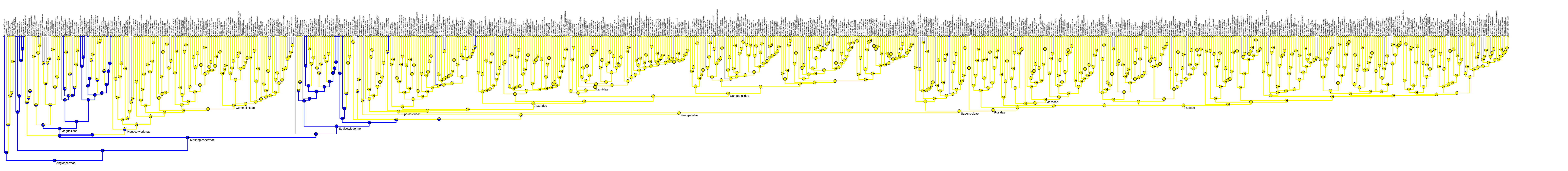

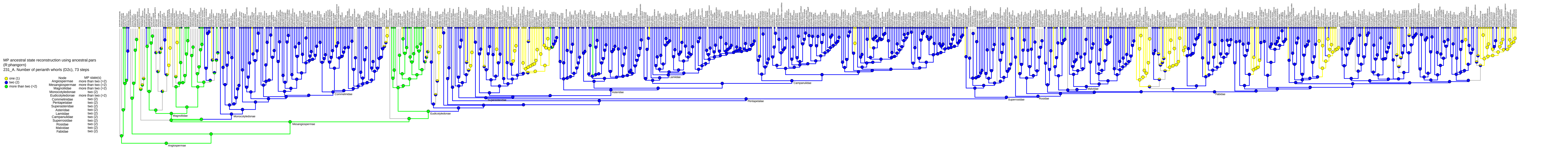
MP ancestral state reconstruction using ancestral.pars
(R:phangorn)
231_A. Number of perianth whorls (D2c), 73 steps
one (1)
two (2)
more than two (>2)
Node
MP state(s)
Angiospermae
more than two (>2)
Mesangiospermae
more than two (>2)
Magnoliidae
more than two (>2)
Monocotyledonae
two (2)
Eudicotyledonae
more than two (>2)
Commelinidae
two (2)
Pentapetalae
two (2)
Superasteridae
two (2)
Asteridae
two (2)
Lamiidae
two (2)
Campanulidae
two (2)
Superrosidae
two (2)
Rosidae
two (2)
Malvidae
two (2)
Fabidae
two (2)
Angiospermae
Mesangiospermae
Magnoliidae
Monocotyledonae
Eudicotyledonae
Commelinidae
Asteridae
Lamiidae
Campanulidae
Pentapetalae
Superrosidae
Rosidae
Malvidae
Fabidae

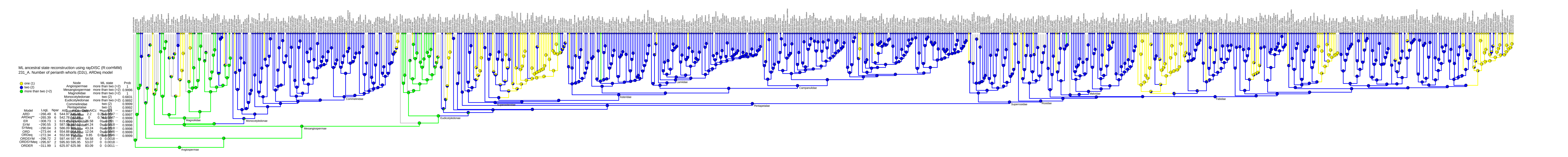

ML ancestral state reconstruction using rayDISC (R:corHMM)
231_A. Number of perianth whorls (D2c), ARDeq model
one (1)
two (2)
more than two (>2)
Node | ML state | Prob
Angiospermae | more than two (>2) | 1
Mesangiospermae | more than two (>2) | 0.9996
Magnoliidae | more than two (>2) | 1
Monocotyledonae | two (2) | 0.6831
Eudicotyledonae | more than two (>2) | 0.9892
Commelinidae | two (2) | 0.9999
Pentapetalae | two (2) | 0.9992
Superasteridae | two (2) | 0.9987
Asteridae | two (2) | 0.9997
Lamiidae | two (2) | 0.9999
Campanulidae | two (2) | 0.9999
Superrosidae | two (2) | 0.9998
Rosidae | two (2) | 0.9998
Malvidae | two (2) | 0.9999
Fabidae | two (2) | 0.9999
Model LogL Npar AIC AICc DeltaAICc w q01 ...
ARD −266.49 6 544.97 545.08 2.2 0.25 0.0047 ...
ARDeq** −265.39 6 542.78 542.88 0 0.74 0.0047 ...
ER −308.73 1 619.46 619.46 76.58 0 0.001 ...
SYM −290.55 3 587.09 587.12 44.24 0 0.0018 ...
SYMeq −290.04 3 586.09 586.12 43.24 0 0.0018 ...
ORD −273.44 4 554.88 554.93 12.04 0 0.0046 ...
ORDeq −272.34 4 552.68 552.73 9.85 0.01 0.0046 ...
ORDSYM −296.72 2 597.44 597.46 54.58 0 0.0018 ...
ORDSYMeq −295.97 2 595.93 595.95 53.07 0 0.0018 ...
ORDER −311.99 1 625.97 625.98 83.09 0 0.0011 ...
Angiospermae
Mesangiospermae
Magnoliidae
Monocotyledonae
Commelinidae
Eudicotyledonae
Pentapetalae
Asteridae
Lamiidae
Campanulidae
Superrosidae
Rosidae
Malvidae
Fabidae

MP ancestral state reconstruction using ancestral.pars
(R:phangorn)
232_A. Perianth merism (4−state) (D2c), 84 steps

- dimerous
- trimerous
- tetramerous
- pentamerous

Node	MP state(s)
Angiospermae	trimerous
Mesangiospermae	trimerous
Magnoliidae	trimerous
Monocotyledonae	trimerous
Eudicotyledonae	dimerous / trimerous
Commelinidae	trimerous
Pentapetalae	pentamerous
Superasteridae	pentamerous
Asteridae	pentamerous
Lamiidae	pentamerous
Campanulidae	pentamerous
Superrosidae	pentamerous
Rosidae	pentamerous
Malvidae	pentamerous
Fabidae	pentamerous

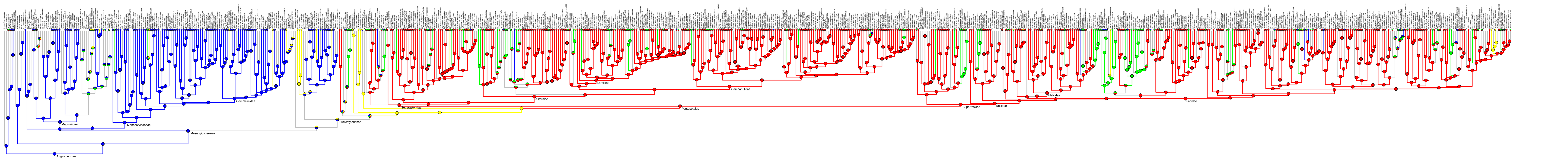

ML ancestral state reconstruction using rayDISC (R:corHMM)
232_A. Perianth merism (4−state) (D2c), SYMeq model

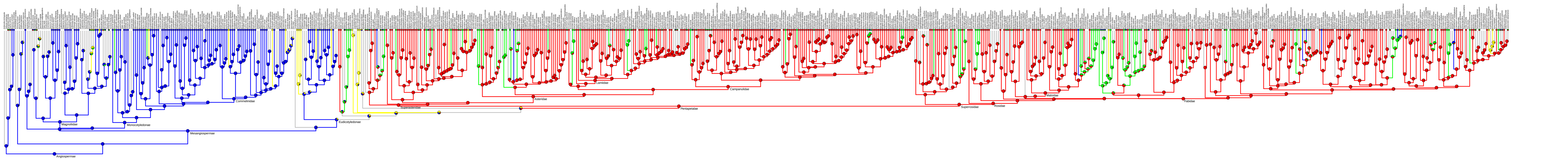

Node	ML state	Prob
Angiospermae	trimerous	1
Mesangiospermae	trimerous	0.9975
Magnoliidae	trimerous	1
Monocotyledonae	trimerous	1
Eudicotyledonae	trimerous	0.7223
Commelinidae	trimerous	1
Pentapetalae	pentamerous	0.9991
Superasteridae	pentamerous	0.9997
Asteridae	pentamerous	0.9997
Lamiidae	pentamerous	1
Campanulidae	pentamerous	1
Superrosidae	pentamerous	1
Rosidae	pentamerous	1
Malvidae	pentamerous	1
Fabidae	pentamerous	1

Model	LogL	Npar	AIC	AICc	DeltaAICc	w	q01	
ARD	−320.52	12	665.03	665.43	4.32	0.07	0.0014	···
ARDeq	−319.49	12	662.98	663.38	2.26	0.19	9e−04	···
ER	−361.07	1	724.13	724.14	63.02	0	8e−04	···
SYM	−325.81	6	663.63	663.73	2.61	0.16	0.0011	···
SYMeq**	−324.51	6	661.01	661.12	0	0.58	0.0011	···
ORD	−336.08	6	684.16	684.27	23.15	0	0	···
ORDeq	−335.41	6	682.82	682.93	21.81	0	0	···
ORDSYM	−345.1	3	696.2	696.23	35.11	0	0.0018	···
ORDSYMeq	−343.87	3	693.75	693.78	32.66	0	0.0018	···
ORDER	−347.12	1	696.24	696.25	35.13	0	0.0023	···

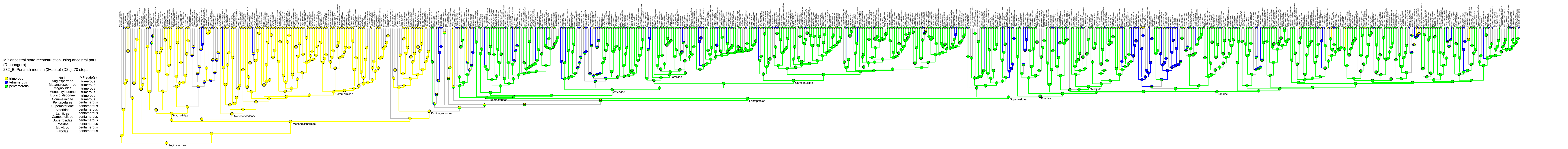
MP ancestral state reconstruction using ancestral.pars
(R:phangorn)
232_B. Perianth merism (3−state) (D2c), 70 steps
trimerous
tetramerous
pentamerous
Node
MP state(s)
Angiospermae trimerous
Mesangiospermae trimerous
Magnoliidae trimerous
Monocotyledonae trimerous
Eudicotyledonae trimerous
Commelinidae trimerous
Pentapetalae pentamerous
Superasteridae pentamerous
Asteridae pentamerous
Lamiidae pentamerous
Campanulidae pentamerous
Superrosidae pentamerous
Rosidae pentamerous
Malvidae pentamerous
Fabidae pentamerous

ML ancestral state reconstruction using rayDISC (R:corHMM)
232_B. Perianth merism (3−state) (D2c), ORD model

- trimerous
- tetramerous
- pentamerous

Node	ML state	Prob
Angiospermae	trimerous	0.8059
Mesangiospermae	trimerous	0.8776
Magnoliidae	trimerous	0.9852
Monocotyledonae	trimerous	0.9968
Eudicotyledonae	trimerous	0.6132
Commelinidae	trimerous	1
Pentapetalae	pentamerous	0.9857
Superasteridae	pentamerous	0.9903
Asteridae	pentamerous	0.9977
Lamiidae	pentamerous	0.9998
Campanulidae	pentamerous	0.9998
Superrosidae	pentamerous	0.9981
Rosidae	pentamerous	0.999
Malvidae	pentamerous	0.9998
Fabidae	pentamerous	1

Model	LogL	Npar	AIC	AICc	DeltaAICc	w	q01	...
ARD	−253.8	6	519.6	519.71	4.06	0.03	8e-04	···
ARDeq	−253.04	6	518.08	518.18	2.53	0.07	8e-04	···
ER	−280.95	1	563.89	563.9	48.25	0	0.001	···
SYM	−255.96	3	517.92	517.95	2.3	0.08	9e-04	···
SYMeq	−254.91	3	515.82	515.85	0.2	0.22	9e-04	···
ORD*	−253.8	4	515.6	515.65	0	0.25	8e-04	···
ORDeq	−254.1	4	516.2	516.25	0.6	0.18	3e-04	···
ORDSYM	−257.57	2	519.14	519.15	3.5	0.04	0.0014	···
ORDSYMeq	−256.57	2	517.14	517.15	1.5	0.12	0.0014	···
ORDER	−260.29	1	522.58	522.58	6.93	0.01	0.0022	···

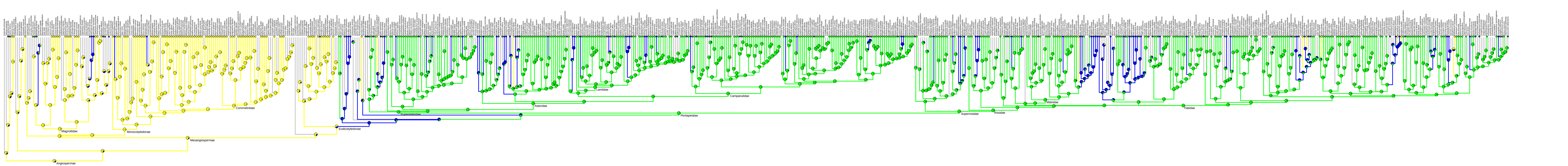

MP ancestral state reconstruction using ancestral.pars
(R:phangorn)
234_A. Perianth differentiation (binary) (D2d), 64 steps

- undifferentiated
- differentiated

Node	MP state(s)
Angiospermae	undifferentiated
Mesangiospermae	undifferentiated
Magnoliidae	undifferentiated
Monocotyledonae	undifferentiated
Eudicotyledonae	undifferentiated
Commelinidae	undifferentiated
Pentapetalae	differentiated
Superasteridae	differentiated
Asteridae	differentiated
Lamiidae	differentiated
Campanulidae	differentiated
Superrosidae	differentiated
Rosidae	differentiated
Malvidae	differentiated
Fabidae	differentiated

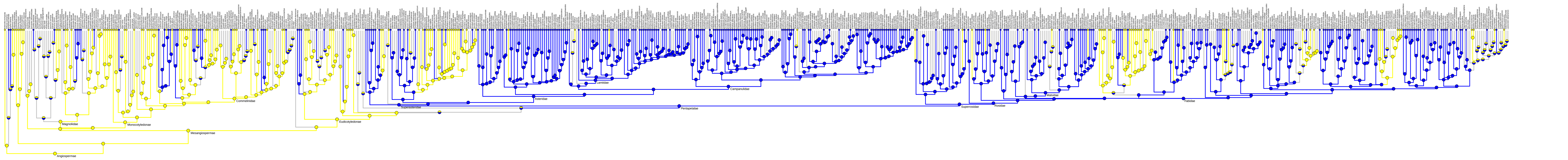

ML ancestral state reconstruction using rayDISC (R:corHMM)
234_A. Perianth differentiation (binary) (D2d), ARDeq model

- undifferentiated
- differentiated

Node	ML state	Prob
Angiospermae	undifferentiated	1
Mesangiospermae	undifferentiated	0.9994
Magnoliidae	undifferentiated	0.999
Monocotyledonae	undifferentiated	0.9998
Eudicotyledonae	undifferentiated	0.9541
Commelinidae	undifferentiated	0.9998
Pentapetalae	differentiated	0.9475
Superasteridae	differentiated	0.949
Asteridae	differentiated	0.9983
Lamiidae	differentiated	0.9998
Campanulidae	differentiated	1
Superrosidae	differentiated	0.9894
Rosidae	differentiated	0.9936
Malvidae	differentiated	0.9995
Fabidae	differentiated	0.9994

Model	LogL	Npar	AIC	AICc	DeltaAICc	W	q01	q10
ARD	−199.18	2	402.36	402.37	1.34	0.34	0.0041	0.0015
ARDeq**	−198.51	2	401.02	401.04	0	0.66	0.0041	0.0015
ER	−206.88	1	415.76	415.76	14.72	0	0.0024	0.0024
UNI01	−230.87	1	463.74	463.75	62.71	0	0.0078	
UNI10	−232.79	1	467.58	467.58	66.54	0		0.0032

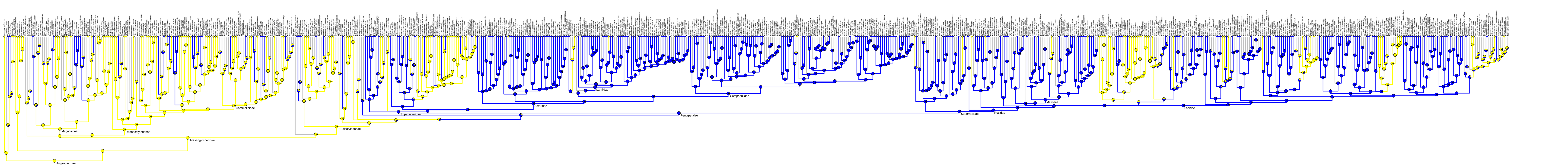

MP ancestral state reconstruction using ancestral.pars
(R:phangorn)
204_A. Fusion of perianth (D2c), 77 steps

- free (<5%)
- fused (>5%)

Node	MP state(s)
Angiospermae	free (<5%)
Mesangiospermae	free (<5%)
Magnoliidae	free (<5%)
Monocotyledonae	free (<5%)
Eudicotyledonae	free (<5%)
Commelinidae	free (<5%)
Pentapetalae	free (<5%)
Superasteridae	free (<5%)
Asteridae	free (<5%) / fused (>5%)
Lamiidae	free (<5%) / fused (>5%)
Campanulidae	fused (>5%)
Superrosidae	free (<5%)
Rosidae	free (<5%)
Malvidae	free (<5%)
Fabidae	free (<5%)

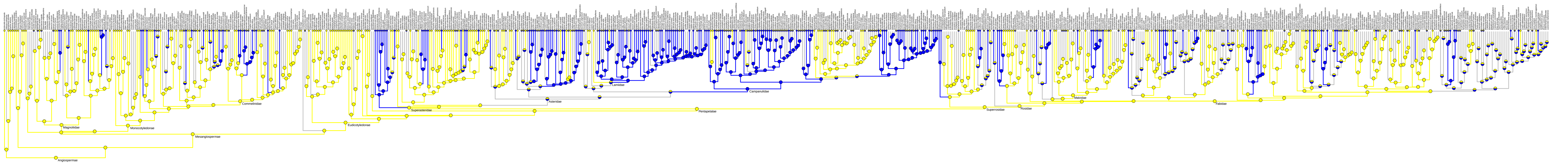

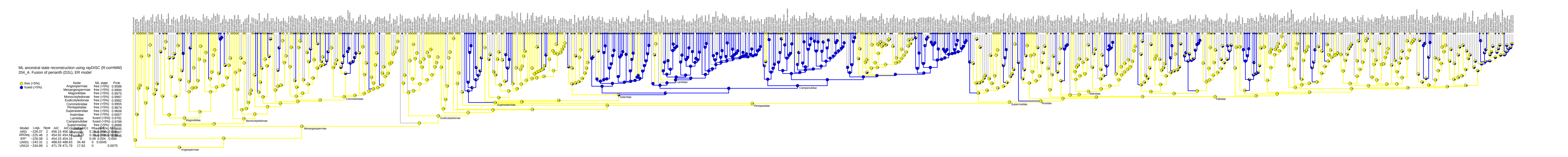
ML ancestral state reconstruction using rayDISC (R:corHMM)
204_A. Fusion of perianth (D2c), ER model
free (<5%)
fused (>5%)
Node | ML state | Prob
Angiospermae free (<5%) 0.9586
Mesangiospermae free (<5%) 0.9994
Magnoliidae free (<5%) 0.9975
Monocotyledonae free (<5%) 0.9987
Eudicotyledonae free (<5%) 0.9992
Commelinidae free (<5%) 0.9955
Pentapetalae free (<5%) 0.9874
Superasteridae free (<5%) 0.9606
Asteridae free (<5%) 0.6657
Lamiidae fused (>5%) 0.9781
Campanulidae fused (>5%) 0.9789
Superrosidae free (<5%) 0.9886
Rosidae free (<5%) 0.9895
Malvidae free (<5%) 0.9967
Fabidae free (<5%) 0.9996
Model LogL Npar AIC AICc DeltaAICc w q01 q10
ARD −226.07 2 456.15 456.16 2 0.18 0.004 0.004
ARDeq −225.46 2 454.92 454.93 0.77 0.33 0.004 0.0039
ER* −226.08 1 454.15 454.16 0 0.49 0.004 0.004
UNI01 −243.31 1 488.63 488.63 34.48 0 0.0045
UNI10 −234.89 1 471.78 471.79 17.63 0 0.0075
Angiospermae
Mesangiospermae
Magnoliidae
Monocotyledonae
Commelinidae
Eudicotyledonae
Superasteridae
Asteridae
Lamiidae
Campanulidae
Pentapetalae
Superrosidae
Rosidae
Malvidae
Fabidae

MP ancestral state reconstruction using ancestral.pars (R:phangorn)
207_A. Symmetry of perianth (binary) (D2d), 55 steps

- actinomorphic
- zygomorphic

Node	MP state(s)
Angiospermae	actinomorphic
Mesangiospermae	actinomorphic
Magnoliidae	actinomorphic
Monocotyledonae	actinomorphic
Eudicotyledonae	actinomorphic
Commelinidae	actinomorphic
Pentapetalae	actinomorphic
Superasteridae	actinomorphic
Asteridae	actinomorphic
Lamiidae	actinomorphic
Campanulidae	actinomorphic
Superrosidae	actinomorphic
Rosidae	actinomorphic
Malvidae	actinomorphic
Fabidae	actinomorphic

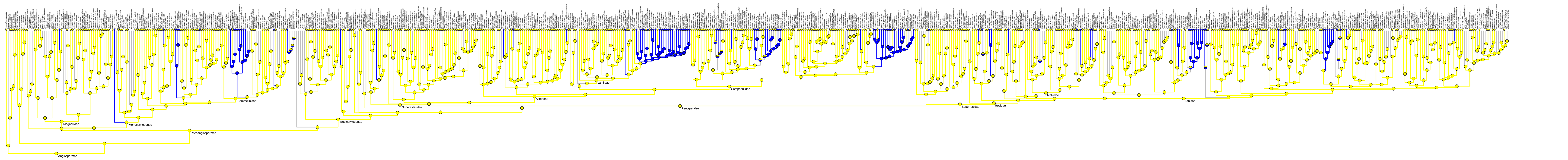

ML ancestral state reconstruction using rayDISC (R:corHMM)
207_A. Symmetry of perianth (binary) (D2d), ARDeq model

- actinomorphic
- zygomorphic

Node	ML state	Prob
Angiospermae	actinomorphic	0.9992
Mesangiospermae	actinomorphic	0.9995
Magnoliidae	actinomorphic	0.9991
Monocotyledonae	actinomorphic	0.9964
Eudicotyledonae	actinomorphic	0.9988
Commelinidae	actinomorphic	0.9948
Pentapetalae	actinomorphic	1
Superasteridae	actinomorphic	1
Asteridae	actinomorphic	0.9999
Lamiidae	actinomorphic	0.9976
Campanulidae	actinomorphic	0.9999
Superrosidae	actinomorphic	1
Rosidae	actinomorphic	0.9999
Malvidae	actinomorphic	0.9996
Fabidae	actinomorphic	1

Model	LogL	Npar	AIC	AICc	DeltaAICc	w	q01	q10
ARD	−209.71	2	423.41	423.43	1.04	0.37	0.0013	0.0064
ARDeq**	−209.18	2	422.37	422.38	0	0.63	0.0013	0.0063
ER	−219.05	1	440.1	440.11	17.73	0	0.0015	0.0015
UNI01	−263.15	1	528.3	528.3	105.92	0	0.0017	
UNI10	−217.79	1	437.58	437.59	15.2	0		0.0151

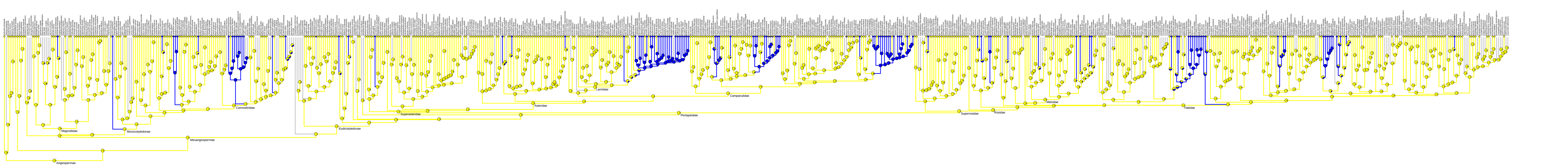

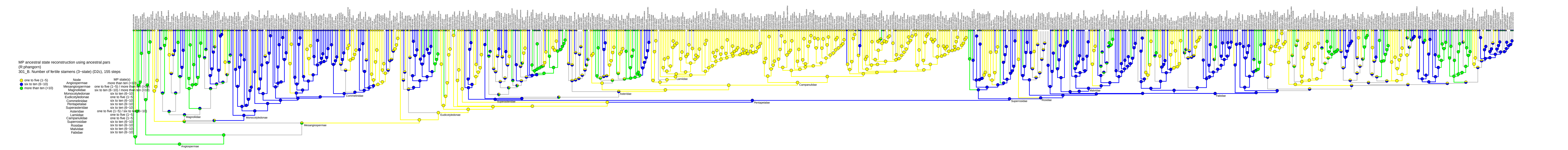

MP ancestral state reconstruction using ancestral.pars
(R:phangorn)
301_B. Number of fertile stamens (3−state) (D2c), 155 steps
one to five (1−5)
six to ten (6−10)
more than ten (>10)
Node | MP state(s)
Angiospermae | more than ten (>10)
Mesangiospermae | one to five (1−5) / more than ten (>10)
Magnoliidae | six to ten (6−10) / more than ten (>10)
Monocotyledonae | six to ten (6−10)
Eudicotyledonae | one to five (1−5)
Commelinidae | six to ten (6−10)
Pentapetalae | six to ten (6−10)
Superasteridae | six to ten (6−10)
Asteridae | one to five (1−5) / six to ten (6−10)
Lamiidae | one to five (1−5)
Campanulidae | one to five (1−5)
Superrosidae | six to ten (6−10)
Rosidae | six to ten (6−10)
Malvidae | six to ten (6−10)
Fabidae | six to ten (6−10)

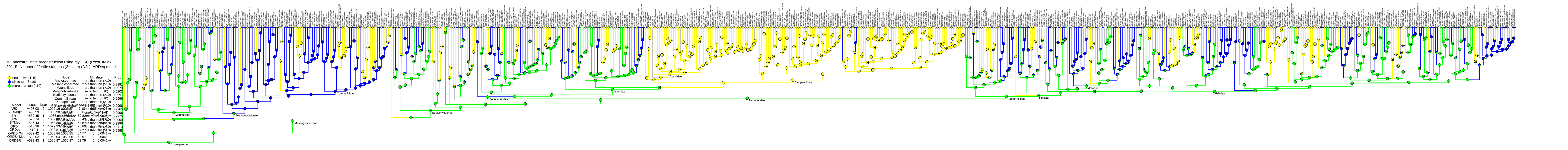

ML ancestral state reconstruction using rayDISC (R:corHMM)
301_B. Number of fertile stamens (3−state) (D2c), ARDeq model
one to five (1−5)
six to ten (6−10)
more than ten (>10)

Node	ML state	Prob
Angiospermae	more than ten (>10)	1
Mesangiospermae	more than ten (>10)	0.9991
Magnoliidae	more than ten (>10)	0.9979
Monocotyledonae	six to ten (6−10)	0.5202
Eudicotyledonae	more than ten (>10)	0.9991
Commelinidae	six to ten (6−10)	0.9996
Pentapetalae	more than ten (>10)	1
Superasteridae	more than ten (>10)	0.9998
Asteridae	more than ten (>10)	0.9965
Lamiidae	one to five (1−5)	0.9996
Campanulidae	one to five (1−5)	0.9975
Superrosidae	more than ten (>10)	0.9999
Rosidae	more than ten (>10)	0.9996
Malvidae	more than ten (>10)	0.9113
Fabidae	more than ten (>10)	0.9998

Model	LogL	Npar	AIC	AICc	DeltaAICc	w	q01
ARD	−497.08	6	1006.16	1006.27	2.18	0.25	4e−04 ···
ARDeq**	−495.99	6	1003.98	1004.08	0	0.75	4e−04 ···
ER	−532.45	1	1066.9	1066.9	62.82	0	0.0025 ···
SYM	−526.74	3	1059.48	1059.51	55.43	0	0.0034 ···
SYMeq	−526.43	3	1058.86	1058.89	54.81	0	0.0034 ···
ORD	−510.96	4	1029.92	1029.97	25.89	0	8e−04 ···
ORDeq	−510.4	4	1028.81	1028.86	24.77	0	8e−04 ···
ORDSYM	−532.42	2	1068.84	1068.85	64.77	0	0.0041 ···
ORDSYMeq	−532.02	2	1068.04	1068.06	63.97	0	0.0041 ···
ORDER	−532.43	1	1066.87	1066.87	62.79	0	0.0041 ···

Angiospermae
Mesangiospermae
Magnoliidae
Monocotyledonae
Commelinidae
Eudicotyledonae
Pentapetalae
Superasteridae
Asteridae
Lamiidae
Campanulidae
Superrosidae
Rosidae
Malvidae
Fabidae

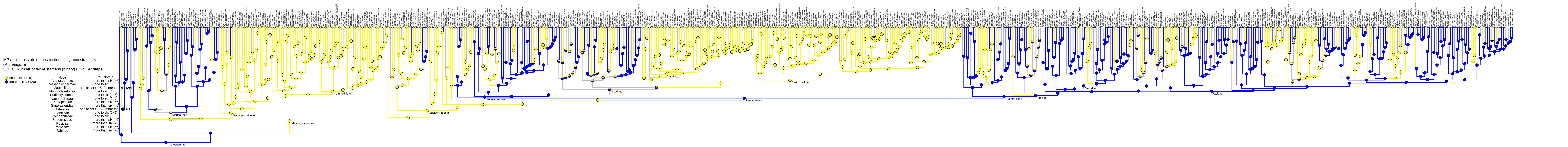
MP ancestral state reconstruction using ancestral.pars
(R:phangorn)
301_C. Number of fertile stamens (binary) (D2c), 92 steps
one to six (1−6)
more than six (>6)
Node
MP state(s)
Angiospermae more than six (>6)
Mesangiospermae one to six (1−6)
Magnoliidae one to six (1−6) / more than six (>6)
Monocotyledonae one to six (1−6)
Eudicotyledonae one to six (1−6)
Commelinidae one to six (1−6)
Pentapetalae more than six (>6)
Superasteridae more than six (>6)
Asteridae one to six (1−6) / more than six (>6)
Lamiidae one to six (1−6)
Campanulidae one to six (1−6)
Superrosidae more than six (>6)
Rosidae more than six (>6)
Malvidae more than six (>6)
Fabidae more than six (>6)

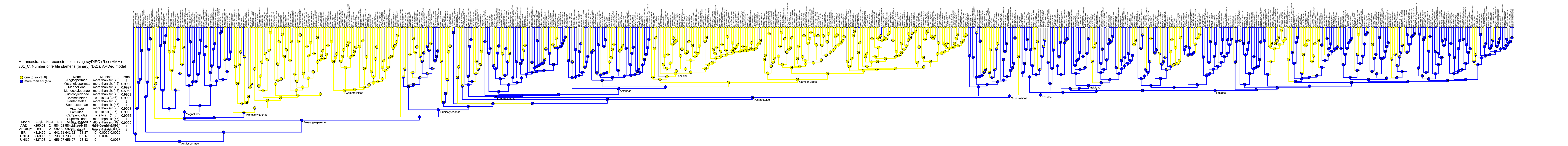
ML ancestral state reconstruction using rayDISC (R:corHMM)
301_C. Number of fertile stamens (binary) (D2c), ARDeq model
one to six (1−6)
more than six (>6)
Node | ML state | Prob
Angiospermae | more than six (>6) | 1
Mesangiospermae | more than six (>6) | 0.9988
Magnoliidae | more than six (>6) | 0.9997
Monocotyledonae | more than six (>6) | 0.5053
Eudicotyledonae | more than six (>6) | 0.9969
Commelinidae | one to six (1−6) | 0.9999
Pentapetalae | more than six (>6) | 1
Superasteridae | more than six (>6) | 1
Asteridae | more than six (>6) | 0.9998
Lamiidae | one to six (1−6) | 0.9992
Campanulidae | one to six (1−6) | 0.9955
Superrosidae | more than six (>6) | 1
Rosidae | more than six (>6) | 0.9999
Malvidae | more than six (>6) | 1
Fabidae | more than six (>6) | 1
Model | LogL | Npar | AIC | AICc | DeltaAICc | w | q01 | q10
ARD | −290.01 | 2 | 584.02 | 584.03 | 1.38 | 0.33 | 6e−04 | 0.0054
ARDeq** | −289.32 | 2 | 582.63 | 582.65 | 0 | 0.67 | 6e−04 | 0.0054
ER | −319.76 | 1 | 641.51 | 641.52 | 58.87 | 0 | 0.0029 | 0.0029
UNI01 | −368.16 | 1 | 738.31 | 738.32 | 155.67 | 0 | 0.0043
UNI10 | −327.03 | 1 | 656.07 | 656.07 | 73.43 | 0 | | 0.0067

MP ancestral state reconstruction using ancestral.pars
(R:phangorn)
330_A. Androecium structural phyllotaxy (binary) (D2d), 12 steps

- whorled (yellow)
- spiral (blue)

Node	MP state(s)
Angiospermae	whorled / spiral
Mesangiospermae	whorled
Magnoliidae	whorled
Monocotyledonae	whorled
Eudicotyledonae	whorled
Commelinidae	whorled
Pentapetalae	whorled
Superasteridae	whorled
Asteridae	whorled
Lamiidae	whorled
Campanulidae	whorled
Superrosidae	whorled
Rosidae	whorled
Malvidae	whorled
Fabidae	whorled

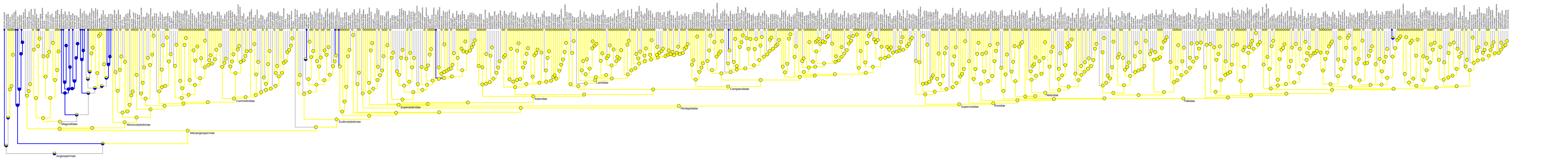

ML ancestral state reconstruction using rayDISC (R:corHMM)
330_A. Androecium structural phyllotaxy (binary) (D2d), ARD model

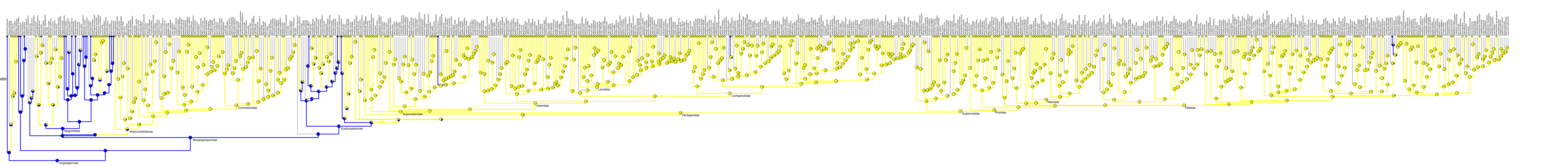

Node	ML state	Prob
Angiospermae	spiral	0.9993
Mesangiospermae	spiral	0.9809
Magnoliidae	spiral	0.9793
Monocotyledonae	whorled	0.5978
Eudicotyledonae	spiral	0.8702
Commelinidae	whorled	0.9999
Pentapetalae	whorled	0.999
Superasteridae	whorled	0.9997
Asteridae	whorled	1
Lamiidae	whorled	1
Campanulidae	whorled	1
Superrosidae	whorled	0.9998
Rosidae	whorled	0.9999
Malvidae	whorled	1
Fabidae	whorled	1

Model	LogL	Npar	AIC	AICc	DeltaAICc	w	q01	q10
ARD**	−53.79	2	111.58	111.59	0	0.9	2e−04	0.0065
ARDeq	−57.31	2	118.63	118.64	7.05	0.03	4e−04	0
ER	−58.1	1	118.19	118.2	6.6	0.03	4e−04	4e−04
UNI01	−58.01	1	118.01	118.02	6.43	0.04	4e−04	
UNI10	−80.52	1	163.03	163.04	51.45	0		0.0168

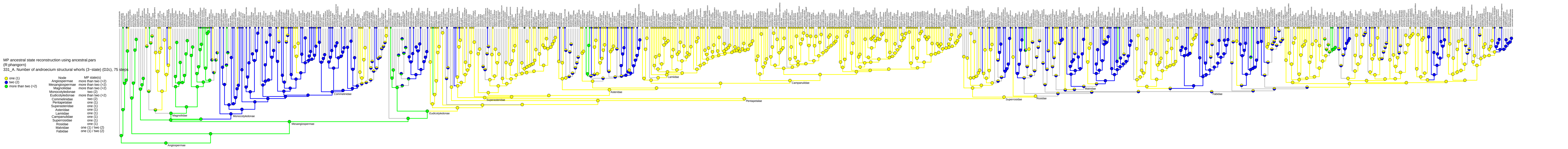

MP ancestral state reconstruction using ancestral.pars
(R:phangorn)
331_A. Number of androecium structural whorls (3−state) (D2c), 75 steps
one (1)
two (2)
more than two (>2)
Node | MP state(s)
Angiospermae | more than two (>2)
Mesangiospermae | more than two (>2)
Magnoliidae | more than two (>2)
Monocotyledonae | two (2)
Eudicotyledonae | more than two (>2)
Commelinidae | two (2)
Pentapetalae | one (1)
Superasteridae | one (1)
Asteridae | one (1)
Lamiidae | one (1)
Campanulidae | one (1)
Superrosidae | one (1)
Rosidae | one (1)
Malvidae | one (1) / two (2)
Fabidae | one (1) / two (2)
Angiospermae
Mesangiospermae
Magnoliidae
Monocotyledonae
Commelinidae
Eudicotyledonae
Superasteridae
Pentapetalae
Asteridae
Lamiidae
Campanulidae
Superrosidae
Rosidae
Malvidae
Fabidae

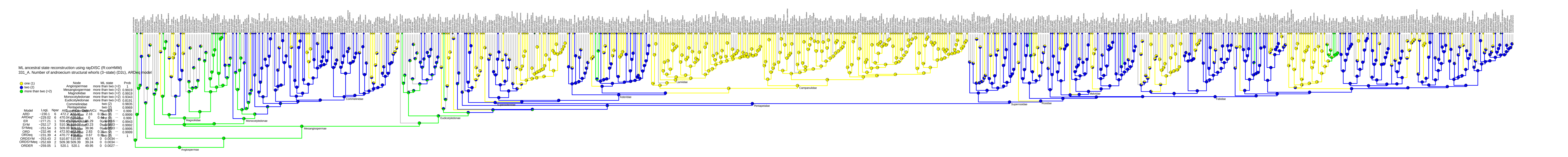
ML ancestral state reconstruction using rayDISC (R:corHMM)
331_A. Number of androecium structural whorls (3−state) (D2c), ARDeq model
one (1)
two (2)
more than two (>2)
Node | ML state | Prob
Angiospermae | more than two (>2) | 1
Mesangiospermae | more than two (>2) | 0.9919
Magnoliidae | more than two (>2) | 0.9919
Monocotyledonae | more than two (>2) | 0.9343
Eudicotyledonae | more than two (>2) | 0.8191
Commelinidae | two (2) | 0.9835
Pentapetalae | two (2) | 0.9969
Superasteridae | two (2) | 0.999
Asteridae | two (2) | 0.9999
Lamiidae | one (1) | 0.999
Campanulidae | one (1) | 0.9943
Superrosidae | two (2) | 0.9992
Rosidae | two (2) | 0.9995
Malvidae | two (2) | 0.9999
Fabidae | two (2) | 1
Model | LogL | Npar | AIC | AICc | DeltaAICc | w | q01 | ···
ARD | −230.1 | 6 | 472.2 | 472.31 | 2.16 | 0.15 | 0 | ···
ARDeq* | −229.02 | 6 | 470.04 | 470.15 | 0 | 0.44 | 0 | ···
ER | −277.21 | 1 | 556.43 | 556.43 | 86.29 | 0 | 0.0016 | ···
SYM | −252.17 | 3 | 510.34 | 510.37 | 40.23 | 0 | 0.0033 | ···
SYMeq | −251.54 | 3 | 509.08 | 509.11 | 38.96 | 0 | 0.0033 | ···
ORD | −232.46 | 4 | 472.93 | 472.98 | 2.83 | 0.11 | 0 | ···
ORDeq | −231.39 | 4 | 470.77 | 470.82 | 0.67 | 0.31 | 0 | ···
ORDSYM | −253.43 | 2 | 510.87 | 510.88 | 40.74 | 0 | 0.0034 | ···
ORDSYMeq | −252.69 | 2 | 509.38 | 509.39 | 39.24 | 0 | 0.0034 | ···
ORDER | −259.05 | 1 | 520.1 | 520.1 | 49.95 | 0 | 0.0027 | ···
Magnoliidae
Monocotyledonae
Mesangiospermae
Eudicotyledonae
Commelinidae
Asteridae
Lamiidae
Campanulidae
Pentapetalae
Superrosidae
Rosidae
Malvidae
Fabidae
Angiospermae

MP ancestral state reconstruction using ancestral.pars
(R:phangorn)
332_A. Androecium structural merism (4−state) (D2c), 58 steps

- dimerous
- trimerous
- tetramerous
- pentamerous

Node	MP state(s)
Angiospermae	trimerous / tetramerous / pentamerous
Mesangiospermae	trimerous / tetramerous / pentamerous
Magnoliidae	trimerous
Monocotyledonae	trimerous
Eudicotyledonae	trimerous / tetramerous / pentamerous
Commelinidae	trimerous
Pentapetalae	pentamerous
Superasteridae	pentamerous
Asteridae	pentamerous
Lamiidae	pentamerous
Campanulidae	pentamerous
Superrosidae	pentamerous
Rosidae	pentamerous
Malvidae	pentamerous
Fabidae	pentamerous

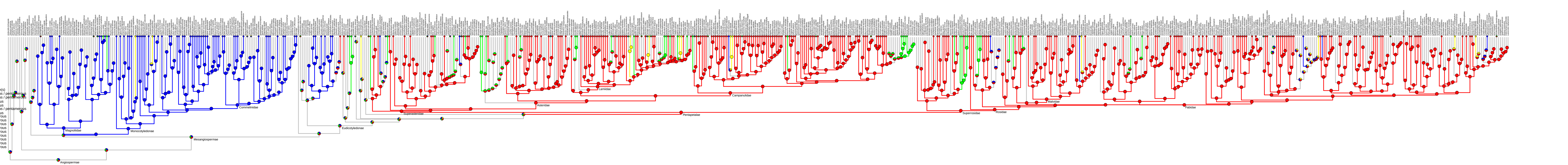

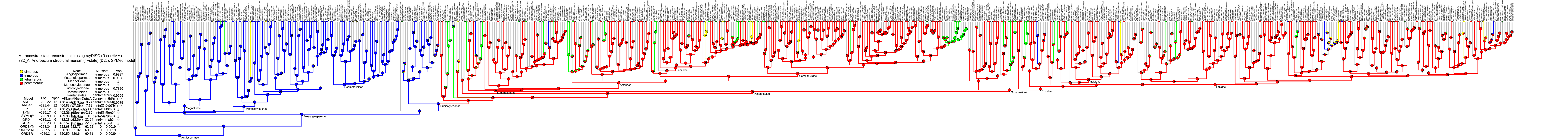
ML ancestral state reconstruction using rayDISC (R:corHMM)
332_A. Androecium structural merism (4−state) (D2c), SYMeq model
dimerous
trimerous
tetramerous
pentamerous
Node ML state Prob
Angiospermae trimerous 0.9997
Mesangiospermae trimerous 0.9958
Magnoliidae trimerous 1
Monocotyledonae trimerous 1
Eudicotyledonae trimerous 0.7826
Commelinidae trimerous 1
Pentapetalae pentamerous 0.9999
Superasteridae pentamerous 0.9999
Asteridae pentamerous 0.9985
Lamiidae pentamerous 0.9999
Campanulidae pentamerous 1
Superrosidae pentamerous 1
Rosidae pentamerous 1
Malvidae pentamerous 1
Fabidae pentamerous 1
Model LogL Npar AIC AICc DeltaAICc w q01
ARD −222.22 12 468.43 468.83 8.74 0.01 0.0087 ···
ARDeq −221.44 12 466.88 467.28 7.19 0.02 0.0074 ···
ER −238.12 1 478.25 478.25 18.16 0 8e−04 ···
SYM −225.17 6 462.33 462.44 2.35 0.23 5e−04 ···
SYMeq** −223.99 6 459.98 460.09 0 0.74 5e−04 ···
ORD −235.11 6 482.23 482.34 22.24 0 100 ···
ORDeq −235.28 6 482.57 482.67 22.58 0 100 ···
ORDSYM −258.34 3 522.68 522.71 62.62 0 0.0019 ···
ORDSYMeq −257.5 3 520.99 521.02 60.93 0 0.0019 ···
ORDER −259.3 1 520.59 520.6 60.51 0 0.0029 ···
Angiospermae
Mesangiospermae
Magnoliidae
Monocotyledonae
Commelinidae
Eudicotyledonae
Pentapetalae
Asteridae
Lamiidae
Campanulidae
Superrosidae
Rosidae
Malvidae
Fabidae

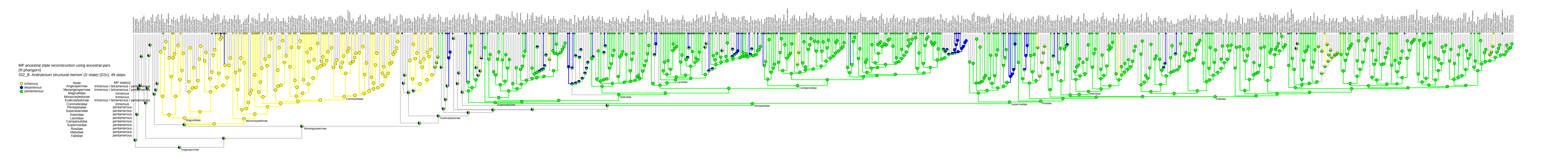
MP ancestral state reconstruction using ancestral.pars
(R:phangorn)
332_B. Androecium structural merism (3−state) (D2c), 49 steps
trimerous
tetramerous
pentamerous
Node
MP state(s)
Angiospermae trimerous / tetramerous / pentamerous
Mesangiospermae trimerous / tetramerous / pentamerous
Magnoliidae trimerous
Monocotyledonae trimerous
Eudicotyledonae trimerous / tetramerous / pentamerous
Commelinidae trimerous
Pentapetalae pentamerous
Superasteridae pentamerous
Asteridae pentamerous
Lamiidae pentamerous
Campanulidae pentamerous
Superrosidae pentamerous
Rosidae pentamerous
Malvidae pentamerous
Fabidae pentamerous
Magnoliidae
Monocotyledonae
Commelinidae
Mesangiospermae
Angiospermae
Eudicotyledonae
Superasteridae
Pentapetalae
Asteridae
Lamiidae
Campanulidae
Superrosidae
Rosidae
Malvidae
Fabidae

ML ancestral state reconstruction using rayDISC (R:corHMM)
332_B. Androecium structural merism (3−state) (D2c), ARDeq model

- trimerous
- tetramerous
- pentamerous

Node	ML state	Prob
Angiospermae	trimerous	1
Mesangiospermae	trimerous	0.9998
Magnoliidae	trimerous	1
Monocotyledonae	trimerous	1
Eudicotyledonae	trimerous	0.8221
Commelinidae	trimerous	1
Pentapetalae	tetramerous	0.9999
Superasteridae	tetramerous	1
Asteridae	tetramerous	0.9998
Lamiidae	tetramerous	0.9639
Campanulidae	tetramerous	0.8347
Superrosidae	tetramerous	0.9989
Rosidae	tetramerous	0.9919
Malvidae	tetramerous	0.5173
Fabidae	tetramerous	0.9424

Model	LogL	Npar	AIC	AICc	DeltaAICc	w	q01	
ARD	−171.93	6	355.86	355.97	2.01	0.23	8e−04	···
ARDeq**	−170.93	6	353.85	353.96	0	0.63	8e−04	···
ER	−186.8	1	375.59	375.6	21.64	0	0.0011	···
SYM	−176.94	3	359.88	359.91	5.95	0.03	4e−04	···
SYMeq	−176.02	3	358.05	358.08	4.12	0.08	4e−04	···
ORD	−177.01	4	362.02	362.07	8.11	0.01	6e−04	···
ORDeq	−176.64	4	361.27	361.32	7.36	0.02	4e−04	···
ORDSYM	−185.02	2	374.04	374.06	20.1	0	0.0018	···
ORDSYMeq	−184.24	2	372.48	372.5	18.54	0	0.0018	···
ORDER	−186.04	1	374.08	374.09	20.13	0	0.0026	···

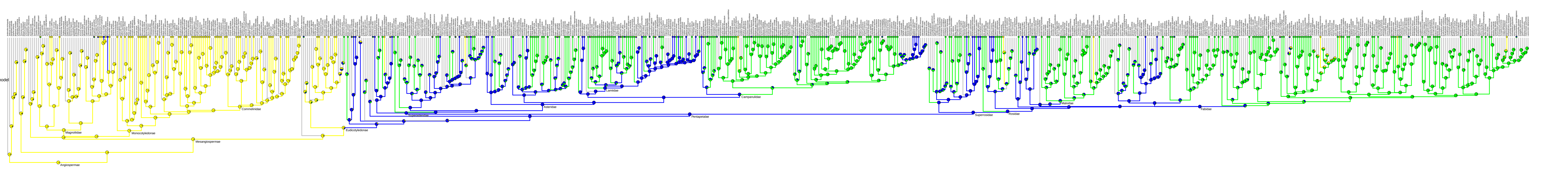

MP ancestral state reconstruction using ancestral.pars
(R:phangorn)
305_A. Filament (binary) (D2d), 55 steps

- laminar (wide)
- typical (narrow)

Node	MP state(s)
Angiospermae	laminar (wide)
Mesangiospermae	laminar (wide)
Magnoliidae	laminar (wide)
Monocotyledonae	typical (narrow)
Eudicotyledonae	typical (narrow)
Commelinidae	laminar (wide) / typical (narrow)
Pentapetalae	typical (narrow)
Superasteridae	typical (narrow)
Asteridae	typical (narrow)
Lamiidae	typical (narrow)
Campanulidae	typical (narrow)
Superrosidae	typical (narrow)
Rosidae	typical (narrow)
Malvidae	typical (narrow)
Fabidae	typical (narrow)

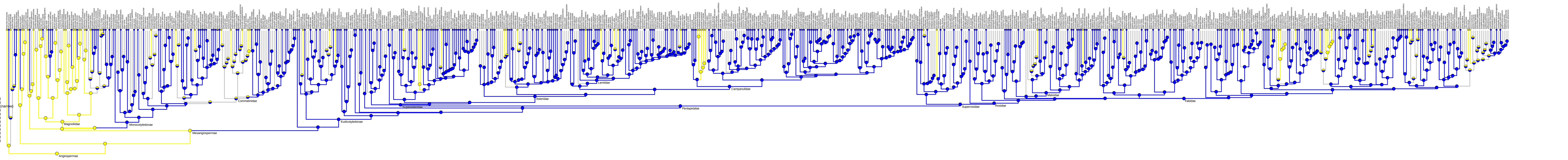

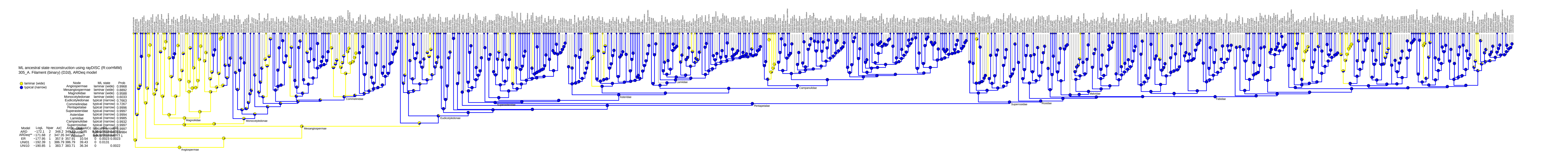

ML ancestral state reconstruction using rayDISC (R:corHMM)
305_A. Filament (binary) (D2d), ARDeq model
laminar (wide)
typical (narrow)
Node | ML state | Prob
Angiospermae laminar (wide) 0.9968
Mesangiospermae laminar (wide) 0.8892
Magnoliidae laminar (wide) 0.9588
Monocotyledonae laminar (wide) 0.6033
Eudicotyledonae typical (narrow) 0.7663
Commelinidae typical (narrow) 0.7267
Pentapetalae typical (narrow) 0.9996
Superasteridae typical (narrow) 0.9997
Asteridae typical (narrow) 0.9994
Lamiidae typical (narrow) 0.9985
Campanulidae typical (narrow) 0.9932
Superrosidae typical (narrow) 0.9997
Rosidae typical (narrow) 0.9997
Malvidae typical (narrow) 0.9984
Fabidae typical (narrow) 1
Model LogL Npar AIC AICc DeltaAICc w q01 q10
ARD −172.1 2 348.2 348.22 0.85 0.39 0.0072 0.0021
ARDeq** −171.68 2 347.35 347.37 0 0.6 0.0073 0.0021
ER −177.95 1 357.9 357.91 10.54 0 0.0023 0.0023
UNI01 −192.39 1 386.79 386.79 39.43 0 0.0131
UNI10 −190.85 1 383.7 383.71 36.34 0 0.0022
Angiospermae
Mesangiospermae
Magnoliidae
Monocotyledonae
Commelinidae
Eudicotyledonae
Asteridae
Lamiidae
Campanulidae
Pentapetalae
Superrosidae
Rosidae
Malvidae
Fabidae

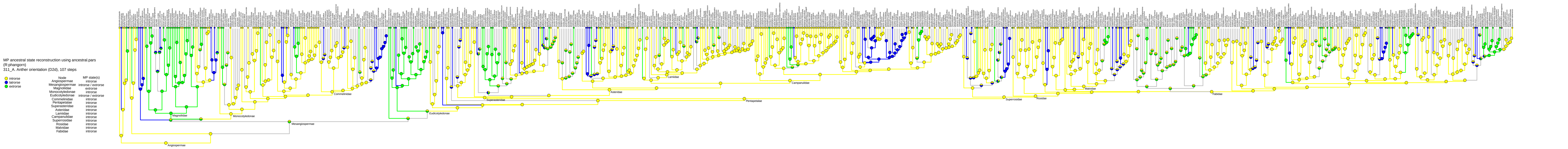
MP ancestral state reconstruction using ancestral.pars
(R:phangorn)
311_A. Anther orientation (D2d), 107 steps
introrse
latrorse
extrorse
Node
MP state(s)
Angiospermae introrse
Mesangiospermae introrse / extrorse
Magnoliidae extrorse
Monocotyledonae introrse
Eudicotyledonae introrse / extrorse
Commelinidae introrse
Pentapetalae introrse
Superasteridae introrse
Asteridae introrse
Lamiidae introrse
Campanulidae introrse
Superrosidae introrse
Rosidae introrse
Malvidae introrse
Fabidae introrse
Angiospermae
Mesangiospermae
Magnoliidae
Monocotyledonae
Commelinidae
Eudicotyledonae
Superasteridae
Asteridae
Lamiidae
Campanulidae
Pentapetalae
Superrosidae
Rosidae
Malvidae
Fabidae

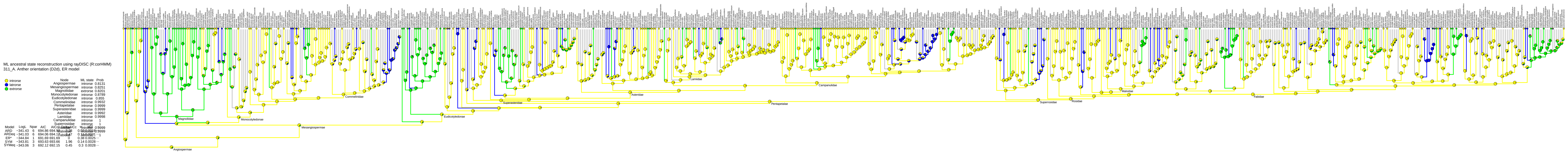

ML ancestral state reconstruction using rayDISC (R:corHMM)
311_A. Anther orientation (D2d), ER model
introrse
latrorse
extrorse
Node | ML state | Prob
Angiospermae | introrse | 0.8131
Mesangiospermae | introrse | 0.8251
Magnoliidae | extrorse | 0.8201
Monocotyledonae | introrse | 0.8789
Eudicotyledonae | introrse | 0.855
Commelinidae | introrse | 0.9932
Pentapetalae | introrse | 0.9999
Superasteridae | introrse | 0.9999
Asteridae | introrse | 0.9992
Lamiidae | introrse | 0.9998
Campanulidae | introrse | 1
Superrosidae | introrse | 1
Rosidae | introrse | 0.9999
Malvidae | introrse | 0.9999
Fabidae | introrse | 1
Model | LogL | Npar | AIC | AICc | DeltaAICc | w | q01
ARD | −341.43 | 6 | 694.86 | 694.97 | 3.28 | 0.07 | 0.0016 ···
ARDeq | −341.03 | 6 | 694.06 | 694.17 | 2.47 | 0.11 | 0.0031 ···
ER* | −344.84 | 1 | 691.69 | 691.69 | 0 | 0.38 | 0.0025 ···
SYM | −343.81 | 3 | 693.63 | 693.66 | 1.96 | 0.14 | 0.0028 ···
SYMeq | −343.06 | 3 | 692.12 | 692.15 | 0.45 | 0.3 | 0.0028 ···

MP ancestral state reconstruction using ancestral.pars
(R:phangorn)
312_A. Anther attachment (binary) (D2d), 97 steps

- basifixed
- dorsifixed

Node	MP state(s)
Angiospermae	basifixed
Mesangiospermae	basifixed
Magnoliidae	basifixed
Monocotyledonae	basifixed
Eudicotyledonae	basifixed
Commelinidae	basifixed
Pentapetalae	basifixed
Superasteridae	basifixed
Asteridae	basifixed
Lamiidae	basifixed
Campanulidae	basifixed / dorsifixed
Superrosidae	basifixed
Rosidae	dorsifixed
Malvidae	dorsifixed
Fabidae	dorsifixed

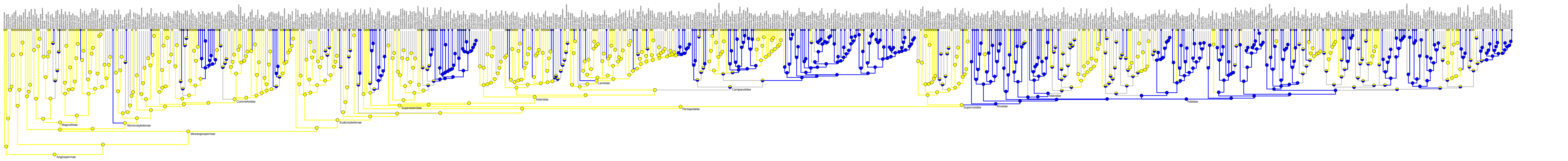

ML ancestral state reconstruction using rayDISC (R:corHMM)
312_A. Anther attachment (binary) (D2d), ARDeq model

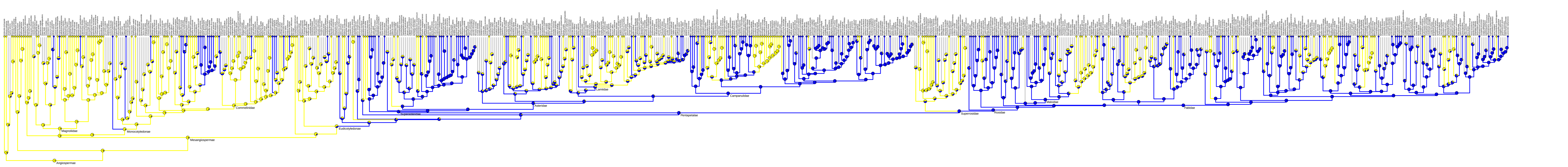

- basifixed
- dorsifixed

Node	ML state	Prob
Angiospermae	basifixed	0.98
Mesangiospermae	basifixed	0.9301
Magnoliidae	basifixed	0.958
Monocotyledonae	basifixed	0.9354
Eudicotyledonae	basifixed	0.6534
Commelinidae	basifixed	0.9446
Pentapetalae	dorsifixed	0.9386
Superasteridae	dorsifixed	0.9417
Asteridae	dorsifixed	0.8878
Lamiidae	dorsifixed	0.5937
Campanulidae	dorsifixed	0.9739
Superrosidae	dorsifixed	0.9448
Rosidae	dorsifixed	0.9666
Malvidae	dorsifixed	0.9803
Fabidae	dorsifixed	0.9999

Model	LogL	Npar	AIC	AICc	DeltaAICc	w	q01	q10
ARD	−264.16	2	532.33	532.34	0.54	0.41	0.004	0.0074
ARDeq**	−263.9	2	531.79	531.81	0	0.54	0.004	0.0072
ER	−267.34	1	536.68	536.69	4.88	0.05	0.0053	0.0053
UNI01	−300.74	1	603.49	603.49	71.69	0	0.0057	
UNI10	−276.35	1	554.69	554.7	22.89	0		0.0074

MP ancestral state reconstruction using ancestral.pars
(R:phangorn)
313_A. Anther dehiscence (3−state) (D2d), 10 steps

- longitudinal slit
- H−valvate
- flap−valvate

Node	MP state(s)
Angiospermae	longitudinal slit
Mesangiospermae	longitudinal slit
Magnoliidae	longitudinal slit
Monocotyledonae	longitudinal slit
Eudicotyledonae	longitudinal slit
Commelinidae	longitudinal slit
Pentapetalae	longitudinal slit
Superasteridae	longitudinal slit
Asteridae	longitudinal slit
Lamiidae	longitudinal slit
Campanulidae	longitudinal slit
Superrosidae	longitudinal slit
Rosidae	longitudinal slit
Malvidae	longitudinal slit
Fabidae	longitudinal slit

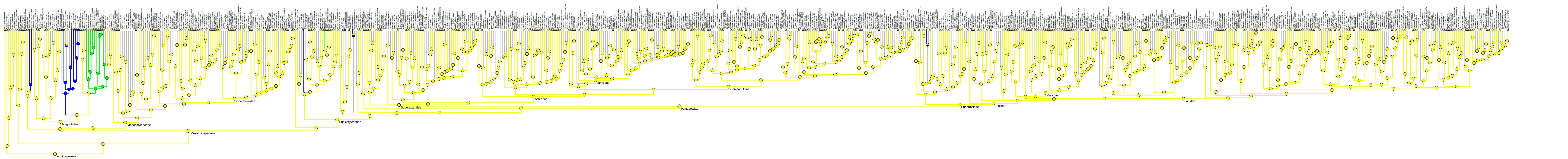

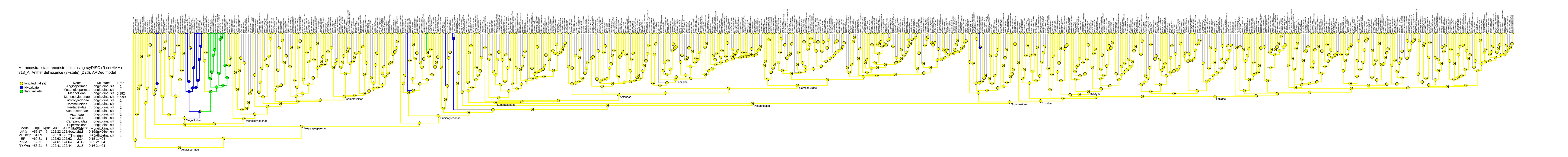

ML ancestral state reconstruction using rayDISC (R:corHMM)
313_A. Anther dehiscence (3−state) (D2d), ARDeq model
longitudinal slit
H−valvate
flap−valvate
Node ML state Prob
Angiospermae longitudinal slit 1
Mesangiospermae longitudinal slit 1
Magnoliidae longitudinal slit 0.992
Monocotyledonae longitudinal slit 0.9999
Eudicotyledonae longitudinal slit 1
Commelinidae longitudinal slit 1
Pentapetalae longitudinal slit 1
Superasteridae longitudinal slit 1
Asteridae longitudinal slit 1
Lamiidae longitudinal slit 1
Campanulidae longitudinal slit 1
Superrosidae longitudinal slit 1
Rosidae longitudinal slit 1
Malvidae longitudinal slit 1
Fabidae longitudinal slit 1
Model LogL Npar AIC AICc DeltaAICc w
ARD −55.17 6 122.33 122.44 2.15 0.16
ARDeq* −54.09 6 120.18 120.29 0
ER −60.31 1 122.62 122.63 2.34 0.15 1e−04 ···
SYM −59.3 3 124.61 124.64 4.35 0.05 2e−04 ···
SYMeq −58.21 3 122.41 122.44 2.15 0.16 2e−04 ···
Angiospermae
Mesangiospermae
Magnoliidae
Monocotyledonae
Commelinidae
Eudicotyledonae
Superasteridae
Pentapetalae
Asteridae
Lamiidae
Campanulidae
Superrosidae
Rosidae
Malvidae
Fabidae

MP ancestral state reconstruction using ancestral.pars
(R:phangorn)
401_B. Number of structural carpels (5−state) (D2c), 188 steps

- one (1)
- two (2)
- three (3)
- four or five (4−5)
- more than five (>5)

Node	MP state(s)
Angiospermae	more than five (>5)
Mesangiospermae	more than five (>5)
Magnoliidae	more than five (>5)
Monocotyledonae	three (3)
Eudicotyledonae	more than five (>5)
Commelinidae	three (3)
Pentapetalae	three (3)
Superasteridae	three (3)
Asteridae	four or five (4−5)
Lamiidae	two (2)
Campanulidae	two (2)
Superrosidae	three (3)
Rosidae	three (3)
Malvidae	four or five (4−5)
Fabidae	four or five (4−5)

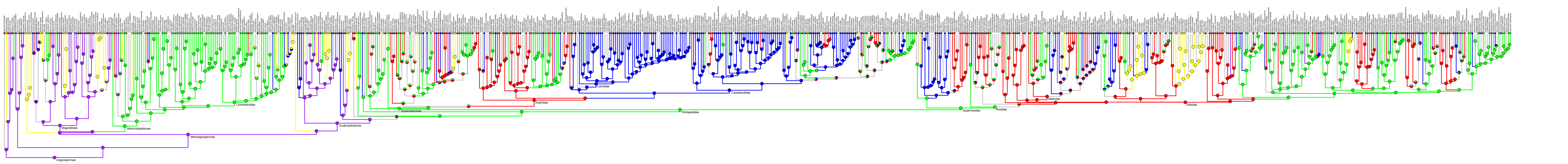

ML ancestral state reconstruction using rayDISC (R:corHMM)
401_B. Number of structural carpels (5−state) (D2c), ARDeq model

- one (1)
- two (2)
- three (3)
- four or five (4−5)
- more than five (>5)

Node	ML state	Prob
Angiospermae	more than five (>5)	1
Mesangiospermae	more than five (>5)	0.9999
Magnoliidae	more than five (>5)	0.9998
Monocotyledonae	more than five (>5)	0.7023
Eudicotyledonae	more than five (>5)	0.9845
Commelinidae	three (3)	0.9999
Pentapetalae	four or five (4−5)	0.9996
Superasteridae	four or five (4−5)	0.9993
Asteridae	four or five (4−5)	0.9917
Lamiidae	two (2)	0.9976
Campanulidae	two (2)	0.9641
Superrosidae	four or five (4−5)	0.9994
Rosidae	four or five (4−5)	0.9993
Malvidae	four or five (4−5)	0.9991
Fabidae	four or five (4−5)	1

Model	LogL	Npar	AIC	AICc	DeltaAICc	w	q01	···
ARD	−665.46	20	1370.91	1372	3.21	0.17	0.0015	···
ARDeq**	−663.85	20	1367.7	1368.79	0	0.83	0.0015	···
ER	−715.93	1	1433.87	1433.87	65.08	0	0.0015	···
SYM	−682.83	10	1385.65	1385.93	17.14	0	0.001	···
SYMeq	−681.56	10	1383.13	1383.41	14.62	0	0.001	···
ORD	−756.03	8	1528.06	1528.25	159.45	0	7e−04	···
ORDeq	−755.01	8	1526.03	1526.21	157.42	0	7e−04	···
ORDSYM	−759.28	4	1526.55	1526.6	157.81	0	0.0039	···
ORDSYMeq	−758.45	4	1524.91	1524.96	156.17	0	0.0039	···
ORDER	−765.02	1	1532.04	1532.05	163.25	0	0.0055	···

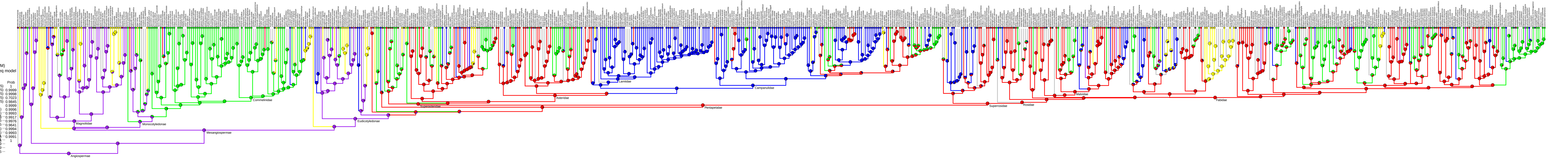

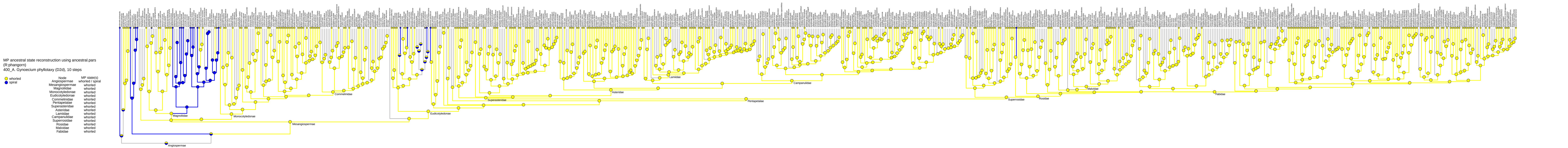
MP ancestral state reconstruction using ancestral.pars
(R:phangorn)
400_A. Gynoecium phyllotaxy (D2d), 10 steps
whorled
spiral
Node
MP state(s)
Angiospermae whorled / spiral
Mesangiospermae whorled
Magnoliidae whorled
Monocotyledonae whorled
Eudicotyledonae whorled
Commelinidae whorled
Pentapetalae whorled
Superasteridae whorled
Asteridae whorled
Lamiidae whorled
Campanulidae whorled
Superrosidae whorled
Rosidae whorled
Malvidae whorled
Fabidae whorled
Angiospermae
Mesangiospermae
Magnoliidae
Monocotyledonae
Commelinidae
Eudicotyledonae
Superasteridae
Asteridae
Lamiidae
Campanulidae
Pentapetalae
Superrosidae
Rosidae
Malvidae
Fabidae

ML ancestral state reconstruction using rayDISC (R:corHMM)
400_A. Gynoecium phyllotaxy (D2d), ARDeq model

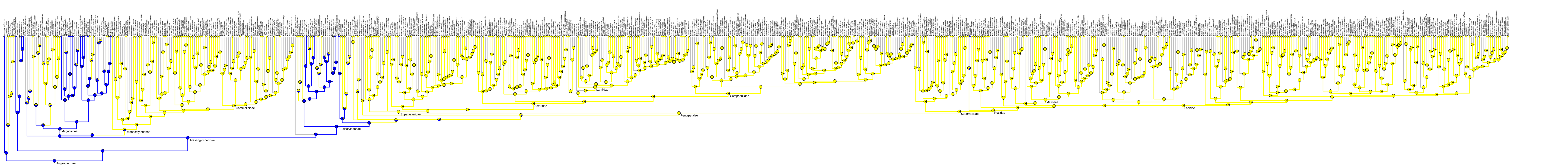

Node	ML state	Prob
Angiospermae	spiral	1
Mesangiospermae	spiral	0.9999
Magnoliidae	spiral	0.9988
Monocotyledonae	whorled	0.5576
Eudicotyledonae	spiral	0.998
Commelinidae	whorled	0.9999
Pentapetalae	whorled	0.9794
Superasteridae	whorled	0.9952
Asteridae	whorled	1
Lamiidae	whorled	1
Campanulidae	whorled	1
Superrosidae	whorled	0.9813
Rosidae	whorled	0.9813
Malvidae	whorled	1
Fabidae	whorled	1

Model	LogL	Npar	AIC	AICc	DeltaAICc	w	q01	q10
ARD	−38.98	2	81.97	81.98	1.39	0.32	1e−04	0.0064
ARDeq**	−38.29	2	80.58	80.59	0	0.63	1e−04	0.0064
ER	−49.14	1	100.29	100.29	19.7	0	4e−04	4e−04
UNI01	−51.05	1	104.11	104.11	23.52	0	4e−04	
UNI10	−41.74	1	85.47	85.48	4.88	0.05		0.0075

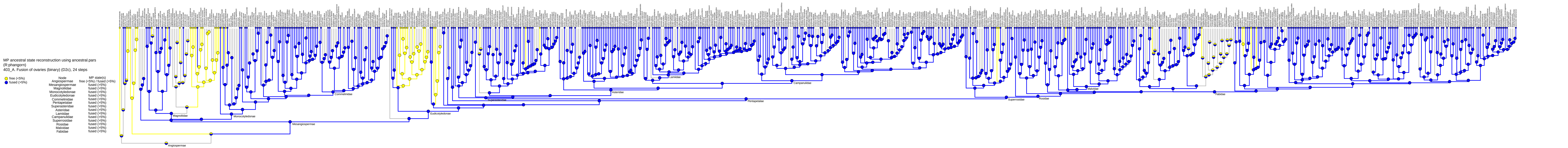
MP ancestral state reconstruction using ancestral.pars
(R:phangorn)
403_A. Fusion of ovaries (binary) (D2c), 24 steps
free (<5%)
fused (>5%)
Node
MP state(s)
Angiospermae
free (<5%) / fused (>5%)
Mesangiospermae
fused (>5%)
Magnoliidae
fused (>5%)
Monocotyledonae
fused (>5%)
Eudicotyledonae
fused (>5%)
Commelinidae
fused (>5%)
Pentapetalae
fused (>5%)
Superasteridae
fused (>5%)
Asteridae
fused (>5%)
Lamiidae
fused (>5%)
Campanulidae
fused (>5%)
Superrosidae
fused (>5%)
Rosidae
fused (>5%)
Malvidae
fused (>5%)
Fabidae
fused (>5%)
Angiospermae
Mesangiospermae
Magnoliidae
Monocotyledonae
Commelinidae
Eudicotyledonae
Pentapetalae
Asteridae
Lamiidae
Campanulidae
Superrosidae
Rosidae
Malvidae
Fabidae

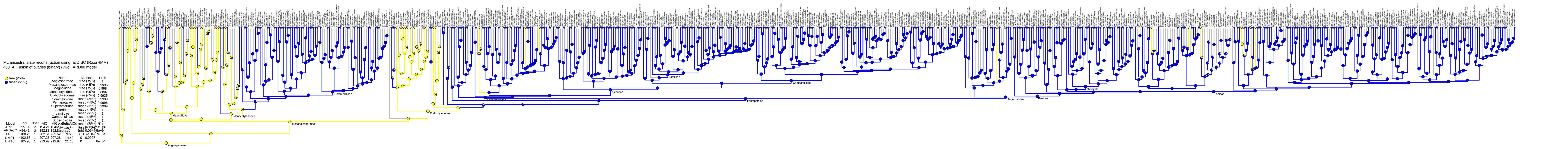

ML ancestral state reconstruction using rayDISC (R:corHMM)
403_A. Fusion of ovaries (binary) (D2c), ARDeq model
free (<5%)
fused (>5%)
Node ML state Prob
Angiospermae free (<5%) 1
Mesangiospermae free (<5%) 0.9995
Magnoliidae free (<5%) 0.998
Monocotyledonae free (<5%) 0.8807
Eudicotyledonae free (<5%) 0.9935
Commelinidae fused (>5%) 0.9999
Pentapetalae fused (>5%) 0.9996
Superasteridae fused (>5%) 0.9999
Asteridae fused (>5%) 1
Lamiidae fused (>5%) 1
Campanulidae fused (>5%) 1
Superrosidae fused (>5%) 1
Rosidae
Malvidae
Fabidae
Model LogL Npar AIC AICc DeltaAICc w q01 q10
ARD −95.11 2 194.21 194.23 1.38 0.33 0.0044 5e−04
ARDeq** −94.41 2 192.83 192.84 0 0.66 0.0044 5e−04
ER −100.26 1 202.51 202.52 9.68 0.01 7e−04 7e−04
UNI01 −102.63 1 207.26 207.26 14.42 0 0.0097
UNI10 −105.98 1 213.97 213.97 21.13 0 8e−04
Angiospermae
Mesangiospermae
Magnoliidae
Monocotyledonae
Commelinidae
Eudicotyledonae
Asteridae
Lamiidae
Campanulidae
Pentapetalae
Superrosidae
Rosidae
Malvidae
Fabidae

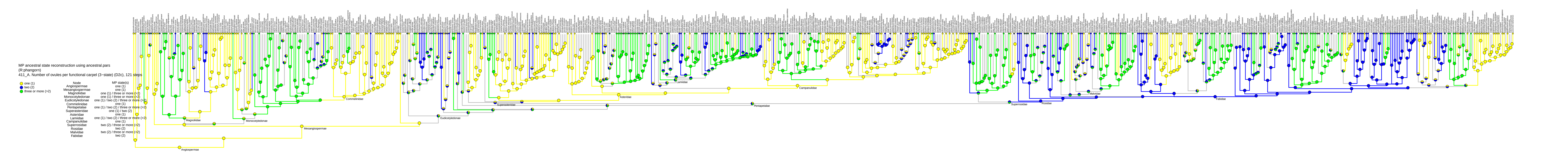
MP ancestral state reconstruction using ancestral.pars
(R:phangorn)
411_A. Number of ovules per functional carpel (3−state) (D2c), 121 steps
one (1)
two (2)
three or more (>2)
Node
MP state(s)
Angiospermae
one (1)
Mesangiospermae
one (1)
Magnoliidae
one (1) / three or more (>2)
Monocotyledonae
one (1) / three or more (>2)
Eudicotyledonae
one (1) / two (2) / three or more (>2)
Commelinidae
one (1)
Pentapetalae
one (1) / two (2) / three or more (>2)
Superasteridae
one (1) / two (2)
Asteridae
one (1)
Lamiidae
one (1) / two (2) / three or more (>2)
Campanulidae
one (1)
Superrosidae
two (2) / three or more (>2)
Rosidae
two (2)
Malvidae
two (2) / three or more (>2)
Fabidae
two (2)
Angiospermae
Mesangiospermae
Magnoliidae
Monocotyledonae
Commelinidae
Eudicotyledonae
Superasteridae
Asteridae
Lamiidae
Campanulidae
Pentapetalae
Superrosidae
Rosidae
Malvidae
Fabidae

ML ancestral state reconstruction using rayDISC (R:corHMM)
411_A. Number of ovules per functional carpel (3−state) (D2c), ARDeq model

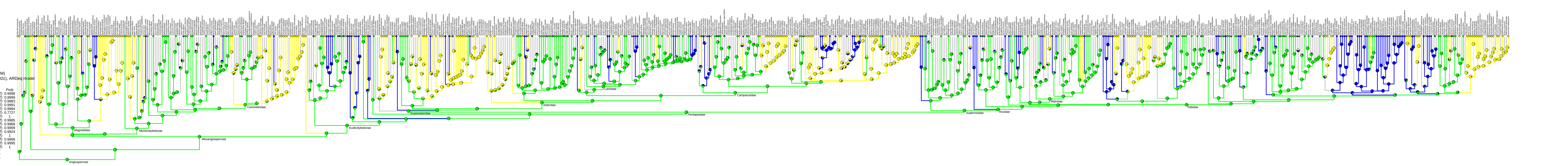

- one (1)
- two (2)
- three or more (>2)

Node	ML state	Prob
Angiospermae	three or more (>2)	0.9998
Mesangiospermae	three or more (>2)	0.9999
Magnoliidae	three or more (>2)	0.9983
Monocotyledonae	three or more (>2)	0.9991
Eudicotyledonae	three or more (>2)	0.9994
Commelinidae	three or more (>2)	0.7727
Pentapetalae	three or more (>2)	1
Superasteridae	three or more (>2)	0.9985
Asteridae	three or more (>2)	0.9968
Lamiidae	three or more (>2)	0.9999
Campanulidae	three or more (>2)	0.9924
Superrosidae	three or more (>2)	1
Rosidae	three or more (>2)	0.9999
Malvidae	three or more (>2)	0.9995
Fabidae	three or more (>2)	1

Model	LogL	Npar	AIC	AICc	DeltaAICc	w	q01	
ARD	−389.27	6	790.54	790.65	1.91	0.28	0.0013	···
ARDeq**	−388.32	6	788.63	788.74	0	0.72	0.0013	···
ER	−400.17	1	802.33	802.34	13.6	0	0.0027	···
SYM	−399.58	3	805.17	805.2	16.46	0	0.0025	···
SYMeq	−398.85	3	803.69	803.72	14.98	0	0.0025	···
ORD	−397.11	4	802.22	802.27	13.53	0	0	···
ORDeq	−400.66	4	809.32	809.37	20.63	0	0.0023	···
ORDSYM	−410.87	2	825.75	825.76	37.02	0	0.0052	···
ORDSYMeq	−410.62	2	825.24	825.26	36.52	0	0.0052	···
ORDER	−411.98	1	825.95	825.96	37.22	0	0.006	···
