# Supplementary material for: The ancestral flower of angiosperms and its early diversification
Source: Nat Commun. 2017 Aug 1;8:16047. doi: 10.1038/ncomms16047 (PMC5543309; doi:10.1038/ncomms16047)

MP ancestral state reconstruction using ancestral.pars
(R:phangorn)
100_A. Functional sex of flowers (D2d), 97 steps

- bisexual
- unisexual

| Node | MP state(s) |
|---|---|
| Angiospermae | bisexual / unisexual |
| Mesangiospermae | bisexual |
| Magnoliidae | bisexual |
| Monocotyledonae | bisexual |
| Eudicotyledonae | bisexual |
| Commelinidae | bisexual |
| Pentapetalae | bisexual |
| Superasteridae | bisexual |
| Asteridae | bisexual |
| Lamiidae | bisexual |
| Campanulidae | bisexual |
| Superrosidae | bisexual |
| Rosidae | bisexual |
| Malvidae | bisexual |
| Fabidae | bisexual |

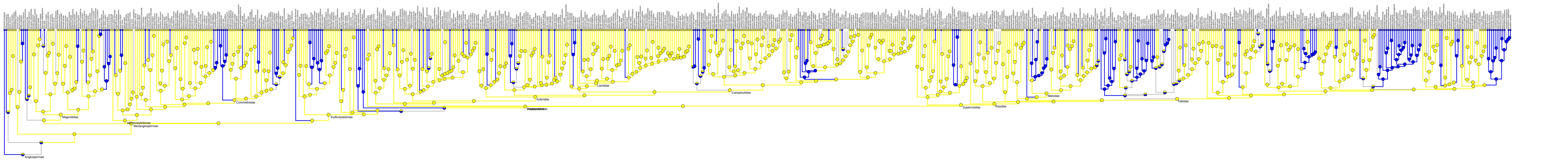

ML ancestral state reconstruction using rayDISC (R:corHMM)
100_A. Functional sex of flowers (D2d), ARDeq model

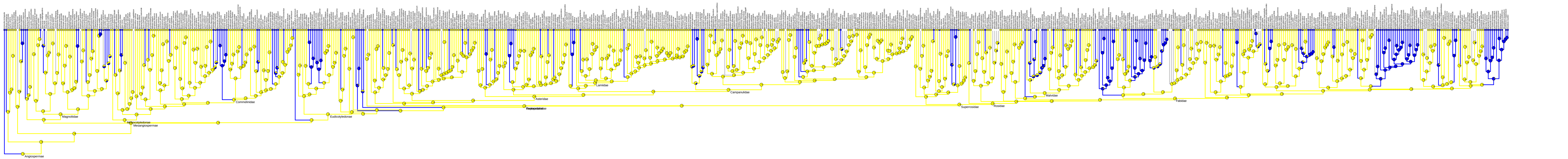


| Node | ML state | Prob |
|---|---|---|
| Angiospermae | bisexual | 0.9999 |
| Mesangiospermae | bisexual | 1 |
| Magnoliidae | bisexual | 1 |
| Monocotyledonae | bisexual | 1 |
| Eudicotyledonae | bisexual | 1 |
| Commelinidae | bisexual | 0.9998 |
| Pentapetalae | bisexual | 1 |
| Superasteridae | bisexual | 1 |
| Asteridae | bisexual | 1 |
| Lamiidae | bisexual | 1 |
| Campanulidae | bisexual | 0.9995 |
| Superrosidae | bisexual | 1 |
| Rosidae | bisexual | 1 |
| Malvidae | bisexual | 0.9997 |
| Fabidae | bisexual | 1 |

| Model | LogL | Npar | AIC | AICc | DeltaAICc | w | q01 | q10 |
|---|---|---|---|---|---|---|---|---|
| ARD | −314.26 | 2 | 632.51 | 632.53 | 1.23 | 0.25 | 0.0028 | 8e−04 |
| ARDeq* | −313.64 | 2 | 631.28 | 631.3 | 0 | 0.47 | 0.0028 | 8e−04 |
| ER | −316.61 | 1 | 635.21 | 635.22 | 3.92 | 0.07 | 0.0027 | 0.0027 |
| UNI01 | −315.43 | 1 | 632.86 | 632.86 | 1.57 | 0.21 | 0.0029 | |
| UNI10 | −361.08 | 1 | 724.16 | 724.16 | 92.87 | 0 | | 0.011 |

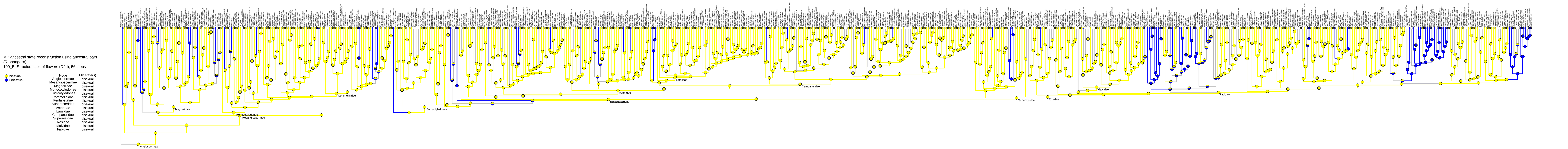

MP ancestral state reconstruction using ancestral.pars
(R:phangorn)
100_B. Structural sex of flowers (D2d), 56 steps
bisexual
unisexual
Node
MP state(s)
Angiospermae bisexual
Mesangiospermae bisexual
Magnoliidae bisexual
Monocotyledonae bisexual
Eudicotyledonae bisexual
Commelinidae bisexual
Pentapetalae bisexual
Superasteridae bisexual
Asteridae bisexual
Lamiidae bisexual
Campanulidae bisexual
Superrosidae bisexual
Rosidae bisexual
Malvidae bisexual
Fabidae bisexual
Angiospermae
Magnoliidae
Monocotyledonae
Mesangiospermae
Commelinidae
Eudicotyledonae
Asteridae
Lamiidae
Campanulidae
Superrosidae
Rosidae
Malvidae
Fabidae

ML ancestral state reconstruction using rayDISC (R:corHMM)
100_B. Structural sex of flowers (D2d), ARDeq model

- bisexual
- unisexual

| Node | ML state | Prob |
|---|---|---|
| Angiospermae | bisexual | 0.9943 |
| Mesangiospermae | bisexual | 0.9996 |
| Magnoliidae | bisexual | 0.9997 |
| Monocotyledonae | bisexual | 0.9997 |
| Eudicotyledonae | bisexual | 0.9998 |
| Commelinidae | bisexual | 0.9999 |
| Pentapetalae | bisexual | 0.9997 |
| Superasteridae | bisexual | 0.9997 |
| Asteridae | bisexual | 1 |
| Lamiidae | bisexual | 0.9999 |
| Campanulidae | bisexual | 1 |
| Superrosidae | bisexual | 1 |
| Rosidae | bisexual | 1 |
| Malvidae | bisexual | 1 |
| Fabidae | bisexual | 0.9966 |

| Model | LogL | Npar | AIC | AICc | DeltaAICc | w | q01 | q10 |
|---|---|---|---|---|---|---|---|---|
| ARD | −208.18 | 2 | 420.35 | 420.37 | 0.77 | 0.36 | 0.0014 | 0.0041 |
| ARDeq** | −207.79 | 2 | 419.58 | 419.59 | 0 | 0.52 | 0.0014 | 0.0039 |
| ER | −210.26 | 1 | 422.52 | 422.53 | 2.93 | 0.12 | 0.0015 | 0.0015 |
| UNI01 | −223.38 | 1 | 448.77 | 448.77 | 29.18 | 0 | 0.0016 | |
| UNI10 | −234.9 | 1 | 471.8 | 471.81 | 52.22 | 0 | | 0.0153 |

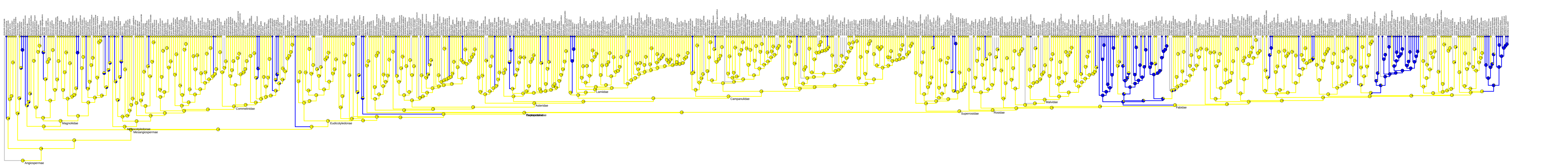

MP ancestral state reconstruction using ancestral.pars
(R:phangorn)
102_B. Ovary position (binary) (D2d), 81 steps

- superior
- inferior

| Node | MP state(s) |
|---|---|
| Angiospermae | superior |
| Mesangiospermae | superior |
| Magnoliidae | superior |
| Monocotyledonae | superior |
| Eudicotyledonae | superior |
| Commelinidae | superior |
| Pentapetalae | superior |
| Superasteridae | superior |
| Asteridae | superior |
| Lamiidae | superior |
| Campanulidae | superior / inferior |
| Superrosidae | superior / inferior |
| Rosidae | superior / inferior |
| Malvidae | superior |
| Fabidae | superior |

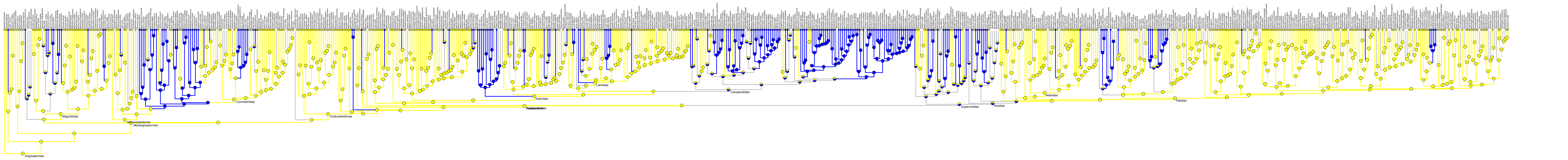

ML ancestral state reconstruction using rayDISC (R:corHMM)
102_B. Ovary position (binary) (D2d), ARDeq model

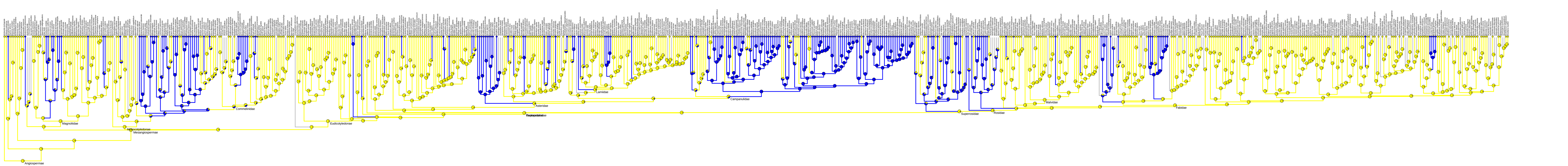


- superior (yellow)
- inferior (blue)

| Node | ML state | Prob |
|---|---|---|
| Angiospermae | superior | 0.9997 |
| Mesangiospermae | superior | 0.9993 |
| Magnoliidae | superior | 0.9958 |
| Monocotyledonae | superior | 0.9931 |
| Eudicotyledonae | superior | 0.9999 |
| Commelinidae | superior | 0.6208 |
| Pentapetalae | superior | 0.9985 |
| Superasteridae | superior | 0.9985 |
| Asteridae | superior | 0.9395 |
| Lamiidae | superior | 0.9533 |
| Campanulidae | superior | 0.706 |
| Superrosidae | superior | 0.9727 |
| Rosidae | superior | 0.9724 |
| Malvidae | superior | 1 |
| Fabidae | superior | 0.9996 |

| Model | LogL | Npar | AIC | AICc | DeltaAICc | w | q01 | q10 |
|---|---|---|---|---|---|---|---|---|
| ARD | −271.79 | 2 | 547.58 | 547.6 | 1.14 | 0.3 | 0.0019 | 0.0037 |
| ARDeq** | −271.22 | 2 | 546.44 | 546.46 | 0 | 0.53 | 0.0019 | 0.0036 |
| ER | −273.41 | 1 | 548.82 | 548.83 | 2.37 | 0.16 | 0.0023 | 0.0023 |
| UNI01 | −285.14 | 1 | 572.28 | 572.28 | 25.83 | 0 | 0.0026 | |
| UNI10 | −295.89 | 1 | 593.77 | 593.78 | 47.32 | 0 | | 0.0093 |

MP ancestral state reconstruction using ancestral.pars
(R:phangorn)
201_A. Perianth presence (D2c), 19 steps

- absent
- present

| Node | MP state(s) |
|---|---|
| Angiospermae | present |
| Mesangiospermae | present |
| Magnoliidae | present |
| Monocotyledonae | present |
| Eudicotyledonae | present |
| Commelinidae | present |
| Pentapetalae | present |
| Superasteridae | present |
| Asteridae | present |
| Lamiidae | present |
| Campanulidae | present |
| Superrosidae | present |
| Rosidae | present |
| Malvidae | present |
| Fabidae | present |

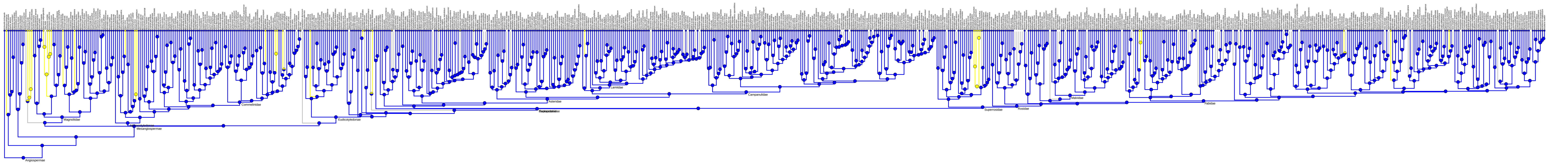

ML ancestral state reconstruction using rayDISC (R:corHMM)
201_A. Perianth presence (D2c), UNI10 model

- absent
- present

| Node | ML state | Prob |
|---|---|---|
| Angiospermae | present | 1 |
| Mesangiospermae | present | 1 |
| Magnoliidae | present | 1 |
| Monocotyledonae | present | 1 |
| Eudicotyledonae | present | 1 |
| Commelinidae | present | 1 |
| Pentapetalae | present | 1 |
| Superasteridae | present | 1 |
| Asteridae | present | 1 |
| Lamiidae | present | 1 |
| Campanulidae | present | 1 |
| Superrosidae | present | 1 |
| Rosidae | present | 1 |
| Malvidae | present | 1 |
| Fabidae | present | 1 |

| Model | LogL | Npar | AIC | AICc | DeltaAICc | w | q01 | q10 |
|---|---|---|---|---|---|---|---|---|
| ARD | −94.29 | 2 | 192.58 | 192.59 | 2.01 | 0.13 | 0 | 4e−04 |
| ARDeq | −93.59 | 2 | 191.19 | 191.21 | 0.62 | 0.26 | 0 | 4e−04 |
| ER | −94.6 | 1 | 191.21 | 191.21 | 0.63 | 0.26 | 4e−04 | 4e−04 |
| UNI01 | −112.42 | 1 | 226.83 | 226.84 | 36.26 | 0 | 0.0196 | |
| UNI10* | −94.29 | 1 | 190.58 | 190.58 | 0 | 0.35 | | 4e−04 |

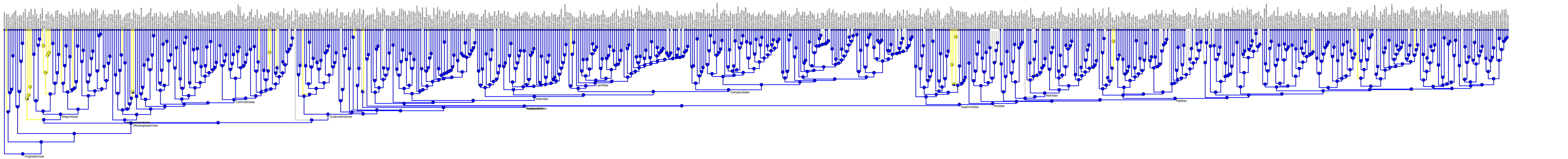

MP ancestral state reconstruction using ancestral.pars
(R:phangorn)
201_B. Number of perianth parts (3−state) (D2c), 86 steps

- one to five (1−5)
- six to ten (6−10)
- more than ten (>10)

| Node | MP state(s) |
|---|---|
| Angiospermae | six to ten (6−10) / more than ten (>10) |
| Mesangiospermae | six to ten (6−10) |
| Magnoliidae | six to ten (6−10) |
| Monocotyledonae | six to ten (6−10) |
| Eudicotyledonae | six to ten (6−10) |
| Commelinidae | six to ten (6−10) |
| Pentapetalae | six to ten (6−10) |
| Superasteridae | six to ten (6−10) |
| Asteridae | six to ten (6−10) |
| Lamiidae | six to ten (6−10) |
| Campanulidae | six to ten (6−10) |
| Superrosidae | six to ten (6−10) |
| Rosidae | six to ten (6−10) |
| Malvidae | six to ten (6−10) |
| Fabidae | six to ten (6−10) |

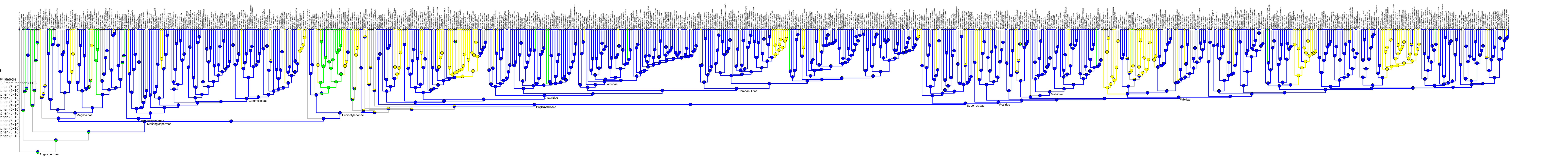

ML ancestral state reconstruction using rayDISC (R:corHMM)
201_B. Number of perianth parts (3−state) (D2c), ARDeq model

- one to five (1−5)
- six to ten (6−10)
- more than ten (>10)

| Node | ML state | Prob |
|---|---|---|
| Angiospermae | more than ten (>10) | 1 |
| Mesangiospermae | more than ten (>10) | 0.9991 |
| Magnoliidae | more than ten (>10) | 0.9994 |
| Monocotyledonae | more than ten (>10) | 0.6023 |
| Eudicotyledonae | more than ten (>10) | 0.9893 |
| Commelinidae | six to ten (6−10) | 0.9999 |
| Pentapetalae | six to ten (6−10) | 0.9788 |
| Superasteridae | six to ten (6−10) | 0.9788 |
| Asteridae | six to ten (6−10) | 1 |
| Lamiidae | six to ten (6−10) | 0.9999 |
| Campanulidae | six to ten (6−10) | 1 |
| Superrosidae | six to ten (6−10) | 0.9992 |
| Rosidae | six to ten (6−10) | 0.9994 |
| Malvidae | six to ten (6−10) | 0.9998 |
| Fabidae | six to ten (6−10) | 0.9987 |

| Model | LogL | Npar | AIC | AICc | DeltaAICc | w | q01 | |
|---|---|---|---|---|---|---|---|---|
| ARD | −326.05 | 6 | 664.11 | 664.22 | 2.11 | 0.25 | 0.0038 | ··· |
| ARDeq** | −325 | 6 | 662 | 662.1 | 0 | 0.73 | 0.0038 | ··· |
| ER | −350.38 | 1 | 702.76 | 702.76 | 40.66 | 0 | 0.0012 | ··· |
| SYM | −339.37 | 3 | 684.73 | 684.76 | 22.66 | 0 | 0.0019 | ··· |
| SYMeq | −338.56 | 3 | 683.11 | 683.14 | 21.04 | 0 | 0.0019 | ··· |
| ORD | −332.11 | 4 | 672.21 | 672.26 | 10.16 | 0 | 0.0039 | ··· |
| ORDeq | −331.04 | 4 | 670.07 | 670.13 | 8.02 | 0.01 | 0.0039 | ··· |
| ORDSYM | −342.14 | 2 | 688.27 | 688.29 | 26.19 | 0 | 0.002 | ··· |
| ORDSYMeq | −341.44 | 2 | 686.89 | 686.9 | 24.8 | 0 | 0.0019 | ··· |
| ORDER | −351.49 | 1 | 704.98 | 704.98 | 42.88 | 0 | 0.0013 | ··· |

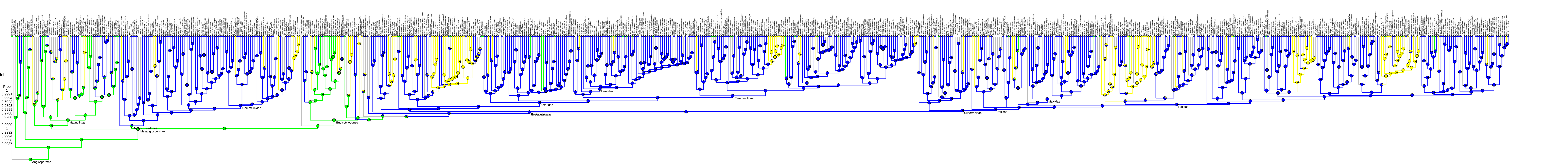

MP ancestral state reconstruction using ancestral.pars
(R:phangorn)
201_C. Number of perianth parts (binary) (D2c), 69 steps

- one to six (1−6)
- more than six (>6)

| Node | MP state(s) |
| --- | --- |
| Angiospermae | more than six (>6) |
| Mesangiospermae | one to six (1−6) / more than six (>6) |
| Magnoliidae | one to six (1−6) / more than six (>6) |
| Monocotyledonae | one to six (1−6) |
| Eudicotyledonae | one to six (1−6) |
| Commelinidae | one to six (1−6) |
| Pentapetalae | more than six (>6) |
| Superasteridae | more than six (>6) |
| Asteridae | more than six (>6) |
| Lamiidae | more than six (>6) |
| Campanulidae | more than six (>6) |
| Superrosidae | more than six (>6) |
| Rosidae | more than six (>6) |
| Malvidae | more than six (>6) |
| Fabidae | more than six (>6) |

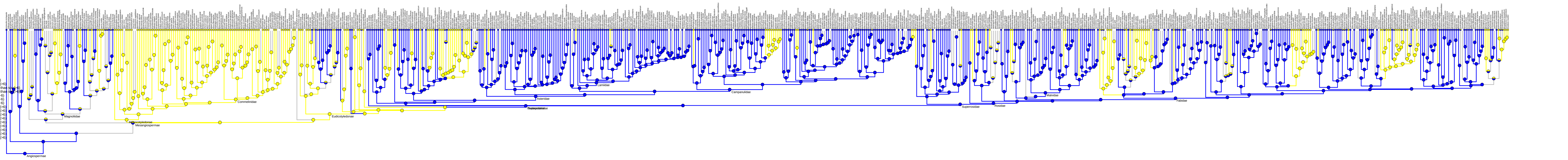

ML ancestral state reconstruction using rayDISC (R:corHMM)
201_C. Number of perianth parts (binary) (D2c), ARDeq model

- one to six (1−6)
- more than six (>6)

| Node | ML state | Prob |
|---|---|---|
| Angiospermae | more than six (>6) | 1 |
| Mesangiospermae | more than six (>6) | 0.9995 |
| Magnoliidae | more than six (>6) | 0.9999 |
| Monocotyledonae | one to six (1−6) | 0.5951 |
| Eudicotyledonae | more than six (>6) | 0.9998 |
| Commelinidae | one to six (1−6) | 1 |
| Pentapetalae | more than six (>6) | 1 |
| Superasteridae | more than six (>6) | 1 |
| Asteridae | more than six (>6) | 1 |
| Lamiidae | more than six (>6) | 1 |
| Campanulidae | more than six (>6) | 1 |
| Superrosidae | more than six (>6) | 1 |
| Rosidae | more than six (>6) | 1 |
| Malvidae | more than six (>6) | 1 |
| Fabidae | more than six (>6) | 1 |

| Model | LogL | Npar | AIC | AICc | DeltaAICc | w | q01 | q10 |
|---|---|---|---|---|---|---|---|---|
| ARD | −246.96 | 2 | 497.93 | 497.94 | 1.38 | 0.33 | 6e−04 | 0.0024 |
| ARDeq** | −246.28 | 2 | 496.55 | 496.57 | 0 | 0.66 | 6e−04 | 0.0024 |
| ER | −253.1 | 1 | 508.19 | 508.2 | 11.63 | 0 | 0.002 | 0.002 |
| UNI01 | −326.66 | 1 | 655.33 | 655.33 | 158.77 | 0 | 0.0076 | |
| UNI10 | −251.29 | 1 | 504.59 | 504.59 | 8.03 | 0.01 | | 0.0028 |

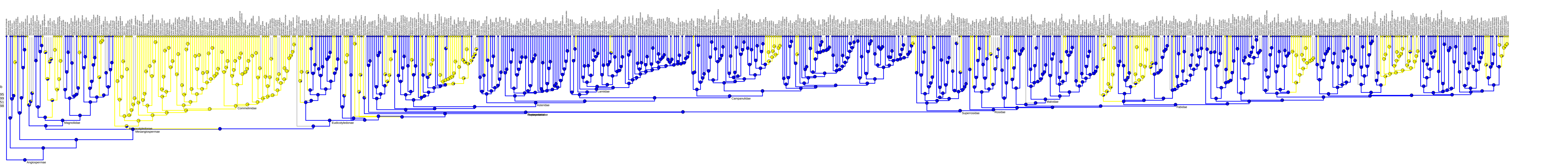

MP ancestral state reconstruction using ancestral.pars
(R:phangorn)
230_A. Perianth phyllotaxy (binary) (D2d), 16 steps

whorled
spiral

| Node | MP state(s) |
|---|---|
| Angiospermae | whorled / spiral |
| Mesangiospermae | whorled |
| Magnoliidae | whorled |
| Monocotyledonae | whorled |
| Eudicotyledonae | whorled |
| Commelinidae | whorled |
| Pentapetalae | whorled |
| Superasteridae | whorled |
| Asteridae | whorled |
| Lamiidae | whorled |
| Campanulidae | whorled |
| Superrosidae | whorled |
| Rosidae | whorled |
| Malvidae | whorled |
| Fabidae | whorled |

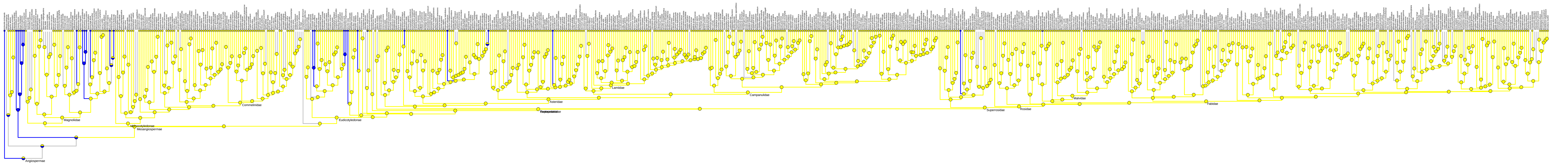

ML ancestral state reconstruction using rayDISC (R:corHMM)
230_A. Perianth phyllotaxy (binary) (D2d), ARDeq model

- whorled
- spiral

| Node | ML state | Prob |
|---|---|---|
| Angiospermae | spiral | 1 |
| Mesangiospermae | spiral | 0.9997 |
| Magnoliidae | spiral | 0.9975 |
| Monocotyledonae | spiral | 0.6441 |
| Eudicotyledonae | spiral | 0.9986 |
| Commelinidae | whorled | 0.9999 |
| Pentapetalae | whorled | 0.9779 |
| Superasteridae | whorled | 0.9779 |
| Asteridae | whorled | 0.9999 |
| Lamiidae | whorled | 1 |
| Campanulidae | whorled | 1 |
| Superrosidae | whorled | 0.9966 |
| Rosidae | whorled | 0.998 |
| Malvidae | whorled | 1 |
| Fabidae | whorled | 1 |

| Model | LogL | Npar | AIC | AICc | DeltaAICc | w | q01 | q10 |
|---|---|---|---|---|---|---|---|---|
| ARD | −70.42 | 2 | 144.85 | 144.86 | 1.38 | 0.33 | 2e−04 | 0.0092 |
| ARDeq** | −69.73 | 2 | 143.46 | 143.48 | 0 | 0.66 | 2e−04 | 0.0092 |
| ER | −76.67 | 1 | 155.35 | 155.36 | 11.88 | 0 | 4e−04 | 4e−04 |
| UNI01 | −77.07 | 1 | 156.15 | 156.15 | 12.67 | 0 | 4e−04 | |
| UNI10 | −80.22 | 1 | 162.44 | 162.44 | 18.96 | 0 | | 0.0161 |

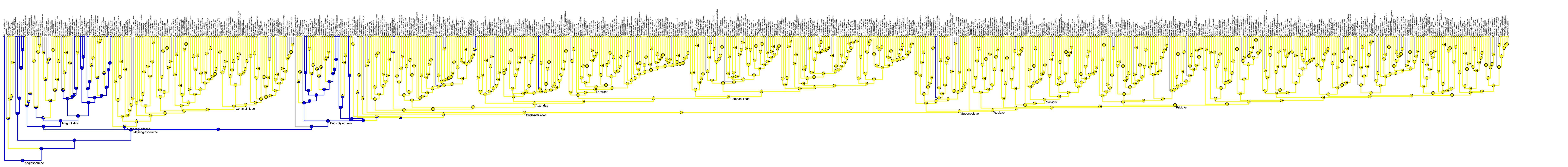

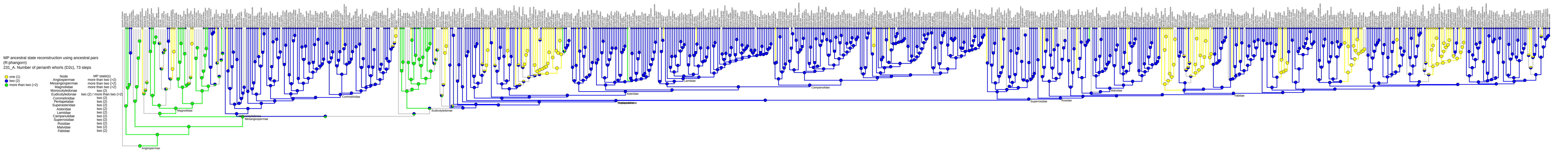
MP ancestral state reconstruction using ancestral.pars
(R:phangorn)
231_A. Number of perianth whorls (D2c), 73 steps
one (1)
two (2)
more than two (>2)
Node
MP state(s)
Angiospermae
more than two (>2)
Mesangiospermae
more than two (>2)
Magnoliidae
more than two (>2)
Monocotyledonae
two (2)
Eudicotyledonae
two (2) / more than two (>2)
Commelinidae
two (2)
Pentapetalae
two (2)
Superasteridae
two (2)
Asteridae
two (2)
Lamiidae
two (2)
Campanulidae
two (2)
Superrosidae
two (2)
Rosidae
two (2)
Malvidae
two (2)
Fabidae
two (2)

ML ancestral state reconstruction using rayDISC (R:corHMM)
231_A. Number of perianth whorls (D2c), ARDeq model

- one (1)
- two (2)
- more than two (>2)

| Node | ML state | Prob |
|---|---|---|
| Angiospermae | more than two (>2) | 1 |
| Mesangiospermae | more than two (>2) | 0.9995 |
| Magnoliidae | more than two (>2) | 1 |
| Monocotyledonae | two (2) | 0.4988 |
| Eudicotyledonae | more than two (>2) | 0.9846 |
| Commelinidae | two (2) | 1 |
| Pentapetalae | two (2) | 0.9992 |
| Superasteridae | two (2) | 0.9992 |
| Asteridae | two (2) | 0.9998 |
| Lamiidae | two (2) | 0.9999 |
| Campanulidae | two (2) | 1 |
| Superrosidae | two (2) | 1 |
| Rosidae | two (2) | 1 |
| Malvidae | two (2) | 0.9999 |
| Fabidae | two (2) | 0.9971 |

| Model | LogL | Npar | AIC | AICc | DeltaAICc | w | q01 | ··· |
|---|---|---|---|---|---|---|---|---|
| ARD | −268.31 | 6 | 548.61 | 548.72 | 2.17 | 0.25 | 0.0045 | ··· |
| ARDeq** | −267.22 | 6 | 546.44 | 546.55 | 0 | 0.74 | 0.0045 | ··· |
| ER | −308.97 | 1 | 619.94 | 619.94 | 73.4 | 0 | 0.001 | ··· |
| SYM | −291.07 | 3 | 588.13 | 588.16 | 41.62 | 0 | 0.0018 | ··· |
| SYMeq | −290.62 | 3 | 587.24 | 587.27 | 40.72 | 0 | 0.0018 | ··· |
| ORD | −274.98 | 4 | 557.96 | 558.01 | 11.46 | 0 | 0.0043 | ··· |
| ORDeq | −273.88 | 4 | 555.77 | 555.82 | 9.27 | 0.01 | 0.0043 | ··· |
| ORDSYM | −297.42 | 2 | 598.83 | 598.85 | 52.3 | 0 | 0.0018 | ··· |
| ORDSYMeq | −296.69 | 2 | 597.39 | 597.4 | 50.86 | 0 | 0.0018 | ··· |
| ORDER | −312.18 | 1 | 626.36 | 626.36 | 79.82 | 0 | 0.0011 | ··· |

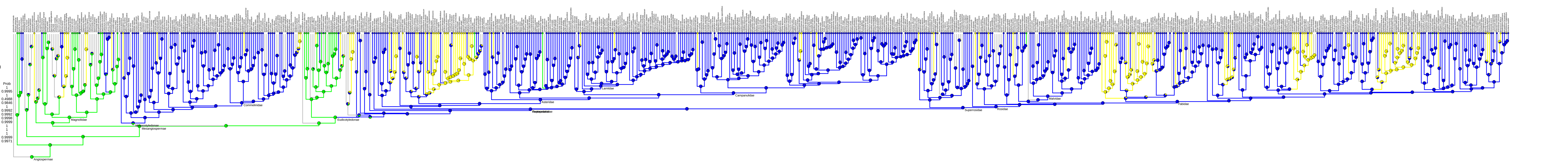

MP ancestral state reconstruction using ancestral.pars
(R:phangorn)
232_A. Perianth merism (4−state) (D2c), 84 steps

- dimerous
- trimerous
- tetramerous
- pentamerous

| Node | MP state(s) |
| --- | --- |
| Angiospermae | trimerous |
| Mesangiospermae | trimerous |
| Magnoliidae | trimerous |
| Monocotyledonae | trimerous |
| Eudicotyledonae | dimerous / trimerous |
| Commelinidae | trimerous |
| Pentapetalae | pentamerous |
| Superasteridae | pentamerous |
| Asteridae | pentamerous |
| Lamiidae | pentamerous |
| Campanulidae | pentamerous |
| Superrosidae | pentamerous |
| Rosidae | pentamerous |
| Malvidae | pentamerous |
| Fabidae | pentamerous |

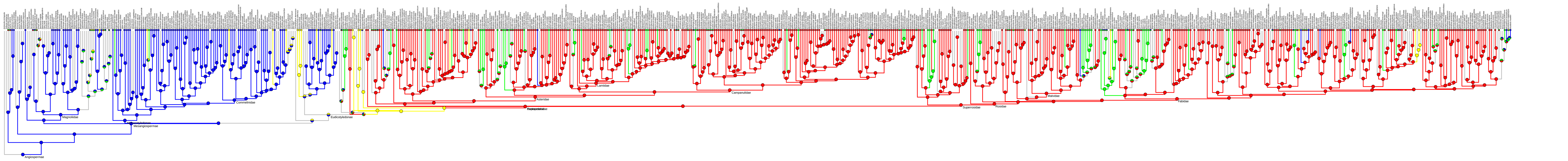

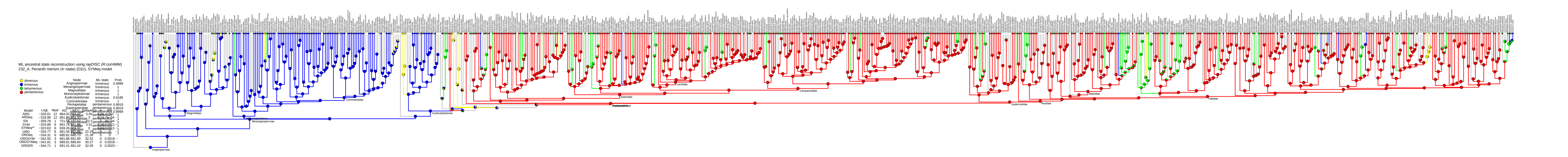


| Node | ML state | Prob |
|---|---|---|
| Angiospermae | trimerous | 0.9999 |
| Mesangiospermae | trimerous | 1 |
| Magnoliidae | trimerous | 1 |
| Monocotyledonae | trimerous | 1 |
| Eudicotyledonae | trimerous | 0.9185 |
| Commelinidae | trimerous | 1 |
| Pentapetalae | pentamerous | 0.9919 |
| Superasteridae | pentamerous | 0.9919 |
| Asteridae | pentamerous | 0.9999 |
| Lamiidae | pentamerous | 1 |
| Campanulidae | pentamerous | 1 |
| Superrosidae | pentamerous | 1 |
| Rosidae | pentamerous | 1 |
| Malvidae | pentamerous | 1 |
| Fabidae | pentamerous | 1 |

| Model | LogL | Npar | AIC | AICc | DeltaAICc | w | q01 | |
|---|---|---|---|---|---|---|---|---|
| ARD | −320.01 | 12 | 664.02 | 664.42 | 5.05 | 0.05 | 0.001 | ··· |
| ARDeq | −318.98 | 12 | 661.97 | 662.37 | 3 | 0.14 | 7e−04 | ··· |
| ER | −359.78 | 1 | 721.56 | 721.57 | 62.2 | 0 | 8e−04 | ··· |
| SYM | −324.89 | 6 | 661.78 | 661.88 | 2.51 | 0.18 | 0.0011 | ··· |
| SYMeq** | −323.63 | 6 | 659.26 | 659.37 | 0 | 0.63 | 0.0011 | ··· |
| ORD | −334.77 | 6 | 681.55 | 681.66 | 22.29 | 0 | 0 | ··· |
| ORDeq | −334.31 | 6 | 680.62 | 680.73 | 21.36 | 0 | 0 | ··· |
| ORDSYM | −342.93 | 3 | 691.86 | 691.89 | 32.52 | 0 | 0.0018 | ··· |
| ORDSYMeq | −341.81 | 3 | 689.61 | 689.64 | 30.27 | 0 | 0.0018 | ··· |
| ORDER | −344.71 | 1 | 691.41 | 691.42 | 32.05 | 0 | 0.0023 | ··· |

MP ancestral state reconstruction using ancestral.pars
(R:phangorn)
232_B. Perianth merism (3−state) (D2c), 70 steps

- trimerous
- tetramerous
- pentamerous

| Node | MP state(s) |
| --- | --- |
| Angiospermae | trimerous |
| Mesangiospermae | trimerous |
| Magnoliidae | trimerous |
| Monocotyledonae | trimerous |
| Eudicotyledonae | trimerous |
| Commelinidae | trimerous |
| Pentapetalae | pentamerous |
| Superasteridae | pentamerous |
| Asteridae | pentamerous |
| Lamiidae | pentamerous |
| Campanulidae | pentamerous |
| Superrosidae | pentamerous |
| Rosidae | pentamerous |
| Malvidae | pentamerous |
| Fabidae | pentamerous |

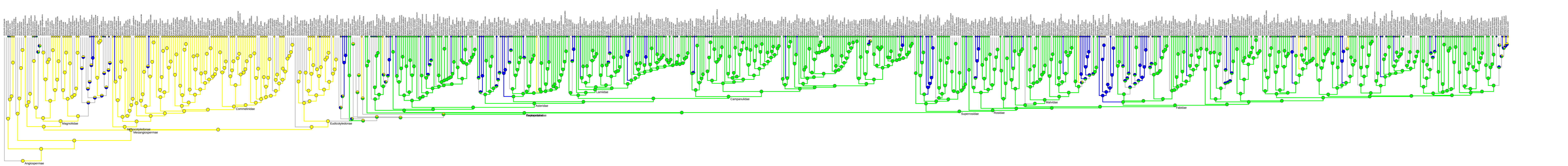

ML ancestral state reconstruction using rayDISC (R:corHMM)
232_B. Perianth merism (3−state) (D2c), SYMeq model

- trimerous
- tetramerous
- pentamerous

| Node | ML state | Prob |
|---|---|---|
| Angiospermae | trimerous | 1 |
| Mesangiospermae | trimerous | 1 |
| Magnoliidae | trimerous | 1 |
| Monocotyledonae | trimerous | 1 |
| Eudicotyledonae | trimerous | 0.9492 |
| Commelinidae | trimerous | 1 |
| Pentapetalae | pentamerous | 0.9966 |
| Superasteridae | pentamerous | 0.9966 |
| Asteridae | pentamerous | 0.9999 |
| Lamiidae | pentamerous | 1 |
| Campanulidae | pentamerous | 1 |
| Superrosidae | pentamerous | 1 |
| Rosidae | pentamerous | 1 |
| Malvidae | pentamerous | 1 |
| Fabidae | pentamerous | 1 |

| Model | LogL | Npar | AIC | AICc | DeltaAICc | w | q01 | ... |
|---|---|---|---|---|---|---|---|---|
| ARD | −254.79 | 6 | 521.58 | 521.69 | 5.12 | 0.02 | 7e−04 | ··· |
| ARDeq | −253.96 | 6 | 519.92 | 520.03 | 3.46 | 0.05 | 7e−04 | ··· |
| ER | −280.85 | 1 | 563.71 | 563.71 | 47.14 | 0 | 0.001 | ··· |
| SYM | −256.29 | 3 | 518.59 | 518.62 | 2.05 | 0.11 | 8e−04 | ··· |
| SYMeq* | −255.27 | 3 | 516.54 | 516.57 | 0 | 0.3 | 8e−04 | ··· |
| ORD | −255.18 | 4 | 518.36 | 518.41 | 1.84 | 0.12 | 9e−04 | ··· |
| ORDeq | −254.33 | 4 | 516.65 | 516.7 | 0.13 | 0.28 | 9e−04 | ··· |
| ORDSYM | −258.55 | 2 | 521.11 | 521.12 | 4.55 | 0.03 | 0.0014 | ··· |
| ORDSYMeq | −257.56 | 2 | 519.12 | 519.14 | 2.57 | 0.08 | 0.0014 | ··· |
| ORDER | −261.27 | 1 | 524.54 | 524.55 | 7.98 | 0.01 | 0.0023 | ··· |

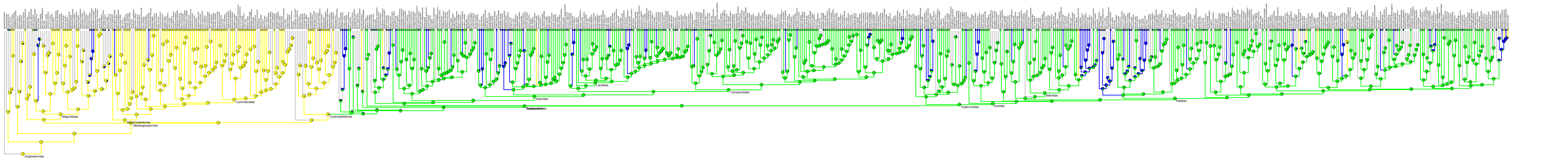

MP ancestral state reconstruction using ancestral.pars (R:phangorn)
234_A. Perianth differentiation (binary) (D2d), 64 steps

- undifferentiated
- differentiated

| Node | MP state(s) |
|---|---|
| Angiospermae | undifferentiated |
| Mesangiospermae | undifferentiated |
| Magnoliidae | undifferentiated / differentiated |
| Monocotyledonae | undifferentiated |
| Eudicotyledonae | undifferentiated |
| Commelinidae | undifferentiated |
| Pentapetalae | differentiated |
| Superasteridae | differentiated |
| Asteridae | differentiated |
| Lamiidae | differentiated |
| Campanulidae | differentiated |
| Superrosidae | differentiated |
| Rosidae | differentiated |
| Malvidae | differentiated |
| Fabidae | differentiated |

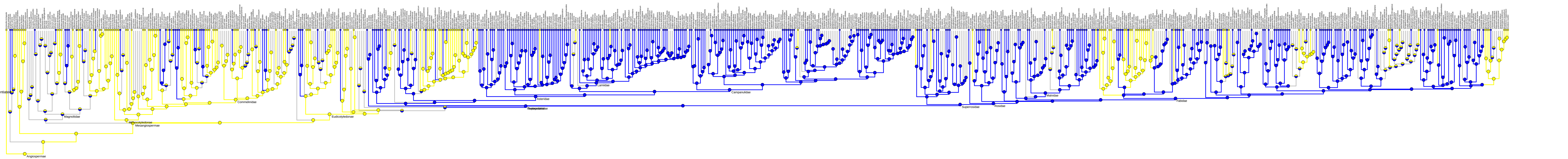

ML ancestral state reconstruction using rayDISC (R:corHMM)
234_A. Perianth differentiation (binary) (D2d), ARDeq model

- undifferentiated
- differentiated

| Node | ML state | Prob |
|---|---|---|
| Angiospermae | undifferentiated | 1 |
| Mesangiospermae | undifferentiated | 1 |
| Magnoliidae | undifferentiated | 0.9992 |
| Monocotyledonae | undifferentiated | 0.9999 |
| Eudicotyledonae | undifferentiated | 0.978 |
| Commelinidae | undifferentiated | 0.9998 |
| Pentapetalae | differentiated | 0.9441 |
| Superasteridae | differentiated | 0.9441 |
| Asteridae | differentiated | 0.999 |
| Lamiidae | differentiated | 0.9999 |
| Campanulidae | differentiated | 1 |
| Superrosidae | differentiated | 0.9949 |
| Rosidae | differentiated | 0.9973 |
| Malvidae | differentiated | 0.9993 |
| Fabidae | differentiated | 0.9898 |

| Model | LogL | Npar | AIC | AICc | DeltaAICc | w | q01 | q10 |
|---|---|---|---|---|---|---|---|---|
| ARD | −199.02 | 2 | 402.04 | 402.06 | 1.3 | 0.34 | 0.0041 | 0.0014 |
| ARDeq** | −198.37 | 2 | 400.74 | 400.75 | 0 | 0.66 | 0.0041 | 0.0014 |
| ER | −207.02 | 1 | 416.04 | 416.05 | 15.29 | 0 | 0.0024 | 0.0024 |
| UNI01 | −231.24 | 1 | 464.48 | 464.48 | 63.73 | 0 | 0.0082 | |
| UNI10 | −231.65 | 1 | 465.3 | 465.3 | 64.55 | 0 | | 0.0032 |

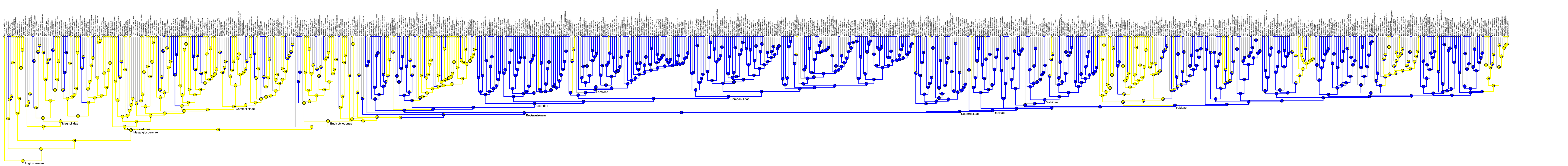

MP ancestral state reconstruction using ancestral.pars
(R:phangorn)
204_A. Fusion of perianth (D2c), 78 steps

- free (<5%)
- fused (>5%)

| Node | MP state(s) |
|---|---|
| Angiospermae | free (<5%) |
| Mesangiospermae | free (<5%) |
| Magnoliidae | free (<5%) |
| Monocotyledonae | free (<5%) |
| Eudicotyledonae | free (<5%) |
| Commelinidae | free (<5%) |
| Pentapetalae | free (<5%) / fused (>5%) |
| Superasteridae | free (<5%) / fused (>5%) |
| Asteridae | free (<5%) |
| Lamiidae | free (<5%) / fused (>5%) |
| Campanulidae | free (<5%) / fused (>5%) |
| Superrosidae | free (<5%) / fused (>5%) |
| Rosidae | free (<5%) / fused (>5%) |
| Malvidae | free (<5%) |
| Fabidae | free (<5%) |

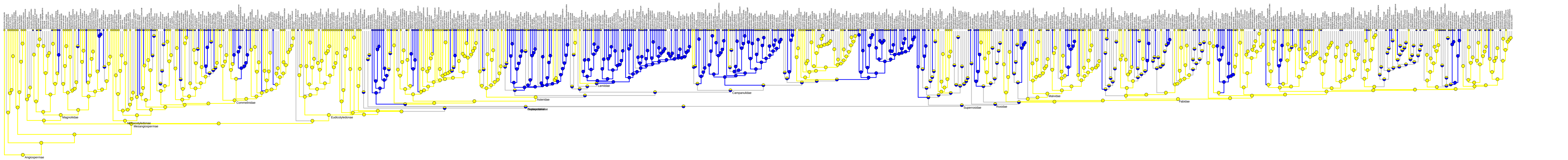

ML ancestral state reconstruction using rayDISC (R:corHMM)
204_A. Fusion of perianth (D2c), ER model

- free (<5%)
- fused (>5%)

| Node | ML state | Prob |
| --- | --- | --- |
| Angiospermae | free (<5%) | 0.9207 |
| Mesangiospermae | free (<5%) | 0.9999 |
| Magnoliidae | free (<5%) | 0.9985 |
| Monocotyledonae | free (<5%) | 0.9995 |
| Eudicotyledonae | free (<5%) | 0.9998 |
| Commelinidae | free (<5%) | 0.9962 |
| Pentapetalae | free (<5%) | 0.9922 |
| Superasteridae | free (<5%) | 0.9922 |
| Asteridae | free (<5%) | 0.7228 |
| Lamiidae | fused (>5%) | 0.9849 |
| Campanulidae | fused (>5%) | 0.9809 |
| Superrosidae | free (<5%) | 0.9925 |
| Rosidae | free (<5%) | 0.9933 |
| Malvidae | free (<5%) | 0.9968 |
| Fabidae | free (<5%) | 0.9997 |

| Model | LogL | Npar | AIC | AICc | DeltaAICc | w | q01 | q10 |
| --- | --- | --- | --- | --- | --- | --- | --- | --- |
| ARD | −225.91 | 2 | 455.82 | 455.84 | 1.79 | 0.19 | 0.0041 | 0.0037 |
| ARDeq | −225.35 | 2 | 454.71 | 454.72 | 0.67 | 0.34 | 0.0041 | 0.0036 |
| ER* | −226.02 | 1 | 454.04 | 454.05 | 0 | 0.47 | 0.004 | 0.004 |
| UNI01 | −241.48 | 1 | 484.96 | 484.97 | 30.92 | 0 | 0.0046 | |
| UNI10 | −235.58 | 1 | 473.15 | 473.16 | 19.11 | 0 | | 0.0076 |

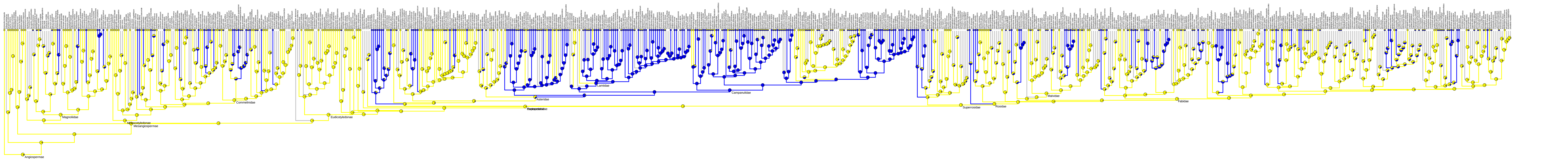

MP ancestral state reconstruction using ancestral.pars
(R:phangorn)
207_A. Symmetry of perianth (binary) (D2d), 55 steps

- actinomorphic
- zygomorphic

| Node | MP state(s) |
|---|---|
| Angiospermae | actinomorphic |
| Mesangiospermae | actinomorphic |
| Magnoliidae | actinomorphic |
| Monocotyledonae | actinomorphic |
| Eudicotyledonae | actinomorphic |
| Commelinidae | actinomorphic |
| Pentapetalae | actinomorphic |
| Superasteridae | actinomorphic |
| Asteridae | actinomorphic |
| Lamiidae | actinomorphic |
| Campanulidae | actinomorphic |
| Superrosidae | actinomorphic |
| Rosidae | actinomorphic |
| Malvidae | actinomorphic |
| Fabidae | actinomorphic |

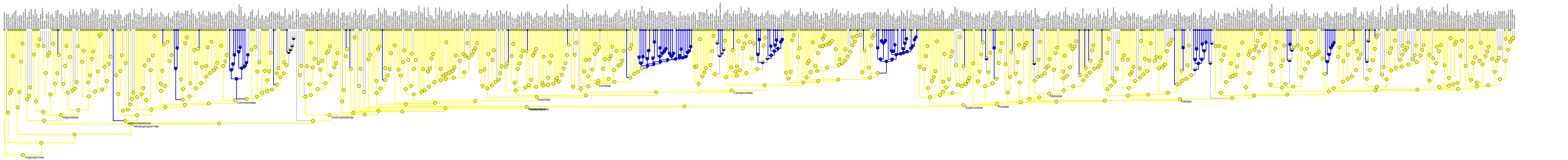

ML ancestral state reconstruction using rayDISC (R:corHMM)
207_A. Symmetry of perianth (binary) (D2d), ARDeq model

- actinomorphic
- zygomorphic

| Node | ML state | Prob |
|---|---|---|
| Angiospermae | actinomorphic | 0.9981 |
| Mesangiospermae | actinomorphic | 0.9999 |
| Magnoliidae | actinomorphic | 0.9997 |
| Monocotyledonae | actinomorphic | 0.9985 |
| Eudicotyledonae | actinomorphic | 0.9996 |
| Commelinidae | actinomorphic | 0.9961 |
| Pentapetalae | actinomorphic | 1 |
| Superasteridae | actinomorphic | 1 |
| Asteridae | actinomorphic | 1 |
| Lamiidae | actinomorphic | 0.9985 |
| Campanulidae | actinomorphic | 1 |
| Superrosidae | actinomorphic | 1 |
| Rosidae | actinomorphic | 1 |
| Malvidae | actinomorphic | 0.9998 |
| Fabidae | actinomorphic | 0.9999 |

| Model | LogL | Npar | AIC | AICc | DeltaAICc | w | q01 | q10 |
|---|---|---|---|---|---|---|---|---|
| ARD | −210.26 | 2 | 424.51 | 424.53 | 0.94 | 0.38 | 0.0013 | 0.0057 |
| ARDeq** | −209.79 | 2 | 423.57 | 423.59 | 0 | 0.62 | 0.0013 | 0.0056 |
| ER | −218.48 | 1 | 438.97 | 438.97 | 15.38 | 0 | 0.0014 | 0.0014 |
| UNI01 | −263.34 | 1 | 528.69 | 528.69 | 105.11 | 0 | 0.0018 | |
| UNI10 | −219.81 | 1 | 441.62 | 441.62 | 18.03 | 0 | | 0.0155 |

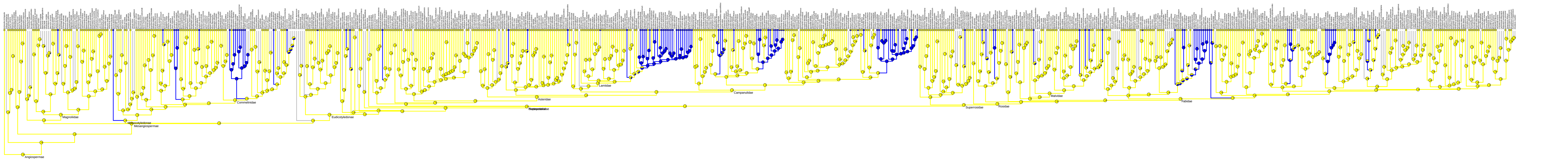

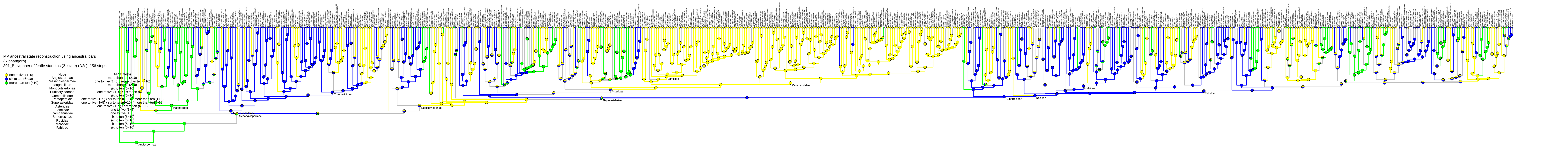
MP ancestral state reconstruction using ancestral.pars
(R:phangorn)
301_B. Number of fertile stamens (3−state) (D2c), 156 steps
one to five (1−5)
six to ten (6−10)
more than ten (>10)
Node
MP state(s)
Angiospermae more than ten (>10)
Mesangiospermae one to five (1−5) / more than ten (>10)
Magnoliidae more than ten (>10)
Monocotyledonae six to ten (6−10)
Eudicotyledonae one to five (1−5) / six to ten (6−10)
Commelinidae six to ten (6−10)
Pentapetalae one to five (1−5) / six to ten (6−10) / more than ten (>10)
Superasteridae one to five (1−5) / six to ten (6−10) / more than ten (>10)
Asteridae one to five (1−5) / six to ten (6−10)
Lamiidae one to five (1−5)
Campanulidae one to five (1−5)
Superrosidae six to ten (6−10)
Rosidae six to ten (6−10)
Malvidae six to ten (6−10)
Fabidae six to ten (6−10)
Magnoliidae
Angiospermae
Mesangiospermae
Commelinidae
Eudicotyledonae
Asteridae
Lamiidae
Campanulidae
Superrosidae
Rosidae
Malvidae
Fabidae

ML ancestral state reconstruction using rayDISC (R:corHMM)
301_B. Number of fertile stamens (3−state) (D2c), ARDeq model

- one to five (1−5)
- six to ten (6−10)
- more than ten (>10)

| Node | ML state | Prob |
|---|---|---|
| Angiospermae | more than ten (>10) | 1 |
| Mesangiospermae | more than ten (>10) | 0.9995 |
| Magnoliidae | more than ten (>10) | 0.9997 |
| Monocotyledonae | more than ten (>10) | 0.634 |
| Eudicotyledonae | more than ten (>10) | 0.9996 |
| Commelinidae | six to ten (6−10) | 0.9997 |
| Pentapetalae | more than ten (>10) | 1 |
| Superasteridae | more than ten (>10) | 1 |
| Asteridae | more than ten (>10) | 0.9988 |
| Lamiidae | one to five (1−5) | 0.9997 |
| Campanulidae | one to five (1−5) | 0.9971 |
| Superrosidae | more than ten (>10) | 1 |
| Rosidae | more than ten (>10) | 0.9998 |
| Malvidae | more than ten (>10) | 0.9096 |
| Fabidae | more than ten (>10) | 0.9999 |

| Model | LogL | Npar | AIC | AICc | DeltaAICc | w | q01 | ··· |
|---|---|---|---|---|---|---|---|---|
| ARD | −492.9 | 6 | 997.8 | 997.91 | 2.15 | 0.25 | 4e−04 | ··· |
| ARDeq** | −491.83 | 6 | 995.65 | 995.76 | 0 | 0.75 | 4e−04 | ··· |
| ER | −533.13 | 1 | 1068.25 | 1068.26 | 72.5 | 0 | 0.0025 | ··· |
| SYM | −527.27 | 3 | 1060.55 | 1060.58 | 64.82 | 0 | 0.0035 | ··· |
| SYMeq | −526.96 | 3 | 1059.92 | 1059.95 | 64.19 | 0 | 0.0035 | ··· |
| ORD | −506.84 | 4 | 1021.67 | 1021.72 | 25.96 | 0 | 7e−04 | ··· |
| ORDeq | −506.26 | 4 | 1020.52 | 1020.57 | 24.81 | 0 | 7e−04 | ··· |
| ORDSYM | −531.45 | 2 | 1066.89 | 1066.91 | 71.15 | 0 | 0.0041 | ··· |
| ORDSYMeq | −531.09 | 2 | 1066.19 | 1066.2 | 70.44 | 0 | 0.0041 | ··· |
| ORDER | −531.46 | 1 | 1064.92 | 1064.92 | 69.16 | 0 | 0.0041 | ··· |

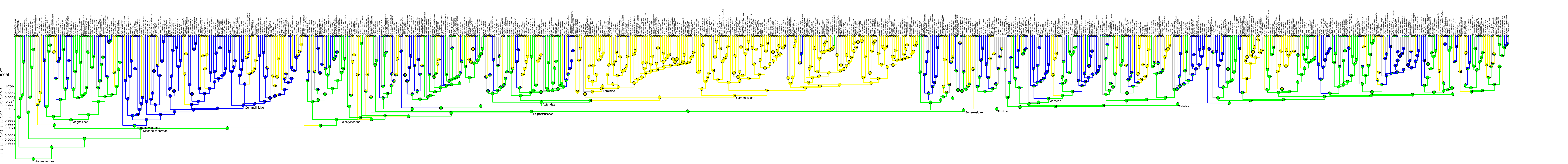

MP ancestral state reconstruction using ancestral.pars
(R:phangorn)
301_C. Number of fertile stamens (binary) (D2c), 93 steps

- one to six (1−6)
- more than six (>6)

| Node | MP state(s) |
|---|---|
| Angiospermae | more than six (>6) |
| Mesangiospermae | one to six (1−6) / more than six (>6) |
| Magnoliidae | one to six (1−6) / more than six (>6) |
| Monocotyledonae | one to six (1−6) |
| Eudicotyledonae | one to six (1−6) |
| Commelinidae | one to six (1−6) |
| Pentapetalae | more than six (>6) |
| Superasteridae | more than six (>6) |
| Asteridae | more than six (>6) |
| Lamiidae | one to six (1−6) |
| Campanulidae | one to six (1−6) |
| Superrosidae | more than six (>6) |
| Rosidae | more than six (>6) |
| Malvidae | more than six (>6) |
| Fabidae | more than six (>6) |

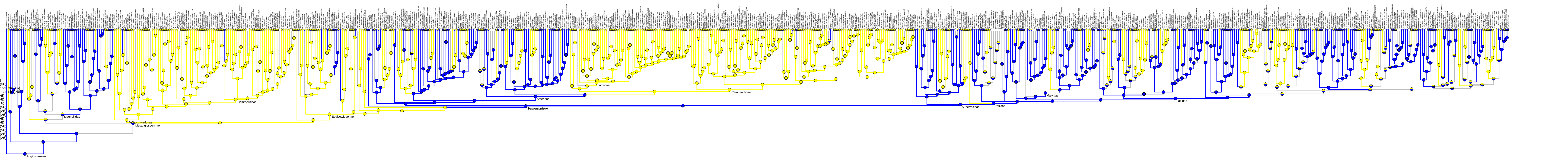

ML ancestral state reconstruction using rayDISC (R:corHMM)
301_C. Number of fertile stamens (binary) (D2c), ARDeq model

- one to six (1−6)
- more than six (>6)

| Node | ML state | Prob |
|---|---|---|
| Angiospermae | more than six (>6) | 1 |
| Mesangiospermae | more than six (>6) | 0.999 |
| Magnoliidae | more than six (>6) | 1 |
| Monocotyledonae | more than six (>6) | 0.6481 |
| Eudicotyledonae | more than six (>6) | 0.9973 |
| Commelinidae | one to six (1−6) | 0.9999 |
| Pentapetalae | more than six (>6) | 1 |
| Superasteridae | more than six (>6) | 1 |
| Asteridae | more than six (>6) | 0.9999 |
| Lamiidae | one to six (1−6) | 0.9993 |
| Campanulidae | one to six (1−6) | 0.9951 |
| Superrosidae | more than six (>6) | 1 |
| Rosidae | more than six (>6) | 0.9999 |
| Malvidae | more than six (>6) | 1 |
| Fabidae | more than six (>6) | 1 |

| Model | LogL | Npar | AIC | AICc | DeltaAICc | w | q01 | q10 |
|---|---|---|---|---|---|---|---|---|
| ARD | −288.42 | 2 | 580.85 | 580.86 | 1.38 | 0.33 | 5e−04 | 0.0054 |
| ARDeq** | −287.73 | 2 | 579.47 | 579.48 | 0 | 0.67 | 5e−04 | 0.0054 |
| ER | −320.94 | 1 | 643.89 | 643.89 | 64.41 | 0 | 0.0029 | 0.0029 |
| UNI01 | −364.2 | 1 | 730.4 | 730.41 | 150.92 | 0 | 0.0043 | |
| UNI10 | −325.34 | 1 | 652.67 | 652.68 | 73.19 | 0 | | 0.0068 |

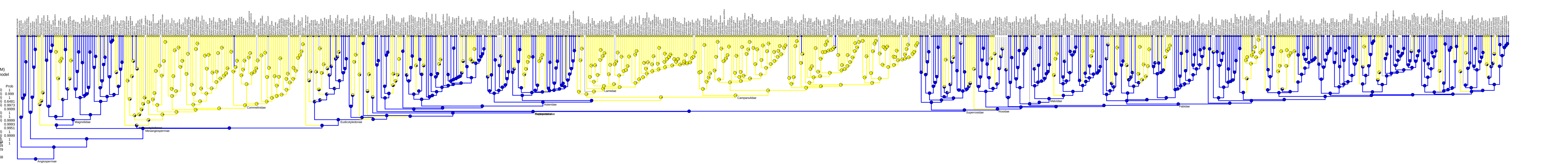

MP ancestral state reconstruction using ancestral.pars
(R:phangorn)
330_A. Androecium structural phyllotaxy (binary) (D2d), 12 steps

- whorled
- spiral

| Node | MP state(s) |
|---|---|
| Angiospermae | whorled / spiral |
| Mesangiospermae | whorled / spiral |
| Magnoliidae | whorled / spiral |
| Monocotyledonae | whorled |
| Eudicotyledonae | whorled |
| Commelinidae | whorled |
| Pentapetalae | whorled |
| Superasteridae | whorled |
| Asteridae | whorled |
| Lamiidae | whorled |
| Campanulidae | whorled |
| Superrosidae | whorled |
| Rosidae | whorled |
| Malvidae | whorled |
| Fabidae | whorled |

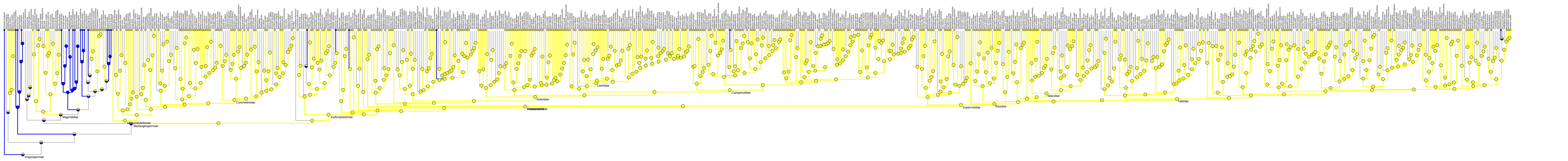

ML ancestral state reconstruction using rayDISC (R:corHMM)
330_A. Androecium structural phyllotaxy (binary) (D2d), ARD model

- whorled
- spiral

| Node | ML state | Prob |
|---|---|---|
| Angiospermae | spiral | 0.9992 |
| Mesangiospermae | spiral | 0.9883 |
| Magnoliidae | spiral | 0.9898 |
| Monocotyledonae | spiral | 0.5712 |
| Eudicotyledonae | spiral | 0.8962 |
| Commelinidae | whorled | 0.9999 |
| Pentapetalae | whorled | 0.9927 |
| Superasteridae | whorled | 0.9927 |
| Asteridae | whorled | 1 |
| Lamiidae | whorled | 1 |
| Campanulidae | whorled | 1 |
| Superrosidae | whorled | 0.9998 |
| Rosidae | whorled | 0.9999 |
| Malvidae | whorled | 1 |
| Fabidae | whorled | 1 |

| Model | LogL | Npar | AIC | AICc | DeltaAICc | w | q01 | q10 |
|---|---|---|---|---|---|---|---|---|
| ARD** | −54.27 | 2 | 112.53 | 112.55 | 0 | 0.92 | 1e−04 | 0.007 |
| ARDeq | −57.95 | 2 | 119.9 | 119.92 | 7.37 | 0.02 | 4e−04 | 0 |
| ER | −58.67 | 1 | 119.34 | 119.34 | 6.8 | 0.03 | 4e−04 | 4e−04 |
| UNI01 | −58.64 | 1 | 119.29 | 119.29 | 6.75 | 0.03 | 4e−04 | |
| UNI10 | −80.64 | 1 | 163.29 | 163.29 | 50.75 | 0 | | 0.0186 |

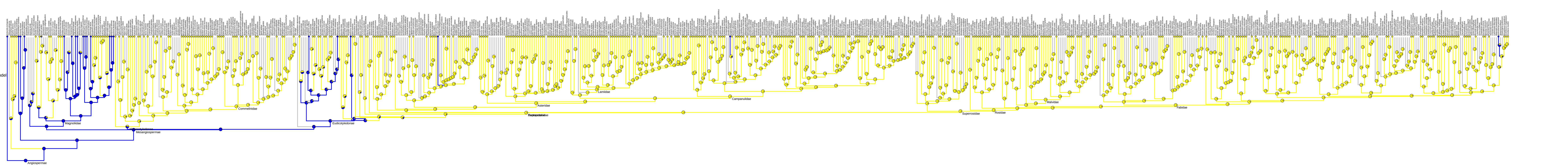

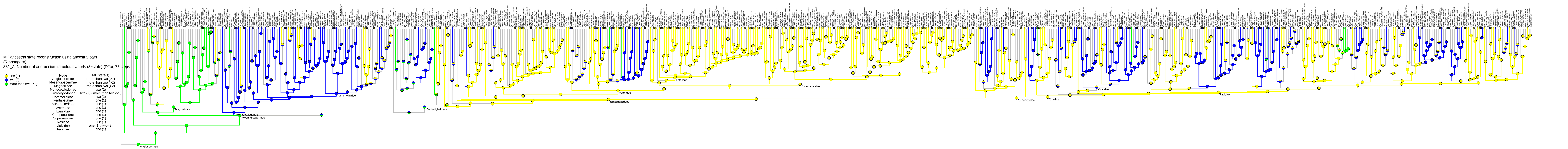

MP ancestral state reconstruction using ancestral.pars
(R:phangorn)
331_A. Number of androecium structural whorls (3−state) (D2c), 75 steps
one (1)
two (2)
more than two (>2)
Node
MP state(s)
Angiospermae
more than two (>2)
Mesangiospermae
more than two (>2)
Magnoliidae
more than two (>2)
Monocotyledonae
two (2)
Eudicotyledonae
two (2) / more than two (>2)
Commelinidae
two (2)
Pentapetalae
one (1)
Superasteridae
one (1)
Asteridae
one (1)
Lamiidae
one (1)
Campanulidae
one (1)
Superrosidae
one (1)
Rosidae
one (1)
Malvidae
one (1) / two (2)
Fabidae
one (1)

ML ancestral state reconstruction using rayDISC (R:corHMM)
331_A. Number of androecium structural whorls (3−state) (D2c), ORDeq model

- one (1)
- two (2)
- more than two (>2)

| Node | ML state | Prob |
|---|---|---|
| Angiospermae | more than two (>2) | 1 |
| Mesangiospermae | more than two (>2) | 0.9911 |
| Magnoliidae | more than two (>2) | 0.9909 |
| Monocotyledonae | more than two (>2) | 0.956 |
| Eudicotyledonae | more than two (>2) | 0.8422 |
| Commelinidae | two (2) | 0.9353 |
| Pentapetalae | two (2) | 0.969 |
| Superasteridae | two (2) | 0.969 |
| Asteridae | two (2) | 0.9999 |
| Lamiidae | one (1) | 0.999 |
| Campanulidae | one (1) | 0.993 |
| Superrosidae | two (2) | 0.9981 |
| Rosidae | two (2) | 0.9989 |
| Malvidae | two (2) | 0.9996 |
| Fabidae | two (2) | 1 |

| Model | LogL | Npar | AIC | AICc | DeltaAICc | w | c01 | ··· |
|---|---|---|---|---|---|---|---|---|
| ARD | −232.39 | 6 | 476.78 | 476.88 | 3.72 | 0.08 | 0 | ··· |
| ARDeq | −231.35 | 6 | 474.69 | 474.8 | 1.64 | 0.23 | 0 | ··· |
| ER | −278.37 | 1 | 558.74 | 558.75 | 85.59 | 0 | 0.0016 | ··· |
| SYM | −253.62 | 3 | 513.24 | 513.27 | 40.11 | 0 | 0.0033 | ··· |
| SYMeq | −252.95 | 3 | 511.91 | 511.94 | 38.78 | 0 | 0.0033 | ··· |
| ORD | −233.59 | 4 | 475.19 | 475.24 | 2.08 | 0.18 | 0 | ··· |
| ORDeq** | −232.55 | 4 | 473.11 | 473.16 | 0 | 0.51 | 0 | ··· |
| ORDSYM | −254.21 | 2 | 512.42 | 512.43 | 39.27 | 0 | 0.0034 | ··· |
| ORDSYMeq | −253.49 | 2 | 510.99 | 511 | 37.84 | 0 | 0.0033 | ··· |
| ORDER | −259.57 | 1 | 521.15 | 521.15 | 47.99 | 0 | 0.0027 | ··· |

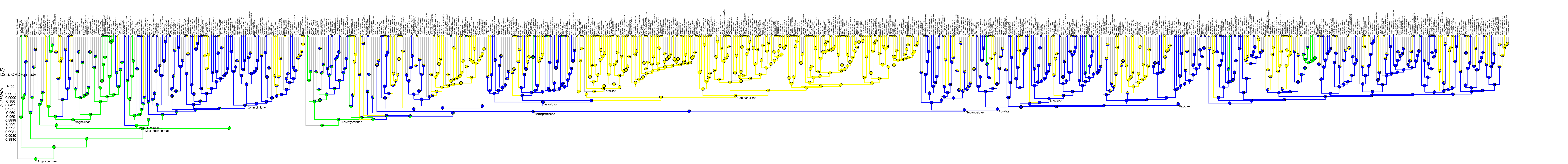

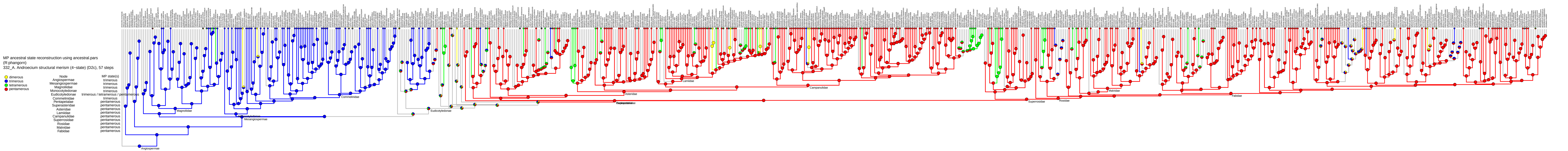
MP ancestral state reconstruction using ancestral.pars
(R:phangorn)
332_A. Androecium structural merism (4−state) (D2c), 57 steps
dimerous
trimerous
tetramerous
pentamerous
Node
Angiospermae
Mesangiospermae
Magnoliidae
Monocotyledonae
Eudicotyledonae
Commelinidae
Pentapetalae
Superasteridae
Asteridae
Lamiidae
Campanulidae
Superrosidae
Rosidae
Malvidae
Fabidae
MP state(s)
trimerous
trimerous
trimerous
trimerous
trimerous / tetramerous / pentamerous
trimerous
pentamerous
pentamerous
pentamerous
pentamerous
pentamerous
pentamerous
pentamerous
pentamerous
pentamerous

ML ancestral state reconstruction using rayDISC (R:corHMM)
332_A. Androecium structural merism (4−state) (D2c), ORDeq model

- dimerous
- trimerous
- tetramerous
- pentamerous

| Node | ML state | Prob |
|---|---|---|
| Angiospermae | tetramerous | 0.9889 |
| Mesangiospermae | tetramerous | 0.9974 |
| Magnoliidae | tetramerous | 0.9986 |
| Monocotyledonae | tetramerous | 0.6313 |
| Eudicotyledonae | tetramerous | 0.861 |
| Commelinidae | trimerous | 0.9662 |
| Pentapetalae | pentamerous | 0.9917 |
| Superasteridae | pentamerous | 0.9917 |
| Asteridae | pentamerous | 0.9999 |
| Lamiidae | pentamerous | 1 |
| Campanulidae | pentamerous | 1 |
| Superrosidae | pentamerous | 1 |
| Rosidae | pentamerous | 1 |
| Malvidae | pentamerous | 1 |
| Fabidae | pentamerous | 1 |

| Model | LogL | Npar | AIC | AICc | DeltaAICc | w | q01 | |
|---|---|---|---|---|---|---|---|---|
| ARD | −222.05 | 12 | 468.11 | 468.51 | 289.11 | 0 | 0.0082 | ··· |
| ARDeq | −221.23 | 12 | 466.47 | 466.87 | 287.46 | 0 | 0.007 | ··· |
| ER | −236.89 | 1 | 475.79 | 475.79 | 296.39 | 0 | 8e−04 | ··· |
| SYM | −224.29 | 6 | 460.58 | 460.69 | 281.29 | 0 | 5e−04 | ··· |
| SYMeq | −223.05 | 6 | 458.1 | 458.2 | 278.8 | 0 | 5e−04 | ··· |
| ORD | −84.47 | 6 | 180.94 | 181.05 | 1.65 | 0.31 | 76.3945 | ··· |
| ORDeq** | −83.65 | 6 | 179.3 | 179.4 | 0 | 0.69 | 76.0406 | ··· |
| ORDSYM | −258.07 | 3 | 522.13 | 522.16 | 342.76 | 0 | 0.0019 | ··· |
| ORDSYMeq | −257.18 | 3 | 520.36 | 520.39 | 340.98 | 0 | 0.0019 | ··· |
| ORDER | −258.96 | 1 | 519.92 | 519.93 | 340.52 | 0 | 0.0029 | ··· |

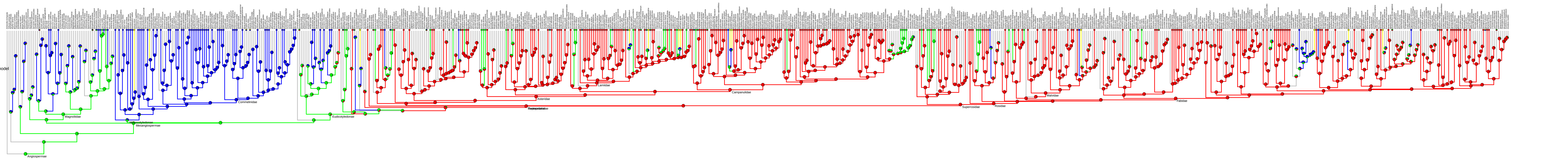

MP ancestral state reconstruction using ancestral.pars
(R:phangorn)
332_B. Androecium structural merism (3−state) (D2c), 48 steps

- trimerous
- tetramerous
- pentamerous

| Node | MP state(s) |
|---|---|
| Angiospermae | trimerous |
| Mesangiospermae | trimerous |
| Magnoliidae | trimerous |
| Monocotyledonae | trimerous |
| Eudicotyledonae | trimerous / tetramerous / pentamerous |
| Commelinidae | trimerous |
| Pentapetalae | pentamerous |
| Superasteridae | pentamerous |
| Asteridae | pentamerous |
| Lamiidae | pentamerous |
| Campanulidae | pentamerous |
| Superrosidae | pentamerous |
| Rosidae | pentamerous |
| Malvidae | pentamerous |
| Fabidae | pentamerous |

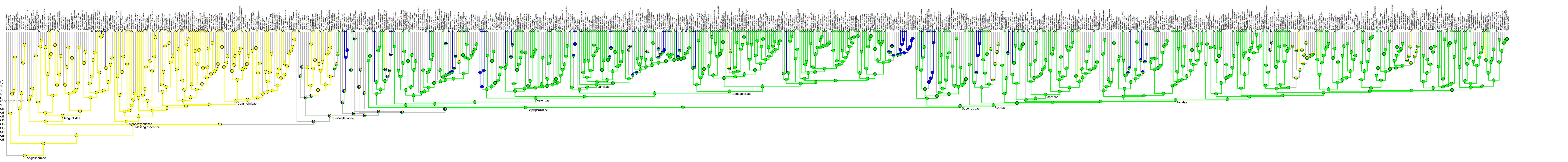

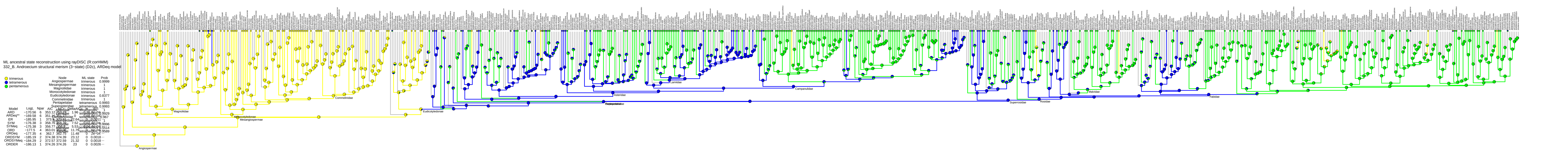
ML ancestral state reconstruction using rayDISC (R:corHMM)
332_B. Androecium structural merism (3−state) (D2c), ARDeq model
trimerous
tetramerous
pentamerous
Node | ML state | Prob
Angiospermae | trimerous | 0.9999
Mesangiospermae | trimerous | 1
Magnoliidae | trimerous | 1
Monocotyledonae | trimerous | 1
Eudicotyledonae | trimerous | 0.8377
Commelinidae | trimerous | 1
Pentapetalae | tetramerous | 0.9993
Superasteridae | tetramerous | 0.9993
Asteridae | tetramerous | 1
Lamiidae | tetramerous | 0.9929
Campanulidae | tetramerous | 0.967
Superrosidae | tetramerous | 1
Rosidae | tetramerous | 0.9996
Malvidae | pentamerous | 0.5514
Fabidae | tetramerous | 0.9589
Model | LogL | Npar | AIC | AICc | DeltaAICc | w | q01 | ···
ARD | −170.56 | 6 | 353.12 | 353.23 | 1.96 | 0.26 | 8e−04 | ···
ARDeq** | −169.58 | 6 | 351.16 | 351.27 | 0 | 0.68 | 8e−04 | ···
ER | −185.95 | 1 | 373.9 | 373.91 | 22.64 | 0 | 0.0011 | ···
SYM | −176.38 | 3 | 358.76 | 358.79 | 7.52 | 0.02 | 4e−04 | ···
SYMeq | −175.38 | 3 | 356.77 | 356.8 | 5.53 | 0.04 | 4e−04 | ···
ORD | −177.5 | 4 | 363.01 | 363.06 | 11.79 | 0 | 6e−04 | ···
ORDeq | −177.35 | 4 | 362.7 | 362.75 | 11.48 | 0 | 2e−04 | ···
ORDSYM | −185.19 | 2 | 374.38 | 374.39 | 23.12 | 0 | 0.0018 | ···
ORDSYMeq | −184.29 | 2 | 372.57 | 372.59 | 21.32 | 0 | 0.0018 | ···
ORDER | −186.13 | 1 | 374.26 | 374.26 | 23 | 0 | 0.0026 | ···
Angiospermae
Magnoliidae
Monocotyledonae
Mesangiospermae
Commelinidae
Eudicotyledonae
Asteridae
Lamiidae
Campanulidae
Superasteridae
Pentapetalae
Superrosidae
Rosidae
Malvidae
Fabidae

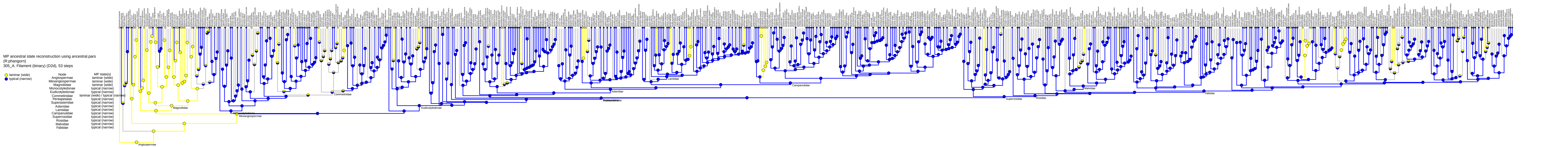
MP ancestral state reconstruction using ancestral.pars
(R:phangorn)
305_A. Filament (binary) (D2d), 53 steps
laminar (wide)
typical (narrow)
Node
MP state(s)
Angiospermae laminar (wide)
Mesangiospermae laminar (wide)
Magnoliidae laminar (wide)
Monocotyledonae typical (narrow)
Eudicotyledonae typical (narrow)
Commelinidae laminar (wide) / typical (narrow)
Pentapetalae typical (narrow)
Superasteridae typical (narrow)
Asteridae typical (narrow)
Lamiidae typical (narrow)
Campanulidae typical (narrow)
Superrosidae typical (narrow)
Rosidae typical (narrow)
Malvidae typical (narrow)
Fabidae typical (narrow)

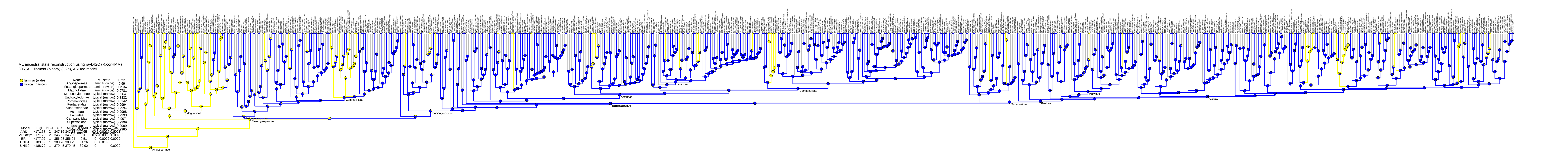
ML ancestral state reconstruction using rayDISC (R:corHMM)
305_A. Filament (binary) (D2d), ARDeq model
laminar (wide)
typical (narrow)
Node | ML state | Prob
Angiospermae | laminar (wide) | 0.99
Mesangiospermae | laminar (wide) | 0.7934
Magnoliidae | laminar (wide) | 0.9781
Monocotyledonae | typical (narrow) | 0.564
Eudicotyledonae | typical (narrow) | 0.8832
Commelinidae | typical (narrow) | 0.8142
Pentapetalae | typical (narrow) | 0.9994
Superasteridae | typical (narrow) | 0.9994
Asteridae | typical (narrow) | 0.9998
Lamiidae | typical (narrow) | 0.9993
Campanulidae | typical (narrow) | 0.997
Superrosidae | typical (narrow) | 0.9999
Rosidae | typical (narrow) | 0.9999
Malvidae | typical (narrow) | 0.9985
Fabidae | typical (narrow) | 1
Model | LogL | Npar | AIC | AICc | DeltaAICc | w | q01 | q10
ARD | −171.58 | 2 | 347.16 | 347.18 | 0.65 | 0.42 | 0.0066 | 0.0021
ARDeq** | −171.26 | 2 | 346.52 | 346.53 | 0 | 0.58 | 0.0068 | 0.002
ER | −177.02 | 1 | 356.03 | 356.04 | 9.51 | 0 | 0.0022 | 0.0022
UNI01 | −189.39 | 1 | 380.78 | 380.79 | 34.26 | 0 | 0.0135 |
UNI10 | −188.72 | 1 | 379.45 | 379.45 | 32.92 | 0 | | 0.0022
Magnoliidae
Monocotyledonae
Mesangiospermae
Angiospermae
Commelinidae
Eudicotyledonae
Asteridae
Lamiidae
Campanulidae
Superrosidae
Rosidae
Malvidae
Fabidae

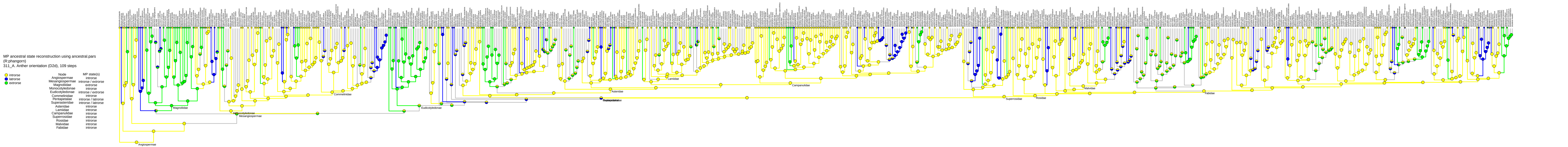
MP ancestral state reconstruction using ancestral.pars
(R:phangorn)
311_A. Anther orientation (D2d), 109 steps
introrse
latrorse
extrorse
Node
MP state(s)
Angiospermae
introrse
Mesangiospermae
introrse / extrorse
Magnoliidae
extrorse
Monocotyledonae
introrse
Eudicotyledonae
introrse / extrorse
Commelinidae
introrse
Pentapetalae
introrse / latrorse
Superasteridae
introrse / latrorse
Asteridae
introrse
Lamiidae
introrse
Campanulidae
introrse
Superrosidae
introrse
Rosidae
introrse
Malvidae
introrse
Fabidae
introrse
Angiospermae
Magnoliidae
Mesangiospermae
Commelinidae
Eudicotyledonae
Asteridae
Lamiidae
Campanulidae
Superrosidae
Rosidae
Malvidae
Fabidae

ML ancestral state reconstruction using rayDISC (R:corHMM)
311_A. Anther orientation (D2d), SYMeq model

- introrse
- latrorse
- extrorse

| Node | ML state | Prob |
|---|---|---|
| Angiospermae | introrse | 0.9982 |
| Mesangiospermae | introrse | 0.9699 |
| Magnoliidae | extrorse | 0.8936 |
| Monocotyledonae | introrse | 0.9787 |
| Eudicotyledonae | introrse | 0.9687 |
| Commelinidae | introrse | 0.9941 |
| Pentapetalae | introrse | 0.9993 |
| Superasteridae | introrse | 0.9993 |
| Asteridae | introrse | 0.9993 |
| Lamiidae | introrse | 0.9998 |
| Campanulidae | introrse | 1 |
| Superrosidae | introrse | 1 |
| Rosidae | introrse | 1 |
| Malvidae | introrse | 0.9998 |
| Fabidae | introrse | 0.9997 |

| Model | LogL | Npar | AIC | AICc | DeltaAICc | w | q01 | ··· |
|---|---|---|---|---|---|---|---|---|
| ARD | −343.57 | 6 | 699.14 | 699.25 | 2.21 | 0.1 | 0.0036 | ··· |
| ARDeq | −343.24 | 6 | 698.48 | 698.59 | 1.56 | 0.14 | 0.0035 | ··· |
| ER | −347.52 | 1 | 697.03 | 697.04 | 0.01 | 0.31 | 0.0026 | ··· |
| SYM | −346.28 | 3 | 698.56 | 698.59 | 1.56 | 0.14 | 0.0029 | ··· |
| SYMeq* | −345.5 | 3 | 697 | 697.03 | 0 | 0.31 | 0.0029 | ··· |

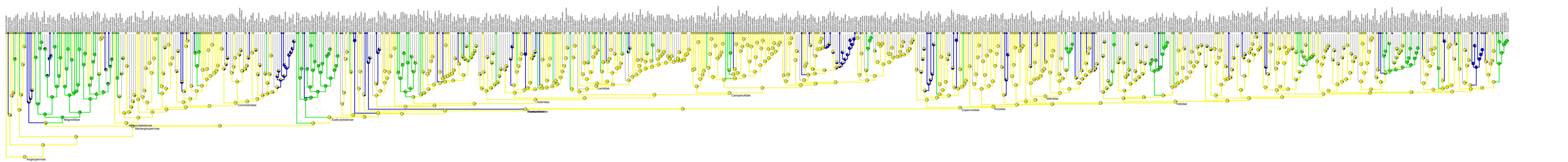

MP ancestral state reconstruction using ancestral.pars
(R:phangorn)
312_A. Anther attachment (binary) (D2d), 97 steps

- basifixed
- dorsifixed

| Node | MP state(s) |
|---|---|
| Angiospermae | basifixed |
| Mesangiospermae | basifixed |
| Magnoliidae | basifixed |
| Monocotyledonae | basifixed |
| Eudicotyledonae | basifixed |
| Commelinidae | basifixed |
| Pentapetalae | basifixed |
| Superasteridae | basifixed |
| Asteridae | basifixed |
| Lamiidae | basifixed |
| Campanulidae | basifixed / dorsifixed |
| Superrosidae | basifixed |
| Rosidae | dorsifixed |
| Malvidae | dorsifixed |
| Fabidae | dorsifixed |

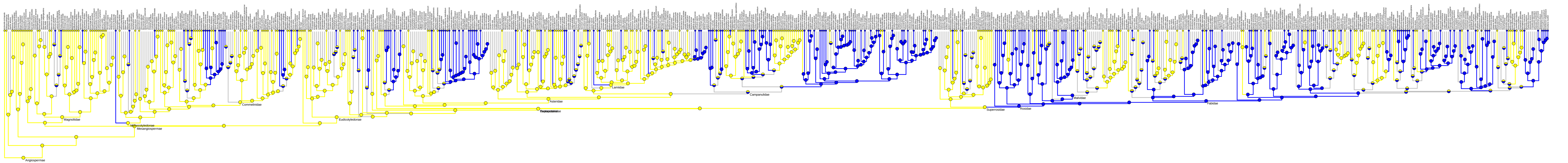

ML ancestral state reconstruction using rayDISC (R:corHMM)
312_A. Anther attachment (binary) (D2d), ARDeq model

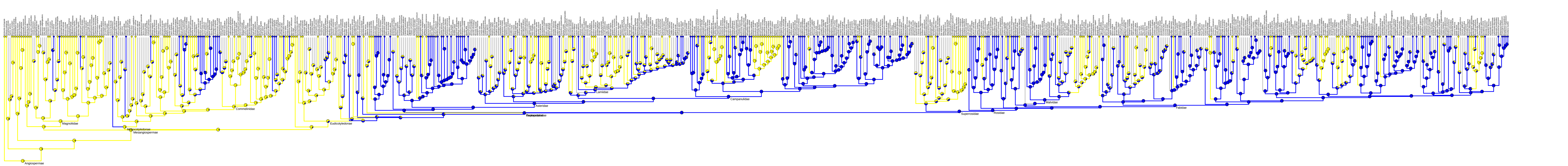


| Node | ML state | Prob |
|---|---|---|
| Angiospermae | basifixed | 0.9763 |
| Mesangiospermae | basifixed | 0.9547 |
| Magnoliidae | basifixed | 0.9783 |
| Monocotyledonae | basifixed | 0.9411 |
| Eudicotyledonae | basifixed | 0.7081 |
| Commelinidae | basifixed | 0.9359 |
| Pentapetalae | dorsifixed | 0.9171 |
| Superasteridae | dorsifixed | 0.9171 |
| Asteridae | dorsifixed | 0.9381 |
| Lamiidae | dorsifixed | 0.6477 |
| Campanulidae | dorsifixed | 0.9841 |
| Superrosidae | dorsifixed | 0.9495 |
| Rosidae | dorsifixed | 0.9731 |
| Malvidae | dorsifixed | 0.9757 |
| Fabidae | dorsifixed | 0.9998 |

| Model | LogL | Npar | AIC | AICc | DeltaAICc | w | q01 | q10 |
|---|---|---|---|---|---|---|---|---|
| ARD | −264.94 | 2 | 533.87 | 533.89 | 0.47 | 0.41 | 0.0039 | 0.0074 |
| ARDeq** | −264.7 | 2 | 533.4 | 533.42 | 0 | 0.51 | 0.004 | 0.0073 |
| ER | −267.53 | 1 | 537.06 | 537.07 | 3.65 | 0.08 | 0.0053 | 0.0053 |
| UNI01 | −296.15 | 1 | 594.3 | 594.3 | 60.89 | 0 | 0.0057 | |
| UNI10 | −276.23 | 1 | 554.46 | 554.47 | 21.05 | 0 | | 0.0075 |

MP ancestral state reconstruction using ancestral.pars
(R:phangorn)
313_A. Anther dehiscence (3−state) (D2d), 10 steps

- longitudinal slit
- H−valvate
- flap−valvate

| Node | MP state(s) |
|---|---|
| Angiospermae | longitudinal slit |
| Mesangiospermae | longitudinal slit |
| Magnoliidae | longitudinal slit |
| Monocotyledonae | longitudinal slit |
| Eudicotyledonae | longitudinal slit |
| Commelinidae | longitudinal slit |
| Pentapetalae | longitudinal slit |
| Superasteridae | longitudinal slit |
| Asteridae | longitudinal slit |
| Lamiidae | longitudinal slit |
| Campanulidae | longitudinal slit |
| Superrosidae | longitudinal slit |
| Rosidae | longitudinal slit |
| Malvidae | longitudinal slit |
| Fabidae | longitudinal slit |

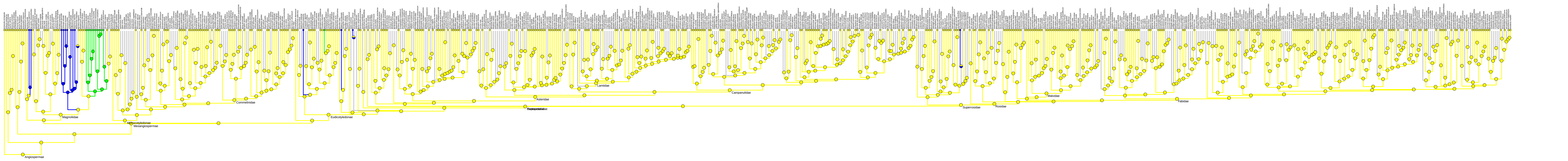

ML ancestral state reconstruction using rayDISC (R:corHMM)
313_A. Anther dehiscence (3−state) (D2d), ARDeq model

- longitudinal slit
- H−valvate
- flap−valvate

| Node | ML state | Prob |
|---|---|---|
| Angiospermae | longitudinal slit | 1 |
| Mesangiospermae | longitudinal slit | 0.9999 |
| Magnoliidae | longitudinal slit | 0.9722 |
| Monocotyledonae | longitudinal slit | 1 |
| Eudicotyledonae | longitudinal slit | 1 |
| Commelinidae | longitudinal slit | 1 |
| Pentapetalae | longitudinal slit | 1 |
| Superasteridae | longitudinal slit | 1 |
| Asteridae | longitudinal slit | 1 |
| Lamiidae | longitudinal slit | 1 |
| Campanulidae | longitudinal slit | 1 |
| Superrosidae | longitudinal slit | 1 |
| Rosidae | longitudinal slit | 1 |
| Malvidae | longitudinal slit | 1 |
| Fabidae | longitudinal slit | 1 |

| Model | LogL | Npar | AIC | AICc | DeltaAICc | w | q01 | |
|---|---|---|---|---|---|---|---|---|
| ARD | −55.71 | 6 | 123.41 | 123.52 | 2.04 | 0.14 | 2e−04 | ··· |
| ARDeq* | −54.68 | 6 | 121.37 | 121.48 | 0 | 0.38 | 2e−04 | ··· |
| ER | −60.41 | 1 | 122.82 | 122.83 | 1.35 | 0.19 | 1e−04 | ··· |
| SYM | −59.39 | 3 | 124.78 | 124.81 | 3.34 | 0.07 | 2e−04 | ··· |
| SYMeq | −58.29 | 3 | 122.59 | 122.62 | 1.14 | 0.22 | 2e−04 | ··· |

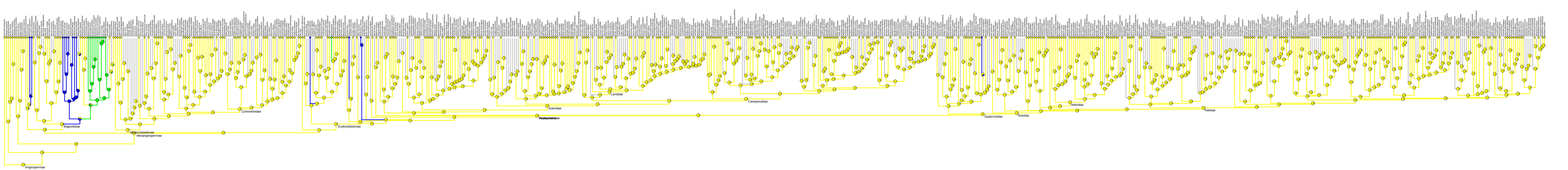

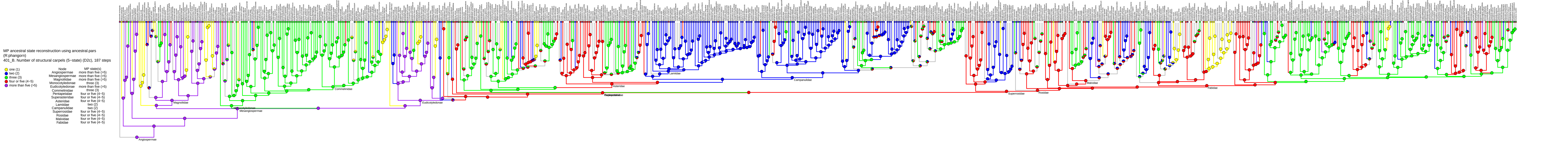

MP ancestral state reconstruction using ancestral.pars
(R:phangorn)
401_B. Number of structural carpels (5−state) (D2c), 187 steps
one (1)
two (2)
three (3)
four or five (4−5)
more than five (>5)
Node
MP state(s)
Angiospermae
more than five (>5)
Mesangiospermae
more than five (>5)
Magnoliidae
more than five (>5)
Monocotyledonae
three (3)
Eudicotyledonae
more than five (>5)
Commelinidae
three (3)
Pentapetalae
four or five (4−5)
Superasteridae
four or five (4−5)
Asteridae
four or five (4−5)
Lamiidae
two (2)
Campanulidae
two (2)
Superrosidae
four or five (4−5)
Rosidae
four or five (4−5)
Malvidae
four or five (4−5)
Fabidae
four or five (4−5)
Angiospermae
Mesangiospermae
Magnoliidae
Commelinidae
Eudicotyledonae
Asteridae
Lamiidae
Campanulidae
Superrosidae
Rosidae
Malvidae
Fabidae

ML ancestral state reconstruction using rayDISC (R:corHMM)
401_B. Number of structural carpels (5−state) (D2c), ARDeq model

- one (1)
- two (2)
- three (3)
- four or five (4−5)
- more than five (>5)

| Node | ML state | Prob |
|---|---|---|
| Angiospermae | more than five (>5) | 1 |
| Mesangiospermae | more than five (>5) | 0.9999 |
| Magnoliidae | more than five (>5) | 0.9999 |
| Monocotyledonae | more than five (>5) | 0.8253 |
| Eudicotyledonae | more than five (>5) | 0.9941 |
| Commelinidae | three (3) | 0.9999 |
| Pentapetalae | four or five (4−5) | 0.9982 |
| Superasteridae | four or five (4−5) | 0.9982 |
| Asteridae | four or five (4−5) | 0.994 |
| Lamiidae | two (2) | 0.9985 |
| Campanulidae | two (2) | 0.9659 |
| Superrosidae | four or five (4−5) | 0.9999 |
| Rosidae | four or five (4−5) | 0.9998 |
| Malvidae | four or five (4−5) | 0.9991 |
| Fabidae | four or five (4−5) | 1 |

| Model | LogL | Npar | AIC | AICc | DeltaAICc | w | q01 | ··· |
|---|---|---|---|---|---|---|---|---|
| ARD | −664.41 | 20 | 1368.82 | 1369.9 | 3.12 | 0.17 | 0.0014 | ··· |
| ARDeq** | −662.85 | 20 | 1365.7 | 1366.79 | 0 | 0.83 | 0.0014 | ··· |
| ER | −718.48 | 1 | 1438.96 | 1438.96 | 72.18 | 0 | 0.0015 | ··· |
| SYM | −688 | 10 | 1396 | 1396.28 | 29.49 | 0 | 8e−04 | ··· |
| SYMeq | −686.76 | 10 | 1393.52 | 1393.8 | 27.01 | 0 | 8e−04 | ··· |
| ORD | −756.87 | 8 | 1529.73 | 1529.92 | 163.13 | 0 | 7e−04 | ··· |
| ORDeq | −755.95 | 8 | 1527.91 | 1528.09 | 161.31 | 0 | 7e−04 | ··· |
| ORDSYM | −760.38 | 4 | 1528.77 | 1528.82 | 162.03 | 0 | 0.0038 | ··· |
| ORDSYMeq | −759.68 | 4 | 1527.35 | 1527.4 | 160.61 | 0 | 0.0038 | ··· |
| ORDER | −766.45 | 1 | 1534.89 | 1534.9 | 168.11 | 0 | 0.0055 | ··· |

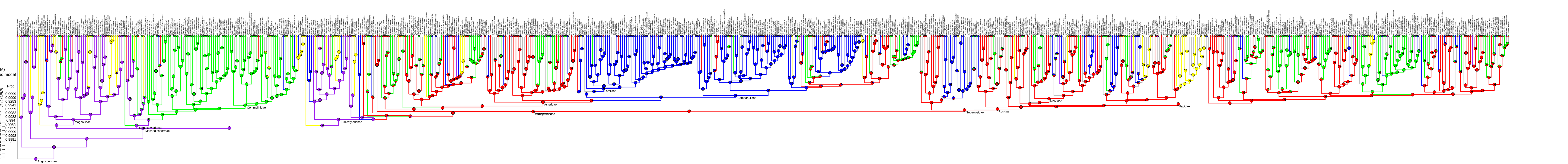

MP ancestral state reconstruction using ancestral.pars
(R:phangorn)
400_A. Gynoecium phyllotaxy (D2d), 10 steps

whorled
spiral

| Node | MP state(s) |
|---|---|
| Angiospermae | whorled / spiral |
| Mesangiospermae | whorled / spiral |
| Magnoliidae | whorled / spiral |
| Monocotyledonae | whorled |
| Eudicotyledonae | whorled |
| Commelinidae | whorled |
| Pentapetalae | whorled |
| Superasteridae | whorled |
| Asteridae | whorled |
| Lamiidae | whorled |
| Campanulidae | whorled |
| Superrosidae | whorled |
| Rosidae | whorled |
| Malvidae | whorled |
| Fabidae | whorled |

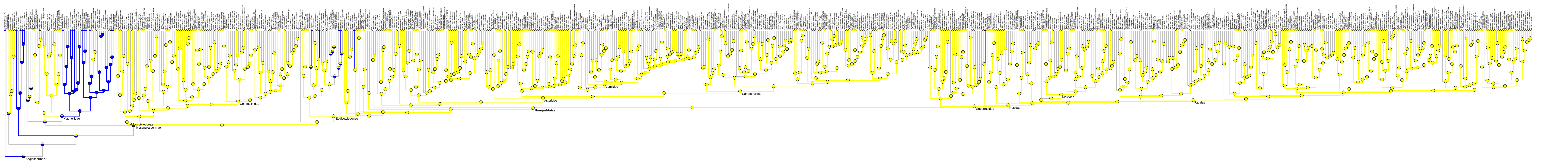

ML ancestral state reconstruction using rayDISC (R:corHMM)
400_A. Gynoecium phyllotaxy (D2d), ARDeq model

- whorled
- spiral

| Node | ML state | Prob |
|---|---|---|
| Angiospermae | spiral | 1 |
| Mesangiospermae | spiral | 1 |
| Magnoliidae | spiral | 0.9997 |
| Monocotyledonae | spiral | 0.6149 |
| Eudicotyledonae | spiral | 0.9988 |
| Commelinidae | whorled | 0.9999 |
| Pentapetalae | whorled | 0.9742 |
| Superasteridae | whorled | 0.9742 |
| Asteridae | whorled | 1 |
| Lamiidae | whorled | 1 |
| Campanulidae | whorled | 1 |
| Superrosidae | whorled | 0.9884 |
| Rosidae | whorled | 0.9884 |
| Malvidae | whorled | 1 |
| Fabidae | whorled | 1 |

| Model | LogL | Npar | AIC | AICc | DeltaAICc | w | q01 | q10 |
|---|---|---|---|---|---|---|---|---|
| ARD | −38.54 | 2 | 81.08 | 81.1 | 1.39 | 0.32 | 0 | 0.0067 |
| ARDeq** | −37.85 | 2 | 79.7 | 79.71 | 0 | 0.64 | 0 | 0.0067 |
| ER | −49.57 | 1 | 101.13 | 101.14 | 21.42 | 0 | 4e−04 | 4e−04 |
| UNI01 | −51.34 | 1 | 104.69 | 104.69 | 24.98 | 0 | 4e−04 | |
| UNI10 | −41.6 | 1 | 85.2 | 85.2 | 5.49 | 0.04 | | 0.0081 |

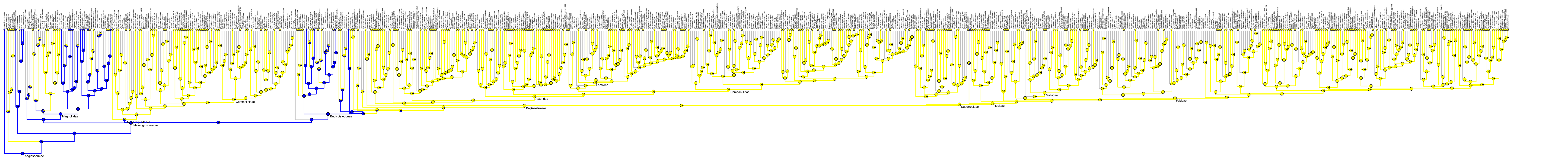

MP ancestral state reconstruction using ancestral.pars
(R:phangorn)
403_A. Fusion of ovaries (binary) (D2c), 24 steps

- free (<5%)
- fused (>5%)

| Node | MP state(s) |
|---|---|
| Angiospermae | free (<5%) / fused (>5%) |
| Mesangiospermae | free (<5%) / fused (>5%) |
| Magnoliidae | free (<5%) / fused (>5%) |
| Monocotyledonae | fused (>5%) |
| Eudicotyledonae | free (<5%) / fused (>5%) |
| Commelinidae | fused (>5%) |
| Pentapetalae | fused (>5%) |
| Superasteridae | fused (>5%) |
| Asteridae | fused (>5%) |
| Lamiidae | fused (>5%) |
| Campanulidae | fused (>5%) |
| Superrosidae | fused (>5%) |
| Rosidae | fused (>5%) |
| Malvidae | fused (>5%) |
| Fabidae | fused (>5%) |

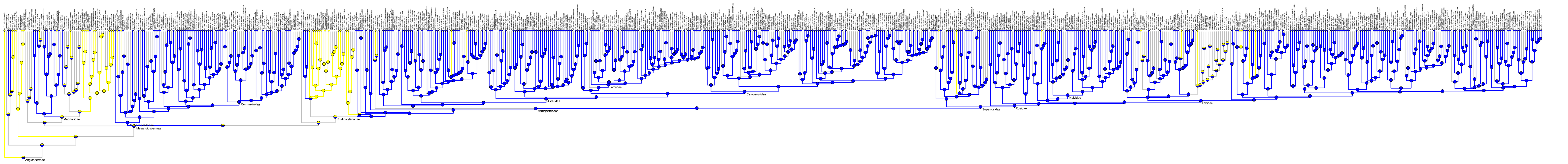

ML ancestral state reconstruction using rayDISC (R:corHMM)
403_A. Fusion of ovaries (binary) (D2c), ER model

- free (<5%)
- fused (>5%)

| Node | ML state | Prob |
|---|---|---|
| Angiospermae | fused (>5%) | 0.6618 |
| Mesangiospermae | fused (>5%) | 0.9941 |
| Magnoliidae | fused (>5%) | 0.9916 |
| Monocotyledonae | fused (>5%) | 0.9994 |
| Eudicotyledonae | fused (>5%) | 0.9957 |
| Commelinidae | fused (>5%) | 1 |
| Pentapetalae | fused (>5%) | 1 |
| Superasteridae | fused (>5%) | 1 |
| Asteridae | fused (>5%) | 1 |
| Lamiidae | fused (>5%) | 1 |
| Campanulidae | fused (>5%) | 1 |
| Superrosidae | fused (>5%) | 1 |
| Rosidae | fused (>5%) | 1 |
| Malvidae | fused (>5%) | 1 |
| Fabidae | fused (>5%) | 1 |

| Model | LogL | Npar | AIC | AICc | DeltaAICc | w | q01 | q10 |
|---|---|---|---|---|---|---|---|---|
| ARD | −100.54 | 2 | 205.08 | 205.1 | 1.73 | 0.2 | 9e−04 | 7e−04 |
| ARDeq | −100.52 | 2 | 205.03 | 205.05 | 1.68 | 0.21 | 9e−04 | 7e−04 |
| ER* | −100.68 | 1 | 203.36 | 203.37 | 0 | 0.49 | 7e−04 | 7e−04 |
| UNI01 | −102.29 | 1 | 206.59 | 206.59 | 3.22 | 0.1 | 0.0101 | |
| UNI10 | −105.94 | 1 | 213.88 | 213.89 | 10.52 | 0 | | 8e−04 |

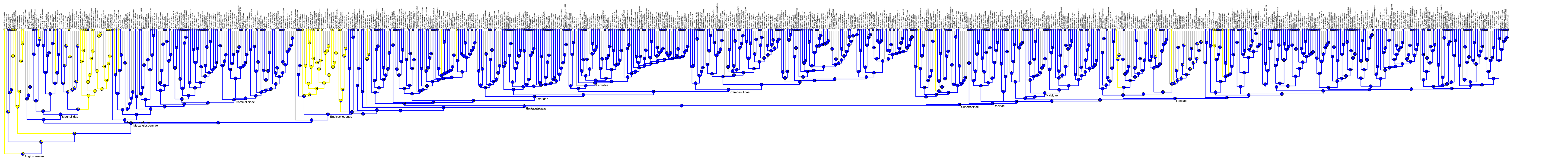

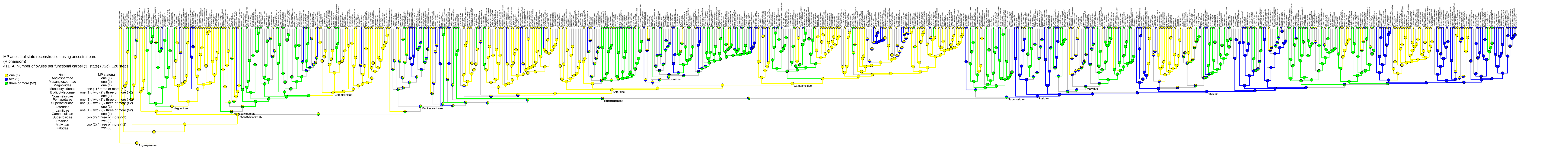
MP ancestral state reconstruction using ancestral.pars
(R:phangorn)
411_A. Number of ovules per functional carpel (3−state) (D2c), 120 steps
one (1)
two (2)
three or more (>2)
Node
MP state(s)
Angiospermae one (1)
Mesangiospermae one (1)
Magnoliidae one (1)
Monocotyledonae one (1) / three or more (>2)
Eudicotyledonae one (1) / two (2) / three or more (>2)
Commelinidae one (1)
Pentapetalae one (1) / two (2) / three or more (>2)
Superasteridae one (1) / two (2) / three or more (>2)
Asteridae one (1)
Lamiidae one (1) / two (2) / three or more (>2)
Campanulidae one (1)
Superrosidae two (2) / three or more (>2)
Rosidae two (2)
Malvidae two (2) / three or more (>2)
Fabidae two (2)
Angiospermae
Magnoliidae
Mesangiospermae
Commelinidae
Eudicotyledonae
Asteridae
Lamiidae
Campanulidae
Superrosidae
Rosidae
Malvidae
Fabidae

ML ancestral state reconstruction using rayDISC (R:corHMM)
411_A. Number of ovules per functional carpel (3−state) (D2c), ARDeq model

- one (1)
- two (2)
- three or more (>2)

| Node | ML state | Prob |
|---|---|---|
| Angiospermae | three or more (>2) | 0.9998 |
| Mesangiospermae | three or more (>2) | 1 |
| Magnoliidae | three or more (>2) | 0.9982 |
| Monocotyledonae | three or more (>2) | 0.9998 |
| Eudicotyledonae | three or more (>2) | 0.9999 |
| Commelinidae | three or more (>2) | 0.8759 |
| Pentapetalae | three or more (>2) | 1 |
| Superasteridae | three or more (>2) | 1 |
| Asteridae | three or more (>2) | 0.984 |
| Lamiidae | three or more (>2) | 1 |
| Campanulidae | three or more (>2) | 0.9971 |
| Superrosidae | three or more (>2) | 1 |
| Rosidae | three or more (>2) | 0.9999 |
| Malvidae | three or more (>2) | 0.9996 |
| Fabidae | three or more (>2) | 1 |

| Model | LogL | Npar | AIC | AICc | DeltaAICc | w | q01 | ... |
|---|---|---|---|---|---|---|---|---|
| ARD | −384.28 | 6 | 780.57 | 780.68 | 1.9 | 0.28 | 0.0013 | ··· |
| ARDeq** | −383.33 | 6 | 778.67 | 778.77 | 0 | 0.72 | 0.0013 | ··· |
| ER | −398.73 | 1 | 799.46 | 799.47 | 20.69 | 0 | 0.0027 | ··· |
| SYM | −398.04 | 3 | 802.08 | 802.11 | 23.34 | 0 | 0.0022 | ··· |
| SYMeq | −397.58 | 3 | 801.15 | 801.18 | 22.41 | 0 | 0.0022 | ··· |
| ORD | −393.81 | 4 | 795.61 | 795.66 | 16.89 | 0 | 0 | ··· |
| ORDeq | −392.88 | 4 | 793.76 | 793.81 | 15.03 | 0 | 0 | ··· |
| ORDSYM | −410.81 | 2 | 825.63 | 825.64 | 46.87 | 0 | 0.0051 | ··· |
| ORDSYMeq | −410.6 | 2 | 825.2 | 825.22 | 46.45 | 0 | 0.0051 | ··· |
| ORDER | −412.04 | 1 | 826.08 | 826.08 | 47.31 | 0 | 0.006 | ··· |

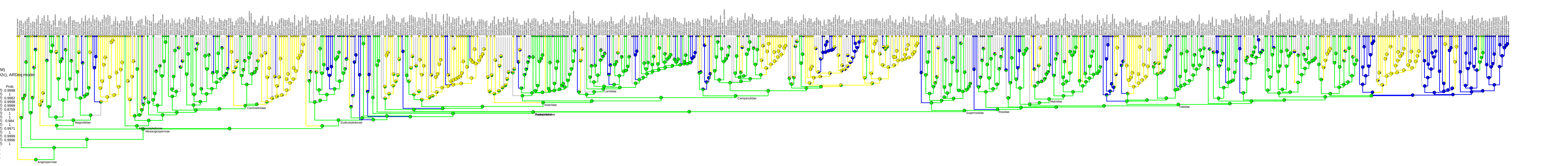

Supplement: Supplementary Data 21 [file ncomms16047-s22.pdf]
