# Supplementary material for: The ancestral flower of angiosperms and its early diversification
Source: Nat Commun. 2017 Aug 1;8:16047. doi: 10.1038/ncomms16047 (PMC5543309; doi:10.1038/ncomms16047)

MP ancestral state reconstruction using ancestral.pars
(R:phangorn)
100_A. Functional sex of flowers (D2d), 98 steps

- bisexual
- unisexual

Node	MP state(s)
Angiospermae	bisexual / unisexual
Mesangiospermae	bisexual
Magnoliidae	bisexual
Monocotyledonae	bisexual
Eudicotyledonae	bisexual
Commelinidae	bisexual
Pentapetalae	bisexual
Superasteridae	bisexual
Asteridae	bisexual
Lamiidae	bisexual
Campanulidae	bisexual
Superrosidae	bisexual
Rosidae	bisexual
Malvidae	bisexual
Fabidae	bisexual

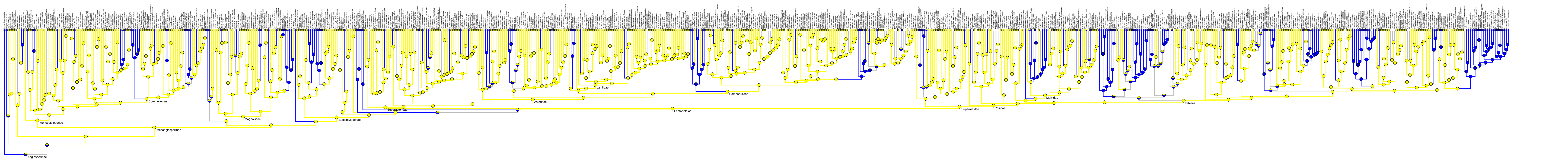

ML ancestral state reconstruction using rayDISC (R:corHMM)
100_A. Functional sex of flowers (D2d), ARDeq model

- bisexual
- unisexual

Node	ML state	Prob
Angiospermae	bisexual	0.9999
Mesangiospermae	bisexual	1
Magnoliidae	bisexual	1
Monocotyledonae	bisexual	0.9999
Eudicotyledonae	bisexual	1
Commelinidae	bisexual	0.9997
Pentapetalae	bisexual	1
Superasteridae	bisexual	1
Asteridae	bisexual	1
Lamiidae	bisexual	1
Campanulidae	bisexual	0.9991
Superrosidae	bisexual	1
Rosidae	bisexual	1
Malvidae	bisexual	0.9995
Fabidae	bisexual	1

Model	LogL	Npar	AIC	AICc	DeltaAICc	w	q01	q10
ARD	−312.08	2	628.16	628.18	1.24	0.25	0.0028	0.001
ARDeq*	−311.46	2	626.92	626.94	0	0.46	0.0028	9e−04
ER	−313.98	1	629.95	629.96	3.02	0.1	0.0028	0.0028
UNI01	−313.34	1	628.68	628.68	1.74	0.19	0.0029	
UNI10	−359.8	1	721.61	721.61	94.68	0		0.0112

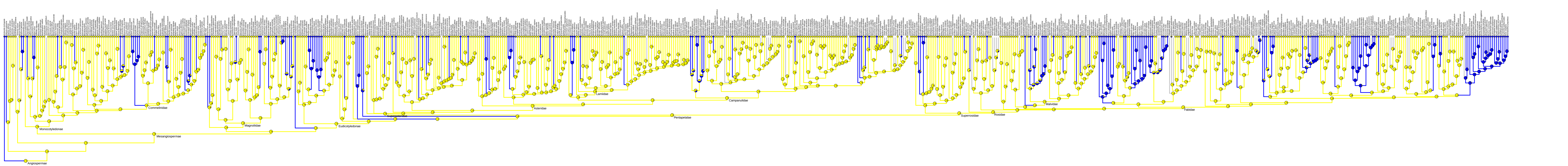

MP ancestral state reconstruction using ancestral.pars
(R:phangorn)
100_B. Structural sex of flowers (D2d), 57 steps

- bisexual
- unisexual

Node	MP state(s)
Angiospermae	bisexual
Mesangiospermae	bisexual
Magnoliidae	bisexual
Monocotyledonae	bisexual
Eudicotyledonae	bisexual
Commelinidae	bisexual
Pentapetalae	bisexual
Superasteridae	bisexual
Asteridae	bisexual
Lamiidae	bisexual
Campanulidae	bisexual
Superrosidae	bisexual
Rosidae	bisexual
Malvidae	bisexual
Fabidae	bisexual

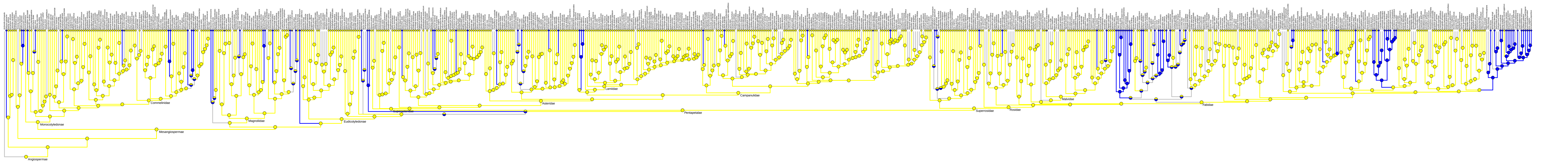

ML ancestral state reconstruction using rayDISC (R:corHMM)
100_B. Structural sex of flowers (D2d), ARDeq model

- bisexual
- unisexual

Node	ML state	Prob
Angiospermae	bisexual	0.9964
Mesangiospermae	bisexual	0.9995
Magnoliidae	bisexual	0.9996
Monocotyledonae	bisexual	0.9995
Eudicotyledonae	bisexual	0.9998
Commelinidae	bisexual	0.9999
Pentapetalae	bisexual	1
Superasteridae	bisexual	1
Asteridae	bisexual	1
Lamiidae	bisexual	0.9999
Campanulidae	bisexual	1
Superrosidae	bisexual	1
Rosidae	bisexual	1
Malvidae	bisexual	1
Fabidae	bisexual	0.9976

Model	LogL	Npar	AIC	AICc	DeltaAICc	w	q01	q10
ARD	−206.91	2	417.82	417.83	0.85	0.35	0.0015	0.0041
ARDeq**	−206.48	2	416.97	416.98	0	0.54	0.0015	0.0039
ER	−209.08	1	420.16	420.16	3.18	0.11	0.0015	0.0015
UNI01	−223.23	1	448.46	448.47	31.48	0	0.0016	
UNI10	−234.37	1	470.74	470.74	53.76	0		0.0154

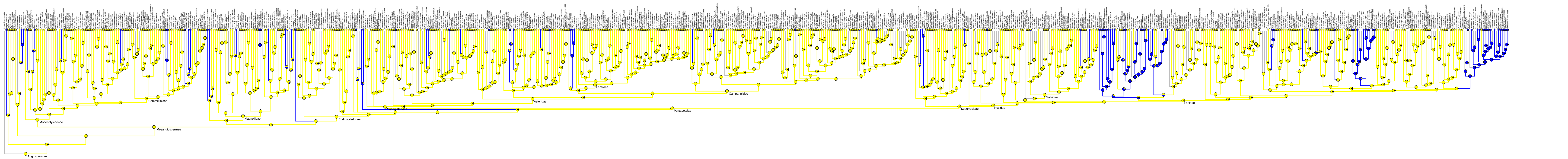

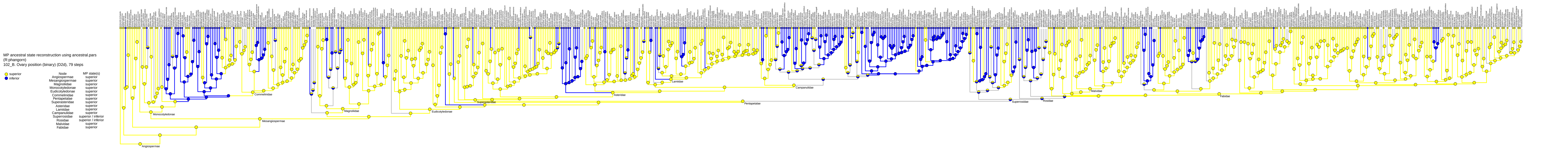
MP ancestral state reconstruction using ancestral.pars
(R:phangorn)
102_B. Ovary position (binary) (D2d), 79 steps
superior
inferior
Node
MP state(s)
Angiospermae superior
Mesangiospermae superior
Magnoliidae superior
Monocotyledonae superior
Eudicotyledonae superior
Commelinidae superior
Pentapetalae superior
Superasteridae superior
Asteridae superior
Lamiidae superior
Campanulidae superior
Superrosidae superior / inferior
Rosidae superior / inferior
Malvidae superior
Fabidae superior
Commelinidae
Monocotyledonae
Mesangiospermae
Angiospermae
Magnoliidae
Eudicotyledonae
Superasteridae
Asteridae
Lamiidae
Campanulidae
Pentapetalae
Superrosidae
Rosidae
Malvidae
Fabidae

ML ancestral state reconstruction using rayDISC (R:corHMM)
102_B. Ovary position (binary) (D2d), ARDeq model

- superior
- inferior

Node	ML state	Prob
Angiospermae	superior	0.9998
Mesangiospermae	superior	0.9991
Magnoliidae	superior	0.9958
Monocotyledonae	superior	0.9835
Eudicotyledonae	superior	0.9999
Commelinidae	superior	0.6642
Pentapetalae	superior	0.9979
Superasteridae	superior	0.9995
Asteridae	superior	0.9399
Lamiidae	superior	0.9519
Campanulidae	superior	0.7333
Superrosidae	superior	0.9569
Rosidae	superior	0.957
Malvidae	superior	[illegible]
Fabidae	superior	0.9994

Model	LogL	Npar	AIC	AICc	DeltaAICc	w	q01	q10
ARD	−271.06	2	546.11	[illegible]	1.18	0.3	0.0019	0.0037
ARDeq**	−270.47	2	544.93	544.95	0	0.55	0.0019	0.0037
ER	−272.76	1	547.53	547.53	2.59	0.15	0.0023	0.0023
UNI01	−281.98	1	565.95	565.96	21.01	0	0.0026	
UNI10	−295.2	1	592.4	592.4	47.46	0		0.0094

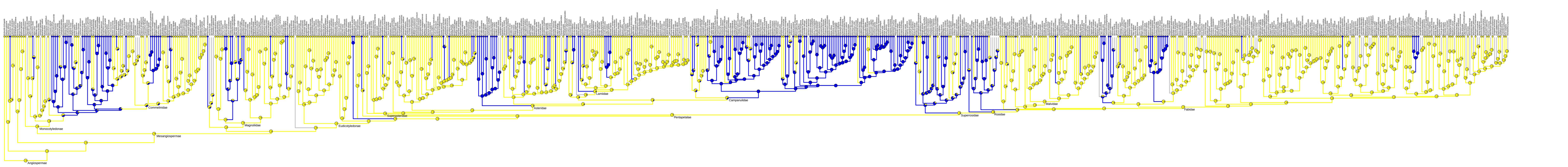

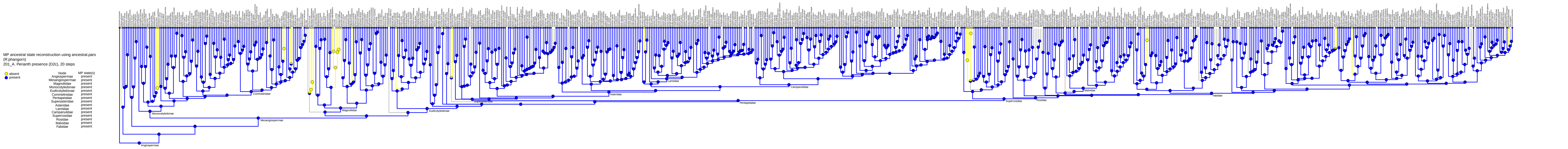
MP ancestral state reconstruction using ancestral.pars
(R:phangorn)
201_A. Perianth presence (D2c), 20 steps
absent
present
Node
MP state(s)
Angiospermae present
Mesangiospermae present
Magnoliidae present
Monocotyledonae present
Eudicotyledonae present
Commelinidae present
Pentapetalae present
Superasteridae present
Asteridae present
Lamiidae present
Campanulidae present
Superrosidae present
Rosidae present
Malvidae present
Fabidae present
Angiospermae
Mesangiospermae
Monocotyledonae
Commelinidae
Magnoliidae
Eudicotyledonae
Asteridae
Lamiidae
Campanulidae
Pentapetalae
Superrosidae
Rosidae
Malvidae
Fabidae

ML ancestral state reconstruction using rayDISC (R:corHMM)
201_A. Perianth presence (D2c), UNI10 model

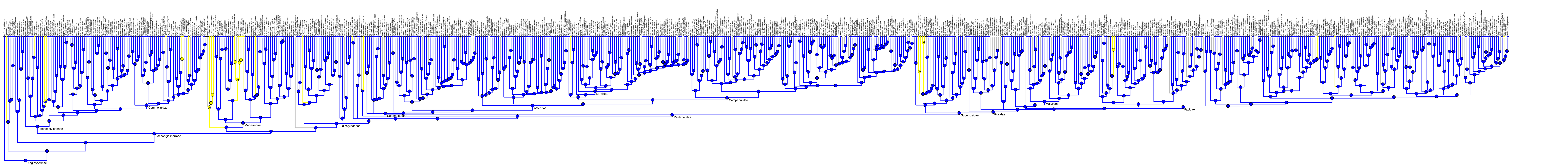

Node	ML state	Prob
Angiospermae	present	1
Mesangiospermae	present	1
Magnoliidae	present	1
Monocotyledonae	present	1
Eudicotyledonae	present	1
Commelinidae	present	1
Pentapetalae	present	1
Superasteridae	present	1
Asteridae	present	1
Lamiidae	present	1
Campanulidae	present	1
Superrosidae	present	1
Rosidae	present	1
Malvidae	present	1
Fabidae	present	1

Model	LogL	Npar	AIC	AICc	DeltaAICc	w	q01	q10
ARD	−95.77	2	195.54	195.55	2.01	0.13	0	5e−04
ARDeq	−95.08	2	194.15	194.17	0.62	0.26	0	5e−04
ER	−96.05	1	194.09	194.1	0.56	0.27	5e−04	5e−04
UNI01	−114.11	1	230.21	230.22	36.67	0	0.02	
UNI10*	−95.77	1	193.54	193.54	0	0.35		5e−04

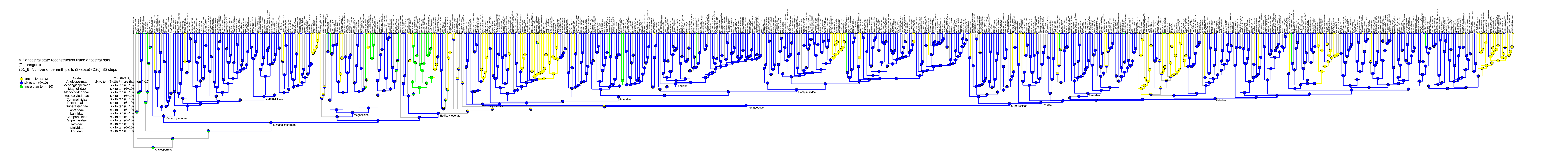
MP ancestral state reconstruction using ancestral.pars
(R:phangorn)
201_B. Number of perianth parts (3−state) (D2c), 85 steps
one to five (1−5)
six to ten (6−10)
more than ten (>10)
Node
MP state(s)
Angiospermae six to ten (6−10) / more than ten (>10)
Mesangiospermae six to ten (6−10)
Magnoliidae six to ten (6−10)
Monocotyledonae six to ten (6−10)
Eudicotyledonae six to ten (6−10)
Commelinidae six to ten (6−10)
Pentapetalae six to ten (6−10)
Superasteridae six to ten (6−10)
Asteridae six to ten (6−10)
Lamiidae six to ten (6−10)
Campanulidae six to ten (6−10)
Superrosidae six to ten (6−10)
Rosidae six to ten (6−10)
Malvidae six to ten (6−10)
Fabidae six to ten (6−10)
Commelinidae
Monocotyledonae
Magnoliidae
Mesangiospermae
Eudicotyledonae
Superasteridae
Asteridae
Lamiidae
Campanulidae
Pentapetalae
Superrosidae
Rosidae
Malvidae
Fabidae
Angiospermae

ML ancestral state reconstruction using rayDISC (R:corHMM)
201_B. Number of perianth parts (3−state) (D2c), ARDeq model

- one to five (1−5)
- six to ten (6−10)
- more than ten (>10)

Node	ML state	Prob
Angiospermae	more than ten (>10)	1
Mesangiospermae	more than ten (>10)	0.9977
Magnoliidae	more than ten (>10)	0.9995
Monocotyledonae	six to ten (6−10)	0.6429
Eudicotyledonae	more than ten (>10)	0.9896
Commelinidae	six to ten (6−10)	0.9999
Pentapetalae	six to ten (6−10)	0.9927
Superasteridae	six to ten (6−10)	0.9978
Asteridae	six to ten (6−10)	0.9999
Lamiidae	six to ten (6−10)	0.9999
Campanulidae	six to ten (6−10)	0.9999
Superrosidae	six to ten (6−10)	0.9988
Rosidae	six to ten (6−10)	0.9991
Malvidae	six to ten (6−10)	0.9999
Fabidae	six to ten (6−10)	0.9993

Model	LogL	Npar	AIC	AICc	DeltaAICc	w	q01	
ARD	−323.1	6	658.2	658.3	2.14	0.25	0.004	···
ARDeq**	−322.03	6	656.06	656.17	0	0.73	0.004	···
ER	−348.35	1	698.7	698.71	42.54	0	0.0012	···
SYM	−337.02	3	680.04	680.07	23.9	0	0.002	···
SYMeq	−336.16	3	678.32	678.35	22.19	0	0.002	···
ORD	−328.91	4	665.83	665.88	9.71	0.01	0.0042	···
ORDeq	−327.84	4	663.67	663.72	7.56	0.02	0.0041	···
ORDSYM	−339.89	2	683.78	683.8	27.63	0	0.002	···
ORDSYMeq	−339.14	2	682.27	682.29	26.12	0	0.002	···
ORDER	−349.6	1	701.21	701.21	45.05	0	0.0014	···

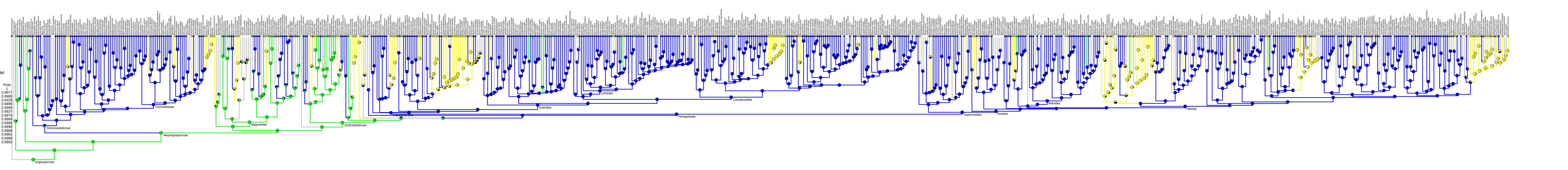

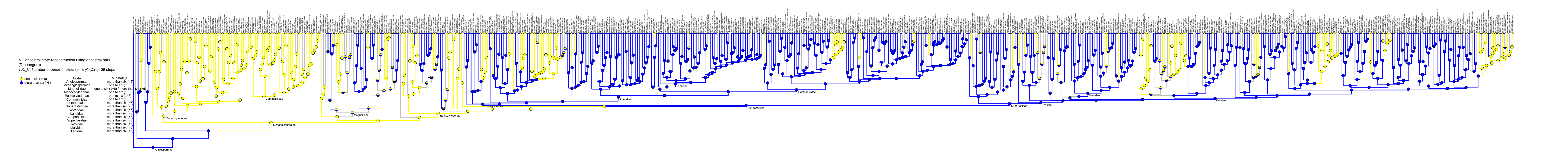
MP ancestral state reconstruction using ancestral.pars
(R:phangorn)
201_C. Number of perianth parts (binary) (D2c), 69 steps
one to six (1−6)
more than six (>6)
Node | MP state(s)
Angiospermae | more than six (>6)
Mesangiospermae | one to six (1−6)
Magnoliidae | one to six (1−6) / more than six (>6)
Monocotyledonae | one to six (1−6)
Eudicotyledonae | one to six (1−6)
Commelinidae | one to six (1−6)
Pentapetalae | more than six (>6)
Superasteridae | more than six (>6)
Asteridae | more than six (>6)
Lamiidae | more than six (>6)
Campanulidae | more than six (>6)
Superrosidae | more than six (>6)
Rosidae | more than six (>6)
Malvidae | more than six (>6)
Fabidae | more than six (>6)
Angiospermae
Monocotyledonae
Commelinidae
Mesangiospermae
Magnoliidae
Eudicotyledonae
Superasteridae
Asteridae
Lamiidae
Campanulidae
Pentapetalae
Superrosidae
Rosidae
Malvidae
Fabidae

ML ancestral state reconstruction using rayDISC (R:corHMM)
201_C. Number of perianth parts (binary) (D2c), ARDeq model

- one to six (1−6)
- more than six (>6)

Node	ML state	Prob
Angiospermae	more than six (>6)	1
Mesangiospermae	more than six (>6)	0.9971
Magnoliidae	more than six (>6)	0.9999
Monocotyledonae	one to six (1−6)	0.7993
Eudicotyledonae	more than six (>6)	0.9999
Commelinidae	one to six (1−6)	1
Pentapetalae	more than six (>6)	1
Superasteridae	more than six (>6)	1
Asteridae	more than six (>6)	1
Lamiidae	more than six (>6)	1
Campanulidae	more than six (>6)	1
Superrosidae	more than six (>6)	1
Rosidae	more than six (>6)	1
Malvidae	more than six (>6)	1
Fabidae	more than six (>6)	1

Model	LogL	Npar	AIC	AICc	DeltaAICc	w	q01	q10
ARD	−245.96	2	495.91	[illegible]	1.38	[illegible]	6e−04	0.0025
ARDeq**	−245.27	2	494.53	494.55	0	0.66	6e−04	0.0025
ER	−251.67	1	505.34	505.35	10.8	0	0.0021	0.0021
UNI01	−319.59	1	641.19	641.19	146.64	0	0.0076	
UNI10	−251.65	1	505.3	505.3	10.75	0		0.0028

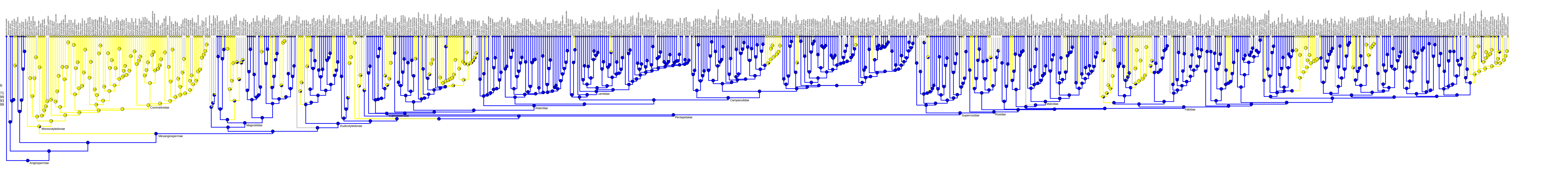

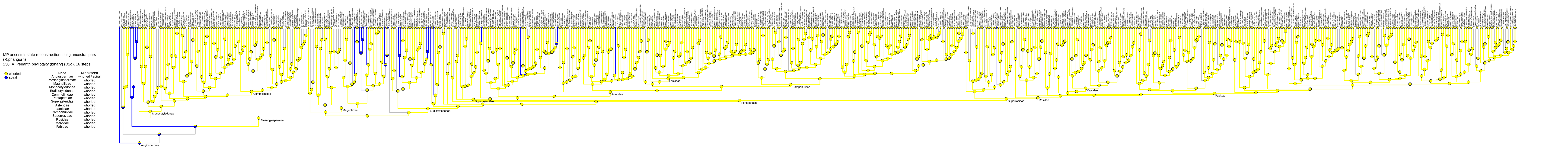
MP ancestral state reconstruction using ancestral.pars
(R:phangorn)
230_A. Perianth phyllotaxy (binary) (D2d), 16 steps
whorled
spiral
Node
MP state(s)
Angiospermae
whorled / spiral
Mesangiospermae
whorled
Magnoliidae
whorled
Monocotyledonae
whorled
Eudicotyledonae
whorled
Commelinidae
whorled
Pentapetalae
whorled
Superasteridae
whorled
Asteridae
whorled
Lamiidae
whorled
Campanulidae
whorled
Superrosidae
whorled
Rosidae
whorled
Malvidae
whorled
Fabidae
whorled
Commelinidae
Monocotyledonae
Mesangiospermae
Angiospermae
Magnoliidae
Eudicotyledonae
Superasteridae
Asteridae
Lamiidae
Campanulidae
Pentapetalae
Superrosidae
Rosidae
Malvidae
Fabidae

ML ancestral state reconstruction using rayDISC (R:corHMM)
230_A. Perianth phyllotaxy (binary) (D2d), ARD model

- whorled
- spiral

Node	ML state	Prob
Angiospermae	spiral	0.9991
Mesangiospermae	spiral	0.9991
Magnoliidae	spiral	0.9975
Monocotyledonae	whorled	0.6024
Eudicotyledonae	spiral	0.9985
Commelinidae	whorled	0.9999
Pentapetalae	whorled	0.9794
Superasteridae	whorled	0.9845
Asteridae	whorled	0.9997
Lamiidae	whorled	1
Campanulidae	whorled	1
Superrosidae	whorled	0.9945
Rosidae	whorled	0.9966
Malvidae	whorled	1
Fabidae	whorled	1

Model	LogL	Npar	AIC	AICc	DeltaAICc	[illegible]	q01	q10
ARD***	−69.87	2	143.75	143.76	0	0.99	2e−04	0.0093
ARDeq	−75.71	2	155.42	155.43	11.67	0	4e−04	0
ER	−76.18	1	154.37	154.37	10.61	0	4e−04	4e−04
UNI01	−76.4	1	154.8	154.81	11.04	0	4e−04	
UNI10	−78.94	1	159.89	159.89	16.13	0		0.0161

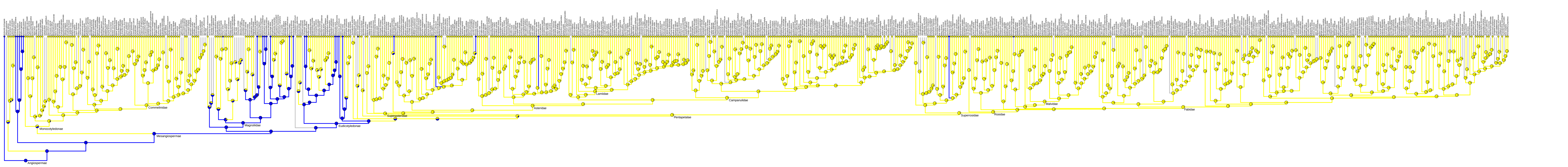

MP ancestral state reconstruction using ancestral.pars
(R:phangorn)
231_A. Number of perianth whorls (D2c), 73 steps

- one (1)
- two (2)
- more than two (>2)

Node	MP state(s)
Angiospermae	more than two (>2)
Mesangiospermae	more than two (>2)
Magnoliidae	more than two (>2)
Monocotyledonae	two (2)
Eudicotyledonae	more than two (>2)
Commelinidae	two (2)
Pentapetalae	two (2)
Superasteridae	two (2)
Asteridae	two (2)
Lamiidae	two (2)
Campanulidae	two (2)
Superrosidae	two (2)
Rosidae	two (2)
Malvidae	two (2)
Fabidae	two (2)

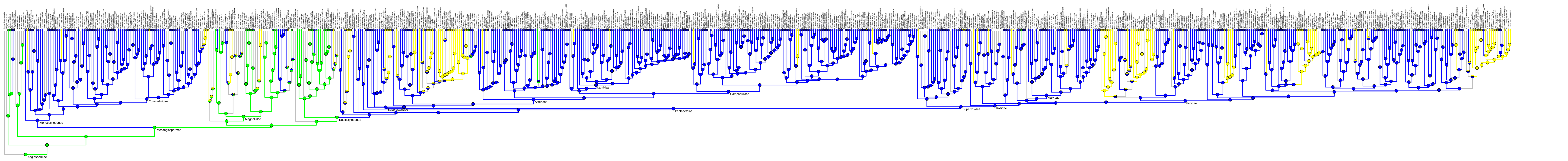

ML ancestral state reconstruction using rayDISC (R:corHMM)
231_A. Number of perianth whorls (D2c), ARDeq model

- one (1)
- two (2)
- more than two (>2)

Node	ML state	Prob
Angiospermae	more than two (>2)	1
Mesangiospermae	more than two (>2)	0.999
Magnoliidae	more than two (>2)	1
Monocotyledonae	two (2)	0.732
Eudicotyledonae	more than two (>2)	0.9929
Commelinidae	two (2)	0.9999
Pentapetalae	two (2)	0.9996
Superasteridae	two (2)	0.9995
Asteridae	two (2)	0.9997
Lamiidae	two (2)	0.9999
Campanulidae	two (2)	1
Superrosidae	two (2)	1
Rosidae	two (2)	0.9999
Malvidae	two (2)	0.9999
Fabidae	two (2)	0.9984

Model	LogL	Npar	AIC	AICc	DeltaAICc	w	q21	···
ARD	−267.02	6	546.03	[illegible]	2.18	0.25	0.0047	···
ARDeq**	−265.93	6	543.85	[illegible]	0	0.74	0.0047	···
ER	−309.43	1	620.85	620.86	76.9	0	0.001	···
SYM	−291.2	3	588.41	[illegible]	44.48	0	0.0019	···
SYMeq	−290.83	3	587.66	[illegible]	43.73	0	0.0018	···
ORD	−274.16	4	556.32	[illegible]	12.41	0	0.0045	···
ORDeq	−273.06	4	554.13	554.18	10.22	0	0.0045	···
ORDSYM	−297.48	2	598.96	598.98	55.02	0	0.0018	···
ORDSYMeq	−296.71	2	597.43	597.44	53.48	0	0.0018	···
ORDER	−312.45	1	626.89	626.9	82.94	0	0.0011	···

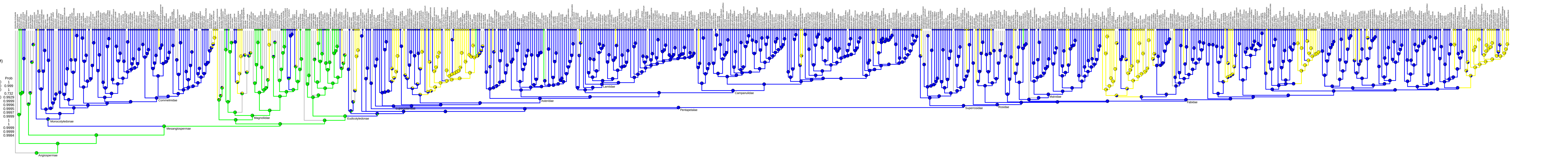

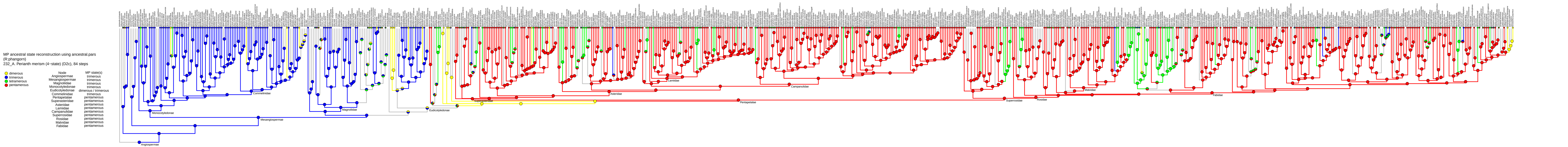
MP ancestral state reconstruction using ancestral.pars
(R:phangorn)
232_A. Perianth merism (4−state) (D2c), 84 steps
dimerous
trimerous
tetramerous
pentamerous
Node
MP state(s)
Angiospermae trimerous
Mesangiospermae trimerous
Magnoliidae trimerous
Monocotyledonae trimerous
Eudicotyledonae dimerous / trimerous
Commelinidae trimerous
Pentapetalae pentamerous
Superasteridae pentamerous
Asteridae pentamerous
Lamiidae pentamerous
Campanulidae pentamerous
Superrosidae pentamerous
Rosidae pentamerous
Malvidae pentamerous
Fabidae pentamerous

ML ancestral state reconstruction using rayDISC (R:corHMM)
232_A. Perianth merism (4−state) (D2c), SYMeq model

- dimerous
- trimerous
- tetramerous
- pentamerous

Node	ML state	Prob
Angiospermae	trimerous	1
Mesangiospermae	trimerous	0.9999
Magnoliidae	trimerous	1
Monocotyledonae	trimerous	0.9999
Eudicotyledonae	trimerous	0.8465
Commelinidae	trimerous	1
Pentapetalae	pentamerous	0.9988
Superasteridae	pentamerous	0.9999
Asteridae	pentamerous	0.9999
Lamiidae	pentamerous	1
Campanulidae	pentamerous	1
Superrosidae	pentamerous	1
Rosidae	pentamerous	1
Malvidae	pentamerous	1
Fabidae	pentamerous	1

Model	LogL	Npar	AIC	AICc	DeltaAICc	w	q01	
ARD	−320.6	12	665.2	665.6	4.89	0.05	0.001	···
ARDeq	−319.49	12	662.98	663.38	2.67	0.16	8e−04	···
ER	−360.2	1	722.39	722.4	61.69	0	8e−04	···
SYM	−325.58	6	663.16	663.27	2.56	0.17	0.0012	···
SYMeq**	−324.3	6	660.6	660.71	0	0.61	0.0012	···
ORD	−336.09	6	684.18	684.28	23.58	0	0	···
ORDeq	−335.22	6	682.44	682.55	21.84	0	0	···
ORDSYM	−344.53	3	695.05	695.08	34.38	0	0.0019	···
ORDSYMeq	−343.36	3	692.73	692.76	32.05	0	0.0019	···
ORDER	−346.22	1	694.45	694.45	33.75	0	0.0024	···

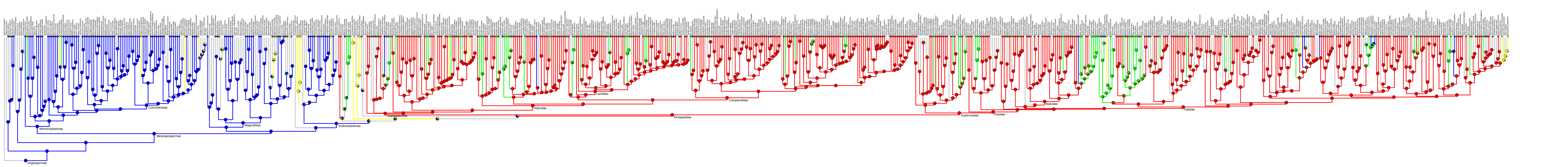

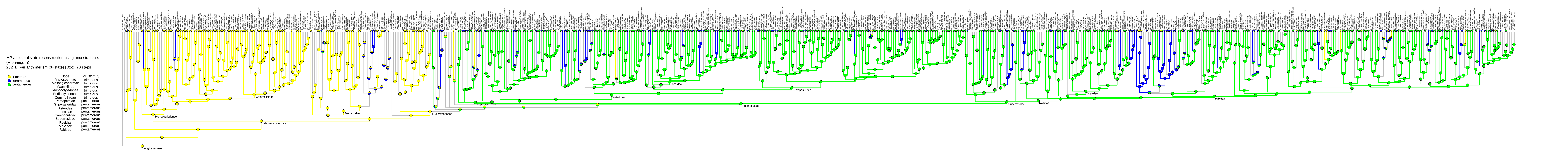
MP ancestral state reconstruction using ancestral.pars
(R:phangorn)
232_B. Perianth merism (3−state) (D2c), 70 steps
trimerous
tetramerous
pentamerous
Node
MP state(s)
Angiospermae trimerous
Mesangiospermae trimerous
Magnoliidae trimerous
Monocotyledonae trimerous
Eudicotyledonae trimerous
Commelinidae trimerous
Pentapetalae pentamerous
Superasteridae pentamerous
Asteridae pentamerous
Lamiidae pentamerous
Campanulidae pentamerous
Superrosidae pentamerous
Rosidae pentamerous
Malvidae pentamerous
Fabidae pentamerous
Commelinidae
Monocotyledonae
Mesangiospermae
Angiospermae
Magnoliidae
Eudicotyledonae
Asteridae
Lamiidae
Campanulidae
Pentapetalae
Superrosidae
Rosidae
Malvidae
Fabidae

ML ancestral state reconstruction using rayDISC (R:corHMM)
232_B. Perianth merism (3−state) (D2c), ORDeq model

- trimerous
- tetramerous
- pentamerous

Node	ML state	Prob
Angiospermae	trimerous	0.9988
Mesangiospermae	trimerous	0.9976
Magnoliidae	trimerous	0.9977
Monocotyledonae	trimerous	0.9996
Eudicotyledonae	trimerous	0.7392
Commelinidae	trimerous	1
Pentapetalae	pentamerous	0.9809
Superasteridae	pentamerous	0.991
Asteridae	pentamerous	0.9991
Lamiidae	pentamerous	0.9998
Campanulidae	pentamerous	0.9999
Superrosidae	pentamerous	0.9981
Rosidae	pentamerous	0.9992
Malvidae	pentamerous	0.9996
Fabidae	pentamerous	0.9997

Model	LogL	Npar	AIC	AICc	DeltaAICc	w	q01	
ARD	−254.32	6	520.65	520.76	5.44	0.02	8e−04	···
ARDeq	−253.42	6	518.83	518.94	3.62	0.05	8e−04	···
ER	−280.25	1	562.5	562.5	47.19	0	0.0011	···
SYM	−255.81	3	517.62	517.65	2.33	0.1	9e−04	···
SYMeq	−254.77	3	515.55	515.58	0.26	0.27	9e−04	···
ORD	−254.52	4	517.05	517.1	1.78	0.13	9e−04	···
ORDeq*	−253.63	4	515.27	515.32	0	0.31	9e−04	···
ORDSYM	−257.91	2	519.82	519.83	4.51	0.03	0.0014	···
ORDSYMeq	−256.9	2	517.8	517.82	2.5	0.09	0.0014	···
ORDER	−260.43	1	522.85	522.86	7.54	0.01	0.0023	···

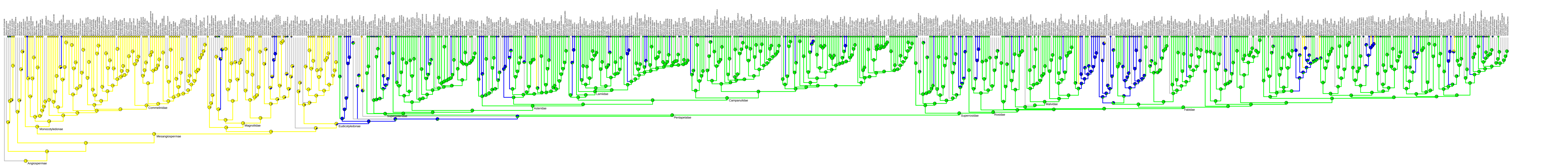

MP ancestral state reconstruction using ancestral.pars
(R:phangorn)
234_A. Perianth differentiation (binary) (D2d), 64 steps

- undifferentiated
- differentiated

Node	MP state(s)
Angiospermae	undifferentiated
Mesangiospermae	undifferentiated
Magnoliidae	undifferentiated / differentiated
Monocotyledonae	undifferentiated
Eudicotyledonae	undifferentiated / differentiated
Commelinidae	undifferentiated
Pentapetalae	differentiated
Superasteridae	differentiated
Asteridae	differentiated
Lamiidae	differentiated
Campanulidae	differentiated
Superrosidae	differentiated
Rosidae	differentiated
Malvidae	differentiated
Fabidae	differentiated

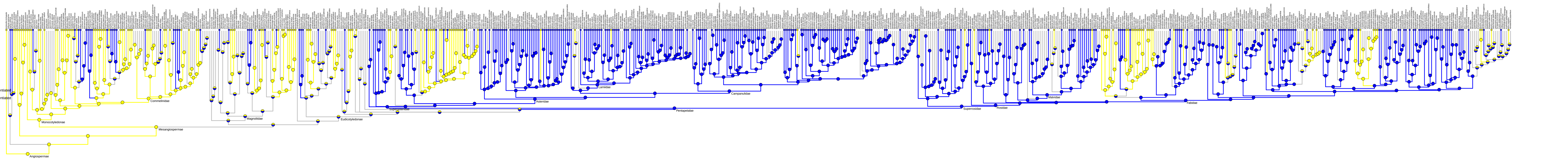

ML ancestral state reconstruction using rayDISC (R:corHMM)
234_A. Perianth differentiation (binary) (D2d), ARDeq model

- undifferentiated (yellow)
- differentiated (blue)

Node	ML state	Prob
Angiospermae	undifferentiated	1
Mesangiospermae	undifferentiated	0.9999
Magnoliidae	undifferentiated	0.9992
Monocotyledonae	undifferentiated	0.9998
Eudicotyledonae	undifferentiated	0.9651
Commelinidae	undifferentiated	0.9998
Pentapetalae	differentiated	0.9414
Superasteridae	differentiated	0.9455
Asteridae	differentiated	0.9984
Lamiidae	differentiated	0.9999
Campanulidae	differentiated	1
Superrosidae	differentiated	0.9916
Rosidae	differentiated	0.9953
Malvidae	differentiated	0.9993
Fabidae	differentiated	0.9893

Model	LogL	Npar	AIC	AICc	DeltaAICc	w	q01	q10
ARD	−200.3	2	404.59	404.61	1.32	0.34	0.0042	0.0015
ARDeq**	−199.64	2	403.28	403.29	0	0.66	0.0042	0.0015
ER	−208.52	1	419.04	419.04	15.75	0	0.0024	0.0024
UNI01	−231.57	1	465.14	465.15	61.86	0	0.0082	
UNI10	−234.38	1	470.76	470.76	67.47	0		0.0032

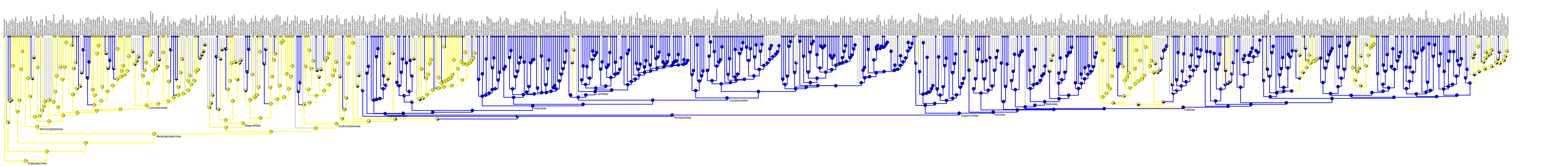

MP ancestral state reconstruction using ancestral.pars
(R:phangorn)
204_A. Fusion of perianth (D2c), 77 steps

- free (<5%)
- fused (>5%)

Node	MP state(s)
Angiospermae	free (<5%)
Mesangiospermae	free (<5%)
Magnoliidae	free (<5%)
Monocotyledonae	free (<5%)
Eudicotyledonae	free (<5%)
Commelinidae	free (<5%)
Pentapetalae	free (<5%)
Superasteridae	free (<5%)
Asteridae	free (<5%)
Lamiidae	free (<5%) / fused (>5%)
Campanulidae	fused (>5%)
Superrosidae	free (<5%)
Rosidae	free (<5%)
Malvidae	free (<5%)
Fabidae	free (<5%)

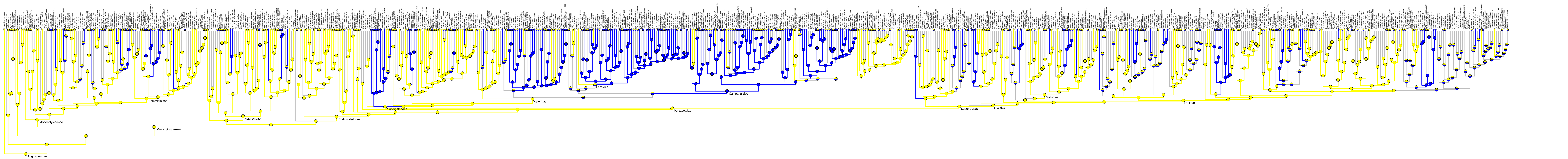

ML ancestral state reconstruction using rayDISC (R:corHMM)
204_A. Fusion of perianth (D2c), ER model

- free (<5%)
- fused (>5%)

Node	ML state	Prob
Angiospermae	free (<5%)	0.9331
Mesangiospermae	free (<5%)	0.9997
Magnoliidae	free (<5%)	0.9986
Monocotyledonae	free (<5%)	0.9986
Eudicotyledonae	free (<5%)	0.9998
Commelinidae	free (<5%)	0.9958
Pentapetalae	free (<5%)	0.9941
Superasteridae	free (<5%)	0.9599
Asteridae	free (<5%)	0.7092
Lamiidae	fused (>5%)	0.9799
Campanulidae	fused (>5%)	0.9731
Superrosidae	free (<5%)	0.995
Rosidae	free (<5%)	0.9954
Malvidae	free (<5%)	0.9965
Fabidae	free (<5%)	0.9996

Model	LogL	Npar	AIC	AICc	DeltaAICc	w	q01	q10
ARD	−225.91	2	455.81	455.83	1.78	0.19	0.0042	0.0037
ARDeq	−225.33	2	454.65	454.67	0.62	0.34	0.0042	0.0037
ER*	−226.02	1	454.04	454.05	0	0.47	0.0041	0.0041
UNI01	−238.92	1	479.85	479.85	25.81	0	0.0046	
UNI10	−235.97	1	473.95	473.95	19.91	0		0.0077

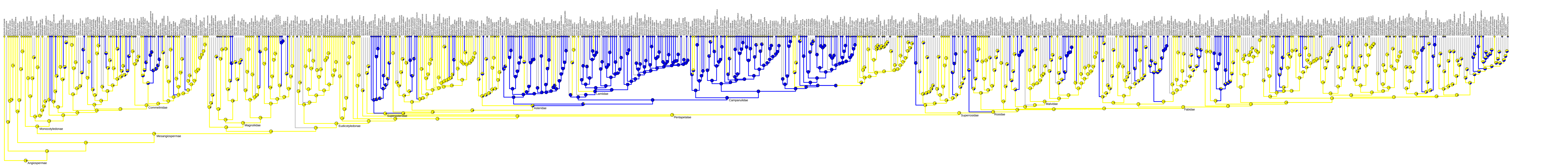

MP ancestral state reconstruction using ancestral.pars
(R:phangorn)
207_A. Symmetry of perianth (binary) (D2d), 55 steps

- actinomorphic
- zygomorphic

Node	MP state(s)
Angiospermae	actinomorphic
Mesangiospermae	actinomorphic
Magnoliidae	actinomorphic
Monocotyledonae	actinomorphic
Eudicotyledonae	actinomorphic
Commelinidae	actinomorphic
Pentapetalae	actinomorphic
Superasteridae	actinomorphic
Asteridae	actinomorphic
Lamiidae	actinomorphic
Campanulidae	actinomorphic
Superrosidae	actinomorphic
Rosidae	actinomorphic
Malvidae	actinomorphic
Fabidae	actinomorphic

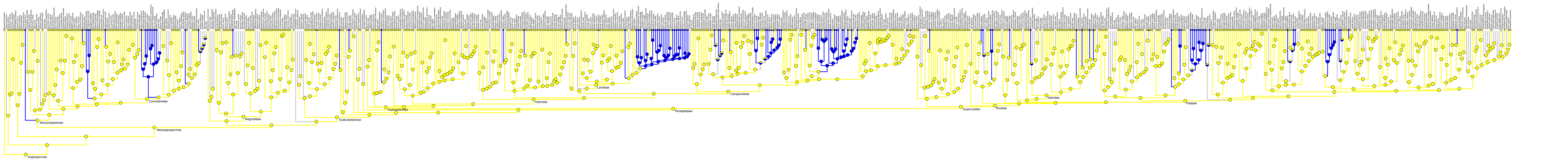

ML ancestral state reconstruction using rayDISC (R:corHMM)
207_A. Symmetry of perianth (binary) (D2d), ARDeq model

- actinomorphic
- zygomorphic

Node	ML state	Prob
Angiospermae	actinomorphic	0.9989
Mesangiospermae	actinomorphic	0.9997
Magnoliidae	actinomorphic	0.9998
Monocotyledonae	actinomorphic	0.9961
Eudicotyledonae	actinomorphic	0.9997
Commelinidae	actinomorphic	0.9951
Pentapetalae	actinomorphic	1
Superasteridae	actinomorphic	1
Asteridae	actinomorphic	1
Lamiidae	actinomorphic	0.9978
Campanulidae	actinomorphic	1
Superrosidae	actinomorphic	1
Rosidae	actinomorphic	1
Malvidae	actinomorphic	0.9997
Fabidae	actinomorphic	0.9999

Model	LogL	Npar	AIC	AICc	DeltaAICc	w	q01	q10
ARD	−209.31	2	422.62	422.63	1	0.38	0.0013	0.0058
ARDeq**	−208.81	2	421.62	421.63	0	0.62	0.0013	0.0056
ER	−217.31	1	436.62	436.63	15	0	0.0015	0.0015
UNI01	−263.32	1	528.64	528.65	107.01	0	0.0018	
UNI10	−218.34	1	438.68	438.69	17.06	0		0.0155

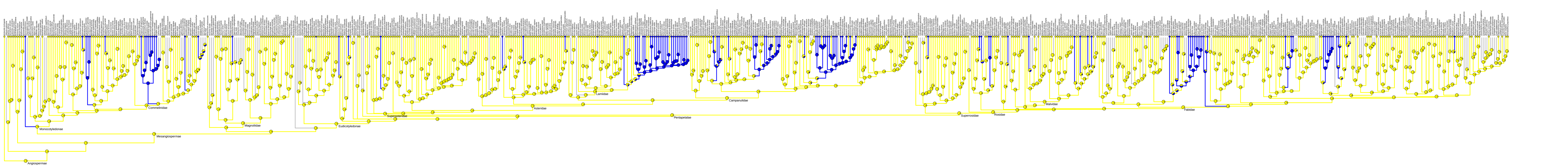

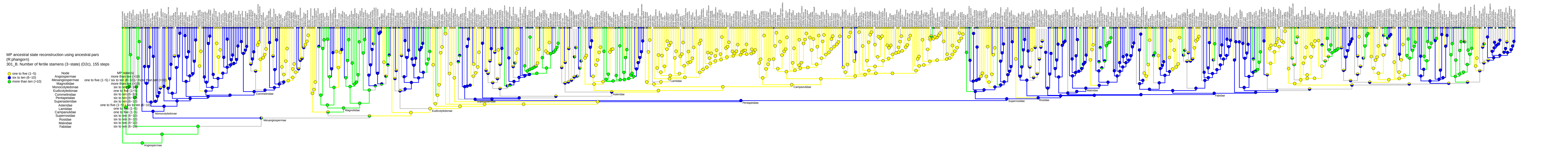

MP ancestral state reconstruction using ancestral.pars
(R:phangorn)
301_B. Number of fertile stamens (3−state) (D2c), 155 steps
one to five (1−5)
six to ten (6−10)
more than ten (>10)
Node | MP state(s)
Angiospermae | more than ten (>10)
Mesangiospermae | one to five (1−5) / six to ten (6−10) / more than ten (>10)
Magnoliidae | more than ten (>10)
Monocotyledonae | six to ten (6−10)
Eudicotyledonae | one to five (1−5)
Commelinidae | six to ten (6−10)
Pentapetalae | six to ten (6−10)
Superasteridae | six to ten (6−10)
Asteridae | one to five (1−5) / six to ten (6−10)
Lamiidae | one to five (1−5)
Campanulidae | one to five (1−5)
Superrosidae | six to ten (6−10)
Rosidae | six to ten (6−10)
Malvidae | six to ten (6−10)
Fabidae | six to ten (6−10)
Monocotyledonae
Commelinidae
Mesangiospermae
Angiospermae
Magnoliidae
Eudicotyledonae
Superasteridae
Asteridae
Lamiidae
Campanulidae
Pentapetalae
Superrosidae
Rosidae
Malvidae
Fabidae

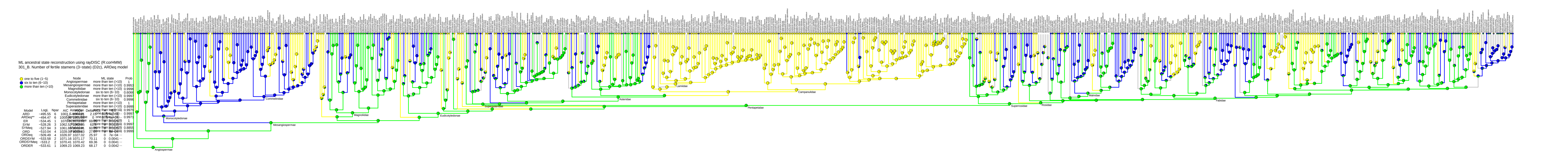
ML ancestral state reconstruction using rayDISC (R:corHMM)
301_B. Number of fertile stamens (3−state) (D2c), ARDeq model
one to five (1−5)
six to ten (6−10)
more than ten (>10)
Node ML state Prob
Angiospermae more than ten (>10) 1
Mesangiospermae more than ten (>10) 0.9953
Magnoliidae more than ten (>10) 0.9996
Monocotyledonae six to ten (6−10) 0.6066
Eudicotyledonae more than ten (>10) 0.9997
Commelinidae six to ten (6−10) 0.9996
Pentapetalae more than ten (>10) 1
Superasteridae more than ten (>10) 0.9999
Asteridae more than ten (>10) 0.9979
Lamiidae one to five (1−5) 0.9997
Campanulidae one to five (1−5) 0.9971
Superrosidae more than ten (>10) 1
Rosidae more than ten (>10) 0.9997
Malvidae more than ten (>10) 0.8855
Fabidae more than ten (>10) 0.9999
Model LogL Npar AIC AICc DeltaAICc w q01 ···
ARD −495.55 6 1003.1 1003.21 2.15 0.25 4e−04 ···
ARDeq** −494.47 6 1000.93 1001.06 0 0.75 4e−04 ···
ER −534.45 1 1070.9 1070.91 69.85 0 0.0026 ···
SYM −528.26 3 1062.52 1062.55 61.5 0 0.0035 ···
SYMeq −527.94 3 1061.88 1061.91 60.85 0 0.0035 ···
ORD −510.04 4 1028.08 1028.13 27.07 0 8e−04 ···
ORDeq −509.49 4 1026.97 1027.02 25.97 0 7e−04 ···
ORDSYM −533.58 2 1071.16 1071.17 70.11 0 0.0041 ···
ORDSYMeq −533.2 2 1070.41 1070.42 69.36 0 0.0041 ···
ORDER −533.61 1 1069.23 1069.23 68.17 0 0.0042 ···
Commelinidae
Monocotyledonae
Magnoliidae
Mesangiospermae
Eudicotyledonae
Superasteridae
Pentapetalae
Lamiidae
Campanulidae
Asteridae
Superrosidae
Rosidae
Malvidae
Fabidae
Angiospermae

MP ancestral state reconstruction using ancestral.pars
(R:phangorn)
301_C. Number of fertile stamens (binary) (D2c), 93 steps

- one to six (1−6)
- more than six (>6)

Node	MP state(s)
Angiospermae	more than six (>6)
Mesangiospermae	one to six (1−6)
Magnoliidae	one to six (1−6) / more than six (>6)
Monocotyledonae	one to six (1−6)
Eudicotyledonae	one to six (1−6)
Commelinidae	one to six (1−6)
Pentapetalae	more than six (>6)
Superasteridae	more than six (>6)
Asteridae	one to six (1−6) / more than six (>6)
Lamiidae	one to six (1−6)
Campanulidae	one to six (1−6)
Superrosidae	more than six (>6)
Rosidae	more than six (>6)
Malvidae	more than six (>6)
Fabidae	more than six (>6)

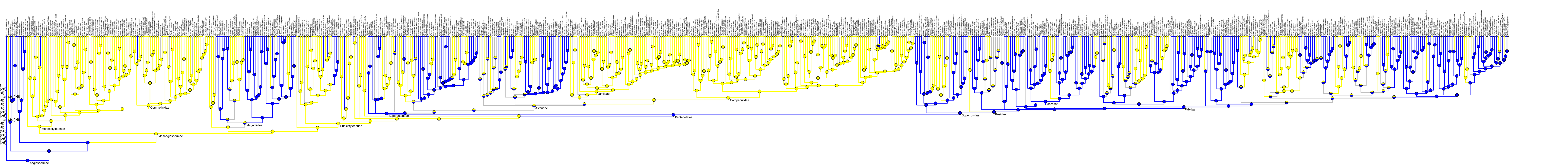

ML ancestral state reconstruction using rayDISC (R:corHMM)
301_C. Number of fertile stamens (binary) (D2c), ARDeq model

- one to six (1−6)
- more than six (>6)

Node	ML state	Prob
Angiospermae	more than six (>6)	1
Mesangiospermae	more than six (>6)	0.9977
Magnoliidae	more than six (>6)	1
Monocotyledonae	one to six (1−6)	0.605
Eudicotyledonae	more than six (>6)	0.9988
Commelinidae	one to six (1−6)	0.9999
Pentapetalae	more than six (>6)	1
Superasteridae	more than six (>6)	1
Asteridae	more than six (>6)	0.9999
Lamiidae	one to six (1−6)	0.9992
Campanulidae	one to six (1−6)	0.9951
Superrosidae	more than six (>6)	1
Rosidae	more than six (>6)	0.9999
Malvidae	more than six (>6)	1
Fabidae	more than six (>6)	1

Model	LogL	Npar	AIC	AICc	DeltaAICc	w	q01	q10
ARD	−289.19	2	582.37	[illegible]	1.38	0.33	5e−04	0.0055
ARDeq**	−288.5	2	580.99	581.01	0	0.67	5e−04	0.0055
ER	−321.11	1	644.23	644.23	63.22	0	0.003	0.003
UNI01	−365.05	1	732.1	732.1	151.09	0	0.0044	
UNI10	−326.48	1	654.97	654.97	73.96	0		0.0069

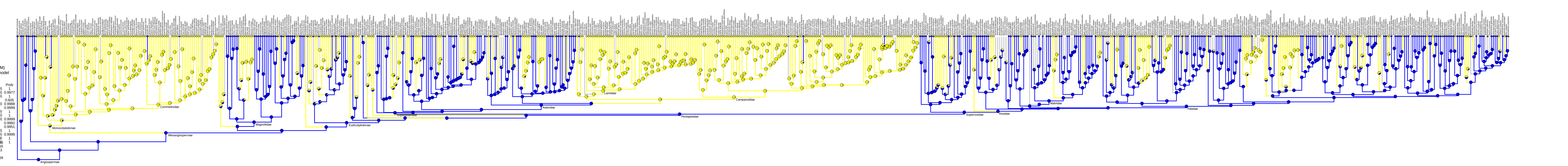

MP ancestral state reconstruction using ancestral.pars (R:phangorn)
330_A. Androecium structural phyllotaxy (binary) (D2d), 12 steps

- whorled
- spiral

Node	MP state(s)
Angiospermae	whorled / spiral
Mesangiospermae	whorled
Magnoliidae	whorled
Monocotyledonae	whorled
Eudicotyledonae	whorled
Commelinidae	whorled
Pentapetalae	whorled
Superasteridae	whorled
Asteridae	whorled
Lamiidae	whorled
Campanulidae	whorled
Superrosidae	whorled
Rosidae	whorled
Malvidae	whorled
Fabidae	whorled

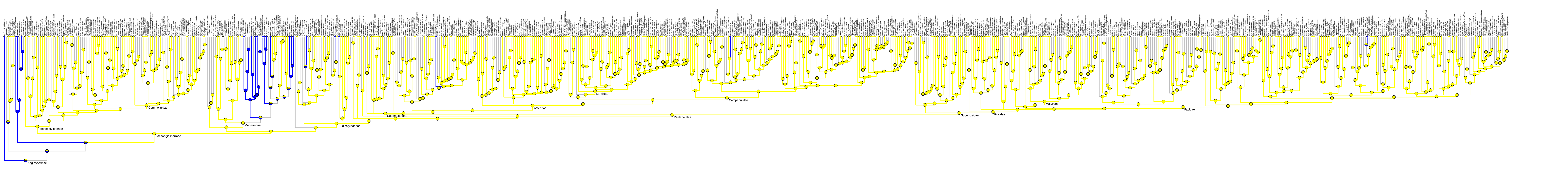

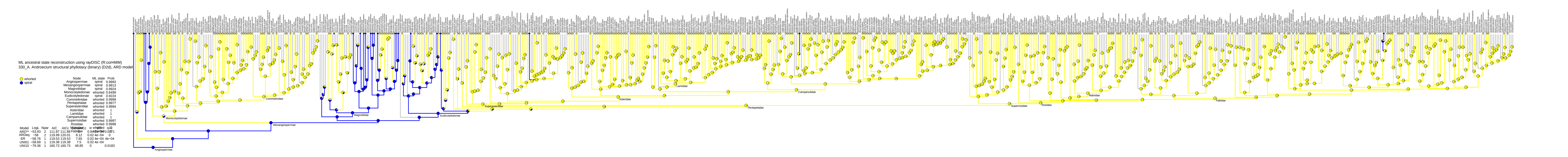
ML ancestral state reconstruction using rayDISC (R:corHMM)
330_A. Androecium structural phyllotaxy (binary) (D2d), ARD model
whorled
spiral
Node ML state Prob
Angiospermae spiral 0.9993
Mesangiospermae spiral 0.9913
Magnoliidae spiral 0.9924
Monocotyledonae whorled 0.6499
Eudicotyledonae spiral 0.9224
Commelinidae whorled 0.9999
Pentapetalae whorled 0.9977
Superasteridae whorled 0.9994
Asteridae whorled 1
Lamiidae whorled 1
Campanulidae whorled 1
Superrosidae whorled 0.9997
Rosidae whorled 0.9998
Malvidae whorled 1
Fabidae whorled 1
Model LogL Npar AIC AICc DeltaAICc w q01 q10
ARD** −53.93 2 111.87 111.88 0 0.94 2e−04 0.0071
ARDeq −58 2 119.99 120.01 8.12 0.02 4e−04 0
ER −58.76 1 119.53 119.53 7.65 0.02 4e−04 4e−04
UNI01 −58.69 1 119.38 119.38 7.5 0.02 4e−04
UNI10 −79.36 1 160.73 160.73 48.85 0 0.0183
Angiospermae
Mesangiospermae
Monocotyledonae
Commelinidae
Magnoliidae
Eudicotyledonae
Superasteridae
Pentapetalae
Asteridae
Lamiidae
Campanulidae
Superrosidae
Rosidae
Malvidae
Fabidae

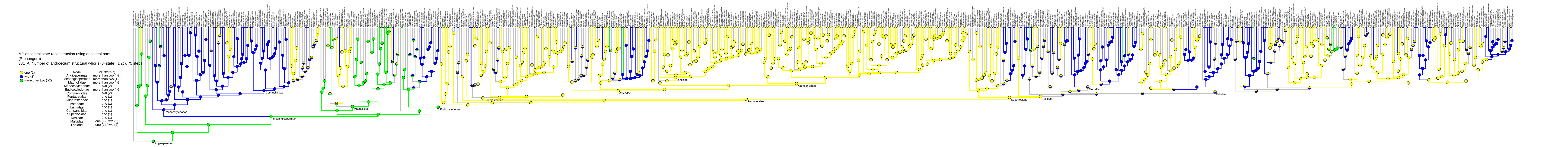
MP ancestral state reconstruction using ancestral.pars
(R:phangorn)
331_A. Number of androecium structural whorls (3−state) (D2c), 75 steps
one (1)
two (2)
more than two (>2)
Node | MP state(s)
Angiospermae | more than two (>2)
Mesangiospermae | more than two (>2)
Magnoliidae | more than two (>2)
Monocotyledonae | two (2)
Eudicotyledonae | more than two (>2)
Commelinidae | two (2)
Pentapetalae | one (1)
Superasteridae | one (1)
Asteridae | one (1)
Lamiidae | one (1)
Campanulidae | one (1)
Superrosidae | one (1)
Rosidae | one (1)
Malvidae | one (1) / two (2)
Fabidae | one (1) / two (2)
Commelinidae
Monocotyledonae
Magnoliidae
Mesangiospermae
Eudicotyledonae
Angiospermae
Superasteridae
Lamiidae
Asteridae
Campanulidae
Pentapetalae
Superrosidae
Rosidae
Malvidae
Fabidae

ML ancestral state reconstruction using rayDISC (R:corHMM)
331_A. Number of androecium structural whorls (3−state) (D2c), ARDeq model

- one (1)
- two (2)
- more than two (>2)

Node	ML state	Prob
Angiospermae	more than two (>2)	1
Mesangiospermae	more than two (>2)	0.9874
Magnoliidae	more than two (>2)	0.9889
Monocotyledonae	more than two (>2)	0.8839
Eudicotyledonae	more than two (>2)	0.8062
Commelinidae	two (2)	0.9733
Pentapetalae	two (2)	0.9932
Superasteridae	two (2)	0.9973
Asteridae	two (2)	0.9999
Lamiidae	one (1)	0.999
Campanulidae	one (1)	0.9935
Superrosidae	two (2)	0.9987
Rosidae	two (2)	0.9993
Malvidae	two (2)	0.9997
Fabidae	two (2)	1

Model	LogL	Npar	AIC	AICc	DeltaAICc	w	q01	...
ARD	−231.14	6	474.28	474.38	2.1	0.16	0	...
ARDeq*	−230.09	6	472.18	472.28	0	0.44	0	...
ER	−278.89	1	559.78	559.78	87.5	0	0.0017	...
SYM	−254.54	3	515.08	515.11	42.83	0	0.0033	...
SYMeq	−253.87	3	513.73	513.76	41.48	0	0.0033	...
ORD	−233.57	4	475.15	475.2	2.91	0.1	0	...
ORDeq	−232.53	4	473.05	473.1	0.82	0.3	0	...
ORDSYM	−255.21	2	514.43	514.44	42.16	0	0.0034	...
ORDSYMeq	−254.47	2	512.94	512.95	40.67	0	0.0034	...
ORDER	−260.43	1	522.85	522.86	50.57	0	0.0027	...

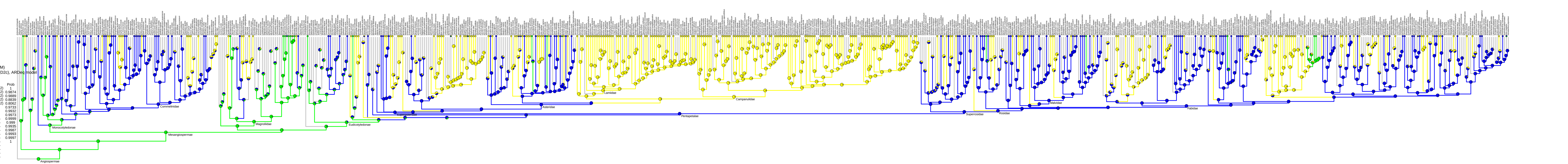

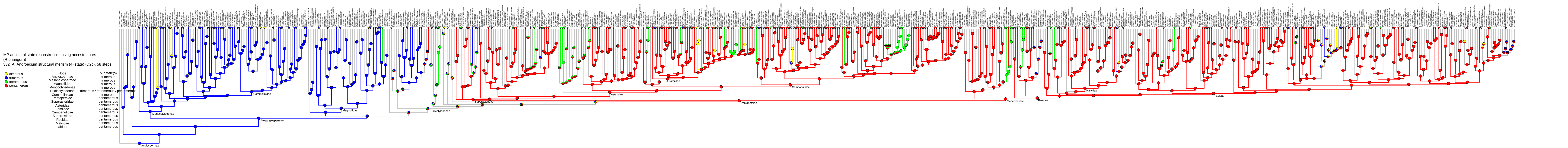
MP ancestral state reconstruction using ancestral.pars
(R:phangorn)
332_A. Androecium structural merism (4−state) (D2c), 58 steps
dimerous
trimerous
tetramerous
pentamerous
Node
Angiospermae
Mesangiospermae
Magnoliidae
Monocotyledonae
Eudicotyledonae
Commelinidae
Pentapetalae
Superasteridae
Asteridae
Lamiidae
Campanulidae
Superrosidae
Rosidae
Malvidae
Fabidae
MP state(s)
trimerous
trimerous
trimerous
trimerous
trimerous / tetramerous / pentamerous
trimerous
pentamerous
pentamerous
pentamerous
pentamerous
pentamerous
pentamerous
pentamerous
pentamerous
pentamerous

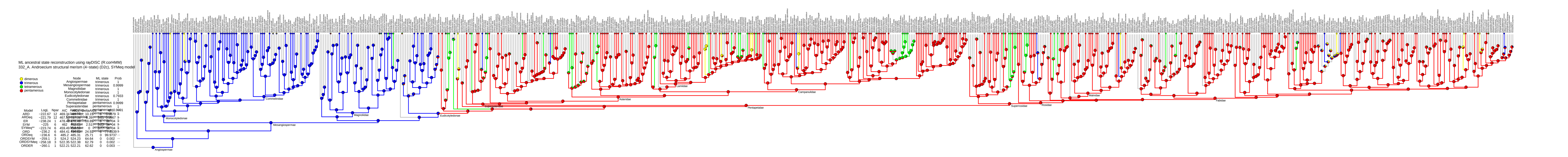
ML ancestral state reconstruction using rayDISC (R:corHMM)
332_A. Androecium structural merism (4−state) (D2c), SYMeq model
dimerous
trimerous
tetramerous
pentamerous
Node | ML state | Prob
Angiospermae | trimerous | 1
Mesangiospermae | trimerous | 0.9999
Magnoliidae | trimerous | 1
Monocotyledonae | trimerous | 1
Eudicotyledonae | trimerous | 0.7933
Commelinidae | trimerous | 1
Pentapetalae | pentamerous | 0.9999
Superasteridae | pentamerous | 1
Asteridae | pentamerous | 0.9991
Lamiidae | pentamerous | 1
Campanulidae | pentamerous | 1
Superrosidae | pentamerous | 1
Rosidae | pentamerous | 1
Malvidae | pentamerous | 1
Fabidae | pentamerous | 1
Model | LogL | Npar | AIC | AICc | DeltaAICc | w | q01
ARD | −222.67 | 12 | 469.34 | 469.74 | 10.15 | 0 | 0.0079 | ...
ARDeq | −221.79 | 12 | 467.58 | 467.98 | 8.39 | 0.01 | 0.0067 | ...
ER | −238.24 | 1 | 478.48 | 478.48 | 18.89 | 0 | 8e−04 | ...
SYM | −225 | 6 | 462 | 462.11 | 2.51 | 0.22 | 5e−04 | ...
SYMeq** | −223.74 | 6 | 459.49 | 459.59 | 0 | 0.77 | 5e−04 | ...
ORD | −236.2 | 6 | 484.41 | 484.52 | 24.92 | 0 | 77.5339 | ...
ORDeq | −236.6 | 6 | 485.2 | 485.31 | 25.71 | 0 | 99.9737 | ...
ORDSYM | −259.1 | 3 | 524.2 | 524.23 | 64.64 | 0 | 0.002 | ...
ORDSYMeq | −258.18 | 3 | 522.35 | 522.38 | 62.79 | 0 | 0.002 | ...
ORDER | −260.1 | 1 | 522.21 | 522.21 | 62.62 | 0 | 0.003 | ...
Angiospermae
Mesangiospermae
Monocotyledonae
Commelinidae
Magnoliidae
Eudicotyledonae
Pentapetalae
Asteridae
Lamiidae
Campanulidae
Superrosidae
Rosidae
Malvidae
Fabidae

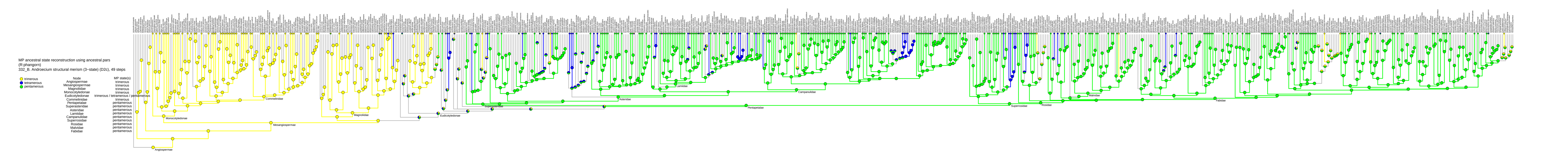
MP ancestral state reconstruction using ancestral.pars
(R:phangorn)
332_B. Androecium structural merism (3−state) (D2c), 49 steps
trimerous
tetramerous
pentamerous
Node
Angiospermae
Mesangiospermae
Magnoliidae
Monocotyledonae
Eudicotyledonae
Commelinidae
Pentapetalae
Superasteridae
Asteridae
Lamiidae
Campanulidae
Superrosidae
Rosidae
Malvidae
Fabidae
MP state(s)
trimerous
trimerous
trimerous
trimerous
trimerous / tetramerous / pentamerous
trimerous
pentamerous
pentamerous
pentamerous
pentamerous
pentamerous
pentamerous
pentamerous
pentamerous
pentamerous
Angiospermae
Mesangiospermae
Monocotyledonae
Commelinidae
Magnoliidae
Eudicotyledonae
Superasteridae
Asteridae
Lamiidae
Campanulidae
Pentapetalae
Superrosidae
Rosidae
Malvidae
Fabidae

ML ancestral state reconstruction using rayDISC (R:corHMM)
332_B. Androecium structural merism (3−state) (D2c), ARDeq model

- trimerous
- tetramerous
- pentamerous

Node	ML state	Prob
Angiospermae	trimerous	0.9999
Mesangiospermae	trimerous	1
Magnoliidae	trimerous	1
Monocotyledonae	trimerous	1
Eudicotyledonae	trimerous	0.8316
Commelinidae	trimerous	1
Pentapetalae	tetramerous	0.9999
Superasteridae	tetramerous	1
Asteridae	tetramerous	1
Lamiidae	tetramerous	1
Campanulidae	tetramerous	1
Superrosidae	tetramerous	1
Rosidae	tetramerous	1
Malvidae	pentamerous	0.5273
Fabidae	tetramerous	0.9994

Model	LogL	Npar	AIC	AICc	DeltaAICc	w	q31	···
ARD	−170.59	6	353.18	353.29	1.98	0.26	8e−04	···
ARDeq**	−169.6	6	351.2	351.31	0	0.7	8e−04	···
ER	−186.71	1	375.42	375.42	24.12	0	0.0011	···
SYM	−176.92	3	359.84	359.87	8.57	0.01	4e−04	···
SYMeq	−175.92	3	357.83	357.86	6.55	0.03	4e−04	···
ORD	−178.03	4	364.05	364.11	12.8	0	6e−04	···
ORDeq	−177.5	4	362.99	363.05	11.74	0	2e−04	···
ORDSYM	−185.53	2	375.06	375.07	23.77	0	0.0019	···
ORDSYMeq	−184.62	2	373.24	373.26	21.95	0	0.0019	···
ORDER	−186.47	1	374.94	374.94	23.64	0	0.0026	···

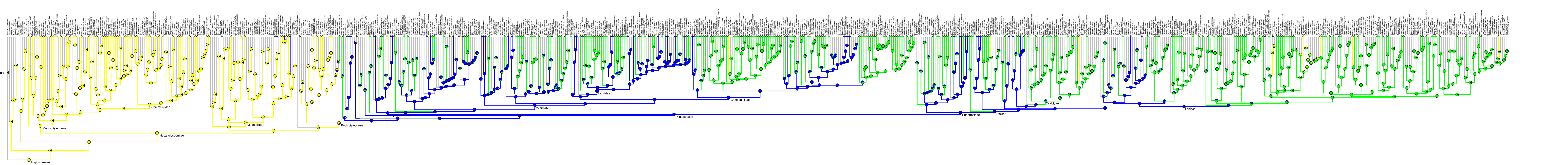

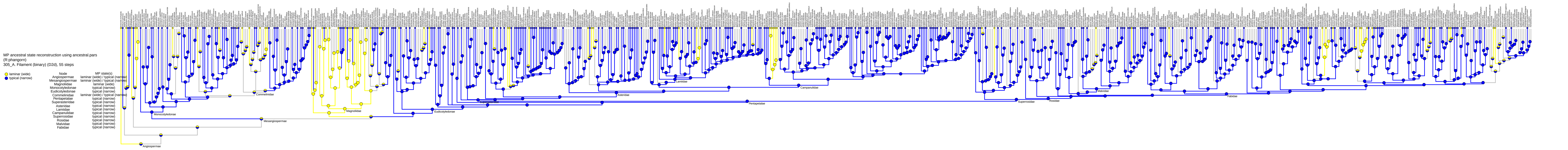
MP ancestral state reconstruction using ancestral.pars
(R:phangorn)
305_A. Filament (binary) (D2d), 55 steps
laminar (wide)
typical (narrow)
Node
MP state(s)
Angiospermae laminar (wide) / typical (narrow)
Mesangiospermae laminar (wide) / typical (narrow)
Magnoliidae laminar (wide)
Monocotyledonae typical (narrow)
Eudicotyledonae typical (narrow)
Commelinidae laminar (wide) / typical (narrow)
Pentapetalae typical (narrow)
Superasteridae typical (narrow)
Asteridae typical (narrow)
Lamiidae typical (narrow)
Campanulidae typical (narrow)
Superrosidae typical (narrow)
Rosidae typical (narrow)
Malvidae typical (narrow)
Fabidae typical (narrow)
Commelinidae
Monocotyledonae
Mesangiospermae
Angiospermae
Magnoliidae
Eudicotyledonae
Lamiidae
Asteridae
Campanulidae
Pentapetalae
Superrosidae
Rosidae
Malvidae
Fabidae

ML ancestral state reconstruction using rayDISC (R:corHMM)
305_A. Filament (binary) (D2d), ARDeq model

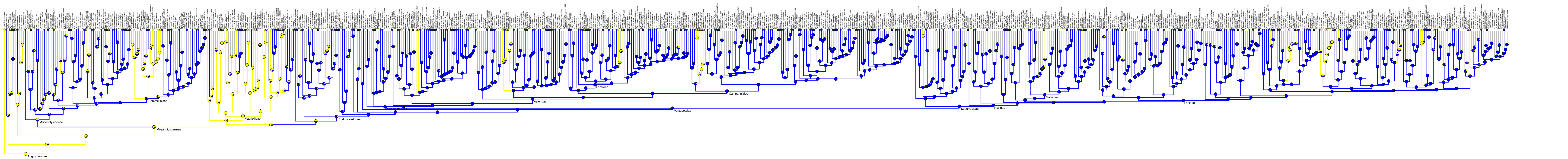

Node	ML state	Prob
Angiospermae	laminar (wide)	0.9909
Mesangiospermae	laminar (wide)	0.8185
Magnoliidae	laminar (wide)	0.981
Monocotyledonae	typical (narrow)	0.5102
Eudicotyledonae	typical (narrow)	0.8083
Commelinidae	typical (narrow)	0.7418
Pentapetalae	typical (narrow)	0.9996
Superasteridae	typical (narrow)	0.9998
Asteridae	typical (narrow)	0.9997
Lamiidae	typical (narrow)	0.9989
Campanulidae	typical (narrow)	0.9959
Superrosidae	typical (narrow)	0.9998
Rosidae	typical (narrow)	0.9998
Malvidae	typical (narrow)	0.9981
Fabidae	typical (narrow)	1

Model	LogL	Npar	AIC	AICc	DeltaAICc	w	q01	q10
ARD	−172.12	2	348.24	348.26	0.67	0.42	0.0072	0.0021
ARDeq**	−171.79	2	347.57	347.59	0	0.58	0.0074	0.0021
ER	−177.98	1	357.96	357.97	10.38	0	0.0023	0.0023
UNI01	−189.97	1	381.95	381.95	34.36	0	0.0136	
UNI10	−189.94	1	381.87	381.88	34.29	0		0.0022

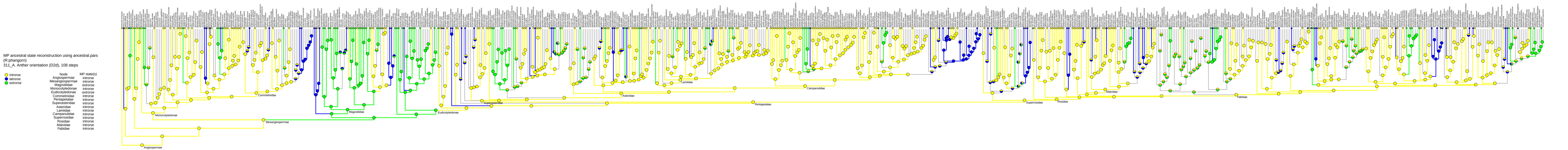
MP ancestral state reconstruction using ancestral.pars
(R:phangorn)
311_A. Anther orientation (D2d), 108 steps
introrse
latrorse
extrorse
Node
MP state(s)
Angiospermae introrse
Mesangiospermae introrse
Magnoliidae extrorse
Monocotyledonae introrse
Eudicotyledonae extrorse
Commelinidae introrse
Pentapetalae introrse
Superasteridae introrse
Asteridae introrse
Lamiidae introrse
Campanulidae introrse
Superrosidae introrse
Rosidae introrse
Malvidae introrse
Fabidae introrse
Commelinidae
Monocotyledonae
Mesangiospermae
Angiospermae
Magnoliidae
Eudicotyledonae
Superasteridae
Lamiidae
Asteridae
Campanulidae
Pentapetalae
Superrosidae
Rosidae
Malvidae
Fabidae

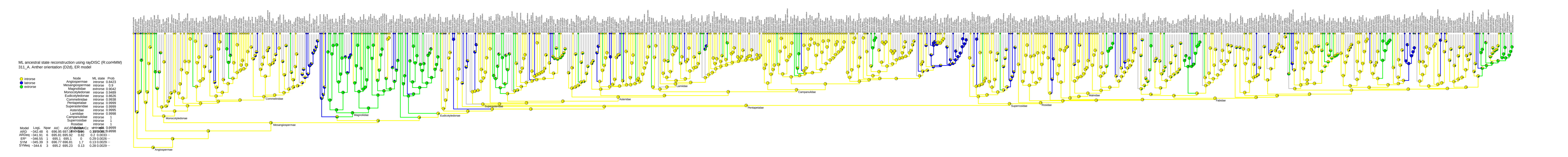
ML ancestral state reconstruction using rayDISC (R:corHMM)
311_A. Anther orientation (D2d), ER model
introrse
latrorse
extrorse
Node ML state Prob
Angiospermae introrse 0.8423
Mesangiospermae introrse 0.9
Magnoliidae extrorse 0.9042
Monocotyledonae introrse 0.9489
Eudicotyledonae introrse 0.8626
Commelinidae introrse 0.9938
Pentapetalae introrse 0.9999
Superasteridae introrse 0.9999
Asteridae introrse 0.9995
Lamiidae introrse 0.9998
Campanulidae introrse 1
Superrosidae introrse 1
Rosidae introrse 1
Malvidae introrse 0.9999
Fabidae introrse 0.9998
Model LogL Npar AIC AICc DeltaAICc w
ARD −342.48 6 696.95 697.06 1.96 0.11 0.0017 ···
ARDeq −341.91 6 695.81 695.92 0.82 0.2 0.0033 ···
ER* −346.55 1 695.1 695.1 0 0.29 0.0026 ···
SYM −345.39 3 696.77 696.81 1.7 0.13 0.0029 ···
SYMeq −344.6 3 695.2 695.23 0.13 0.28 0.0029 ···
Angiospermae
Mesangiospermae
Monocotyledonae
Commelinidae
Magnoliidae
Eudicotyledonae
Superasteridae
Pentapetalae
Asteridae
Lamiidae
Campanulidae
Superrosidae
Rosidae
Malvidae
Fabidae

MP ancestral state reconstruction using ancestral.pars
(R:phangorn)
312_A. Anther attachment (binary) (D2d), 96 steps

- basifixed (yellow)
- dorsifixed (blue)

Node	MP state(s)
Angiospermae	basifixed
Mesangiospermae	basifixed
Magnoliidae	basifixed
Monocotyledonae	basifixed
Eudicotyledonae	basifixed
Commelinidae	basifixed
Pentapetalae	basifixed
Superasteridae	basifixed
Asteridae	basifixed
Lamiidae	basifixed
Campanulidae	basifixed / dorsifixed
Superrosidae	basifixed
Rosidae	dorsifixed
Malvidae	dorsifixed
Fabidae	dorsifixed

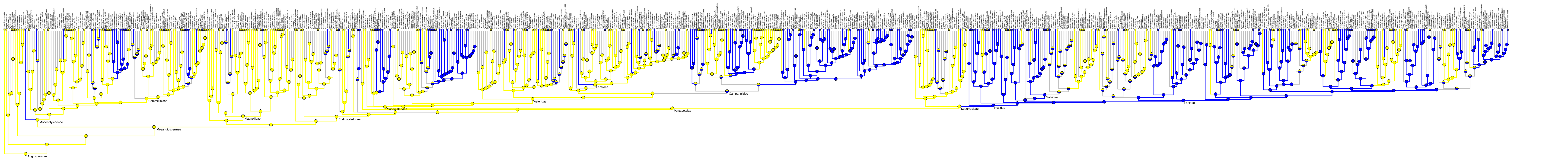

ML ancestral state reconstruction using rayDISC (R:corHMM)
312_A. Anther attachment (binary) (D2d), ARDeq model

- basifixed
- dorsifixed

Node	ML state	Prob
Angiospermae	basifixed	0.9767
Mesangiospermae	basifixed	0.941
Magnoliidae	basifixed	0.972
Monocotyledonae	basifixed	0.9235
Eudicotyledonae	basifixed	0.6727
Commelinidae	basifixed	0.9329
Pentapetalae	dorsifixed	0.9377
Superasteridae	dorsifixed	0.9445
Asteridae	dorsifixed	0.9264
Lamiidae	dorsifixed	0.7008
Campanulidae	dorsifixed	0.9812
Superrosidae	dorsifixed	0.9456
Rosidae	dorsifixed	0.9694
Malvidae	dorsifixed	0.9712
Fabidae	dorsifixed	0.9998

Model	LogL	Npar	AIC	AICc	DeltaAICc	wi	q01	q10
ARD	−264.88	2	533.76	533.77	0.51	0.4	0.004	0.0076
ARDeq**	−264.62	2	533.25	533.26	0	0.52	0.004	0.0074
ER	−267.47	1	536.94	536.94	3.68	0.08	0.0055	0.0055
UNI01	−295.34	1	592.68	592.69	59.43	0	0.0058	
UNI10	−276.74	1	555.48	555.48	22.22	0		0.0076

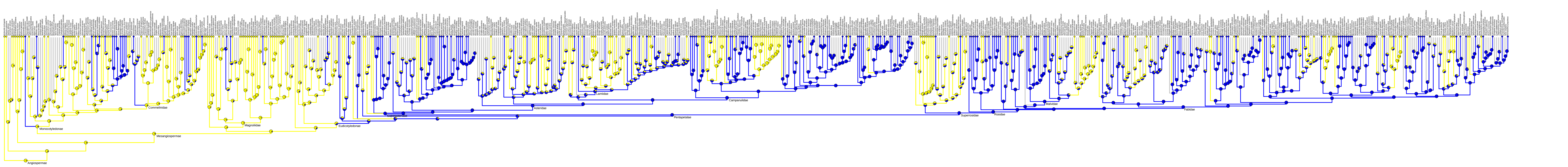

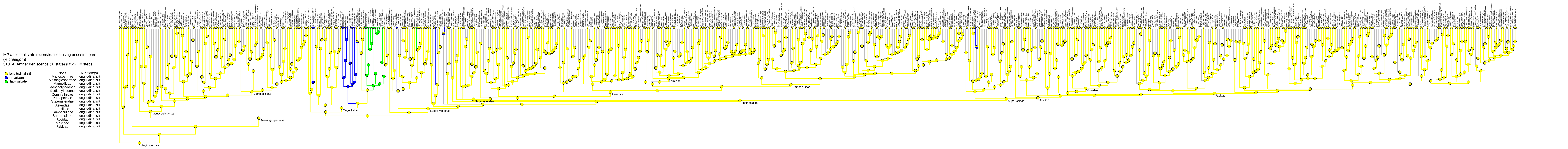
MP ancestral state reconstruction using ancestral.pars
(R:phangorn)
313_A. Anther dehiscence (3−state) (D2d), 10 steps
longitudinal slit
H−valvate
flap−valvate
Node
MP state(s)
Angiospermae longitudinal slit
Mesangiospermae longitudinal slit
Magnoliidae longitudinal slit
Monocotyledonae longitudinal slit
Eudicotyledonae longitudinal slit
Commelinidae longitudinal slit
Pentapetalae longitudinal slit
Superasteridae longitudinal slit
Asteridae longitudinal slit
Lamiidae longitudinal slit
Campanulidae longitudinal slit
Superrosidae longitudinal slit
Rosidae longitudinal slit
Malvidae longitudinal slit
Fabidae longitudinal slit
Commelinidae
Monocotyledonae
Mesangiospermae
Angiospermae
Magnoliidae
Eudicotyledonae
Superasteridae
Asteridae
Lamiidae
Campanulidae
Pentapetalae
Superrosidae
Rosidae
Malvidae
Fabidae

ML ancestral state reconstruction using rayDISC (R:corHMM)
313_A. Anther dehiscence (3−state) (D2d), ARDeq model

- longitudinal slit
- H−valvate
- flap−valvate

Node	ML state	Prob
Angiospermae	longitudinal slit	1
Mesangiospermae	longitudinal slit	1
Magnoliidae	longitudinal slit	0.9601
Monocotyledonae	longitudinal slit	1
Eudicotyledonae	longitudinal slit	1
Commelinidae	longitudinal slit	1
Pentapetalae	longitudinal slit	1
Superasteridae	longitudinal slit	1
Asteridae	longitudinal slit	1
Lamiidae	longitudinal slit	1
Campanulidae	longitudinal slit	1
Superrosidae	longitudinal slit	1
Rosidae	longitudinal slit	1
Malvidae	longitudinal slit	1
Fabidae	longitudinal slit	1

Model	LogL	Npar	AIC	AICc	DeltaAICc	w	q01	···
ARD	−55.64	6	123.27	123.38	2.05	0.16	2e−04	···
ARDeq*	−54.61	6	121.22	121.33	0	0.45	2e−04	···
ER	−60.71	1	123.41	123.42	2.08	0.16	2e−04	···
SYM	−59.7	3	125.41	125.44	4.11	0.06	2e−04	···
SYMeq	−58.61	3	123.21	123.24	1.91	0.17	2e−04	···

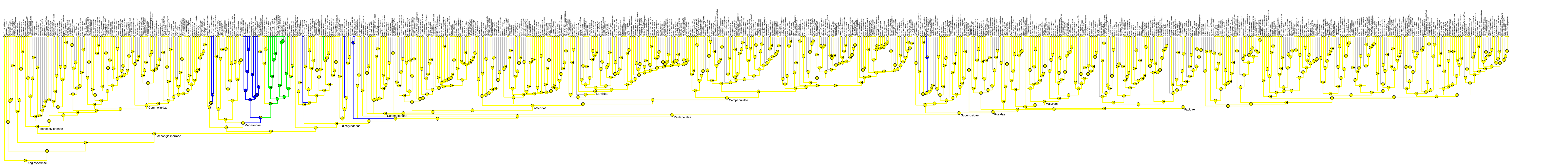

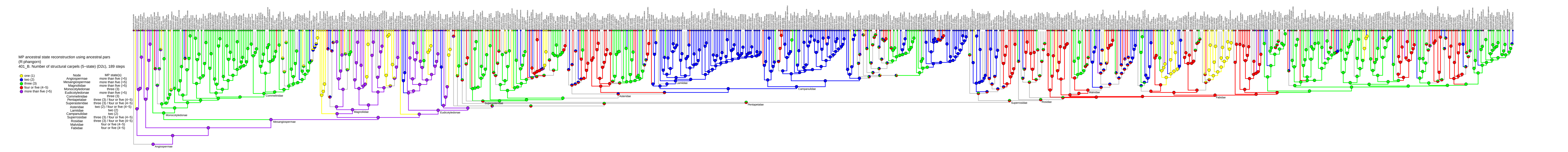
MP ancestral state reconstruction using ancestral.pars
(R:phangorn)
401_B. Number of structural carpels (5−state) (D2c), 189 steps
one (1)
two (2)
three (3)
four or five (4−5)
more than five (>5)
Node
MP state(s)
Angiospermae more than five (>5)
Mesangiospermae more than five (>5)
Magnoliidae more than five (>5)
Monocotyledonae three (3)
Eudicotyledonae more than five (>5)
Commelinidae three (3)
Pentapetalae three (3) / four or five (4−5)
Superasteridae three (3) / four or five (4−5)
Asteridae two (2) / four or five (4−5)
Lamiidae two (2)
Campanulidae two (2)
Superrosidae three (3) / four or five (4−5)
Rosidae three (3) / four or five (4−5)
Malvidae four or five (4−5)
Fabidae four or five (4−5)
Commelinidae
Monocotyledonae
Magnoliidae
Mesangiospermae
Angiospermae
Eudicotyledonae
Superasteridae
Asteridae
Lamiidae
Campanulidae
Pentapetalae
Superrosidae
Rosidae
Malvidae
Fabidae

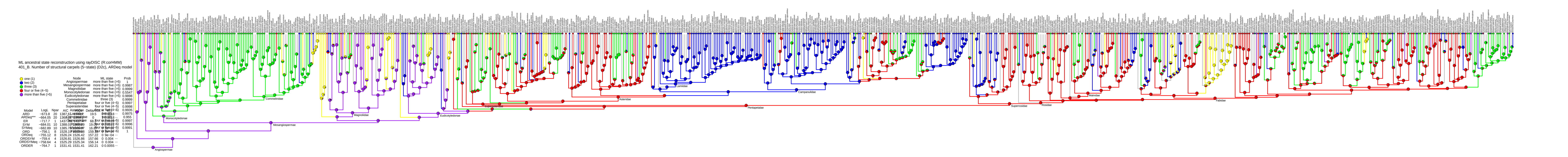
ML ancestral state reconstruction using rayDISC (R:corHMM)
401_B. Number of structural carpels (5−state) (D2c), ARDeq model
one (1)
two (2)
three (3)
four or five (4−5)
more than five (>5)
Node ML state Prob
Angiospermae more than five (>5) 1
Mesangiospermae more than five (>5) 0.9987
Magnoliidae more than five (>5) 0.9999
Monocotyledonae more than five (>5) 0.5547
Eudicotyledonae more than five (>5) 0.9898
Commelinidae three (3) 0.9999
Pentapetalae four or five (4−5) 0.9997
Superasteridae four or five (4−5) 0.9998
Asteridae four or five (4−5) 0.9935
Lamiidae two (2) 0.9975
Campanulidae two (2) 0.955
Superrosidae four or five (4−5) 0.9997
Rosidae four or five (4−5) 0.9996
Malvidae four or five (4−5) 0.9991
Fabidae four or five (4−5) 1
Model LogL Npar AIC AICc DeltaAICc w qe01
ARD −673.8 20 1387.61 19.5 0.0018 ···
ARDeq*** −664.05 20 1368.11 0 0.0018 ···
ER −717.7 1 1437.39 68.2 0.0016 ···
SYM −684.01 10 1388.01 19.09 0.0011 ···
SYMeq −682.89 10 1385.78 16.87 9e−04 ···
ORD −756.1 8 1528.19 159.18 9e−04 ···
ORDeq −755.12 8 1526.24 1526.42 157.22 0 9e−04 ···
ORDSYM −759.4 4 1526.81 1526.86 157.66 0 0.004 ···
ORDSYMeq −758.64 4 1525.29 1525.34 156.14 0 0.004 ···
ORDER −764.7 1 1531.41 1531.41 162.21 0 0.0055 ···
Commelinidae
Monocotyledonae
Magnoliidae
Mesangiospermae
Eudicotyledonae
Angiospermae
Asteridae
Lamiidae
Campanulidae
Pentapetalae
Superrosidae
Rosidae
Malvidae
Fabidae

MP ancestral state reconstruction using ancestral.pars
(R:phangorn)
400_A. Gynoecium phyllotaxy (D2d), 10 steps

- whorled
- spiral

Node	MP state(s)
Angiospermae	whorled / spiral
Mesangiospermae	whorled
Magnoliidae	whorled
Monocotyledonae	whorled
Eudicotyledonae	whorled
Commelinidae	whorled
Pentapetalae	whorled
Superasteridae	whorled
Asteridae	whorled
Lamiidae	whorled
Campanulidae	whorled
Superrosidae	whorled
Rosidae	whorled
Malvidae	whorled
Fabidae	whorled

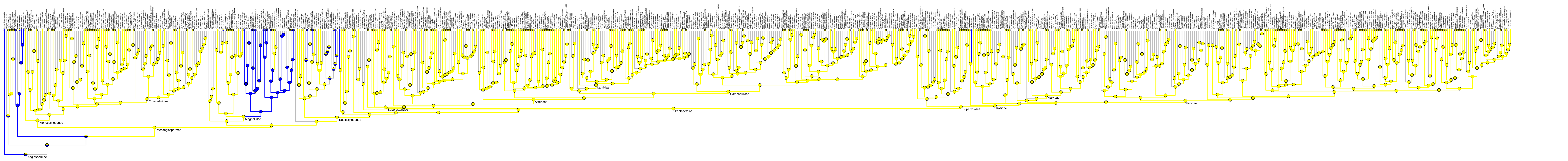

ML ancestral state reconstruction using rayDISC (R:corHMM)
400_A. Gynoecium phyllotaxy (D2d), ARDeq model

- whorled
- spiral

Node	ML state	Prob
Angiospermae	spiral	1
Mesangiospermae	spiral	0.9998
Magnoliidae	spiral	0.9997
Monocotyledonae	whorled	0.6307
Eudicotyledonae	spiral	0.9987
Commelinidae	whorled	0.9999
Pentapetalae	whorled	0.9748
Superasteridae	whorled	0.9943
Asteridae	whorled	1
Lamiidae	whorled	1
Campanulidae	whorled	1
Superrosidae	whorled	0.9786
Rosidae	whorled	0.9787
Malvidae	whorled	1
Fabidae	whorled	1

Model	LogL	Npar	AIC	AICc	DeltaAICc	w	q01	q10
ARD	−38.72	2	81.45	81.46	1.39	0.31	1e−04	0.0068
ARDeq**	−38.03	2	80.06	80.07	0	0.62	1e−04	0.0068
ER	−49.53	1	101.05	101.06	20.98	0	4e−04	4e−04
UNI01	−51.24	1	104.47	104.48	24.4	0	4e−04	
UNI10	−41.33	1	84.65	84.66	4.58	0.06		0.008

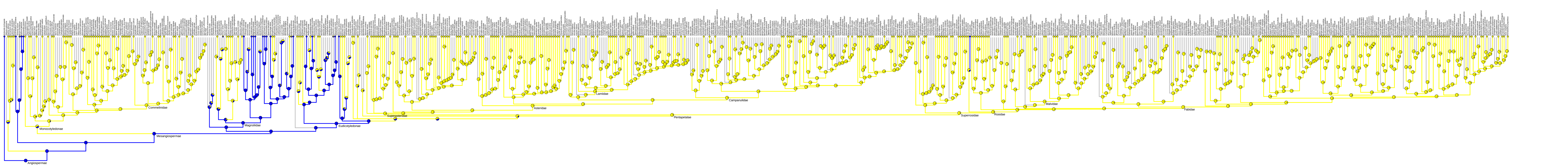

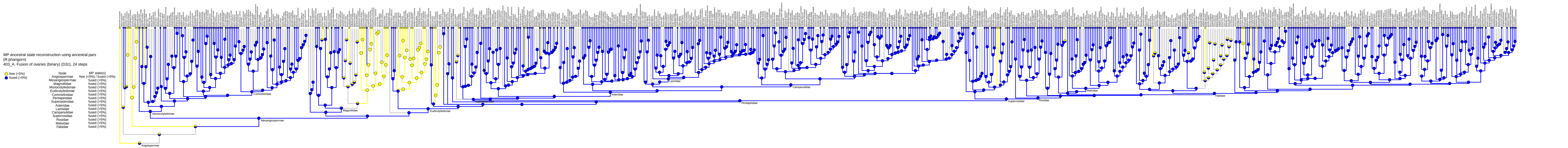
MP ancestral state reconstruction using ancestral.pars
(R:phangorn)
403_A. Fusion of ovaries (binary) (D2c), 24 steps
free (<5%)
fused (>5%)
Node
MP state(s)
Angiospermae
free (<5%) / fused (>5%)
Mesangiospermae
fused (>5%)
Magnoliidae
fused (>5%)
Monocotyledonae
fused (>5%)
Eudicotyledonae
fused (>5%)
Commelinidae
fused (>5%)
Pentapetalae
fused (>5%)
Superasteridae
fused (>5%)
Asteridae
fused (>5%)
Lamiidae
fused (>5%)
Campanulidae
fused (>5%)
Superrosidae
fused (>5%)
Rosidae
fused (>5%)
Malvidae
fused (>5%)
Fabidae
fused (>5%)
Commelinidae
Monocotyledonae
Mesangiospermae
Angiospermae
Magnoliidae
Eudicotyledonae
Lamiidae
Asteridae
Campanulidae
Pentapetalae
Superrosidae
Rosidae
Malvidae
Fabidae

ML ancestral state reconstruction using rayDISC (R:corHMM)
403_A. Fusion of ovaries (binary) (D2c), ARDeq model

- free (<5%)
- fused (>5%)

Node	ML state	Prob
Angiospermae	free (<5%)	1
Mesangiospermae	free (<5%)	0.9992
Magnoliidae	free (<5%)	0.9992
Monocotyledonae	free (<5%)	0.8142
Eudicotyledonae	free (<5%)	0.9956
Commelinidae	fused (>5%)	0.9999
Pentapetalae	fused (>5%)	0.9974
Superasteridae	fused (>5%)	0.9978
Asteridae	fused (>5%)	1
Lamiidae	fused (>5%)	1
Campanulidae	fused (>5%)	1
Superrosidae	fused (>5%)	0.9998
Rosidae	fused (>5%)	0.9999
Malvidae	fused (>5%)	1
Fabidae	fused (>5%)	0.9999

Model	LogL	Npar	AIC	AICc	DeltaAICc	w	q01	q10
ARD	−94.72	2	193.43	193.45	1.38	0.33	0.0045	5e−04
ARDeq**	−94.03	2	192.05	192.07	0	0.66	0.0045	5e−04
ER	−100.22	1	202.44	202.44	10.38	0	7e−04	7e−04
UNI01	−100.89	1	203.79	203.79	11.72	0	0.0102	
UNI10	−105.42	1	212.84	212.84	20.78	0		8e−04

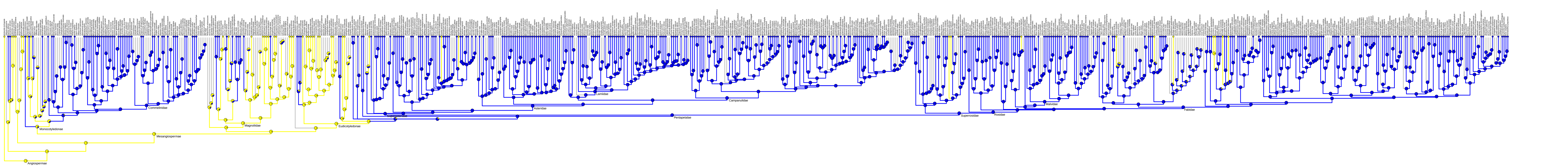

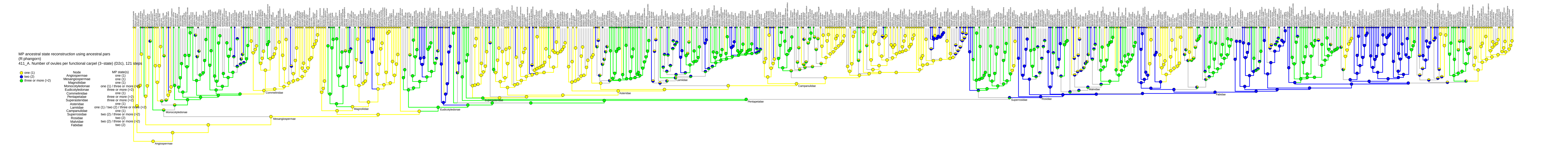
MP ancestral state reconstruction using ancestral.pars
(R:phangorn)
411_A. Number of ovules per functional carpel (3−state) (D2c), 121 steps
one (1)
two (2)
three or more (>2)
Node
MP state(s)
Angiospermae
one (1)
Mesangiospermae
one (1)
Magnoliidae
one (1)
Monocotyledonae
one (1) / three or more (>2)
Eudicotyledonae
three or more (>2)
Commelinidae
one (1)
Pentapetalae
three or more (>2)
Superasteridae
three or more (>2)
Asteridae
one (1)
Lamiidae
one (1) / two (2) / three or more (>2)
Campanulidae
one (1)
Superrosidae
two (2) / three or more (>2)
Rosidae
two (2)
Malvidae
two (2) / three or more (>2)
Fabidae
two (2)

ML ancestral state reconstruction using rayDISC (R:corHMM)
411_A. Number of ovules per functional carpel (3−state) (D2c), ARDeq model

- one (1)
- two (2)
- three or more (>2)

Node	ML state	Prob
Angiospermae	three or more (>2)	0.9998
Mesangiospermae	three or more (>2)	1
Magnoliidae	three or more (>2)	0.997
Monocotyledonae	three or more (>2)	0.9994
Eudicotyledonae	three or more (>2)	0.9998
Commelinidae	three or more (>2)	0.8332
Pentapetalae	three or more (>2)	1
Superasteridae	three or more (>2)	0.9994
Asteridae	three or more (>2)	0.9941
Lamiidae	three or more (>2)	0.9999
Campanulidae	three or more (>2)	0.9965
Superrosidae	three or more (>2)	1
Rosidae	three or more (>2)	0.9999
Malvidae	three or more (>2)	0.9994
Fabidae	three or more (>2)	0.9999

Model	LogL	Npar	AIC	AICc	DeltaAICc	w	q01	···
ARD	−388.9	6	789.8	789.9	1.88	0.28	0.0013	···
ARDeq**	−387.96	6	787.92	788.03	0	0.72	0.0013	···
ER	−400.35	1	802.71	802.71	14.69	0	0.0027	···
SYM	−399.78	3	805.57	805.6	17.57	0	0.0025	···
SYMeq	−399.09	3	804.18	804.21	16.19	0	0.0025	···
ORD	−399.51	4	807.02	807.07	19.05	0	0.0022	···
ORDeq	−398.86	4	805.73	805.78	17.75	0	0.0021	···
ORDSYM	−410.55	2	825.09	825.11	37.08	0	0.0053	···
ORDSYMeq	−410.31	2	824.62	824.64	36.61	0	0.0052	···
ORDER	−411.55	1	825.11	825.11	37.09	0	0.0061	···

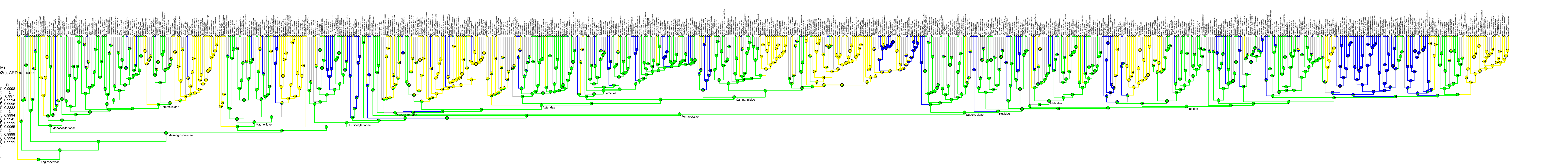

Supplement: Supplementary Data 22 [file ncomms16047-s23.pdf]
