# Supplementary material for: The ancestral flower of angiosperms and its early diversification
Source: Nat Commun. 2017 Aug 1;8:16047. doi: 10.1038/ncomms16047 (PMC5543309; doi:10.1038/ncomms16047)
Supplement: Supplementary Data 23 [file ncomms16047-s24.pdf]

MP ancestral state reconstruction using ancestral.pars
(R:phangorn)
100_A. Functional sex of flowers (D2d), 95 steps

- bisexual
- unisexual

Node	MP state(s)
Angiospermae	bisexual / unisexual
Mesangiospermae	bisexual
Magnoliidae	bisexual
Monocotyledonae	bisexual
Eudicotyledonae	bisexual
Commelinidae	bisexual
Pentapetalae	bisexual
Superasteridae	bisexual
Asteridae	bisexual
Lamiidae	bisexual
Campanulidae	bisexual
Superrosidae	bisexual
Rosidae	bisexual
Malvidae	bisexual
Fabidae	bisexual

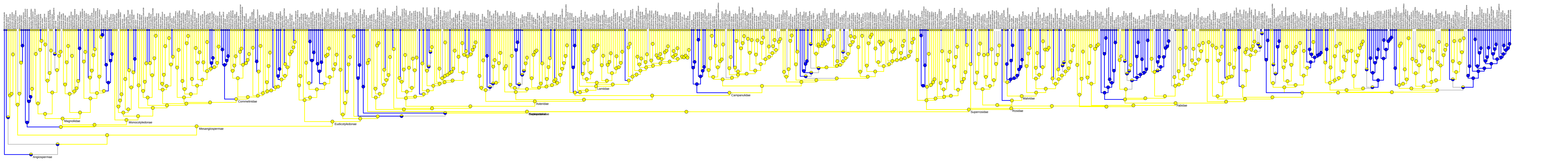

ML ancestral state reconstruction using rayDISC (R:corHMM)
100_A. Functional sex of flowers (D2d), ARDeq model

- bisexual
- unisexual

Node	ML state	Prob
Angiospermae	bisexual	1
Mesangiospermae	bisexual	1
Magnoliidae	bisexual	1
Monocotyledonae	bisexual	1
Eudicotyledonae	bisexual	1
Commelinidae	bisexual	0.9997
Pentapetalae	bisexual	1
Superasteridae	bisexual	1
Asteridae	bisexual	1
Lamiidae	bisexual	1
Campanulidae	bisexual	0.9991
Superrosidae	bisexual	1
Rosidae	bisexual	1
Malvidae	bisexual	0.9993
Fabidae	bisexual	1

Model	LogL	Npar	AIC	AICc	DeltaAICc	w	q01	q10
ARD	−311.75	2	627.5	627.51	1.26	0.24	0.0028	8e−04
ARDeq*	−311.12	2	626.24	626.25	0	0.45	0.0028	7e−04
ER	−314.19	1	630.38	630.38	4.13	0.06	0.0027	0.0027
UNI01	−312.72	1	627.44	627.45	1.19	0.25	0.0028	
UNI10	−358.95	1	719.9	719.9	93.65	0		0.011

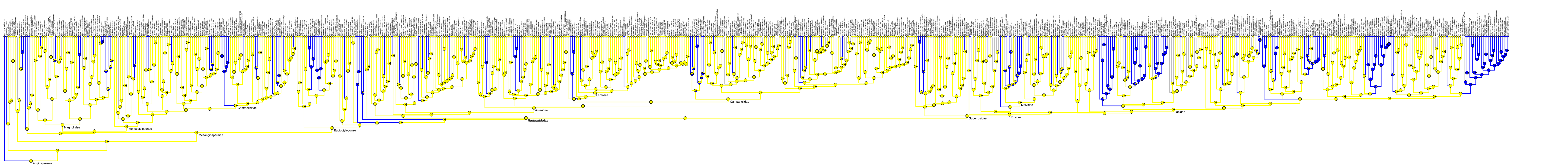

MP ancestral state reconstruction using ancestral.pars
(R:phangorn)
100_B. Structural sex of flowers (D2d), 54 steps

- bisexual
- unisexual

Node	MP state(s)
Angiospermae	bisexual
Mesangiospermae	bisexual
Magnoliidae	bisexual
Monocotyledonae	bisexual
Eudicotyledonae	bisexual
Commelinidae	bisexual
Pentapetalae	bisexual
Superasteridae	bisexual
Asteridae	bisexual
Lamiidae	bisexual
Campanulidae	bisexual
Superrosidae	bisexual
Rosidae	bisexual
Malvidae	bisexual
Fabidae	bisexual

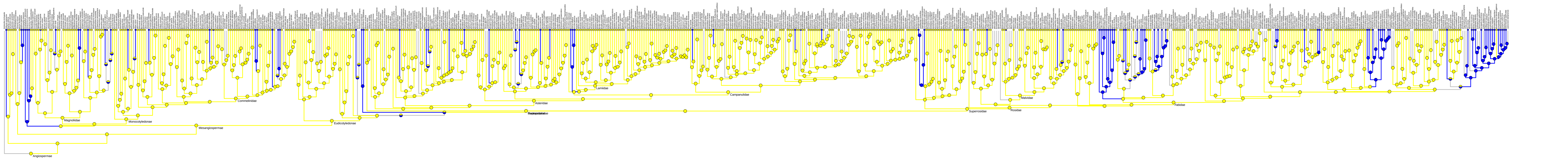

ML ancestral state reconstruction using rayDISC (R:corHMM)
100_B. Structural sex of flowers (D2d), ARDeq model

- bisexual
- unisexual

Node	ML state	Prob
Angiospermae	bisexual	0.9972
Mesangiospermae	bisexual	0.9992
Magnoliidae	bisexual	0.9999
Monocotyledonae	bisexual	0.9998
Eudicotyledonae	bisexual	0.9999
Commelinidae	bisexual	1
Pentapetalae	bisexual	1
Superasteridae	bisexual	1
Asteridae	bisexual	1
Lamiidae	bisexual	0.9999
Campanulidae	bisexual	1
Superrosidae	bisexual	1
Rosidae	bisexual	1
Malvidae	bisexual	0.9999
Fabidae	bisexual	0.9961

Model	LogL	Npar	AIC	AICc	DeltaAICc	w	q01	q10
ARD	−207.51	2	419.02	419.03	0.88	0.31	0.0014	0.0037
ARDeq*	−207.07	2	418.14	418.15	0	0.48	0.0014	0.0035
ER	−208.89	1	419.78	419.78	1.63	0.21	0.0015	0.0015
UNI01	−227.23	1	456.47	456.47	38.32	0	0.0016	
UNI10	−234.16	1	470.31	470.32	52.17	0		0.0152

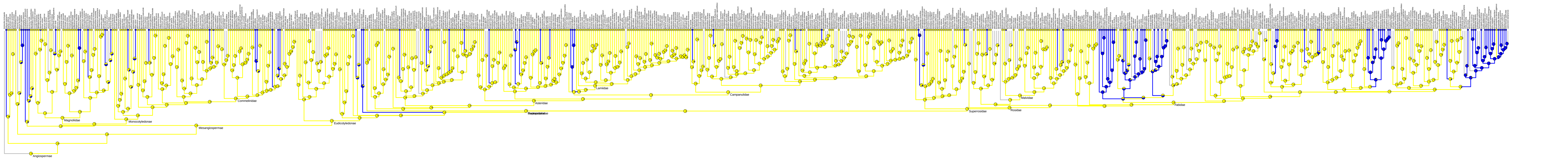

MP ancestral state reconstruction using ancestral.pars
(R:phangorn)
102_B. Ovary position (binary) (D2d), 78 steps

- superior
- inferior

Node	MP state(s)
Angiospermae	superior
Mesangiospermae	superior
Magnoliidae	superior
Monocotyledonae	superior
Eudicotyledonae	superior
Commelinidae	superior
Pentapetalae	superior
Superasteridae	superior
Asteridae	superior
Lamiidae	superior
Campanulidae	superior
Superrosidae	superior
Rosidae	superior
Malvidae	superior
Fabidae	superior

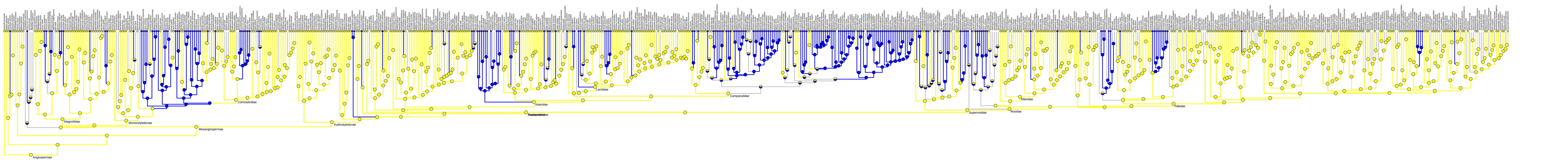

ML ancestral state reconstruction using rayDISC (R:corHMM)
102_B. Ovary position (binary) (D2d), ARDeq model

- superior
- inferior

Node	ML state	Prob
Angiospermae	superior	0.9999
Mesangiospermae	superior	0.9999
Magnoliidae	superior	0.999
Monocotyledonae	superior	0.9948
Eudicotyledonae	superior	0.9999
Commelinidae	superior	0.787
Pentapetalae	superior	0.9998
Superasteridae	superior	0.9998
Asteridae	superior	0.9636
Lamiidae	superior	0.9708
Campanulidae	superior	0.7795
Superrosidae	superior	0.991
Rosidae	superior	0.9918
Malvidae	superior	0.9999
Fabidae	superior	0.9997

Model	LogL	Npar	AIC	AICc	DeltaAICc	w	q01	q10
ARD	−270.5	2	545	545.02	1.21	0.27	0.0019	0.0034
ARDeq*	−269.9	2	543.79	543.81	0	0.49	0.0019	0.0033
ER	−271.63	1	545.26	545.27	1.46	0.24	0.0022	0.0022
UNI01	−279.19	1	560.39	560.39	16.58	0	0.0025	
UNI10	−296.05	1	594.09	594.1	50.29	0		0.0091

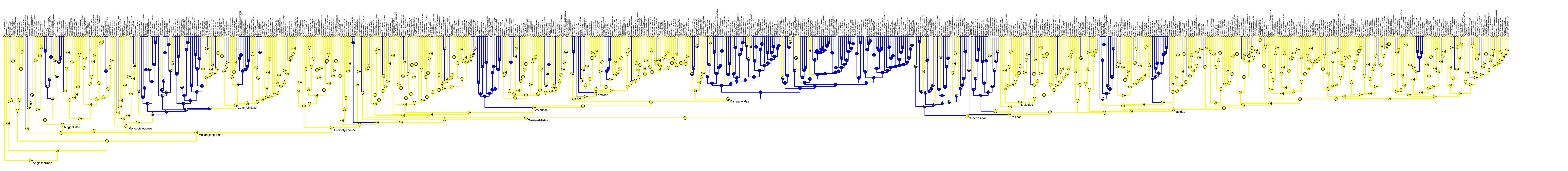

MP ancestral state reconstruction using ancestral.pars
(R:phangorn)
201_A. Perianth presence (D2c), 20 steps

absent
present

Node	MP state(s)
Angiospermae	present
Mesangiospermae	present
Magnoliidae	present
Monocotyledonae	present
Eudicotyledonae	present
Commelinidae	present
Pentapetalae	present
Superasteridae	present
Asteridae	present
Lamiidae	present
Campanulidae	present
Superrosidae	present
Rosidae	present
Malvidae	present
Fabidae	present

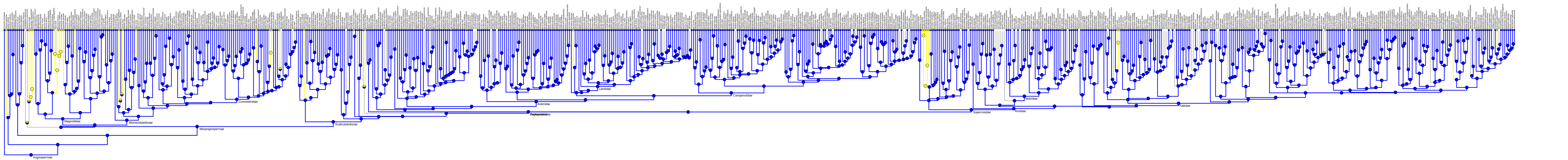

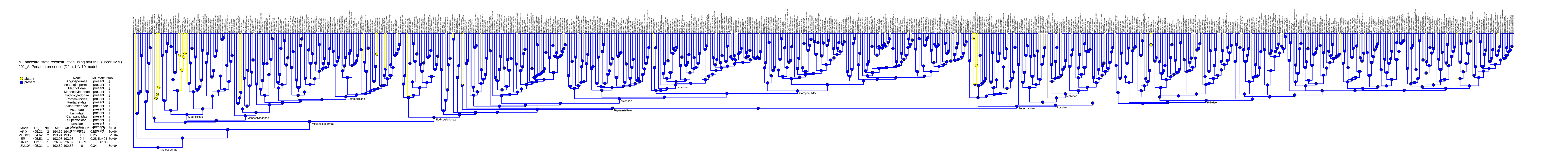
ML ancestral state reconstruction using rayDISC (R:corHMM)
201_A. Perianth presence (D2c), UNI10 model
absent
present
Node ML state Prob
Angiospermae present 1
Mesangiospermae present 1
Magnoliidae present 1
Monocotyledonae present 1
Eudicotyledonae present 1
Commelinidae present 1
Pentapetalae present 1
Superasteridae present 1
Asteridae present 1
Lamiidae present 1
Campanulidae present 1
Superrosidae present 1
Rosidae present 1
Malvidae present 1
Fabidae present 1
Model LogL Npar AIC AICc DeltaAICc w q01 q10
ARD −95.31 2 194.62 194.64 2.01 0.13 0 5e−04
ARDeq −94.62 2 193.24 193.25 0.62 0.25 0 5e−04
ER −95.51 1 193.03 193.03 0.4 0.28 5e−04 5e−04
UNI01 −112.16 1 226.32 226.32 33.69 0 0.0193
UNI10* −95.31 1 192.62 192.63 0 0.34 5e−04
Angiospermae
Magnoliidae
Monocotyledonae
Commelinidae
Mesangiospermae
Eudicotyledonae
Asteridae
Lamiidae
Campanulidae
Superrosidae
Rosidae
Malvidae
Fabidae

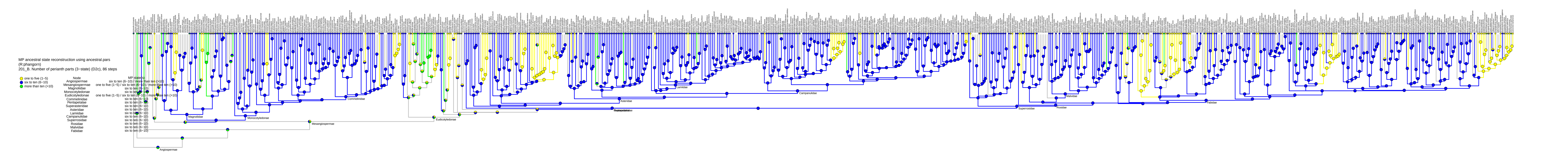
MP ancestral state reconstruction using ancestral.pars
(R:phangorn)
201_B. Number of perianth parts (3−state) (D2c), 86 steps
one to five (1−5)
six to ten (6−10)
more than ten (>10)
Node
MP state(s)
Angiospermae six to ten (6−10) / more than ten (>10)
Mesangiospermae one to five (1−5) / six to ten (6−10) / more than ten (>10)
Magnoliidae six to ten (6−10)
Monocotyledonae six to ten (6−10)
Eudicotyledonae one to five (1−5) / six to ten (6−10) / more than ten (>10)
Commelinidae six to ten (6−10)
Pentapetalae six to ten (6−10)
Superasteridae six to ten (6−10)
Asteridae six to ten (6−10)
Lamiidae six to ten (6−10)
Campanulidae six to ten (6−10)
Superrosidae six to ten (6−10)
Rosidae six to ten (6−10)
Malvidae six to ten (6−10)
Fabidae six to ten (6−10)
Magnoliidae
Monocotyledonae
Commelinidae
Mesangiospermae
Eudicotyledonae
Angiospermae
Asteridae
Lamiidae
Campanulidae
Superrosidae
Rosidae
Malvidae
Fabidae

ML ancestral state reconstruction using rayDISC (R:corHMM)
201_B. Number of perianth parts (3−state) (D2c), ARDeq model

- one to five (1−5)
- six to ten (6−10)
- more than ten (>10)

Node	ML state	Prob
Angiospermae	more than ten (>10)	1
Mesangiospermae	more than ten (>10)	0.9992
Magnoliidae	more than ten (>10)	0.9989
Monocotyledonae	six to ten (6−10)	0.6009
Eudicotyledonae	more than ten (>10)	0.9931
Commelinidae	six to ten (6−10)	0.9999
Pentapetalae	six to ten (6−10)	0.9938
Superasteridae	six to ten (6−10)	0.9938
Asteridae	six to ten (6−10)	0.9999
Lamiidae	six to ten (6−10)	0.9999
Campanulidae	six to ten (6−10)	0.9999
Superrosidae	six to ten (6−10)	0.9989
Rosidae	six to ten (6−10)	0.9992
Malvidae	six to ten (6−10)	0.9998
Fabidae	six to ten (6−10)	0.995

Model	LogL	Npar	AIC	AICc	DeltaAICc	w	q01	
ARD	−326.67	6	665.35	665.45	2.13	0.25	0.004	···
ARDeq**	−325.61	6	663.21	663.32	0	0.73	0.004	···
ER	−351.86	1	705.73	705.73	42.41	0	0.0012	···
SYM	−340.65	3	687.31	687.34	24.02	0	0.002	···
SYMeq	−339.82	3	685.63	685.66	22.34	0	0.0019	···
ORD	−332.94	4	673.88	673.93	10.61	0	0.0041	···
ORDeq	−331.86	4	671.73	671.78	8.46	0.01	0.0041	···
ORDSYM	−344.09	2	692.17	692.19	28.86	0	0.002	···
ORDSYMeq	−343.35	2	690.71	690.72	27.4	0	0.002	···
ORDER	−353.58	1	709.15	709.16	45.84	0	0.0013	···

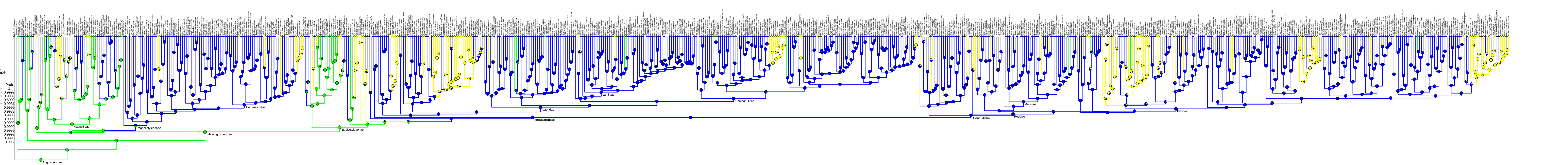

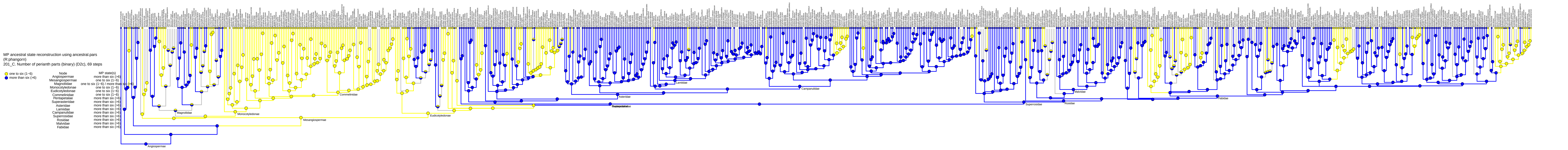
MP ancestral state reconstruction using ancestral.pars
(R:phangorn)
201_C. Number of perianth parts (binary) (D2c), 69 steps
one to six (1−6)
more than six (>6)
Node
MP state(s)
Angiospermae more than six (>6)
Mesangiospermae one to six (1−6)
Magnoliidae one to six (1−6) / more than six (>6)
Monocotyledonae one to six (1−6)
Eudicotyledonae one to six (1−6)
Commelinidae one to six (1−6)
Pentapetalae more than six (>6)
Superasteridae more than six (>6)
Asteridae more than six (>6)
Lamiidae more than six (>6)
Campanulidae more than six (>6)
Superrosidae more than six (>6)
Rosidae more than six (>6)
Malvidae more than six (>6)
Fabidae more than six (>6)

ML ancestral state reconstruction using rayDISC (R:corHMM)
201_C. Number of perianth parts (binary) (D2c), ARDeq model

- one to six (1−6)
- more than six (>6)

Node	ML state	Prob
Angiospermae	more than six (>6)	1
Mesangiospermae	more than six (>6)	0.9997
Magnoliidae	more than six (>6)	0.9996
Monocotyledonae	one to six (1−6)	0.7904
Eudicotyledonae	more than six (>6)	0.9999
Commelinidae	one to six (1−6)	1
Pentapetalae	more than six (>6)	1
Superasteridae	more than six (>6)	1
Asteridae	more than six (>6)	1
Lamiidae	more than six (>6)	1
Campanulidae	more than six (>6)	1
Superrosidae	more than six (>6)	1
Rosidae	more than six (>6)	1
Malvidae	more than six (>6)	1
Fabidae	more than six (>6)	1

Model	LogL	Npar	AIC	AICc	DeltaAICc	w	q01	q10
ARD	−249.53	2	503.05	503.07	1.38	0.33	6e−04	0.0024
ARDeq**	−248.84	2	501.68	501.69	0	0.65	6e−04	0.0024
ER	−255.01	1	512.03	512.03	10.34	0	0.0021	0.0021
UNI01	−326.16	1	654.31	654.32	152.63	0	0.0077	
UNI10	−253.7	1	509.39	509.4	7.7	0.01		0.0027

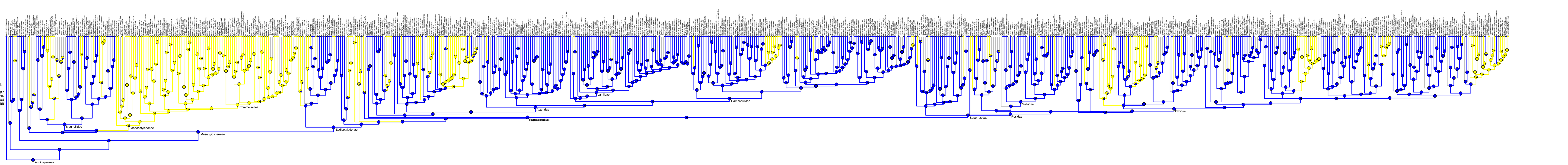

MP ancestral state reconstruction using ancestral.pars
(R:phangorn)
230_A. Perianth phyllotaxy (binary) (D2d), 16 steps

- whorled
- spiral

Node	MP state(s)
Angiospermae	whorled / spiral
Mesangiospermae	whorled
Magnoliidae	whorled
Monocotyledonae	whorled
Eudicotyledonae	whorled
Commelinidae	whorled
Pentapetalae	whorled
Superasteridae	whorled
Asteridae	whorled
Lamiidae	whorled
Campanulidae	whorled
Superrosidae	whorled
Rosidae	whorled
Malvidae	whorled
Fabidae	whorled

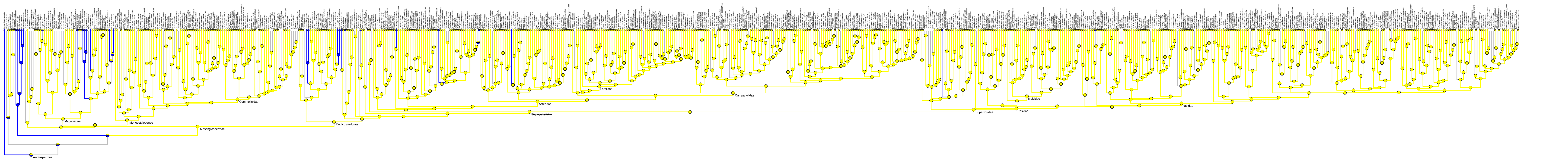

ML ancestral state reconstruction using rayDISC (R:corHMM)
230_A. Perianth phyllotaxy (binary) (D2d), ARDeq model

- whorled
- spiral

Node	ML state	Prob
Angiospermae	spiral	1
Mesangiospermae	spiral	0.9997
Magnoliidae	spiral	0.9947
Monocotyledonae	whorled	0.5446
Eudicotyledonae	spiral	0.9989
Commelinidae	whorled	0.9999
Pentapetalae	whorled	0.9734
Superasteridae	whorled	0.9734
Asteridae	whorled	0.9995
Lamiidae	whorled	1
Campanulidae	whorled	1
Superrosidae	whorled	0.9914
Rosidae	whorled	0.9949
Malvidae	whorled	1
Fabidae	whorled	1

Model	LogL	Npar	AIC	AICc	DeltaAICc	w	q01	q10
ARD	−70.45	2	144.9	144.92	1.38	0.33	2e−04	0.0093
ARDeq**	−69.76	2	143.52	143.53	0	0.66	2e−04	0.0093
ER	−76.8	1	155.6	155.6	12.07	0	4e−04	4e−04
UNI01	−77.3	1	156.6	156.61	13.07	0	4e−04	
UNI10	−78.74	1	159.48	159.48	15.95	0		0.0154

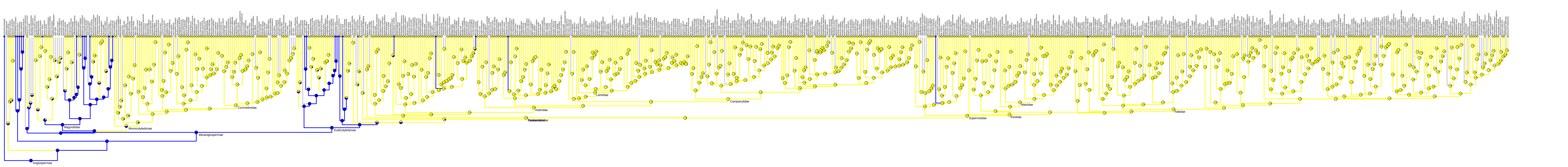

MP ancestral state reconstruction using ancestral.pars
(R:phangorn)
231_A. Number of perianth whorls (D2c), 73 steps

- one (1)
- two (2)
- more than two (>2)

Node	MP state(s)
Angiospermae	more than two (>2)
Mesangiospermae	more than two (>2)
Magnoliidae	more than two (>2)
Monocotyledonae	two (2)
Eudicotyledonae	more than two (>2)
Commelinidae	two (2)
Pentapetalae	two (2)
Superasteridae	two (2)
Asteridae	two (2)
Lamiidae	two (2)
Campanulidae	two (2)
Superrosidae	two (2)
Rosidae	two (2)
Malvidae	two (2)
Fabidae	two (2)

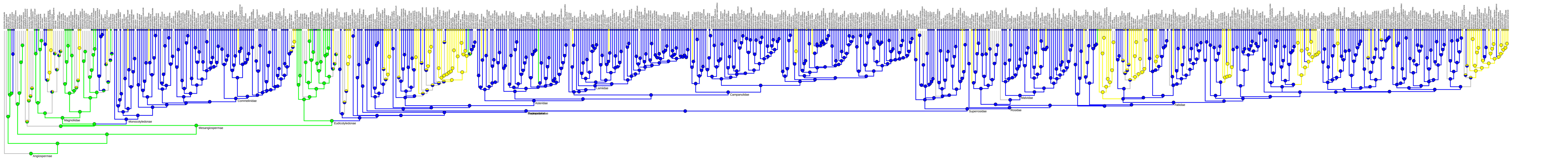

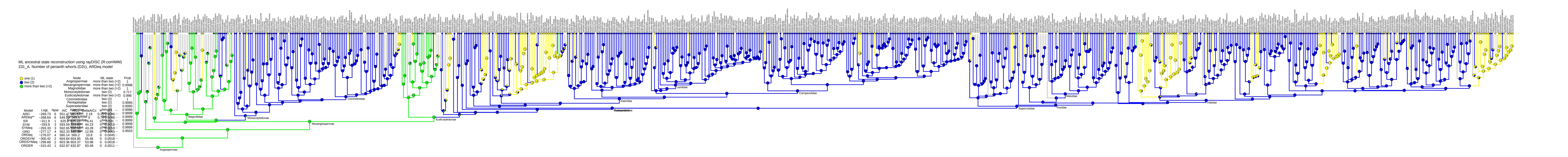

ML ancestral state reconstruction using rayDISC (R:corHMM)
231_A. Number of perianth whorls (D2c), ARDeq model
one (1)
two (2)
more than two (>2)
Node | ML state | Prob
Angiospermae | more than two (>2) | 1
Mesangiospermae | more than two (>2) | 0.9998
Magnoliidae | more than two (>2) | 1
Monocotyledonae | two (2) | 0.717
Eudicotyledonae | more than two (>2) | 0.996
Commelinidae | two (2) | 1
Pentapetalae | two (2) | 0.9995
Superasteridae | two (2) | 0.9995
Asteridae | two (2) | 0.9996
Lamiidae | two (2) | 0.9999
Campanulidae | two (2) | 0.9999
Superrosidae | two (2) | 0.9999
Rosidae | two (2) | 0.9999
Malvidae | two (2) | 0.9999
Fabidae | two (2) | 0.9933
Model | LogL | Npar | AIC | AICc | DeltaAICc | w | q01 | ...
ARD | −269.73 | 6 | 551.47 | 551.57 | 2.18 | 0.25 | 0.0046 | ...
ARDeq** | −268.64 | 6 | 549.29 | 549.4 | 0 | 0.74 | 0.0046 | ...
ER | −311.9 | 1 | 625.8 | 625.81 | 76.41 | 0 | 0.001 | ...
SYM | −293.8 | 3 | 593.59 | 593.62 | 44.23 | 0 | 0.0018 | ...
SYMeq | −293.33 | 3 | 592.65 | 592.68 | 43.28 | 0 | 0.0018 | ...
ORD | −277.17 | 4 | 562.33 | 562.38 | 12.99 | 0 | 0.0045 | ...
ORDeq | −276.07 | 4 | 560.14 | 560.2 | 10.8 | 0 | 0.0045 | ...
ORDSYM | −300.42 | 2 | 604.84 | 604.85 | 55.46 | 0 | 0.0018 | ...
ORDSYMeq | −299.68 | 2 | 603.36 | 603.37 | 53.98 | 0 | 0.0018 | ...
ORDER | −315.43 | 1 | 632.87 | 632.87 | 83.48 | 0 | 0.0011 | ...
Magnoliidae
Monocotyledonae
Mesangiospermae
Eudicotyledonae
Angiospermae
Commelinidae
Asteridae
Lamiidae
Campanulidae
Superrosidae
Rosidae
Malvidae
Fabidae

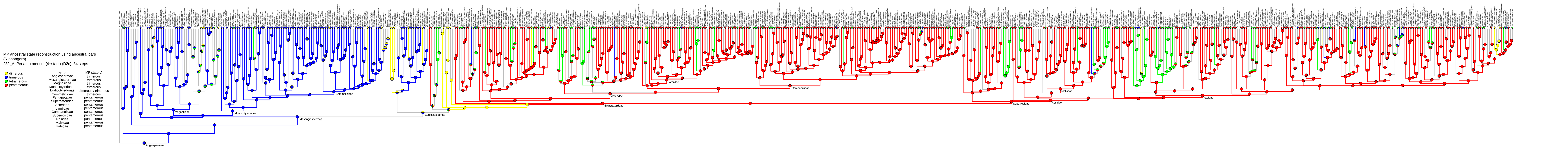
MP ancestral state reconstruction using ancestral.pars
(R:phangorn)
232_A. Perianth merism (4−state) (D2c), 84 steps
dimerous
trimerous
tetramerous
pentamerous
Node
MP state(s)
Angiospermae trimerous
Mesangiospermae trimerous
Magnoliidae trimerous
Monocotyledonae trimerous
Eudicotyledonae dimerous / trimerous
Commelinidae trimerous
Pentapetalae pentamerous
Superasteridae pentamerous
Asteridae pentamerous
Lamiidae pentamerous
Campanulidae pentamerous
Superrosidae pentamerous
Rosidae pentamerous
Malvidae pentamerous
Fabidae pentamerous
Angiospermae
Mesangiospermae
Magnoliidae
Monocotyledonae
Commelinidae
Eudicotyledonae
Asteridae
Lamiidae
Campanulidae
Superrosidae
Rosidae
Malvidae
Fabidae

ML ancestral state reconstruction using rayDISC (R:corHMM)
232_A. Perianth merism (4−state) (D2c), SYMeq model

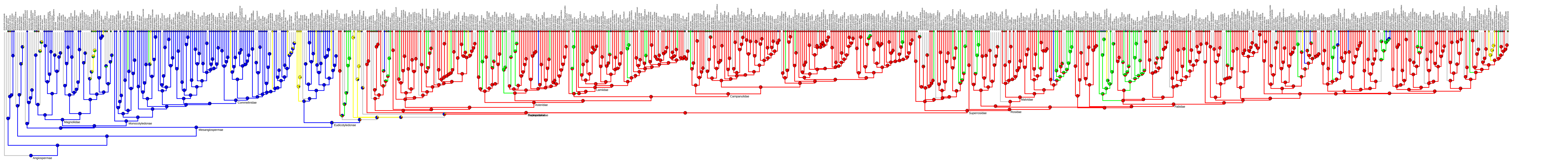

- dimerous
- trimerous
- tetramerous
- pentamerous

Node	ML state	Prob
Angiospermae	trimerous	1
Mesangiospermae	trimerous	0.9996
Magnoliidae	trimerous	1
Monocotyledonae	trimerous	1
Eudicotyledonae	trimerous	0.9073
Commelinidae	trimerous	1
Pentapetalae	pentamerous	0.9997
Superasteridae	pentamerous	0.9997
Asteridae	pentamerous	0.9999
Lamiidae	pentamerous	1
Campanulidae	pentamerous	1
Superrosidae	pentamerous	1
Rosidae	pentamerous	1
Malvidae	pentamerous	0.9999
Fabidae	pentamerous	0.9999

Model	LogL	Npar	AIC	AICc	DeltaAICc	w	q01	···
ARD	−320.86	12	665.72	666.12	6.74	0.02	3e−04	···
ARDeq	−319.73	12	663.46	663.86	4.49	0.07	2e−04	···
ER	−359.5	1	721	721	61.63	0	8e−04	···
SYM	−324.91	6	661.81	661.92	2.55	0.2	0.0012	···
SYMeq**	−323.63	6	659.27	659.37	0	0.7	0.0012	···
ORD	−335.27	6	682.53	682.64	23.26	0	0	···
ORDeq	−334.85	6	681.71	681.81	22.44	0	0	···
ORDSYM	−343.28	3	692.56	692.59	33.21	0	0.0019	···
ORDSYMeq	−342.13	3	690.25	690.28	30.91	0	0.0019	···
ORDER	−344.83	1	691.66	691.66	32.29	0	0.0023	···

MP ancestral state reconstruction using ancestral.pars
(R:phangorn)
232_B. Perianth merism (3−state) (D2c), 70 steps

- trimerous
- tetramerous
- pentamerous

Node	MP state(s)
Angiospermae	trimerous
Mesangiospermae	trimerous
Magnoliidae	trimerous
Monocotyledonae	trimerous
Eudicotyledonae	trimerous
Commelinidae	trimerous
Pentapetalae	pentamerous
Superasteridae	pentamerous
Asteridae	pentamerous
Lamiidae	pentamerous
Campanulidae	pentamerous
Superrosidae	pentamerous
Rosidae	pentamerous
Malvidae	pentamerous
Fabidae	pentamerous

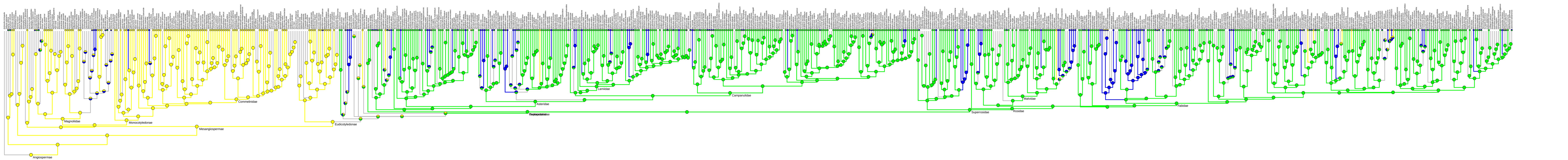

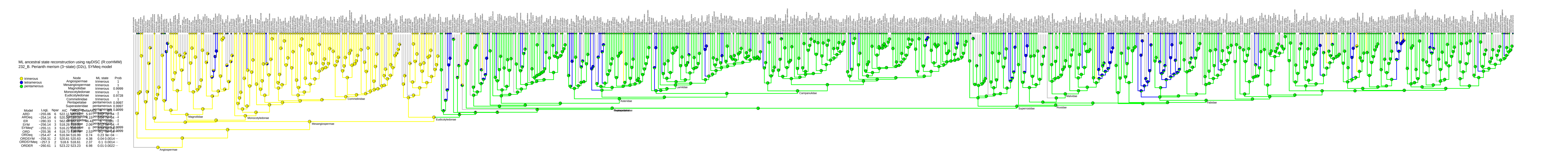
ML ancestral state reconstruction using rayDISC (R:corHMM)
232_B. Perianth merism (3−state) (D2c), SYMeq model
trimerous
tetramerous
pentamerous
Node | ML state | Prob
Angiospermae | trimerous | 1
Mesangiospermae | trimerous | 1
Magnoliidae | trimerous | 0.9999
Monocotyledonae | trimerous | 1
Eudicotyledonae | trimerous | 0.9728
Commelinidae | trimerous | 1
Pentapetalae | pentamerous | 0.9997
Superasteridae | pentamerous | 0.9997
Asteridae | pentamerous | 0.9999
Lamiidae | pentamerous | 1
Campanulidae | pentamerous | 1
Superrosidae | pentamerous | 1
Rosidae | pentamerous | 1
Malvidae | pentamerous | 0.9999
Fabidae | pentamerous | 0.9999
Model | LogL | Npar | AIC | AICc | DeltaAICc | w | q01
ARD | −255.06 | 6 | 522.11 | 5.97 | 7e−04
ARDeq | −254.14 | 6 | 520.29 | 4.15 | 0.04 | 7e−04
ER | −280.33 | 1 | 562.66 | 46.42 | 0.001
SYM | −256.14 | 3 | 518.28 | 2.06 | 0.12 | 9e−04
SYMeq* | −255.11 | 3 | 516.22 | 0 | 0.34 | 9e−04
ORD | −255.36 | 4 | 518.73 | 2.53 | 0.1 | 9e−04
ORDeq | −254.47 | 4 | 516.94 | 516.99 | 0.74 | 0.23 | 9e−04
ORDSYM | −258.31 | 2 | 520.61 | 520.63 | 4.38 | 0.04 | 0.0014
ORDSYMeq | −257.3 | 2 | 518.6 | 518.61 | 2.37 | 0.1 | 0.0014
ORDER | −260.61 | 1 | 523.22 | 523.23 | 6.98 | 0.01 | 0.0022
Magnoliidae
Monocotyledonae
Commelinidae
Mesangiospermae
Angiospermae
Eudicotyledonae
Asteridae
Lamiidae
Campanulidae
Superrosidae
Rosidae
Malvidae
Fabidae

MP ancestral state reconstruction using ancestral.pars
(R:phangorn)
234_A. Perianth differentiation (binary) (D2d), 65 steps

- undifferentiated
- differentiated

Node	MP state(s)
Angiospermae	undifferentiated
Mesangiospermae	undifferentiated
Magnoliidae	undifferentiated / differentiated
Monocotyledonae	undifferentiated
Eudicotyledonae	undifferentiated
Commelinidae	undifferentiated
Pentapetalae	differentiated
Superasteridae	differentiated
Asteridae	differentiated
Lamiidae	differentiated
Campanulidae	differentiated
Superrosidae	differentiated
Rosidae	differentiated
Malvidae	differentiated
Fabidae	differentiated

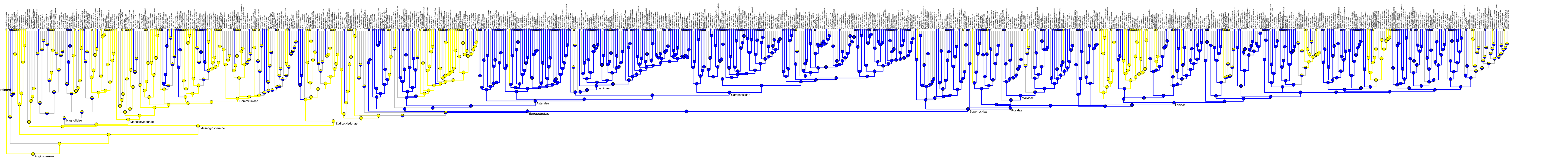

ML ancestral state reconstruction using rayDISC (R:corHMM)
234_A. Perianth differentiation (binary) (D2d), ARDeq model

- undifferentiated
- differentiated

Node	ML state	Prob
Angiospermae	undifferentiated	1
Mesangiospermae	undifferentiated	0.9998
Magnoliidae	undifferentiated	0.9995
Monocotyledonae	undifferentiated	0.9999
Eudicotyledonae	undifferentiated	0.9851
Commelinidae	undifferentiated	0.9998
Pentapetalae	differentiated	0.8866
Superasteridae	differentiated	0.8866
Asteridae	differentiated	0.996
Lamiidae	differentiated	0.9998
Campanulidae	differentiated	1
Superrosidae	differentiated	0.9819
Rosidae	differentiated	0.9889
Malvidae	differentiated	0.9981
Fabidae	differentiated	0.9655

Model	LogL	Npar	AIC	AICc	DeltaAICc	w	q01	q10
ARD	−200.06	2	404.12	404.13	1.32	0.34	0.0043	0.0014
ARDeq**	−199.4	2	402.8	402.82	0	0.66	0.0043	0.0014
ER	−209.3	1	420.59	420.6	17.78	0	0.0024	0.0024
UNI01	−228.15	1	458.29	458.3	55.48	0	0.0081	
UNI10	−234.54	1	471.09	471.09	68.28	0		0.0032

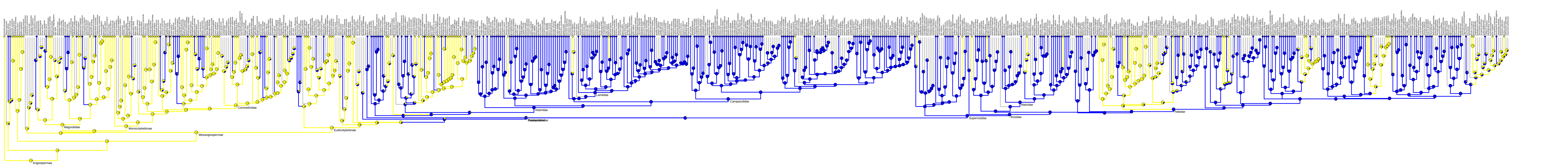

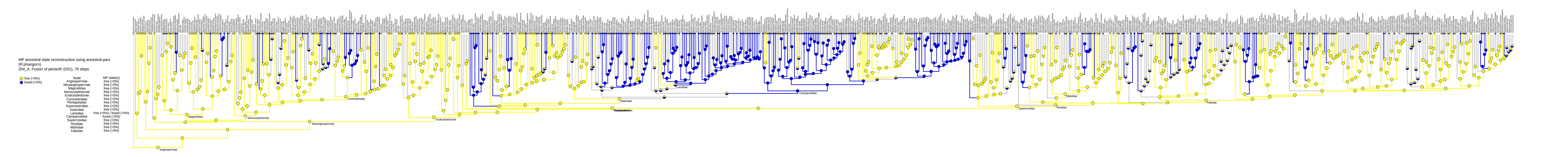
MP ancestral state reconstruction using ancestral.pars
(R:phangorn)
204_A. Fusion of perianth (D2c), 76 steps
free (<5%)
fused (>5%)
Node | MP state(s)
Angiospermae | free (<5%)
Mesangiospermae | free (<5%)
Magnoliidae | free (<5%)
Monocotyledonae | free (<5%)
Eudicotyledonae | free (<5%)
Commelinidae | free (<5%)
Pentapetalae | free (<5%)
Superasteridae | free (<5%)
Asteridae | free (<5%)
Lamiidae | free (<5%) / fused (>5%)
Campanulidae | fused (>5%)
Superrosidae | free (<5%)
Rosidae | free (<5%)
Malvidae | free (<5%)
Fabidae | free (<5%)
Magnoliidae
Monocotyledonae
Commelinidae
Mesangiospermae
Eudicotyledonae
Angiospermae
Asteridae
Lamiidae
Campanulidae
Superrosidae
Rosidae
Malvidae
Fabidae

ML ancestral state reconstruction using rayDISC (R:corHMM)
204_A. Fusion of perianth (D2c), ER model

- free (<5%)
- fused (>5%)

Node	ML state	Prob
Angiospermae	free (<5%)	0.9331
Mesangiospermae	free (<5%)	0.9999
Magnoliidae	free (<5%)	0.9987
Monocotyledonae	free (<5%)	0.9994
Eudicotyledonae	free (<5%)	0.9998
Commelinidae	free (<5%)	0.9975
Pentapetalae	free (<5%)	0.993
Superasteridae	free (<5%)	0.993
Asteridae	free (<5%)	0.6804
Lamiidae	fused (>5%)	0.9782
Campanulidae	fused (>5%)	0.9771
Superrosidae	free (<5%)	0.9944
Rosidae	free (<5%)	0.995
Malvidae	free (<5%)	0.9971
Fabidae	free (<5%)	0.9993

Model	LogL	Npar	AIC	AICc	DeltaAICc	w	q01	q10
ARD	−225.75	2	455.51	455.52	1.65	0.2	0.0041	0.0035
ARDeq	−225.17	2	454.34	454.35	0.48	0.35	0.0041	0.0035
ER*	−225.93	1	453.87	453.87	0	0.45	0.004	0.004
UNI01	−239.31	1	480.61	480.62	26.74	0	0.0045	
UNI10	−237.05	1	476.1	476.11	22.23	0		0.0076

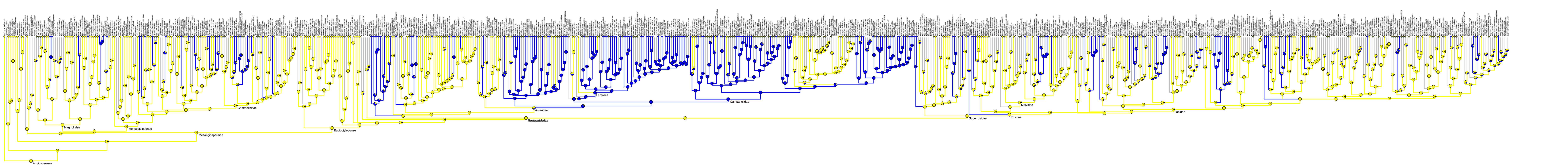

MP ancestral state reconstruction using ancestral.pars
(R:phangorn)
207_A. Symmetry of perianth (binary) (D2d), 55 steps

- actinomorphic
- zygomorphic

Node	MP state(s)
Angiospermae	actinomorphic
Mesangiospermae	actinomorphic
Magnoliidae	actinomorphic
Monocotyledonae	actinomorphic
Eudicotyledonae	actinomorphic
Commelinidae	actinomorphic
Pentapetalae	actinomorphic
Superasteridae	actinomorphic
Asteridae	actinomorphic
Lamiidae	actinomorphic
Campanulidae	actinomorphic
Superrosidae	actinomorphic
Rosidae	actinomorphic
Malvidae	actinomorphic
Fabidae	actinomorphic

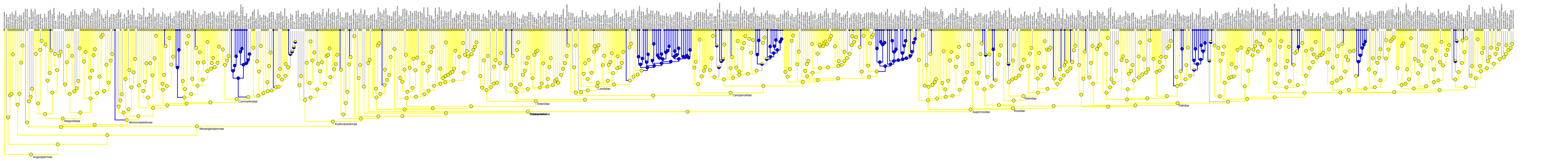

ML ancestral state reconstruction using rayDISC (R:corHMM)
207_A. Symmetry of perianth (binary) (D2d), ARDeq model

- actinomorphic
- zygomorphic

Node	ML state	Prob
Angiospermae	actinomorphic	0.9991
Mesangiospermae	actinomorphic	1
Magnoliidae	actinomorphic	0.9998
Monocotyledonae	actinomorphic	0.9981
Eudicotyledonae	actinomorphic	0.9998
Commelinidae	actinomorphic	0.9979
Pentapetalae	actinomorphic	1
Superasteridae	actinomorphic	1
Asteridae	actinomorphic	1
Lamiidae	actinomorphic	0.9982
Campanulidae	actinomorphic	0.9999
Superrosidae	actinomorphic	1
Rosidae	actinomorphic	1
Malvidae	actinomorphic	0.9997
Fabidae	actinomorphic	0.9999

Model	LogL	Npar	AIC	AICc	DeltaAICc	w	q01	q10
ARD	−209.93	2	423.87	423.88	1.03	0.37	0.0013	0.0054
ARDeq**	−209.42	2	422.84	422.86	0	0.63	0.0013	0.0053
ER	−217.33	1	436.66	436.66	13.81	0	0.0015	0.0015
UNI01	−261.66	1	525.32	525.33	102.47	0	0.0018	
UNI10	−219.78	1	441.57	441.57	18.72	0		0.0152

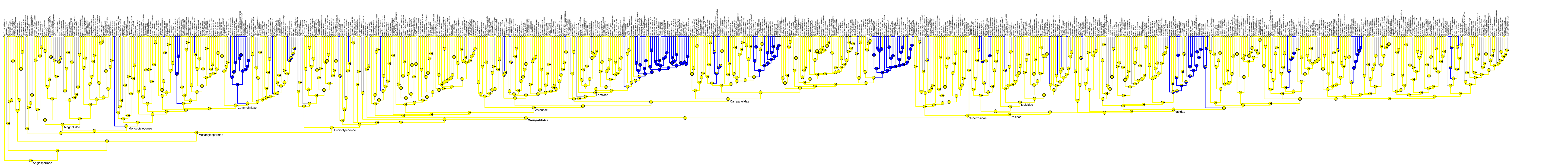

MP ancestral state reconstruction using ancestral.pars
(R:phangorn)
301_B. Number of fertile stamens (3−state) (D2c), 156 steps

- one to five (1−5)
- six to ten (6−10)
- more than ten (>10)

Node	MP state(s)
Angiospermae	more than ten (>10)
Mesangiospermae	one to five (1−5) / six to ten (6−10)
Magnoliidae	six to ten (6−10)
Monocotyledonae	six to ten (6−10)
Eudicotyledonae	one to five (1−5) / six to ten (6−10)
Commelinidae	six to ten (6−10)
Pentapetalae	one to five (1−5) / six to ten (6−10) / more than ten (>10)
Superasteridae	one to five (1−5) / six to ten (6−10) / more than ten (>10)
Asteridae	one to five (1−5) / six to ten (6−10)
Lamiidae	one to five (1−5)
Campanulidae	one to five (1−5)
Superrosidae	six to ten (6−10)
Rosidae	six to ten (6−10)
Malvidae	six to ten (6−10)
Fabidae	six to ten (6−10)

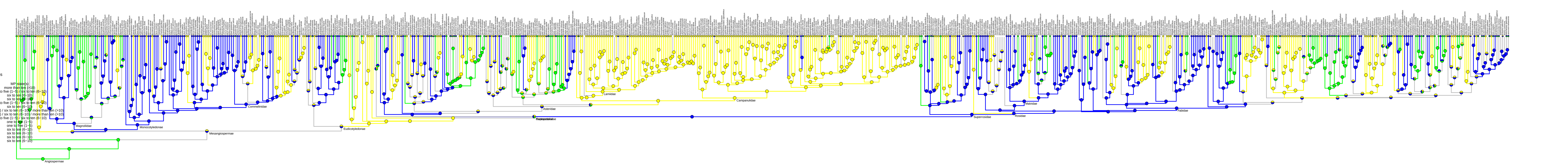

ML ancestral state reconstruction using rayDISC (R:corHMM)
301_B. Number of fertile stamens (3−state) (D2c), ARDeq model

- one to five (1−5)
- six to ten (6−10)
- more than ten (>10)

Node	ML state	Prob
Angiospermae	more than ten (>10)	1
Mesangiospermae	more than ten (>10)	0.9998
Magnoliidae	more than ten (>10)	0.999
Monocotyledonae	six to ten (6−10)	0.5435
Eudicotyledonae	more than ten (>10)	0.9998
Commelinidae	six to ten (6−10)	0.9997
Pentapetalae	more than ten (>10)	1
Superasteridae	more than ten (>10)	1
Asteridae	more than ten (>10)	0.9975
Lamiidae	one to five (1−5)...	0.9996
Campanulidae	one to five (1−5)...	0.9967
Superrosidae	more than ten (>10)	0.9999
Rosidae	more than ten (>10)	0.9996
Malvidae	more than ten (>10)	0.8199
Fabidae	more than ten (>10)	0.9998

Model	LogL	Npar	AIC	AICc	DeltaAICc	w	q01	
ARD	−493.78	6	999.56	999.66	2.15	0.25	3e−04	···
ARDeq**	−492.7	6	997.4	997.51	0	0.75	3e−04	···
ER	−534	1	1070.01	1070.01	72.51	0	0.0025	···
SYM	−527.69	3	1061.38	1061.41	63.9	0	0.0036	···
SYMeq	−527.35	3	1060.71	1060.74	63.23	0	0.0036	···
ORD	−507.3	4	1022.61	1022.66	25.15	0	7e−04	···
ORDeq	−506.79	4	1021.57	1021.62	24.11	0	7e−04	···
ORDSYM	−530.84	2	1065.69	1065.7	68.19	0	0.004	···
ORDSYMeq	−530.47	2	1064.94	1064.96	67.45	0	0.004	···
ORDER	−530.88	1	1063.77	1063.77	66.26	0	0.0041	···

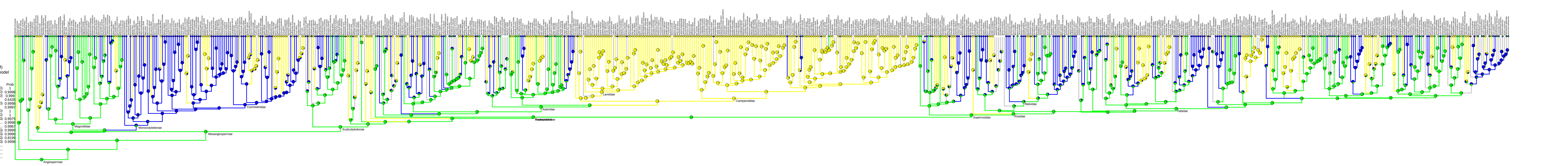

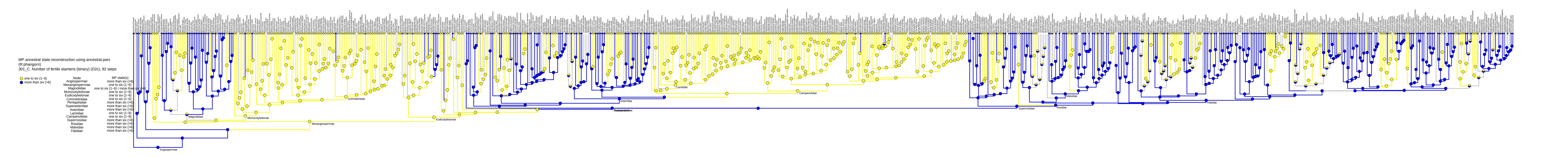
MP ancestral state reconstruction using ancestral.pars
(R:phangorn)
301_C. Number of fertile stamens (binary) (D2c), 92 steps
one to six (1−6)
more than six (>6)
Node
Angiospermae
Mesangiospermae
Magnoliidae
Monocotyledonae
Eudicotyledonae
Commelinidae
Pentapetalae
Superasteridae
Asteridae
Lamiidae
Campanulidae
Superrosidae
Rosidae
Malvidae
Fabidae
MP state(s)
more than six (>6)
one to six (1−6)
one to six (1−6) / more than six (>6)
one to six (1−6)
one to six (1−6)
one to six (1−6)
more than six (>6)
more than six (>6)
more than six (>6)
one to six (1−6)
one to six (1−6)
more than six (>6)
more than six (>6)
more than six (>6)
more than six (>6)

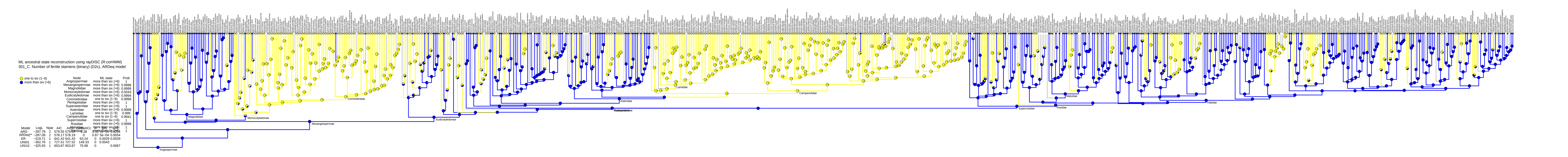
ML ancestral state reconstruction using rayDISC (R:corHMM)
301_C. Number of fertile stamens (binary) (D2c), ARDeq model
one to six (1−6)
more than six (>6)
Node | ML state | Prob
Angiospermae | more than six (>6) | 1
Mesangiospermae | more than six (>6) | 0.9996
Magnoliidae | more than six (>6) | 0.9999
Monocotyledonae | more than six (>6) | 0.5043
Eudicotyledonae | more than six (>6) | 0.9994
Commelinidae | one to six (1−6) | 0.9999
Pentapetalae | more than six (>6) | 1
Superasteridae | more than six (>6) | 1
Asteridae | more than six (>6) | 0.9999
Lamiidae | one to six (1−6) | 0.999
Campanulidae | one to six (1−6) | 0.9941
Superrosidae | more than six (>6) | 1
Rosidae | more than six (>6) | 0.9999
Malvidae | more than six (>6) | 1
Fabidae | more than six (>6) | 1
Model | LogL | Npar | AIC | AICc | DeltaAICc | w | q01 | q10
ARD | −287.78 | 2 | 579.55 | 579.57 | 1.38 | 0.33 | 5e−04 | 0.0054
ARDeq** | −287.09 | 2 | 578.17 | 578.19 | 0 | 0.67 | 5e−04 | 0.0054
ER | −319.71 | 1 | 641.42 | 641.43 | 63.24 | 0 | 0.0029 | 0.0029
UNI01 | −362.76 | 1 | 727.51 | 727.52 | 149.33 | 0 | 0.0043 |
UNI10 | −325.93 | 1 | 653.87 | 653.87 | 75.68 | 0 | | 0.0067
Angiospermae
Mesangiospermae
Magnoliidae
Monocotyledonae
Commelinidae
Eudicotyledonae
Asteridae
Lamiidae
Campanulidae
Superrosidae
Rosidae
Malvidae
Fabidae

MP ancestral state reconstruction using ancestral.pars
(R:phangorn)
330_A. Androecium structural phyllotaxy (binary) (D2d), 12 steps

- whorled
- spiral

Node	MP state(s)
Angiospermae	whorled / spiral
Mesangiospermae	whorled
Magnoliidae	whorled
Monocotyledonae	whorled
Eudicotyledonae	whorled
Commelinidae	whorled
Pentapetalae	whorled
Superasteridae	whorled
Asteridae	whorled
Lamiidae	whorled
Campanulidae	whorled
Superrosidae	whorled
Rosidae	whorled
Malvidae	whorled
Fabidae	whorled

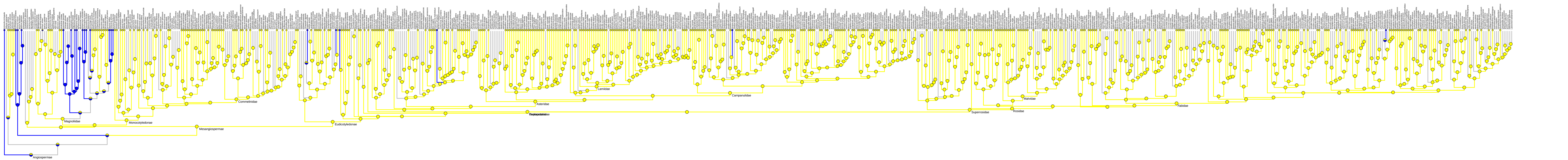

ML ancestral state reconstruction using rayDISC (R:corHMM)
330_A. Androecium structural phyllotaxy (binary) (D2d), ARD model

- whorled
- spiral

Node	ML state	Prob
Angiospermae	spiral	0.9993
Mesangiospermae	spiral	0.9902
Magnoliidae	spiral	0.9885
Monocotyledonae	whorled	0.6055
Eudicotyledonae	spiral	0.9503
Commelinidae	whorled	0.9999
Pentapetalae	whorled	0.9974
Superasteridae	whorled	0.9974
Asteridae	whorled	1
Lamiidae	whorled	1
Campanulidae	whorled	1
Superrosidae	whorled	0.9996
Rosidae	whorled	0.9998
Malvidae	whorled	1
Fabidae	whorled	1

Model	LogL	Npar	AIC	AICc	DeltaAICc	w	q01	q10
ARD**	−53.97	2	111.93	111.95	0	0.9	1e−04	0.0071
ARDeq	−57.49	2	118.99	119	7.05	0.03	4e−04	0
ER	−58.24	1	118.49	118.49	6.54	0.03	4e−04	4e−04
UNI01	−58.19	1	118.37	118.38	6.43	0.04	4e−04	
UNI10	−77.73	1	157.45	157.46	45.51	0		0.0175

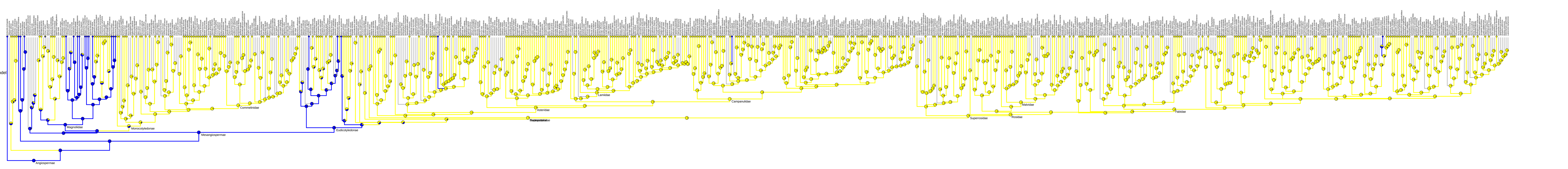

MP ancestral state reconstruction using ancestral.pars
(R:phangorn)
331_A. Number of androecium structural whorls (3−state) (D2c), 75 steps

- one (1)
- two (2)
- more than two (>2)

Node	MP state(s)
Angiospermae	more than two (>2)
Mesangiospermae	more than two (>2)
Magnoliidae	more than two (>2)
Monocotyledonae	two (2)
Eudicotyledonae	more than two (>2)
Commelinidae	two (2)
Pentapetalae	one (1)
Superasteridae	one (1)
Asteridae	one (1)
Lamiidae	one (1)
Campanulidae	one (1)
Superrosidae	one (1)
Rosidae	one (1)
Malvidae	one (1) / two (2)
Fabidae	one (1)

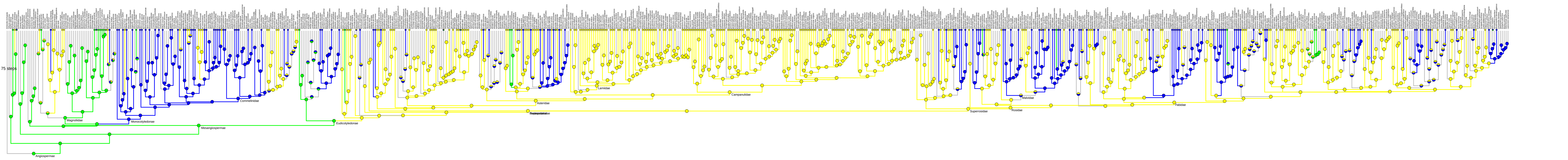

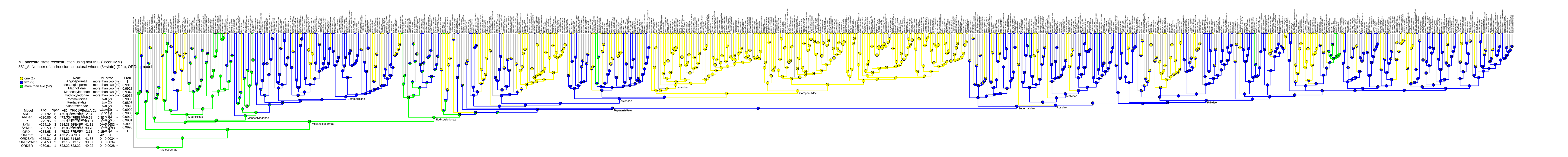

ML ancestral state reconstruction using rayDISC (R:corHMM)
331_A. Number of androecium structural whorls (3−state) (D2c), ORDeq model
one (1)
two (2)
more than two (>2)
Node ML state Prob
Angiospermae more than two (>2) 1
Mesangiospermae more than two (>2) 0.9916
Magnoliidae more than two (>2) 0.9928
Monocotyledonae more than two (>2) 0.9342
Eudicotyledonae more than two (>2) 0.9035
Commelinidae two (2) 0.9803
Pentapetalae two (2) 0.9893
Superasteridae two (2) 0.9893
Asteridae two (2) 0.9999
Lamiidae one (1) 0.9985
Campanulidae one (1) 0.9912
Superrosidae two (2) 0.9981
Rosidae two (2) 0.999
Malvidae two (2) 0.9996
Fabidae two (2) 1
Model LogL Npar AIC AICc DeltaAICc w d01 ...
ARD −231.92 6 475.83 475.94 2.64 0.11 0 ...
ARDeq −230.86 6 473.71 473.82 0.52 0.32 0 ...
ER −279.95 1 561.91 561.91 88.61 0 0.0017 ...
SYM −254.19 3 514.38 514.41 41.11 0 0.0033 ...
SYMeq −253.53 3 513.05 513.08 39.78 0 0.0033 ...
ORD −233.68 4 475.36 475.41 2.11 0.15 0 ...
ORDeq* −232.62 4 473.25 473.3 0 0.42 0 ...
ORDSYM −255.31 2 514.61 514.63 41.33 0 0.0034 ...
ORDSYMeq −254.58 2 513.16 513.17 39.87 0 0.0034 ...
ORDER −260.61 1 523.22 523.22 49.92 0 0.0028 ...
Angiospermae
Mesangiospermae
Magnoliidae
Monocotyledonae
Commelinidae
Eudicotyledonae
Asteridae
Lamiidae
Campanulidae
Superrosidae
Rosidae
Malvidae
Fabidae

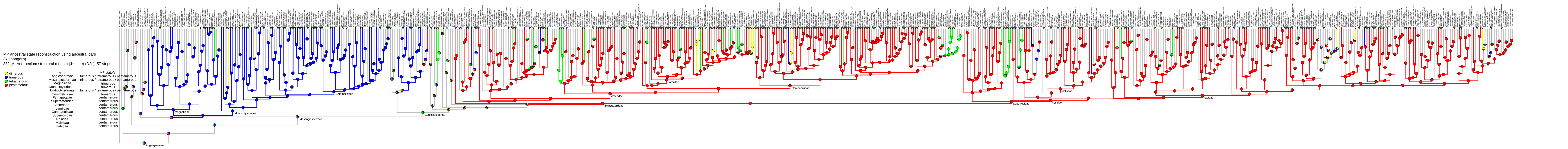
MP ancestral state reconstruction using ancestral.pars
(R:phangorn)
332_A. Androecium structural merism (4−state) (D2c), 57 steps
dimerous
trimerous
tetramerous
pentamerous
Node
MP state(s)
Angiospermae trimerous / tetramerous / pentamerous
Mesangiospermae trimerous / tetramerous / pentamerous
Magnoliidae trimerous
Monocotyledonae trimerous
Eudicotyledonae trimerous / tetramerous / pentamerous
Commelinidae trimerous
Pentapetalae pentamerous
Superasteridae pentamerous
Asteridae pentamerous
Lamiidae pentamerous
Campanulidae pentamerous
Superrosidae pentamerous
Rosidae pentamerous
Malvidae pentamerous
Fabidae pentamerous
Angiospermae
Magnoliidae
Monocotyledonae
Mesangiospermae
Commelinidae
Eudicotyledonae
Asteridae
Lamiidae
Campanulidae
Superrosidae
Rosidae
Malvidae
Fabidae

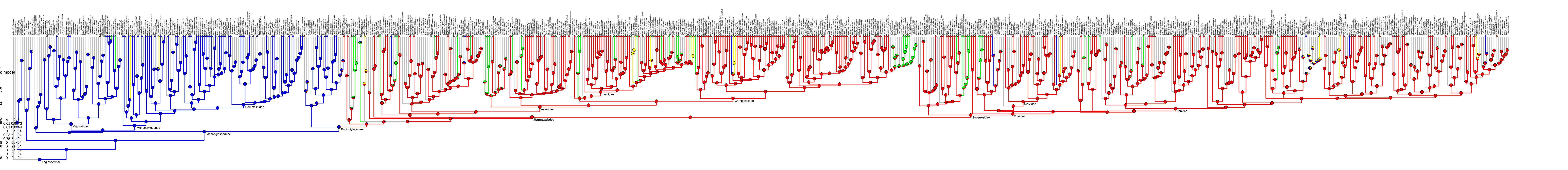

ML ancestral state reconstruction using rayDISC (R:corHMM)
332_A. Androecium structural merism (4−state) (D2c), SYMeq model

- dimerous
- trimerous
- tetramerous
- pentamerous

Node	ML state	Prob
Angiospermae	trimerous	0.9996
Mesangiospermae	trimerous	0.9947
Magnoliidae	trimerous	1
Monocotyledonae	trimerous	1
Eudicotyledonae	trimerous	0.8742
Commelinidae	trimerous	1
Pentapetalae	pentamerous	1
Superasteridae	pentamerous	1
Asteridae	pentamerous	0.9993
Lamiidae	pentamerous	0.9999
Campanulidae	pentamerous	1
Superrosidae	pentamerous	1
Rosidae	pentamerous	1
Malvidae	pentamerous	1
Fabidae	pentamerous	1

Model	LogL	Npar	AIC	AICc	DeltaAICc	w	q01	···
ARD	−222.17	12	468.35	468.75	9.82	0.01	0.0073	···
ARDeq	−221.32	12	466.64	467.04	8.11	0.01	0.0064	···
ER	−237.54	1	477.08	477.09	18.16	0	8e−04	···
SYM	−224.59	6	461.18	461.29	2.36	0.23	5e−04	···
SYMeq**	−223.41	6	458.82	458.93	0	0.75	5e−04	···
ORD	−1e+06	6	2000012	2000012.11	1999553.18	0	9e−04	···
ORDeq	−1e+06	6	2000012	2000012.11	1999553.18	0	9e−04	···
ORDSYM	−1e+06	3	2000006	2000006.03	1999547.1	0	9e−04	···
ORDSYMeq	−1e+06	3	2000006	2000006.03	1999547.1	0	9e−04	···
ORDER	−1e+06	1	2000002	2000002.01	1999543.08	0	9e−04	···

MP ancestral state reconstruction using ancestral.pars
(R:phangorn)
332_B. Androecium structural merism (3−state) (D2c), 48 steps

- trimerous
- tetramerous
- pentamerous

Node	MP state(s)
Angiospermae	trimerous / tetramerous / pentamerous
Mesangiospermae	trimerous / tetramerous / pentamerous
Magnoliidae	trimerous
Monocotyledonae	trimerous
Eudicotyledonae	trimerous / tetramerous / pentamerous
Commelinidae	trimerous
Pentapetalae	pentamerous
Superasteridae	pentamerous
Asteridae	pentamerous
Lamiidae	pentamerous
Campanulidae	pentamerous
Superrosidae	pentamerous
Rosidae	pentamerous
Malvidae	pentamerous
Fabidae	pentamerous

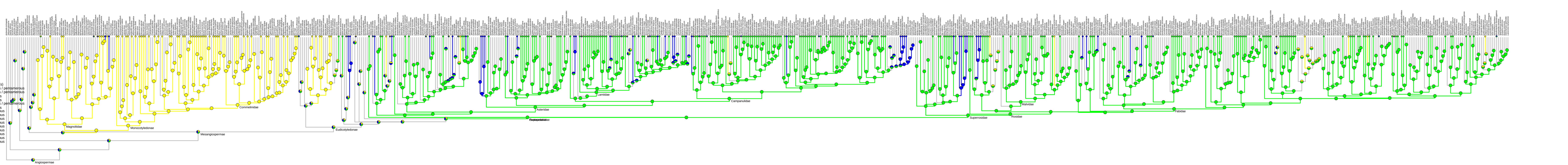

ML ancestral state reconstruction using rayDISC (R:corHMM)
332_B. Androecium structural merism (3−state) (D2c), ARDeq model

- trimerous
- tetramerous
- pentamerous

Node	ML state	Prob
Angiospermae	trimerous	0.9999
Mesangiospermae	trimerous	0.9999
Magnoliidae	trimerous	1
Monocotyledonae	trimerous	1
Eudicotyledonae	trimerous	0.9043
Commelinidae	trimerous	1
Pentapetalae	tetramerous	1
Superasteridae	tetramerous	1
Asteridae	tetramerous	1
Lamiidae	tetramerous	0.9839
Campanulidae	tetramerous	0.89
Superrosidae	tetramerous	0.9997
Rosidae	tetramerous	0.9975
Malvidae	pentamerous	0.6889
Fabidae	tetramerous	0.9084

Model	LogL	Npar	AIC	AICc	DeltaAICc	w	q01	...
ARD	−171.34	6	354.69	[illegible]	2	0.24	9e−04	···
ARDeq**	−170.34	6	352.69	352.8	0	0.66	9e−04	···
ER	−185.92	1	373.84	373.85	21.05	0	0.0011	···
SYM	−176.7	3	359.39	359.42	6.63	0.02	4e−04	···
SYMeq	−175.77	3	357.54	[illegible]	4.77	0.06	4e−04	···
ORD	−178.11	4	364.21	364.27	11.47	0	6e−04	···
ORDeq	−177.63	4	363.26	363.32	10.52	0	4e−04	···
ORDSYM	−185.37	2	374.74	374.75	21.96	0	0.0019	···
ORDSYMeq	−184.59	2	373.17	373.19	20.39	0	0.0018	···
ORDER	−186.18	1	374.37	374.37	21.58	0	0.0026	···

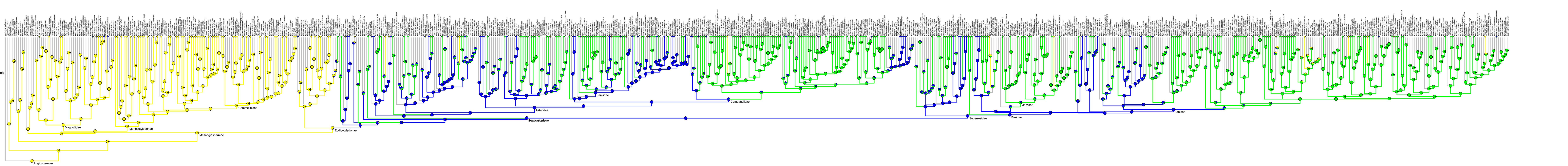

MP ancestral state reconstruction using ancestral.pars
(R:phangorn)
305_A. Filament (binary) (D2d), 55 steps

- laminar (wide)
- typical (narrow)

Node	MP state(s)
Angiospermae	laminar (wide) / typical (narrow)
Mesangiospermae	laminar (wide) / typical (narrow)
Magnoliidae	laminar (wide)
Monocotyledonae	typical (narrow)
Eudicotyledonae	typical (narrow)
Commelinidae	laminar (wide) / typical (narrow)
Pentapetalae	typical (narrow)
Superasteridae	typical (narrow)
Asteridae	typical (narrow)
Lamiidae	typical (narrow)
Campanulidae	typical (narrow)
Superrosidae	typical (narrow)
Rosidae	typical (narrow)
Malvidae	typical (narrow)
Fabidae	typical (narrow)

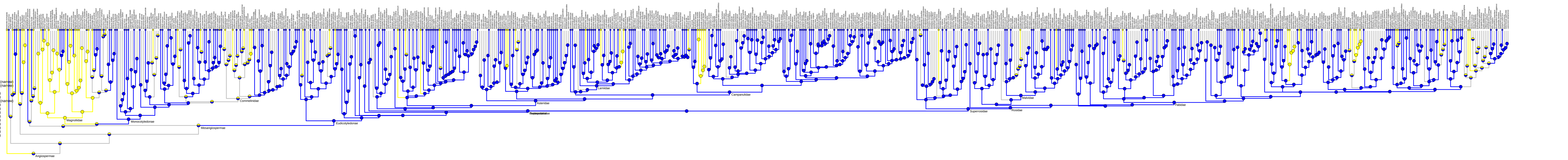

ML ancestral state reconstruction using rayDISC (R:corHMM)
305_A. Filament (binary) (D2d), ARDeq model

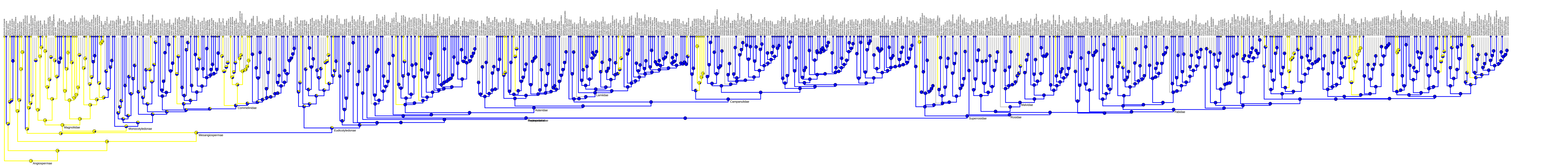

- laminar (wide)
- typical (narrow)

Node	ML state	Prob
Angiospermae	laminar (wide)	0.9954
Mesangiospermae	laminar (wide)	0.8659
Magnoliidae	laminar (wide)	0.9606
Monocotyledonae	laminar (wide)	0.575
Eudicotyledonae	typical (narrow)	0.6082
Commelinidae	typical (narrow)	0.7223
Pentapetalae	typical (narrow)	0.9989
Superasteridae	typical (narrow)	0.9989
Asteridae	typical (narrow)	0.9995
Lamiidae	typical (narrow)	0.9986
Campanulidae	typical (narrow)	0.9934
Superrosidae	typical (narrow)	0.9995
Rosidae	typical (narrow)	0.9996
Malvidae	typical (narrow)	0.9967
Fabidae	typical (narrow)	1

Model	LogL	Npar	AIC	AICc	DeltaAICc	w	q01	q10
ARD	−173.1	2	350.2	350.21	0.79	0.4	0.0075	0.0021
ARDeq**	−172.7	2	349.4	349.42	0	0.59	0.0077	0.0021
ER	−178.42	1	358.83	358.84	9.42	0.01	0.0023	0.0023
UNI01	−189.11	1	380.22	380.23	30.81	0	0.0134	
UNI10	−189.85	1	381.69	381.7	32.28	0		0.0022

MP ancestral state reconstruction using ancestral.pars
(R:phangorn)
311_A. Anther orientation (D2d), 109 steps

- introrse
- latrorse
- extrorse

Node	MP state(s)
Angiospermae	introrse
Mesangiospermae	introrse / extrorse
Magnoliidae	extrorse
Monocotyledonae	introrse
Eudicotyledonae	introrse / extrorse
Commelinidae	introrse
Pentapetalae	introrse / latrorse
Superasteridae	introrse / latrorse
Asteridae	introrse
Lamiidae	introrse
Campanulidae	introrse
Superrosidae	introrse
Rosidae	introrse
Malvidae	introrse
Fabidae	introrse

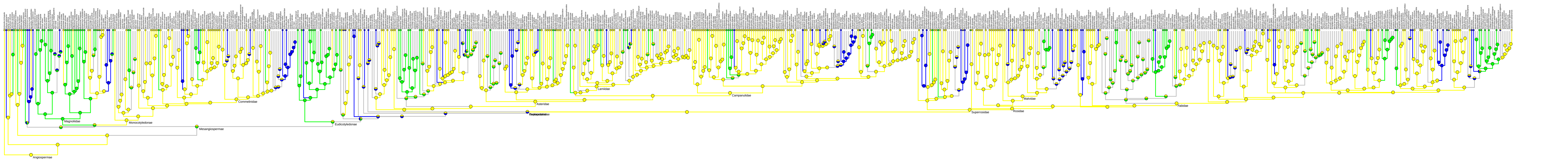

ML ancestral state reconstruction using rayDISC (R:corHMM)
311_A. Anther orientation (D2d), SYMeq model

- introrse
- latrorse
- extrorse

Node	ML state	Prob
Angiospermae	introrse	0.9986
Mesangiospermae	introrse	0.9711
Magnoliidae	extrorse	0.8449
Monocotyledonae	introrse	0.9677
Eudicotyledonae	introrse	0.9716
Commelinidae	introrse	0.9942
Pentapetalae	introrse	0.9999
Superasteridae	introrse	0.9999
Asteridae	introrse	0.9992
Lamiidae	introrse	0.9998
Campanulidae	introrse	1
Superrosidae	introrse	1
Rosidae	introrse	0.9999
Malvidae	introrse	0.9997
Fabidae	introrse	0.9992

Model	LogL	Npar	AIC	AICc	DeltaAICc	w	q01	
ARD	−345.25	6	702.5	702.61	1.91	0.12	0.0037	···
ARDeq	−345.52	6	703.04	703.15	2.45	0.09	0.0022	···
ER	−349.35	1	700.71	700.71	0.01	0.32	0.0026	···
SYM	−348.13	3	702.26	702.3	1.6	0.14	0.0029	···
SYMeq*	−347.33	3	700.67	700.7	0	0.32	0.0029	···

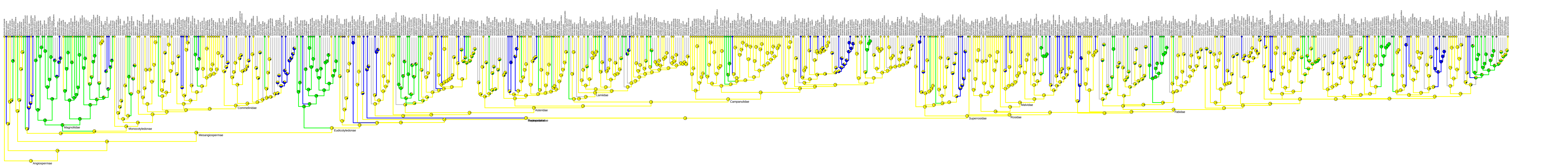

MP ancestral state reconstruction using ancestral.pars
(R:phangorn)
312_A. Anther attachment (binary) (D2d), 98 steps

- basifixed
- dorsifixed

Node	MP state(s)
Angiospermae	basifixed
Mesangiospermae	basifixed
Magnoliidae	basifixed
Monocotyledonae	basifixed
Eudicotyledonae	basifixed
Commelinidae	basifixed
Pentapetalae	basifixed
Superasteridae	basifixed
Asteridae	basifixed
Lamiidae	basifixed
Campanulidae	basifixed / dorsifixed
Superrosidae	basifixed
Rosidae	dorsifixed
Malvidae	dorsifixed
Fabidae	dorsifixed

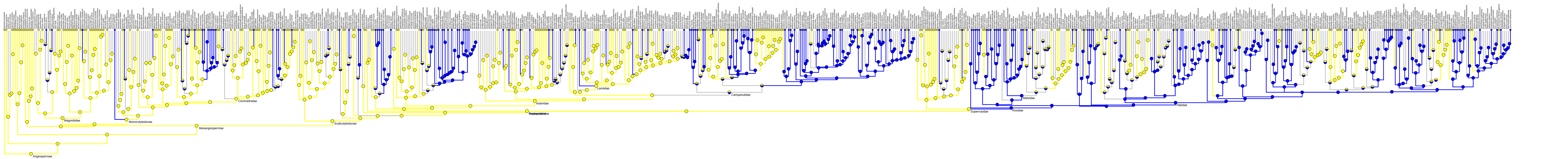

ML ancestral state reconstruction using rayDISC (R:corHMM)
312_A. Anther attachment (binary) (D2d), ARDeq model

- basifixed
- dorsifixed

Node	ML state	Prob
Angiospermae	basifixed	0.9811
Mesangiospermae	basifixed	0.9548
Magnoliidae	basifixed	0.9737
Monocotyledonae	basifixed	0.9514
Eudicotyledonae	basifixed	0.7772
Commelinidae	basifixed	0.953
Pentapetalae	dorsifixed	0.9184
Superasteridae	dorsifixed	0.9184
Asteridae	dorsifixed	0.9073
Lamiidae	dorsifixed	0.6674
Campanulidae	dorsifixed	0.9732
Superrosidae	dorsifixed	0.9312
Rosidae	dorsifixed	0.9626
Malvidae	dorsifixed	0.9615
Fabidae	dorsifixed	0.9995

Model	LogL	Npar	AIC	AICc	DeltaAICc	w	q01	q10
ARD	−264.58	2	533.16	533.17	0.58	0.39	0.0039	0.0074
ARDeq**	−264.29	2	532.57	532.59	0	0.52	0.004	0.0073
ER	−266.96	1	535.92	535.92	3.33	0.1	0.0053	0.0053
UNI01	−295.76	1	593.52	593.52	60.93	0	0.0057	
UNI10	−274.75	1	551.51	551.51	18.92	0		0.0075

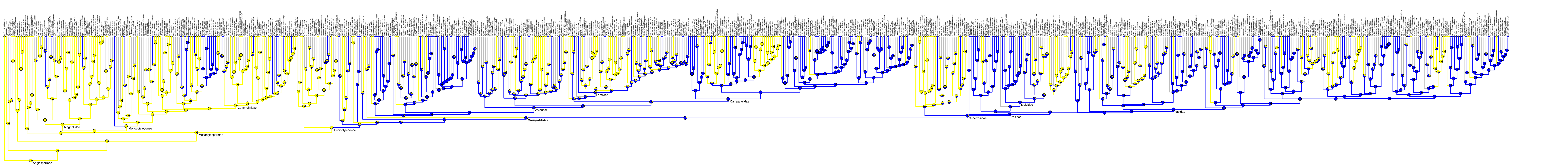

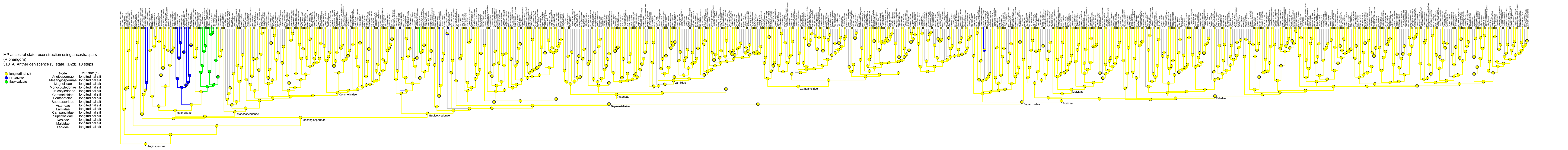
MP ancestral state reconstruction using ancestral.pars
(R:phangorn)
313_A. Anther dehiscence (3−state) (D2d), 10 steps
longitudinal slit
H−valvate
flap−valvate
Node MP state(s)
Angiospermae longitudinal slit
Mesangiospermae longitudinal slit
Magnoliidae longitudinal slit
Monocotyledonae longitudinal slit
Eudicotyledonae longitudinal slit
Commelinidae longitudinal slit
Pentapetalae longitudinal slit
Superasteridae longitudinal slit
Asteridae longitudinal slit
Lamiidae longitudinal slit
Campanulidae longitudinal slit
Superrosidae longitudinal slit
Rosidae longitudinal slit
Malvidae longitudinal slit
Fabidae longitudinal slit
Magnoliidae
Monocotyledonae
Commelinidae
Mesangiospermae
Eudicotyledonae
Asteridae
Lamiidae
Campanulidae
Superrosidae
Rosidae
Malvidae
Fabidae
Angiospermae

ML ancestral state reconstruction using rayDISC (R:corHMM)
313_A. Anther dehiscence (3−state) (D2d), ARDeq model

- longitudinal slit
- H−valvate
- flap−valvate

Node	ML state	Prob
Angiospermae	longitudinal slit	1
Mesangiospermae	longitudinal slit	1
Magnoliidae	longitudinal slit	0.9914
Monocotyledonae	longitudinal slit	1
Eudicotyledonae	longitudinal slit	1
Commelinidae	longitudinal slit	1
Pentapetalae	longitudinal slit	1
Superasteridae	longitudinal slit	1
Asteridae	longitudinal slit	1
Lamiidae	longitudinal slit	1
Campanulidae	longitudinal slit	1
Superrosidae	longitudinal slit	1
Rosidae	longitudinal slit	1
Malvidae	longitudinal slit	1
Fabidae	longitudinal slit	1

Model	LogL	Npar	AIC	AICc	DeltaAICc	w	q01	⋯
ARD	−55.29	6	122.59	122.69	2.07	0.14	2e−04	⋯
ARDeq*	−54.26	6	120.52	120.62	0	0.39	2e−04	⋯
ER	−60.04	1	122.08	122.09	1.46	0.19	1e−04	⋯
SYM	−59.03	3	124.05	124.08	3.46	0.07	2e−04	⋯
SYMeq	−57.93	3	121.85	121.89	1.26	0.21	2e−04	⋯

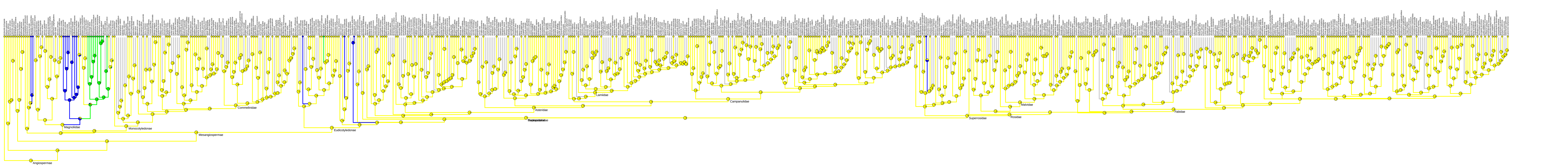

MP ancestral state reconstruction using ancestral.pars (R:phangorn)
401_B. Number of structural carpels (5−state) (D2c), 187 steps

- one (1)
- two (2)
- three (3)
- four or five (4−5)
- more than five (>5)

Node	MP state(s)
Angiospermae	more than five (>5)
Mesangiospermae	more than five (>5)
Magnoliidae	more than five (>5)
Monocotyledonae	three (3)
Eudicotyledonae	more than five (>5)
Commelinidae	three (3)
Pentapetalae	three (3) / four or five (4−5)
Superasteridae	three (3) / four or five (4−5)
Asteridae	two (2) / four or five (4−5)
Lamiidae	two (2)
Campanulidae	two (2)
Superrosidae	three (3) / four or five (4−5)
Rosidae	three (3) / four or five (4−5)
Malvidae	three (3) / four or five (4−5)
Fabidae	four or five (4−5)

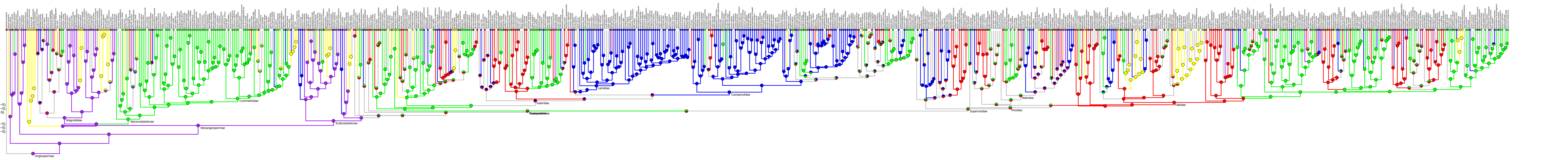

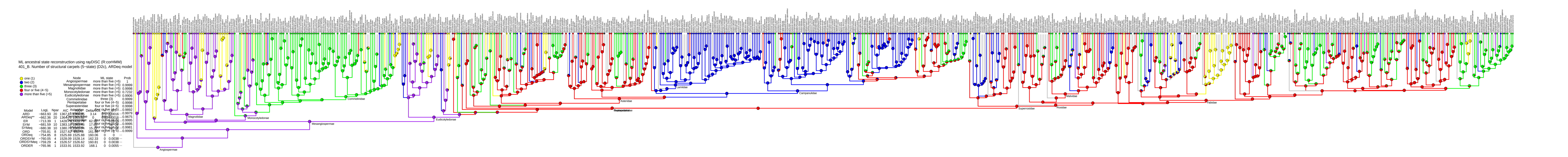

ML ancestral state reconstruction using rayDISC (R:corHMM)
401_B. Number of structural carpels (5−state) (D2c), ARDeq model
one (1)
two (2)
three (3)
four or five (4−5)
more than five (>5)
Node ML state Prob
Angiospermae more than five (>5) 1
Mesangiospermae more than five (>5) 0.9999
Magnoliidae more than five (>5) 0.9998
Monocotyledonae more than five (>5) 0.7232
Eudicotyledonae more than five (>5) 0.9906
Commelinidae three (3) 0.9999
Pentapetalae four or five (4−5) 0.9998
Superasteridae four or five (4−5) 0.9998
Asteridae four or five (4−5) 0.9892
Lamiidae two (2) 0.9979
Campanulidae two (2) 0.9675
Superrosidae four or five (4−5) 0.9995
Rosidae four or five (4−5) 0.9995
Malvidae four or five (4−5) 0.9981
Fabidae four or five (4−5) 0.9999
Model LogL Npar AIC AICc DeltaAICc w q01 ...
ARD −663.93 20 1367.87 1368.95 3.14 0.17 0.0016 ...
ARDeq** −662.36 20 1364.73 1365.82 0 0.83 0.0016 ...
ER −713.39 1 1428.79 1428.79 62.98 0 0.0015 ...
SYM −681.59 10 1383.18 1383.46 17.65 0 9e−04 ...
SYMeq −680.38 10 1380.76 1381.04 15.22 0 8e−04 ...
ORD −755.81 8 1527.62 1527.8 161.98 0 0 ...
ORDeq −754.85 8 1525.69 1525.88 160.06 0 0 ...
ORDSYM −760.05 4 1528.09 1528.14 162.33 0 0.0038 ...
ORDSYMeq −759.29 4 1526.57 1526.62 160.81 0 0.0038 ...
ORDER −765.96 1 1533.91 1533.92 168.1 0 0.0055 ...
Angiospermae
Mesangiospermae
Magnoliidae
Monocotyledonae
Commelinidae
Eudicotyledonae
Lamiidae
Campanulidae
Asteridae
Superrosidae
Rosidae
Malvidae
Fabidae

MP ancestral state reconstruction using ancestral.pars
(R:phangorn)
400_A. Gynoecium phyllotaxy (D2d), 10 steps

- whorled
- spiral

Node	MP state(s)
Angiospermae	whorled / spiral
Mesangiospermae	whorled
Magnoliidae	whorled
Monocotyledonae	whorled
Eudicotyledonae	whorled
Commelinidae	whorled
Pentapetalae	whorled
Superasteridae	whorled
Asteridae	whorled
Lamiidae	whorled
Campanulidae	whorled
Superrosidae	whorled
Rosidae	whorled
Malvidae	whorled
Fabidae	whorled

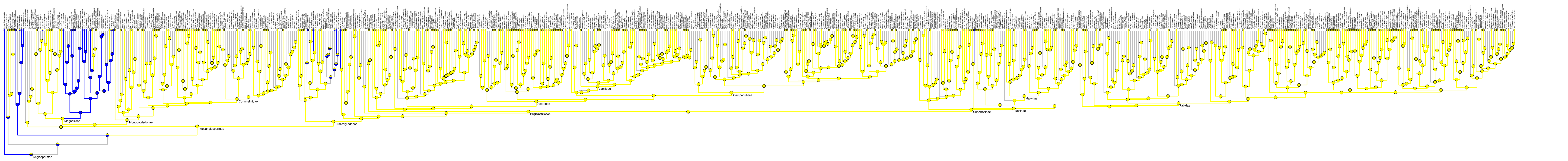

ML ancestral state reconstruction using rayDISC (R:corHMM)
400_A. Gynoecium phyllotaxy (D2d), ARDeq model

- whorled
- spiral

Node	ML state	Prob
Angiospermae	spiral	1
Mesangiospermae	spiral	0.9999
Magnoliidae	spiral	0.9991
Monocotyledonae	whorled	0.5674
Eudicotyledonae	spiral	0.9992
Commelinidae	whorled	0.9999
Pentapetalae	whorled	0.9753
Superasteridae	whorled	0.9753
Asteridae	whorled	1
Lamiidae	whorled	1
Campanulidae	whorled	1
Superrosidae	whorled	0.9791
Rosidae	whorled	0.9792
Malvidae	whorled	1
Fabidae	whorled	1

Model	LogL	Npar	AIC	AICc	DeltaAICc	w	q01	q10
ARD	−39.19	2	82.38	82.4	1.39	0.31	1e−04	0.0069
ARDeq**	−38.5	2	80.99	81.01	0	0.62	1e−04	0.0069
ER	−50	1	102	102	20.99	0	4e−04	4e−04
UNI01	−51.85	1	105.7	105.7	24.69	0	4e−04	
UNI10	−41.79	1	85.58	85.58	4.57	0.06		0.0082

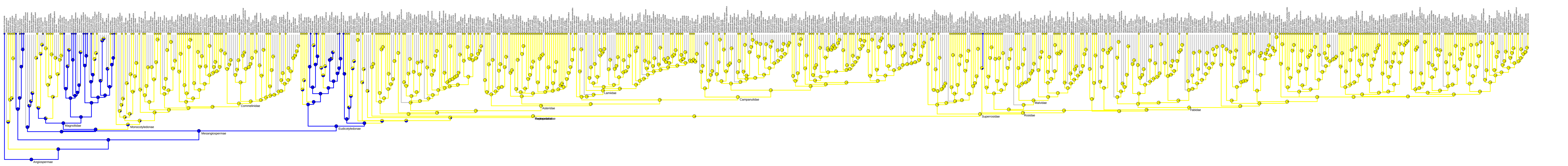

MP ancestral state reconstruction using ancestral.pars
(R:phangorn)
403_A. Fusion of ovaries (binary) (D2c), 24 steps

free (<5%)
fused (>5%)

Node	MP state(s)
Angiospermae	free (<5%) / fused (>5%)
Mesangiospermae	fused (>5%)
Magnoliidae	fused (>5%)
Monocotyledonae	fused (>5%)
Eudicotyledonae	fused (>5%)
Commelinidae	fused (>5%)
Pentapetalae	fused (>5%)
Superasteridae	fused (>5%)
Asteridae	fused (>5%)
Lamiidae	fused (>5%)
Campanulidae	fused (>5%)
Superrosidae	fused (>5%)
Rosidae	fused (>5%)
Malvidae	fused (>5%)
Fabidae	fused (>5%)

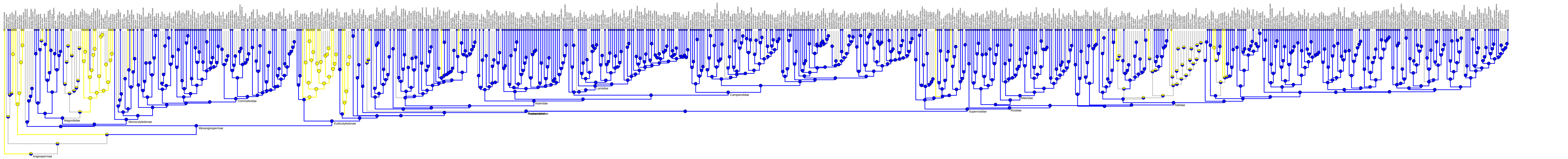

ML ancestral state reconstruction using rayDISC (R:corHMM)
403_A. Fusion of ovaries (binary) (D2c), ARDeq model

- free (<5%)
- fused (>5%)

Node	ML state	Prob
Angiospermae	free (<5%)	1
Mesangiospermae	free (<5%)	0.9998
Magnoliidae	free (<5%)	0.9991
Monocotyledonae	free (<5%)	0.9043
Eudicotyledonae	free (<5%)	0.9975
Commelinidae	fused (>5%)	0.9999
Pentapetalae	fused (>5%)	0.9864
Superasteridae	fused (>5%)	0.9864
Asteridae	fused (>5%)	1
Lamiidae	fused (>5%)	1
Campanulidae	fused (>5%)	1
Superrosidae	fused (>5%)	0.9986
Rosidae	fused (>5%)	0.9994
Malvidae	fused (>5%)	1
Fabidae	fused (>5%)	0.9986

Model	LogL	Npar	AIC	AICc	DeltaAICc	w	q01	q10
ARD	−94.59	2	193.19	193.2	1.38	0.33	0.0047	4e−04
ARDeq**	−93.9	2	191.81	191.82	0	0.65	0.0047	4e−04
ER	−100.62	1	203.23	203.24	11.42	0	7e−04	7e−04
UNI01	−98.42	1	198.84	198.85	7.02	0.02	0.0098	
UNI10	−105.87	1	213.73	213.74	21.91	0		8e−04

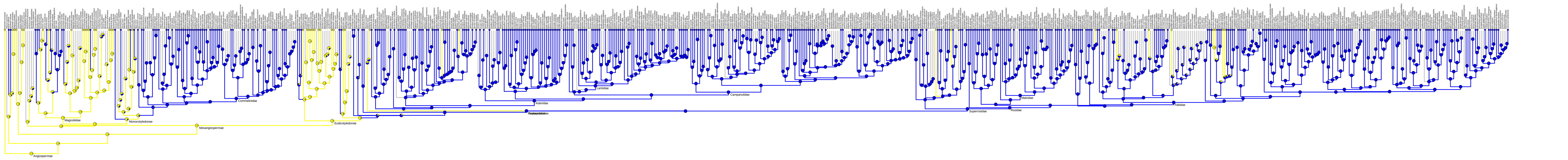

MP ancestral state reconstruction using ancestral.pars
(R:phangorn)
411_A. Number of ovules per functional carpel (3−state) (D2c), 119 steps

- one (1)
- two (2)
- three or more (>2)

Node	MP state(s)
Angiospermae	one (1)
Mesangiospermae	one (1) / three or more (>2)
Magnoliidae	one (1) / three or more (>2)
Monocotyledonae	one (1) / three or more (>2)
Eudicotyledonae	three or more (>2)
Commelinidae	one (1)
Pentapetalae	three or more (>2)
Superasteridae	three or more (>2)
Asteridae	one (1)
Lamiidae	one (1) / two (2) / three or more (>2)
Campanulidae	one (1)
Superrosidae	two (2) / three or more (>2)
Rosidae	two (2)
Malvidae	two (2)
Fabidae	two (2)

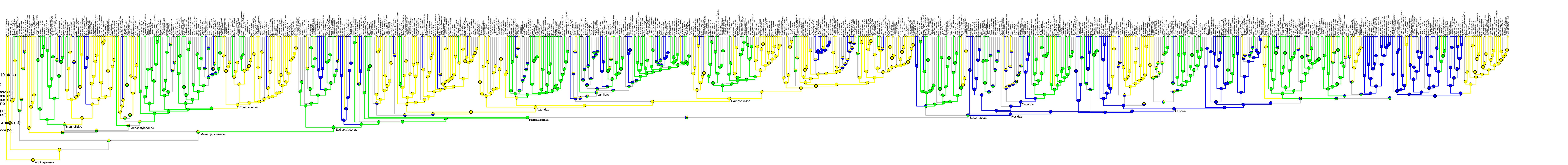

ML ancestral state reconstruction using rayDISC (R:corHMM)
411_A. Number of ovules per functional carpel (3−state) (D2c), ARDeq model

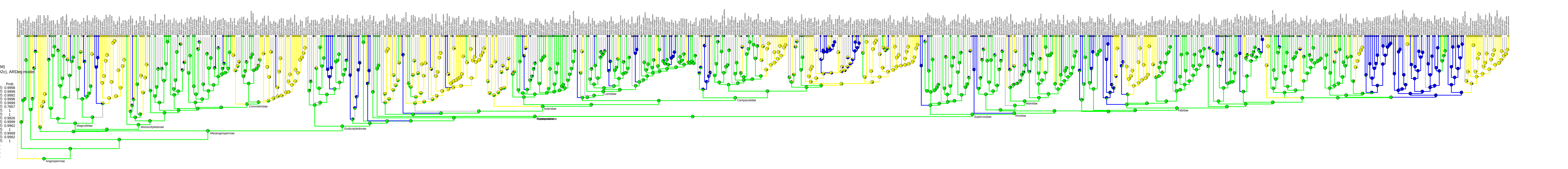

- one (1)
- two (2)
- three or more (>2)

Node	ML state	Prob
Angiospermae	three or more (>2)	0.9998
Mesangiospermae	three or more (>2)	0.9999
Magnoliidae	three or more (>2)	0.9992
Monocotyledonae	three or more (>2)	0.9996
Eudicotyledonae	three or more (>2)	0.9999
Commelinidae	three or more (>2)	0.7857
Pentapetalae	three or more (>2)	1
Superasteridae	three or more (>2)	1
Asteridae	three or more (>2)	0.9826
Lamiidae	three or more (>2)	0.9999
Campanulidae	three or more (>2)	0.9962
Superrosidae	three or more (>2)	1
Rosidae	three or more (>2)	0.9999
Malvidae	three or more (>2)	0.9992
Fabidae	three or more (>2)	1

Model	LogL	Npar	AIC	AICc	DeltaAICc	w	q01	
ARD	−386.52	6	785.05	785.15	1.88	0.28	0.0012	···
ARDeq**	−385.58	6	783.16	783.27	0	0.72	0.0012	···
ER	−399.73	1	801.46	801.47	18.2	0	0.0027	···
SYM	−399.12	3	804.25	804.28	21.01	0	0.0023	···
SYMeq	−398.54	3	803.09	803.12	19.85	0	0.0024	···
ORD	−395.26	4	798.52	798.57	15.3	0	0	···
ORDeq	−399.2	4	806.4	806.45	23.18	0	0.0019	···
ORDSYM	−410.96	2	825.92	825.94	42.67	0	0.0051	···
ORDSYMeq	−410.72	2	825.43	825.45	42.18	0	0.0051	···
ORDER	−412.02	1	826.04	826.04	42.77	0	0.0059	···
